# Exploring the Magnetic Landscape of Easily Exfoliable Two-Dimensional Materials

**DOI:** 10.1021/acsnano.5c16067

**Published:** 2026-04-28

**Authors:** Fatemeh Haddadi, Davide Campi, Flaviano José dos Santos, Nicolas Mounet, Louis Ponet, Nicola Marzari, Marco Gibertini

**Affiliations:** † Theory and Simulation of Materials (THEOS) and National Centre for Computational Design and Discovery of Novel Materials (MARVEL), 27218 École Polytechnique Fédérale de Lausanne (EPFL), CH-1015 Lausanne, Switzerland; ‡ Department of Materials Science, 9305 University of Milano-Bicocca, I-20125 Milano, Italy; § Laboratory for Materials Simulations (LMS) and National Centre for Computational Design and Discovery of Novel Materials (MARVEL), PSI Center for Scientific Computing, Theory and Data, Paul Scherrer Institut, CH-5232 Villigen PSI, Switzerland; ∥ 74350 Centro Brasileiro de Pesquisas Físicas, BR-22290-180 Rio de Janeiro, Brazil; ⊥ PSI Center for Scientific Computing, Theory and Data, Paul Scherrer Institute, CH-5232 Villigen PSI, Switzerland; # Theory of Condensed Matter, Cavendish Laboratory, University of Cambridge, CB3 0US Cambridge, U.K.; ∇ Dipartimento di Scienze Fisiche, Informatiche e Matematiche, 9306 University of Modena and Reggio Emilia, I-41125 Modena, Italy; ○ Centro S3, CNR-Istituto Nanoscienze, I-41125 Modena, Italy

**Keywords:** two-dimensional magnetic materials, hubbard corrections, global and local minima, energy landscape, density-functional theory, DFT + *U*

## Abstract

Magnetic materials often exhibit complex energy landscapes with multiple local minima, each corresponding to a self-consistent electronic structure solution. Finding the global minimum is challenging, and heuristic methods are not always guaranteed to succeed. Here, we apply a recently developed automated workflow to systematically explore the energy landscape of 194 magnetic monolayers obtained from the Materials Cloud two-dimensional structure database and determine their ground-state magnetic order. Our approach enables effective control and sampling of orbital occupation matrices, allowing rapid identification of local minima. We find a diverse set of self-consistent collinear metastable states, further enriched by Hubbard-corrected energy functionals, when the *U* parameters have been computed from first principles using linear-response theory. We categorize the monolayers by their magnetic ordering and highlight promising candidates. Our results include 109 ferromagnetic, 83 antiferromagnetic, and 2 altermagnetic monolayers, along with 12 novel ferromagnetic half-metals with potential for spintronics technologies.

## Graphical abstract

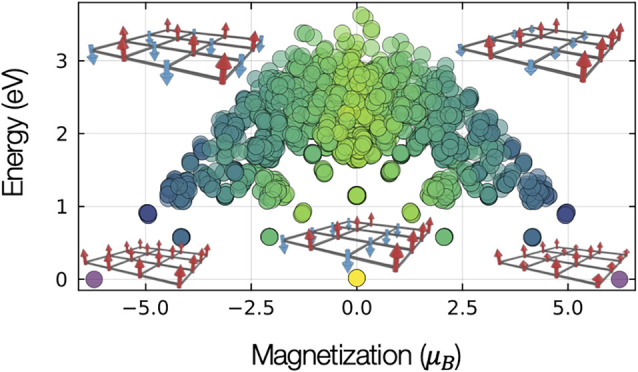

Magnetism in two-dimensional (2D) van der Waals (vdW) materials has garnered significant attention in recent years,^1–9^ not only for potential technological applications but also for the large variety of phenomena that can be hosted by magnetic 2D materials. Indeed, magnetism in 2D displays very different properties depending on the interplay between thermal fluctuations, exchange interactions, and magnetic anisotropy. The thrust to investigate this richness in experiments has stimulated an intense search for novel magnetic monolayers displaying specific phenomena, from Ising anisotropy,^10^ to Berezinskii-Kosterlitz-Thouless transitions,^11^ and heavy-fermion excitations.^12^

Although many candidates have been suggested by a long-term experience with layered magnetic materials, the continuous demand for novel 2D magnets calls for more systematic and exhaustive investigations. In this respect, theoretical approaches, particularly first-principles calculations based on density functional theory (DFT), provide a robust and high-throughput approach to predict novel 2D materials,^13–23^ including magnetic monolayers^13,24,25^ and their properties.^26,27^

Despite many successes, DFT can sometimes struggle to accurately predict the magnetic properties of materials, owing to the localized nature of d- and f-orbitals typically involved in magnetism and their tendency to suffer from self-interaction errors when adopting approximate exchange-correlation functionals. A computationally inexpensive and effective strategy to overcome these limitations is achieved by complementing approximate functionals with self-interaction corrections^28^ in the so-called Hubbard DFT + *U* approach.^29–31^ The Hubbard *U* parameter entering the DFT + *U* approach can be considered, somehow unsatisfactory, as a semiempirical parameter, or can be computed self-consistently, e.g., using linear-response theory.^32^ In the latter case, the theory preserves a first-principles character, and efficient implementations using density-functional perturbation theory (DFPT) are suitable for high-throughput investigations.^31–39^

Another crucial challenge for simulations is the identification of the ground state magnetic configuration of the system. Indeed, the magnetic energy landscape is typically complex, with several local minima associated with different magnetic states. The inclusion of Hubbard corrections, needed for a better description of magnetic systems, typically leads to a proliferation of local minima, making it even more challenging to identify the true ground state (global minimum).^40–49^ Several approaches have been developed to tackle this issue, ranging from efficiently generating different magnetic configurations,^50^ to the manipulation of the occupation matrix for d- or f-orbitals,^40,51–54^ to computing spin waves^55^ and spin spirals,^56^ employing cluster multipole theory,^57^ genetic algorithms^58^ or machine learning methods.^59^

In this work, we thus explore the magnetic properties of easily exfoliable 2D materials from the Materials Cloud two-dimensional structure database (MC2D)^13,14,60^ using our recently developed approaches to reveal novel magnetic monolayers. Starting from 3077 easily and potentially exfoliable monolayers (2004 easily and 1073 potentially exfoliable) with up to 40 atoms per cell,^13,14^ 877 systems with up to 12 atoms per cell are screened with plain DFT using an AiiDA^61,62^ workflow (Chronos).^13^ Out of these systems, 483 monolayers are found to be nonmagnetic, 166 systems were discarded because of failures in the optimization, and 228 monolayers showed a magnetic ground state. For these 228 materials, Hubbard corrections are then included for better accuracy, with the Hubbard parameter computed self-consistently. To identify the magnetic ground state, we employ the Robust Occupation Matrix Energy Optimization (RomeoDFT) algorithm^49^ based on constraining the atomic orbital occupation matrices that allows us to systematically explore the magnetic energy landscape. With this protocol, we identify 194 magnetic 2D materials for which we accurately determine the magnetic ground state and investigate electronic and magnetic properties, including a proxy for the critical temperature. Notably, we identify 12 half-metals, a class of materials with spin-dependent conductivity, where electrons in one spin channel (up or down) are metallic, while those in the opposite channel are insulating, which is especially promising for spintronics applications. The remaining 34 systems were discarded because either they were repeated, found nonmagnetic, consistently failed in the calculation of the Hubbard parameter, or not enough minima were found using RomeoDFT (see Supporting Information).

## Results and Discussions

To identify magnetic 2D materials and their magnetic ground state, we consider monolayers from MC2D,^60^ a portfolio of 2D materials that can be exfoliated from experimentally known 3D parent compounds, identified using high-throughput computational exfoliation.^13,14^ We start from 877 easily exfoliable 2D materials with up to 12 atoms per unit cell. As summarized in Figure 1, the tendency to show magnetism is first tested using an improved version of the AiiDA^61,62^ workflow, named Chronos, originally adopted in ref 13 to screen a smaller set of materials. For each system, the workflow first considers several ferromagnetic configurations through different starting magnetizations on the atoms and computes the optimized geometry and total energy through collinear DFT calculations with an ordinary approximate exchange-correlation functional (PBE) that is based not only on the local electron density but also on its gradient.^63^ If any ferromagnetic state is found to have a total energy lower than a nonmagnetic reference calculation, the system is considered potentially magnetic and additional magnetic configurations are investigated, including antiferromagnetic states that require up to a 2 × 1 supercell to accommodate two magnetic atoms with opposite spins (see Methods section for more details). Although this protocol may overlook materials where approximate exchangecorrelation functionals (such as PBE) do not favor a magnetic state, we expect these cases to be rare. In this way, 228 magnetic monolayers are identified with up to 12 atoms in their primitive cell, together with their potential collinear ground state. Out of these, 174 are found to be ferromagnetic, and further broken down into 100 metals and 74 insulators. The remaining 54 materials show an AFM ground state, with 31 having a finite gap and 23 being metallic.

**Figure 1:**
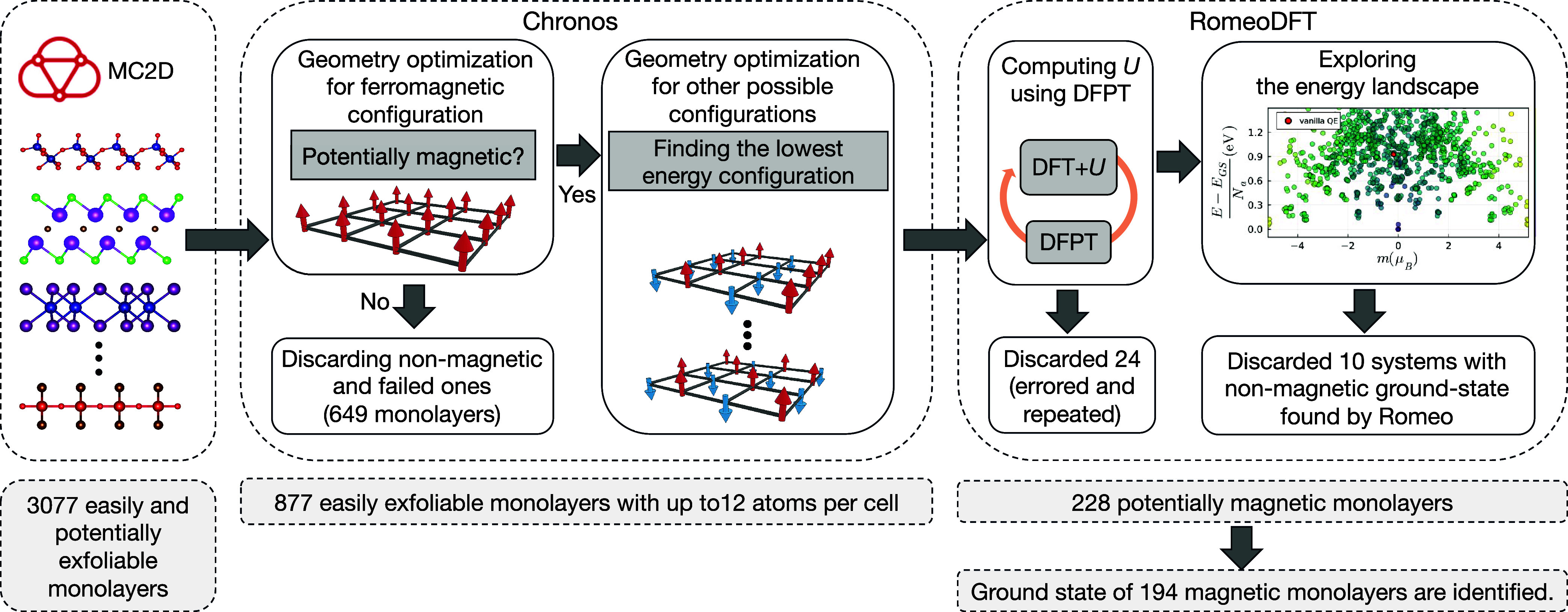
Workflow for identifying the ground state of magnetic monolayers in this work that is done in two main steps. In the first step, 877 easily exfoliable monolayers from MC2D^13,14,60^ are screened. Materials are identified as potentially magnetic if the total energy of the optimized geometry in the ferromagnetic ordering is smaller than the nonmagnetic state. Then, more magnetic configurations, including antiferromagnetic ordering, are explored, making sure there are two magnetic atoms in the unit cell to accommodate antiferromagnetic ordering and the geometry is optimized starting from different starting magnetization (defined by the spin density). 228 magnetic monolayers with up to 12 atoms per unit cell are identified and go to the next step. The Hubbard *U* is computed self-consistently in three consecutive steps using linear-response theory within DFPT. Twenty-four systems that are repeated or failed in the computation of Hubbard parameters are discarded. After that, the geometry and Hubbard *U* are fixed, and the energy landscape is explored by constraining the occupation matrix of d or f orbitals and calculating the total energy of different minima of the energy curvature. The ground state is then the minimum with the lowest energy. Ten systems that are found nonmagnetic at this step are discarded.

Although versatile and flexible, the workflow explores possible magnetic states only through the starting magnetization, that is, through initial conditions on the density of spin up and spin down states on each atom. A more robust way is to initialize from the atomic orbital occupation matrices—particularly d and f orbitals where the magnetization usually stems from—and explore more possible states. Introducing atomic orbitals φ_*m*_1__^I^(**r**) labeled by *m* on the *I*-th atom, we define the atomic occupation matrix as *n*_*m*_1_ *m*_2__^*IJσ*^ = ∑_*v* **k**_ *f*_*v* **k**σ_⟨ψ_*v* **k**σ_|φ_*m*_2__^*J*^⟩ ⟨φ_*m*_1__^*I*^|ψ_*v* **k**σ_⟩, where *f*_*v* **k**σ_ are the occupations of Bloch states |ψ_*v* **k**σ_⟩ and magnetization is an imbalance between the occupation of spin-up and down states *m* ^*I*^ = ∑_*m*_ (*n*_*mm*_^*I*↑^ – *n*_*mm*_^*I*↓^). To explore more systematically the magnetic energy landscape and obtain a more reliable identification of the magnetic ground state, it is important to gain full control over the atomic occupation matrix (at least for the relevant atomic orbitals on the magnetic atoms), not just through the spin density.^40,51–54^ We thus validate the identification of the magnetic ground state by applying a constraint on the atomic orbital occupations and then letting the self-consistent procedure find the closest local minimum in the energy landscape. The overall global minimum corresponding to the magnetic ground state can then be found by exhaustively exploring possible atomic occupations and comparing the energies of the corresponding self-consistent solutions. To perform this task automatically and efficiently, we use the RomeoDFT workflow^49^ (see Methods section for more details). We first consider a subgroup of magnetic monolayers and find that without Hubbard corrections in essentially all cases the same ground state is found either by controlling the starting magnetization by spin density or the constraints on the full atomic occupation matrix (see Figure S1 in Supporting Information). When considering PBE calculations, the search implemented in the Chronos workflow is thus exhaustive enough to reproduce the more sophisticated exploration performed by RomeoDFT.

Most of the identified 2D magnetic materials host electrons in localized d or f orbitals, for which approximate DFT functionals such as PBE tend to suffer from inaccuracies arising from self-interaction.^28,64^ The magnetic ground-state predictions might be affected by such errors and need to be validated against more accurate calculations. As alluded to before, an approach to mitigate self-interaction problems, known as DFT + *U*, relies on complementing DFT functionals with terms inspired by the Hubbard model.^29,31,65–71^ The Hubbard functionals that have a form as in eq 2 are added to the DFT energy functional and remove self-interaction errors when the Hubbard *U* is calculated from DFPT.^32^ Unlike empirical methods that are based on determining the *U* value to reproduce experimental results (such as band gap, lattice constant, etc.), DFPT determines the *U* self-consistently from linear response theory and restores the piece-wise linear behavior of the total energy with respect to the number of electrons in the Hubbard manifold. This method has proven effective for systems with localized, partially filled d or f orbitals, such as transition-metal and rare-earth compounds. Unfortunately, the inclusion of Hubbard corrections makes the energy landscape much more complex, with the appearance of many emergent local minima with similar total energies.^49^ To illustrate this effect, we consider the case of monolayer CoO_2_ in Figure 2, where each point represents a local minimum in the energy landscape, identified by exploring multiple occupation matrices. At the PBE level (*U* = 0), only a limited set of local minima is found, with an overall ferromagnetic ground state (indicated by a star). When including Hubbard corrections, with the Hubbard *U* calculated self-consistently from the linear-response method (PBE + *U*_sc_), the number of local minima significantly increases, making the magnetic energy landscape more complex and the possibility of falling in a local minimum different from the true ground state higher. We thus have that, while at the PBE level (*U* = 0) the number of states is limited and the identification of the global ground-state minimum is simpler (explaining the agreement between Chronos and RomeoDFT at the PBE level—see Figure S1 in the Supporting Information), an approach based on the occupation matrix is mandatory when Hubbard corrections are included. In Figure 2 we also show results for an intermediate case with *U* = 4 eV, where the proliferation of local minima is already present, although less dramatic than at *U*_sc_. Remarkably, the ground state, labeled by a star in each panel, is very sensitive to the value of *U* as the system is predicted to be ferromagnetic at *U* = 0, antiferromagnetic at 4 eV, and then ferromagnetic again (but with a different magnetization) at *U*_sc_ = 8.2 eV.^72^ Therefore, the choice of *U* strongly affects the ground-state properties of the system. To avoid any empiricism, we compute the Hubbard parameter *U* for each material using an efficient formulation of the linear-response approach^32^ within density-functional perturbation theory.^34–36^ As expected, our results confirm that the value of *U* for a given element can vary depending on its chemical environment, oxidation state, and local coordination (see Methods section and Supporting Information for more details).

**Figure 2:**
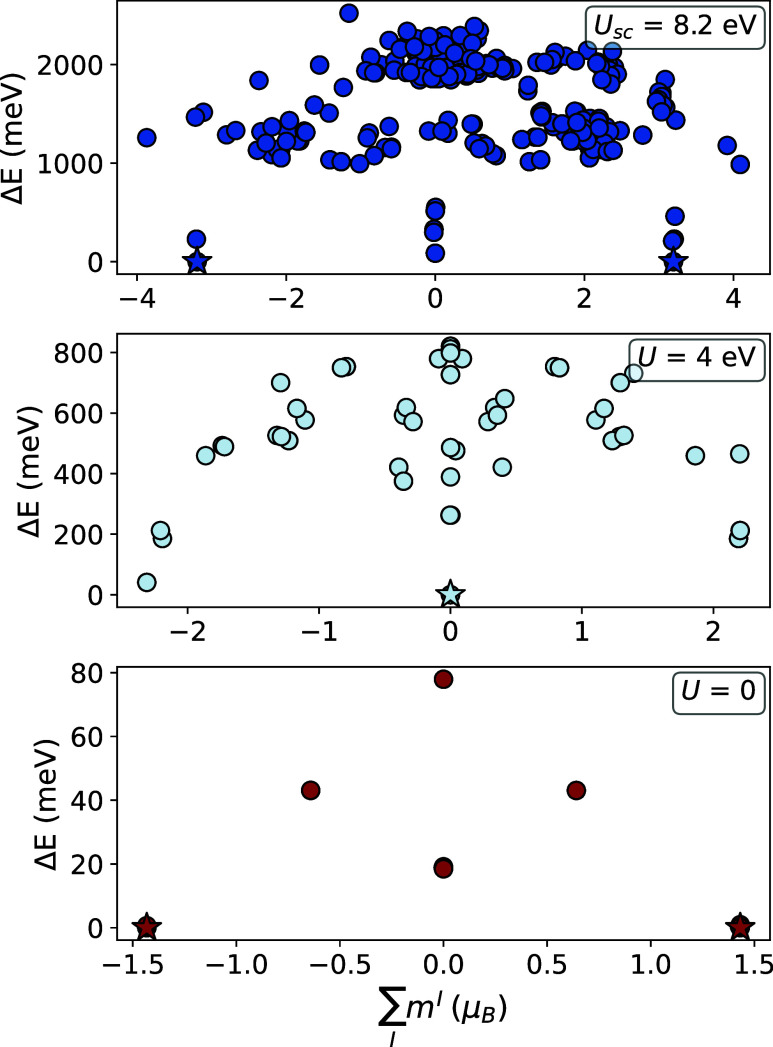
Self-consistent states for monolayer CoO_2_ from PBE + *U* calculations with different values of *U* with the star denoting the ground state identified by the lowest energy. There are six atoms in the unit cell to accommodate AFM ordering. *U*_*sc*_ shows the *U* value that is calculated by DFPT. At the PBE level (*U* = 0) and at PBE + *U*_*sc*_ the system is ferromagnetic with different values of magnetic moments. For an intermediate value of *U* at 4 eV the system exhibits antiferromagnetism. Therefore, selecting the correct *U* value is crucial to find the correct magnetic ordering, and using a method to explore the energy landscape can help avoid converging to an incorrect state. At *U* = 0, the number of states is smaller, which means simpler methods such as Chronos can still effectively find the ground state (see Supporting Information for more details on the case with *U* = 0).

Equipped with self-consistent Hubbard corrections, we navigate the magnetic energy landscape of potentially 2D magnetic materials identified through the Chronos workflow at the PBE level, now using PBE + *U* and a control over the atomic occupation matrix using RomeoDFT. Out of 228 materials, 34 systems are discarded because either they are found to be nonmagnetic or there are convergence issues in the calculation of the Hubbard parameter or self-consistent calculations, leaving us with 194 magnetic monolayers for which we characterize their ground state. The results are summarized in Figure 3, where we report the absolute magnetization per magnetic atom and the band gap of the ground state of all systems, together with their distribution. More details on the specific magnetic configuration, crystal structure, magnetic energy landscape, and band structure for each material are provided in the Supporting Information.

**Figure 3:**
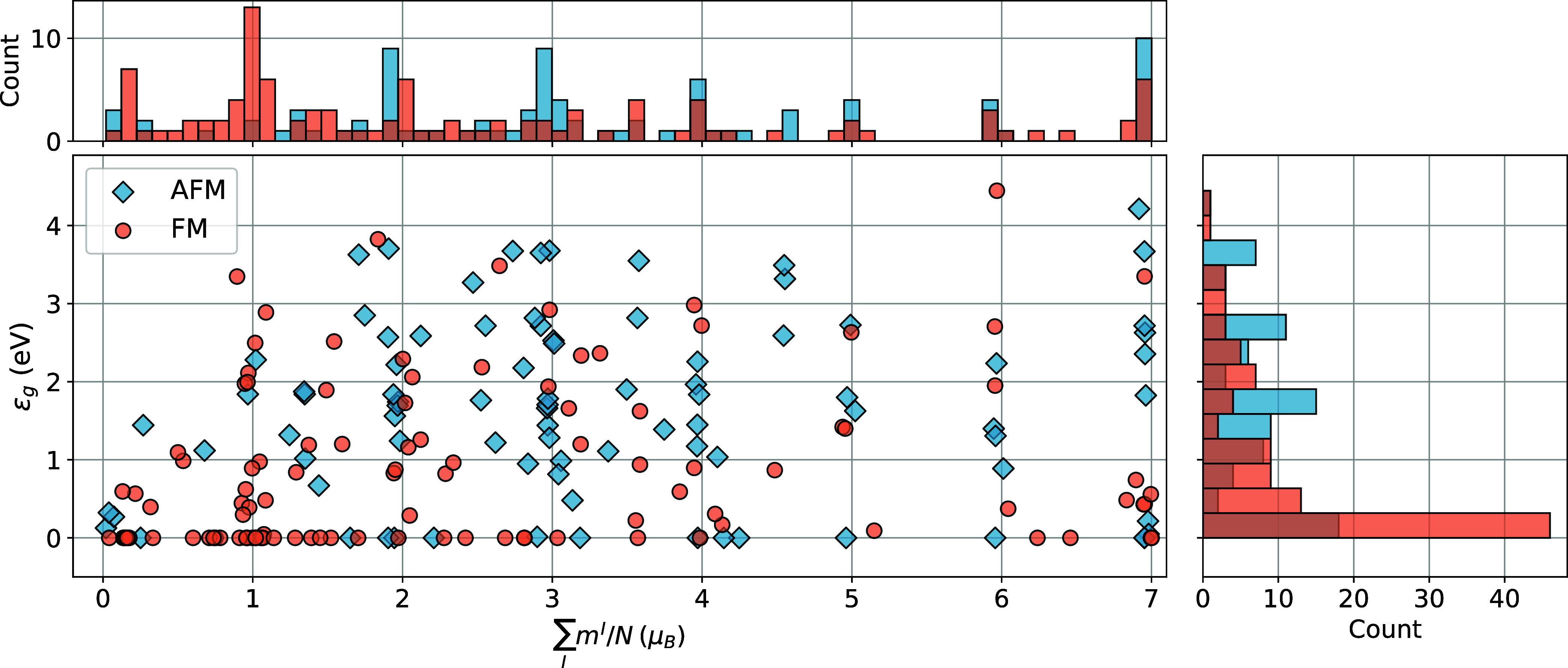
Band gap value, density of magnetic moments per atom, and their distribution for the ground state of the magnetic monolayers studied in this work obtained by exploring their energy landscape with PBE + *U* calculations. *N* is the number of magnetic atoms per cell, I is the index of the magnetic atoms and *m* ^*I*^ is the magnetization defined from occupation matrix (*m* ^*I*^ = ∑_*m*_ (*n*_*mm*_^*I*↑^ – *n*_*mm*_^*I*↓^), for more details see the Methods section). Orange and blue show ferromagnetic and antiferromagnetic systems, respectively.

We categorize all systems into four groups, depending on whether they are ferromagnetic or antiferromagnetic, insulators or metals (we did not find ferrimagnets, and the two altermagnets are included in the antiferromagnetic group). Figure 4 shows the number of systems in each group, with the left and right charts representing results from PBE (Chronos workflow) and PBE + *U* (RomeoDFT workflow) calculations, respectively. Blue and red colors indicate antiferromagnetic and ferromagnetic materials. The innermost rings show the number of systems in each category, while the second rings count metallic (hatched) or semiconducting (solid) systems. In the right chart, the outermost ring indicates the number of systems from the innermost ring that either remained in the same magnetic category (solid fill) or changed categories (dotted fill) from the PBE calculations. As expected, the inclusion of Hubbard corrections leads to an increase in the number of insulating systems. From Figure 4, we also note that using the RomeoDFT workflow with PBE + *U* calculations significantly expands the number of antiferromagnetic systems (blue colors): approximately 40% of these materials were identified as ferromagnetic when using the Chronos workflow with standard PBE calculations. This change can be attributed primarily to two factors: first, the limited capability to explore the energy landscape when using simply a starting magnetization; second, the possible increase in the number of magnetic states after including Hubbard corrections.

**Figure 4:**
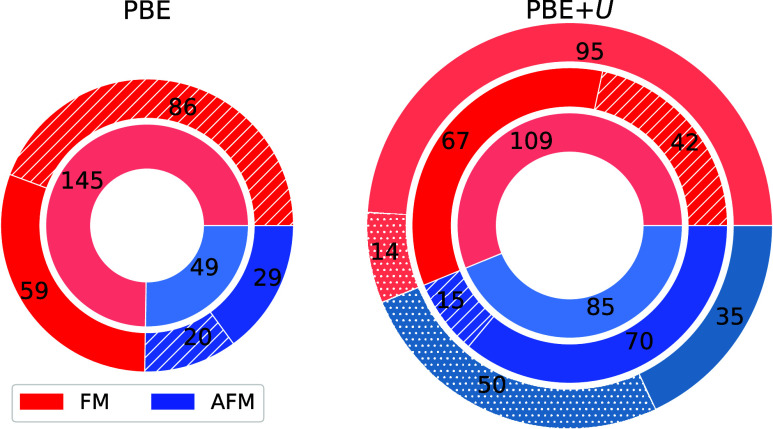
Breakdown of magnetic monolayers identified by two approaches: PBE calculations using Chronos workflow on the left and PBE + *U* calculations using RomeoDFT workflow on the right (see Figure 1). Blue indicates ferromagnetic systems, and red represents antiferromagnetic systems. The inner rings display the number of materials within each magnetic category. The second rings differentiate between metallic (dashed fill) and semiconducting (solid fill) systems for each category. The outermost ring on the right diagram indicates the number of systems that either remained in the same magnetic category as identified by the Chronos workflow (solid fill) or shifted categories (dotted fill). Note that the two altermagnets are considered in the antiferromagnetic category.

We note in passing that among the AFM identified above, we find only two altermagnetic monolayers by generalizing the protocol for 3D systems^73^ to include additional symmetry constraints in 2D.^74,75^ These are RuF_4_ and VF_4_, with the same structural prototype. Both systems were already predicted to be 2D altermagnets in ref 74; we do not see altermagnetism in FeBr_3_, because its crystal structure and symmetry is different from the one studied in ref 74. More details about how we identify these systems are given in the Supporting Information.

Due to the scarcity of experimental results for magnetic monolayers, it is challenging to validate our predictions against experiments. We thus focus on a few cases reported in the existing literature. For example, our results agree with the experimentally observed ferromagnetism in the family of chromium trihalides (CrX_3_; X = Cl, Br, I with CrCl_3_ being 2D-XY ferromagnetic)^10,11,76^ and in CrSBr.^77^ In the family of transition-metal phosphorus trisulfides, our results match the experimentally observed ferromagnetism in CrPS_4_ ^78^ and the antiferromagnetic ordering in NiPS_3_, although suppressed by fluctuations in the monolayer limit.^79^ For the case of FePS_3_, our findings show ferromagnetic ordering, in contrast with the antiferromagnetic ordering observed in experiments.^80^ We attribute this discrepancy to the limited size of the unit cell considered in this work for FePS_3_, which is not able to accommodate the measured zigzag ordering. Indeed, since the primitive cell already contains two magnetic atoms, no supercell is considered, and only a Néel AFM ordering is tested. This limitation, combined with the use of collinear calculations, could influence the accuracy in predicting other antiferromagnetic systems and may lead to discrepancies with other studies,^56,81^ and we expect it to be at the origin of the relatively small number of AFM systems with respect to FM. These results agree with ref 82 where calculations predict FePS_3_ to favor a ferromagnetic ground state when modeled using a triangular primitive cell, while NiPS_3_ is found to be antiferromagnetic. We attribute the different behavior of FePS_3_ and NiPS_3_ in the primitive cell to the fact that zigzag AFM order arises from a competition between the first nearest-neighbor (*J*_1_) and third nearest-neighbor (*J*_3_) exchange interactions that have opposite signs, with the former favoring ferromagnetism and the latter antiferromagnetism. In FePS_3_, *J*_1_ is comparable to *J*_3_ while in NiPS_3_ *J*_3_ is much stronger than *J*_1_, although they both give rise to zigzag AFM order when including the effect of second nearest-neighbor exchange (*J*_2_),^83,84^ which is not accessible in our primitive cell calculations. For CrGeTe_3_, our calculations predict a ferromagnetic ground state consistent with the observed trend in thicker layers^85,86^ and previous DFT calculations.^83,87,88^ We also note that for Ni dihalides (NiX_2_, X= Cl, Br, I) we find a ferromagnetic ground state. This is consistent with the FM character of the first nearest-neighbor exchange parameter reported in the literature,^89^ although the limited size of the supercell adopted does not capture the frustration arising from the competing interaction with third nearest-neighbors.^89^

Among the possible conclusions that we can draw from the high-throughput investigation of the magnetic ground-state of 2D materials, we also focus on the nature of magnetic elements in each structure. Conventional magnetism usually arises from d or f orbitals, qualifying transition metals and lanthanides as the dominant contributors to magnetism. Our results largely confirm such conventional wisdom, as shown in Figure 5, where we report the number of systems in which a given element is magnetic. However, Figure 5 also suggests that certain chalcogen and nonmetal elements (especially oxygen) also play a role in magnetism in 2D compounds, putting forward their potential role in unconventional magnetism. For instance, in GaSe (space group: *p*3̅*m*1) and SiN_2_F_6_, the selenium and nitrogen atoms exhibit non-negligible magnetic moments (0.2 μ_*B*_ and 2 μ_*B*_ respectively). Interestingly, in the case of CdOCl, while the d orbitals of Cd are fully occupied and exhibit negligible magnetic moments, magnetism predominantly arises from the oxygen atoms, which have a magnetic moment of 0.8 μ_*B*_.

**Figure 5:**
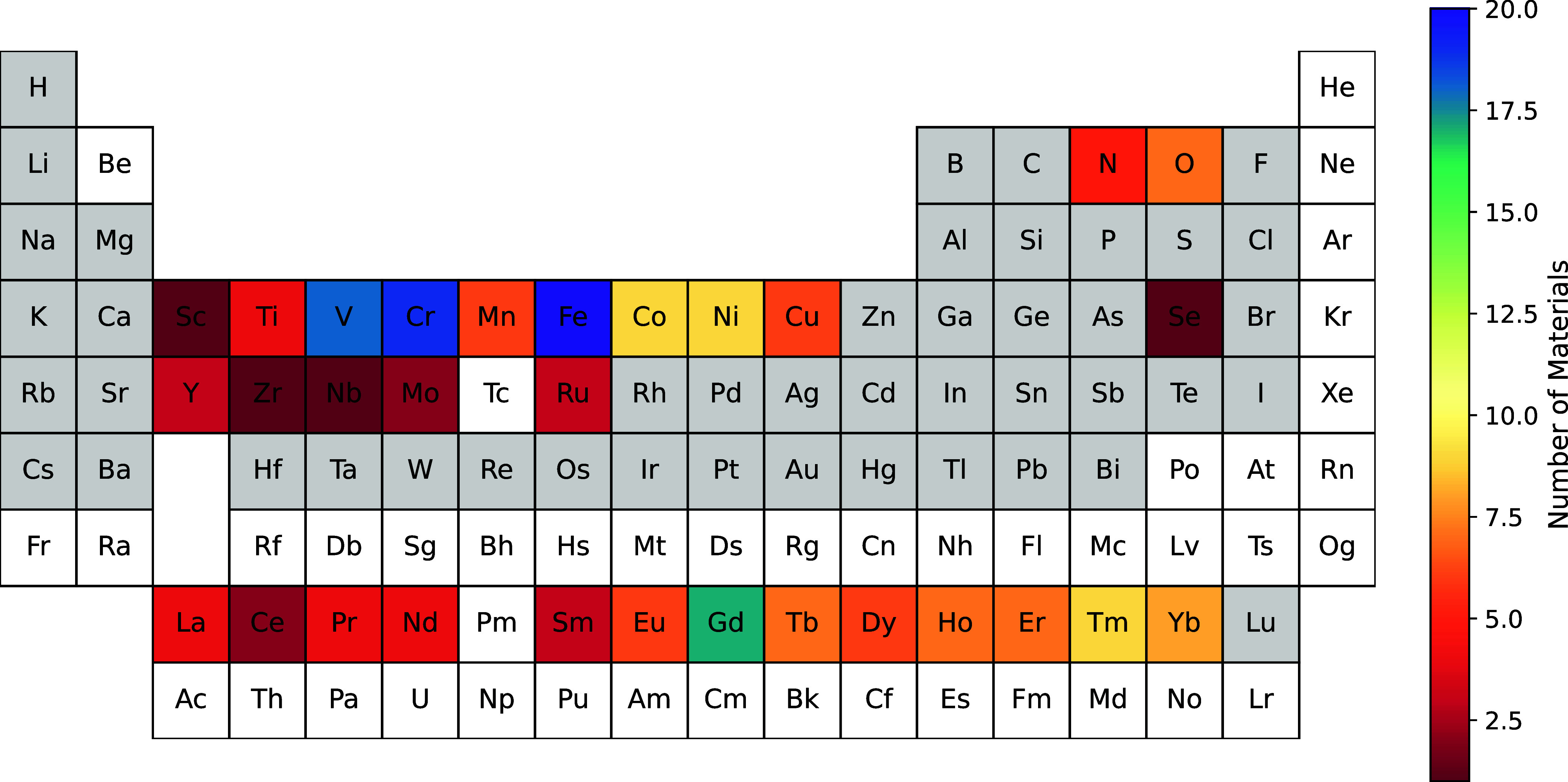
Periodic table displaying color intensity to indicate the number of magnetic monolayers in which each atom is magnetic. An atom is considered magnetic in a system if its magnetic moment exceeds 0.1 μ_*B*_ and it either (i) has the highest magnetic moment in the system or (ii) possesses at least 80% of the maximum magnetic moment in the system. Atoms with a white background are not present in the initial set of 877 systems considered in the Chronos workflow.

Exploring the energy landscape gives us access not only to the ground state of the system but also to the ability to quantify the energy difference between the ground state and other metastable magnetic states. This is particularly relevant when it is possible to identify a metastable state with magnetic moments on the atoms similar to the ground state but different magnetic ordering, e.g., a ferromagnetic ground state and a metastable antiferromagnetic state with comparable absolute magnetization (or vice versa). The energy difference between these states can then be translated into an effective exchange parameter *J̃* of an underlying isotropic Heisenberg model through:

1 $$\mathrm{J̃}=\frac{1}{2N}\left(E_{\mathrm{FM}}-E_{\mathrm{AFM}}\right)$$

where *N* is the number of magnetic atoms per cell while *E*_*FM*_ and *E*_*AFM*_ are the energies of the ferromagnetic and antiferromagnetic states, respectively. This effective isotropic exchange parameter *J̃* is related to an effective nearest-neighbor exchange coupling *J* through *J̃* = *nJ*, where *n* is the number of nearest neighbors. A large energy difference, |*E*_*FM*_ – *E*_*AFM*_|, corresponds to a large exchange parameter *J̃*, stabilizing the magnetic order and possibly resulting in a higher critical temperature. It is important to note that the mapping between first-principles calculations and the Heisenberg model is meaningful only when the magnetization arises from local atomic magnetic moments that have the same values (although different orientations) in the FM and AFM states, i.e., |S_*FM*_| = |S_*AFM*_|. This condition is typically met in insulators, while in general it cannot be readily applied to magnetic metals, where magnetism arises from itinerant electrons. Therefore, we consider only magnetic insulators to compute *E*_*FM*_ – *E*_*AFM*_ and the effective *J̃*.

Figure 6 shows |*E*_*FM*_ – *E*_*AFM*_| divided by the number of magnetic atoms per cell, *i.e*. *J̃*, for the insulating magnetic monolayers for which it was possible to find a metastable magnetic state with similar atomic magnetic moments but different ordering. The corresponding numerical values are provided in Table S1 of the Supporting Information. Systems are sorted with increasing effective *J̃*. The bottom panel contains systems where exchange interactions are weak, making it more challenging for the magnetic ordering to compete with thermal fluctuations. On the contrary, the systems in the top panel are expected to have higher critical transition temperatures. The ranking based on the effective *J̃* seems to reproduce several experimental trends for the ordering temperature. For instance, it is experimentally known that CrSBr has a higher critical temperature than the chromium trihalides,^10,77^ and within this family the iodine compound has the largest *T*_*c*_ while the chlorine one the smallest,^10,11,90–92^ in agreement with the ordering in Figure 6.

**Figure 6:**
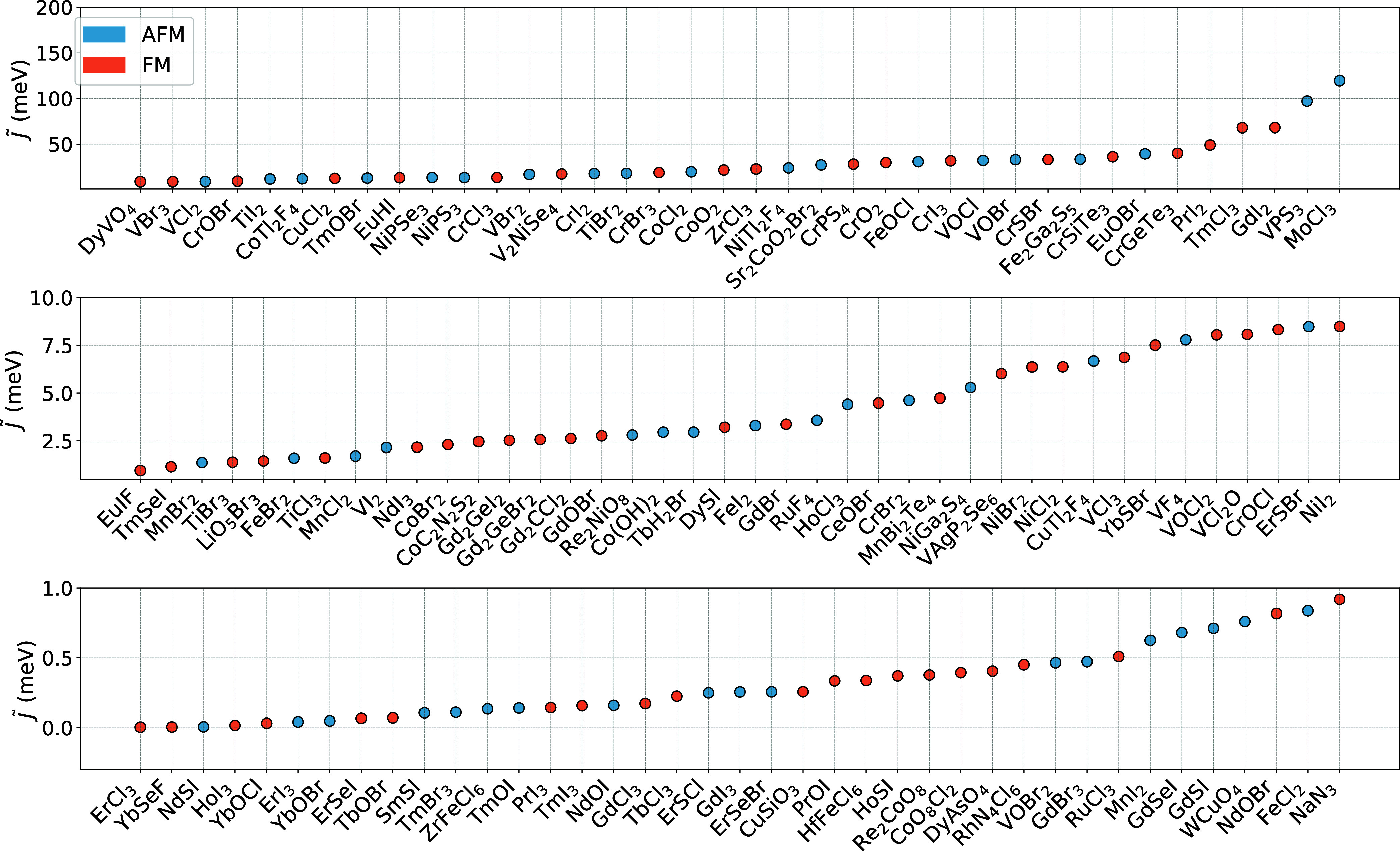
Effective exchange parameter, *J̃*, calculated from eq 1 using the energy difference between the ground state and the closest state with opposite magnetic configuration (with the condition of |*S*_*FM*_| = |*S*_*AFM*_|) obtained by exploring the energy landscape. Blue and orange circles represent antiferromagnetic and ferromagnetic ground states, respectively. The *y*-axis range progressively increases across the three subplots.

To further validate *J̃* as a meaningful proxy for *T*_*c*_, we select three systems with the largest *J* in Figure 6 and compute their transition temperatures using different approaches. We thus focus on MoCl_3_, GdI_2_, and VPS_3_, noting that the latter was already identified as a potentially high-*T*_*c*_ magnetic monolayer based on high-throughput calculations.^93^ Additionally, we include CrI_3_ in our analysis, as experimental results are available for comparison. In addition to the approach based on eq 1 and relying on the energy difference between FM and AFM reference states, we also consider the so-called four-state method,^94^ which calculates the exchange parameter based on the energy differences between four distinct magnetic configurations of a pair of neighboring atoms in a supercell (see Methods section for more details). Remarkably, the four-state method allows the calculation of specific interatomic exchange parameters, not just an effective nearest-neighbor *J* = *J̃*/*n* as in eq 1, and we consider interactions up to the third (fourth) nearest neighbors for CrI_3_ and GdI_2_ (VPS_3_ and MoCl_3_). The exchange parameters computed within the two approaches are then denoted *J* ^*FM–AFM*^ and *J* ^4*s*tates^ in the following. From the knowledge of the exchange parameters, the transition temperature is calculated using either mean-field (MF) theory or classical Monte Carlo (MC) simulations. In the latter, we completely neglect relativistic effects. Indeed, magnetocrystalline anisotropy does not affect significantly the critical temperature for a finite-size system, and even isotropic monolayers can sustain magnetic order at finite temperature when considering realistic sample sizes.^95^

Critical temperatures computed either using MF or MC, with exchange parameters estimated from both eq 1 and the four-state method, are shown in Figure 7 for CrI_3_, GdI_2_, VPS_3_, and MoCl_3_. Trends in *T*_*c*_ derived from both MC and MF methods are consistent with Figure 6, with MF calculations showing higher values, as expected from the well-known overestimation of *T*_*c*_ in mean-field theory.^96^ We note that, in general, different methods for computing the exchange parameters give similar values of *T*_*c*_. The only exception is MoCl_3_, for which the MC estimates of *T*_*c*_ differ significantly between the two methods, with a notably lower value when the exchange parameters are calculated using the four-state method. This discrepancy is attributed to the dimerization of Mo atoms in MoCl_3_, which distorts the otherwise honeycomb lattice of Mo atoms. The energy difference between the FM and AFM is mainly arising from a strong AFM coupling between dimerized pairs, while dimers are only weakly coupled. This feature is correctly captured within the four-state method, while the effective *J̃* approach of eq 1 uniformly distributes the FM-AFM energy difference between the *n* = 3 closest atoms. This failure also affects all MF estimates in a similar way. We thus expect that the effective *J̃* reported in Figure 6 provides a good estimation of the ranking in critical temperature, provided that exchange interactions are distributed evenly between similar neighbors.

**Figure 7:**
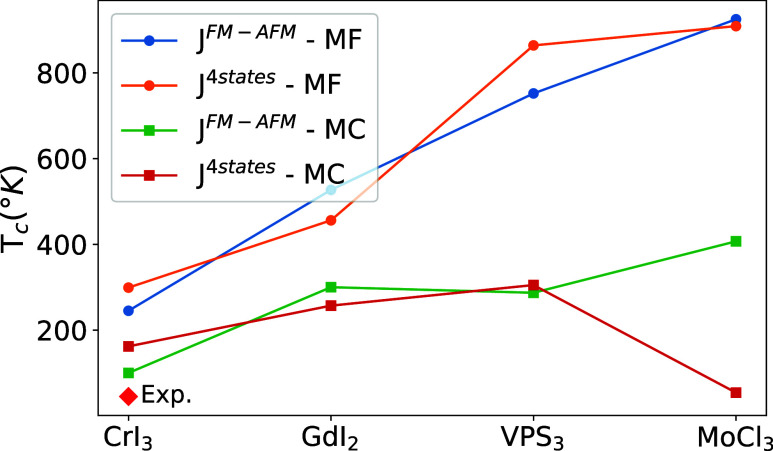
Transition temperature of three magnetic monolayers (GdI_2_, VPS_3_, and MoCl_3_) with the highest *J̃* in Figure 6 from two methods: mean-field (MF) and Monte Carlo (MC) calculations. The exchange parameters used in the calculation of T_*c*_ are obtained using two different approaches: 1- exploring the energy landscape and calculating the energy difference of the FM and AFM states (*J* ^*FM–AFM*^) as given in eq 1, and 2- four-states method (*J* ^4*s*tates^).

We also note that the transition temperature of the CrI_3_ monolayer is higher than the values reported in previous studies and experiments.^10,26,97^ This discrepancy can be attributed to differences in geometry, the relatively large *U* value for CrI_3_ obtained from linear-response,^39^ and the influence of broadening effects in Brillouin-zone integrations. For consistency, we employed the same broadening value as used in the Chronos workflow (270 meV). Since the exchange parameter is derived from total energies–which are sensitive to the choice of broadening–it is likewise influenced by the selected broadening value.

Among metallic systems, we are particularly interested in half-metallic ferromagnetic systems that are interesting for spintronic devices because they can provide fully spin-polarized currents.^98^ We thus search for systems where the Fermi energy crosses the energy bands for one spin channel while the other spin channel remains gapped.^99^ Twelve novel half-metals are identified, whose electronic structure is reported in Figure 8. In several cases the bandwidth is sufficiently large to expect spin-polarized transport even in the presence of disorder. Remarkably, in systems like CuO_2_, Cr_3_O_8_, EuOI, EuOBr_2_, VO_3_, VS_2_O_8_, YbBr_3_, and YbCl_3_ a large gap is present above the metallic spin channel, which is potentially interesting in view of possible optoelectronic control of spin currents. In all these cases a correct description of the magnetic ground state is crucial to have half-metallic properties. For instance, in the case of FeAl_2_S_4_ it is important to have the two iron atoms with different oxidation states (+1 and +2) and thus different magnetic moments of 2.2 μ_*B*_ and 3.2 μ_*B*_. We also stress that, in general, the half-metallic state can be fragile, especially if it is protected by symmetry-induced degeneracies.^100^ Further tests to include the effect of spin–orbit coupling or structural distortions would thus be needed to fully validate predictions, although the procedure outlined above is already sufficient to discard speculative half-metals such as iron dihalides FeX_2_ (with X = Cl, Br, I). These systems have been predicted to be half-metals,^101,102^ although experimental evidence^103–107^ suggests that they have a finite gap. We find them to be indeed insulating, although the mechanism to break the symmetry-induced degeneracy arises from an incorrect AFM configuration, as we do not include relativistic effects or distortions.

**Figure 8:**
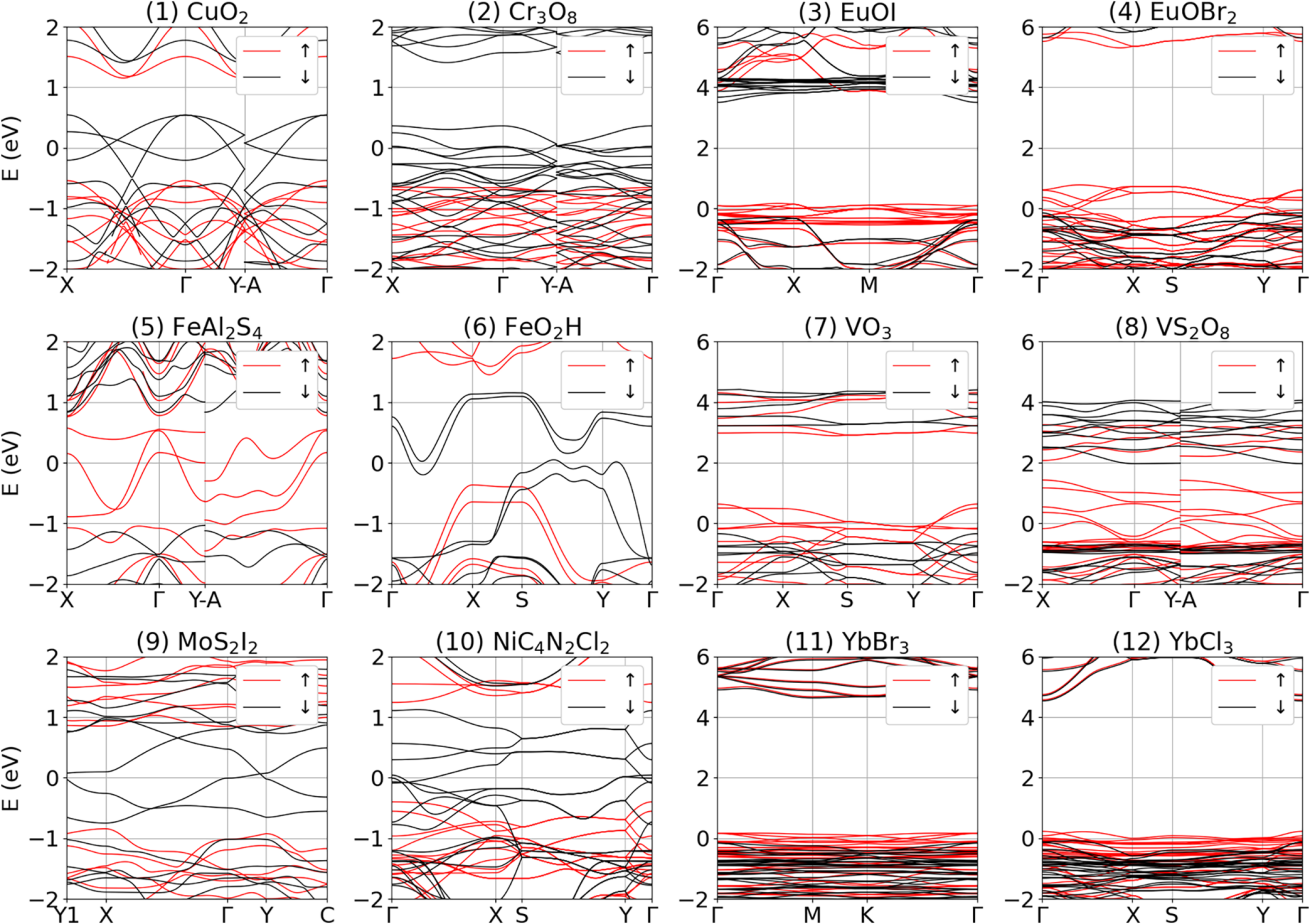
Electronic bands of half metals identified in this work. The Fermi energy is set at zero. Note that the two rightmost columns have a larger energy window to be able to show the band gap of the opposite spin channel.

## Conclusions

In conclusion, we investigate the magnetic ground states of easily exfoliable monolayers from the MC2D database. Starting from 3077 easily and potentially exfoliable monolayers identified in refs 13,14 ., an initial screening is carried out on 877 easily exfoliable systems containing up to 12 atoms per cell using high-throughput density functional theory with the conventional PBE exchange-correlation functional. Testing various magnetic configurations by changing the initial magnetization, 228 magnetic monolayers are identified as potentially magnetic systems. Subsequently, Hubbard corrections are introduced for these systems, with the Hubbard parameter determined self-consistently. The magnetic energy landscape is then thoroughly explored by applying constraints to the occupation matrices of the d or f orbitals, enabling the identification of the global energy minimum. Through this process, we identify 194 magnetic monolayers, comprising 109 ferromagnetic, 83 antiferromagnetic, and 2 altermagnetic systems. To estimate critical temperatures and highlight promising candidates, we employ a proxy based on the energy difference between ferromagnetic and antiferromagnetic states. This method is applied to the 137 monolayers that exhibit an electronic band gap. Among the remaining 57 metallic systems, we identify 12 novel half-metals with strong potential for spintronics applications.

## Methods

In this work, all calculations are collinear and performed using the Quantum ESPRESSO (QE) distribution.^108–110^ Calculations are performed within the spin-polarized generalized-gradient approximation (GGA) for exchange-correlation functional using the Perdew–Burke–Ernzerhof (PBE) prescription. The pseudopotentials are taken from the standard solid-state pseudopotentials (SSSP Efficiency) library^111–115^ that includes different types of pseudopotentials such as norm-conserving (NC), ultrasoft (USPP), and projector-augmented-wave (PAW), together with the recommended wave function and charge-density kinetic-energy cutoffs, that are determined through systematic convergence tests.^111^ We use the most recent versions of the SSSP library available at the time for our calculations. Specifically, version 1.1.2 is used for the PBE calculations, and version 1.3.0 is utilized for the PBE + *U* calculations. The **K**-points density of 0.2 Å^–1^ and **q**-points mesh density of 0.4 Å^–1^ have been set for single self-consistent (scf) and density functional perturbation theory (DFPT) calculations, respectively. In this work, a system (an atom) is magnetic if the absolute total magnetization per cell (of the atom) is larger than 0.1 μ_*B*_. We employ the 2D Coulomb-interaction truncation implemented in QE,^116^ which effectively removes spurious electrostatic interactions between spurious periodic replicas in the out-of-plane direction. This approach requires the vertical cell size to scale with the geometric thickness of the 2D material and amount to at least 15 Å of vacuum spacing even for one-atom thick layers.

In this work we consider systems from MC2D,^13,14,60^ which is created from a collection of 3077 exfoliable monolayer that were identified from prototypes of the Inorganic Crystal Structure Database (ICSD),^117,118^ the Crystallography Open Database (COD),^119^ and Materials Platform for Data Science (MPDS)^120^ databases. We identify and characterize magnetic monolayers in MC2D in two main steps, as shown in Figure 1: in the first step (Chronos step), using PBE calculations, potentially magnetic monolayers are identified, and in the second step (RomeoDFT step), after including Hubbard corrections and exploring the energy landscape, the magnetic ground state of these materials is found. An initial screening was performed using an automated AiiDA^61,62^ workflow (Chronos) on 877 easily exfoliable materials (i.e., materials with a binding energy per unit of area lower or equal to 30 meV/Å^2^ when computed with the vdW-DF2-c09 functional or lower or equal to 35 meV/Å^2^ when computed with the rvv10) with up to 12 atoms per unit cell, using PBE calculations, and 228 magnetic monolayers are identified by the following procedure: the geometry of the system is optimized in ferromagnetic and nonmagnetic orderings. If the ferromagnetic ordering shows lower energy than the nonmagnetic case, the system is considered to be potentially magnetic, and other antiferromagnetic orderings (making sure the supercell size is now 2 × 1 to accommodate two magnetic atoms) and five random configurations are created. The system is optimized starting from different starting magnetization defined by spin density (*m* ^*I*^ = ∫(ρ^*I*,↑^ (**r**) – ρ^*I*,↓^ (**r**))*d* **r** where ρ describes the density and I is the atom index). As stated before this simplified approach is effective in capturing the correct magnetic ground state in the absence of Hubbard correction (see Supporting Information). However, it is important to note that there are still local minima within the energy landscape that are accessible through the manipulations of the orbital occupations.

This can be achieved using RomeoDFT, which corresponds to the second step of our overall workflow. In this approach, the system is induced toward a set of target occupation matrices *ñ* ^*I*^ using Lagrange multipliers

$$Ẽ=E_{\mathrm{DFT}}+E_{U}+\sum_{I,m_{1},m_{2}}\lambda_{m_{1},m_{2}}^{I}\left(n_{m_{1},m_{2}}^{I}-{ñ}_{m_{1},m_{2}}^{I}\right)$$

with

2 $$E_{U}=\frac{1}{2}\sum_{I}\sum_{\sigma m_{1}m_{2}}U^{I}\left(\delta_{m_{1}m_{2}}-n^{I\sigma }n_{m_{1}m_{2}}^{I\sigma }\right)n_{m_{2}m_{1}}^{I\sigma }$$

where E_*U*_ is the Hubbard corrected energy, λ is the Lagrange multiplier, representing the strength of the energy penalty associated with a deviation from the target occupation matrices, *I* is the atomic site index, *m*_1_ and *m*_2_ are the magnetic quantum numbers associated with a specific angular momentum and *U* ^*I*^ is the on-site Hubbard parameters. *n*_*m*_1_ *m*_2__^*Iσ*^ are the generalized atomic occupation matrices which are computed by projecting the Kohn–Sham (KS) wave functions ψ_*v* **k**σ_(**r**) on atomic orbitals φ_*m*_1__^*I*^(**r**) as *n*_*m*_1_ *m*_2__^*Iσ*^ = ∑_*v* **k**_ *f*_*v* **k**σ_ ⟨ψ_*v* **k**σ_|φ_*m*_2__^*J*^⟩ ⟨φ_*m*_1__^*I*^|ψ_*v* **k**σ_⟩, where *f*_*v* **k**σ_ are the occupations of KS states. The general formulation of DFT + *U* is discussed in refs 31,34,36,38 . We note that in this case the magnetization is defined from the occupation matrix of spin up and down: *m* ^*I*^ = ∑_*m*_ (*n*_*mm*_^*I*↑^ – *n*_*mm*_^*I*↓^). After imposing the constraint for a fixed number of self-consistent cycles, the constraint is released and the system is allowed to evolve freely to the closest self-consistent energy minimum. More information about RomeoDFT is provided in ref 49. This implementation is made in Quantum ESPRESSO and the process is automatized in *Julia* and can be found on GitHub.^121^ We use RomeoDFT workflow to determine the ground state at the PBE + *U* level for 194 magnetic monolayers, identified by Chronos with a filter of up to 12 atoms per primitive cell. To keep the computational cost manageable, we explore possible antiferromagnetic configurations by accommodating for at least two magnetic ions per simulation cell, which means allowing at most for a 2 × 1 supercell up to 24 atoms. This means some nontrivial AFM configurations are beyond the present scope. In this procedure, an initial unconstrained self-consistent calculation is performed to obtain the reference atomic occupation matrices. Then, 10 random trial target occupations are generated either with the same number of electrons or ± 1 electrons to account for possible oxidation states. These trial occupations serve as starting points for new self-consistent calculations, leading to distinct local minima in the energy landscape. They can give rise to new unique states or converge to previous states. The new generation of trial target occupations are the mean value of the occupation matrices of the previous calculations. The iterative search continues until the ratio of newly discovered unique states to total calculated trials falls below a small predefined threshold. Due to computational limitations, the procedure may also be stopped manually after several hundred states have been explored. In such cases, there remains a small uncertainty as to whether the absolute ground state has been reached. In principle, for each solution with an atomic magnetic moment *m* ^*I*^, a corresponding solution with −*m* ^*I*^ should exist. However, it is possible that the process is stopped before the opposite-spin counterpart is explored. While the exploration of noncollinear configurations of the density matrix is currently not accessible within RomeoDFT, identifying prospective noncollinear magnetic states remains an interesting direction for future research that lies beyond the scope of the present work.

The required data to reproduce the RomeoDFT results for all the materials, along with instructions, are given in the Materials Cloud Archive.^122^ For three representative systems, we provided the full set of self-consistent states identified using RomeDFT.

In order to calculate the *U* parameter, we use DFPT^34,36^ as implemented in the HP code,^32,35,38^ which is part of QE. The Hubbard *U* parameter is computed in three iterative steps. Within each iteration the *U* value is updated as the average values of the initial and calculated values in the previous step (only in the special case of CoO_2_, because the ground state is highly sensitive to the choice of *U*, in order to avoid falling into the wrong state we do not update the *U* value as the average of the previous step, instead, in each step we use the same calculated value of the *U* from the previous step). Our results show that with this approach, the convergence threshold of *U* for 87% of the systems is less than 0.5 eV. If the convergence of 0.5 eV has not been reached in three steps, we do another final step. After the calculation of the *U*, the geometry and the Hubbard *U* are fixed, and we explore the energy landscape to find the global minimum. For the systems with fully occupied d orbitals, we use a zero value for the *U* because the effects of the Hubbard correction at integer occupations are negligible.^32^ In the entire process, the geometry is fixed to the ground state as identified by Chronos prior to optimization to avoid introducing geometric optimization only on one state. The appropriate approach involves relaxing the geometry across all states within the energy landscape, which can be computationally demanding.

To further characterize the magnetic properties of our materials, we mapped their electronic structure onto a model Heisenberg Hamiltonian

3 $$H=\sum_{\mathrm{ij}}J_{\mathrm{ij}}{Ŝ}_{i}\cdot {Ŝ}_{j}$$

where *J*_*ij*_ corresponds to the magnetic exchange coupling between the net spins in sites *i* and *j*. The magnitude of the spins is included in the *J*. The sum runs over every spin such that each coupling is counted twice, i.e., *J*_*ij*_ and *J*_*ji*_ are both included. Our convention is such that a negative *J* indicates a ferromagnetic coupling, and a positive *J* is indicative of antiferromagnetic interaction. The exchange parameters reported here refer to the isotropic Heisenberg model, and the SOC-induced anisotropic or chiral exchange terms that are responsible for frustration and topological spin textures are not included in this study.^89^ The transition temperature is calculated from mean-field theory through the formula

4 $$T_{c}=\frac{2\mathrm{J̃}}{3k_{B}}$$

where *k*_*B*_ is the Boltzmann constant and *J̃* = ∑_*j*_ *J*_*ij*_. We calculated the exchange parameters using two approaches. First, we employed the |*E*_*FM*_ – *E*_*AFM*_| method, which maps this total energy difference onto a nearest-neighbor-only model Hamiltonian and the results are shown in Figure 6. Here, the exchange coupling *J* is assumed to be present between *n* equivalent nearest neighbors (*J̃* = *nJ*) and given by $J=\frac{\Delta E}{2\mathrm{nN}}$ , where Δ*E* is the energy difference between self-consistent ferromagnetic and antiferromagnetic spin configurations and *N* the number of magnetic atoms in the unit cell. To account for interactions beyond the first nearest neighbors, we employ the four-state method. In this approach, a large supercell is considered (4 × 4 × 1), and each *J*_*ij*_ exchange interaction is calculated by computing the total energy when the pair of spins in sites *i* and *j* are in four distinct collinear spin configurations while keeping all other spins ferromagnetically ordered. The four configurations are up–up, down–up, up–down, and down–down concerning the quantization axis. The exchange parameter is then calculated by^94^

5 $$J_{\mathrm{ij}}=\frac{1}{8}\left(E_{↑↑}+E_{↓↓}-E_{↑↓}-E_{↓↑}\right)$$

The calculated exchange parameters for CrI_3_ are *J*_1_ = −9.2 meV, *J*_2_ = −1.6 meV and *J*_3_ = −0.6 meV; for GdI_2_ they are *J*_1_ = −9.6 meV, *J*_2_ = −0.2 meV and *J*_3_ = −0.005 meV; for VPS_3_ they are *J*_1_ = 37.2 meV, *J*_2_ = 34.1 meV, *J*_3_ = 0.5 meV and *J*_4_ = 0.5 meV; and for MoCl_3_ are *J*_1_ = 126.7 meV, *J*_2_ = −4.0 meV, *J*_3_ = −0.4 meV and *J*_4_ = 0.1 meV.

Finally, to go beyond the mean-field theory and obtain a more precise estimate of the paramagnetic critical temperatures, we use the Monte Carlo Metropolis algorithm as implemented in the Vampire package^123^ for a few selected materials. Here, we use periodic boundaries in 2D, an equilibration time of 5.000 Monte Carlo steps, and statistical averaging also over 5.000 steps. We use the “curie-temperature” program varying the temperature from 0 to 500 K with a step of 10 K. We used a simulation box corresponding a 8 × 8 supercell. The critical temperatures *T*_*C*_ are then obtained by fitting the Monte Carlo magnetization curves to the mean-field formula M = *M*_0_ (1 – *T*/*T*_*C*_)^β^, where *M*_0_ and the critical exponent β are also fitting parameters. To verify that the number of thermalization steps was sufficient, we performed a convergence test for MoCl_3_ in which the number of steps was doubled. The resulting transition temperature changed by only ∼1 K, confirming that our chosen thermalization length is adequate.

It is important to note the approximations involved in our results. Besides assuming a magnetism driven by localized magnetic moments, the mapping of the |*E*_*FM*_ – *E*_*AFM*_| onto the Heisenberg model considers only nearest neighbors coupling. This can be regarded as a mean-field approximation. For instance, in MoCl_3_, the Mo atoms transition from a honeycomb lattice, where each site has three equidistant nearest neighbors, to a triangular lattice of dimers. In this configuration, the MoMo distance within a dimer is 1.06 Å shorter than the distance between dimers (see Supporting Information).

Anisotropy is also neglected in our calculations, and all calculations are done within the nonrelativistic limit. While anisotropy could play a significant role in 2D materials, we note that the role of magnetocrystalline anisotropy (MAE) has been assessed using Monte Carlo simulations on a model honeycomb lattice, showing that moderate MAE values (*D*/*J* < 0.1 with *D* being the anisotropy strength) alter *T*_*c*_ by less than 10%. This is consistent with recent DFT-based estimates of MAE (approximately 0.011 meV per atom) in comparable 2D systems.^24,124^ Our result aligns with a recent research that shows that even for isotropic 2D systems in lab setups short-range interactions can be large enough to stabilize the magnetic order at a finite temperature due to finite-size effects.^95^ Therefore, although anisotropy plays a role in a quantitative estimation of the critical temperature, its magnitude in the present materials is unlikely to change the qualitative classification of magnetic ground states.

## Supplementary Material



## Notes

Details of the Chronos workflow; comparison between RomeoDFT and Chronos at PBE level; calculation of Hubbard *U*; dimerization in MoCl_3_; altermagnetic systems; structural, electronic, and magnetic information on all materials (PDF)

## ◆

CERN (European Organization for Nuclear Research), CH-1211 Geneva, Switzerland

## References

[1] Burch K. S., Mandrus D., Park J.-G. (2018). Magnetism in Two-Dimensional van
Der Waals Materials. Nature.

[2] Wang Q. H., Bedoya-Pinto A., Blei M., Dismukes A. H., Hamo A., Jenkins S., Koperski M., Liu Y., Sun Q.-C., Telford E. J. (2022). The Magnetic Genome of Two-Dimensional van
Der Waals Materials. ACS Nano.

[3] Rhone T. D., Bhattarai R., Gavras H., Lusch B., Salim M., Mattheakis M., Larson D. T., Krockenberger Y., Kaxiras E. (2023). Artificial Intelligence
Guided Studies of van Der Waals
Magnets. Adv. Theory Simul..

[4] Gibertini M., Koperski M., Morpurgo A. F., Novoselov K. S. (2019). Magnetic
2D Materials and Heterostructures. Nat. Nanotechnol..

[5] Sødequist J., Olsen T. (2023). Type II Multiferroic
Order in Two-Dimensional Transition Metal Halides
from First Principles Spin-Spiral Calculations. 2D Mater..

[6] Ovesen M., Olsen T. (2024). Orbital Magnetization in Two-Dimensional
Materials from High-Throughput
Computational Screening. 2D Mater..

[7] Xin C., Yin Y., Song B., Fan Z., Song Y., Pan F. (2023). Machine Learning-Accelerated
Discovery of Novel 2D Ferromagnetic Materials with Strong Magnetization. Chip.

[8] Shen Z.-X., Su C., He L. (2022). High-Throughput Computation and Structure Prototype
Analysis for Two-Dimensional Ferromagnetic Materials. npj Comput. Mater..

[9] Xia W., Sakurai M., Balasubramanian B., Liao T., Wang R., Zhang C., Sun H., Ho K.-M., Chelikowsky J. R., Sellmyer D. J., Wang C. Z. (2022). Accelerating
the Discovery of Novel
Magnetic Materials Using Machine Learning–Guided Adaptive Feedback. Proc. Natl. Acad. Sci. U.S.A..

[10] Huang B., Clark G., Navarro-Moratalla E., Klein D. R., Cheng R., Seyler K. L., Zhong D., Schmidgall E., McGuire M. A., Cobden D. H. (2017). Layer-Dependent Ferromagnetism
in a van Der Waals Crystal down to the Monolayer Limit. Nature.

[11] Bedoya-Pinto A., Ji J.-R., Pandeya A. K., Gargiani P., Valvidares M., Sessi P., Taylor J. M., Radu F., Chang K., Parkin S. S. P. (2021). Intrinsic 2D-XY Ferromagnetism in a van Der Waals Monolayer. Science.

[12] Posey V. A., Turkel S., Rezaee M., Devarakonda A., Kundu A. K., Ong C. S., Thinel M., Chica D. G., Vitalone R. A., Jing R. (2024). Two-Dimensional Heavy
Fermions in the van Der Waals Metal CeSiI. Nature.

[13] Mounet N., Gibertini M., Schwaller P., Campi D., Merkys A., Marrazzo A., Sohier T., Castelli I. E., Cepellotti A., Pizzi G., Marzari N. (2018). Two-Dimensional Materials from High-Throughput
Computational Exfoliation of Experimentally Known Compounds. Nat. Nanotechnol..

[14] Campi D., Mounet N., Gibertini M., Pizzi G., Marzari N. (2023). Expansion
of the Materials Cloud 2D Database. ACS Nano.

[15] Lebègue S., Björkman T., Klintenberg M., Nieminen R. M., Eriksson O. (2013). Two-Dimensional
Materials from Data Filtering and*Ab Initio* Calculations. Phys. Rev. X.

[16] Rasmussen F. A., Thygesen K. S. (2015). Computational 2D Materials Database:
Electronic Structure
of Transition-Metal Dichalcogenides and Oxides. J. Phys. Chem. C.

[17] Choudhary K., Kalish I., Beams R., Tavazza F. (2017). High-Throughput
Identification
and Characterization of Two-dimensional Materials Using Density Functional
Theory. Sci. Rep..

[18] Ashton M., Paul J., Sinnott S. B., Hennig R. G. (2017). Topology-Scaling
Identification of Layered Solids and Stable Exfoliated 2D Materials. Phys. Rev. Lett..

[19] Cheon G., Duerloo K.-A. N., Sendek A. D., Porter C., Chen Y., Reed E. J. (2017). Data Mining for
New Two- and One-Dimensional Weakly
Bonded Solids and Lattice-Commensurate Heterostructures. Nano Lett..

[20] Zhou J., Shen L., Costa M. D., Persson K. A., Ong S. P., Huck P., Lu Y., Ma X., Chen Y., Tang H., Feng Y. P. (2019). 2DMatPedia, an Open
Computational
Database of Two-Dimensional Materials from Top-down and Bottom-up
Approaches. Sci. Data.

[21] Haastrup S., Strange M., Pandey M., Deilmann T., Schmidt P. S., Hinsche N. F., Gjerding M. N., Torelli D., Larsen P. M., Riis-Jensen A. C. (2018). The Computational 2D
Materials Database: High-Throughput
Modeling and Discovery of Atomically Thin Crystals. 2D Mater..

[22] Gjerding M. N., Taghizadeh A., Rasmussen A., Ali S., Bertoldo F., Deilmann T., Knøsgaard N. R., Kruse M., Larsen A. H., Manti S. (2021). Recent
Progress of the Computational 2D Materials Database
(C2DB). 2D Mater..

[23] Xu S., Jia F., Dai N. (2025). High throughput
discovery of 2D ferromagnetic and multiferroic
transition metal oxyhalides and nitrogen halides. npj Comput. Mater..

[24] Torelli D., Thygesen K. S., Olsen T. (2019). High Throughput Computational Screening
for 2D Ferromagnetic Materials: The Critical Role of Anisotropy and
Local Correlations. 2D Mater..

[25] Kabiraj A., Kumar M., Mahapatra S. (2020). High-Throughput
Discovery of High
Curie Point Two-Dimensional Ferromagnetic Materials. npj Comput. Mater..

[26] Torelli D., Olsen T. (2020). First Principles Heisenberg Models
of 2D Magnetic Materials: The
Importance of Quantum Corrections to the Exchange Coupling. J. Phys.: Condens. Matter.

[27] Torelli D., Olsen T. (2019). Calculating Critical
Temperatures for Ferromagnetic Order in Two-Dimensional
Materials. 2D Mater..

[28] Kulik H. J., Cococcioni M., Scherlis D. A., Marzari N. (2006). Density Functional
Theory in Transition-Metal Chemistry: A Self-Consistent Hubbard U
Approach. Phys. Rev. Lett..

[29] Anisimov V. I., Zaanen J., Andersen O. K. (1991). Band Theory
and Mott Insulators:
Hubbard *U* Instead of Stoner *I*. Phys. Rev. B.

[30] Anisimov V. I., Aryasetiawan F., Lichtenstein A. I. (1997). First-Principles Calculations of
the Electronic Structure and Spectra of Strongly Correlated Systems:
The LDA + *U* Method. J. Phys.:
Condens. Matter.

[31] Dudarev S. L., Botton G. A., Savrasov S. Y., Humphreys C. J., Sutton A. P. (1998). Electron-Energy-Loss Spectra and the Structural Stability
of Nickel Oxide: An LSDA+U Study. Phys. Rev.
B.

[32] Cococcioni M., de Gironcoli S. (2005). Linear Response
Approach to the Calculation of the
Effective Interaction Parameters in the LDA+U Method. Phys. Rev. B.

[33] Moore G. C., Horton M. K., Ganose A. M., Siron M., Linscott E., O’Regan D. D., Persson K. A. (2024). High-Throughput
Determination of
Hubbard U and Hund J Values for Transition Metal Oxides via Linear
Response Formalism. Phys. Rev. Mater..

[34] Timrov I., Marzari N., Cococcioni M. (2021). Self-Consistent Hubbard Parameters
from Density-Functional Perturbation Theory in the Ultrasoft and Projector-Augmented
Wave Formulations. Phys. Rev. B.

[35] Timrov I., Marzari N., Cococcioni M. (2022). HP –
A Code for the Calculation
of Hubbard Parameters Using Density-Functional Perturbation Theory. Comput. Phys. Commun..

[36] Timrov I., Marzari N., Cococcioni M. (2018). Hubbard Parameters
from Density-Functional
Perturbation Theory. Phys. Rev. B.

[37] Bastonero L., Malica C., Macke E., Bercx M., Huber S., Timrov I., Marzari N. (2025). First-principles
Hubbard parameters
with automated and reproducible workflows. npj
Comput. Mater..

[38] Campo V. L., Cococcioni M. (2010). Extended DFT
+ *U* + *V* Method with On-Site and
Inter-Site Electronic
Interactions. J. Phys.: Condens. Matter.

[39] Haddadi F., Linscott E., Timrov I., Marzari N., Gibertini M. (2024). On-Site and
Intersite Hubbard Corrections in Magnetic Monolayers: The Case of
FePS 3 and CrI 3. Phys. Rev. Mater..

[40] Meredig B., Thompson A., Hansen H. A., Wolverton C., van de Walle A. (2010). Method for Locating Low-Energy Solutions
within DFT
+ U. Phys. Rev. B.

[41] Zhou F., Ozoliņš V. (2009). Obtaining
Correct Orbital Ground
States in f -Electron Systems Using a Nonspherical Self-Interaction-Corrected
LDA + U Method. Phys. Rev. B.

[42] Ylvisaker E. R., Pickett W. E., Koepernik K. (2009). Anisotropy
and Magnetism in the LSDA
+ U Method. Phys. Rev. B.

[43] Jollet F., Jomard G., Amadon B., Crocombette J. P., Torumba D. (2009). Hybrid Functional for Correlated
Electrons in the Projector
Augmented-Wave Formalism: Study of Multiple Minima for Actinide Oxides. Phys. Rev. B.

[44] Jomard G., Amadon B., Bottin F., Torrent M. (2008). Structural,
Thermodynamic,
and Electronic Properties of Plutonium Oxides from First Principles. Phys. Rev. B.

[45] Amadon B., Jollet F., Torrent M. (2008). *γ* vand *β* Cerium: LDA + U Calculations of Ground-State
Parameters. Phys. Rev. B.

[46] Zhang W., Koepernik K., Richter M., Eschrig H. (2009). Magnetic Phase Transition
in CoO under High Pressure: A Challenge for LSDA + U. Phys. Rev. B.

[47] Kasinathan D., Koepernik K., Nitzsche U., Rosner H. (2007). Ferromagnetism
Induced
by Orbital Order in the Charge-Transfer Insulator Cs 2 AgF 4: An Electronic
Structure Study. Phys. Rev. Lett..

[48] Shick A. B., Janiš V., Drchal V., Pickett W. E. (2004). Spin and Orbital
Magnetic State of U Ge 2 under Pressure. Phys.
Rev. B.

[49] Ponet L., Di Lucente E., Marzari N. (2024). The Energy Landscape of Magnetic
Materials. npj Comput. Mater..

[50] Horton M. K., Montoya J. H., Liu M., Persson K. A. (2019). High-Throughput
Prediction of the Ground-State Collinear Magnetic Order of Inorganic
Materials Using Density Functional Theory. npj
Comput. Mater..

[51] Payne A., Avedaño-Franco G., He X., Bousquet E., Romero A. H. (2019). Optimizing the Orbital Occupation
in the Multiple Minima
Problem of Magnetic Materials from the Metaheuristic Firefly Algorithm. Phys. Chem. Chem. Phys..

[52] Allen J. P., Watson G. W. (2014). Occupation Matrix Control of d- and f-Electron Localisations
Using DFT+U. Phys. Chem. Chem. Phys..

[53] Dorado B., Jomard G., Freyss M., Bertolus M. (2010). Stability of Oxygen
Point Defects in UO 2 by First-Principles DFT + U Calculations: Occupation
Matrix Control and Jahn-Teller Distortion. Phys.
Rev. B.

[54] Dorado B., Amadon B., Freyss M., Bertolus M. (2009). DFT + U Calculations
of the Ground State and Metastable States of Uranium Dioxide. Phys. Rev. B.

[55] Tellez-Mora A., He X., Bousquet E., Wirtz L., Romero A. H. (2024). Systematic Determination
of a Material’s Magnetic Ground State from First Principles. npj Comput. Mater..

[56] Sødequist J., Olsen T. (2024). Magnetic Order in the Computational
2D Materials Database (C2DB)
from High Throughput Spin Spiral Calculations. npj Comput. Mater..

[57] Huebsch M.-T., Nomoto T., Suzuki M.-T., Arita R. (2021). Benchmark for *Ab Initio* Prediction of Magnetic Structures
Based on Cluster-Multipole
Theory. Phys. Rev. X.

[58] Zheng F., Zhang P. (2021). MagGene: A Genetic
Evolution Program for Magnetic Structure Prediction. Comput. Phys. Commun..

[59] Baumsteiger J., Celiberti L., Rinke P., Todorović M., Franchini C. (2025). Exploring noncollinear magnetic energy landscapes with
Bayesian optimization. Digital Discovery.

[60] The Materials Cloud Two-Dimensional Structure Database (MC2D). https://mc2d.materialscloud.org/.

[61] Pizzi G., Cepellotti A., Sabatini R., Marzari N., Kozinsky B. (2016). AiiDA: Automated
Interactive Infrastructure and Database for Computational Science. Comput. Mater. Sci..

[62] Huber S. P., Zoupanos S., Uhrin M., Talirz L., Kahle L., Häuselmann R., Gresch D., Müller T., Yakutovich A. V., Andersen C. W. (2020). AiiDA 1.0, a Scalable
Computational Infrastructure for Automated Reproducible Workflows
and Data Provenance. Sci. Data.

[63] Perdew J. P., Burke K., Ernzerhof M. (1996). Generalized
Gradient Approximation
Made Simple. Phys. Rev. Lett..

[64] Cohen A. J., Mori-Sánchez P., Yang W. (2008). Insights into Current Limitations
of Density Functional Theory. Science.

[65] Bao J. L., Gagliardi L., Truhlar D. G. (2018). Self-Interaction Error in Density
Functional Theory: An Appraisal. J. Phys. Chem.
Lett..

[66] Himmetoglu B., Floris A., De Gironcoli S., Cococcioni M. (2014). Hubbard-Corrected
DFT Energy Functionals: The LDA+U Description of Correlated Systems. Int. J. Quantum Chem..

[67] Mosquera M. A., Wasserman A. (2014). Derivative Discontinuities in Density Functional Theory. Mol. Phys..

[68] Anisimov V. I., Solovyev I. V., Korotin M. A., Czyżyk M. T., Sawatzky G. A. (1993). Density-Functional Theory and NiO Photoemission Spectra. Phys. Rev. B.

[69] Solovyev I. V., Dederichs P. H., Anisimov V. I. (1994). Corrected Atomic
Limit in the Local-Density
Approximation and the Electronic Structure of *d* Impurities
in Rb. Phys. Rev. B.

[70] Liechtenstein A. I., Anisimov V. I., Zaanen J. (1995). Density-Functional
Theory and Strong
Interactions: Orbital Ordering in Mott-Hubbard Insulators. Phys. Rev. B.

[71] Perdew J. P., Parr R. G., Levy M., Balduz J. L. (1982). Density-Functional
Theory for Fractional Particle Number: Derivative Discontinuities
of the Energy. Phys. Rev. Lett..

[72] Liang L., Du S., Wang L., Liu Z., Wu J., Zhang S. (2021). Tunable Magnetic
and Electronic Properties of the 2D CoO_2_ Layer. J. Phys. Chem. C.

[73] Smolyanyuk A., Šmejkal L., Mazin I. I. (2024). A Tool to Check
Whether a Symmetry-Compensated
Collinear Magnetic Material Is Antiferro- or Altermagnetic. SciPost Phys. Codebases.

[74] Sødequist J., Olsen T. (2024). Two-Dimensional Altermagnets
from High Throughput Computational Screening:
Symmetry Requirements, Chiral Magnons, and Spin-Orbit Effects. Appl. Phys. Lett..

[75] Zeng S., Zhao Y.-J. (2024). Description of two-dimensional
altermagnetism: Categorization
using spin group theory. Phys. Rev. B.

[76] Chen W., Sun Z., Wang Z., Gu L., Xu X., Wu S., Gao C. (2019). Direct Observation
of van Der Waals Stacking-Dependent Interlayer
Magnetism. Science.

[77] Lee K., Dismukes A. H., Telford E. J., Wiscons R. A., Wang J., Xu X., Nuckolls C., Dean C. R., Roy X., Zhu X. (2021). Magnetic Order
and Symmetry in the 2D Semiconductor CrSBr. Nano Lett..

[78] Son J., Son S., Park P., Kim M., Tao Z., Oh J., Lee T., Lee S., Kim J., Zhang K. (2021). Air-Stable
and Layer-Dependent Ferromagnetism in Atomically Thin van Der Waals
CrPS_4_. ACS Nano.

[79] Kim K., Lim S. Y., Lee J.-U., Lee S., Kim T. Y., Park K., Jeon G. S., Park C.-H., Park J.-G., Cheong H. (2019). Suppression of Magnetic Ordering
in XXZ-type Antiferromagnetic
Monolayer NiPS_3_. Nat. Commun..

[80] Lee J.-U., Lee S., Ryoo J. H., Kang S., Kim T. Y., Kim P., Park C.-H., Park J.-G., Cheong H. (2016). Ising-Type Magnetic
Ordering in Atomically Thin FePS_3_. Nano Lett..

[81] Olsen T. (2024). Antiferromagnetism
in Two-Dimensional Materials: Progress and Computational Challenges. 2D Mater..

[82] Chittari B. L., Park Y., Lee D., Han M., MacDonald A. H., Hwang E., Jung J. (2016). Electronic and magnetic
properties
of single-layer *MPX*
_3_ metal phosphorous
trichalcogenides. Phys. Rev. B.

[83] Sivadas N., Daniels M. W., Swendsen R. H., Okamoto S., Xiao D. (2015). Magnetic ground
state of semiconducting transition-metal trichalcogenide monolayers. Phys. Rev. B.

[84] Olsen T. (2021). Magnetic Anisotropy
and Exchange Interactions of Two-Dimensional FePS_3_, NiPS_3_ and MnPS_3_ from First Principles Calculations. J. Phys. D: Appl. Phys..

[85] Xing W., Chen Y., Odenthal P. M. (2017). 2D
Mater. 4 024009. 2D Mater..

[86] Gong C., Li L., Li Z., Ji H., Stern A., Xia Y., Cao T., Bao W., Wang C., Wang Y. (2017). Discovery
of Intrinsic Ferromagnetism in Two-Dimensional van Der Waals Crystals. Nature.

[87] Xu C., Feng J., Xiang H., Bellaiche L. (2018). Interplay
between Kitaev interaction and single ion anisotropy in ferromagnetic
CrI3 and CrGeTe3 monolayers. npj Comput. Mater..

[88] Menichetti G., Calandra M., Polini M. (2019). Electronic structure
and magnetic
properties of few-layer Cr2Ge2Te6: the key role of nonlocal electron–electron
interaction effects. 2D Mater..

[89] Amoroso D., Barone P., Picozzi S. (2020). Spontaneous
skyrmionic lattice from
anisotropic symmetric exchange in a Ni-halide monolayer. Nat. Commun..

[90] Kim M., Kumaravadivel P., Birkbeck J., Kuang W., Xu S. G., Hopkinson D. G., Knolle J., McClarty P. A., Berdyugin A. I., Ben Shalom M. (2019). Micromagnetometry of Two-Dimensional Ferromagnets. Nat. Electron..

[91] Kim H. H., Yang B., Li S., Jiang S., Jin C., Tao Z., Nichols G., Sfigakis F., Zhong S., Li C. (2019). Evolution of Interlayer and Intralayer Magnetism in
Three Atomically
Thin Chromium Trihalides. Proc. Natl. Acad.
Sci. U.S.A..

[92] Zhang Z., Shang J., Jiang C., Rasmita A., Gao W., Yu T. (2019). Direct Photoluminescence Probing of Ferromagnetism in Monolayer Two-Dimensional
CrBr_3_. Nano Lett..

[93] Torelli D., Moustafa H., Jacobsen K. W., Olsen T. (2020). High-Throughput Computational
Screening for Two-Dimensional Magnetic Materials Based on Experimental
Databases of Three-Dimensional Compounds. npj
Comput. Mater..

[94] Xiang H. J., Kan E. J., Wei S.-H., Whangbo M.-H., Gong X. G. (2011). Predicting
the Spin-Lattice Order of Frustrated Systems from First Principles. Phys. Rev. B.

[95] Jenkins S., Rózsa L., Atxitia U., Evans R. F. L., Novoselov K. S., Santos E. J. G. (2022). Breaking through the Mermin-Wagner
Limit in 2D van
Der Waals Magnets. Nat. Commun..

[96] Garanin D. A. (1996). Self-Consistent
Gaussian Approximation for Classical Spin Systems: Thermodynamics. Phys. Rev. B.

[97] Pizzochero M., Yadav R., Yazyev O. V. (2020). Magnetic Exchange
Interactions in
Monolayer CrI _3_ from Many-Body Wavefunction Calculations. 2D Mater..

[98] Li X., Yang J. (2016). First-Principles Design of Spintronics Materials. Natl. Sci. Rev..

[99] De
Groot R. A., Mueller F. M., Engen P. G. V., Buschow K. H. J. (1983). New
Class of Materials: Half-Metallic Ferromagnets. Phys. Rev. Lett..

[100] Yao Q., Li J., Liu Q. (2021). Fragile Symmetry-Protected
Half Metallicity
in Two-Dimensional van Der Waals Magnets: A Case Study of Monolayer
Fe Cl 2. Phys. Rev. B.

[101] Ashton M., Gluhovic D., Sinnott S. B., Guo J., Stewart D. A., Hennig R. G. (2017). Two-Dimensional Intrinsic Half-Metals
With Large Spin Gaps. Nano Lett..

[102] Torun E., Sahin H., Singh S. K., Peeters F. M. (2015). Stable
Half-Metallic Monolayers of FeCl2. Appl. Phys.
Lett..

[103] Hadjadj S. E., González-Orellana C., Lawrence J., Bikaljević D., Peña-Díaz M., Gargiani P., Aballe L., Naumann J., Niño M. Á., Foerster M. (2023). Epitaxial Monolayers of the Magnetic 2D
Semiconductor FeBr _2_ Grown on Au(111). Chem. Mater..

[104] Prayitno T. B. (2021). Controlling
Phase Transition in Monolayer Metal Diiodides
XI _2_ (X: Fe, Co, and Ni) by Carrier Doping. J. Phys.: Condens. Matter.

[105] Zhou X., Jiang T., Tao Y., Ji Y., Wang J., Lai T., Zhong D. (2024). Evidence of Ferromagnetism
and Ultrafast Dynamics of Demagnetization in an Epitaxial FeCl _2_ Monolayer. ACS Nano.

[106] Kong X., Li L., Liang L., Peeters F. M., Liu X.-J. (2020). The Magnetic, Electronic, and Light-Induced
Topological
Properties in Two-Dimensional Hexagonal FeX2 (X = Cl, Br, I) Monolayers. Appl. Phys. Lett..

[107] Cai S., Yang F., Gao C. (2020). FeCl_2_ Monolayer on HOPG:
Art of Growth and Momentum Filtering Effect. Nanoscale.

[108] Giannozzi P., Baroni S., Bonini N., Calandra M., Car R., Cavazzoni C., Ceresoli D., Chiarotti G. L., Cococcioni M., Dabo I. (2009). QUANTUM ESPRESSO: A
Modular and Open-Source Software Project for Quantum Simulations of
Materials. J. Phys.: Condens. Matter.

[109] Giannozzi P., Andreussi O., Brumme T., Bunau O., Buongiorno Nardelli M., Calandra M., Car R., Cavazzoni C., Ceresoli D., Cococcioni M. (2017). Advanced Capabilities
for Materials Modelling with Quantum ESPRESSO. J. Phys.: Condens. Matter.

[110] Giannozzi P., Baseggio O., Bonfà P., Brunato D., Car R., Carnimeo I., Cavazzoni C., de Gironcoli S., Delugas P., Ferrari Ruffino F. (2020). Q Uantum ESPRESSO toward the Exascale. J. Chem.
Phys..

[111] Prandini G., Marrazzo A., Castelli I. E., Mounet N., Marzari N. (2018). Precision and Efficiency in Solid-State Pseudopotential
Calculations. npj Computat. Mater..

[112] Lejaeghere K., Bihlmayer G., Björkman T., Blaha P., Blügel S., Blum V., Caliste D., Castelli I. E., Clark S. J., Dal Corso A. (2016). Reproducibility in Density Functional Theory Calculations of Solids. Science.

[113] Vanderbilt D. (1990). Soft Self-Consistent Pseudopotentials
in a Generalized
Eigenvalue Formalism. Phys. Rev. B.

[114] Dal Corso A. (2014). Pseudopotentials Periodic Table:
From H to Pu. Comput. Mater. Sci..

[115] Garrity K. F., Bennett J. W., Rabe K. M., Vanderbilt D. (2014). Pseudopotentials
for High-Throughput DFT Calculations. Comput.
Mater. Sci..

[116] Sohier T., Calandra M., Mauri F. (2017). Density functional
perturbation theory for gated two-dimensional heterostructures: Theoretical
developments and application to flexural phonons in graphene. Phys. Rev. B.

[117] FIZ-Karlsruhe, Inorganic Crystal Structure Database (ICSD) 2019, http://www.fiz-karlsruhe.com/icsd.html. database version: 2017.2, Access date: January 02, 2019.

[118] Bergerhoff G., Hundt R., Sievers R., Brown I. D. (1983). The Inorganic
Crystal Structure Data Base. J. Chem. Inf. Comput.
Sci..

[119] Gražulis S., Daškevič A., Merkys A., Chateigner D., Lutterotti L., Quirós M., Serebryanaya N. R., Moeck P., Downs R. T., Le Bail A. (2012). Crystallography
Open Database (COD): An Open-Access Collection of Crystal Structures
and Platform for World-Wide Collaboration. Nucleic
Acids Res..

[120] Blokhin, E. ; Villars, P. MPDS: Materials Platform for Data Science. https://mpds.io.

[121] Ponet, L. RomeoDFT. https://github.com/louisponet/RomeoDFT.jl.

[122] Haddadi, F. ; Campi, D. ; dos Santos, F. ; Mounet, N. ; Ponet, L. ; Marzari, N. ; Gibertini, M. Materials Cloud Archive: Exploring the magnetic landscape of easily-exfoliable two-dimensional materials. https://archive.materialscloud.org/records/4x37g-nre30. 10.1021/acsnano.5c16067 PMC13173658 42046324

[123] Evans R. F. L., Fan W. J., Chureemart P., Ostler T. A., Ellis M. O. A., Chantrell R. W. (2014). Atomistic
Spin Model Simulations of Magnetic Nanomaterials. J. Phys.: Condens. Matter.

[124] Orlando F., Droghetti A., Varrassi L., Cuono G., Franchini C., Barone P., Marrazzo A., Gibertini M., Stavrić S., Picozzi S. (2026). AMaRaNTA: automated first-principles
exchange parameters in 2D magnets. npj Comput.
Mater..

