# Supplementary material for: Exploring the Magnetic Landscape of Easily Exfoliable Two-Dimensional Materials
Source: ACS Nano. 2026 Apr 28;20(18):13528–41. doi: 10.1021/acsnano.5c16067 (PMC13173658; doi:10.1021/acsnano.5c16067)
Supplement: Supplementary file 1 [file nn5c16067_si_001.pdf]

# Supporting information: "Exploring the magnetic landscape of easily-exfoliable two-dimensional materials"

Fatemeh Haddadi,[1,2,*] Davide Campi,[3] Flaviano José dos Santos,[4,5,6] Nicolas Mounet,[1,2,7] Louis Ponet,[1,2] Nicola Marzari,[1,2] and Marco Gibertini[8,9]

[1] *Theory and Simulation of Materials (THEOS), École Polytechnique Fédérale de Lausanne (EPFL), CH-1015 Lausanne, Switzerland*
[2] *National Centre for Computational Design and Discovery of Novel Materials (MARVEL), École Polytechnique Fédérale de Lausanne (EPFL), CH-1015 Lausanne, Switzerland*
[3] *Department of Materials Science, University of Milano-Bicocca, Via R. Cozzi 55, Milano 20125, Italy*
[4] *Laboratory for Materials Simulations (LMS), Paul Scherrer Institut, Villigen PSI, Switzerland*
[5] *National Centre for Computational Design and Discovery of Novel Materials (MARVEL), 5232 Villigen PSI, Switzerland*
[6] *Centro Brasileiro de Pesquisas Físicas, Rua Doutor Xavier Sigaud 150, Rio de Janeiro, 22290-180, Brazil*
[7] *CERN (European Organization for Nuclear Research), Geneva, Switzerland*
[8] *Dipartimento di Scienze Fisiche, Informatiche e Matematiche, University of Modena and Reggio Emilia, I-41125 Modena, Italy*
[9] *Centro S3, CNR-Istituto Nanoscienze, I-41125 Modena, Italy*

## S1. Details of the Chronos workflow

The Chronos workflow determines the magnetic ground state at the PBE level through a two-step process. At first, it screens for ferromagnetic states, generating multiple initial configurations: some by setting an integer initial spin on notoriously magnetic elements, others by setting random initial spin states on each atomic species. After a geometry optimization with spin-polarized DFT, the final total energy is compared with the non-magnetic case and, if a non-trivial magnetic state is found with a lower energy, a more complete screening of the possible antiferromagnetic states is launched using a 2×1 supercell, avoiding symmetry-redundant configurations. Ferromagnetic and non-magnetic configurations are also recomputed on the same supercell, and all the final energies are compared to identify the one with the minimum energy per formula unit, which is taken as the system's ground state. Using Chronos, we screened a total of 877 easily exfoliable materials (i.e. materials with a binding energy per unit of area lower than or equal to 30 meV/Å$^2$) and up to 12 atoms per unit cell from 3077 easily and potentially systems identified in Refs. 1, 2, finding 228 monolayers with a magnetic ground state. These include 56 magnetic monolayers already identified in Ref. [2] that have been independently recomputed with the Chronos workflow. The use of different pseudopotentials, SSSP version 1.1.2 in this work and SSSP version 0.7 in [2] resulted in slight variations of the optimized structural parameters and magnetic moments. Moreover, the magnetic structures from Ref. [2] have been subjected to a standardization step of the monolayer cell, resulting in an in-plane rotation of the cell, which has been omitted in the present work.

## S2. Comparing RomeoDFT and Chronos at PBE level

For this set of calculations, we use the same pseudopotentials (SSSP v. 1.1.2) in RomeoDFT to be able to compare the energies with the results from Chronos. The comparison is shown in Figure S1 where the absolute total magnetization and band gap values of the ground state found by the two methods are reported. The top panel in Figure S1 identifies whether each system is antiferromagnetic ($\sum_I m_I = 0$) or ferromagnetic ($\sum_I m_I \neq 0$), while the bottom panel determines whether it is metallic ($\epsilon_g = 0$) or semiconductor ($\epsilon_g \neq 0$). The figure demonstrates that, in the absence of Hubbard corrections, in most of the cases the two methods give the same results, although numerical discrepancies between the two methodologies are evident in some cases (such as $NiCl_2$ and $VS_2$). The most significant difference occurs in the case of NdOBr, where Chronos shows that the system is metallic and ferromagnetic with total magnetic moments as small as 0.8 $\mu_B$, while RomeoDFT shows that the ground state is semiconducting with an overall magnetic moment of 6 $\mu_B$. Interestingly, the ground state found by RomeoDFT is about 2.6 eV/cell lower in energy than the ground state found by Chronos. Therefore, this system is a strong evidence that, under the same conditions and parameters, RomeoDFT is able to find the true ground state of the system. Nonetheless, in most of

* fatemeh.haddadi@epfl.ch

the cases, Chronos is able to find the correct ground state and, being computationally more efficient than RomeoDFT, we have therefore used it in the main text to identify potentially magnetic systems, which are then studied more extensively through RomeoDFT including Hubbard corrections.

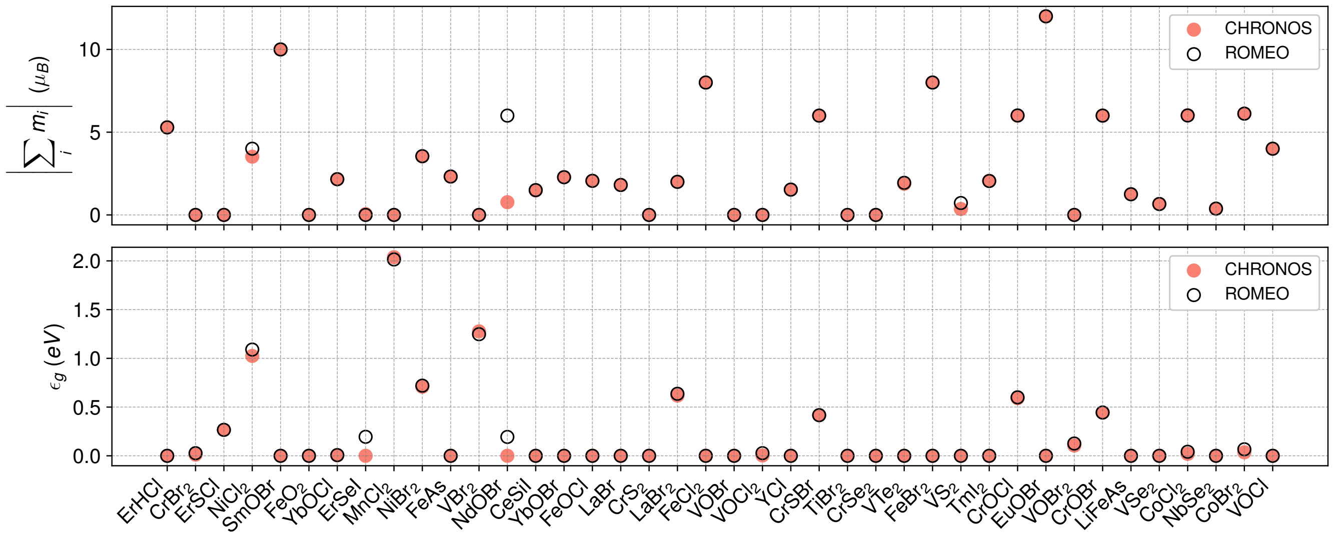


**Figure S1:** Total magnetization and band gap of the ground state for a subset of 40 magnetic monolayers identified from Chronos compared to RomeoDFT's results when no Hubbard correction is included. For consistency, only for these calculations, we used the same pseudopotential library used in Chronos (SSSP version 1.1.2) in RomeoDFT calculations. For the calculations considering the Hubbard $U$ the pseudopotentials are from SSSP latest library (version 1.3).

## S3. Calculation of the Hubbard $U$

The Hubbard $U$ parameters used in the main text are not obtained in a one-shot approach, but actually computed self-consistently. As detailed in the Methods, this means that the procedure to compute the Hubbard $U$ is repeated (at least three times), and the $U$ computed from density-functional perturbation theory (DFPT) starting from a ground-state density obtained with a Hubbard parameter that is the average of the input and output $U$ of the previous step. Figure S2 shows the converging behavior of a group of atoms in different materials with respect to the number of steps. In the first step an arbitrary non-zero value between 4-5 eV is used; for some cases like $Cu$ there was convergence issues in the first step that could be lifted by starting with a smaller $U$. The values of the Hubbard $U$ for the atoms in Figure S2 are shown in Figure S3. The error bars in Figure S3 illustrate the range between the input and output values from the last step, with the point itself representing their average. Figure S3 demonstrates that the same atomic species in different materials can exhibit a wide range of Hubbard $U$ values, for example for Iron it variesg from 3 to 8 eV, while for Manganese it has a narrower range of 2 to 4 eV.
In order to see how the $U$ value varies for each atom depending on the oxidation state, Figure S4 shows the calculated Hubbard $U$ values for different atomic species, averaged over multiple compounds, along with their oxidation states. We use the Hubbard $U$ to explore the energy landscape of magnetic monolayers using RomeoDFT package and calculate their effective exchange parameter, $\tilde{J}$. Table S1 shows the $\tilde{J}$ values for the systems in the Figure 6 of the main text.

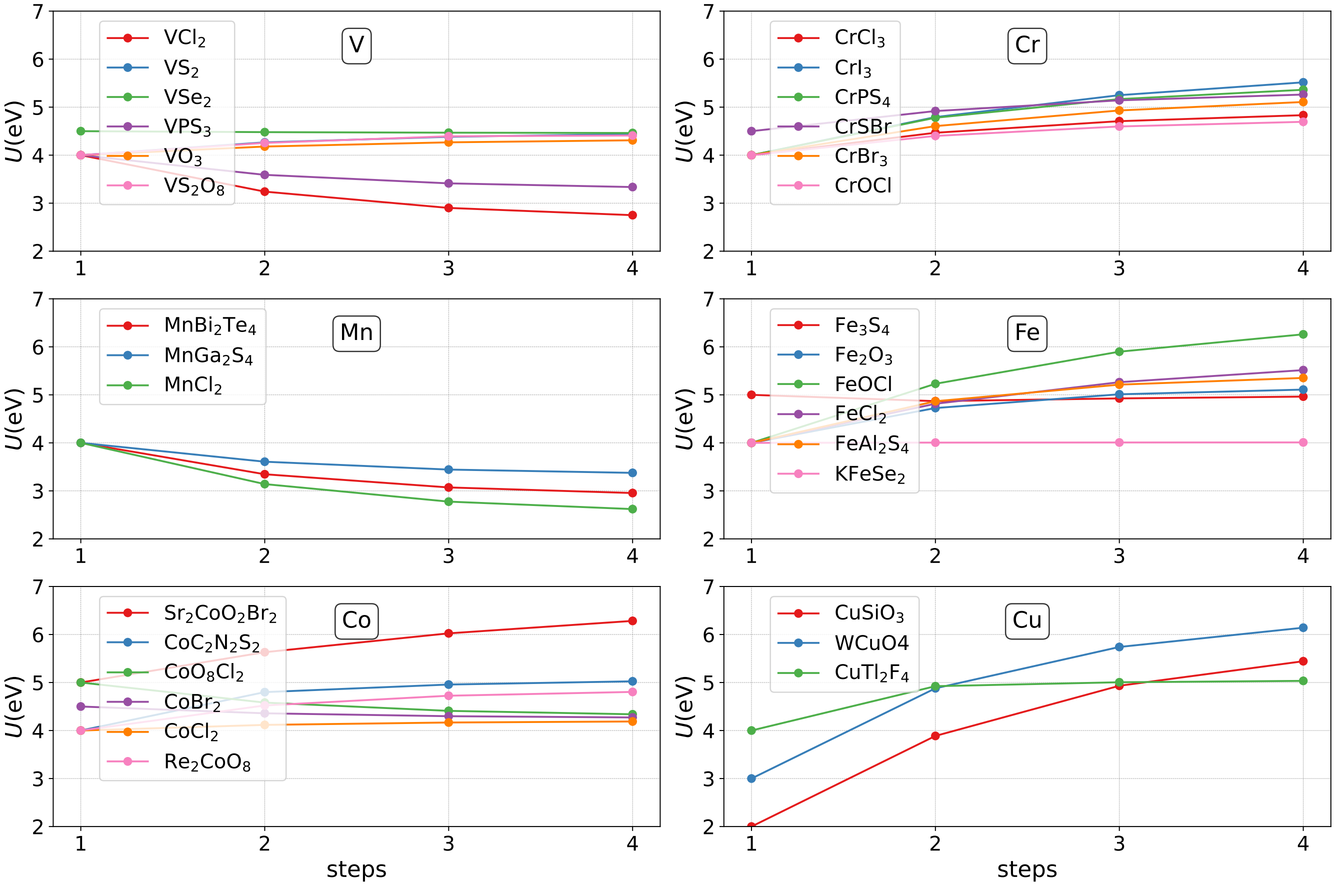


**Figure S2:** The convergence behavior of the Hubbard $U$ for V, Cr, Mn, Fe, Co, and Cu atoms in different magnetic monolayers.

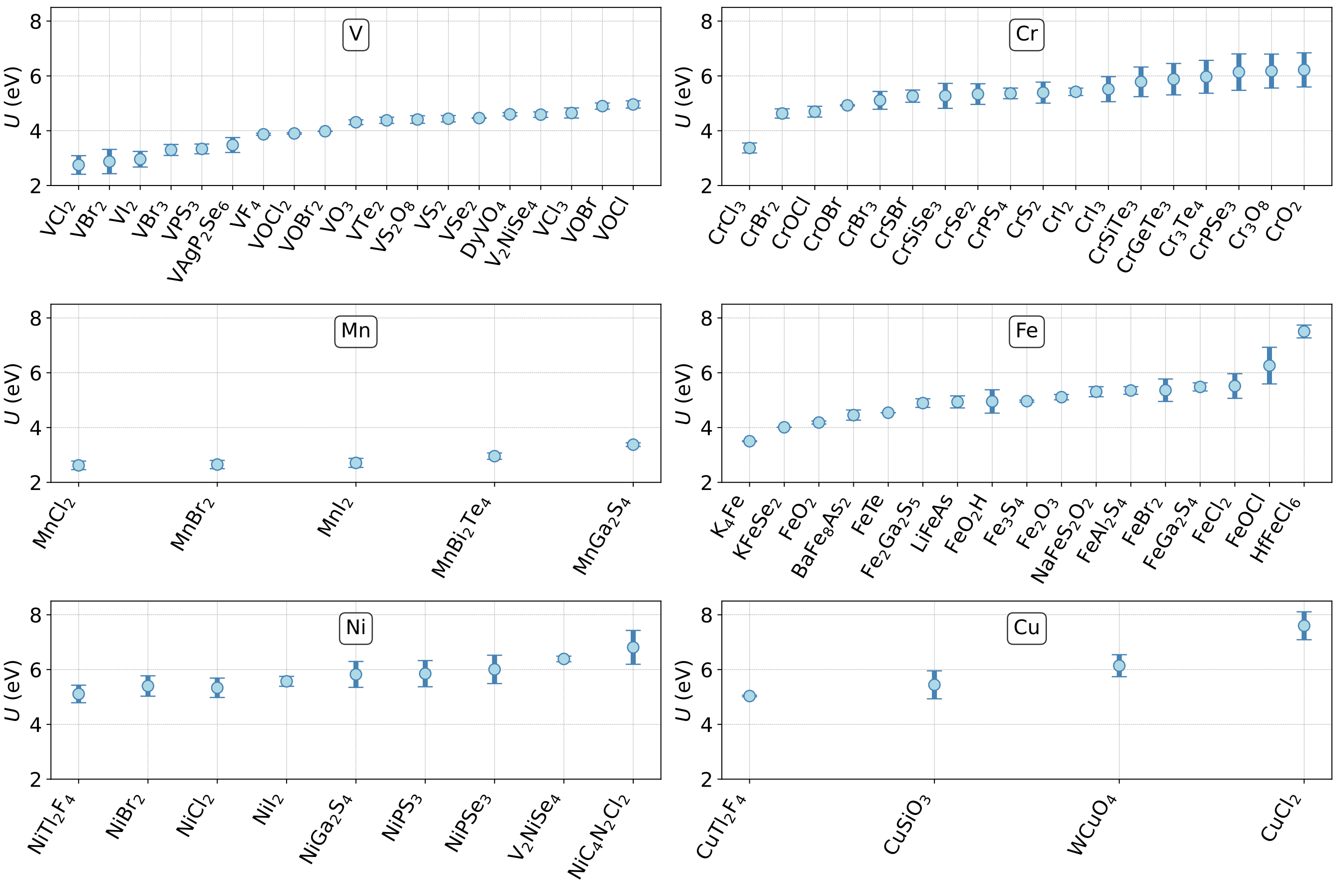


**Figure S3:** The values of Hubbard $U$ used to find the magnetic ground state of magnetic monolayers. The points show the Hubbard $U$ values calculated as the average of the error bars.

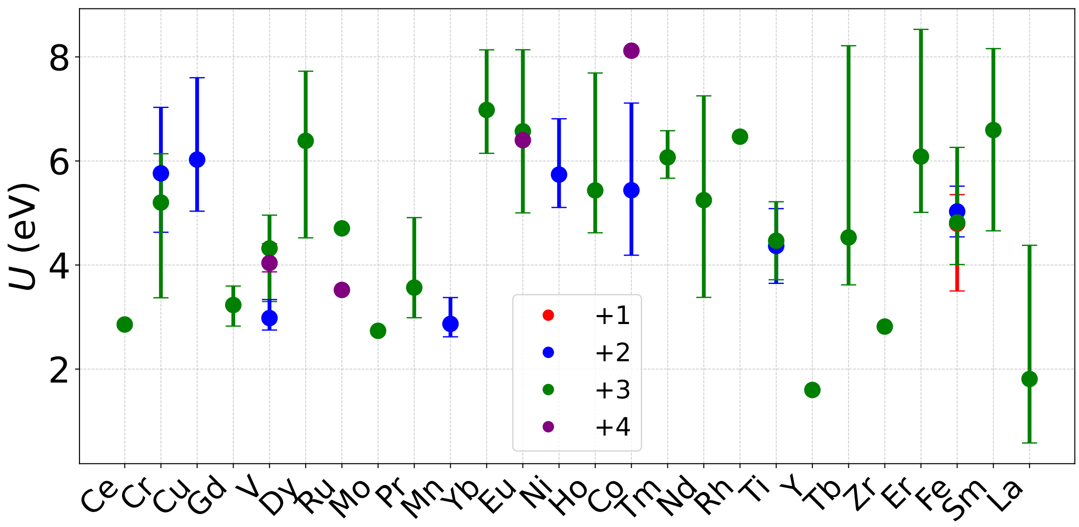


**Figure S4:** The Hubbard $U$ values and oxidation states for magnetic atoms, calculated using linear-response theory. Each point reports the average $U$ value for each atomic species, and the error bar indicates the minimum and maximum values observed across different materials. The color shows the oxidation states.

## S4. Dimerization in $MoCl_3$

As stated in the main text, in order to calculate the exchange parameter from the Heisenberg model, we extract the energy of the ground state and the closest opposite magnetic state. These two states must correspond to the same value of $S$, i.e. magnitude of the magnetic moment on the atoms. We note that there is always a small difference between the magnetic moment of the FM and AFM states in our calculations, but the difference is typically negligible (less than 0.1 $\mu_B$ in 98% of cases). The only system that shows the largest difference is $MoCl_3$, where the magnetic moment is 2.12 $\mu_B$ for the ground state (antiferromagnetic) and 1.76 $\mu_B$ for the closest opposite (ferromagnetic) state. This might be due to the dimerization in $MoCl_3$ where two nearest neighbors are closer to each other ($J_1$) than to the other ones ($J_2$) [3] (Figure S5), which may lead to different magnetic moments in the AFM state.

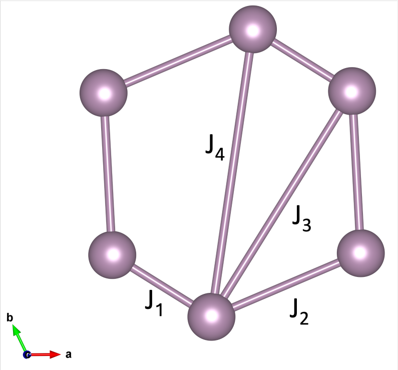


**Figure S5:** Distorted honeycomb lattice in $MoCl_3$, and the corresponding exchange parameter. For simplicity, only Mo atoms are shown.

## S5. Altermagnetic systems

Altermagnets are a special class of materials with symmetry enforced compensation in magnetization between spin-up and spin-down sublattices [4]. Unlike standard antiferromagnets, the two spin sublattices are connected by proper/improper rotations but not by symmetries like inversion or translation, and this gives rise to a finite splitting between spin-up and spin-down bands along certain directions in reciprocal space. Reference [5] introduces a symmetry-analysis tool that classifies three-dimensional materials as altermagnetic or antiferromagnetic based on their magnetic space-group symmetries. Although this tool was originally developed for bulk systems, we applied it to our two-dimensional structures by evaluating their magnetic space groups under the same symmetry operations. This analysis indicated that two systems in our dataset, $VF_4$ and $RuF_4$, satisfy the symmetry criteria for altermagnetism in 3D.

However, for two-dimensional materials, the reduced dimensionality imposes stricter symmetry constraints: any symmetry involving out-of-plane operations does not affect the 2D wave vector. As a result, in order to have altermagnetism the two spin sublattices should not be connected not only by inversion or translation as in 3D, but also by two-fold rotation around a vertical axis or by horizontal mirror reflection (possibly combined with a translation) [6, 7]. Thus, many magnetic space groups that can host altermagnetism in 3D do not support it in 2D, and only a restricted subset can give rise to altermagnetic order.

The two altermagnetic candidates, $VF_4$ and $RuF_4$, satisfy also these additional constraints and thus can be considered as 2D altermagnets. Indeed they were already identified by Sødequist et al. [6], who showed that magnetic monolayers with $P2_1'/c'$ symmetry can host altermagnetism. For the other altermagnetic candidates reported in Sødequist et al., either the materials are not contained in our database or their symmetries differ from those obtained in our magnetic calculations.

## S6. Structural, electronic, and magnetic information

In this section, we provide full details for all the magnetic monolayers with a link to the MC2D database. For each system we provide the results as following:

**TABLE S1:** Effective exchange parameters, $\tilde{J}$ (in meV), for the systems shown in Figure 6 of the main text.

| system | $\tilde{J}$ | system | $\tilde{J}$ | system | $\tilde{J}$ | system | $\tilde{J}$ |
|---|---|---|---|---|---|---|---|
| $ErCl_3$ | 0.004 | $GdBr_3$ | 0.47 | GdBr | 3.38 | $CrCl_3$ | 13.25 |
| YbSeF | 0.01 | $RuCl_3$ | 0.51 | $RuF_4$ | 3.58 | $VBr_2$ | 16.7 |
| NdSI | 0.01 | $MnI_2$ | 0.63 | $HoCl_3$ | 4.42 | $V_2NiSe_4$ | 17.14 |
| $HoI_3$ | 0.02 | GdSeI | 0.68 | CeOBr | 4.48 | $CrI_2$ | 17.59 |
| YbOCl | 0.03 | GdSI | 0.71 | $CrBr_2$ | 4.62 | $TiBr_2$ | 17.83 |
| $ErI_3$ | 0.04 | $WCuO_4$ | 0.76 | $MnBi_2Te_4$ | 4.74 | $CrBr_3$ | 18.61 |
| YbOBr | 0.05 | NdOBr | 0.82 | $NiGa_2S_4$ | 5.29 | $CoCl_2$ | 19.55 |
| ErSeI | 0.07 | $FeCl_2$ | 0.84 | $VAgP_2Se_6$ | 6.02 | $CoO_2$ | 21.5 |
| TbOBr | 0.07 | $NaN_3$ | 0.92 | $NiBr_2$ | 6.37 | $ZrCl_3$ | 22.59 |
| SmSI | 0.11 | EuIF | 0.95 | $NiCl_2$ | 6.38 | $NiTl_2F_4$ | 23.8 |
| $TmBr_3$ | 0.11 | TmSeI | 1.15 | $CuTl_2F_4$ | 6.69 | $Sr_2CoO_2Br_2$ | 27.13 |
| $ZrFeCl_6$ | 0.13 | $MnBr_2$ | 1.37 | $VCl_3$ | 6.88 | $CrPS_4$ | 27.98 |
| TmOI | 0.14 | $TiBr_3$ | 1.39 | YbSBr | 7.51 | $CrO_2$ | 29.62 |
| $PrI_3$ | 0.14 | $LiO_5Br_3$ | 1.46 | $VF_4$ | 7.79 | FeOCl | 30.72 |
| $TmI_3$ | 0.16 | $FeBr_2$ | 1.6 | $VOCl_2$ | 8.05 | $CrI_3$ | 31.68 |
| NdOI | 0.16 | $TiCl_3$ | 1.61 | $VCl_2O$ | 8.08 | VOCl | 32.08 |
| $GdCl_3$ | 0.17 | $MnCl_2$ | 1.7 | CrOCl | 8.32 | VOBr | 32.97 |
| $TbCl_3$ | 0.23 | $VI_2$ | 2.16 | ErSBr | 8.48 | CrSBr | 33.07 |
| ErSCl | 0.25 | $NdI_3$ | 2.17 | $NiI_2$ | 8.49 | $Fe_2Ga_2S_5$ | 33.41 |
| $GdI_3$ | 0.26 | $CoBr_2$ | 2.31 | $DyVO_4$ | 8.76 | $CrSiTe_3$ | 36.14 |
| ErSeBr | 0.26 | $CoC_2N_2S_2$ | 2.46 | $VBr_3$ | 8.76 | EuOBr | 39.37 |
| $CuSiO_3$ | 0.26 | $Gd_2GeI_2$ | 2.53 | $VCl_2$ | 8.86 | $CrGeTe_3$ | 39.97 |
| PrOI | 0.34 | $Gd_2GeBr_2$ | 2.57 | CrOBr | 9.19 | $PrI_2$ | 49.05 |
| $HfFeCl_6$ | 0.34 | $Gd_2CCl_2$ | 2.62 | $TiI_2$ | 11.63 | $TmCl_3$ | 67.93 |
| HoSI | 0.37 | GdOBr | 2.77 | $CoTl_2F_4$ | 11.88 | $GdI_2$ | 68.08 |
| $Re2CoO_8$ | 0.38 | $Re_2NiO_8$ | 2.81 | $CuCl_2$ | 12.33 | $VPS_3$ | 97.18 |
| $CoO_8Cl_2$ | 0.39 | $Co(OH)_2$ | 2.96 | TmOBr | 12.63 | $MoCl_3$ | 119.57 |
| $DyAsO_4$ | 0.41 | $TbH_2Br$ | 2.96 | EuHI | 12.98 | | |
| $RhN_4Cl_6$ | 0.45 | DySI | 3.22 | $NiPSe_3$ | 13.17 | | |
| $VOBr_2$ | 0.47 | $FeI_2$ | 3.3 | $NiPS_3$ | 13.18 | | |

- **Geometry:** the view of the system from $x$, $y$ and $z$ axis are shown.

- **Electronic bandstructure:** Energy bands of the systems are given along high symmetry points of the first Brillouin zone in a representative window around Fermi energy. The bands for spin up and down are given in different colors.

- **Unique states:** For each system, we provide a figure representing the self-consistent unique states [8] of the energy landscape identified using RomeoDFT. Each point shows a unique self-consistent state, with its color denoting the average distance ($\eta$) of the occupied bands of that state and the ground state (as defined in Equation 5 of reference [9]). The $y$ axis shows the energy difference of the state with respect to the ground state energy, and the $x$ axis shows the sum of atomic magnetic moments in that state. The red dot represents the state obtained from an unconstrained QE calculation (vanilla QE).

- **Lattice vectors:** Cartesian components of the lattice vectors are given in Å. Note that because the Chronos workflow entails geometry optimization, there is a small geometrical difference between our structures and the one on MC2D.

- **Atomic positions:** Atomic coordinates, Hubbard $U$ (in eV), and magnetic moments of atoms are provided in this part. The magnetic moments are calculated by two methods: 1) by integrating the spin up and down density over a sphere, ($m_i = \int(\rho^{I,\uparrow}(\mathbf{r}) - \rho^{I,\downarrow}(\mathbf{r}))d\mathbf{r}$, and is shown by $\mathrm{m}_{in}$, and 2) using occupation matrix for spin up and down that is achieved by projecting Kohn-Sham wavefunctions on atomic orbitals, $m_o = \sum_m \left(n^{I\uparrow}_{mm} - n^{I\downarrow}_{mm}\right)$, and is shown by $\mathrm{m}_o$. The total and absolute magnetization are calculated using the integration of spin-up and spin-down charge density. Figure S6 compares the magnetic moments obtained from the two methods for different atoms across various materials. The results show good agreement between the two approaches. The only noticeable discrepancy appears for nitrogen, which may arise from the small predefined radius of the integration sphere used for the nitrogen atom.

As discussed in the main text, one limitation of the RomeoDFT workflow for some materials is an insufficient number of identified minima. This issue arises primarily from two factors: the stopping condition of the algorithm and convergence issues. In RomeoDFT, the new generation of trial target occupations are the mean value of the occupation matrices of the previous calculations. This process is repeated, and newly discovered states are used to create new target occupations until the ratio of newly discovered unique states to the number of trials falls below a given threshold. If no new states are identified after several iterations, the process stops. In our dataset, for 38 systems, the number of identified unique states is less than 10 unique states. Out of these, 27 stopped because the termination condition of Romeo was met, i.e, the number of newly discovered states in the last generation to the total number of trials is below the default convergence threshold. These systems are: $BaFe_8As_2$, CdOCl, $Cr_3Te_4$, $CrCl_3$ (Cm), $CrPSe_3$, $CuO_2$ ($\bar{P}1$), $DyBr_3$, $DyI_3$, $EuBr_3$, $EuOBr_2$, $Fe_2O_3$, $Fe_3S_4$, GaSe, $HoBr_3$, HoOBr, HoSeI, $KPtNCl_3$, $LaNb_2O_7$, $MnBi_2Te_4$, $PrBr_3$, ScCl, $SmI_3$, $TbBr_3$ (C2/m), $TmBr_3$, $TmCl_3$, $TmI_3$, and $YbCl_3$ (C2/m). For the remaining 11 systems, the workflow did not stop due to the stop condition but rather because of technical issues in RomeoDFT. In these cases, the algorithm unexpectedly generates an excessively large number of target occupations (on the order of thousands), causing the procedure to diverge; such calculations were manually terminated. Nevertheless, each of these systems still includes several hundred completed trials, so it is highly likely that the true ground state was reached. In other cases, the workflow terminated due to what appears to be an internal error rather than convergence. These systems are: LaI, $TmI_2$, $VI_2$, $VO_3$, and YI. In a few additional cases, the workflow terminates due to internal errors rather than the stopping condition. These materials are $CrBr_2$, $Cu_2O_3Cl$, GdCBr, $GdCl_3$, $WCuO_4$, and $YbI_3$. Overall, the combination of the stopping condition and convergence issues for certain target occupations can result in a limited number of identified minima for some systems.

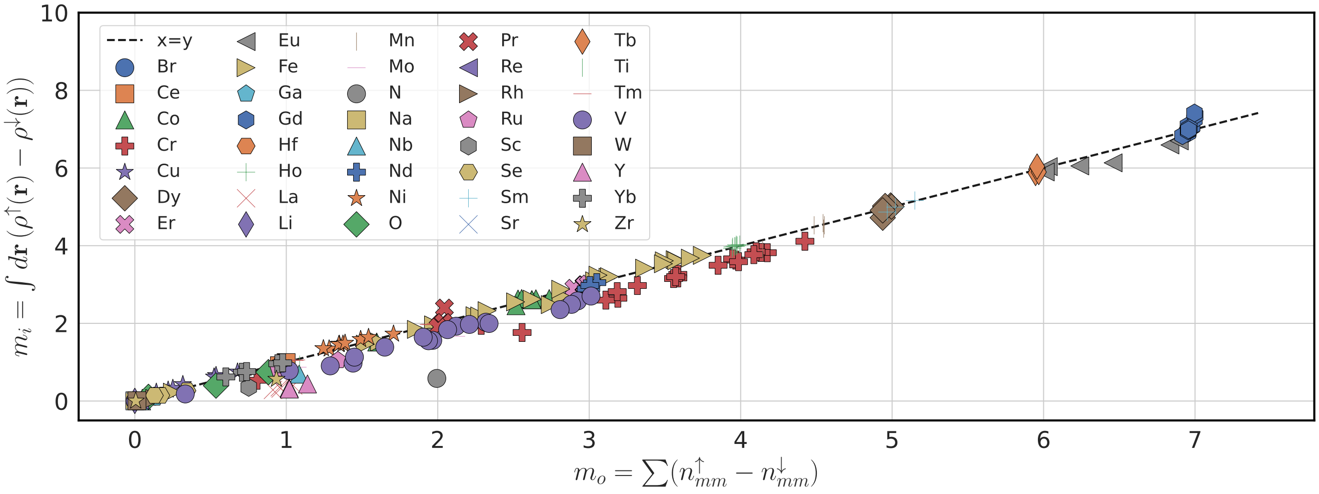


**Figure S6:** Magnetic moment of different atoms in different systems computed from two methods: integration of the magnetization density over a sphere around each atom ($m_i$, vertical axis) or from the difference in the trace of the occupation matrices for spin up and down ($m_o$, horizontal axis).

## $BaFe_8As_2$ (FM)

Band gap: 0.0 eV
Total magnetization: $-22.41\ \mu_B/cell$
Absolute magnetization: $24.04\ \mu_B/cell$
MC2D entry: https://mc2d.materialscloud.org/#/details/mc2d-2069

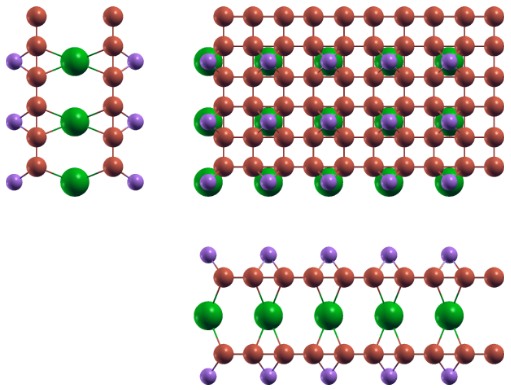

**Geometry:** Views of $BaFe_8As_2$ as seen from the $x$ axis (left), the $y$ axis (bottom), and the $z$ axis (center).

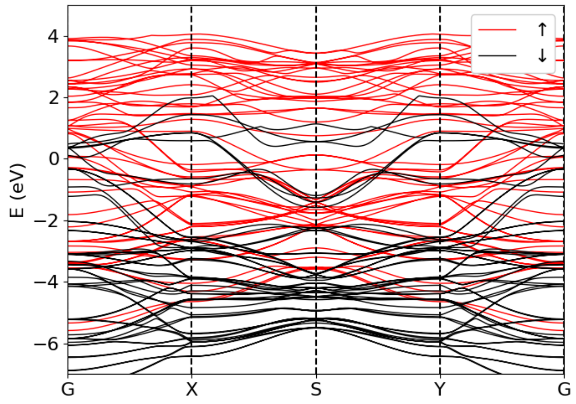


**Electronic bandstructure:** Spin-resolved energy bands of monolayer $BaFe_8As_2$ along a high-symmetry path.

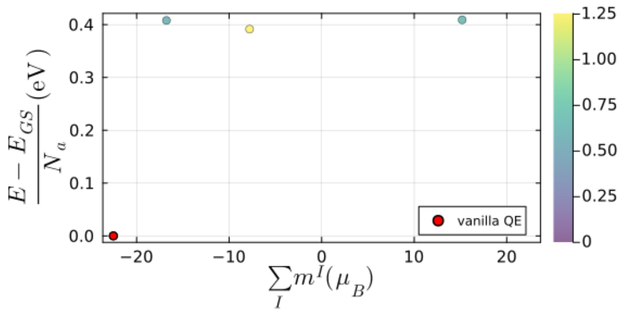


**Unique states:** Representation of 5 self-consistent unique states for monolayer $BaFe_8As_2$ identified using RomeoDFT (see Section S6).

**Lattice vectors:** Cartesian components (in [Å]) of the lattice vectors for $BaFe_8As_2$.

| | $x$ | $y$ | $z$ |
|---|---|---|---|
| $\boldsymbol{a_1}$ | 4.6548 | 0.0000 | 0.0000 |
| $\boldsymbol{a_2}$ | 0.0000 | 4.6548 | 0.0000 |
| $\boldsymbol{a_3}$ | 0.0000 | 0.0000 | 30.5381 |

**Atomic positions:** Fractional coordinates, Hubbard $U$ (in eV) and magnetic moments (in $\mu_B$, computed from orbital occupations $m_o$ or integration spheres $m_i$) of each atom of monolayer $BaFe_8As_2$.

| atom | $x$ | $y$ | $z$ | $U$ | $m_o$ | $m_i$ |
|---|---|---|---|---|---|---|
| Fe | 0.75 | −0.75 | 0.10 | 4.45 | −2.81 | −2.70 |
| Fe | 0.25 | −0.75 | 0.10 | 4.45 | −2.81 | −2.70 |
| Fe | 0.75 | −0.25 | 0.10 | 4.45 | −2.81 | −2.70 |
| Fe | 0.25 | −0.25 | 0.10 | 4.45 | −2.81 | −2.70 |
| Fe | 0.75 | −0.75 | −0.10 | 4.45 | −2.81 | −2.70 |
| Fe | 0.25 | −0.75 | −0.10 | 4.45 | −2.81 | −2.70 |
| Fe | 0.75 | −0.25 | −0.10 | 4.45 | −2.81 | −2.70 |
| Fe | 0.25 | −0.25 | −0.10 | 4.45 | −2.81 | −2.70 |
| As | 0.0 | 0.0 | 0.15 | 0.0 | – | 0.11 |
| Ba | 0.0 | 0.0 | 0.0 | 0.0 | – | 0.06 |
| As | 0.0 | 0.0 | −0.15 | 0.0 | – | 0.11 |

## CdOCl (FM)

Band gap: 0.13 eV
Total magnetization: −2.15 $\mu_B/cell$
Absolute magnetization: 2.32 $\mu_B/cell$
MC2D entry: https://mc2d.materialscloud.org/#/details/mc2d-83

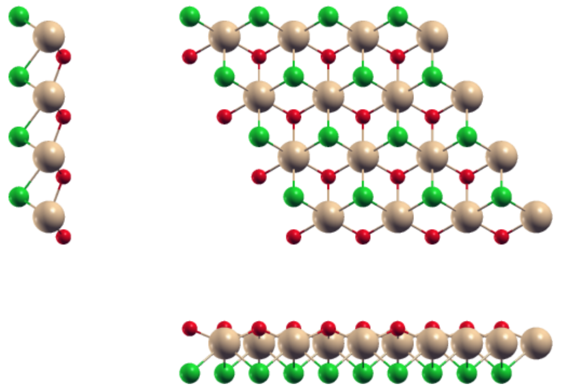

**Geometry:** Views of CdOCl as seen from the $x$ axis (left), the $y$ axis (bottom), and the $z$ axis (center).

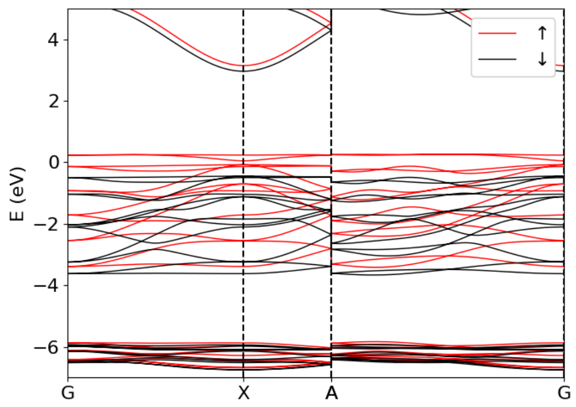


**Electronic bandstructure:** Spin-resolved energy bands of monolayer CdOCl along a high-symmetry path.

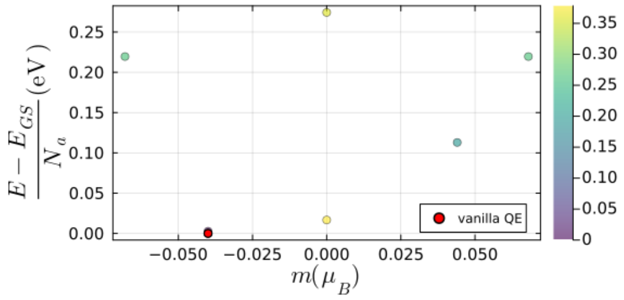


**Unique states:** Representation of 7 self-consistent unique states for monolayer CdOCl identified using RomeoDFT (see Section S6).

**Lattice vectors:** Cartesian components (in [Å]) of the lattice vectors for CdOCl.

| | $x$ | $y$ | $z$ |
|---|---|---|---|
| $\boldsymbol{a_1}$ | 7.4624 | 0.0000 | 0.0000 |
| $\boldsymbol{a_2}$ | −1.8656 | 3.2313 | 0.0000 |
| $\boldsymbol{a_3}$ | 0.0000 | 0.0000 | 22.4758 |

**Atomic positions:** Fractional coordinates, Hubbard $U$ (in eV) and magnetic moments (in $\mu_B$, computed from orbital occupations $m_o$ or integration spheres $m_i$) of each atom of monolayer CdOCl.

| atom | $x$ | $y$ | $z$ | $U$ | $m_o$ | $m_i$ |
|---|---|---|---|---|---|---|
| Cd | 0.33 | 0.33 | 0.48 | 0.00 | −0.02 | −0.03 |
| Cd | 0.83 | 0.33 | 0.48 | 0.00 | −0.02 | −0.03 |
| Cl | 0.17 | 0.67 | 0.55 | 0.0 | – | −0.18 |
| O | 0.0 | 0.0 | 0.44 | 0.0 | – | −0.78 |
| Cl | 0.67 | 0.67 | 0.55 | 0.0 | – | −0.18 |
| O | 0.50 | 0.0 | 0.44 | 0.0 | – | −0.78 |

## CeOBr (FM)

Band gap: 2.11 eV
Total magnetization: $-2.0\ \mu_B/cell$
Absolute magnetization: $2.14\ \mu_B/cell$
MC2D entry: https://mc2d.materialscloud.org/#/details/mc2d-2189

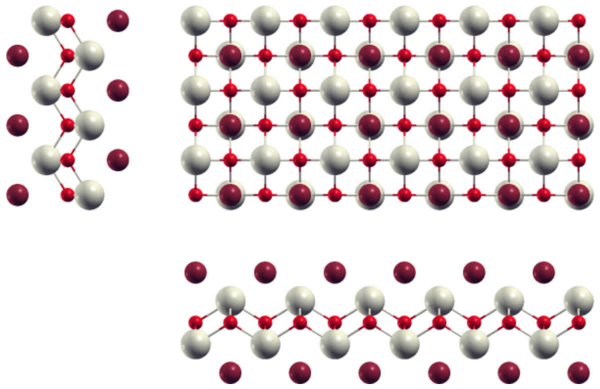

**Geometry:** Views of CeOBr as seen from the $x$ axis (left), the $y$ axis (bottom), and the $z$ axis (center).

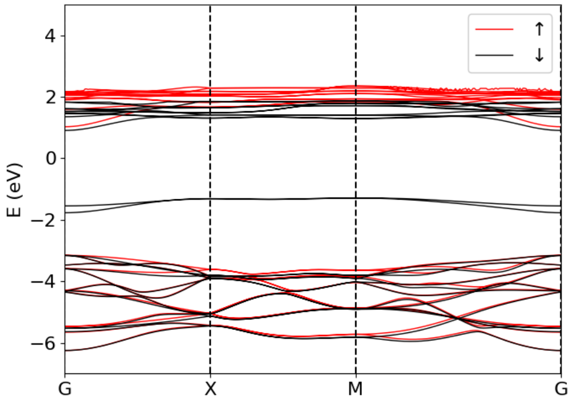


**Electronic bandstructure:** Spin-resolved energy bands of monolayer CeOBr along a high-symmetry path.

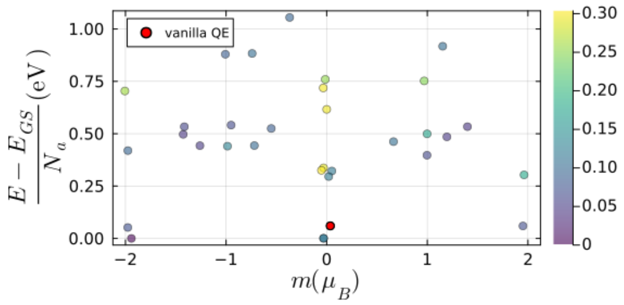


**Unique states:** Representation of 32 self-consistent unique states for monolayer CeOBr identified using RomeoDFT (see Section S6).

**Lattice vectors:** Cartesian components (in [Å]) of the lattice vectors for CeOBr.

| | $x$ | $y$ | $z$ |
|---|---|---|---|
| $\boldsymbol{a_1}$ | 3.9939 | 0.0000 | 0.0000 |
| $\boldsymbol{a_2}$ | 0.0000 | 3.9939 | 0.0000 |
| $\boldsymbol{a_3}$ | 0.0000 | 0.0000 | 22.6899 |

**Atomic positions:** Fractional coordinates, Hubbard $U$ (in eV) and magnetic moments (in $\mu_B$, computed from orbital occupations $m_o$ or integration spheres $m_i$) of each atom of monolayer CeOBr.

| atom | $x$ | $y$ | $z$ | $U$ | $m_o$ | $m_i$ |
|---|---|---|---|---|---|---|
| Ce | 0.25 | −0.25 | 0.05 | 2.80 | −0.95 | −0.91 |
| Ce | −0.25 | −0.75 | −0.05 | 2.80 | −0.99 | −0.93 |
| Br | −0.25 | −0.75 | 0.13 | 0.0 | – | 0.00 |
| Br | 0.25 | −0.25 | −0.13 | 0.0 | – | 0.00 |
| O | −0.25 | −0.25 | 0.0 | 0.0 | – | 0.01 |
| O | 0.25 | −0.75 | 0.0 | 0.0 | – | 0.01 |

## CeSiI (FM)

Band gap: 0.0 eV
Total magnetization: 2.08 $\mu_B/cell$
Absolute magnetization: 2.23 $\mu_B/cell$
MC2D entry: https://mc2d.materialscloud.org/#/details/mc2d-86

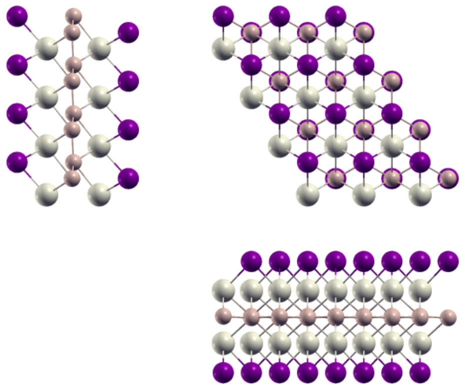

**Geometry:** Views of CeSiI as seen from the $x$ axis (left), the $y$ axis (bottom), and the $z$ axis (center).

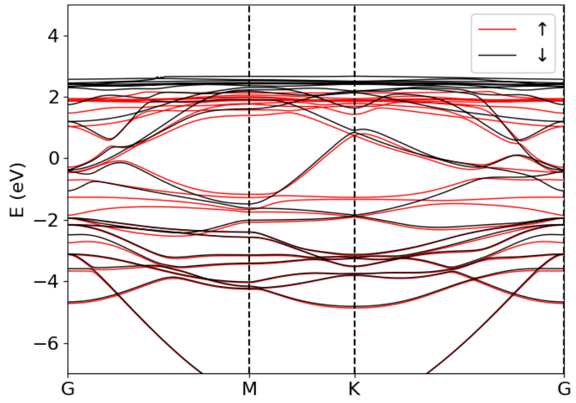


**Electronic bandstructure:** Spin-resolved energy bands of monolayer CeSiI along a high-symmetry path.

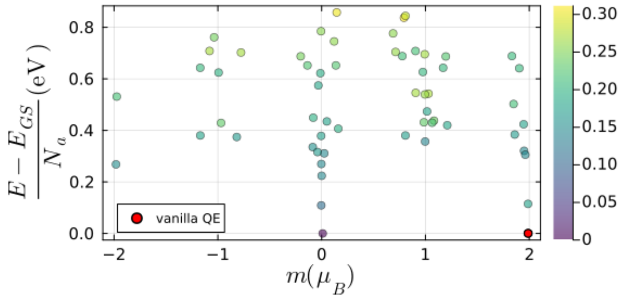


**Unique states:** Representation of 59 self-consistent unique states for monolayer CeSiI identified using RomeoDFT (see Section S6).

**Lattice vectors:** Cartesian components (in [Å]) of the lattice vectors for CeSiI.

| | $x$ | $y$ | $z$ |
|---|---|---|---|
| $\boldsymbol{a_1}$ | 4.1556 | 0.0000 | 0.0000 |
| $\boldsymbol{a_2}$ | −2.0778 | 3.5989 | 0.0000 |
| $\boldsymbol{a_3}$ | 0.0000 | 0.0000 | 28.2829 |

**Atomic positions:** Fractional coordinates, Hubbard $U$ (in eV) and magnetic moments (in $\mu_B$, computed from orbital occupations $m_o$ or integration spheres $m_i$) of each atom of monolayer CeSiI.

| atom | $x$ | $y$ | $z$ | $U$ | $m_o$ | $m_i$ |
|---|---|---|---|---|---|---|
| Ce | 0.0 | 0.0 | 0.57 | 2.91 | 1.00 | 0.99 |
| Ce | 0.0 | 0.0 | 0.43 | 2.91 | 1.00 | 0.99 |
| Si | 0.67 | 0.33 | 0.50 | 0.0 | – | −0.01 |
| I | 0.33 | 0.67 | 0.65 | 0.0 | – | −0.01 |
| Si | 0.33 | 0.67 | 0.50 | 0.0 | – | −0.01 |
| I | 0.67 | 0.33 | 0.35 | 0.0 | – | −0.01 |

## $Co(OH)_2$ (AFM)

Band gap: 1.22 eV
Total magnetization: 0.0 $\mu_B/cell$
Absolute magnetization: 5.86 $\mu_B/cell$
MC2D entry: https://mc2d.materialscloud.org/#/details/mc2d-127

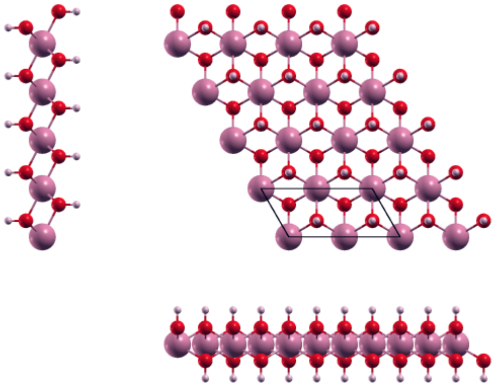

**Geometry:** Views of $Co(OH)_2$ as seen from the $x$ axis (left), the $y$ axis (bottom), and the $z$ axis (center).

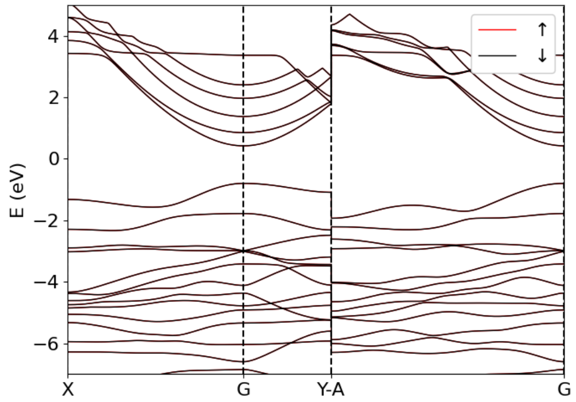


**Electronic bandstructure:** Spin-resolved energy bands of monolayer $Co(OH)_2$ along a high-symmetry path.

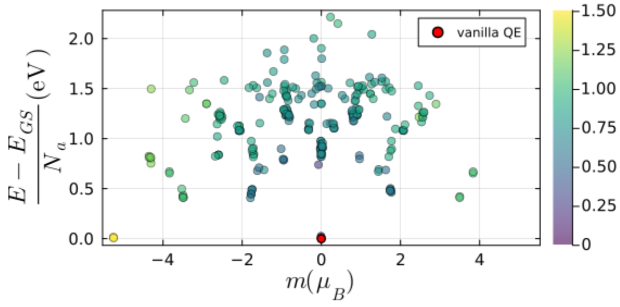


**Unique states:** Representation of 254 self-consistent unique states for monolayer $Co(OH)_2$ identified using RomeoDFT (see Section S6).

**Lattice vectors:** Cartesian components (in [Å]) of the lattice vectors for $Co(OH)_2$.

| | $x$ | $y$ | $z$ |
|---|---|---|---|
| $\boldsymbol{a_1}$ | 6.2236 | 0.0000 | 0.0000 |
| $\boldsymbol{a_2}$ | −1.5559 | 2.6949 | 0.0000 |
| $\boldsymbol{a_3}$ | 0.0000 | 0.0000 | 23.8142 |

**Atomic positions:** Fractional coordinates, Hubbard $U$ (in eV) and magnetic moments (in $\mu_B$, computed from orbital occupations $m_o$ or integration spheres $m_i$) of each atom of monolayer $Co(OH)_2$.

| atom | $x$ | $y$ | $z$ | $U$ | $m_o$ | $m_i$ |
|---|---|---|---|---|---|---|
| Co | 0.0 | 0.0 | 0.50 | 6.84 | −2.62 | −2.61 |
| Co | 0.50 | 0.0 | 0.50 | 6.84 | 2.62 | 2.61 |
| H | 0.17 | 0.67 | 0.42 | 0.0 | – | 0.00 |
| O | 0.17 | 0.67 | 0.46 | 0.0 | – | −0.01 |
| H | 0.33 | 0.33 | 0.58 | 0.0 | – | 0.00 |
| O | 0.33 | 0.33 | 0.54 | 0.0 | – | 0.01 |
| H | 0.67 | 0.67 | 0.42 | 0.0 | – | 0.00 |
| O | 0.67 | 0.67 | 0.46 | 0.0 | – | 0.01 |
| H | 0.83 | 0.33 | 0.58 | 0.0 | – | 0.00 |
| O | 0.83 | 0.33 | 0.54 | 0.0 | – | −0.01 |

## $CoBr_2$ (FM)

Band gap: 2.19 eV
Total magnetization: $-6.0\ \mu_B/cell$
Absolute magnetization: $6.06\ \mu_B/cell$
MC2D entry: https://mc2d.materialscloud.org/#/details/mc2d-31

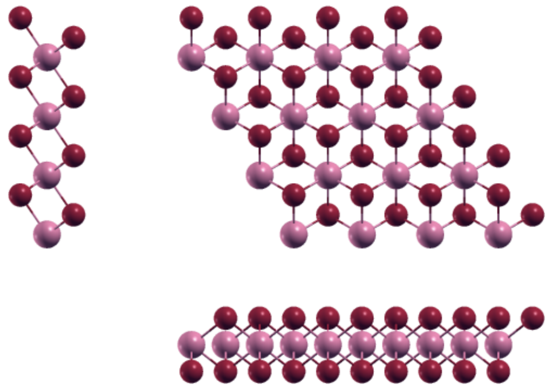

**Geometry:** Views of $CoBr_2$ as seen from the $x$ axis (left), the $y$ axis (bottom), and the $z$ axis (center).

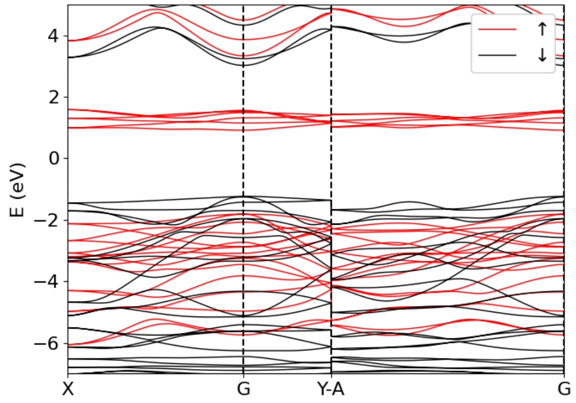


**Electronic bandstructure:** Spin-resolved energy bands of monolayer $CoBr_2$ along a high-symmetry path.

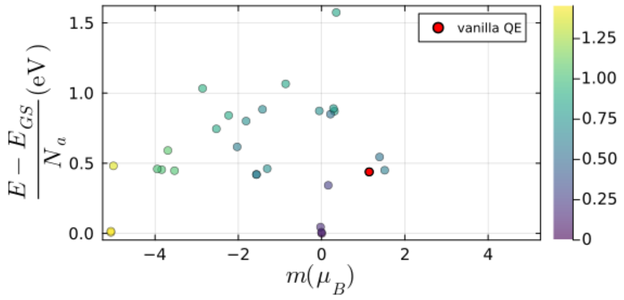


**Unique states:** Representation of 30 self-consistent unique states for monolayer $CoBr_2$ identified using RomeoDFT (see Section S6).

**Lattice vectors:** Cartesian components (in [Å]) of the lattice vectors for $CoBr_2$.

| | $x$ | $y$ | $z$ |
|---|---|---|---|
| $\boldsymbol{a_1}$ | 7.4130 | 0.0000 | 0.0000 |
| $\boldsymbol{a_2}$ | −1.8533 | 3.2099 | 0.0000 |
| $\boldsymbol{a_3}$ | 0.0000 | 0.0000 | 22.7404 |

**Atomic positions:** Fractional coordinates, Hubbard $U$ (in eV) and magnetic moments (in $\mu_B$, computed from orbital occupations $m_o$ or integration spheres $m_i$) of each atom of monolayer $CoBr_2$.

| atom | $x$ | $y$ | $z$ | $U$ | $m_o$ | $m_i$ |
|---|---|---|---|---|---|---|
| Co | 0.0 | 0.0 | 0.50 | 4.27 | −2.53 | −2.63 |
| Co | 0.50 | 0.0 | 0.50 | 4.27 | −2.53 | −2.63 |
| Br | 0.17 | 0.67 | 0.56 | 0.0 | – | −0.11 |
| Br | 0.33 | 0.33 | 0.44 | 0.0 | – | −0.12 |
| Br | 0.67 | 0.67 | 0.56 | 0.0 | – | −0.12 |
| Br | 0.83 | 0.33 | 0.44 | 0.0 | – | −0.11 |

## $CoC_2N_2S_2$ (FM)

Band gap: 0.44 eV
Total magnetization: $-2.0\ \mu_B/cell$
Absolute magnetization: 2.33 $\mu_B/cell$
MC2D entry: https://mc2d.materialscloud.org/#/details/mc2d-1258

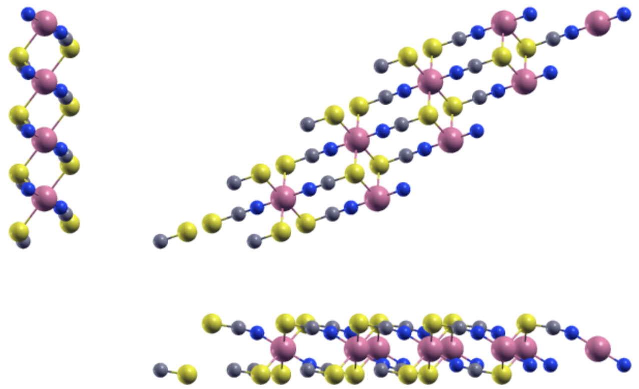

**Geometry:** Views of $CoC_2N_2S_2$ as seen from the $x$ axis (left), the $y$ axis (bottom), and the $z$ axis (center).

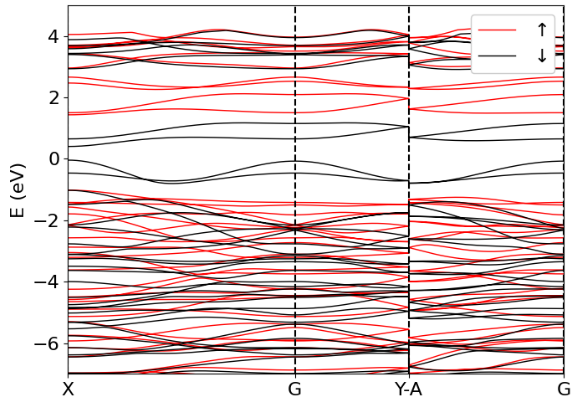


**Electronic bandstructure:** Spin-resolved energy bands of monolayer $CoC_2N_2S_2$ along a high-symmetry path.

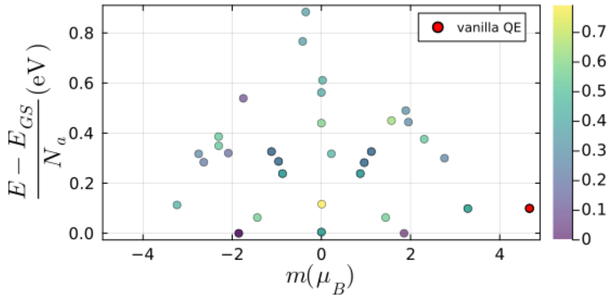


**Unique states:** Representation of 41 self-consistent unique states for monolayer $CoC_2N_2S_2$ identified using RomeoDFT (see Section S6).

**Lattice vectors:** Cartesian components (in [Å]) of the lattice vectors for $CoC_2N_2S_2$.

| | $x$ | $y$ | $z$ |
|---|---|---|---|
| $\boldsymbol{a_1}$ | 10.9031 | 0.0000 | 0.0000 |
| $\boldsymbol{a_2}$ | 4.2690 | 3.3905 | 0.0000 |
| $\boldsymbol{a_3}$ | 0.0000 | 0.0000 | 22.9605 |

**Atomic positions:** Fractional coordinates, Hubbard $U$ (in eV) and magnetic moments (in $\mu_B$, computed from orbital occupations $m_o$ or integration spheres $m_i$) of each atom of monolayer $CoC_2N_2S_2$.

| atom | $x$ | $y$ | $z$ | $U$ | $m_o$ | $m_i$ |
|---|---|---|---|---|---|---|
| Co | 0.40 | 0.81 | 0.50 | 5.02 | −0.93 | −0.90 |
| Co | 0.90 | 0.81 | 0.50 | 5.02 | −0.93 | −0.90 |
| C | 0.04 | 0.08 | 0.55 | 0.0 | – | 0.00 |
| S | 0.12 | 0.24 | 0.56 | 0.0 | – | 0.00 |
| N | 0.48 | 0.97 | 0.54 | 0.0 | – | 0.00 |
| C | 0.27 | 0.54 | 0.45 | 0.0 | – | 0.00 |
| S | 0.19 | 0.38 | 0.44 | 0.0 | – | 0.00 |
| N | 0.33 | 0.65 | 0.46 | 0.0 | – | 0.00 |
| C | 0.54 | 0.08 | 0.55 | 0.0 | – | 0.00 |
| S | 0.62 | 0.24 | 0.56 | 0.0 | – | 0.00 |
| N | 0.98 | 0.97 | 0.54 | 0.0 | – | 0.00 |
| C | 0.77 | 0.54 | 0.45 | 0.0 | – | 0.00 |
| S | 0.69 | 0.38 | 0.44 | 0.0 | – | 0.00 |
| N | 0.83 | 0.65 | 0.46 | 0.0 | – | 0.00 |

# $CoCl_2$ (AFM)

Band gap: 2.72 eV
Total magnetization: 0.0 $\mu_B/cell$
Absolute magnetization: 5.82 $\mu_B/cell$
MC2D entry: https://mc2d.materialscloud.org/#/details/mc2d-87

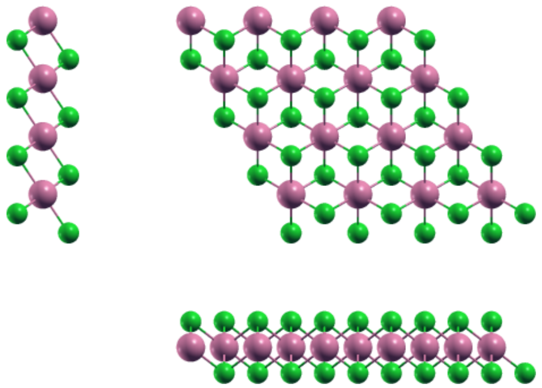

**Geometry:** Views of $CoCl_2$ as seen from the $x$ axis (left), the $y$ axis (bottom), and the $z$ axis (center).

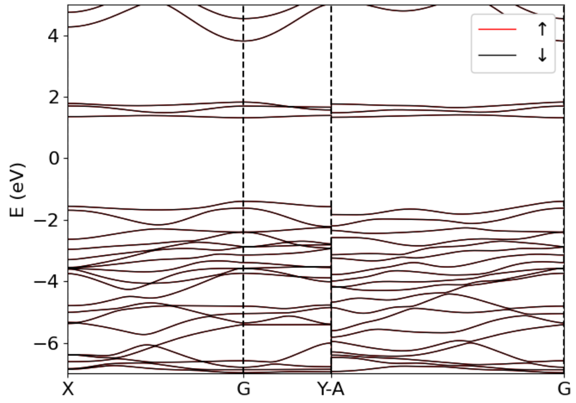


**Electronic bandstructure:** Spin-resolved energy bands of monolayer $CoCl_2$ along a high-symmetry path.

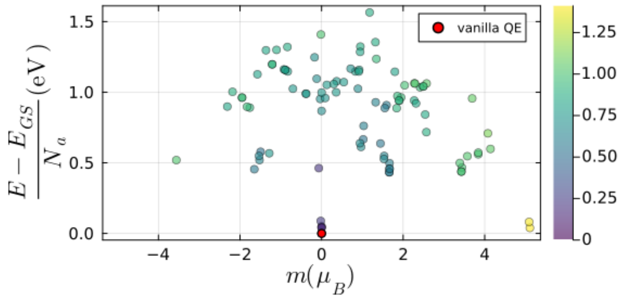


**Unique states:** Representation of 98 self-consistent unique states for monolayer $CoCl_2$ identified using RomeoDFT (see Section S6).

**Lattice vectors:** Cartesian components (in [Å]) of the lattice vectors for $CoCl_2$.

| | $x$ | $y$ | $z$ |
|---|---|---|---|
| $\boldsymbol{a_1}$ | 7.0040 | 0.0000 | 0.0000 |
| $\boldsymbol{a_2}$ | −1.7510 | 3.0328 | 0.0000 |
| $\boldsymbol{a_3}$ | 0.0000 | 0.0000 | 22.5807 |

**Atomic positions:** Fractional coordinates, Hubbard $U$ (in eV) and magnetic moments (in $\mu_B$, computed from orbital occupations $m_o$ or integration spheres $m_i$) of each atom of monolayer $CoCl_2$.

| atom | $x$ | $y$ | $z$ | $U$ | $m_o$ | $m_i$ |
|---|---|---|---|---|---|---|
| Co | 0.17 | 0.67 | 0.50 | 4.19 | 2.55 | 2.64 |
| Co | 0.67 | 0.67 | 0.50 | 4.19 | −2.55 | −2.64 |
| Cl | 0.33 | 0.33 | 0.56 | 0.0 | – | 0.04 |
| Cl | 0.0 | 0.0 | 0.44 | 0.0 | – | 0.04 |
| Cl | 0.83 | 0.33 | 0.56 | 0.0 | – | −0.04 |
| Cl | 0.50 | 0.0 | 0.44 | 0.0 | – | −0.04 |

## $CoO_2$ (FM)

Band gap: 1.20 eV
Total magnetization: $-2.0\ \mu_B/cell$
Absolute magnetization: $4.69\ \mu_B/cell$
MC2D entry: https://mc2d.materialscloud.org/#/details/mc2d-129

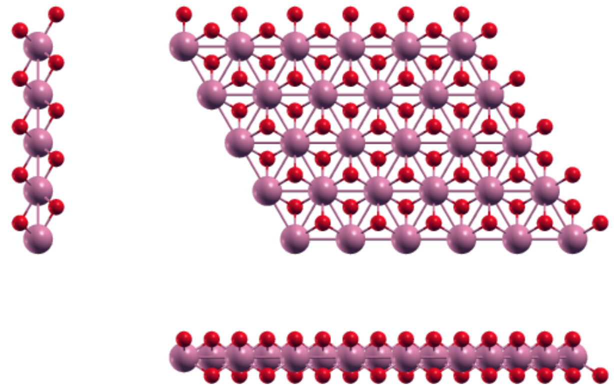

**Geometry:** Views of $CoO_2$ as seen from the $x$ axis (left), the $y$ axis (bottom), and the $z$ axis (center).

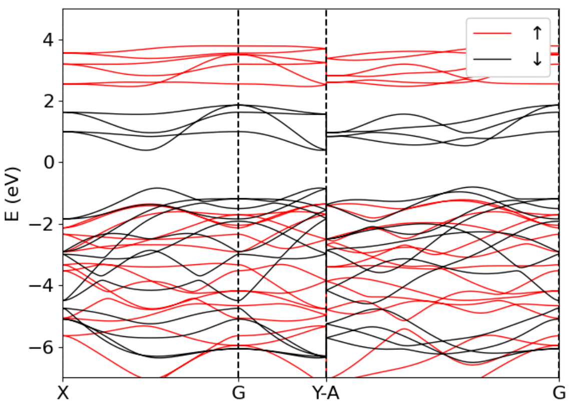


**Electronic bandstructure:** Spin-resolved energy bands of monolayer $CoO_2$ along a high-symmetry path.

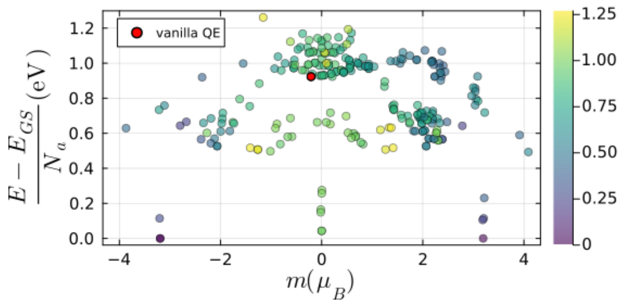


**Unique states:** Representation of 258 self-consistent unique states for monolayer $CoO_2$ identified using RomeoDFT (see Section S6).

**Lattice vectors:** Cartesian components (in [Å]) of the lattice vectors for $CoO_2$.

| | $x$ | $y$ | $z$ |
|---|---|---|---|
| $\boldsymbol{a_1}$ | 5.6396 | 0.0000 | 0.0000 |
| $\boldsymbol{a_2}$ | −1.4099 | 2.4420 | 0.0000 |
| $\boldsymbol{a_3}$ | 0.0000 | 0.0000 | 21.8656 |

**Atomic positions:** Fractional coordinates, Hubbard $U$ (in eV) and magnetic moments (in $\mu_B$, computed from orbital occupations $m_o$ or integration spheres $m_i$) of each atom of monolayer $CoO_2$.

| atom | $x$ | $y$ | $z$ | $U$ | $m_o$ | $m_i$ |
|---|---|---|---|---|---|---|
| Co | 0.0 | 0.0 | 0.50 | 8.12 | −1.60 | −1.52 |
| Co | 0.50 | 0.0 | 0.50 | 8.12 | −1.60 | −1.52 |
| O | 0.17 | 0.67 | 0.46 | 0.0 | – | 0.30 |
| O | 0.33 | 0.33 | 0.54 | 0.0 | – | 0.30 |
| O | 0.67 | 0.67 | 0.46 | 0.0 | – | 0.30 |
| O | 0.83 | 0.33 | 0.54 | 0.0 | – | 0.30 |

## $CoO_8Cl_2$ (FM)

Band gap: 3.48 eV
Total magnetization: $-6.0\ \mu_B/cell$
Absolute magnetization: $6.09\ \mu_B/cell$
MC2D entry: https://mc2d.materialscloud.org/#/details/mc2d-1207

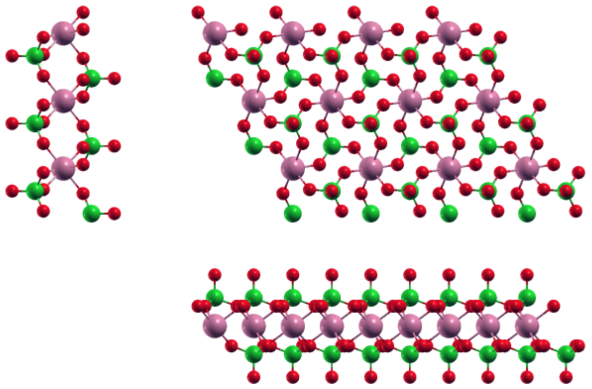

**Geometry:** Views of $CoO_8Cl_2$ as seen from the $x$ axis (left), the $y$ axis (bottom), and the $z$ axis (center).

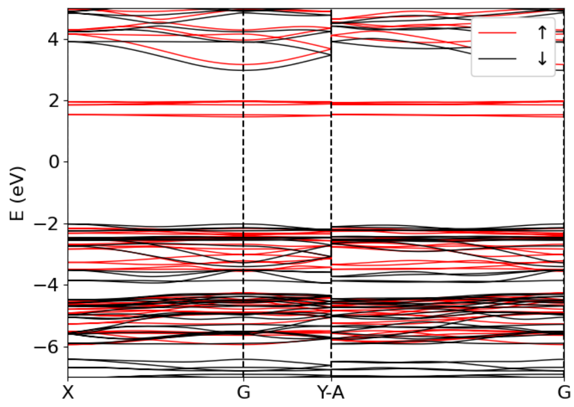


**Electronic bandstructure:** Spin-resolved energy bands of monolayer $CoO_8Cl_2$ along a high-symmetry path.

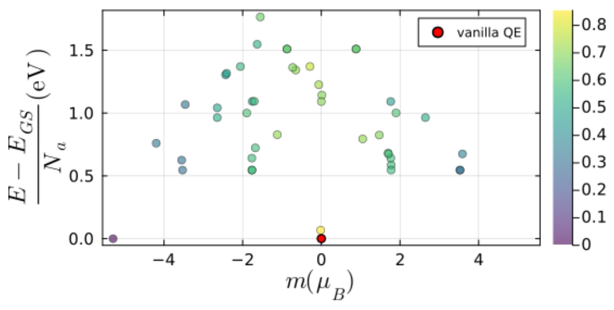


**Unique states:** Representation of 46 self-consistent unique states for monolayer $CoO_8Cl_2$ identified using RomeoDFT (see Section S6).

**Lattice vectors:** Cartesian components (in [Å]) of the lattice vectors for $CoO_8Cl_2$.

| | $x$ | $y$ | $z$ |
|---|---|---|---|
| $\boldsymbol{a_1}$ | 9.7480 | 0.0000 | 0.0000 |
| $\boldsymbol{a_2}$ | −2.4370 | 4.2210 | 0.0000 |
| $\boldsymbol{a_3}$ | 0.0000 | 0.0000 | 26.3498 |

**Atomic positions:** Fractional coordinates, Hubbard $U$ (in eV) and magnetic moments (in $\mu_B$, computed from orbital occupations $m_o$ or integration spheres $m_i$) of each atom of monolayer $CoO_8Cl_2$.

| atom | $x$ | $y$ | $z$ | $U$ | $m_o$ | $m_i$ |
|---|---|---|---|---|---|---|
| Co | 0.17 | 0.67 | 0.50 | 4.34 | −2.65 | −2.63 |
| Co | 0.67 | 0.67 | 0.50 | 4.34 | −2.65 | −2.63 |
| Cl | 0.33 | 0.33 | 0.57 | 0.0 | – | 0.00 |
| O | 0.20 | 0.36 | 0.55 | 0.0 | – | −0.02 |
| O | 0.48 | 0.61 | 0.55 | 0.0 | – | −0.02 |
| O | 0.32 | 0.04 | 0.55 | 0.0 | – | −0.02 |
| O | 0.33 | 0.33 | 0.62 | 0.0 | – | −0.01 |
| Cl | 0.0 | 1.00 | 0.43 | 0.0 | – | 0.00 |
| O | 0.14 | 0.98 | 0.45 | 0.0 | – | −0.02 |
| O | 0.35 | 0.73 | 0.45 | 0.0 | – | −0.02 |
| O | 0.01 | 0.30 | 0.45 | 0.0 | – | −0.02 |
| O | 0.0 | 1.00 | 0.38 | 0.0 | – | −0.01 |
| Cl | 0.83 | 0.33 | 0.57 | 0.0 | – | 0.00 |
| O | 0.70 | 0.36 | 0.55 | 0.0 | – | −0.02 |
| O | 0.98 | 0.61 | 0.55 | 0.0 | – | −0.02 |
| O | 0.82 | 0.04 | 0.55 | 0.0 | – | −0.02 |
| O | 0.83 | 0.33 | 0.62 | 0.0 | – | −0.01 |
| Cl | 0.50 | 1.00 | 0.43 | 0.0 | – | 0.00 |
| O | 0.64 | 0.98 | 0.45 | 0.0 | – | −0.02 |
| O | 0.85 | 0.73 | 0.45 | 0.0 | – | −0.02 |
| O | 0.51 | 0.30 | 0.45 | 0.0 | – | −0.02 |
| O | 0.50 | 1.00 | 0.38 | 0.0 | – | −0.01 |

# $CoTl_2F_4$ (AFM)

Band gap: 3.67 eV
Total magnetization: 0.0 $\mu_B/cell$
Absolute magnetization: 5.87 $\mu_B/cell$
MC2D entry: https://mc2d.materialscloud.org/#/details/mc2d-2675

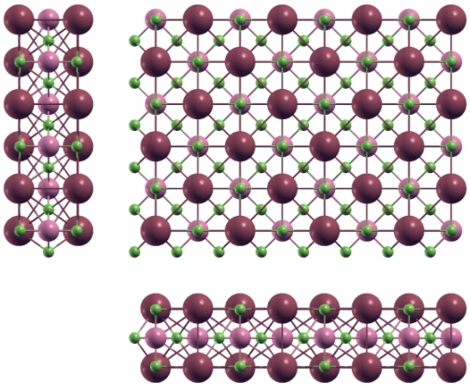

**Geometry:** Views of $CoTl_2F_4$ as seen from the $x$ axis (left), the $y$ axis (bottom), and the $z$ axis (center).

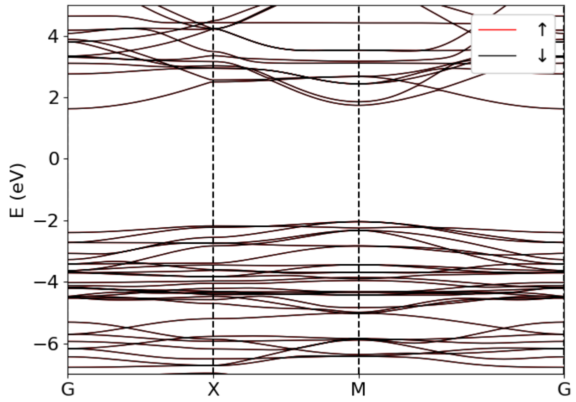


**Electronic bandstructure:** Spin-resolved energy bands of monolayer $CoTl_2F_4$ along a high-symmetry path.

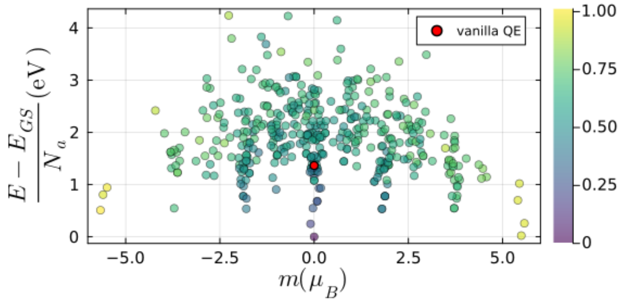


**Unique states:** Representation of 432 self-consistent unique states for monolayer $CoTl_2F_4$ identified using RomeoDFT (see Section S6).

**Lattice vectors:** Cartesian components (in [Å]) of the lattice vectors for $CoTl_2F_4$.

| | $x$ | $y$ | $z$ |
|---|---|---|---|
| $\boldsymbol{a_1}$ | −4.1621 | 4.1621 | 0.0000 |
| $\boldsymbol{a_2}$ | −4.1621 | −4.1621 | 0.0000 |
| $\boldsymbol{a_3}$ | 0.0000 | 0.0000 | 20.1474 |

**Atomic positions:** Fractional coordinates, Hubbard $U$ (in eV) and magnetic moments (in $\mu_B$, computed from orbital occupations $m_o$ or integration spheres $m_i$) of each atom of monolayer $CoTl_2F_4$.

| atom | $x$ | $y$ | $z$ | $U$ | $m_o$ | $m_i$ |
|---|---|---|---|---|---|---|
| Co | 0.25 | 0.75 | 0.0 | 7.11 | −2.74 | −2.64 |
| Co | 0.75 | 0.25 | 0.0 | 7.11 | 2.74 | 2.64 |
| Tl | 1.25 | 1.25 | 0.90 | 0.0 | – | 0.0 |
| Tl | 0.75 | 0.75 | 0.90 | 0.0 | – | 0.0 |
| Tl | 0.25 | 0.25 | 0.10 | 0.0 | – | 0.0 |
| Tl | 0.75 | 0.75 | 0.10 | 0.0 | – | 0.0 |
| F | 0.75 | 1.25 | 0.90 | 0.0 | – | 0.01 |
| F | 1.25 | 0.75 | 0.90 | 0.0 | – | −0.01 |
| F | 0.25 | 0.75 | 0.10 | 0.0 | – | −0.01 |
| F | 0.75 | 0.25 | 0.10 | 0.0 | – | 0.01 |
| F | 0.50 | 0.50 | 0.0 | 0.0 | – | 0.0 |
| F | 0.0 | 0.0 | 0.0 | 0.0 | – | 0.0 |
| F | 0.0 | 0.50 | 0.0 | 0.0 | – | 0.0 |
| F | 0.50 | 0.0 | 0.0 | 0.0 | – | 0.0 |

## $Cr_3O_8$ (FM)

Band gap: 0.0 eV
Total magnetization: $-3.97\ \mu_B/cell$
Absolute magnetization: $12.42\ \mu_B/cell$
MC2D entry: https://mc2d.materialscloud.org/#/details/mc2d-2618

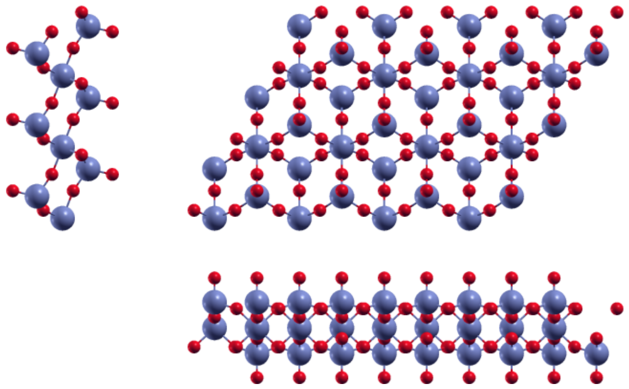

**Geometry:** Views of $Cr_3O_8$ as seen from the $x$ axis (left), the $y$ axis (bottom), and the $z$ axis (center).

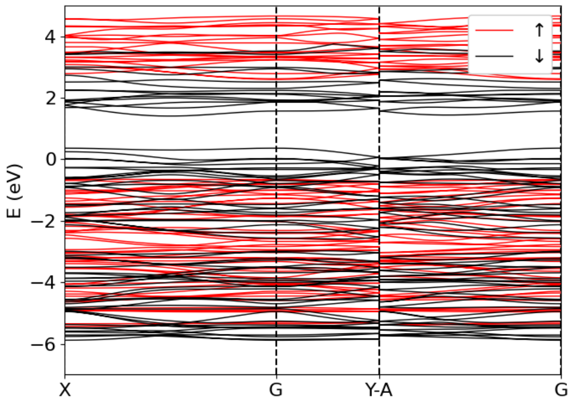


**Electronic bandstructure:** Spin-resolved energy bands of monolayer $Cr_3O_8$ along a high-symmetry path.

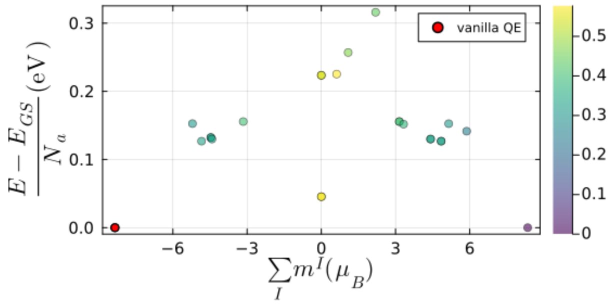


**Unique states:** Representation of 25 self-consistent unique states for monolayer $Cr_3O_8$ identified using RomeoDFT (see Section S6).

**Lattice vectors:** Cartesian components (in [Å]) of the lattice vectors for $Cr_3O_8$.

| | $x$ | $y$ | $z$ |
|---|---|---|---|
| $\boldsymbol{a_1}$ | 0.0000 | 10.7582 | 0.0000 |
| $\boldsymbol{a_2}$ | −4.5188 | 2.6895 | 0.0000 |
| $\boldsymbol{a_3}$ | 0.0000 | 0.0000 | 24.3250 |

**Atomic positions:** Fractional coordinates, Hubbard $U$ (in eV) and magnetic moments (in $\mu_B$, computed from orbital occupations $m_o$ or integration spheres $m_i$) of each atom of monolayer $Cr_3O_8$.

| atom | $x$ | $y$ | $z$ | $U$ | $m_o$ | $m_i$ |
|---|---|---|---|---|---|---|
| Cr | 0.08 | 0.69 | −0.07 | 6.18 | −0.81 | −0.53 |
| Cr | −0.08 | 0.31 | 0.07 | 6.18 | −0.81 | −0.53 |
| Cr | 0.25 | 0.0 | 0.0 | 6.18 | −2.56 | −1.76 |
| Cr | 0.58 | 0.69 | −0.07 | 6.18 | −0.81 | −0.53 |
| Cr | 0.42 | 0.31 | 0.07 | 6.18 | −0.81 | −0.53 |
| Cr | 0.75 | 0.0 | 0.0 | 6.18 | −2.56 | −1.76 |
| O | −0.10 | 0.89 | −0.05 | 0.0 | – | 0.22 |
| O | 0.16 | 0.89 | −0.05 | 0.0 | – | 0.22 |
| O | 0.15 | 0.39 | −0.03 | 0.0 | – | 0.20 |
| O | 0.10 | 0.61 | −0.13 | 0.0 | – | 0.18 |
| O | −0.16 | 0.11 | 0.05 | 0.0 | – | 0.22 |
| O | 0.10 | 0.11 | 0.05 | 0.0 | – | 0.22 |
| O | −0.15 | 0.61 | 0.03 | 0.0 | – | 0.20 |
| O | −0.10 | 0.39 | 0.13 | 0.0 | – | 0.18 |
| O | 0.40 | 0.89 | −0.05 | 0.0 | – | 0.22 |
| O | 0.66 | 0.89 | −0.05 | 0.0 | – | 0.22 |
| O | 0.65 | 0.39 | −0.03 | 0.0 | – | 0.20 |
| O | 0.60 | 0.61 | −0.13 | 0.0 | – | 0.18 |
| O | 0.34 | 0.11 | 0.05 | 0.0 | – | 0.22 |
| O | 0.60 | 0.11 | 0.05 | 0.0 | – | 0.22 |
| O | 0.35 | 0.61 | 0.03 | 0.0 | – | 0.20 |
| O | 0.40 | 0.39 | 0.13 | 0.0 | – | 0.18 |

## $Cr_3Te_4$ (AFM)

Band gap: 0.0 eV
Total magnetization: $-0.0\ \mu_B/cell$
Absolute magnetization: $28.14\ \mu_B/cell$
MC2D entry: https://mc2d.materialscloud.org/#/details/mc2d-1248

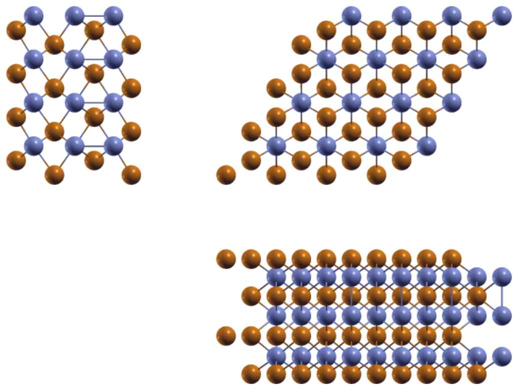

**Geometry:** Views of $Cr_3Te_4$ as seen from the $x$ axis (left), the $y$ axis (bottom), and the $z$ axis (center).

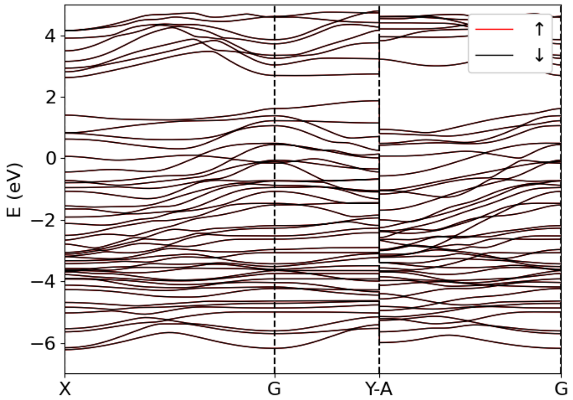


**Electronic bandstructure:** Spin-resolved energy bands of monolayer $Cr_3Te_4$ along a high-symmetry path.

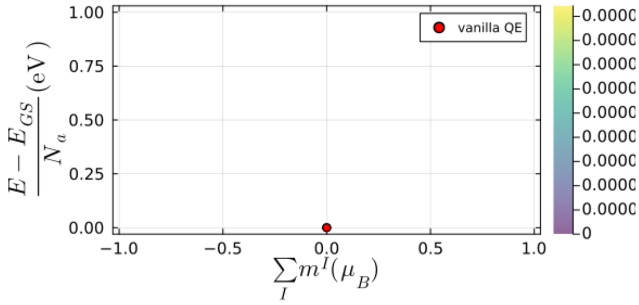


**Unique states:** Representation of 2 self-consistent unique states for monolayer $Cr_3Te_4$ identified using RomeoDFT (see Section S6).

**Lattice vectors:** Cartesian components (in [Å]) of the lattice vectors for $Cr_3Te_4$.

| | $x$ | $y$ | $z$ |
|---|---|---|---|
| $\boldsymbol{a_1}$ | 8.0289 | −0.0028 | 0.0000 |
| $\boldsymbol{a_2}$ | 2.0083 | 3.4760 | 0.0000 |
| $\boldsymbol{a_3}$ | 0.0000 | 0.0000 | 29.6009 |

**Atomic positions:** Fractional coordinates, Hubbard $U$ (in eV) and magnetic moments (in $\mu_B$, computed from orbital occupations $m_o$ or integration spheres $m_i$) of each atom of monolayer $Cr_3Te_4$.

| atom | $x$ | $y$ | $z$ | $U$ | $m_o$ | $m_i$ |
|---|---|---|---|---|---|---|
| Cr | 0.50 | 1.00 | 0.61 | 5.97 | 4.18 | 3.82 |
| Cr | 0.50 | 1.00 | 0.50 | 7.03 | 4.42 | 4.11 |
| Cr | 0.50 | 1.00 | 0.39 | 5.91 | 4.14 | 3.81 |
| Cr | 1.00 | 1.00 | 0.61 | 5.97 | −4.18 | −3.82 |
| Cr | 1.00 | 1.00 | 0.50 | 7.03 | −4.42 | −4.11 |
| Cr | 1.00 | 1.00 | 0.39 | 5.91 | −4.14 | −3.81 |
| Te | 0.17 | 0.33 | 0.55 | 0.0 | – | 0.10 |
| Te | 0.33 | 0.67 | 0.66 | 0.0 | – | −0.08 |
| Te | 0.33 | 0.67 | 0.45 | 0.0 | – | −0.09 |
| Te | 0.17 | 0.33 | 0.34 | 0.0 | – | 0.08 |
| Te | 0.67 | 0.33 | 0.55 | 0.0 | – | −0.10 |
| Te | 0.83 | 0.67 | 0.66 | 0.0 | – | 0.08 |
| Te | 0.83 | 0.67 | 0.45 | 0.0 | – | 0.09 |
| Te | 0.67 | 0.33 | 0.34 | 0.0 | – | −0.08 |

## $CrBr_2$ (AFM)

Band gap: 1.45 eV
Total magnetization: 0.0 $\mu_B/cell$
Absolute magnetization: 8.44 $\mu_B/cell$
MC2D entry: https://mc2d.materialscloud.org/#/details/mc2d-2703

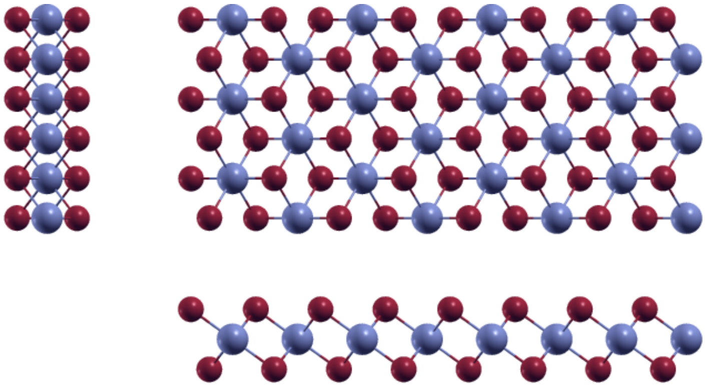

**Geometry:** Views of $CrBr_2$ as seen from the $x$ axis (left), the $y$ axis (bottom), and the $z$ axis (center).

**Lattice vectors:** Cartesian components (in [Å]) of the lattice vectors for $CrBr_2$.

| | $x$ | $y$ | $z$ |
|---|---|---|---|
| $\boldsymbol{a_1}$ | −0.0062 | −6.4469 | 0.0000 |
| $\boldsymbol{a_2}$ | 3.9405 | 0.0026 | 0.0000 |
| $\boldsymbol{a_3}$ | 0.0000 | 0.0000 | 23.0365 |

**Atomic positions:** Fractional coordinates, Hubbard $U$ (in eV) and magnetic moments (in $\mu_B$, computed from orbital occupations $m_o$ or integration spheres $m_i$) of each atom of monolayer $CrBr_2$.

| atom | $x$ | $y$ | $z$ | $U$ | $m_o$ | $m_i$ |
|---|---|---|---|---|---|---|
| Cr | 0.50 | 0.50 | 0.50 | 4.63 | −3.97 | −3.63 |
| Cr | 1.00 | 1.00 | 0.50 | 4.63 | 3.97 | 3.63 |
| Br | 0.82 | 0.51 | 0.56 | 0.0 | – | 0.02 |
| Br | 0.32 | 1.01 | 0.56 | 0.0 | – | −0.02 |
| Br | 0.68 | 1.00 | 0.44 | 0.0 | – | −0.02 |
| Br | 1.18 | 0.50 | 0.44 | 0.0 | – | 0.02 |

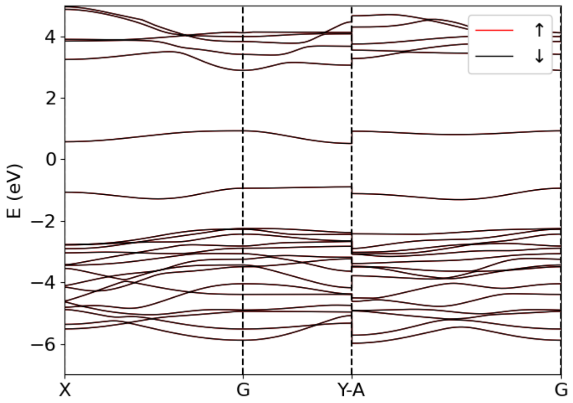


**Electronic bandstructure:** Spin-resolved energy bands of monolayer $CrBr_2$ along a high-symmetry path.

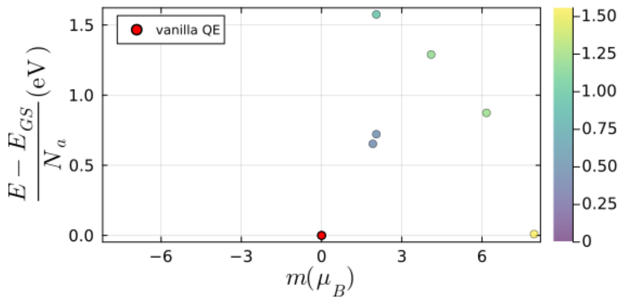


**Unique states:** Representation of 8 self-consistent unique states for monolayer $CrBr_2$ identified using RomeoDFT (see Section S6).

## $CrBr_3$ (FM)

Band gap: 1.62 eV
Total magnetization: −6.0 $\mu_B/cell$
Absolute magnetization: 8.57 $\mu_B/cell$
MC2D entry: https://mc2d.materialscloud.org/#/details/mc2d-608

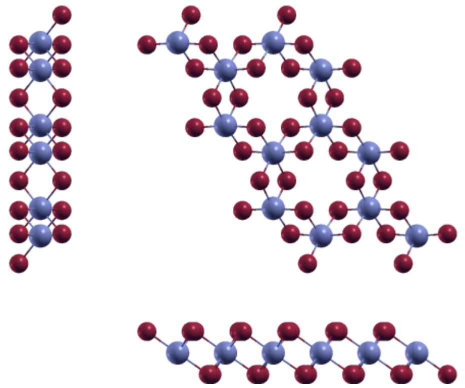

**Geometry:** Views of $CrBr_3$ as seen from the $x$ axis (left), the $y$ axis (bottom), and the $z$ axis (center).

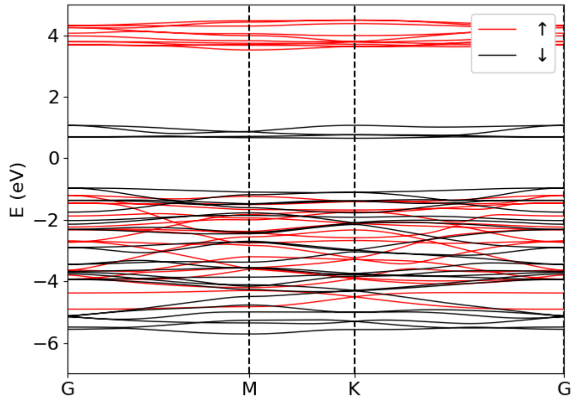


**Electronic bandstructure:** Spin-resolved energy bands of monolayer $CrBr_3$ along a high-symmetry path.

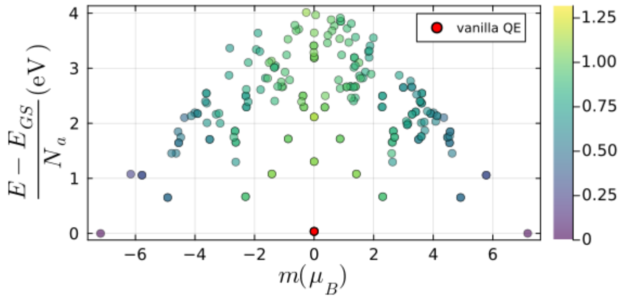


**Unique states:** Representation of 203 self-consistent unique states for monolayer $CrBr_3$ identified using RomeoDFT (see Section S6).

**Lattice vectors:** Cartesian components (in [Å]) of the lattice vectors for $CrBr_3$.

| | $x$ | $y$ | $z$ |
|---|---|---|---|
| $\boldsymbol{a_1}$ | 6.4168 | 0.0000 | 0.0000 |
| $\boldsymbol{a_2}$ | −3.2084 | 5.5571 | 0.0000 |
| $\boldsymbol{a_3}$ | 0.0000 | 0.0000 | 22.8672 |

**Atomic positions:** Fractional coordinates, Hubbard $U$ (in eV) and magnetic moments (in $\mu_B$, computed from orbital occupations $m_o$ or integration spheres $m_i$) of each atom of monolayer $CrBr_3$.

| atom | $x$ | $y$ | $z$ | $U$ | $m_o$ | $m_i$ |
|---|---|---|---|---|---|---|
| Cr | 0.67 | 0.33 | 0.50 | 5.11 | −3.58 | −3.26 |
| Cr | 0.33 | 0.67 | 0.50 | 5.11 | −3.58 | −3.26 |
| Br | 0.00 | 0.36 | 0.56 | 0.0 | – | 0.15 |
| Br | 0.64 | 0.64 | 0.56 | 0.0 | – | 0.15 |
| Br | 0.36 | 1.00 | 0.56 | 0.0 | – | 0.15 |
| Br | 0.64 | 0.00 | 0.44 | 0.0 | – | 0.15 |
| Br | 1.00 | 0.64 | 0.44 | 0.0 | – | 0.15 |
| Br | 0.36 | 0.36 | 0.44 | 0.0 | – | 0.15 |

## $CrCl_3$ (C2) (FM)

Band gap: 2.34 eV
Total magnetization: $-6.0\ \mu_B/cell$
Absolute magnetization: $7.25\ \mu_B/cell$
MC2D entry: https://mc2d.materialscloud.org/#/details/mc2d-1329

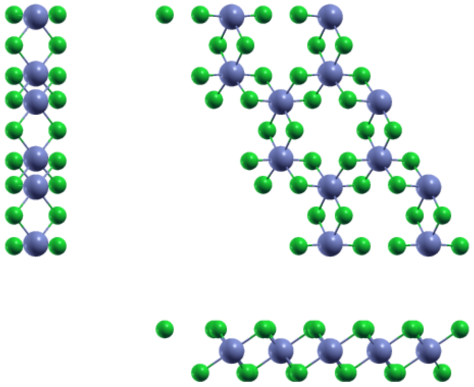

**Geometry:** Views of $CrCl_3$ (C2) as seen from the $x$ axis (left), the $y$ axis (bottom), and the $z$ axis (center).

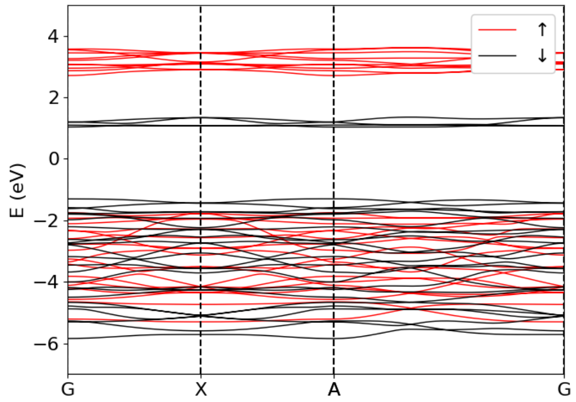


**Electronic bandstructure:** Spin-resolved energy bands of monolayer $CrCl_3$ (C2) along a high-symmetry path.

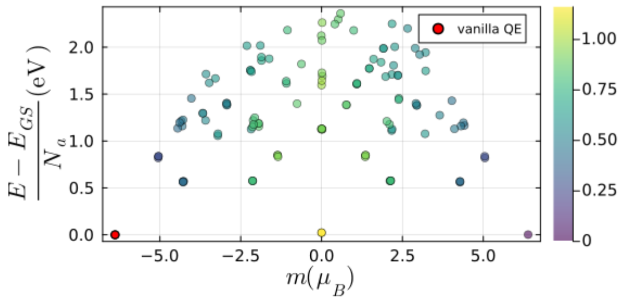


**Unique states:** Representation of 124 self-consistent unique states for monolayer $CrCl_3$ (C2) identified using RomeoDFT (see Section S6).

**Lattice vectors:** Cartesian components (in [Å]) of the lattice vectors for $CrCl_3$ (C2).

| | $x$ | $y$ | $z$ |
|---|---|---|---|
| $\boldsymbol{a_1}$ | 6.0559 | 0.0000 | 0.0000 |
| $\boldsymbol{a_2}$ | −3.0280 | 5.2334 | 0.0000 |
| $\boldsymbol{a_3}$ | 0.0000 | 0.0000 | 23.0229 |

**Atomic positions:** Fractional coordinates, Hubbard $U$ (in eV) and magnetic moments (in $\mu_B$, computed from orbital occupations $m_o$ or integration spheres $m_i$) of each atom of monolayer $CrCl_3$ (C2).

| atom | $x$ | $y$ | $z$ | $U$ | $m_o$ | $m_i$ |
|---|---|---|---|---|---|---|
| Cr | 0.56 | 0.11 | 0.50 | 3.37 | −3.19 | −2.88 |
| Cr | 0.89 | 0.78 | 0.50 | 3.37 | −3.19 | −2.88 |
| Cl | 0.22 | 0.09 | 0.56 | 0.0 | – | 0.07 |
| Cl | 0.86 | 0.09 | 0.44 | 0.0 | – | 0.07 |
| Cl | 0.86 | 0.45 | 0.56 | 0.0 | – | 0.07 |
| Cl | 0.58 | 0.45 | 0.44 | 0.0 | – | 0.07 |
| Cl | 0.58 | 0.81 | 0.56 | 0.0 | – | 0.07 |
| Cl | 0.22 | 0.81 | 0.44 | 0.0 | – | 0.07 |

# $CrCl_3$ (Cm) (FM)

Band gap: 2.36 eV
Total magnetization: 6.0 $\mu_B/cell$
Absolute magnetization: 7.62 $\mu_B/cell$
MC2D entry: https://mc2d.materialscloud.org/#/details/mc2d-352

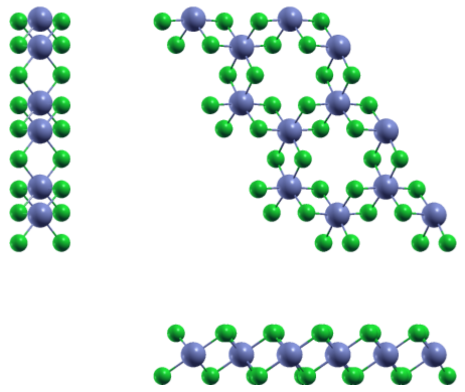

**Geometry:** Views of $CrCl_3$ (Cm) as seen from the $x$ axis (left), the $y$ axis (bottom), and the $z$ axis (center).

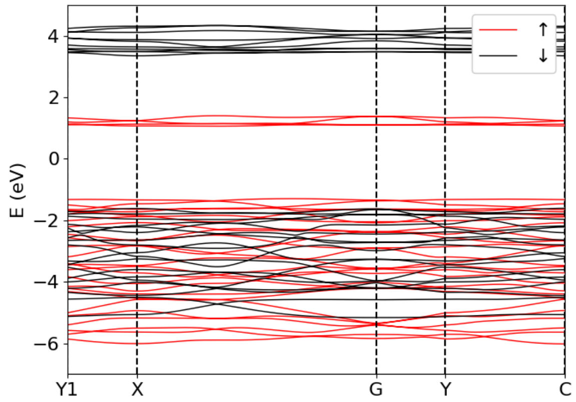


**Electronic bandstructure:** Spin-resolved energy bands of monolayer $CrCl_3$ (Cm) along a high-symmetry path.

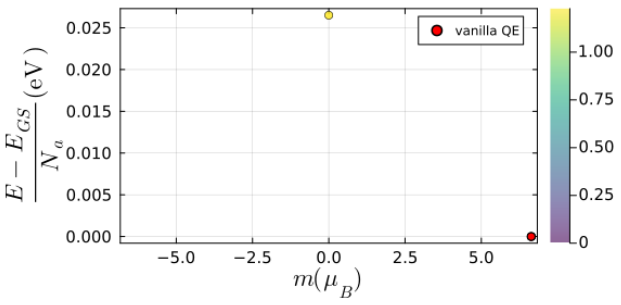


**Unique states:** Representation of 4 self-consistent unique states for monolayer $CrCl_3$ (Cm) identified using RomeoDFT (see Section S6).

**Lattice vectors:** Cartesian components (in [Å]) of the lattice vectors for $CrCl_3$ (Cm).

| | $x$ | $y$ | $z$ |
|---|---|---|---|
| $\boldsymbol{a_1}$ | 6.0329 | −0.1682 | 0.0000 |
| $\boldsymbol{a_2}$ | −2.8438 | 5.3233 | 0.0000 |
| $\boldsymbol{a_3}$ | 0.0000 | 0.0000 | 23.1040 |

**Atomic positions:** Fractional coordinates, Hubbard $U$ (in eV) and magnetic moments (in $\mu_B$, computed from orbital occupations $m_o$ or integration spheres $m_i$) of each atom of monolayer $CrCl_3$ (Cm).

| atom | $x$ | $y$ | $z$ | $U$ | $m_o$ | $m_i$ |
|---|---|---|---|---|---|---|
| Cr | 0.85 | 0.52 | 0.50 | 4.83 | 3.32 | 2.97 |
| Cr | 0.52 | 0.85 | 0.50 | 4.83 | 3.32 | 2.97 |
| Cl | 0.55 | 0.55 | 0.56 | 0.0 | – | −0.10 |
| Cl | 0.19 | 0.83 | 0.56 | 0.0 | – | −0.10 |
| Cl | 0.83 | 0.19 | 0.56 | 0.0 | – | −0.10 |
| Cl | 0.19 | 0.55 | 0.44 | 0.0 | – | −0.10 |
| Cl | 0.55 | 0.19 | 0.44 | 0.0 | – | −0.10 |
| Cl | 0.83 | 0.83 | 0.44 | 0.0 | – | −0.10 |

## $CrGeTe_3$ (FM)

Band gap: 0.17 eV
Total magnetization: $-5.93\ \mu_B/cell$
Absolute magnetization: $10.85\ \mu_B/cell$
MC2D entry: https://mc2d.materialscloud.org/#/details/mc2d-548

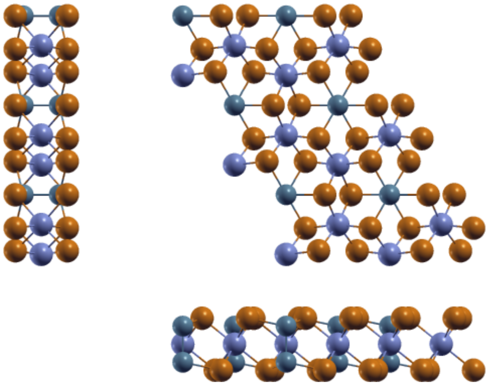

**Geometry:** Views of $CrGeTe_3$ as seen from the $x$ axis (left), the $y$ axis (bottom), and the $z$ axis (center).

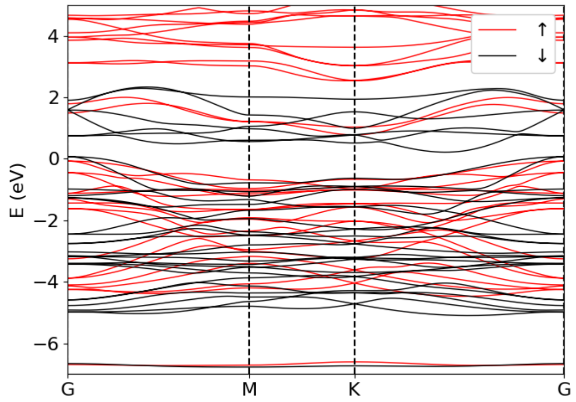


**Electronic bandstructure:** Spin-resolved energy bands of monolayer $CrGeTe_3$ along a high-symmetry path.

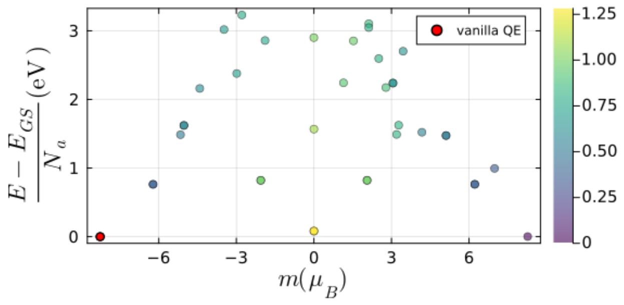


**Unique states:** Representation of 38 self-consistent unique states for monolayer $CrGeTe_3$ identified using RomeoDFT (see Section S6).

**Lattice vectors:** Cartesian components (in [Å]) of the lattice vectors for $CrGeTe_3$.

| | $x$ | $y$ | $z$ |
|---|---|---|---|
| $\boldsymbol{a_1}$ | 6.9016 | 0.0000 | 0.0000 |
| $\boldsymbol{a_2}$ | −3.4508 | 5.9770 | 0.0000 |
| $\boldsymbol{a_3}$ | 0.0000 | 0.0000 | 23.3436 |

**Atomic positions:** Fractional coordinates, Hubbard $U$ (in eV) and magnetic moments (in $\mu_B$, computed from orbital occupations $m_o$ or integration spheres $m_i$) of each atom of monolayer $CrGeTe_3$.

| atom | $x$ | $y$ | $z$ | $U$ | $m_o$ | $m_i$ |
|---|---|---|---|---|---|---|
| Cr | 0.67 | 0.33 | 0.50 | 5.88 | −4.13 | −3.83 |
| Cr | 0.0 | 0.0 | 0.50 | 5.88 | −4.13 | −3.83 |
| Ge | 0.33 | 0.67 | 0.45 | 0.0 | – | −0.01 |
| Te | 0.96 | 0.67 | 0.43 | 0.0 | – | 0.21 |
| Te | 0.33 | 0.29 | 0.43 | 0.0 | – | 0.21 |
| Te | 0.71 | 0.04 | 0.43 | 0.0 | – | 0.21 |
| Te | 0.71 | 0.67 | 0.57 | 0.0 | – | 0.21 |
| Te | 0.33 | 0.04 | 0.57 | 0.0 | – | 0.21 |
| Te | 0.96 | 0.29 | 0.57 | 0.0 | – | 0.21 |
| Ge | 0.33 | 0.67 | 0.55 | 0.0 | – | −0.01 |

## $CrI_2$ (AFM)

Band gap: 1.04 eV
Total magnetization: −0.0 $\mu_B/cell$
Absolute magnetization: 8.87 $\mu_B/cell$
MC2D entry: https://mc2d.materialscloud.org/#/details/mc2d-2716

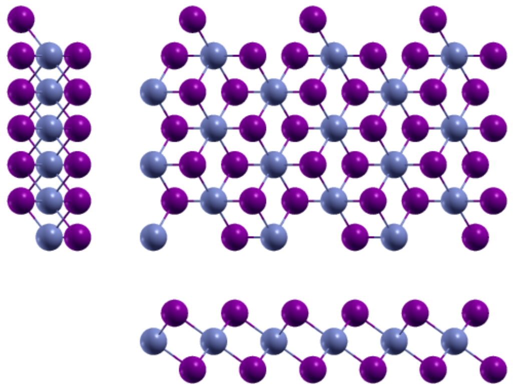

**Geometry:** Views of $CrI_2$ as seen from the $x$ axis (left), the $y$ axis (bottom), and the $z$ axis (center).

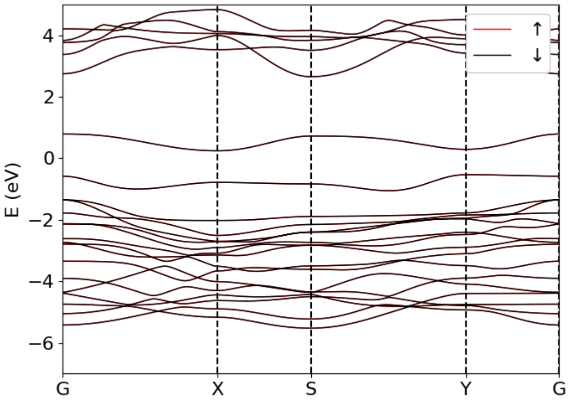


**Electronic bandstructure:** Spin-resolved energy bands of monolayer $CrI_2$ along a high-symmetry path.

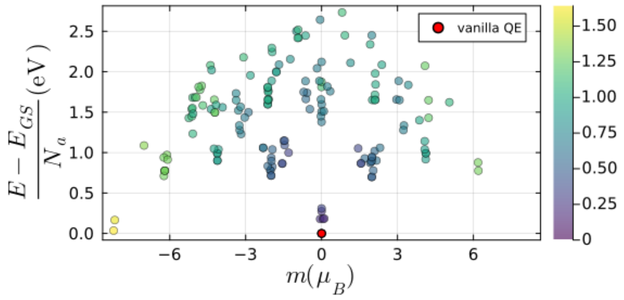


**Unique states:** Representation of 158 self-consistent unique states for monolayer $CrI_2$ identified using RomeoDFT (see Section S6).

**Lattice vectors:** Cartesian components (in [Å]) of the lattice vectors for $CrI_2$.

| | $x$ | $y$ | $z$ |
|---|---|---|---|
| $\boldsymbol{a_1}$ | 0.0000 | −6.9257 | 0.0000 |
| $\boldsymbol{a_2}$ | 4.1821 | 0.0000 | 0.0000 |
| $\boldsymbol{a_3}$ | 0.0000 | 0.0000 | 18.2099 |

**Atomic positions:** Fractional coordinates, Hubbard $U$ (in eV) and magnetic moments (in $\mu_B$, computed from orbital occupations $m_o$ or integration spheres $m_i$) of each atom of monolayer $CrI_2$.

| atom | $x$ | $y$ | $z$ | $U$ | $m_o$ | $m_i$ |
|---|---|---|---|---|---|---|
| Cr | 0.50 | 0.50 | 0.0 | 5.42 | −4.10 | −3.84 |
| Cr | 0.0 | 0.0 | 0.0 | 5.42 | 4.10 | 3.84 |
| I | 1.18 | 0.50 | 0.91 | 0.0 | – | 0.02 |
| I | 0.68 | 1.00 | 0.91 | 0.0 | – | −0.02 |
| I | 0.82 | 0.50 | 0.09 | 0.0 | – | 0.02 |
| I | 0.32 | 1.00 | 0.09 | 0.0 | – | −0.02 |

## $CrI_3$ (FM)

Band gap: 0.90 eV
Total magnetization: 5.76 $\mu_B/cell$
Absolute magnetization: 10.21 $\mu_B/cell$
MC2D entry: https://mc2d.materialscloud.org/#/details/mc2d-2551

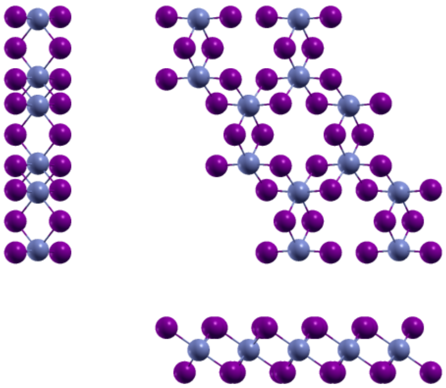

**Geometry:** Views of $CrI_3$ as seen from the $x$ axis (left), the $y$ axis (bottom), and the $z$ axis (center).

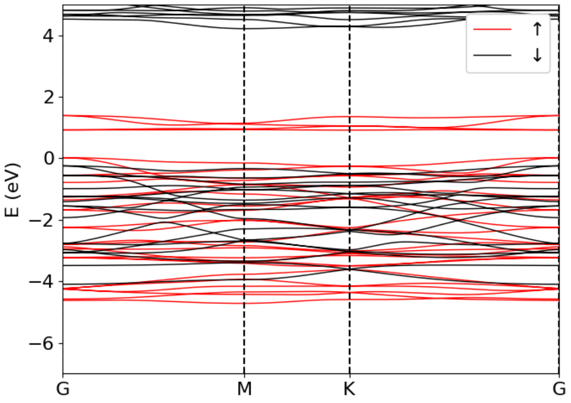


**Electronic bandstructure:** Spin-resolved energy bands of monolayer $CrI_3$ along a high-symmetry path.

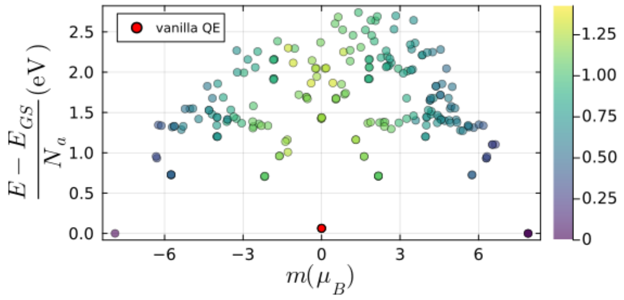


**Unique states:** Representation of 211 self-consistent unique states for monolayer $CrI_3$ identified using RomeoDFT (see Section S6).

**Lattice vectors:** Cartesian components (in [Å]) of the lattice vectors for $CrI_3$.

| | $x$ | $y$ | $z$ |
|---|---|---|---|
| $\boldsymbol{a_1}$ | −3.5063 | −6.0731 | 0.0000 |
| $\boldsymbol{a_2}$ | 7.0126 | 0.0000 | 0.0000 |
| $\boldsymbol{a_3}$ | 0.0000 | 0.0000 | 18.1019 |

**Atomic positions:** Fractional coordinates, Hubbard $U$ (in eV) and magnetic moments (in $\mu_B$, computed from orbital occupations $m_o$ or integration spheres $m_i$) of each atom of monolayer $CrI_3$.

| atom | $x$ | $y$ | $z$ | $U$ | $m_o$ | $m_i$ |
|---|---|---|---|---|---|---|
| Cr | 0.33 | 0.17 | −0.50 | 5.52 | 3.95 | 3.66 |
| Cr | −0.33 | −0.17 | −0.50 | 5.52 | 3.95 | 3.66 |
| I | 0.64 | 0.14 | −0.41 | 0.0 | – | −0.24 |
| I | 0.36 | 0.50 | −0.41 | 0.0 | – | −0.24 |
| I | 0.00 | −0.14 | −0.41 | 0.0 | – | −0.24 |
| I | 0.36 | −0.14 | −0.59 | 0.0 | – | −0.24 |
| I | 0.64 | 0.50 | −0.59 | 0.0 | – | −0.24 |
| I | 0.00 | 0.14 | −0.59 | 0.0 | – | −0.24 |

# $CrO_2$ (FM)

Band gap: 0.82 eV
Total magnetization: 4.01 $\mu_B/cell$
Absolute magnetization: 5.4 $\mu_B/cell$
MC2D entry: https://mc2d.materialscloud.org/#/details/mc2d-2083

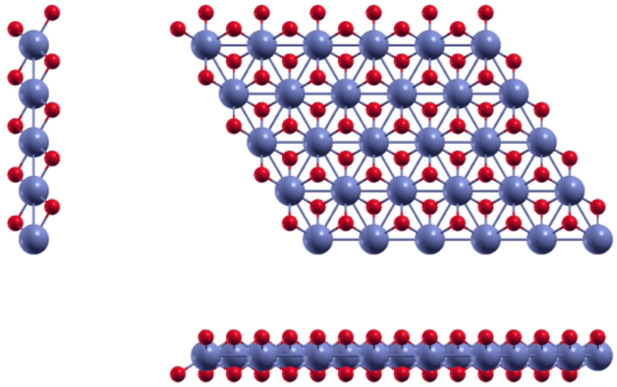

**Geometry:** Views of $CrO_2$ as seen from the $x$ axis (left), the $y$ axis (bottom), and the $z$ axis (center).

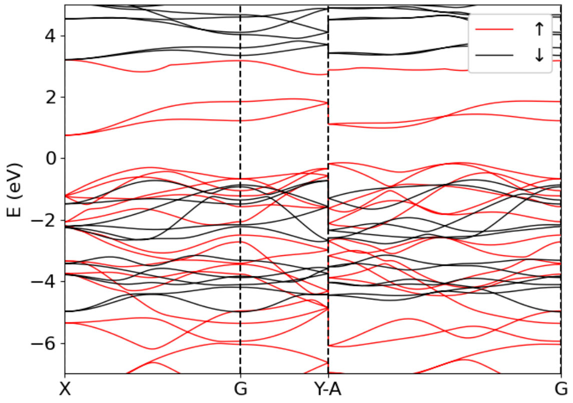


**Electronic bandstructure:** Spin-resolved energy bands of monolayer $CrO_2$ along a high-symmetry path.

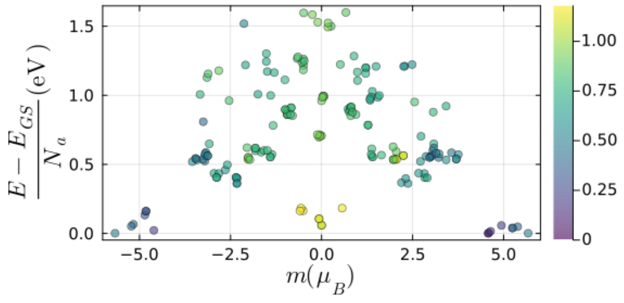


**Unique states:** Representation of 183 self-consistent unique states for monolayer $CrO_2$ identified using RomeoDFT (see Section S6).

**Lattice vectors:** Cartesian components (in [Å]) of the lattice vectors for $CrO_2$.

| | $x$ | $y$ | $z$ |
|---|---|---|---|
| $\boldsymbol{a_1}$ | 2.8910 | −5.0074 | 0.0000 |
| $\boldsymbol{a_2}$ | 1.4455 | 2.5037 | 0.0000 |
| $\boldsymbol{a_3}$ | 0.0000 | 0.0000 | 16.2658 |

**Atomic positions:** Fractional coordinates, Hubbard $U$ (in eV) and magnetic moments (in $\mu_B$, computed from orbital occupations $m_o$ or integration spheres $m_i$) of each atom of monolayer $CrO_2$.

| atom | $x$ | $y$ | $z$ | $U$ | $m_o$ | $m_i$ |
|---|---|---|---|---|---|---|
| Cr | 0.25 | 0.0 | 0.0 | 6.22 | 2.29 | 1.95 |
| Cr | 0.75 | 0.0 | 0.0 | 6.22 | 2.29 | 1.95 |
| O | 0.08 | 0.33 | 0.06 | 0.0 | – | −0.16 |
| O | −0.08 | −0.33 | −0.06 | 0.0 | – | −0.16 |
| O | 0.58 | 0.33 | 0.06 | 0.0 | – | −0.16 |
| O | 0.42 | −0.33 | −0.06 | 0.0 | – | −0.16 |

## CrOBr (FM)

Band gap: 1.20 eV
Total magnetization: $-6.0\ \mu_B/cell$
Absolute magnetization: $7.12\ \mu_B/cell$
MC2D entry: https://mc2d.materialscloud.org/#/details/mc2d-44

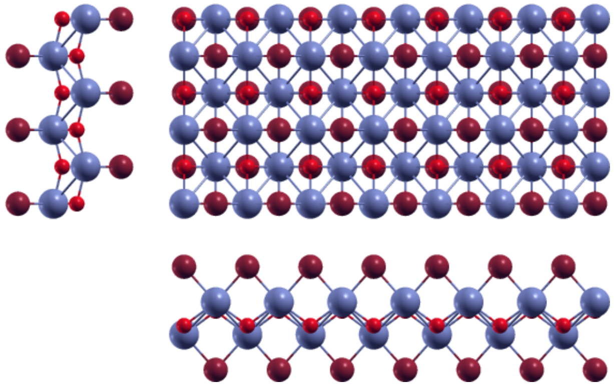

**Geometry:** Views of CrOBr as seen from the $x$ axis (left), the $y$ axis (bottom), and the $z$ axis (center).

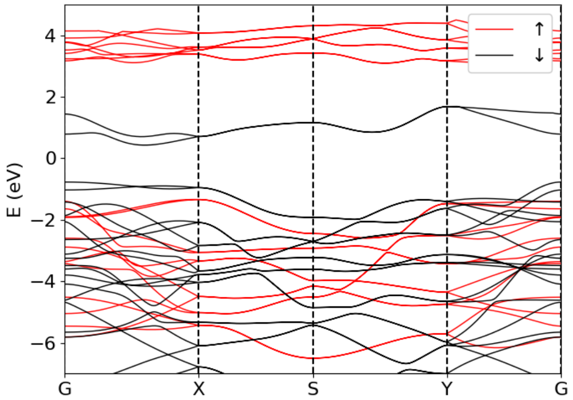


**Electronic bandstructure:** Spin-resolved energy bands of monolayer CrOBr along a high-symmetry path.

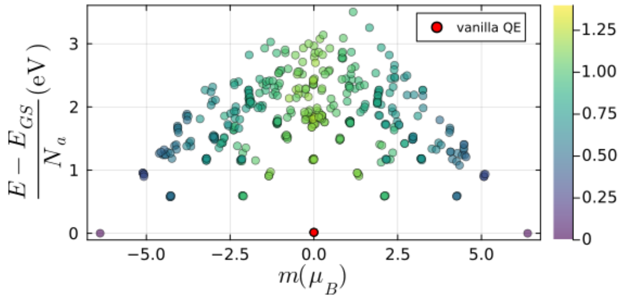


**Unique states:** Representation of 392 self-consistent unique states for monolayer CrOBr identified using RomeoDFT (see Section S6).

**Lattice vectors:** Cartesian components (in [Å]) of the lattice vectors for CrOBr.

| | $x$ | $y$ | $z$ |
|---|---|---|---|
| $\boldsymbol{a_1}$ | 3.3096 | 0.0000 | 0.0000 |
| $\boldsymbol{a_2}$ | 0.0000 | 3.8688 | 0.0000 |
| $\boldsymbol{a_3}$ | 0.0000 | 0.0000 | 24.9530 |

**Atomic positions:** Fractional coordinates, Hubbard $U$ (in eV) and magnetic moments (in $\mu_B$, computed from orbital occupations $m_o$ or integration spheres $m_i$) of each atom of monolayer CrOBr.

| atom | $x$ | $y$ | $z$ | $U$ | $m_o$ | $m_i$ |
|---|---|---|---|---|---|---|
| Cr | 0.50 | 0.50 | 0.47 | 4.93 | −3.19 | −2.65 |
| Cr | 0.0 | 0.0 | 0.53 | 4.93 | −3.19 | −2.65 |
| Br | 0.0 | 0.50 | 0.39 | 0.0 | – | 0.09 |
| O | 0.50 | 0.0 | 0.48 | 0.0 | – | 0.13 |
| Br | 0.50 | 0.0 | 0.61 | 0.0 | – | 0.09 |
| O | 0.0 | 0.50 | 0.52 | 0.0 | – | 0.13 |

## CrOCl (FM)

Band gap: 1.66 eV
Total magnetization: −6.0 $\mu_B/cell$
Absolute magnetization: 6.85 $\mu_B/cell$
MC2D entry: https://mc2d.materialscloud.org/#/details/mc2d-104

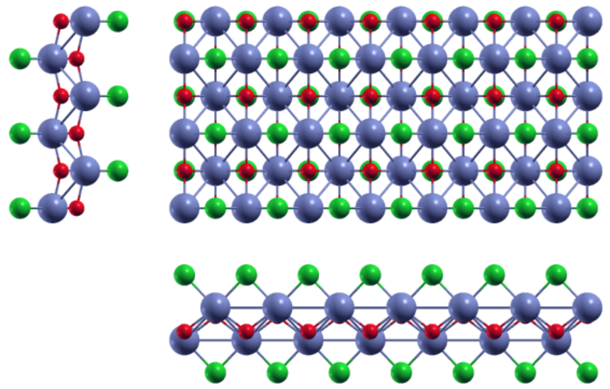

**Geometry:** Views of CrOCl as seen from the $x$ axis (left), the $y$ axis (bottom), and the $z$ axis (center).

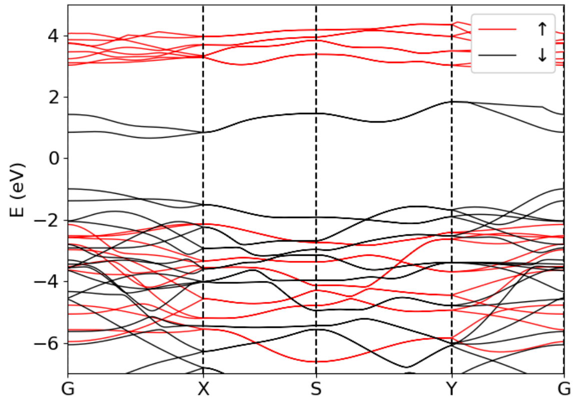


**Electronic bandstructure:** Spin-resolved energy bands of monolayer CrOCl along a high-symmetry path.

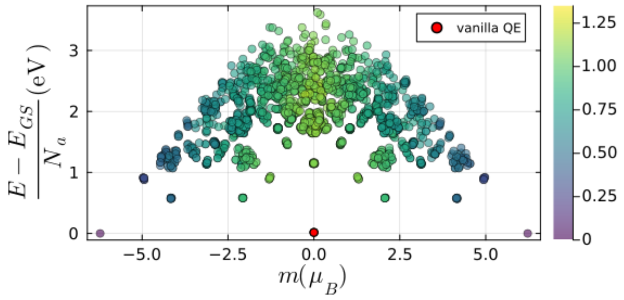


**Unique states:** Representation of 1883 self-consistent unique states for monolayer CrOCl identified using RomeoDFT (see Section S6).

**Lattice vectors:** Cartesian components (in [Å]) of the lattice vectors for CrOCl.

| | $x$ | $y$ | $z$ |
|---|---|---|---|
| $\boldsymbol{a_1}$ | 3.2167 | 0.0000 | 0.0000 |
| $\boldsymbol{a_2}$ | 0.0000 | 3.8773 | 0.0000 |
| $\boldsymbol{a_3}$ | 0.0000 | 0.0000 | 24.8200 |

**Atomic positions:** Fractional coordinates, Hubbard $U$ (in eV) and magnetic moments (in $\mu_B$, computed from orbital occupations $m_o$ or integration spheres $m_i$) of each atom of monolayer CrOCl.

| atom | $x$ | $y$ | $z$ | $U$ | $m_o$ | $m_i$ |
|---|---|---|---|---|---|---|
| Cr | 0.0 | 0.0 | 0.53 | 4.69 | −3.11 | −2.60 |
| Cr | 0.50 | 0.50 | 0.47 | 4.69 | −3.11 | −2.60 |
| Cl | 0.50 | 0.0 | 0.60 | 0.0 | – | 0.06 |
| Cl | 0.0 | 0.50 | 0.40 | 0.0 | – | 0.06 |
| O | 0.50 | 0.0 | 0.48 | 0.0 | – | 0.11 |
| O | 0.0 | 0.50 | 0.52 | 0.0 | – | 0.11 |

# $CrPS_4$ (FM)

Band gap: 0.94 eV
Total magnetization: 6.0 $\mu_B/cell$
Absolute magnetization: 8.65 $\mu_B/cell$
MC2D entry: https://mc2d.materialscloud.org/#/details/mc2d-1349

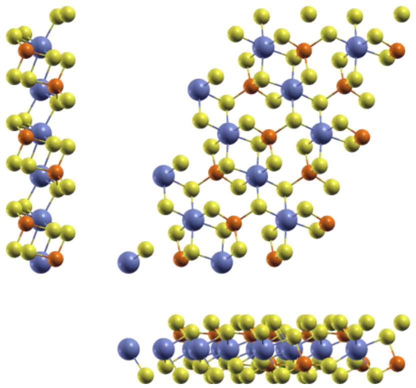

**Geometry:** Views of $CrPS_4$ as seen from the $x$ axis (left), the $y$ axis (bottom), and the $z$ axis (center).

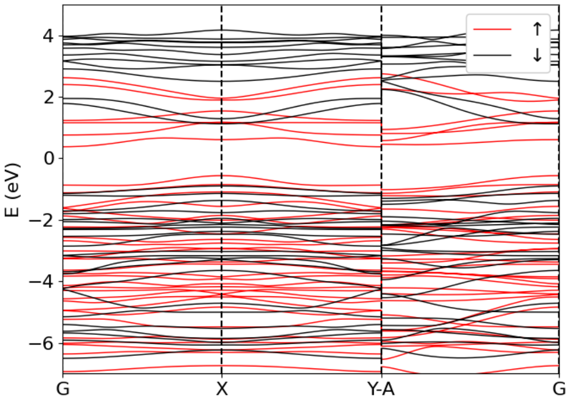


**Electronic bandstructure:** Spin-resolved energy bands of monolayer $CrPS_4$ along a high-symmetry path.

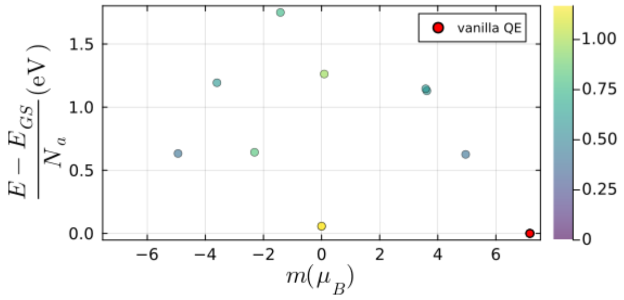


**Unique states:** Representation of 12 self-consistent unique states for monolayer $CrPS_4$ identified using RomeoDFT (see Section S6).

**Lattice vectors:** Cartesian components (in [Å]) of the lattice vectors for $CrPS_4$.

| | $x$ | $y$ | $z$ |
|---|---|---|---|
| $\boldsymbol{a_1}$ | 6.5456 | −0.4542 | 0.0000 |
| $\boldsymbol{a_2}$ | 2.8834 | 5.8938 | 0.0000 |
| $\boldsymbol{a_3}$ | 0.0000 | 0.0000 | 23.5741 |

**Atomic positions:** Fractional coordinates, Hubbard $U$ (in eV) and magnetic moments (in $\mu_B$, computed from orbital occupations $m_o$ or integration spheres $m_i$) of each atom of monolayer $CrPS_4$.

| atom | $x$ | $y$ | $z$ | $U$ | $m_o$ | $m_i$ |
|---|---|---|---|---|---|---|
| Cr | 0.53 | 0.51 | 0.50 | 5.29 | 3.59 | 3.17 |
| Cr | 0.02 | 1.02 | 0.50 | 5.29 | 3.59 | 3.17 |
| P | 0.98 | 0.47 | 0.54 | 0.0 | – | 0.02 |
| S | 0.65 | 0.14 | 0.54 | 0.0 | – | −0.15 |
| S | 0.68 | 0.63 | 0.58 | 0.0 | – | −0.06 |
| S | 0.18 | 0.66 | 0.55 | 0.0 | – | −0.09 |
| P | 0.57 | 0.06 | 0.46 | 0.0 | – | 0.02 |
| S | 0.87 | 0.89 | 0.42 | 0.0 | – | −0.06 |
| S | 0.90 | 0.39 | 0.46 | 0.0 | – | −0.15 |
| S | 0.41 | 0.36 | 0.42 | 0.0 | – | −0.06 |
| S | 0.38 | 0.86 | 0.45 | 0.0 | – | −0.09 |
| S | 0.15 | 0.17 | 0.58 | 0.0 | – | −0.06 |

## $CrPSe_3$ (FM)

Band gap: 0.0 eV
Total magnetization: $-6.75\ \mu_B/cell$
Absolute magnetization: $9.61\ \mu_B/cell$
MC2D entry: https://mc2d.materialscloud.org/#/details/mc2d-1232

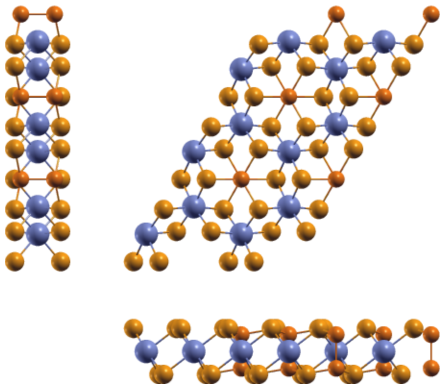

**Geometry:** Views of $CrPSe_3$ as seen from the $x$ axis (left), the $y$ axis (bottom), and the $z$ axis (center).

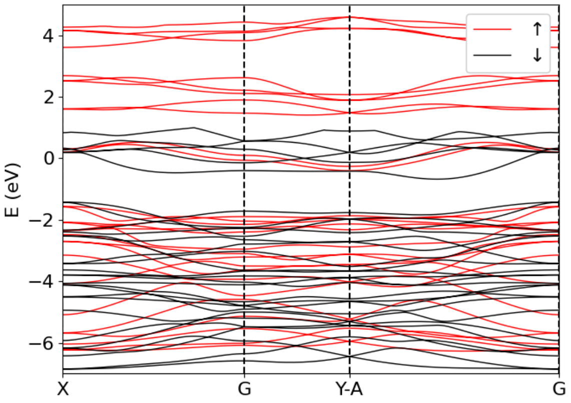


**Electronic bandstructure:** Spin-resolved energy bands of monolayer $CrPSe_3$ along a high-symmetry path.

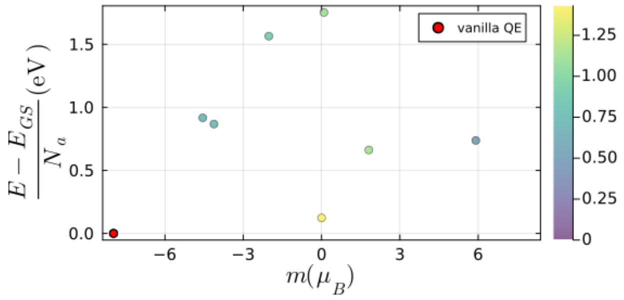


**Unique states:** Representation of 9 self-consistent unique states for monolayer $CrPSe_3$ identified using RomeoDFT (see Section S6).

**Lattice vectors:** Cartesian components (in [Å]) of the lattice vectors for $CrPSe_3$.

| | $x$ | $y$ | $z$ |
|---|---|---|---|
| $a_1$ | 6.3499 | 0.0000 | 0.0000 |
| $a_2$ | 3.1750 | 5.5113 | 0.0000 |
| $a_3$ | 0.0000 | 0.0000 | 23.1919 |

**Atomic positions:** Fractional coordinates, Hubbard $U$ (in eV) and magnetic moments (in $\mu_B$, computed from orbital occupations $m_o$ or integration spheres $m_i$) of each atom of monolayer $CrPSe_3$.

| atom | $x$ | $y$ | $z$ | $U$ | $m_o$ | $m_i$ |
|---|---|---|---|---|---|---|
| Cr | 0.36 | 0.67 | 0.50 | 6.14 | −3.99 | −3.59 |
| Cr | 0.03 | 0.33 | 0.50 | 6.14 | −3.99 | −3.59 |
| P | 0.70 | 0.00 | 0.45 | 0.0 | – | −0.03 |
| Se | 0.06 | 0.0 | 0.43 | 0.0 | – | 0.14 |
| Se | 0.70 | 0.63 | 0.43 | 0.0 | – | 0.14 |
| Se | 0.33 | 0.37 | 0.43 | 0.0 | – | 0.14 |
| Se | 0.33 | 0.0 | 0.57 | 0.0 | – | 0.14 |
| Se | 0.70 | 0.37 | 0.57 | 0.0 | – | 0.14 |
| Se | 0.06 | 0.63 | 0.57 | 0.0 | – | 0.14 |
| P | 0.70 | 0.00 | 0.55 | 0.0 | – | −0.03 |

## $CrS_2$ (AFM)

Band gap: 0.0 eV
Total magnetization: 0.0 $\mu_B/cell$
Absolute magnetization: 7.05 $\mu_B/cell$
MC2D entry: https://mc2d.materialscloud.org/#/details/mc2d-1123

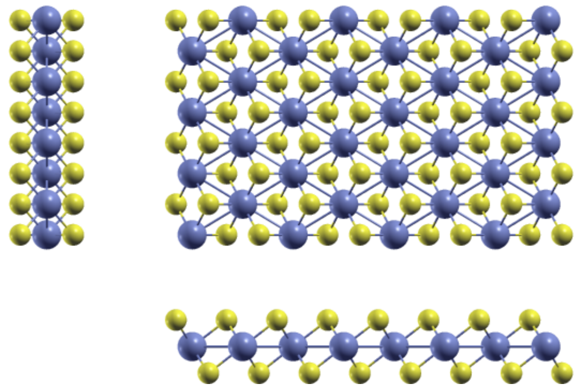

**Geometry:** Views of $CrS_2$ as seen from the $x$ axis (left), the $y$ axis (bottom), and the $z$ axis (center).

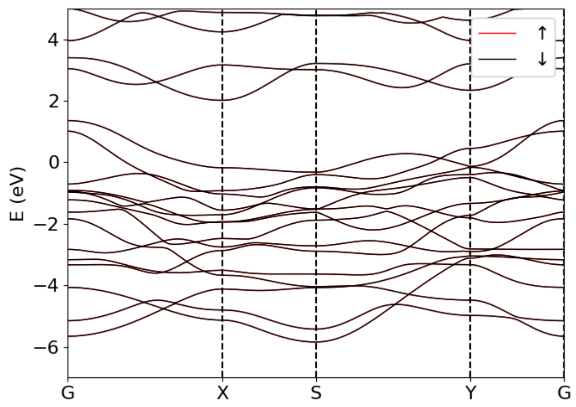


**Electronic bandstructure:** Spin-resolved energy bands of monolayer $CrS_2$ along a high-symmetry path.

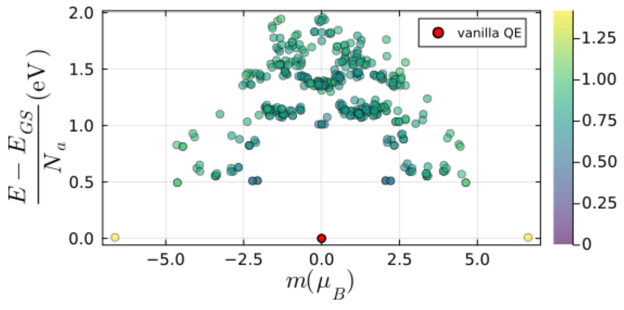


**Unique states:** Representation of 399 self-consistent unique states for monolayer $CrS_2$ identified using RomeoDFT (see Section S6).

**Lattice vectors:** Cartesian components (in [Å]) of the lattice vectors for $CrS_2$.

| | $x$ | $y$ | $z$ |
|---|---|---|---|
| $\boldsymbol{a_1}$ | 0.0000 | −5.4262 | 0.0000 |
| $\boldsymbol{a_2}$ | 3.2888 | 0.0000 | 0.0000 |
| $\boldsymbol{a_3}$ | 0.0000 | 0.0000 | 22.8080 |

**Atomic positions:** Fractional coordinates, Hubbard $U$ (in eV) and magnetic moments (in $\mu_B$, computed from orbital occupations $m_o$ or integration spheres $m_i$) of each atom of monolayer $CrS_2$.

| atom | $x$ | $y$ | $z$ | $U$ | $m_o$ | $m_i$ |
|---|---|---|---|---|---|---|
| Cr | 0.33 | 0.0 | 0.50 | 5.39 | 3.19 | 2.82 |
| Cr | 0.83 | 0.50 | 0.50 | 5.39 | −3.19 | −2.82 |
| S | 1.17 | 0.50 | 0.44 | 0.0 | – | −0.06 |
| S | 0.49 | 0.50 | 0.56 | 0.0 | – | −0.06 |
| S | 0.67 | 0.0 | 0.44 | 0.0 | – | 0.06 |
| S | 0.99 | 0.0 | 0.56 | 0.0 | – | 0.06 |

## CrSBr (FM)

Band gap: 0.22 eV
Total magnetization: $-5.98\ \mu_B/cell$
Absolute magnetization: $8.36\ \mu_B/cell$
MC2D entry: https://mc2d.materialscloud.org/#/details/mc2d-45

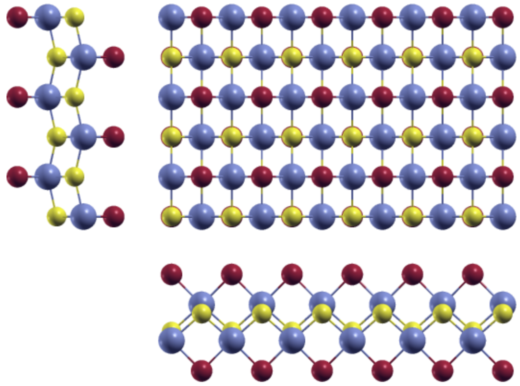

**Geometry:** Views of CrSBr as seen from the $x$ axis (left), the $y$ axis (bottom), and the $z$ axis (center).

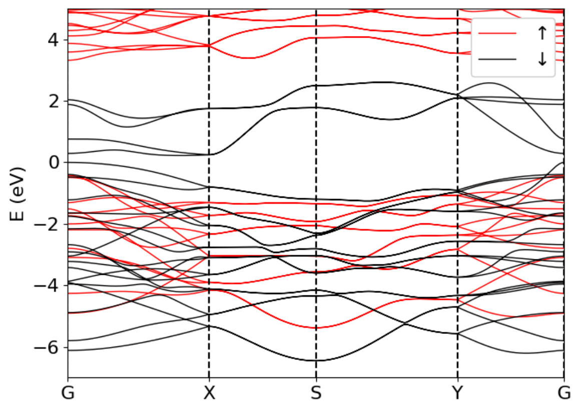


**Electronic bandstructure:** Spin-resolved energy bands of monolayer CrSBr along a high-symmetry path.

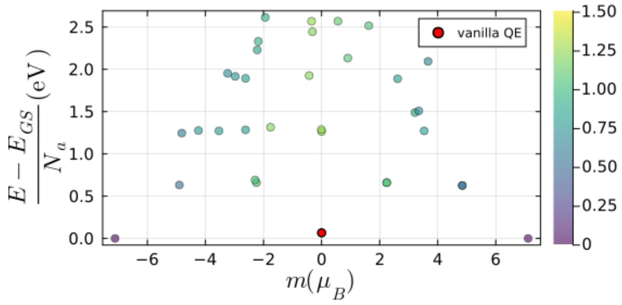


**Unique states:** Representation of 35 self-consistent unique states for monolayer CrSBr identified using RomeoDFT (see Section S6).

**Lattice vectors:** Cartesian components (in [Å]) of the lattice vectors for CrSBr.

| | $x$ | $y$ | $z$ |
|---|---|---|---|
| $\boldsymbol{a_1}$ | 3.5588 | 0.0000 | 0.0000 |
| $\boldsymbol{a_2}$ | 0.0000 | 4.7086 | 0.0000 |
| $\boldsymbol{a_3}$ | 0.0000 | 0.0000 | 25.1694 |

**Atomic positions:** Fractional coordinates, Hubbard $U$ (in eV) and magnetic moments (in $\mu_B$, computed from orbital occupations $m_o$ or integration spheres $m_i$) of each atom of monolayer CrSBr.

| atom | $x$ | $y$ | $z$ | $U$ | $m_o$ | $m_i$ |
|---|---|---|---|---|---|---|
| Cr | 0.50 | 0.0 | 0.46 | 5.26 | −3.56 | −3.15 |
| Cr | 0.0 | 0.50 | 0.54 | 5.26 | −3.56 | −3.15 |
| S | 0.50 | 0.50 | 0.48 | 0.0 | – | 0.34 |
| Br | 0.0 | 0.0 | 0.39 | 0.0 | – | 0.11 |
| S | 0.0 | 0.0 | 0.52 | 0.0 | – | 0.34 |
| Br | 0.50 | 0.50 | 0.61 | 0.0 | – | 0.11 |

## $CrSe_2$ (FM)

Band gap: 0.0 eV
Total magnetization: 5.18 $\mu_B/cell$
Absolute magnetization: 8.99 $\mu_B/cell$
MC2D entry: https://mc2d.materialscloud.org/#/details/mc2d-1651

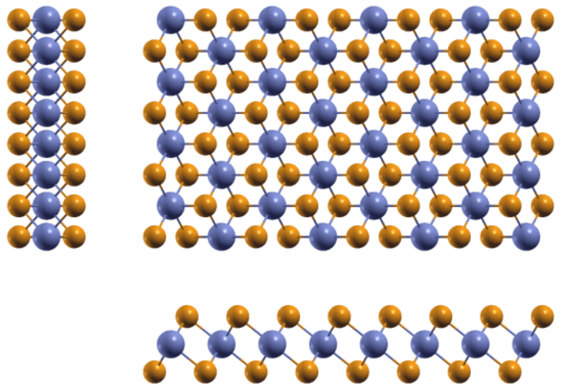

**Geometry:** Views of $CrSe_2$ as seen from the $x$ axis (left), the $y$ axis (bottom), and the $z$ axis (center).

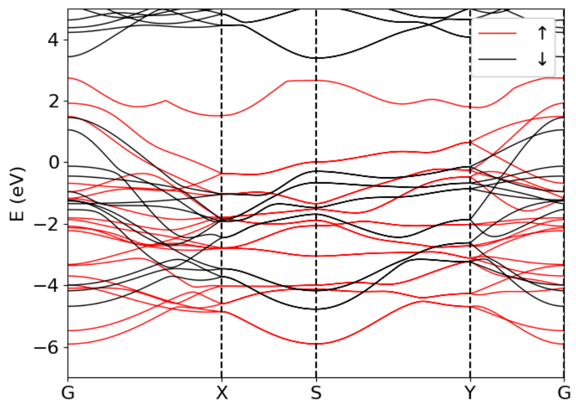


**Electronic bandstructure:** Spin-resolved energy bands of monolayer $CrSe_2$ along a high-symmetry path.

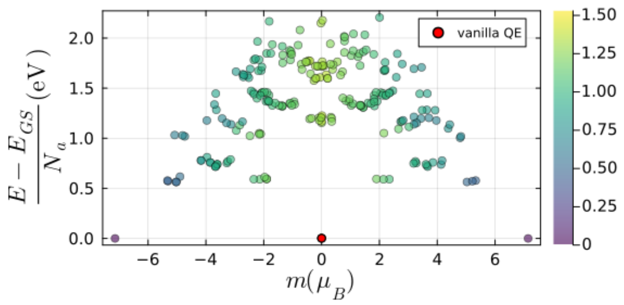


**Unique states:** Representation of 230 self-consistent unique states for monolayer $CrSe_2$ identified using RomeoDFT (see Section S6).

**Lattice vectors:** Cartesian components (in [Å]) of the lattice vectors for $CrSe_2$.

| | $x$ | $y$ | $z$ |
|---|---|---|---|
| $\boldsymbol{a_1}$ | 0.0000 | −5.6736 | 0.0000 |
| $\boldsymbol{a_2}$ | 3.4586 | 0.0000 | 0.0000 |
| $\boldsymbol{a_3}$ | 0.0000 | 0.0000 | 22.8071 |

**Atomic positions:** Fractional coordinates, Hubbard $U$ (in eV) and magnetic moments (in $\mu_B$, computed from orbital occupations $m_o$ or integration spheres $m_i$) of each atom of monolayer $CrSe_2$.

| atom | $x$ | $y$ | $z$ | $U$ | $m_o$ | $m_i$ |
|---|---|---|---|---|---|---|
| Cr | 1.17 | 0.50 | 0.50 | 5.34 | 3.57 | 3.20 |
| Cr | 0.67 | 1.00 | 0.50 | 5.34 | 3.57 | 3.20 |
| Se | 0.33 | 1.00 | 0.43 | 0.0 | – | −0.32 |
| Se | 1.01 | 1.00 | 0.57 | 0.0 | – | −0.32 |
| Se | 0.83 | 0.50 | 0.43 | 0.0 | – | −0.32 |
| Se | 0.51 | 0.50 | 0.57 | 0.0 | – | −0.32 |

## $CrSiSe_3$ (FM)

Band gap: 0.59 eV
Total magnetization: 5.87 $\mu_B/cell$
Absolute magnetization: 9.71 $\mu_B/cell$
MC2D entry: https://mc2d.materialscloud.org/#/details/mc2d-2122

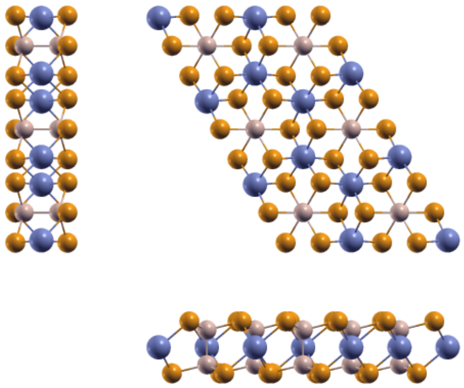

**Geometry:** Views of $CrSiSe_3$ as seen from the $x$ axis (left), the $y$ axis (bottom), and the $z$ axis (center).

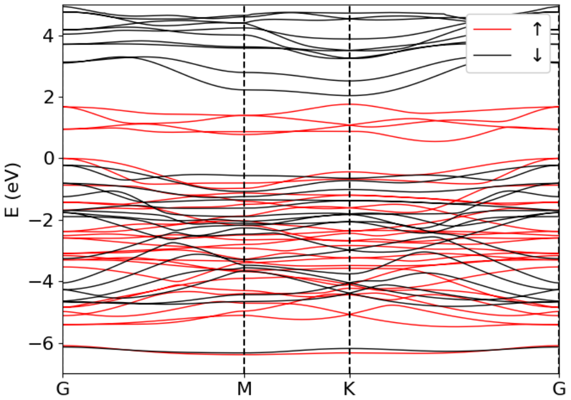


**Electronic bandstructure:** Spin-resolved energy bands of monolayer $CrSiSe_3$ along a high-symmetry path.

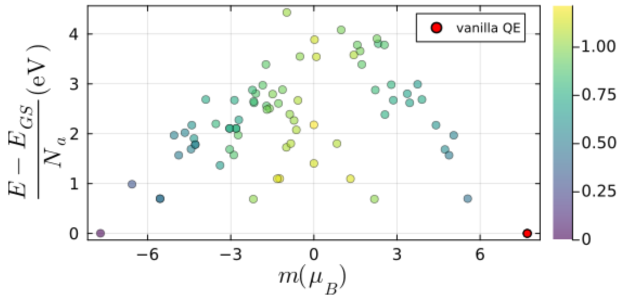


**Unique states:** Representation of 77 self-consistent unique states for monolayer $CrSiSe_3$ identified using RomeoDFT (see Section S6).

**Lattice vectors:** Cartesian components (in [Å]) of the lattice vectors for $CrSiSe_3$.

| | $x$ | $y$ | $z$ |
|---|---|---|---|
| $\boldsymbol{a_1}$ | −3.1005 | −5.3703 | 0.0000 |
| $\boldsymbol{a_2}$ | 6.2011 | 0.0000 | 0.0000 |
| $\boldsymbol{a_3}$ | 0.0000 | 0.0000 | 18.3976 |

**Atomic positions:** Fractional coordinates, Hubbard $U$ (in eV) and magnetic moments (in $\mu_B$, computed from orbital occupations $m_o$ or integration spheres $m_i$) of each atom of monolayer $CrSiSe_3$.

| atom | $x$ | $y$ | $z$ | $U$ | $m_o$ | $m_i$ |
|---|---|---|---|---|---|---|
| Cr | 0.17 | 0.83 | 1.00 | 5.27 | 3.85 | 3.50 |
| Cr | 0.83 | 0.17 | 0.0 | 5.27 | 3.85 | 3.50 |
| Si | 0.50 | 0.50 | 0.94 | 0.0 | – | 0.00 |
| Si | 0.50 | 0.50 | 0.06 | 0.0 | – | 0.00 |
| Se | 0.14 | 0.14 | 0.91 | 0.0 | – | −0.17 |
| Se | 0.86 | 0.50 | 0.91 | 0.0 | – | −0.17 |
| Se | 0.50 | 0.86 | 0.91 | 0.0 | – | −0.17 |
| Se | 0.50 | 0.14 | 0.09 | 0.0 | – | −0.17 |
| Se | 0.86 | 0.86 | 0.09 | 0.0 | – | −0.17 |
| Se | 0.14 | 0.50 | 0.09 | 0.0 | – | −0.17 |

## $CrSiTe_3$ (FM)

Band gap: 0.31 eV
Total magnetization: 5.94 $\mu_B/cell$
Absolute magnetization: 10.64 $\mu_B/cell$
MC2D entry: https://mc2d.materialscloud.org/#/details/mc2d-446

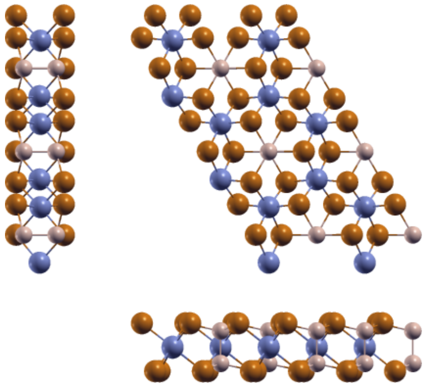

**Geometry:** Views of $CrSiTe_3$ as seen from the $x$ axis (left), the $y$ axis (bottom), and the $z$ axis (center).

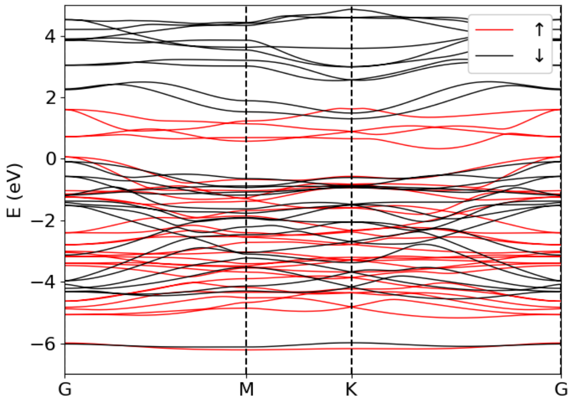


**Electronic bandstructure:** Spin-resolved energy bands of monolayer $CrSiTe_3$ along a high-symmetry path.

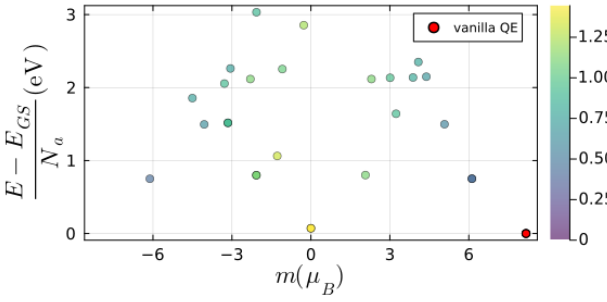


**Unique states:** Representation of 28 self-consistent unique states for monolayer $CrSiTe_3$ identified using RomeoDFT (see Section S6).

**Lattice vectors:** Cartesian components (in [Å]) of the lattice vectors for $CrSiTe_3$.

| | $x$ | $y$ | $z$ |
|---|---|---|---|
| $\boldsymbol{a_1}$ | 6.8270 | 0.0000 | 0.0000 |
| $\boldsymbol{a_2}$ | −3.4135 | 5.9123 | 0.0000 |
| $\boldsymbol{a_3}$ | 0.0000 | 0.0000 | 23.3991 |

**Atomic positions:** Fractional coordinates, Hubbard $U$ (in eV) and magnetic moments (in $\mu_B$, computed from orbital occupations $m_o$ or integration spheres $m_i$) of each atom of monolayer $CrSiTe_3$.

| atom | $x$ | $y$ | $z$ | $U$ | $m_o$ | $m_i$ |
|---|---|---|---|---|---|---|
| Cr | 0.33 | 0.67 | 0.50 | 5.78 | 4.09 | 3.77 |
| Cr | 0.0 | 0.0 | 0.50 | 5.78 | 4.09 | 3.77 |
| Si | 0.67 | 0.33 | 0.45 | 0.0 | – | 0.01 |
| Te | 0.30 | 0.33 | 0.43 | 0.0 | – | −0.18 |
| Te | 0.03 | 0.70 | 0.43 | 0.0 | – | −0.18 |
| Te | 0.67 | 0.97 | 0.43 | 0.0 | – | −0.18 |
| Te | 0.03 | 0.34 | 0.57 | 0.0 | – | −0.18 |
| Te | 0.30 | 0.97 | 0.57 | 0.0 | – | −0.18 |
| Te | 0.66 | 0.70 | 0.57 | 0.0 | – | −0.18 |
| Si | 0.67 | 0.33 | 0.55 | 0.0 | – | 0.01 |

## $Cu_2O_3Cl$ (FM)

Band gap: 0.0 eV
Total magnetization: $-1.47\ \mu_B/cell$
Absolute magnetization: $1.59\ \mu_B/cell$
MC2D entry: https://mc2d.materialscloud.org/#/details/mc2d-2090

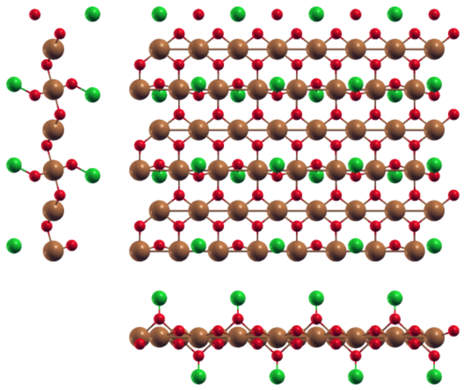

**Geometry:** Views of $Cu_2O_3Cl$ as seen from the $x$ axis (left), the $y$ axis (bottom), and the $z$ axis (center).

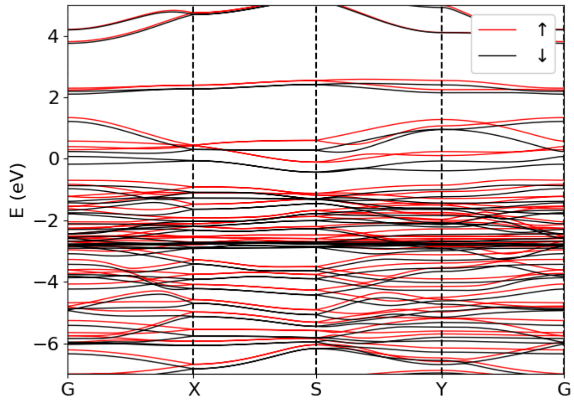


**Electronic bandstructure:** Spin-resolved energy bands of monolayer $Cu_2O_3Cl$ along a high-symmetry path.

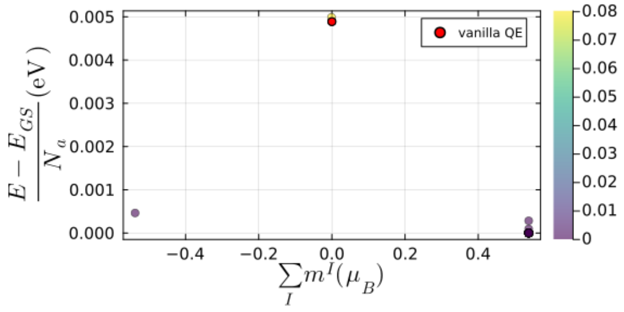


**Unique states:** Representation of 5 self-consistent unique states for monolayer $Cu_2O_3Cl$ identified using RomeoDFT (see Section S6).

**Lattice vectors:** Cartesian components (in [Å]) of the lattice vectors for $Cu_2O_3Cl$.

| | $x$ | $y$ | $z$ |
|---|---|---|---|
| $a_1$ | 0.0000 | −5.6729 | 0.0000 |
| $a_2$ | 5.8375 | 0.0000 | 0.0000 |
| $a_3$ | 0.0000 | 0.0000 | 23.0154 |

**Atomic positions:** Fractional coordinates, Hubbard $U$ (in eV) and magnetic moments (in $\mu_B$, computed from orbital occupations $m_o$ or integration spheres $m_i$) of each atom of monolayer $Cu_2O_3Cl$.

| atom | $x$ | $y$ | $z$ | $U$ | $m_o$ | $m_i$ |
|---|---|---|---|---|---|---|
| Cu | 0.50 | 0.0 | 0.0 | 0.00 | −0.22 | −0.29 |
| Cu | 0.0 | 0.0 | 0.0 | 0.00 | −0.22 | −0.29 |
| Cu | 0.75 | 0.50 | 0.00 | 0.00 | −0.05 | −0.07 |
| Cu | 0.25 | 0.50 | 0.00 | 0.00 | −0.05 | −0.07 |
| O | 0.75 | −0.07 | 0.06 | 0.0 | – | −0.13 |
| O | 0.50 | 0.31 | 0.02 | 0.0 | – | −0.07 |
| O | 0.00 | 0.31 | 0.02 | 0.0 | – | −0.07 |
| Cl | 0.25 | −0.07 | −0.12 | 0.0 | – | −0.03 |
| O | 0.25 | 0.07 | −0.06 | 0.0 | – | −0.13 |
| O | 1.00 | 0.69 | −0.02 | 0.0 | – | −0.07 |
| O | 0.50 | 0.69 | −0.02 | 0.0 | – | −0.07 |
| Cl | 0.75 | 0.07 | 0.12 | 0.0 | – | −0.03 |

## $CuBr_2$ (AFM)

Band gap: 0.0 eV
Total magnetization: 0.0 $\mu_B/cell$
Absolute magnetization: 1.5 $\mu_B/cell$
MC2D entry: https://mc2d.materialscloud.org/#/details/mc2d-2415

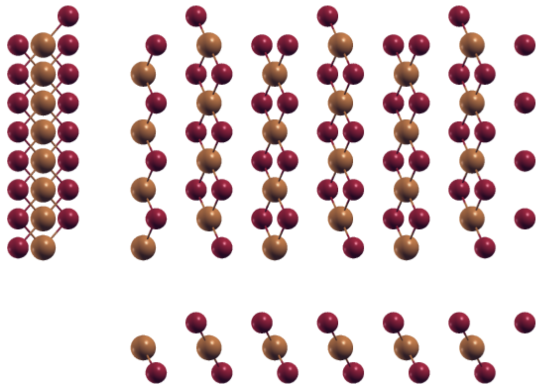

**Geometry:** Views of $CuBr_2$ as seen from the $x$ axis (left), the $y$ axis (bottom), and the $z$ axis (center).

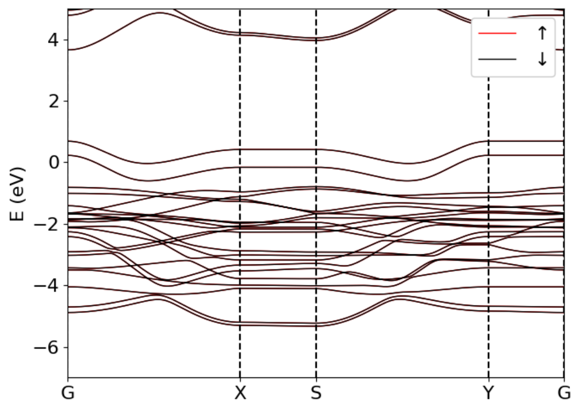


**Electronic bandstructure:** Spin-resolved energy bands of monolayer $CuBr_2$ along a high-symmetry path.

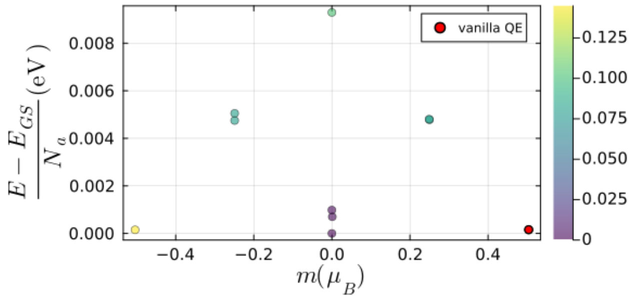


**Unique states:** Representation of 10 self-consistent unique states for monolayer $CuBr_2$ identified using RomeoDFT (see Section S6).

**Lattice vectors:** Cartesian components (in [Å]) of the lattice vectors for $CuBr_2$.

| | $x$ | $y$ | $z$ |
|---|---|---|---|
| $\boldsymbol{a_1}$ | 7.8798 | 0.0000 | 0.0000 |
| $\boldsymbol{a_2}$ | 0.0000 | 3.4708 | 0.0000 |
| $\boldsymbol{a_3}$ | 0.0000 | 0.0000 | 17.8589 |

**Atomic positions:** Fractional coordinates, Hubbard $U$ (in eV) and magnetic moments (in $\mu_B$, computed from orbital occupations $m_o$ or integration spheres $m_i$) of each atom of monolayer $CuBr_2$.

| atom | $x$ | $y$ | $z$ | $U$ | $m_o$ | $m_i$ |
|---|---|---|---|---|---|---|
| Cu | 0.50 | 0.50 | 0.0 | 0.00 | 0.25 | 0.33 |
| Cu | 0.0 | 0.0 | 0.0 | 0.00 | −0.25 | −0.33 |
| Br | 0.90 | 0.50 | 0.92 | 0.0 | – | −0.13 |
| Br | 0.60 | 1.00 | 0.08 | 0.0 | – | 0.13 |
| Br | 1.40 | 1.00 | 0.92 | 0.0 | – | 0.13 |
| Br | 0.10 | 0.50 | 0.08 | 0.0 | – | −0.13 |

# $CuCl_2$ (FM)

Band gap: 0.40 eV
Total magnetization: 1.99 $\mu_B/cell$
Absolute magnetization: 2.11 $\mu_B/cell$
MC2D entry: https://mc2d.materialscloud.org/#/details/mc2d-2159

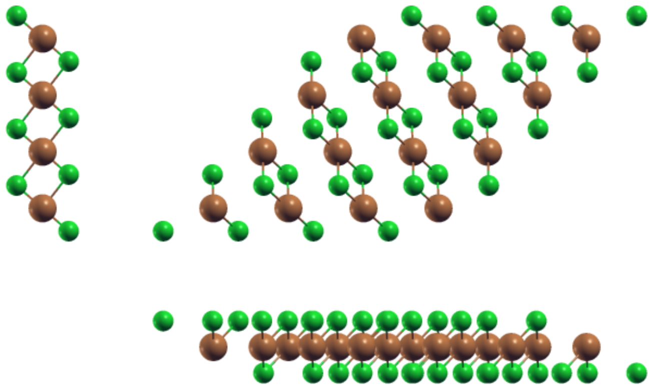

**Geometry:** Views of $CuCl_2$ as seen from the $x$ axis (left), the $y$ axis (bottom), and the $z$ axis (center).

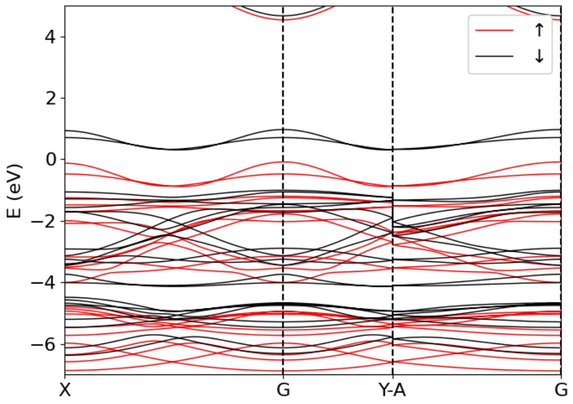


**Electronic bandstructure:** Spin-resolved energy bands of monolayer $CuCl_2$ along a high-symmetry path.

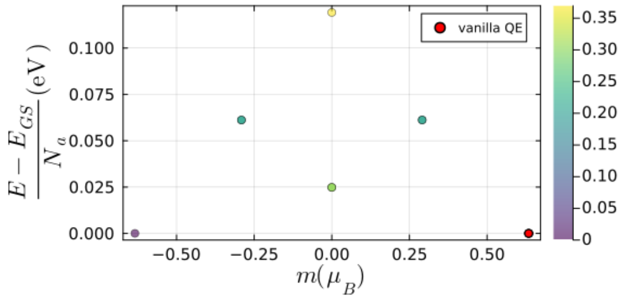


**Unique states:** Representation of 10 self-consistent unique states for monolayer $CuCl_2$ identified using RomeoDFT (see Section S6).

**Lattice vectors:** Cartesian components (in [Å]) of the lattice vectors for $CuCl_2$.

| | $x$ | $y$ | $z$ |
|---|---|---|---|
| $\boldsymbol{a_1}$ | 8.0354 | 0.1536 | 0.0000 |
| $\boldsymbol{a_2}$ | 2.5824 | 3.0788 | 0.0000 |
| $\boldsymbol{a_3}$ | 0.0000 | 0.0000 | 22.7596 |

**Atomic positions:** Fractional coordinates, Hubbard $U$ (in eV) and magnetic moments (in $\mu_B$, computed from orbital occupations $m_o$ or integration spheres $m_i$) of each atom of monolayer $CuCl_2$.

| atom | $x$ | $y$ | $z$ | $U$ | $m_o$ | $m_i$ |
|---|---|---|---|---|---|---|
| Cu | 0.24 | 0.47 | 0.50 | 7.60 | 0.32 | 0.43 |
| Cu | 0.74 | 0.47 | 0.50 | 7.60 | 0.32 | 0.43 |
| Cl | 0.44 | 0.88 | 0.56 | 0.0 | – | 0.22 |
| Cl | 0.03 | 0.07 | 0.44 | 0.0 | – | 0.22 |
| Cl | 0.94 | 0.88 | 0.56 | 0.0 | – | 0.22 |
| Cl | 0.53 | 0.07 | 0.44 | 0.0 | – | 0.22 |

## $CuO_2$ ($P\bar{1}$) (FM)

Band gap: 0.0 eV
Total magnetization: 2.03 $\mu_B/cell$
Absolute magnetization: 2.18 $\mu_B/cell$
MC2D entry: https://mc2d.materialscloud.org/#/details/mc2d-2412

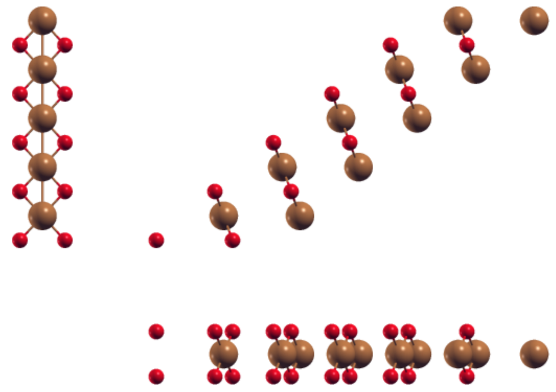

**Geometry:** Views of $CuO_2$ ($P\bar{1}$) as seen from the $x$ axis (left), the $y$ axis (bottom), and the $z$ axis (center).

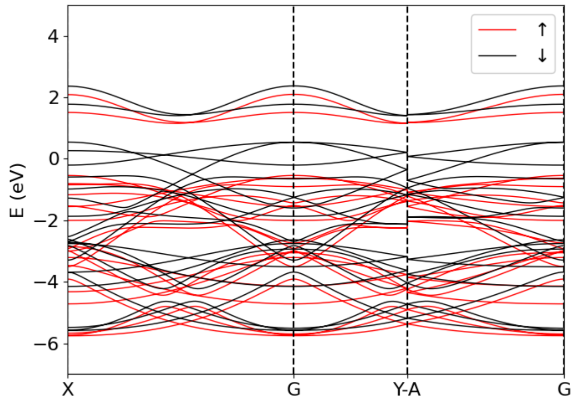


**Electronic bandstructure:** Spin-resolved energy bands of monolayer $CuO_2$ ($P\bar{1}$) along a high-symmetry path.

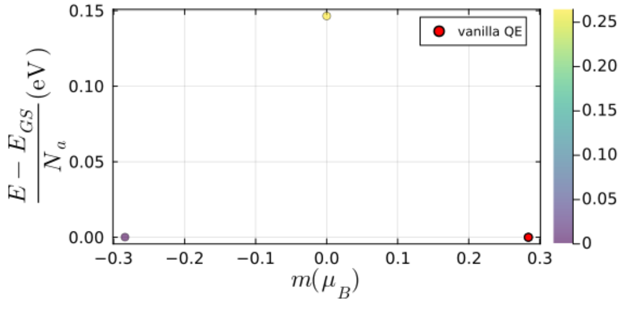


**Unique states:** Representation of 4 self-consistent unique states for monolayer $CuO_2$ ($P\bar{1}$) identified using RomeoDFT (see Section S6).

**Lattice vectors:** Cartesian components (in [Å]) of the lattice vectors for $CuO_2$ ($P\bar{1}$).

| | $x$ | $y$ | $z$ |
|---|---|---|---|
| $\boldsymbol{a_1}$ | 2.7349 | 7.5563 | 0.0000 |
| $\boldsymbol{a_2}$ | −1.3675 | 3.7781 | 0.0000 |
| $\boldsymbol{a_3}$ | 0.0000 | 0.0000 | 16.5346 |

**Atomic positions:** Fractional coordinates, Hubbard $U$ (in eV) and magnetic moments (in $\mu_B$, computed from orbital occupations $m_o$ or integration spheres $m_i$) of each atom of monolayer $CuO_2$ ($P\bar{1}$).

| atom | $x$ | $y$ | $z$ | $U$ | $m_o$ | $m_i$ |
|---|---|---|---|---|---|---|
| Cu | 0.25 | 0.50 | −0.50 | 0.00 | 0.14 | 0.23 |
| Cu | 0.75 | 0.50 | −0.50 | 0.00 | 0.14 | 0.23 |
| O | 0.0 | 0.0 | −0.57 | 0.0 | – | 0.33 |
| O | 0.0 | 0.0 | −0.43 | 0.0 | – | 0.33 |
| O | 0.50 | 0.0 | −0.57 | 0.0 | – | 0.33 |
| O | 0.50 | 0.0 | −0.43 | 0.0 | – | 0.33 |

# $CuO_2$ (Pmmn) (FM)

Band gap: 0.0 eV
Total magnetization: 2.11 $\mu_B/cell$
Absolute magnetization: 2.23 $\mu_B/cell$
MC2D entry: https://mc2d.materialscloud.org/#/details/mc2d-1858

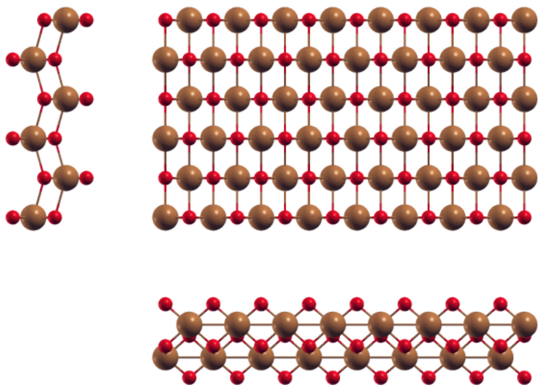

**Geometry:** Views of $CuO_2$ (Pmmn) as seen from the $x$ axis (left), the $y$ axis (bottom), and the $z$ axis (center).

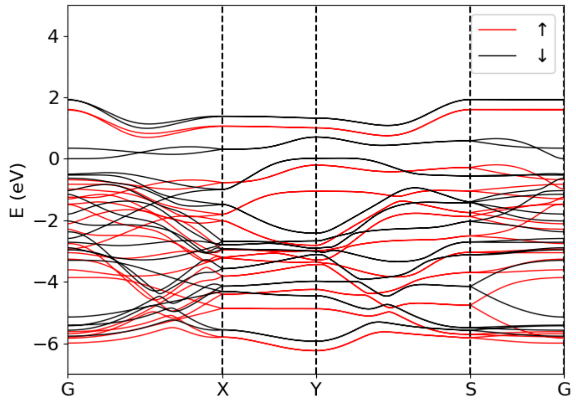


**Electronic bandstructure:** Spin-resolved energy bands of monolayer $CuO_2$ (Pmmn) along a high-symmetry path.

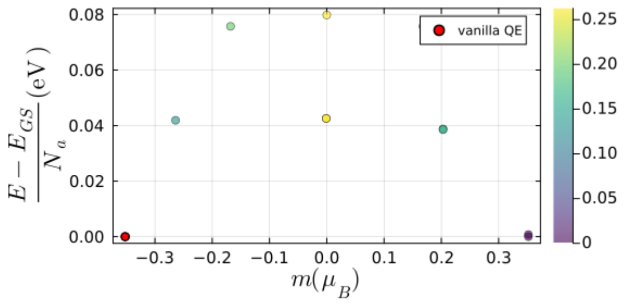


**Unique states:** Representation of 11 self-consistent unique states for monolayer $CuO_2$ (Pmmn) identified using RomeoDFT (see Section S6).

**Lattice vectors:** Cartesian components (in [Å]) of the lattice vectors for $CuO_2$ (Pmmn).

| | $x$ | $y$ | $z$ |
|---|---|---|---|
| $\boldsymbol{a_1}$ | 2.7654 | 0.0000 | 0.0000 |
| $\boldsymbol{a_2}$ | 0.0000 | 4.5637 | 0.0000 |
| $\boldsymbol{a_3}$ | 0.0000 | 0.0000 | 20.4974 |

**Atomic positions:** Fractional coordinates, Hubbard $U$ (in eV) and magnetic moments (in $\mu_B$, computed from orbital occupations $m_o$ or integration spheres $m_i$) of each atom of monolayer $CuO_2$ (Pmmn).

| atom | $x$ | $y$ | $z$ | $U$ | $m_o$ | $m_i$ |
|---|---|---|---|---|---|---|
| Cu | −0.25 | −1.25 | −0.05 | 0.00 | 0.18 | 0.27 |
| Cu | 0.25 | −0.75 | 0.05 | 0.00 | 0.18 | 0.27 |
| O | 0.25 | −1.25 | 0.01 | 0.0 | – | 0.36 |
| O | −0.25 | −0.75 | −0.01 | 0.0 | – | 0.36 |
| O | 0.25 | −1.25 | −0.10 | 0.0 | – | 0.33 |
| O | −0.25 | −0.75 | 0.10 | 0.0 | – | 0.33 |

## $CuSiO_3$ (FM)

Band gap: 0.99 eV
Total magnetization: $-2.0\ \mu_B/cell$
Absolute magnetization: 2.12 $\mu_B/cell$
MC2D entry: https://mc2d.materialscloud.org/#/details/mc2d-933

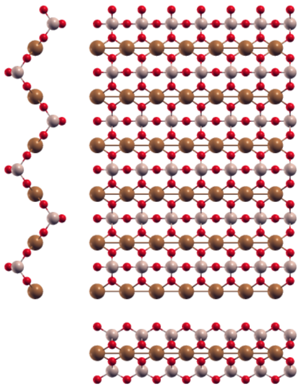

**Geometry:** Views of $CuSiO_3$ as seen from the $x$ axis (left), the $y$ axis (bottom), and the $z$ axis (center).

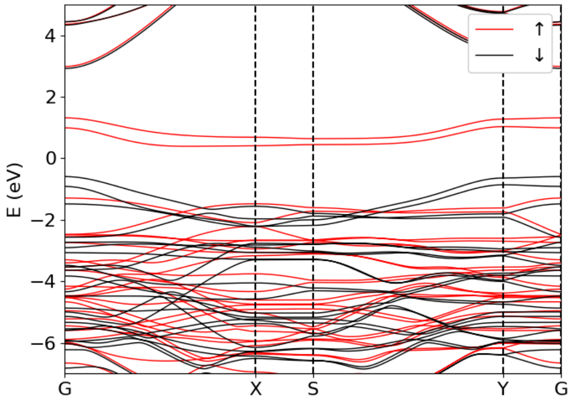


**Electronic bandstructure:** Spin-resolved energy bands of monolayer $CuSiO_3$ along a high-symmetry path.

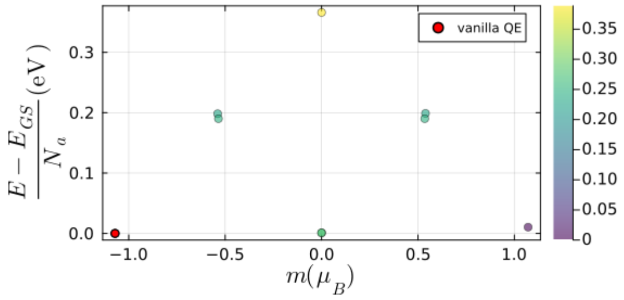


**Unique states:** Representation of 10 self-consistent unique states for monolayer $CuSiO_3$ identified using RomeoDFT (see Section S6).

**Lattice vectors:** Cartesian components (in [Å]) of the lattice vectors for $CuSiO_3$.

| | $x$ | $y$ | $z$ |
|---|---|---|---|
| $\boldsymbol{a_1}$ | 2.8722 | 0.0000 | 0.0000 |
| $\boldsymbol{a_2}$ | 0.0000 | 9.4479 | 0.0000 |
| $\boldsymbol{a_3}$ | 0.0000 | 0.0000 | 25.5519 |

**Atomic positions:** Fractional coordinates, Hubbard $U$ (in eV) and magnetic moments (in $\mu_B$, computed from orbital occupations $m_o$ or integration spheres $m_i$) of each atom of monolayer $CuSiO_3$.

| atom | $x$ | $y$ | $z$ | $U$ | $m_o$ | $m_i$ |
|---|---|---|---|---|---|---|
| Cu | 0.0 | 0.0 | 0.50 | 5.44 | −0.54 | −0.66 |
| Cu | 0.0 | 0.50 | 0.50 | 5.44 | −0.53 | −0.66 |
| Si | 0.50 | 0.25 | 0.57 | 0.0 | – | −0.01 |
| Si | 0.50 | 0.75 | 0.43 | 0.0 | – | −0.01 |
| O | 0.0 | 0.25 | 0.60 | 0.0 | – | −0.02 |
| O | 0.0 | 0.75 | 0.40 | 0.0 | – | −0.02 |
| O | 0.50 | 0.89 | 0.47 | 0.0 | – | −0.13 |
| O | 0.50 | 0.40 | 0.53 | 0.0 | – | −0.13 |
| O | 0.50 | 0.60 | 0.47 | 0.0 | – | −0.13 |
| O | 0.50 | 0.11 | 0.53 | 0.0 | – | −0.13 |

## $CuTl_2F_4$ (AFM)

Band gap: 1.12 eV
Total magnetization: $-0.0\ \mu_B/cell$
Absolute magnetization: 1.98 $\mu_B/cell$
MC2D entry: https://mc2d.materialscloud.org/#/details/mc2d-1787

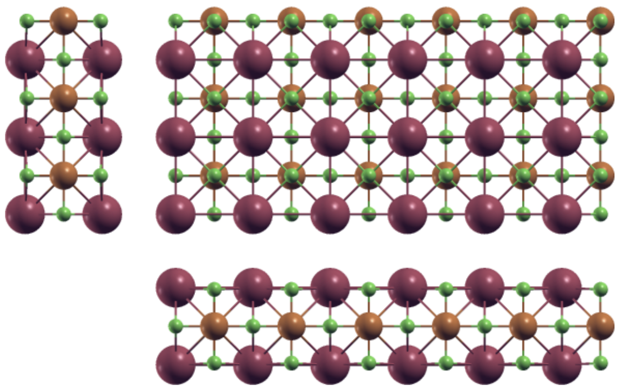

**Geometry:** Views of $CuTl_2F_4$ as seen from the $x$ axis (left), the $y$ axis (bottom), and the $z$ axis (center).

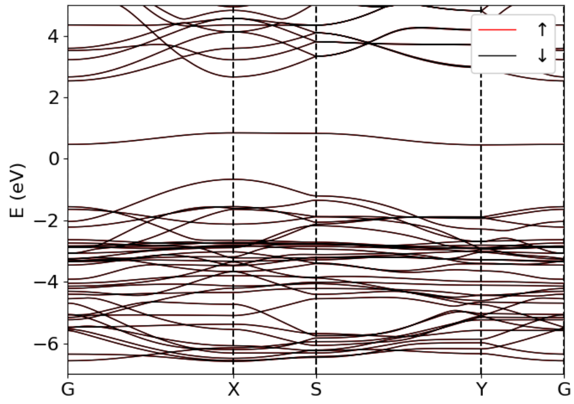


**Electronic bandstructure:** Spin-resolved energy bands of monolayer $CuTl_2F_4$ along a high-symmetry path.

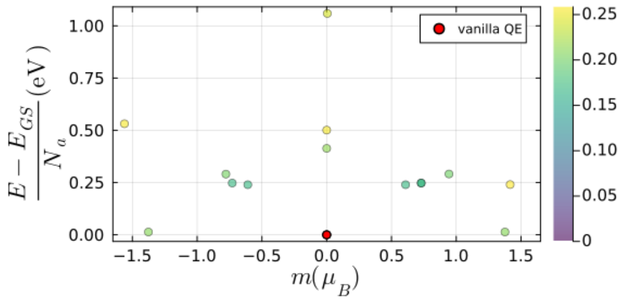


**Unique states:** Representation of 17 self-consistent unique states for monolayer $CuTl_2F_4$ identified using RomeoDFT (see Section S6).

**Lattice vectors:** Cartesian components (in [Å]) of the lattice vectors for $CuTl_2F_4$.

| | $x$ | $y$ | $z$ |
|---|---|---|---|
| $a_1$ | 8.2773 | 0.0000 | 0.0000 |
| $a_2$ | 0.0000 | 4.1386 | 0.0000 |
| $a_3$ | 0.0000 | 0.0000 | 20.0263 |

**Atomic positions:** Fractional coordinates, Hubbard $U$ (in eV) and magnetic moments (in $\mu_B$, computed from orbital occupations $m_o$ or integration spheres $m_i$) of each atom of monolayer $CuTl_2F_4$.

| atom | $x$ | $y$ | $z$ | $U$ | $m_o$ | $m_i$ |
|---|---|---|---|---|---|---|
| Cu | 0.25 | −0.50 | 0.0 | 5.03 | −0.68 | −0.75 |
| Cu | 0.75 | −0.50 | 0.0 | 5.03 | 0.68 | 0.75 |
| Tl | 0.0 | 0.0 | −0.10 | 0.0 | – | 0.0 |
| Tl | 0.0 | 0.0 | 0.10 | 0.0 | – | 0.0 |
| F | 0.25 | −0.50 | −0.10 | 0.0 | – | −0.07 |
| F | 0.25 | 0.0 | 0.0 | 0.0 | – | −0.03 |
| F | 0.0 | −0.50 | 0.0 | 0.0 | – | 0.0 |
| F | 0.25 | −0.50 | 0.10 | 0.0 | – | −0.07 |
| Tl | 0.50 | 0.0 | −0.10 | 0.0 | – | 0.0 |
| Tl | 0.50 | 0.0 | 0.10 | 0.0 | – | 0.0 |
| F | 0.75 | −0.50 | −0.10 | 0.0 | – | 0.07 |
| F | 0.75 | 0.0 | 0.0 | 0.0 | – | 0.03 |
| F | 0.50 | −0.50 | 0.0 | 0.0 | – | 0.0 |
| F | 0.75 | −0.50 | 0.10 | 0.0 | – | 0.07 |

## $DyAsO_4$ (FM)

Band gap: 1.42 eV
Total magnetization: 10.0 $\mu_B/cell$
Absolute magnetization: 10.32 $\mu_B/cell$
MC2D entry: https://mc2d.materialscloud.org/#/details/mc2d-2305

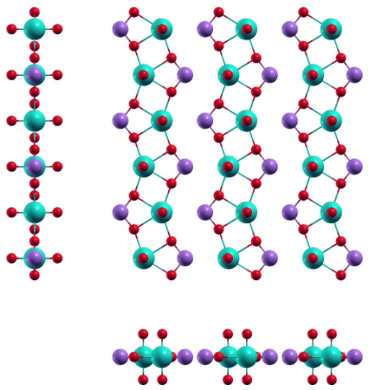

**Geometry:** Views of $DyAsO_4$ as seen from the $x$ axis (left), the $y$ axis (bottom), and the $z$ axis (center).

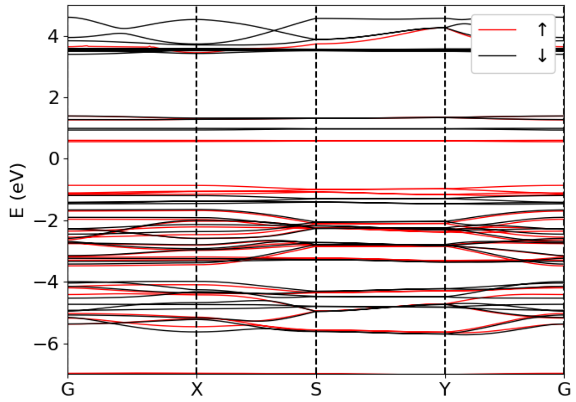


**Electronic bandstructure:** Spin-resolved energy bands of monolayer $DyAsO_4$ along a high-symmetry path.

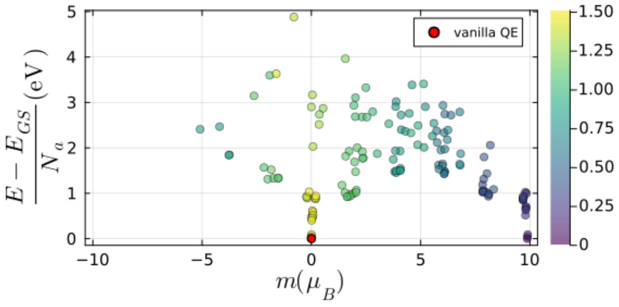


**Unique states:** Representation of 142 self-consistent unique states for monolayer $DyAsO_4$ identified using RomeoDFT (see Section S6).

**Lattice vectors:** Cartesian components (in [Å]) of the lattice vectors for $DyAsO_4$.

| | $x$ | $y$ | $z$ |
|---|---|---|---|
| $\boldsymbol{a_1}$ | 0.0000 | 6.7018 | 0.0000 |
| $\boldsymbol{a_2}$ | −7.2520 | 0.0000 | 0.0000 |
| $\boldsymbol{a_3}$ | 0.0000 | 0.0000 | 19.2418 |

**Atomic positions:** Fractional coordinates, Hubbard $U$ (in eV) and magnetic moments (in $\mu_B$, computed from orbital occupations $m_o$ or integration spheres $m_i$) of each atom of monolayer $DyAsO_4$.

| atom | $x$ | $y$ | $z$ | $U$ | $m_o$ | $m_i$ |
|---|---|---|---|---|---|---|
| Dy | 0.61 | 0.75 | 0.0 | 7.72 | 4.94 | 4.72 |
| Dy | 0.39 | 0.25 | 0.0 | 7.72 | 4.94 | 4.72 |
| O | 0.61 | 0.75 | 0.09 | 0.0 | – | −0.02 |
| O | 0.39 | 0.25 | 0.09 | 0.0 | – | −0.02 |
| As | 0.12 | 0.75 | 0.0 | 0.0 | – | 0.00 |
| As | 0.88 | 0.25 | 0.0 | 0.0 | – | 0.00 |
| O | 0.61 | 0.75 | −0.09 | 0.0 | – | −0.02 |
| O | 0.39 | 0.25 | −0.09 | 0.0 | – | −0.02 |
| O | 0.28 | 0.57 | 0.0 | 0.0 | – | 0.00 |
| O | 0.72 | 0.07 | 0.0 | 0.0 | – | 0.00 |
| O | 0.72 | 0.43 | 0.0 | 0.0 | – | 0.00 |
| O | 0.28 | 0.93 | 0.0 | 0.0 | – | 0.00 |

## $DyBr_3$ (FM)

Band gap: 1.42 eV
Total magnetization: $-0.0\ \mu_B/cell$
Absolute magnetization: $10.17\ \mu_B/cell$
MC2D entry: https://mc2d.materialscloud.org/#/details/mc2d-2409

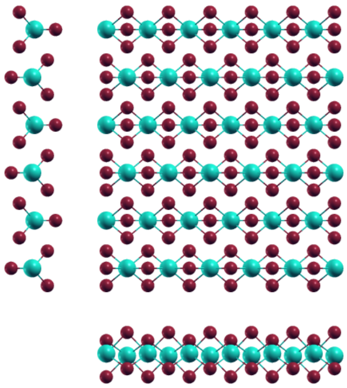

**Geometry:** Views of $DyBr_3$ as seen from the $x$ axis (left), the $y$ axis (bottom), and the $z$ axis (center).

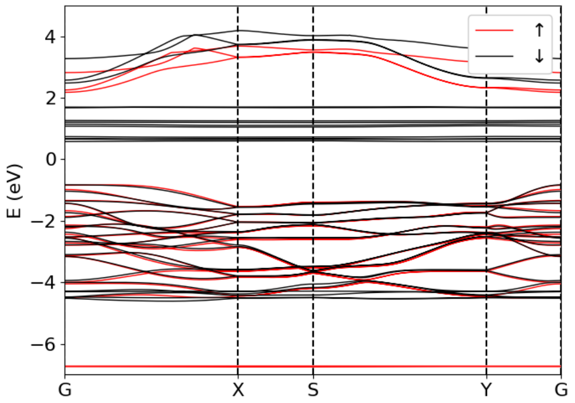


**Electronic bandstructure:** Spin-resolved energy bands of monolayer $DyBr_3$ along a high-symmetry path.

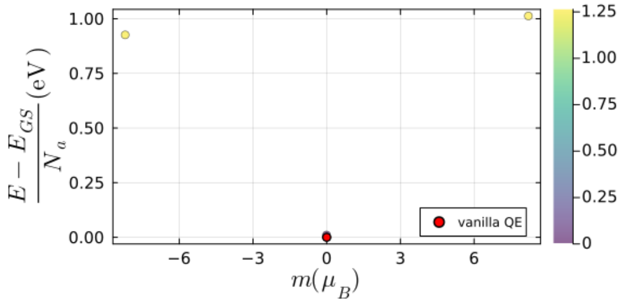


**Unique states:** Representation of 4 self-consistent unique states for monolayer $DyBr_3$ identified using RomeoDFT (see Section S6).

**Lattice vectors:** Cartesian components (in [Å]) of the lattice vectors for $DyBr_3$.

| | $x$ | $y$ | $z$ |
|---|---|---|---|
| $\boldsymbol{a_1}$ | 3.9774 | 0.0000 | 0.0000 |
| $\boldsymbol{a_2}$ | 0.0000 | 9.1688 | 0.0000 |
| $\boldsymbol{a_3}$ | 0.0000 | 0.0000 | 20.4191 |

**Atomic positions:** Fractional coordinates, Hubbard $U$ (in eV) and magnetic moments (in $\mu_B$, computed from orbital occupations $m_o$ or integration spheres $m_i$) of each atom of monolayer $DyBr_3$.

| atom | $x$ | $y$ | $z$ | $U$ | $m_o$ | $m_i$ |
|---|---|---|---|---|---|---|
| Dy | 0.25 | −1.25 | −0.01 | 4.52 | 4.96 | 4.99 |
| Dy | −0.25 | −0.75 | 0.01 | 4.52 | −4.95 | −4.99 |
| Br | −0.25 | −1.43 | 0.07 | 0.0 | – | −0.01 |
| Br | −0.25 | −1.07 | 0.07 | 0.0 | – | −0.01 |
| Br | 0.25 | −0.93 | −0.07 | 0.0 | – | 0.01 |
| Br | 0.25 | −0.57 | −0.07 | 0.0 | – | 0.01 |
| Br | −0.25 | −1.25 | −0.10 | 0.0 | – | −0.01 |
| Br | 0.25 | −0.75 | 0.10 | 0.0 | – | 0.01 |

## $DyI_3$ (AFM)

Band gap: 2.73 eV
Total magnetization: 0.0 $\mu_B/cell$
Absolute magnetization: 10.27 $\mu_B/cell$
MC2D entry: https://mc2d.materialscloud.org/#/details/mc2d-2056

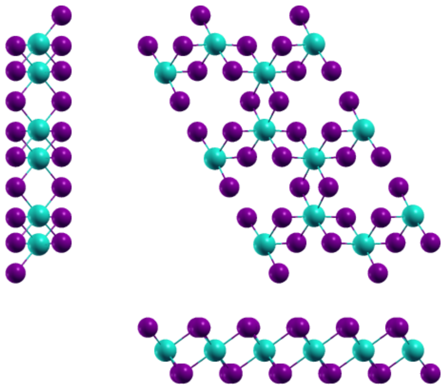

**Geometry:** Views of $DyI_3$ as seen from the $x$ axis (left), the $y$ axis (bottom), and the $z$ axis (center).

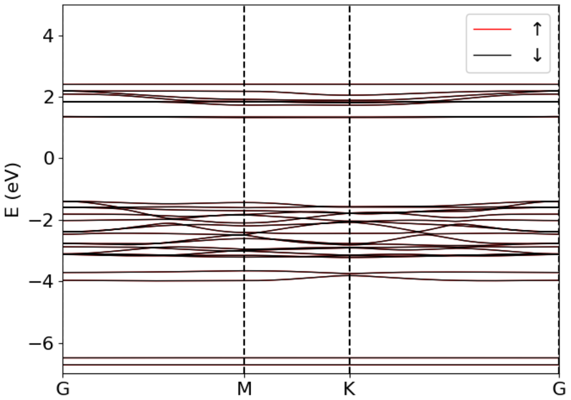


**Electronic bandstructure:** Spin-resolved energy bands of monolayer $DyI_3$ along a high-symmetry path.

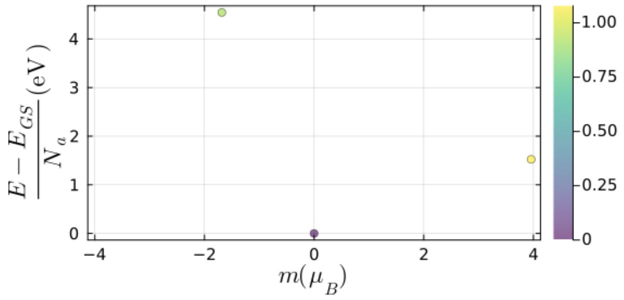


**Unique states:** Representation of 4 self-consistent unique states for monolayer $DyI_3$ identified using RomeoDFT (see Section S6).

**Lattice vectors:** Cartesian components (in [Å]) of the lattice vectors for $DyI_3$.

| | $x$ | $y$ | $z$ |
|---|---|---|---|
| $\boldsymbol{a_1}$ | −3.8435 | −6.6572 | 0.0000 |
| $\boldsymbol{a_2}$ | 7.6870 | 0.0000 | 0.0000 |
| $\boldsymbol{a_3}$ | 0.0000 | 0.0000 | 19.0380 |

**Atomic positions:** Fractional coordinates, Hubbard $U$ (in eV) and magnetic moments (in $\mu_B$, computed from orbital occupations $m_o$ or integration spheres $m_i$) of each atom of monolayer $DyI_3$.

| atom | $x$ | $y$ | $z$ | $U$ | $m_o$ | $m_i$ |
|---|---|---|---|---|---|---|
| Dy | −0.17 | −0.33 | 0.00 | 7.42 | 4.99 | 5.04 |
| Dy | 0.17 | 0.33 | 0.00 | 7.42 | −4.99 | −5.04 |
| I | 0.50 | 0.65 | 0.09 | 0.0 | – | 0.0 |
| I | −0.15 | 0.00 | −0.09 | 0.0 | – | 0.0 |
| I | 0.15 | 0.65 | −0.09 | 0.0 | – | 0.0 |
| I | 0.50 | 0.35 | −0.09 | 0.0 | – | 0.0 |
| I | 0.15 | 0.00 | 0.09 | 0.0 | – | 0.0 |
| I | −0.15 | 0.35 | 0.09 | 0.0 | – | 0.0 |

## DySI (FM)

Band gap: 2.63 eV
Total magnetization: 10.0 $\mu_B/cell$
Absolute magnetization: 10.28 $\mu_B/cell$
MC2D entry: https://mc2d.materialscloud.org/#/details/mc2d-2403

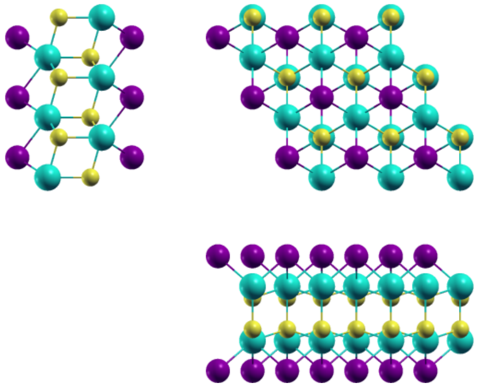

**Geometry:** Views of DySI as seen from the $x$ axis (left), the $y$ axis (bottom), and the $z$ axis (center).

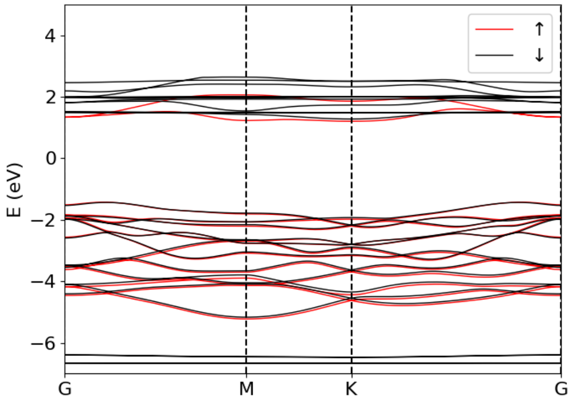


**Electronic bandstructure:** Spin-resolved energy bands of monolayer DySI along a high-symmetry path.

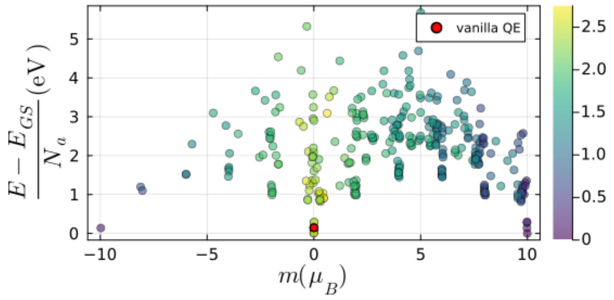


**Unique states:** Representation of 324 self-consistent unique states for monolayer DySI identified using RomeoDFT (see Section S6).

**Lattice vectors:** Cartesian components (in [Å]) of the lattice vectors for DySI.

| | $x$ | $y$ | $z$ |
|---|---|---|---|
| $\boldsymbol{a_1}$ | 2.2541 | −3.9043 | 0.0000 |
| $\boldsymbol{a_2}$ | 2.2541 | 3.9043 | 0.0000 |
| $\boldsymbol{a_3}$ | 0.0000 | 0.0000 | 26.8530 |

**Atomic positions:** Fractional coordinates, Hubbard $U$ (in eV) and magnetic moments (in $\mu_B$, computed from orbital occupations $m_o$ or integration spheres $m_i$) of each atom of monolayer DySI.

| atom | $x$ | $y$ | $z$ | $U$ | $m_o$ | $m_i$ |
|---|---|---|---|---|---|---|
| Dy | −0.33 | −0.17 | −0.07 | 7.48 | 5.00 | 5.01 |
| Dy | 0.33 | 0.17 | 0.07 | 7.48 | 5.00 | 5.01 |
| S | −0.33 | −0.17 | 0.04 | 0.0 | – | −0.03 |
| S | 0.33 | 0.17 | −0.04 | 0.0 | – | −0.03 |
| I | 0.0 | 0.50 | −0.14 | 0.0 | – | −0.02 |
| I | 0.0 | 0.50 | 0.14 | 0.0 | – | −0.02 |

## $DyTe_3$ (AFM)

Band gap: 0.0 eV
Total magnetization: 0.02 $\mu_B/cell$
Absolute magnetization: 10.27 $\mu_B/cell$
MC2D entry: https://mc2d.materialscloud.org/#/details/mc2d-1338

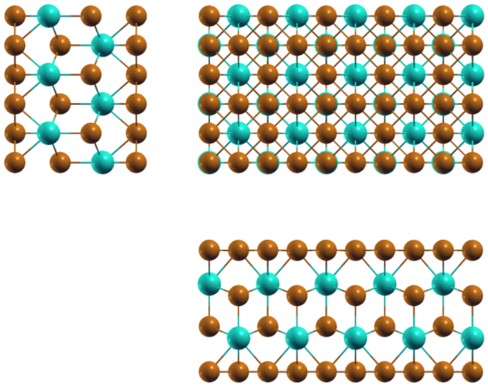

**Geometry:** Views of $DyTe_3$ as seen from the $x$ axis (left), the $y$ axis (bottom), and the $z$ axis (center).

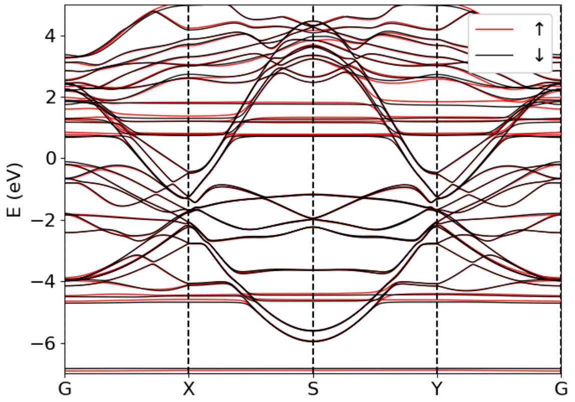


**Electronic bandstructure:** Spin-resolved energy bands of monolayer $DyTe_3$ along a high-symmetry path.

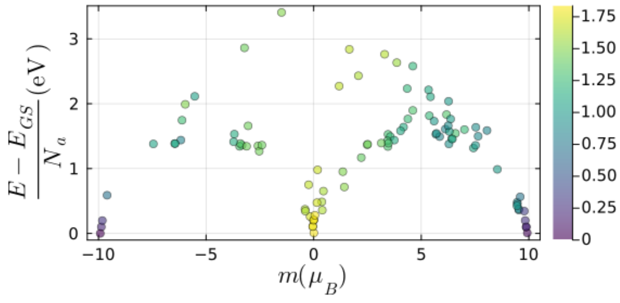


**Unique states:** Representation of 98 self-consistent unique states for monolayer $DyTe_3$ identified using RomeoDFT (see Section S6).

**Lattice vectors:** Cartesian components (in [Å]) of the lattice vectors for $DyTe_3$.

| | $x$ | $y$ | $z$ |
|---|---|---|---|
| $\boldsymbol{a_1}$ | 4.3429 | 0.0000 | 0.0000 |
| $\boldsymbol{a_2}$ | 0.0000 | 4.3447 | 0.0000 |
| $\boldsymbol{a_3}$ | 0.0000 | 0.0000 | 28.9657 |

**Atomic positions:** Fractional coordinates, Hubbard $U$ (in eV) and magnetic moments (in $\mu_B$, computed from orbital occupations $m_o$ or integration spheres $m_i$) of each atom of monolayer $DyTe_3$.

| atom | $x$ | $y$ | $z$ | $U$ | $m_o$ | $m_i$ |
|---|---|---|---|---|---|---|
| Dy | 0.50 | 0.75 | 0.57 | 4.79 | −4.95 | −5.02 |
| Dy | 1.00 | 0.25 | 0.43 | 4.79 | 4.97 | 5.03 |
| Te | 0.50 | 0.75 | 0.46 | 0.0 | – | −0.01 |
| Te | 0.50 | 0.25 | 0.34 | 0.0 | – | −0.01 |
| Te | 0.50 | 0.25 | 0.66 | 0.0 | – | 0.01 |
| Te | 0.0 | 0.25 | 0.54 | 0.0 | – | 0.02 |
| Te | 0.0 | 0.75 | 0.66 | 0.0 | – | 0.01 |
| Te | 1.00 | 0.75 | 0.34 | 0.0 | – | −0.01 |

# $DyVO_4$ (AFM)

Band gap: 3.27 eV
Total magnetization: 0.0 $\mu_B/cell$
Absolute magnetization: 10.08 $\mu_B/cell$
MC2D entry: https://mc2d.materialscloud.org/#/details/mc2d-2277

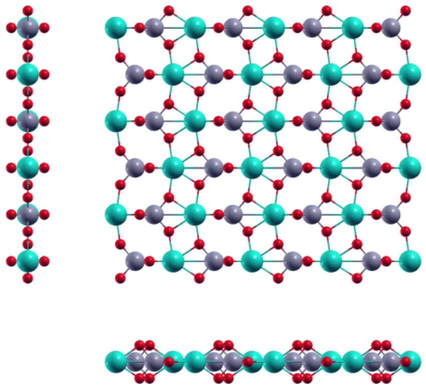

**Geometry:** Views of $DyVO_4$ as seen from the $x$ axis (left), the $y$ axis (bottom), and the $z$ axis (center).

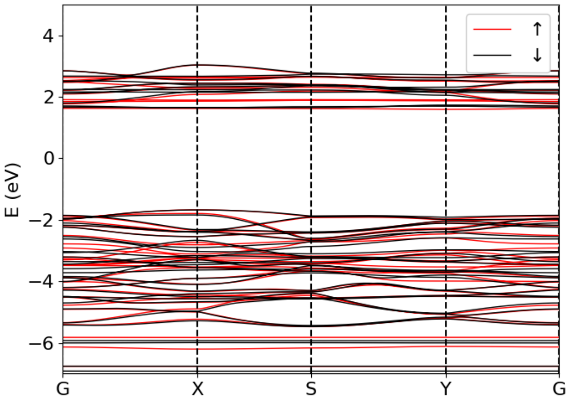


**Electronic bandstructure:** Spin-resolved energy bands of monolayer $DyVO_4$ along a high-symmetry path.

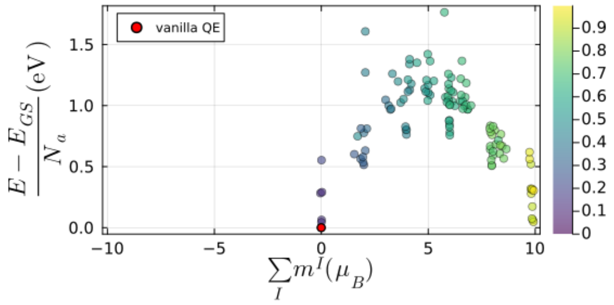


**Unique states:** Representation of 116 self-consistent unique states for monolayer $DyVO_4$ identified using RomeoDFT (see Section S6).

**Lattice vectors:** Cartesian components (in [Å]) of the lattice vectors for $DyVO_4$.

| | $x$ | $y$ | $z$ |
|---|---|---|---|
| $\boldsymbol{a_1}$ | 0.0000 | 6.1252 | 0.0000 |
| $\boldsymbol{a_2}$ | −7.2483 | 0.0000 | 0.0000 |
| $\boldsymbol{a_3}$ | 0.0000 | 0.0000 | 17.1044 |

**Atomic positions:** Fractional coordinates, Hubbard $U$ (in eV) and magnetic moments (in $\mu_B$, computed from orbital occupations $m_o$ or integration spheres $m_i$) of each atom of monolayer $DyVO_4$.

| atom | $x$ | $y$ | $z$ | $U$ | $m_o$ | $m_i$ |
|---|---|---|---|---|---|---|
| Dy | 0.14 | 0.75 | −0.50 | 4.60 | 4.94 | 4.91 |
| Dy | −0.14 | 0.25 | −0.50 | 4.60 | −4.94 | −4.91 |
| V | −0.38 | 0.75 | −0.50 | 4.54 | 0.00 | 0.00 |
| V | 0.38 | 0.25 | −0.50 | 4.54 | 0.01 | 0.01 |
| O | 0.44 | 0.75 | −0.43 | 0.0 | – | 0.00 |
| O | −0.44 | 0.25 | −0.43 | 0.0 | – | 0.00 |
| O | 0.44 | 0.75 | −0.57 | 0.0 | – | 0.00 |
| O | −0.44 | 0.25 | −0.57 | 0.0 | – | 0.00 |
| O | −0.19 | 0.57 | −0.50 | 0.0 | – | 0.00 |
| O | 0.19 | 0.07 | −0.50 | 0.0 | – | 0.00 |
| O | 0.19 | 0.43 | −0.50 | 0.0 | – | 0.00 |
| O | −0.19 | 0.93 | −0.50 | 0.0 | – | 0.00 |

## $ErCl_3$ (FM)

Band gap: 1.94 eV
Total magnetization: $-6.0\ \mu_B/cell$
Absolute magnetization: $6.07\ \mu_B/cell$
MC2D entry: https://mc2d.materialscloud.org/#/details/mc2d-2608

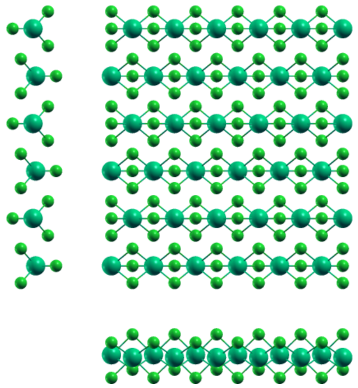

**Geometry:** Views of $ErCl_3$ as seen from the $x$ axis (left), the $y$ axis (bottom), and the $z$ axis (center).

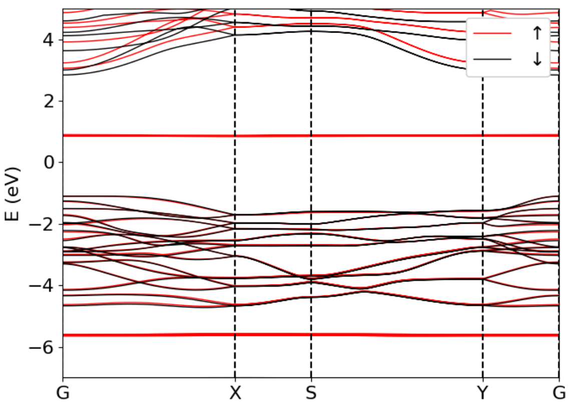


**Electronic bandstructure:** Spin-resolved energy bands of monolayer $ErCl_3$ along a high-symmetry path.

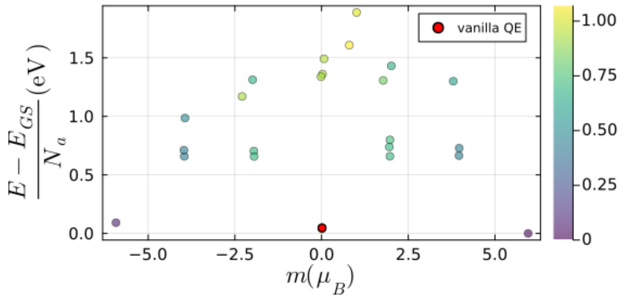


**Unique states:** Representation of 25 self-consistent unique states for monolayer $ErCl_3$ identified using RomeoDFT (see Section S6).

**Lattice vectors:** Cartesian components (in [Å]) of the lattice vectors for $ErCl_3$.

| | $x$ | $y$ | $z$ |
|---|---|---|---|
| $\boldsymbol{a_1}$ | 3.7914 | 0.0000 | 0.0000 |
| $\boldsymbol{a_2}$ | 0.0000 | 8.5515 | 0.0000 |
| $\boldsymbol{a_3}$ | 0.0000 | 0.0000 | 19.7778 |

**Atomic positions:** Fractional coordinates, Hubbard $U$ (in eV) and magnetic moments (in $\mu_B$, computed from orbital occupations $m_o$ or integration spheres $m_i$) of each atom of monolayer $ErCl_3$.

| atom | $x$ | $y$ | $z$ | $U$ | $m_o$ | $m_i$ |
|---|---|---|---|---|---|---|
| Er | 0.75 | 0.75 | 0.01 | 5.01 | −2.97 | −2.99 |
| Er | 0.25 | 0.25 | 0.99 | 5.01 | −2.97 | −2.99 |
| Cl | 0.75 | 0.43 | 0.06 | 0.0 | – | 0.00 |
| Cl | 0.75 | 0.07 | 0.06 | 0.0 | – | 0.00 |
| Cl | 0.25 | 0.93 | 0.94 | 0.0 | – | 0.00 |
| Cl | 0.25 | 0.57 | 0.94 | 0.0 | – | 0.00 |
| Cl | 0.75 | 0.25 | 0.90 | 0.0 | – | 0.00 |
| Cl | 0.25 | 0.75 | 0.10 | 0.0 | – | 0.00 |

## ErHCl (AFM)

Band gap: 0.01 eV
Total magnetization: $-0.0\ \mu_B/cell$
Absolute magnetization: $6.07\ \mu_B/cell$
MC2D entry: https://mc2d.materialscloud.org/#/details/mc2d-105

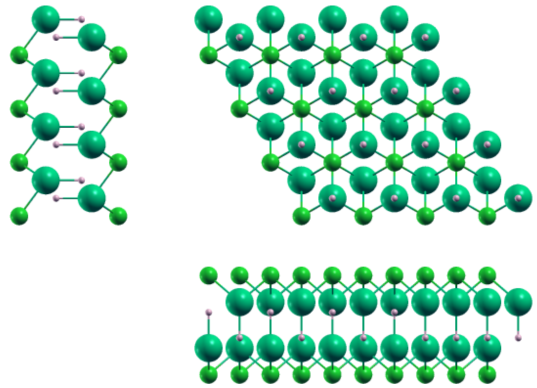

**Geometry:** Views of ErHCl as seen from the $x$ axis (left), the $y$ axis (bottom), and the $z$ axis (center).

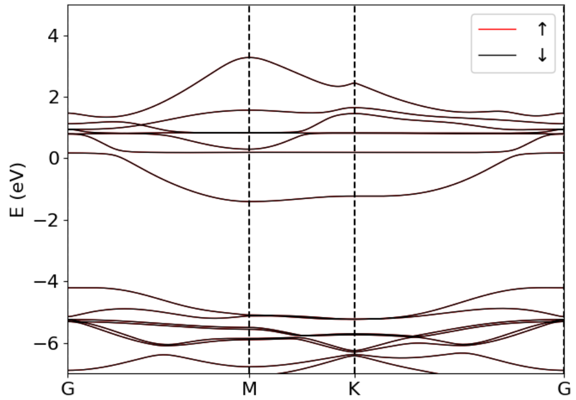


**Electronic bandstructure:** Spin-resolved energy bands of monolayer ErHCl along a high-symmetry path.

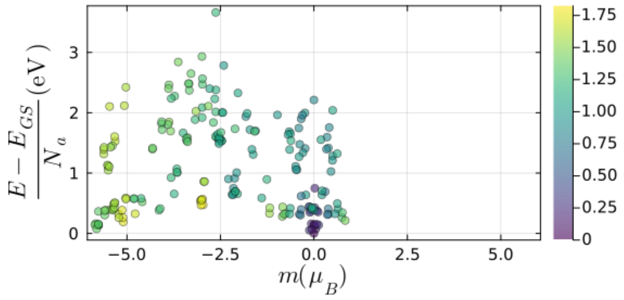


**Unique states:** Representation of 205 self-consistent unique states for monolayer ErHCl identified using RomeoDFT (see Section S6).

**Lattice vectors:** Cartesian components (in [Å]) of the lattice vectors for ErHCl.

| | $x$ | $y$ | $z$ |
|---|---|---|---|
| $\boldsymbol{a_1}$ | 3.8275 | 0.0000 | 0.0000 |
| $\boldsymbol{a_2}$ | −1.9138 | 3.3147 | 0.0000 |
| $\boldsymbol{a_3}$ | 0.0000 | 0.0000 | 26.1107 |

**Atomic positions:** Fractional coordinates, Hubbard $U$ (in eV) and magnetic moments (in $\mu_B$, computed from orbital occupations $m_o$ or integration spheres $m_i$) of each atom of monolayer ErHCl.

| atom | $x$ | $y$ | $z$ | $U$ | $m_o$ | $m_i$ |
|---|---|---|---|---|---|---|
| Er | 0.67 | 0.33 | 0.45 | 7.11 | 2.90 | 2.90 |
| Er | 0.33 | 0.67 | 0.55 | 7.11 | −2.90 | −2.90 |
| H | 0.67 | 0.33 | 0.53 | 0.0 | – | 0.00 |
| Cl | 0.0 | 0.0 | 0.38 | 0.0 | – | 0.00 |
| Cl | 0.0 | 0.0 | 0.62 | 0.0 | – | 0.00 |
| H | 0.33 | 0.67 | 0.47 | 0.0 | – | 0.00 |

## $ErI_3$ (AFM)

Band gap: 1.44 eV
Total magnetization: $-0.0\ \mu_B/cell$
Absolute magnetization: 6.09 $\mu_B/cell$
MC2D entry: https://mc2d.materialscloud.org/#/details/mc2d-2297

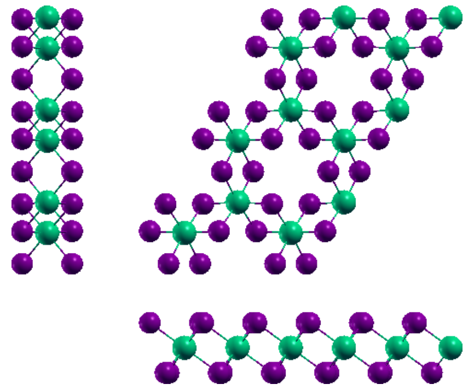

**Geometry:** Views of $ErI_3$ as seen from the $x$ axis (left), the $y$ axis (bottom), and the $z$ axis (center).

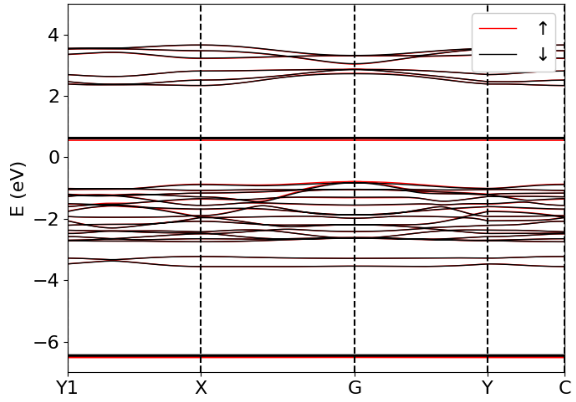


**Electronic bandstructure:** Spin-resolved energy bands of monolayer $ErI_3$ along a high-symmetry path.

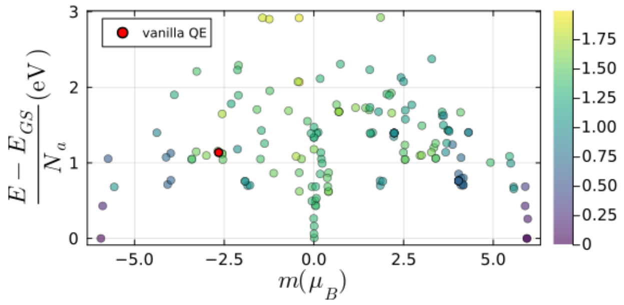


**Unique states:** Representation of 145 self-consistent unique states for monolayer $ErI_3$ identified using RomeoDFT (see Section S6).

**Lattice vectors:** Cartesian components (in [Å]) of the lattice vectors for $ErI_3$.

| | $x$ | $y$ | $z$ |
|---|---|---|---|
| $\boldsymbol{a_1}$ | 7.6129 | 0.0000 | 0.0000 |
| $\boldsymbol{a_2}$ | 3.8065 | 6.5930 | 0.0000 |
| $\boldsymbol{a_3}$ | 0.0000 | 0.0000 | 19.0193 |

**Atomic positions:** Fractional coordinates, Hubbard $U$ (in eV) and magnetic moments (in $\mu_B$, computed from orbital occupations $m_o$ or integration spheres $m_i$) of each atom of monolayer $ErI_3$.

| atom | $x$ | $y$ | $z$ | $U$ | $m_o$ | $m_i$ |
|---|---|---|---|---|---|---|
| Er | −0.33 | −0.33 | 0.0 | 5.66 | −2.97 | −3.00 |
| Er | 0.33 | −0.67 | 0.0 | 5.66 | 2.97 | 3.00 |
| I | 0.65 | −1.00 | −0.09 | 0.0 | – | 0.0 |
| I | 0.0 | −0.35 | 0.09 | 0.0 | – | 0.0 |
| I | 0.65 | −0.65 | 0.09 | 0.0 | – | 0.0 |
| I | 0.35 | −1.00 | 0.09 | 0.0 | – | 0.0 |
| I | 0.0 | −0.65 | −0.09 | 0.0 | – | 0.0 |
| I | 0.35 | −0.35 | −0.09 | 0.0 | – | 0.0 |

## ErSBr (AFM)

Band gap: 1.66 eV
Total magnetization: 0.0 $\mu_B/cell$
Absolute magnetization: 6.04 $\mu_B/cell$
MC2D entry: https://mc2d.materialscloud.org/#/details/mc2d-2576

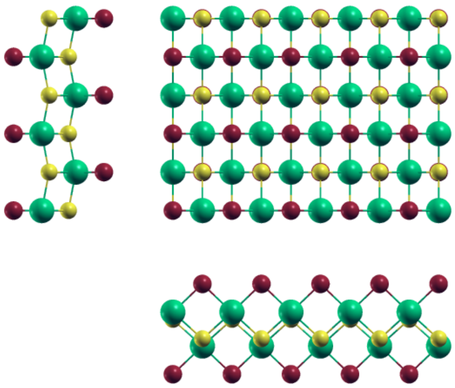

**Geometry:** Views of ErSBr as seen from the $x$ axis (left), the $y$ axis (bottom), and the $z$ axis (center).

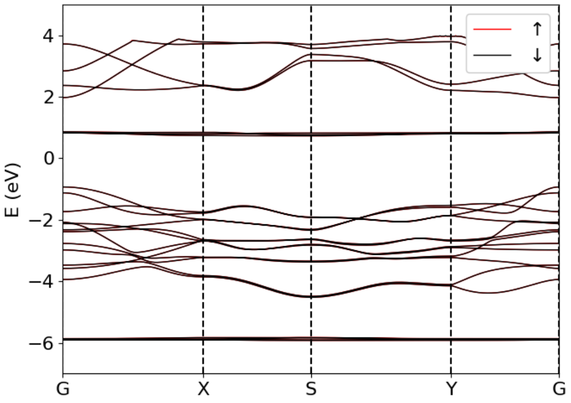


**Electronic bandstructure:** Spin-resolved energy bands of monolayer ErSBr along a high-symmetry path.

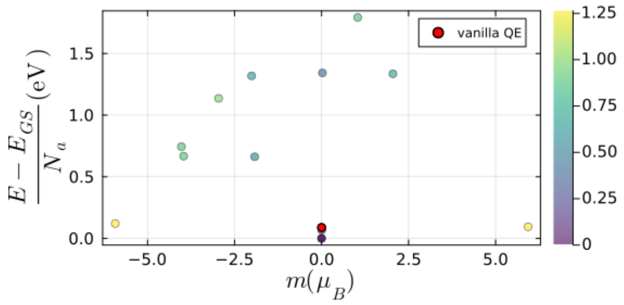


**Unique states:** Representation of 14 self-consistent unique states for monolayer ErSBr identified using RomeoDFT (see Section S6).

**Lattice vectors:** Cartesian components (in [Å]) of the lattice vectors for ErSBr.

| | $x$ | $y$ | $z$ |
|---|---|---|---|
| $a_1$ | 4.0638 | 0.0000 | 0.0000 |
| $a_2$ | 0.0000 | 5.2900 | 0.0000 |
| $a_3$ | 0.0000 | 0.0000 | 24.1907 |

**Atomic positions:** Fractional coordinates, Hubbard $U$ (in eV) and magnetic moments (in $\mu_B$, computed from orbital occupations $m_o$ or integration spheres $m_i$) of each atom of monolayer ErSBr.

| atom | $x$ | $y$ | $z$ | $U$ | $m_o$ | $m_i$ |
|---|---|---|---|---|---|---|
| Er | 0.25 | −0.25 | −0.05 | 5.26 | 2.96 | 2.98 |
| Er | −0.25 | −0.75 | 0.05 | 5.26 | −2.96 | −2.98 |
| S | 0.25 | −0.75 | −0.03 | 0.0 | – | 0.00 |
| Br | −0.25 | −0.25 | −0.13 | 0.0 | – | 0.00 |
| S | −0.25 | −0.25 | 0.03 | 0.0 | – | 0.00 |
| Br | 0.25 | −0.75 | 0.13 | 0.0 | – | 0.00 |

## ErSCl (AFM)

Band gap: 1.71 eV
Total magnetization: 0.0 $\mu_B/cell$
Absolute magnetization: 6.04 $\mu_B/cell$
MC2D entry: https://mc2d.materialscloud.org/#/details/mc2d-106

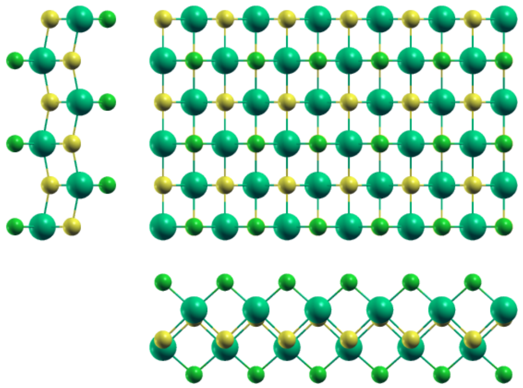

**Geometry:** Views of ErSCl as seen from the $x$ axis (left), the $y$ axis (bottom), and the $z$ axis (center).

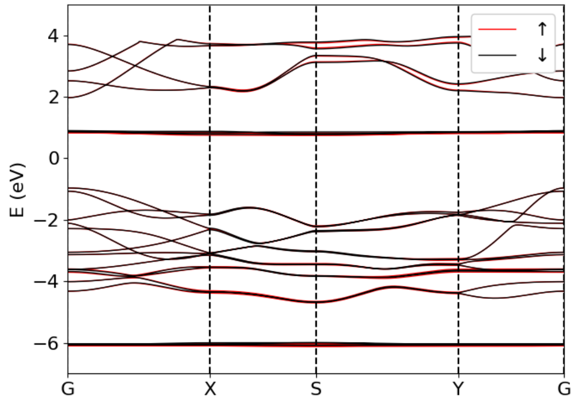


**Electronic bandstructure:** Spin-resolved energy bands of monolayer ErSCl along a high-symmetry path.

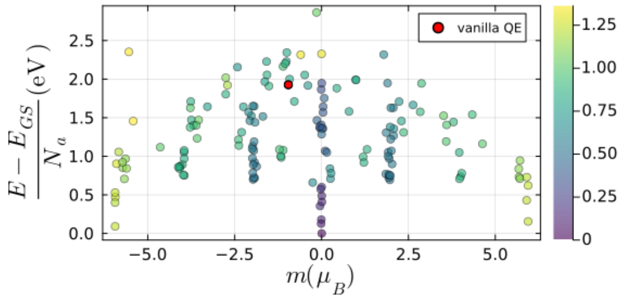


**Unique states:** Representation of 158 self-consistent unique states for monolayer ErSCl identified using RomeoDFT (see Section S6).

**Lattice vectors:** Cartesian components (in [Å]) of the lattice vectors for ErSCl.

| | $x$ | $y$ | $z$ |
|---|---|---|---|
| $\boldsymbol{a_1}$ | 3.9699 | 0.0000 | 0.0000 |
| $\boldsymbol{a_2}$ | 0.0000 | 5.3261 | 0.0000 |
| $\boldsymbol{a_3}$ | 0.0000 | 0.0000 | 25.8063 |

**Atomic positions:** Fractional coordinates, Hubbard $U$ (in eV) and magnetic moments (in $\mu_B$, computed from orbital occupations $m_o$ or integration spheres $m_i$) of each atom of monolayer ErSCl.

| atom | $x$ | $y$ | $z$ | $U$ | $m_o$ | $m_i$ |
|---|---|---|---|---|---|---|
| Er | 0.50 | 0.50 | 0.45 | 5.46 | −2.97 | −2.98 |
| Er | 0.0 | 0.0 | 0.55 | 5.46 | 2.97 | 2.98 |
| S | 0.50 | 0.0 | 0.47 | 0.0 | – | 0.00 |
| S | 0.0 | 0.50 | 0.53 | 0.0 | – | 0.00 |
| Cl | 0.50 | 0.0 | 0.61 | 0.0 | – | 0.00 |
| Cl | 0.0 | 0.50 | 0.38 | 0.0 | – | 0.00 |

## ErSeBr (AFM)

Band gap: 1.78 eV
Total magnetization: $-0.0\ \mu_B/cell$
Absolute magnetization: $6.05\ \mu_B/cell$
MC2D entry: https://mc2d.materialscloud.org/#/details/mc2d-1821

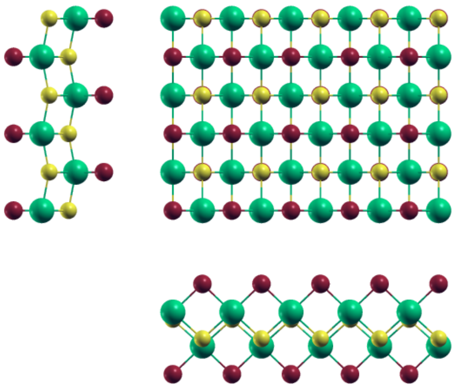

**Geometry:** Views of ErSeBr as seen from the $x$ axis (left), the $y$ axis (bottom), and the $z$ axis (center).

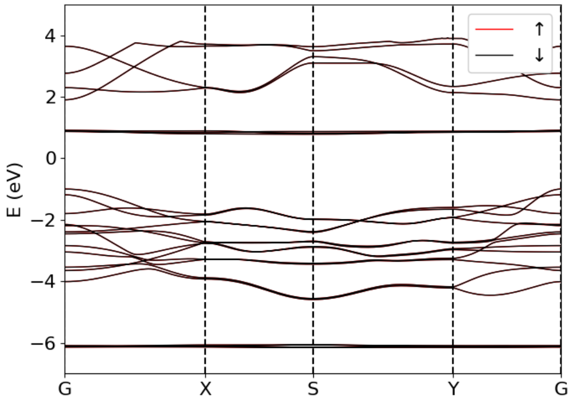


**Electronic bandstructure:** Spin-resolved energy bands of monolayer ErSeBr along a high-symmetry path.

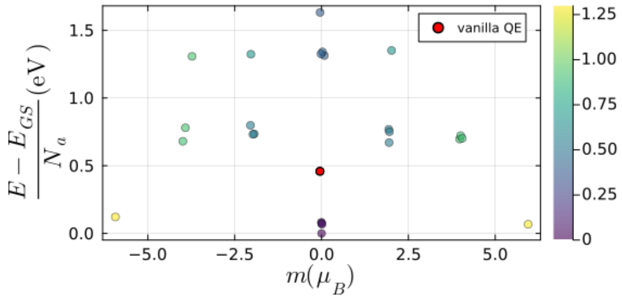


**Unique states:** Representation of 25 self-consistent unique states for monolayer ErSeBr identified using RomeoDFT (see Section S6).

**Lattice vectors:** Cartesian components (in [Å]) of the lattice vectors for ErSeBr.

| | $x$ | $y$ | $z$ |
|---|---|---|---|
| $\boldsymbol{a_1}$ | 4.0638 | 0.0000 | 0.0000 |
| $\boldsymbol{a_2}$ | 0.0000 | 5.2900 | 0.0000 |
| $\boldsymbol{a_3}$ | 0.0000 | 0.0000 | 24.1907 |

**Atomic positions:** Fractional coordinates, Hubbard $U$ (in eV) and magnetic moments (in $\mu_B$, computed from orbital occupations $m_o$ or integration spheres $m_i$) of each atom of monolayer ErSeBr.

| atom | $x$ | $y$ | $z$ | $U$ | $m_o$ | $m_i$ |
|---|---|---|---|---|---|---|
| Er | 0.25 | −0.25 | −0.05 | 5.55 | 2.97 | 2.98 |
| Er | −0.25 | −0.75 | 0.05 | 5.55 | −2.97 | −2.98 |
| S | 0.25 | −0.75 | −0.03 | 0.0 | – | 0.00 |
| Br | −0.25 | −0.25 | −0.13 | 0.0 | – | 0.00 |
| S | −0.25 | −0.25 | 0.03 | 0.0 | – | 0.00 |
| Br | 0.25 | −0.75 | 0.13 | 0.0 | – | 0.00 |

## ErSeI (AFM)

Band gap: 1.28 eV
Total magnetization: 0.0 $\mu_B/cell$
Absolute magnetization: 6.1 $\mu_B/cell$
MC2D entry: https://mc2d.materialscloud.org/#/details/mc2d-137

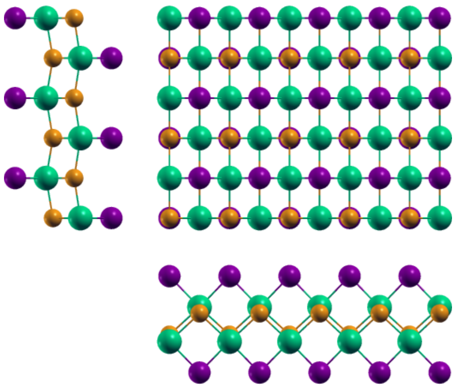

**Geometry:** Views of ErSeI as seen from the $x$ axis (left), the $y$ axis (bottom), and the $z$ axis (center).

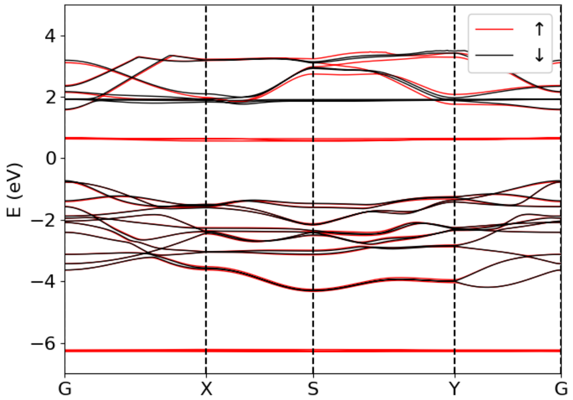


**Electronic bandstructure:** Spin-resolved energy bands of monolayer ErSeI along a high-symmetry path.

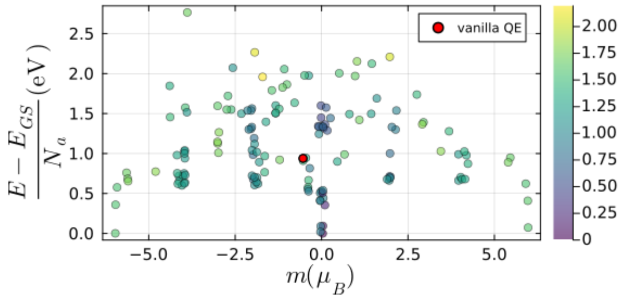


**Unique states:** Representation of 144 self-consistent unique states for monolayer ErSeI identified using RomeoDFT (see Section S6).

**Lattice vectors:** Cartesian components (in [Å]) of the lattice vectors for ErSeI.

| | $x$ | $y$ | $z$ |
|---|---|---|---|
| $\boldsymbol{a_1}$ | 4.2143 | 0.0000 | 0.0000 |
| $\boldsymbol{a_2}$ | 0.0000 | 5.6058 | 0.0000 |
| $\boldsymbol{a_3}$ | 0.0000 | 0.0000 | 26.5088 |

**Atomic positions:** Fractional coordinates, Hubbard $U$ (in eV) and magnetic moments (in $\mu_B$, computed from orbital occupations $m_o$ or integration spheres $m_i$) of each atom of monolayer ErSeI.

| atom | $x$ | $y$ | $z$ | $U$ | $m_o$ | $m_i$ |
|---|---|---|---|---|---|---|
| Er | 0.0 | 0.50 | 0.55 | 8.53 | 2.99 | 3.01 |
| Er | 0.50 | 0.0 | 0.45 | 5.47 | −2.96 | −2.99 |
| Se | 0.50 | 0.50 | 0.47 | 0.0 | – | −0.01 |
| Se | 0.0 | 0.0 | 0.53 | 0.0 | – | −0.01 |
| I | 0.0 | 0.0 | 0.37 | 0.0 | – | 0.00 |
| I | 0.50 | 0.50 | 0.63 | 0.0 | – | −0.01 |

## $EuBr_3$ (FM)

Band gap: 0.37 eV
Total magnetization: 12.0 $\mu_B/cell$
Absolute magnetization: 12.58 $\mu_B/cell$
MC2D entry: https://mc2d.materialscloud.org/#/details/mc2d-2550

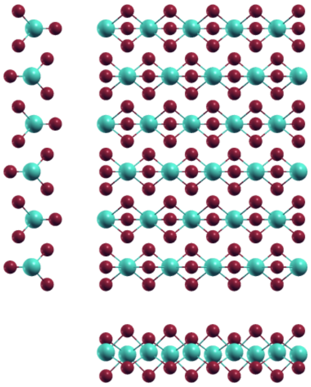

**Geometry:** Views of $EuBr_3$ as seen from the $x$ axis (left), the $y$ axis (bottom), and the $z$ axis (center).

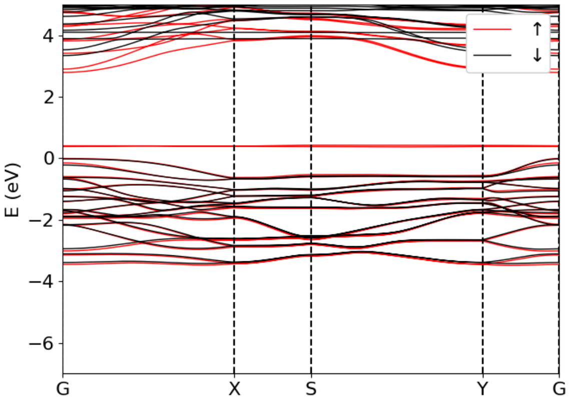


**Electronic bandstructure:** Spin-resolved energy bands of monolayer $EuBr_3$ along a high-symmetry path.

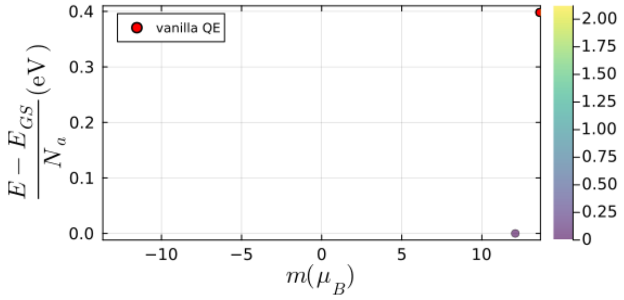


**Unique states:** Representation of 3 self-consistent unique states for monolayer $EuBr_3$ identified using RomeoDFT (see Section S6).

**Lattice vectors:** Cartesian components (in [Å]) of the lattice vectors for $EuBr_3$.

| | $x$ | $y$ | $z$ |
|---|---|---|---|
| $\boldsymbol{a_1}$ | 4.1114 | 0.0000 | 0.0000 |
| $\boldsymbol{a_2}$ | 0.0000 | 9.1756 | 0.0000 |
| $\boldsymbol{a_3}$ | 0.0000 | 0.0000 | 20.4776 |

**Atomic positions:** Fractional coordinates, Hubbard $U$ (in eV) and magnetic moments (in $\mu_B$, computed from orbital occupations $m_o$ or integration spheres $m_i$) of each atom of monolayer $EuBr_3$.

| atom | $x$ | $y$ | $z$ | $U$ | $m_o$ | $m_i$ |
|---|---|---|---|---|---|---|
| Eu | 0.25 | −0.25 | −0.01 | 8.14 | 6.03 | 6.03 |
| Eu | −0.25 | −0.75 | 0.01 | 8.14 | 6.06 | 6.05 |
| Br | −0.25 | −0.07 | 0.07 | 0.0 | – | −0.04 |
| Br | −0.25 | −0.43 | 0.07 | 0.0 | – | −0.04 |
| Br | 0.25 | −0.57 | −0.07 | 0.0 | – | −0.04 |
| Br | 0.25 | −0.93 | −0.07 | 0.0 | – | −0.04 |
| Br | −0.25 | −0.25 | −0.11 | 0.0 | – | −0.04 |
| Br | 0.25 | −0.75 | 0.11 | 0.0 | – | −0.05 |

## EuHI (FM)

Band gap: 0.48 eV
Total magnetization: $-14.0\ \mu_B/cell$
Absolute magnetization: $14.22\ \mu_B/cell$
MC2D entry: https://mc2d.materialscloud.org/#/details/mc2d-2048

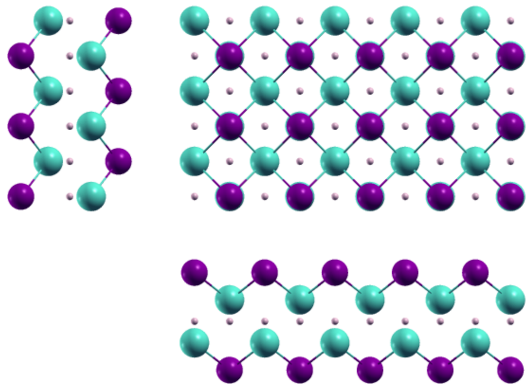

**Geometry:** Views of EuHI as seen from the $x$ axis (left), the $y$ axis (bottom), and the $z$ axis (center).

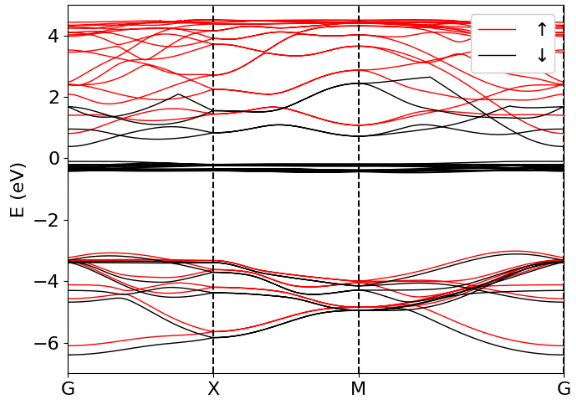


**Electronic bandstructure:** Spin-resolved energy bands of monolayer EuHI along a high-symmetry path.

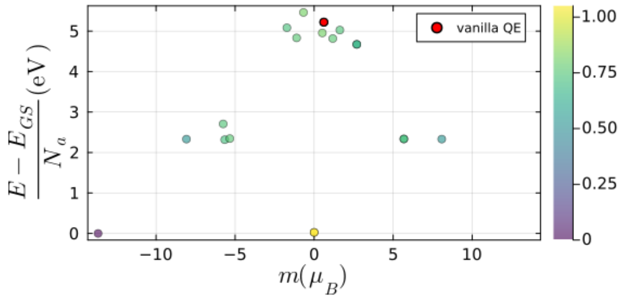


**Unique states:** Representation of 20 self-consistent unique states for monolayer EuHI identified using RomeoDFT (see Section S6).

**Lattice vectors:** Cartesian components (in [Å]) of the lattice vectors for EuHI.

| | $x$ | $y$ | $z$ |
|---|---|---|---|
| $\boldsymbol{a_1}$ | 4.1401 | 0.0000 | 0.0000 |
| $\boldsymbol{a_2}$ | 0.0000 | 4.1401 | 0.0000 |
| $\boldsymbol{a_3}$ | 0.0000 | 0.0000 | 22.6809 |

**Atomic positions:** Fractional coordinates, Hubbard $U$ (in eV) and magnetic moments (in $\mu_B$, computed from orbital occupations $m_o$ or integration spheres $m_i$) of each atom of monolayer EuHI.

| atom | $x$ | $y$ | $z$ | $U$ | $m_o$ | $m_i$ |
|---|---|---|---|---|---|---|
| Eu | 0.25 | −0.25 | 0.06 | 0.00 | −6.83 | −6.59 |
| Eu | −0.25 | −0.75 | −0.06 | 0.00 | −6.83 | −6.59 |
| I | −0.25 | −0.75 | 0.13 | 0.0 | – | 0.03 |
| H | −0.25 | −0.25 | 0.0 | 0.0 | – | 0.00 |
| H | 0.25 | −0.75 | 0.0 | 0.0 | – | 0.00 |
| I | 0.25 | −0.25 | −0.13 | 0.0 | – | 0.03 |

## EuIF (FM)

Band gap: 0.74 eV
Total magnetization: $-14.0\ \mu_B/cell$
Absolute magnetization: $14.17\ \mu_B/cell$
MC2D entry: https://mc2d.materialscloud.org/#/details/mc2d-2428

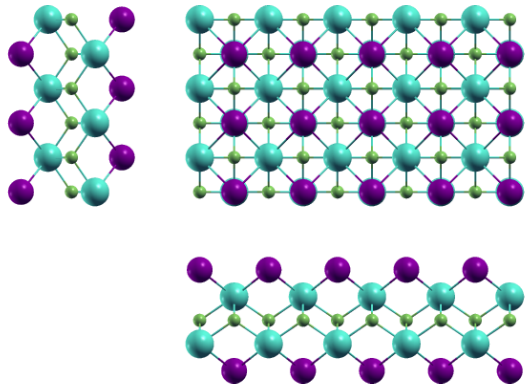

**Geometry:** Views of EuIF as seen from the $x$ axis (left), the $y$ axis (bottom), and the $z$ axis (center).

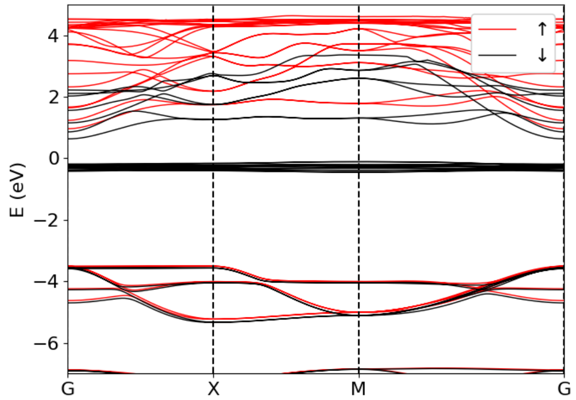


**Electronic bandstructure:** Spin-resolved energy bands of monolayer EuIF along a high-symmetry path.

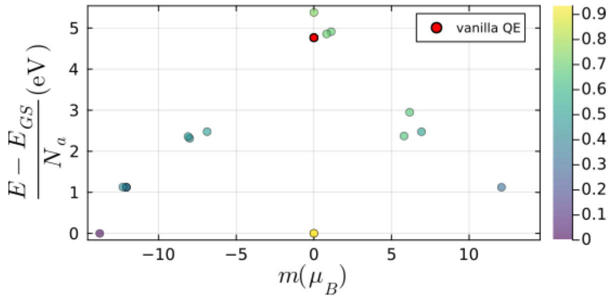


**Unique states:** Representation of 13 self-consistent unique states for monolayer EuIF identified using RomeoDFT (see Section S6).

**Lattice vectors:** Cartesian components (in [Å]) of the lattice vectors for EuIF.

| | $x$ | $y$ | $z$ |
|---|---|---|---|
| $\boldsymbol{a}_1$ | 4.1835 | 0.0000 | 0.0000 |
| $\boldsymbol{a}_2$ | 0.0000 | 4.1835 | 0.0000 |
| $\boldsymbol{a}_3$ | 0.0000 | 0.0000 | 23.8745 |

**Atomic positions:** Fractional coordinates, Hubbard $U$ (in eV) and magnetic moments (in $\mu_B$, computed from orbital occupations $m_\text{o}$ or integration spheres $m_\text{i}$) of each atom of monolayer EuIF.

| atom | $x$ | $y$ | $z$ | $U$ | $m_\text{o}$ | $m_\text{i}$ |
|---|---|---|---|---|---|---|
| Eu | 0.25 | −0.25 | 0.06 | 0.00 | −6.90 | −6.70 |
| Eu | −0.25 | −0.75 | −0.06 | 0.00 | −6.90 | −6.70 |
| I | −0.25 | −0.75 | 0.13 | 0.0 | – | 0.02 |
| I | 0.25 | −0.25 | −0.13 | 0.0 | – | 0.02 |
| F | −0.25 | −0.25 | 0.0 | 0.0 | – | 0.01 |
| F | 0.25 | −0.75 | 0.0 | 0.0 | – | 0.01 |

# EuOBr (AFM)

Band gap: 0.89 eV
Total magnetization: −0.0 $\mu_B/cell$
Absolute magnetization: 12.32 $\mu_B/cell$
MC2D entry: https://mc2d.materialscloud.org/#/details/mc2d-48

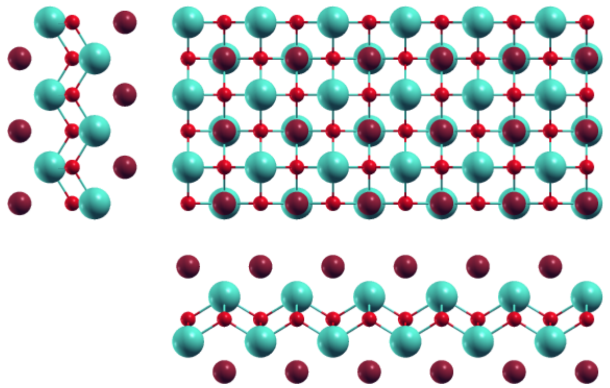

**Geometry:** Views of EuOBr as seen from the $x$ axis (left), the $y$ axis (bottom), and the $z$ axis (center).

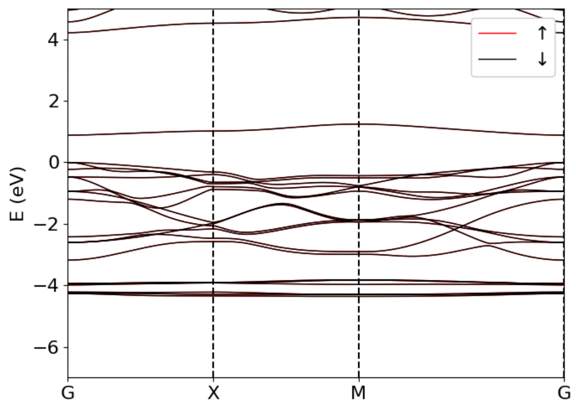


**Electronic bandstructure:** Spin-resolved energy bands of monolayer EuOBr along a high-symmetry path.

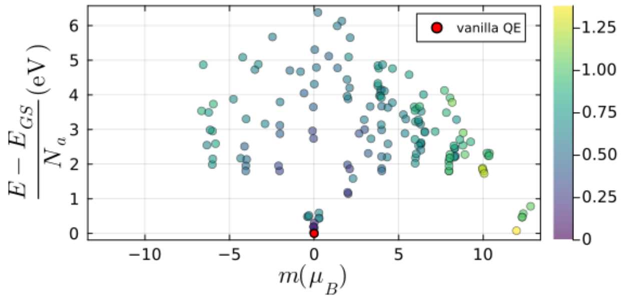


**Unique states:** Representation of 155 self-consistent unique states for monolayer EuOBr identified using RomeoDFT (see Section S6).

**Lattice vectors:** Cartesian components (in [Å]) of the lattice vectors for EuOBr.

| | $x$ | $y$ | $z$ |
|---|---|---|---|
| $\boldsymbol{a_1}$ | 3.9324 | 0.0000 | 0.0000 |
| $\boldsymbol{a_2}$ | 0.0000 | 3.9324 | 0.0000 |
| $\boldsymbol{a_3}$ | 0.0000 | 0.0000 | 25.4259 |

**Atomic positions:** Fractional coordinates, Hubbard $U$ (in eV) and magnetic moments (in $\mu_B$, computed from orbital occupations $m_o$ or integration spheres $m_i$) of each atom of monolayer EuOBr.

| atom | $x$ | $y$ | $z$ | $U$ | $m_o$ | $m_i$ |
|---|---|---|---|---|---|---|
| Eu | 0.0 | 0.50 | 0.55 | 5.00 | 6.01 | 5.90 |
| Eu | 0.50 | 0.0 | 0.45 | 5.00 | −6.01 | −5.90 |
| Br | 0.50 | 0.0 | 0.61 | 0.0 | – | −0.02 |
| Br | 0.0 | 0.50 | 0.39 | 0.0 | – | 0.02 |
| O | 0.50 | 0.50 | 0.50 | 0.0 | – | 0.00 |
| O | 0.0 | 0.0 | 0.50 | 0.0 | – | 0.00 |

## $EuOBr_2$ (FM)

Band gap: 0.0 eV
Total magnetization: 9.78 $\mu_B/cell$
Absolute magnetization: 16.31 $\mu_B/cell$
MC2D entry: https://mc2d.materialscloud.org/#/details/mc2d-1781

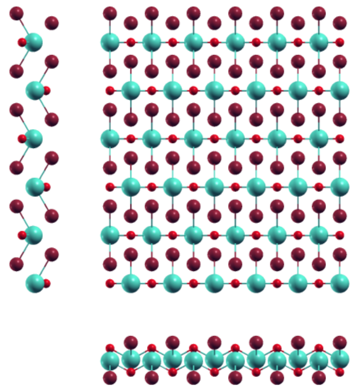

**Geometry:** Views of $EuOBr_2$ as seen from the $x$ axis (left), the $y$ axis (bottom), and the $z$ axis (center).

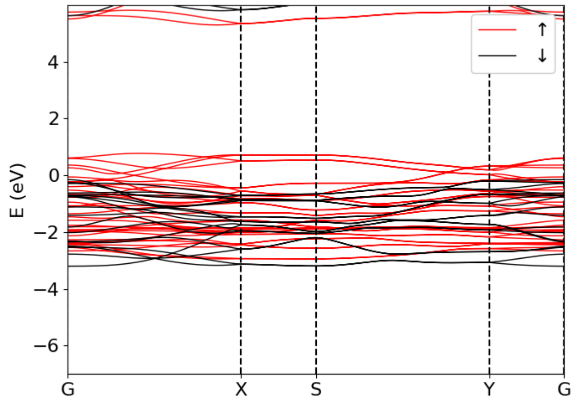


**Electronic bandstructure:** Spin-resolved energy bands of monolayer $EuOBr_2$ along a high-symmetry path.

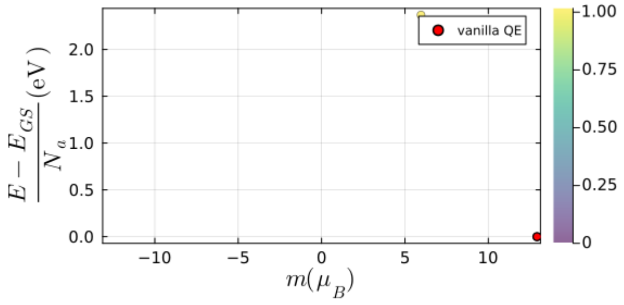


**Unique states:** Representation of 3 self-consistent unique states for monolayer $EuOBr_2$ identified using RomeoDFT (see Section S6).

**Lattice vectors:** Cartesian components (in [Å]) of the lattice vectors for $EuOBr_2$.

| | $x$ | $y$ | $z$ |
|---|---|---|---|
| $\boldsymbol{a_1}$ | −3.8299 | 0.0000 | 0.0000 |
| $\boldsymbol{a_2}$ | 0.0000 | −8.8340 | 0.0000 |
| $\boldsymbol{a_3}$ | 0.0000 | 0.0000 | 18.3368 |

**Atomic positions:** Fractional coordinates, Hubbard $U$ (in eV) and magnetic moments (in $\mu_B$, computed from orbital occupations $m_o$ or integration spheres $m_i$) of each atom of monolayer $EuOBr_2$.

| atom | $x$ | $y$ | $z$ | $U$ | $m_o$ | $m_i$ |
|---|---|---|---|---|---|---|
| Eu | 0.75 | 0.12 | 0.00 | 6.40 | 6.46 | 6.13 |
| Eu | 0.25 | 0.62 | 0.00 | 6.40 | 6.46 | 6.13 |
| Br | 0.75 | 0.82 | −0.09 | 0.0 | – | −0.33 |
| Br | 0.25 | 0.32 | 0.09 | 0.0 | – | −0.33 |
| Br | 0.75 | 0.42 | −0.09 | 0.0 | – | −0.33 |
| Br | 0.25 | 0.92 | 0.09 | 0.0 | – | −0.33 |
| O | 0.75 | 0.62 | 0.06 | 0.0 | – | −0.82 |
| O | 0.25 | 0.12 | −0.06 | 0.0 | – | −0.82 |

## EuOI (FM)

Band gap: 0.0 eV
Total magnetization: 11.91 $\mu_B/cell$
Absolute magnetization: 13.34 $\mu_B/cell$
MC2D entry: https://mc2d.materialscloud.org/#/details/mc2d-138

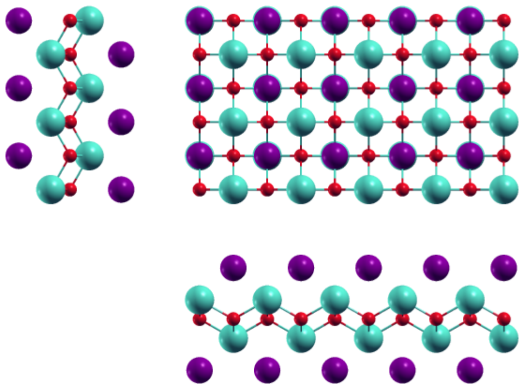

**Geometry:** Views of EuOI as seen from the $x$ axis (left), the $y$ axis (bottom), and the $z$ axis (center).

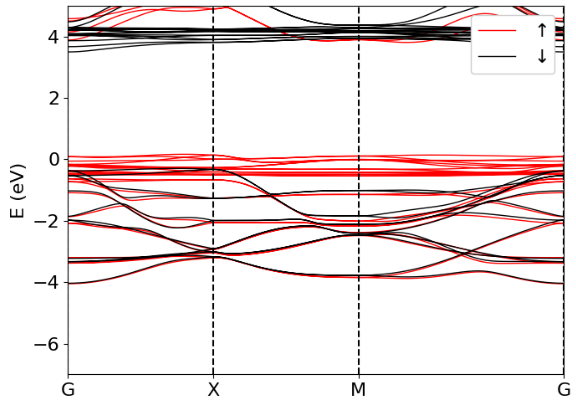


**Electronic bandstructure:** Spin-resolved energy bands of monolayer EuOI along a high-symmetry path.

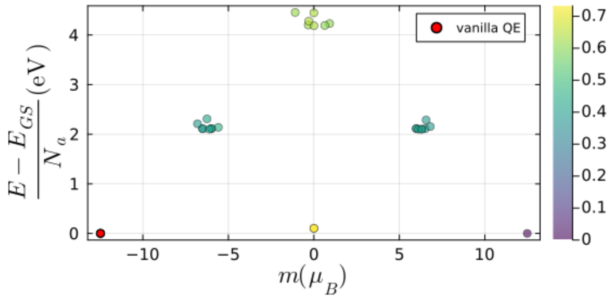


**Unique states:** Representation of 28 self-consistent unique states for monolayer EuOI identified using RomeoDFT (see Section S6).

**Lattice vectors:** Cartesian components (in [Å]) of the lattice vectors for EuOI.

| | $x$ | $y$ | $z$ |
|---|---|---|---|
| $\boldsymbol{a_1}$ | 4.0097 | 0.0000 | 0.0000 |
| $\boldsymbol{a_2}$ | 0.0000 | 4.0097 | 0.0000 |
| $\boldsymbol{a_3}$ | 0.0000 | 0.0000 | 24.1553 |

**Atomic positions:** Fractional coordinates, Hubbard $U$ (in eV) and magnetic moments (in $\mu_B$, computed from orbital occupations $m_o$ or integration spheres $m_i$) of each atom of monolayer EuOI.

| atom | $x$ | $y$ | $z$ | $U$ | $m_o$ | $m_i$ |
|---|---|---|---|---|---|---|
| Eu | −0.25 | −0.75 | 0.05 | 0.00 | 6.24 | 6.05 |
| Eu | 0.25 | −0.25 | −0.05 | 0.00 | 6.24 | 6.05 |
| I | 0.25 | −0.25 | 0.13 | 0.0 | – | −0.15 |
| I | −0.25 | −0.75 | −0.13 | 0.0 | – | −0.15 |
| O | 0.25 | −0.75 | 0.0 | 0.0 | – | −0.18 |
| O | −0.25 | −0.25 | 0.0 | 0.0 | – | −0.18 |

## $Fe_2Ga_2S_5$ (AFM)

Band gap: 1.11 eV
Total magnetization: 0.0 $\mu_B/cell$
Absolute magnetization: 7.64 $\mu_B/cell$
MC2D entry: https://mc2d.materialscloud.org/#/details/mc2d-497

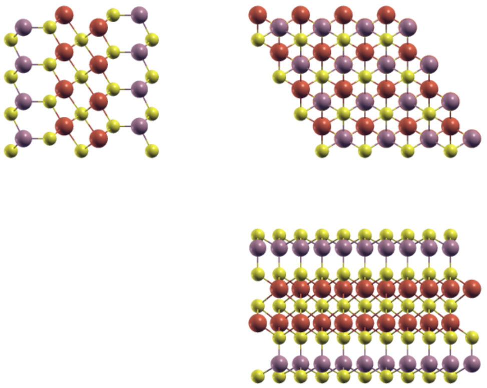

**Geometry:** Views of $Fe_2Ga_2S_5$ as seen from the $x$ axis (left), the $y$ axis (bottom), and the $z$ axis (center).

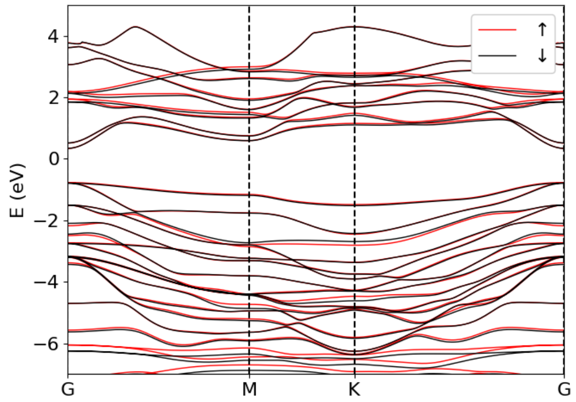


**Electronic bandstructure:** Spin-resolved energy bands of monolayer $Fe_2Ga_2S_5$ along a high-symmetry path.

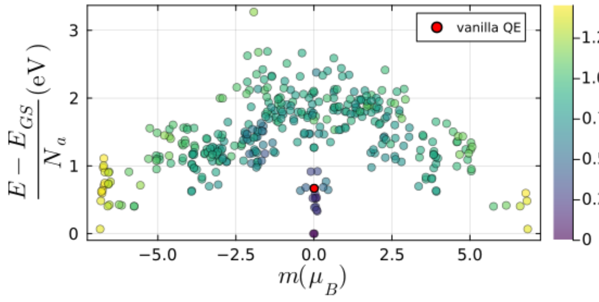


**Unique states:** Representation of 373 self-consistent unique states for monolayer $Fe_2Ga_2S_5$ identified using RomeoDFT (see Section S6).

**Lattice vectors:** Cartesian components (in [Å]) of the lattice vectors for $Fe_2Ga_2S_5$.

| | $x$ | $y$ | $z$ |
|---|---|---|---|
| $\boldsymbol{a_1}$ | 3.6241 | 0.0000 | 0.0000 |
| $\boldsymbol{a_2}$ | −1.8120 | 3.1385 | 0.0000 |
| $\boldsymbol{a_3}$ | 0.0000 | 0.0000 | 31.6709 |

**Atomic positions:** Fractional coordinates, Hubbard $U$ (in eV) and magnetic moments (in $\mu_B$, computed from orbital occupations $m_o$ or integration spheres $m_i$) of each atom of monolayer $Fe_2Ga_2S_5$.

| atom | $x$ | $y$ | $z$ | $U$ | $m_o$ | $m_i$ |
|---|---|---|---|---|---|---|
| Fe | 0.67 | 0.33 | 0.45 | 4.64 | 3.37 | 3.42 |
| Fe | 0.33 | 0.67 | 0.55 | 4.95 | −3.38 | −3.43 |
| Ga | 0.33 | 0.67 | 0.35 | 0.0 | – | 0.01 |
| Ga | 0.67 | 0.33 | 0.65 | 0.0 | – | −0.01 |
| S | 0.33 | 0.67 | 0.42 | 0.0 | – | 0.07 |
| S | 0.67 | 0.33 | 0.58 | 0.0 | – | −0.07 |
| S | 0.0 | 0.0 | 0.50 | 0.0 | – | 0.00 |
| S | 0.0 | 0.0 | 0.31 | 0.0 | – | 0.01 |
| S | 0.0 | 0.0 | 0.69 | 0.0 | – | −0.01 |

## $Fe_2O_3$ (AFM)

Band gap: 0.95 eV
Total magnetization: 0.0 $\mu_B/cell$
Absolute magnetization: 12.85 $\mu_B/cell$
MC2D entry: https://mc2d.materialscloud.org/#/details/mc2d-2337

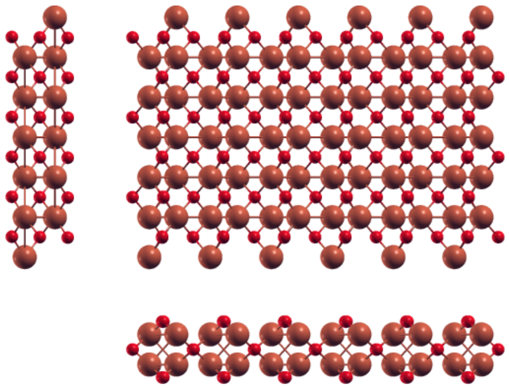

**Geometry:** Views of $Fe_2O_3$ as seen from the $x$ axis (left), the $y$ axis (bottom), and the $z$ axis (center).

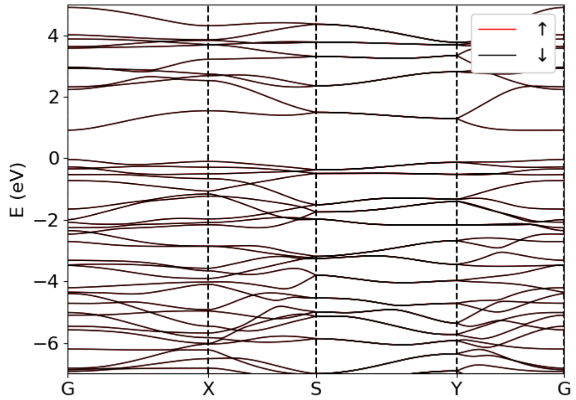


**Electronic bandstructure:** Spin-resolved energy bands of monolayer $Fe_2O_3$ along a high-symmetry path.

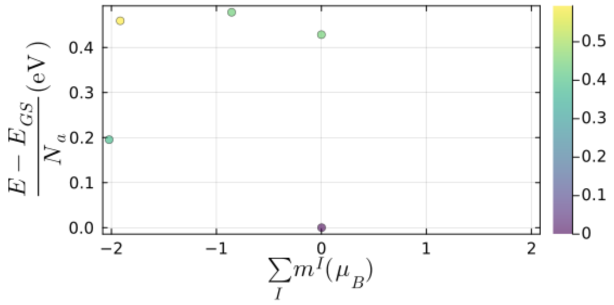

**Unique states:** Representation of 6 self-consistent unique states for monolayer $Fe_2O_3$ identified using RomeoDFT (see Section S6).

**Lattice vectors:** Cartesian components (in [Å]) of the lattice vectors for $Fe_2O_3$.

| | $x$ | $y$ | $z$ |
|---|---|---|---|
| $\boldsymbol{a_1}$ | 0.0000 | −3.9596 | 0.0000 |
| $\boldsymbol{a_2}$ | 5.1887 | 0.0000 | 0.0000 |
| $\boldsymbol{a_3}$ | 0.0000 | 0.0000 | 20.2558 |

**Atomic positions:** Fractional coordinates, Hubbard $U$ (in eV) and magnetic moments (in $\mu_B$, computed from orbital occupations $m_o$ or integration spheres $m_i$) of each atom of monolayer $Fe_2O_3$.

| atom | $x$ | $y$ | $z$ | $U$ | $m_o$ | $m_i$ |
|---|---|---|---|---|---|---|
| Fe | 0.28 | 1.00 | 0.05 | 5.11 | 2.84 | 2.55 |
| Fe | 0.72 | 0.50 | 0.05 | 5.11 | 2.84 | 2.55 |
| Fe | 0.28 | 0.50 | −0.05 | 5.11 | −2.84 | −2.55 |
| Fe | 0.72 | 0.00 | −0.05 | 5.11 | −2.84 | −2.55 |
| O | 0.51 | 0.76 | 0.09 | 0.0 | – | −0.06 |
| O | 0.49 | 0.26 | 0.09 | 0.0 | – | −0.06 |
| O | 0.51 | 0.74 | −0.09 | 0.0 | – | 0.06 |
| O | 0.49 | 0.24 | −0.09 | 0.0 | – | 0.06 |
| O | 0.00 | 0.25 | 0.0 | 0.0 | – | 0.0 |
| O | 1.00 | 0.75 | 0.0 | 0.0 | – | 0.0 |

## $Fe_3S_4$ (FM)

Band gap: 0.0 eV
Total magnetization: 2.12 $\mu_B/cell$
Absolute magnetization: 12.66 $\mu_B/cell$
MC2D entry: https://mc2d.materialscloud.org/#/details/mc2d-1457

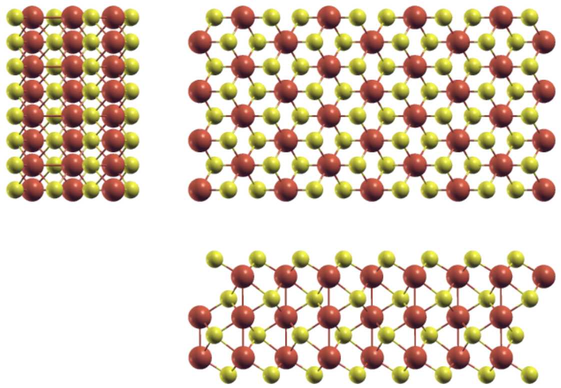

**Geometry:** Views of $Fe_3S_4$ as seen from the $x$ axis (left), the $y$ axis (bottom), and the $z$ axis (center).

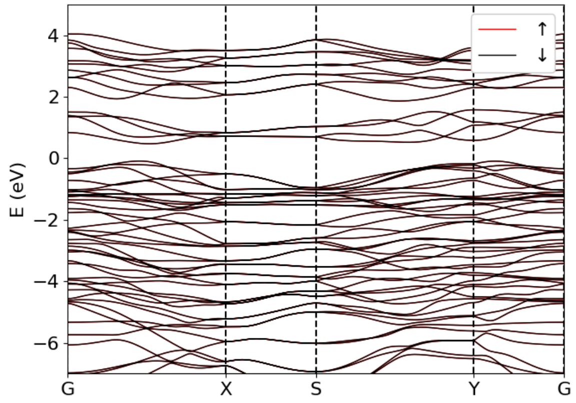


**Electronic bandstructure:** Spin-resolved energy bands of monolayer $Fe_3S_4$ along a high-symmetry path.

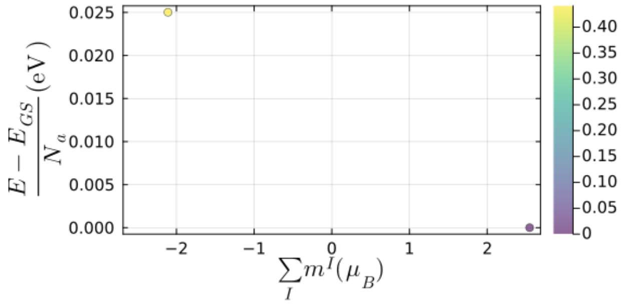


**Unique states:** Representation of 3 self-consistent unique states for monolayer $Fe_3S_4$ identified using RomeoDFT (see Section S6).

**Lattice vectors:** Cartesian components (in [Å]) of the lattice vectors for $Fe_3S_4$.

| | $x$ | $y$ | $z$ |
|---|---|---|---|
| $\boldsymbol{a_1}$ | 0.0000 | −5.7729 | 0.0000 |
| $\boldsymbol{a_2}$ | 3.3007 | 0.0000 | 0.0000 |
| $\boldsymbol{a_3}$ | 0.0000 | 0.0000 | 27.3653 |

**Atomic positions:** Fractional coordinates, Hubbard $U$ (in eV) and magnetic moments (in $\mu_B$, computed from orbital occupations $m_o$ or integration spheres $m_i$) of each atom of monolayer $Fe_3S_4$.

| atom | $x$ | $y$ | $z$ | $U$ | $m_o$ | $m_i$ |
|---|---|---|---|---|---|---|
| Fe | 1.00 | 1.00 | 0.50 | 4.96 | −1.86 | −1.84 |
| Fe | 1.00 | 1.00 | 0.60 | 4.96 | 1.90 | 1.86 |
| Fe | 1.00 | 1.00 | 0.40 | 4.96 | 2.62 | 2.62 |
| Fe | 0.50 | 0.50 | 0.50 | 4.96 | 0.25 | 0.23 |
| Fe | 0.50 | 0.50 | 0.60 | 4.96 | −1.98 | −1.95 |
| Fe | 0.50 | 0.50 | 0.40 | 4.96 | 1.62 | 1.52 |
| S | 1.17 | 0.50 | 0.36 | 0.0 | – | −0.17 |
| S | 0.67 | 1.00 | 0.36 | 0.0 | – | −0.20 |
| S | 0.33 | 1.00 | 0.64 | 0.0 | – | 0.08 |
| S | 0.83 | 0.50 | 0.64 | 0.0 | – | −0.08 |
| S | 0.67 | 1.00 | 0.54 | 0.0 | – | 0.04 |
| S | 1.17 | 0.50 | 0.54 | 0.0 | – | 0.04 |
| S | 0.83 | 0.50 | 0.46 | 0.0 | – | 0.03 |
| S | 0.33 | 1.00 | 0.46 | 0.0 | – | −0.07 |

## $FeAl_2S_4$ (FM)

Band gap: 0.0 eV
Total magnetization: 6.0 $\mu_B/cell$
Absolute magnetization: 6.45 $\mu_B/cell$
MC2D entry: https://mc2d.materialscloud.org/#/details/mc2d-600

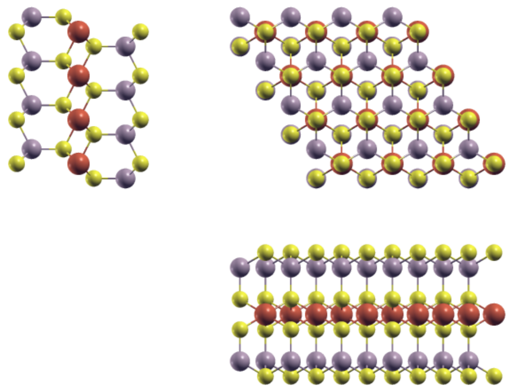

**Geometry:** Views of $FeAl_2S_4$ as seen from the $x$ axis (left), the $y$ axis (bottom), and the $z$ axis (center).

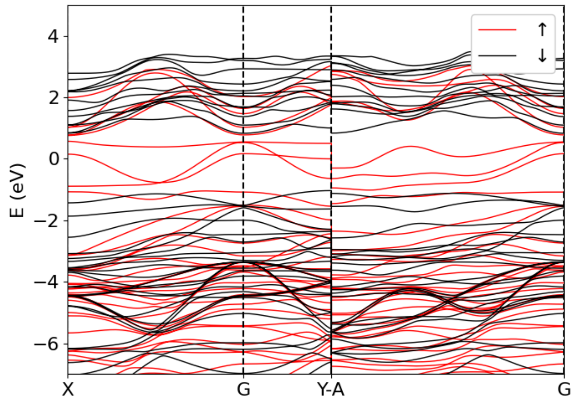


**Electronic bandstructure:** Spin-resolved energy bands of monolayer $FeAl_2S_4$ along a high-symmetry path.

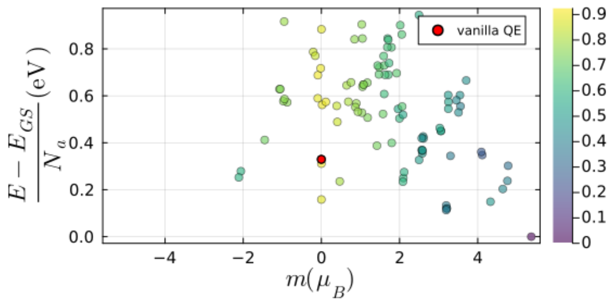


**Unique states:** Representation of 97 self-consistent unique states for monolayer $FeAl_2S_4$ identified using RomeoDFT (see Section S6).

**Lattice vectors:** Cartesian components (in [Å]) of the lattice vectors for $FeAl_2S_4$.

| | $x$ | $y$ | $z$ |
|---|---|---|---|
| $\boldsymbol{a_1}$ | 7.1292 | 0.0000 | 0.0000 |
| $\boldsymbol{a_2}$ | −1.7823 | 3.0870 | 0.0000 |
| $\boldsymbol{a_3}$ | 0.0000 | 0.0000 | 28.6902 |

**Atomic positions:** Fractional coordinates, Hubbard $U$ (in eV) and magnetic moments (in $\mu_B$, computed from orbital occupations $m_o$ or integration spheres $m_i$) of each atom of monolayer $FeAl_2S_4$.

| atom | $x$ | $y$ | $z$ | $U$ | $m_o$ | $m_i$ |
|---|---|---|---|---|---|---|
| Fe | 0.33 | 0.33 | 0.50 | 5.35 | 2.25 | 2.20 |
| Fe | 0.83 | 0.33 | 0.50 | 5.35 | 3.12 | 3.19 |
| S | 0.33 | 0.33 | 0.65 | 0.0 | – | 0.01 |
| Al | 0.50 | 1.00 | 0.39 | 0.0 | – | 0.01 |
| S | 0.50 | 1.00 | 0.46 | 0.0 | – | −0.04 |
| Al | 0.17 | 0.67 | 0.61 | 0.0 | – | 0.01 |
| S | 0.17 | 0.67 | 0.54 | 0.0 | – | −0.04 |
| S | 0.33 | 0.33 | 0.35 | 0.0 | – | 0.01 |
| S | 0.83 | 0.33 | 0.65 | 0.0 | – | 0.01 |
| Al | 1.00 | 1.00 | 0.39 | 0.0 | – | 0.01 |
| S | 1.00 | 1.00 | 0.46 | 0.0 | – | −0.02 |
| Al | 0.67 | 0.67 | 0.61 | 0.0 | – | 0.01 |
| S | 0.67 | 0.67 | 0.54 | 0.0 | – | −0.02 |
| S | 0.83 | 0.33 | 0.35 | 0.0 | – | 0.01 |

## $FeBr_2$ (AFM)

Band gap: 2.82 eV
Total magnetization: $-0.0\ \mu_B/cell$
Absolute magnetization: $7.76\ \mu_B/cell$
MC2D entry: https://mc2d.materialscloud.org/#/details/mc2d-33

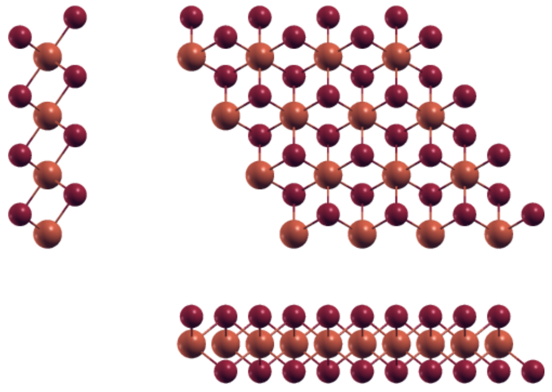

**Geometry:** Views of $FeBr_2$ as seen from the $x$ axis (left), the $y$ axis (bottom), and the $z$ axis (center).

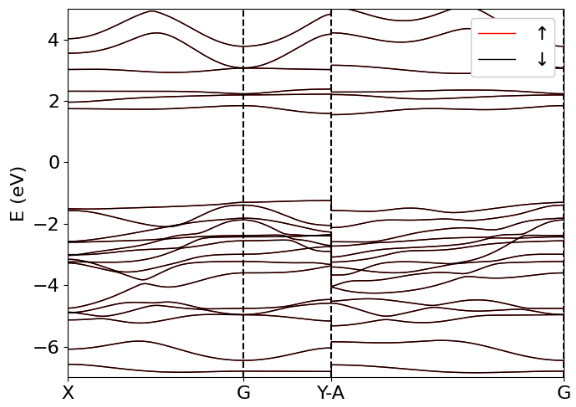


**Electronic bandstructure:** Spin-resolved energy bands of monolayer $FeBr_2$ along a high-symmetry path.

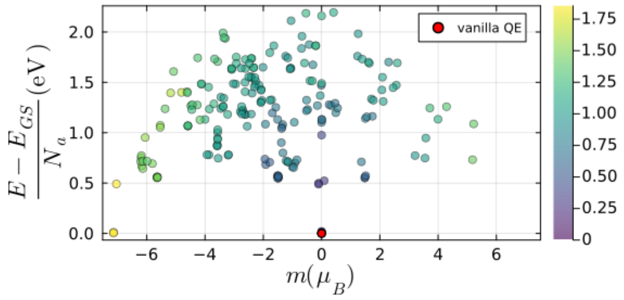


**Unique states:** Representation of 208 self-consistent unique states for monolayer $FeBr_2$ identified using RomeoDFT (see Section S6).

**Lattice vectors:** Cartesian components (in [Å]) of the lattice vectors for $FeBr_2$.

| | $x$ | $y$ | $z$ |
|---|---|---|---|
| $\boldsymbol{a_1}$ | 7.4591 | 0.0000 | 0.0000 |
| $\boldsymbol{a_2}$ | −1.8648 | 3.2299 | 0.0000 |
| $\boldsymbol{a_3}$ | 0.0000 | 0.0000 | 22.6493 |

**Atomic positions:** Fractional coordinates, Hubbard $U$ (in eV) and magnetic moments (in $\mu_B$, computed from orbital occupations $m_o$ or integration spheres $m_i$) of each atom of monolayer $FeBr_2$.

| atom | $x$ | $y$ | $z$ | $U$ | $m_o$ | $m_i$ |
|---|---|---|---|---|---|---|
| Fe | 0.0 | 0.0 | 0.50 | 5.36 | 3.57 | 3.64 |
| Fe | 0.50 | 0.0 | 0.50 | 5.36 | −3.57 | −3.64 |
| Br | 0.17 | 0.67 | 0.43 | 0.0 | – | 0.01 |
| Br | 0.33 | 0.33 | 0.57 | 0.0 | – | −0.01 |
| Br | 0.67 | 0.67 | 0.43 | 0.0 | – | −0.01 |
| Br | 0.83 | 0.33 | 0.57 | 0.0 | – | 0.01 |

# $FeCl_2$ (AFM)

Band gap: 3.55 eV
Total magnetization: 0.0 $\mu_B/cell$
Absolute magnetization: 7.78 $\mu_B/cell$
MC2D entry: https://mc2d.materialscloud.org/#/details/mc2d-89

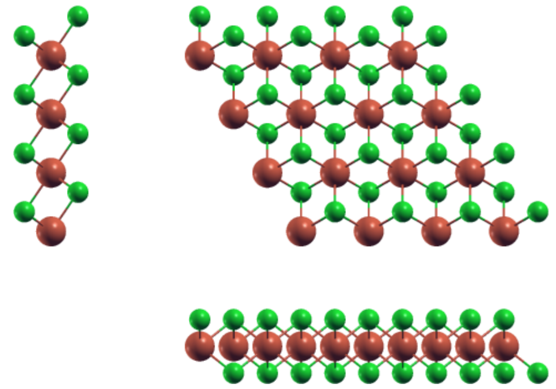

**Geometry:** Views of $FeCl_2$ as seen from the $x$ axis (left), the $y$ axis (bottom), and the $z$ axis (center).

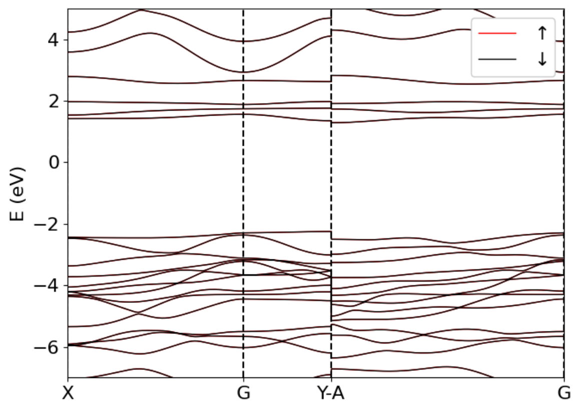


**Electronic bandstructure:** Spin-resolved energy bands of monolayer $FeCl_2$ along a high-symmetry path.

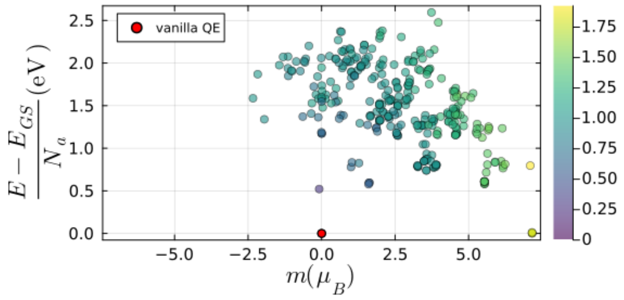


**Unique states:** Representation of 325 self-consistent unique states for monolayer $FeCl_2$ identified using RomeoDFT (see Section S6).

**Lattice vectors:** Cartesian components (in [Å]) of the lattice vectors for $FeCl_2$.

| | $x$ | $y$ | $z$ |
|---|---|---|---|
| $\boldsymbol{a_1}$ | 7.0651 | 0.0000 | 0.0000 |
| $\boldsymbol{a_2}$ | −1.7663 | 3.0593 | 0.0000 |
| $\boldsymbol{a_3}$ | 0.0000 | 0.0000 | 22.4605 |

**Atomic positions:** Fractional coordinates, Hubbard $U$ (in eV) and magnetic moments (in $\mu_B$, computed from orbital occupations $m_o$ or integration spheres $m_i$) of each atom of monolayer $FeCl_2$.

| atom | $x$ | $y$ | $z$ | $U$ | $m_o$ | $m_i$ |
|---|---|---|---|---|---|---|
| Fe | 0.0 | 0.0 | 0.50 | 5.52 | 3.58 | 3.61 |
| Fe | 0.50 | 0.0 | 0.50 | 5.52 | −3.58 | −3.61 |
| Cl | 0.33 | 0.33 | 0.56 | 0.0 | – | −0.02 |
| Cl | 0.17 | 0.67 | 0.44 | 0.0 | – | 0.02 |
| Cl | 0.83 | 0.33 | 0.56 | 0.0 | – | 0.02 |
| Cl | 0.67 | 0.67 | 0.44 | 0.0 | – | −0.02 |

# $FeGa_2S_4$ (AFM)

Band gap: 0.48 eV
Total magnetization: $-0.0\ \mu_B/cell$
Absolute magnetization: $7.32\ \mu_B/cell$
MC2D entry: https://mc2d.materialscloud.org/#/details/mc2d-1612

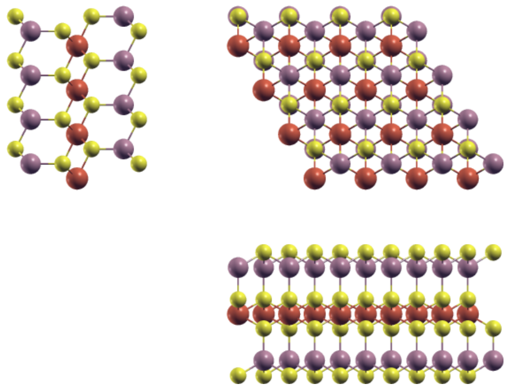

**Geometry:** Views of $FeGa_2S_4$ as seen from the $x$ axis (left), the $y$ axis (bottom), and the $z$ axis (center).

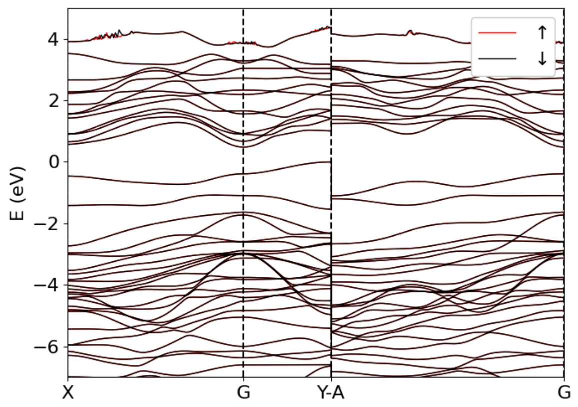


**Electronic bandstructure:** Spin-resolved energy bands of monolayer $FeGa_2S_4$ along a high-symmetry path.

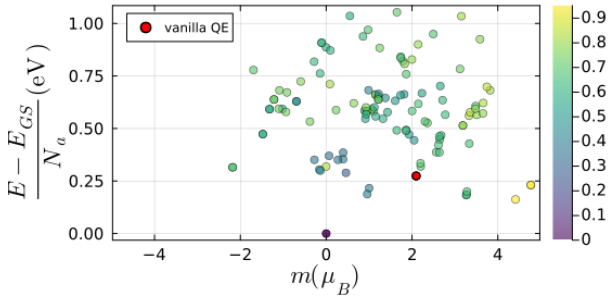


**Unique states:** Representation of 121 self-consistent unique states for monolayer $FeGa_2S_4$ identified using RomeoDFT (see Section S6).

**Lattice vectors:** Cartesian components (in [Å]) of the lattice vectors for $FeGa_2S_4$.

| | $x$ | $y$ | $z$ |
|---|---|---|---|
| $\boldsymbol{a_1}$ | 7.2013 | 0.0000 | 0.0000 |
| $\boldsymbol{a_2}$ | −1.8003 | 3.1183 | 0.0000 |
| $\boldsymbol{a_3}$ | 0.0000 | 0.0000 | 28.7774 |

**Atomic positions:** Fractional coordinates, Hubbard $U$ (in eV) and magnetic moments (in $\mu_B$, computed from orbital occupations $m_o$ or integration spheres $m_i$) of each atom of monolayer $FeGa_2S_4$.

| atom | $x$ | $y$ | $z$ | $U$ | $m_o$ | $m_i$ |
|---|---|---|---|---|---|---|
| Fe | 0.0 | 0.0 | 0.50 | 5.48 | −3.13 | −3.20 |
| Fe | 0.50 | 0.0 | 0.50 | 5.48 | 3.13 | 3.20 |
| Ga | 0.33 | 0.33 | 0.61 | 0.0 | – | 0.00 |
| Ga | 0.17 | 0.67 | 0.39 | 0.0 | – | 0.00 |
| S | 0.33 | 0.33 | 0.54 | 0.0 | – | 0.00 |
| S | 0.17 | 0.67 | 0.46 | 0.0 | – | 0.00 |
| S | 0.33 | 0.33 | 0.35 | 0.0 | – | −0.01 |
| S | 0.17 | 0.67 | 0.65 | 0.0 | – | 0.01 |
| Ga | 0.83 | 0.33 | 0.61 | 0.0 | – | 0.00 |
| Ga | 0.67 | 0.67 | 0.39 | 0.0 | – | 0.00 |
| S | 0.83 | 0.33 | 0.54 | 0.0 | – | 0.00 |
| S | 0.67 | 0.67 | 0.46 | 0.0 | – | 0.00 |
| S | 0.83 | 0.33 | 0.35 | 0.0 | – | 0.01 |
| S | 0.67 | 0.67 | 0.65 | 0.0 | – | −0.01 |

## $FeHO_2$ (FM)

Band gap: 0.0 eV
Total magnetization: 5.97 $\mu_B/cell$
Absolute magnetization: 6.55 $\mu_B/cell$
MC2D entry: https://mc2d.materialscloud.org/#/details/mc2d-1500

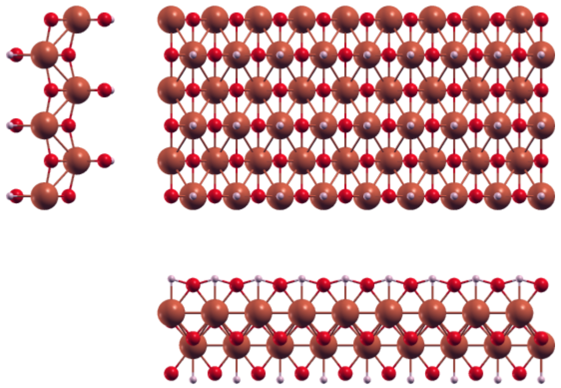

**Geometry:** Views of $FeHO_2$ as seen from the $x$ axis (left), the $y$ axis (bottom), and the $z$ axis (center).

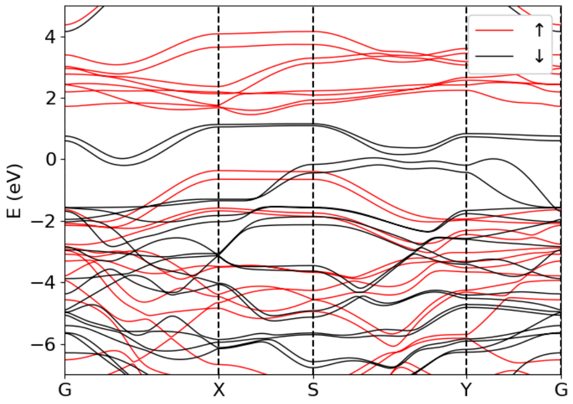


**Electronic bandstructure:** Spin-resolved energy bands of monolayer $FeHO_2$ along a high-symmetry path.

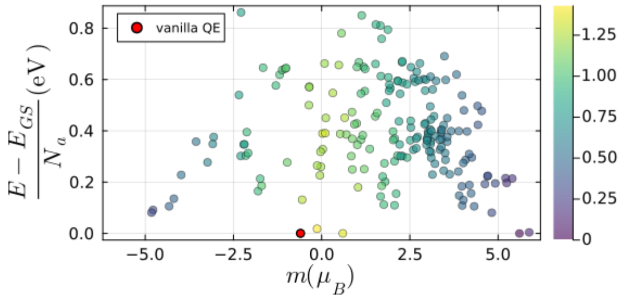


**Unique states:** Representation of 195 self-consistent unique states for monolayer $FeHO_2$ identified using RomeoDFT (see Section S6).

**Lattice vectors:** Cartesian components (in [Å]) of the lattice vectors for $FeHO_2$.

| | $x$ | $y$ | $z$ |
|---|---|---|---|
| $a_1$ | 2.4551 | 0.0000 | 0.0000 |
| $a_2$ | 0.0000 | 3.9815 | 0.0000 |
| $a_3$ | 0.0000 | 0.0000 | 25.8276 |

**Atomic positions:** Fractional coordinates, Hubbard $U$ (in eV) and magnetic moments (in $\mu_B$, computed from orbital occupations $m_o$ or integration spheres $m_i$) of each atom of monolayer $FeHO_2$.

| atom | $x$ | $y$ | $z$ | $U$ | $m_o$ | $m_i$ |
|---|---|---|---|---|---|---|
| Fe | 0.50 | 0.31 | 0.53 | 4.95 | 2.73 | 2.58 |
| Fe | 0.0 | 0.81 | 0.46 | 4.64 | 2.87 | 2.75 |
| H | 0.50 | 0.31 | 0.61 | 0.0 | – | 0.00 |
| O | 0.50 | 0.81 | 0.40 | 0.0 | – | 0.00 |
| O | 0.50 | 0.81 | 0.52 | 0.0 | – | −0.04 |
| H | 0.0 | 0.81 | 0.39 | 0.0 | – | 0.00 |
| O | 0.0 | 0.31 | 0.60 | 0.0 | – | −0.01 |
| O | 0.0 | 0.31 | 0.48 | 0.0 | – | −0.03 |

## $FeI_2$ (AFM)

Band gap: 1.90 eV
Total magnetization: 0.0 $\mu_B/cell$
Absolute magnetization: 7.69 $\mu_B/cell$
MC2D entry: https://mc2d.materialscloud.org/#/details/mc2d-144

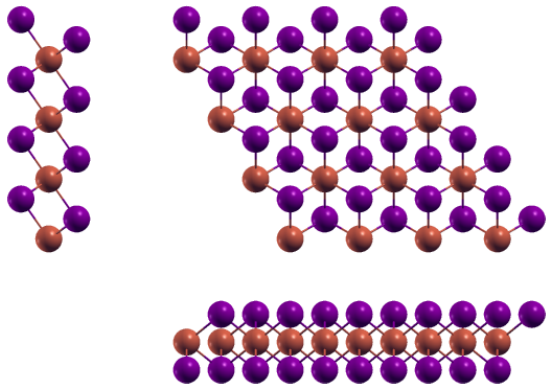

**Geometry:** Views of $FeI_2$ as seen from the $x$ axis (left), the $y$ axis (bottom), and the $z$ axis (center).

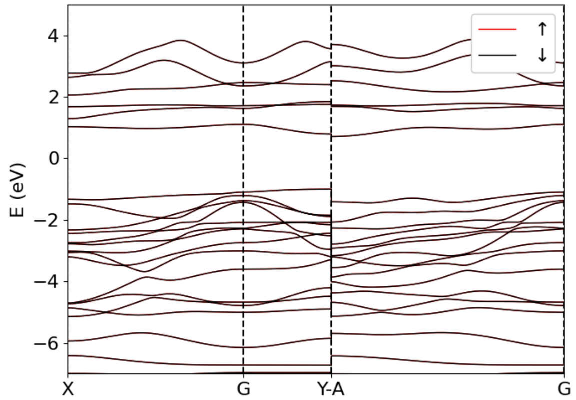


**Electronic bandstructure:** Spin-resolved energy bands of monolayer $FeI_2$ along a high-symmetry path.

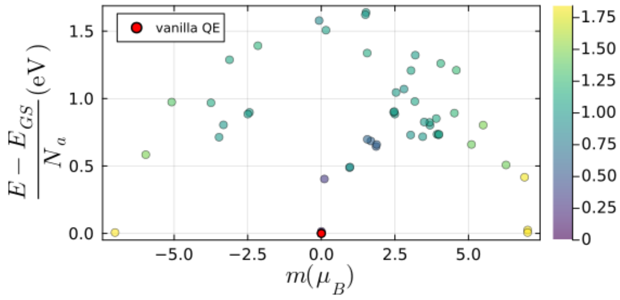


**Unique states:** Representation of 52 self-consistent unique states for monolayer $FeI_2$ identified using RomeoDFT (see Section S6).

**Lattice vectors:** Cartesian components (in [Å]) of the lattice vectors for $FeI_2$.

| | $x$ | $y$ | $z$ |
|---|---|---|---|
| $\boldsymbol{a_1}$ | 4.0113 | −6.9478 | 0.0000 |
| $\boldsymbol{a_2}$ | 2.0057 | 3.4739 | 0.0000 |
| $\boldsymbol{a_3}$ | 0.0000 | 0.0000 | 17.6268 |

**Atomic positions:** Fractional coordinates, Hubbard $U$ (in eV) and magnetic moments (in $\mu_B$, computed from orbital occupations $m_o$ or integration spheres $m_i$) of each atom of monolayer $FeI_2$.

| atom | $x$ | $y$ | $z$ | $U$ | $m_o$ | $m_i$ |
|---|---|---|---|---|---|---|
| Fe | 0.0 | 0.0 | −0.50 | 4.87 | 3.50 | 3.61 |
| Fe | 0.50 | 0.0 | −0.50 | 4.87 | −3.50 | −3.61 |
| I | 0.33 | 0.33 | −0.59 | 0.0 | – | −0.01 |
| I | 0.17 | 0.67 | −0.41 | 0.0 | – | 0.01 |
| I | 0.83 | 0.33 | −0.59 | 0.0 | – | 0.01 |
| I | 0.67 | 0.67 | −0.41 | 0.0 | – | −0.01 |

# $FeO_2$ (Pmmn) (FM)

Band gap: 0.0 eV
Total magnetization: $-5.14\ \mu_B/cell$
Absolute magnetization: $5.74\ \mu_B/cell$
MC2D entry: https://mc2d.materialscloud.org/#/details/mc2d-145

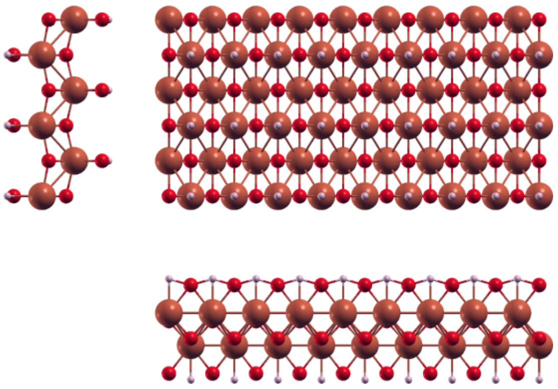

**Geometry:** Views of $FeO_2$ (Pmmn) as seen from the $x$ axis (left), the $y$ axis (bottom), and the $z$ axis (center).

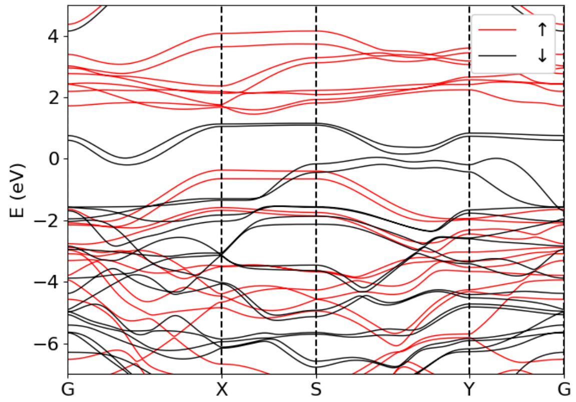


**Electronic bandstructure:** Spin-resolved energy bands of monolayer $FeO_2$ (Pmmn) along a high-symmetry path.

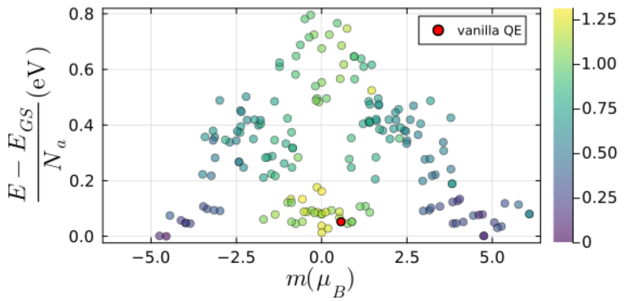


**Unique states:** Representation of 183 self-consistent unique states for monolayer $FeO_2$ (Pmmn) identified using RomeoDFT (see Section S6).

**Lattice vectors:** Cartesian components (in [Å]) of the lattice vectors for $FeO_2$ (Pmmn).

| | $x$ | $y$ | $z$ |
|---|---|---|---|
| $\boldsymbol{a_1}$ | 2.7836 | 0.0000 | 0.0000 |
| $\boldsymbol{a_2}$ | 0.0000 | 3.8028 | 0.0000 |
| $\boldsymbol{a_3}$ | 0.0000 | 0.0000 | 24.0036 |

**Atomic positions:** Fractional coordinates, Hubbard $U$ (in eV) and magnetic moments (in $\mu_B$, computed from orbital occupations $m_o$ or integration spheres $m_i$) of each atom of monolayer $FeO_2$ (Pmmn).

| atom | $x$ | $y$ | $z$ | $U$ | $m_o$ | $m_i$ |
|---|---|---|---|---|---|---|
| Fe | 0.0 | 0.75 | 0.54 | 4.18 | −2.28 | −2.19 |
| Fe | 0.50 | 0.25 | 0.46 | 4.18 | −2.28 | −2.19 |
| O | 0.50 | 0.75 | 0.58 | 0.0 | – | −0.12 |
| O | 0.0 | 0.25 | 0.42 | 0.0 | – | −0.12 |
| O | 0.50 | 0.75 | 0.48 | 0.0 | – | 0.10 |
| O | 0.0 | 0.25 | 0.52 | 0.0 | – | 0.10 |

## FeOCl (AFM)

Band gap: 1.39 eV
Total magnetization: 0.0 $\mu_B/cell$
Absolute magnetization: 9.16 $\mu_B/cell$
MC2D entry: https://mc2d.materialscloud.org/#/details/mc2d-108

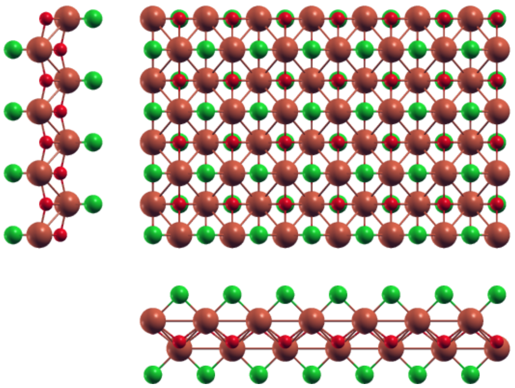

**Geometry:** Views of FeOCl as seen from the $x$ axis (left), the $y$ axis (bottom), and the $z$ axis (center).

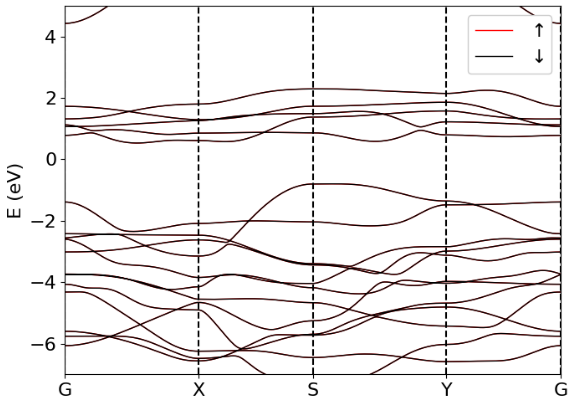


**Electronic bandstructure:** Spin-resolved energy bands of monolayer FeOCl along a high-symmetry path.

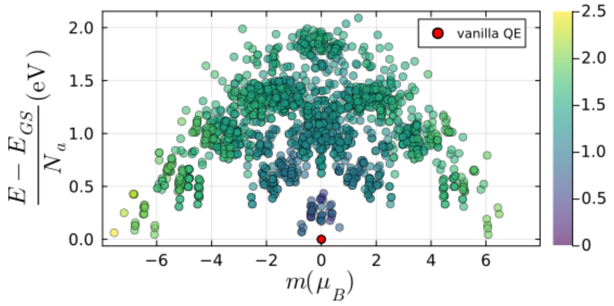


**Unique states:** Representation of 1878 self-consistent unique states for monolayer FeOCl identified using RomeoDFT (see Section S6).

**Lattice vectors:** Cartesian components (in [Å]) of the lattice vectors for FeOCl.

| | $x$ | $y$ | $z$ |
|---|---|---|---|
| $\boldsymbol{a_1}$ | 3.1842 | 0.0000 | 0.0000 |
| $\boldsymbol{a_2}$ | 0.0000 | 3.7169 | 0.0000 |
| $\boldsymbol{a_3}$ | 0.0000 | 0.0000 | 24.8140 |

**Atomic positions:** Fractional coordinates, Hubbard $U$ (in eV) and magnetic moments (in $\mu_B$, computed from orbital occupations $m_o$ or integration spheres $m_i$) of each atom of monolayer FeOCl.

| atom | $x$ | $y$ | $z$ | $U$ | $m_o$ | $m_i$ |
|---|---|---|---|---|---|---|
| Fe | 0.0 | 0.50 | 0.47 | 6.26 | 3.75 | 3.75 |
| Fe | 0.50 | 0.0 | 0.53 | 6.26 | −3.75 | −3.75 |
| Cl | 0.50 | 0.50 | 0.40 | 0.0 | – | 0.19 |
| O | 0.0 | 0.0 | 0.48 | 0.0 | – | −0.02 |
| Cl | 0.0 | 0.0 | 0.60 | 0.0 | – | −0.19 |
| O | 0.50 | 0.50 | 0.52 | 0.0 | – | 0.02 |

## $FePS_3$ (FM)

Band gap: 0.0 eV
Total magnetization: $-3.98\ \mu_B/cell$
Absolute magnetization: $5.16\ \mu_B/cell$
MC2D entry: https://mc2d.materialscloud.org/#/details/mc2d-1245

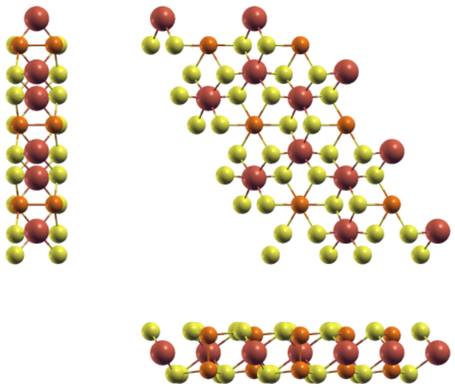

**Geometry:** Views of $FePS_3$ as seen from the $x$ axis (left), the $y$ axis (bottom), and the $z$ axis (center).

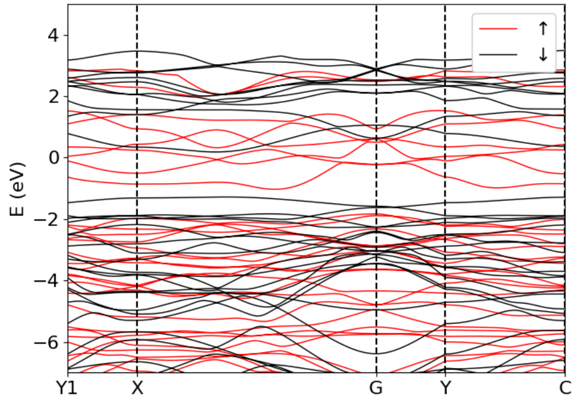


**Electronic bandstructure:** Spin-resolved energy bands of monolayer $FePS_3$ along a high-symmetry path.

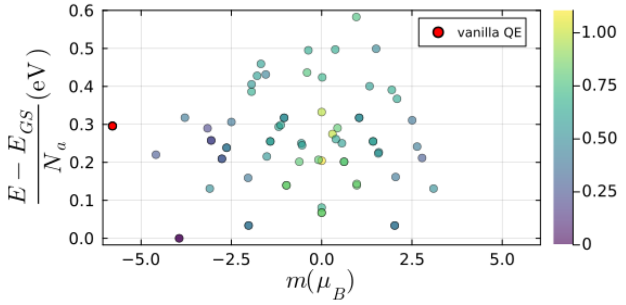


**Unique states:** Representation of 70 self-consistent unique states for monolayer $FePS_3$ identified using RomeoDFT (see Section S6).

**Lattice vectors:** Cartesian components (in [Å]) of the lattice vectors for $FePS_3$.

| | $x$ | $y$ | $z$ |
|---|---|---|---|
| $\boldsymbol{a_1}$ | 5.7528 | 0.0000 | 0.0000 |
| $\boldsymbol{a_2}$ | −2.8599 | 4.9916 | 0.0000 |
| $\boldsymbol{a_3}$ | 0.0000 | 0.0000 | 22.7081 |

**Atomic positions:** Fractional coordinates, Hubbard $U$ (in eV) and magnetic moments (in $\mu_B$, computed from orbital occupations $m_o$ or integration spheres $m_i$) of each atom of monolayer $FePS_3$.

| atom | $x$ | $y$ | $z$ | $U$ | $m_o$ | $m_i$ |
|---|---|---|---|---|---|---|
| Fe | 0.99 | 0.32 | 0.50 | 5.31 | −1.97 | −1.96 |
| Fe | 0.32 | 0.99 | 0.50 | 5.31 | −1.97 | −1.96 |
| P | 0.66 | 0.66 | 0.55 | 0.0 | – | −0.05 |
| S | 0.66 | 0.29 | 0.56 | 0.0 | – | 0.06 |
| S | 0.29 | 0.66 | 0.56 | 0.0 | – | 0.06 |
| S | 0.03 | 0.03 | 0.56 | 0.0 | – | 0.06 |
| P | 0.66 | 0.66 | 0.45 | 0.0 | – | −0.05 |
| S | 0.03 | 0.66 | 0.44 | 0.0 | – | 0.06 |
| S | 0.66 | 0.03 | 0.44 | 0.0 | – | 0.06 |
| S | 0.29 | 0.29 | 0.44 | 0.0 | – | 0.06 |

## FeTe (FM)

Band gap: 0.0 eV
Total magnetization: $-11.77\ \mu_B/cell$
Absolute magnetization: $12.56\ \mu_B/cell$
MC2D entry: https://mc2d.materialscloud.org/#/details/mc2d-148

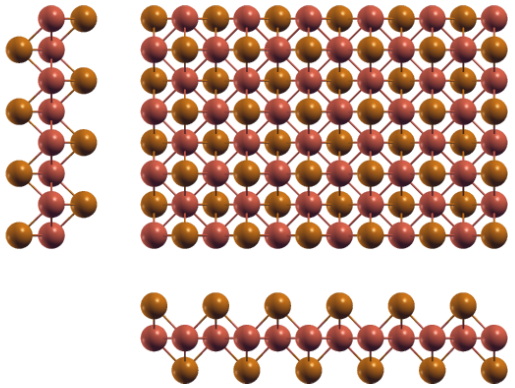

**Geometry:** Views of FeTe as seen from the $x$ axis (left), the $y$ axis (bottom), and the $z$ axis (center).

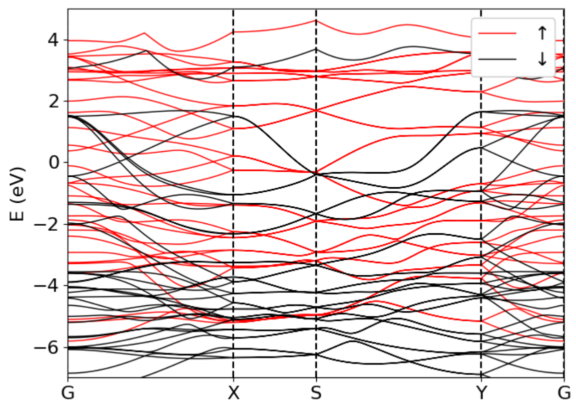


**Electronic bandstructure:** Spin-resolved energy bands of monolayer FeTe along a high-symmetry path.

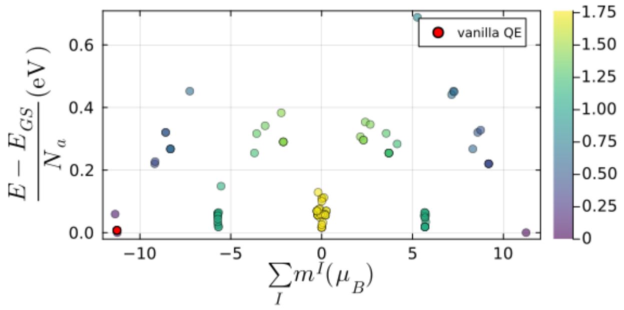


**Unique states:** Representation of 95 self-consistent unique states for monolayer FeTe identified using RomeoDFT (see Section S6).

**Lattice vectors:** Cartesian components (in [Å]) of the lattice vectors for FeTe.

| | $x$ | $y$ | $z$ |
|---|---|---|---|
| $\boldsymbol{a_1}$ | 7.2300 | 0.0000 | 0.0000 |
| $\boldsymbol{a_2}$ | 0.0000 | 3.6150 | 0.0000 |
| $\boldsymbol{a_3}$ | 0.0000 | 0.0000 | 23.2702 |

**Atomic positions:** Fractional coordinates, Hubbard $U$ (in eV) and magnetic moments (in $\mu_B$, computed from orbital occupations $m_o$ or integration spheres $m_i$) of each atom of monolayer FeTe.

| atom | $x$ | $y$ | $z$ | $U$ | $m_o$ | $m_i$ |
|---|---|---|---|---|---|---|
| Fe | 0.0 | 0.0 | 0.50 | 4.54 | −2.81 | −2.88 |
| Fe | 0.25 | 0.50 | 0.50 | 4.54 | −2.81 | −2.88 |
| Fe | 0.50 | 0.0 | 0.50 | 4.54 | −2.81 | −2.88 |
| Fe | 0.75 | 0.50 | 0.50 | 4.54 | −2.81 | −2.88 |
| Te | 0.25 | 0.0 | 0.58 | 0.0 | – | 0.06 |
| Te | 0.0 | 0.50 | 0.42 | 0.0 | – | 0.06 |
| Te | 0.75 | 0.0 | 0.58 | 0.0 | – | 0.06 |
| Te | 0.50 | 0.50 | 0.42 | 0.0 | – | 0.06 |

## GaSe (FM)

Band gap: 0.0 eV
Total magnetization: $-0.92\ \mu_B/cell$
Absolute magnetization: $0.94\ \mu_B/cell$
MC2D entry: https://mc2d.materialscloud.org/#/details/mc2d-2625

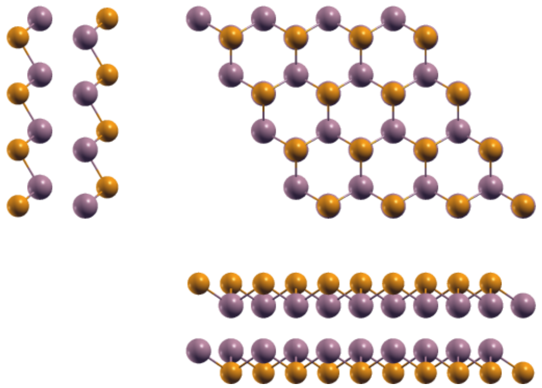

**Geometry:** Views of GaSe as seen from the $x$ axis (left), the $y$ axis (bottom), and the $z$ axis (center).

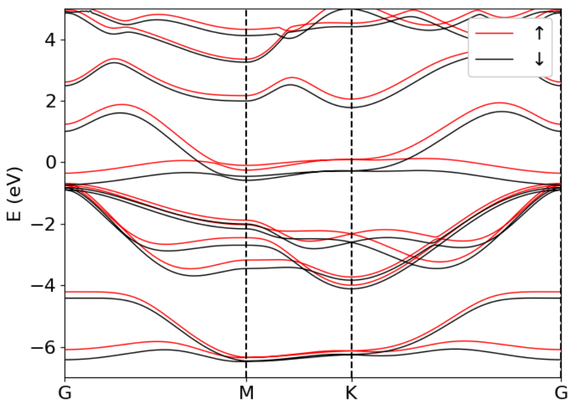


**Electronic bandstructure:** Spin-resolved energy bands of monolayer GaSe along a high-symmetry path.

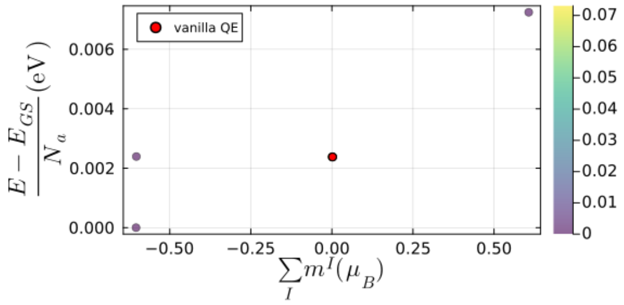


**Unique states:** Representation of 4 self-consistent unique states for monolayer GaSe identified using RomeoDFT (see Section S6).

**Lattice vectors:** Cartesian components (in [Å]) of the lattice vectors for GaSe.

| | $x$ | $y$ | $z$ |
|---|---|---|---|
| $\boldsymbol{a_1}$ | 3.7599 | 0.0000 | 0.0000 |
| $\boldsymbol{a_2}$ | −1.8799 | 3.2561 | 0.0000 |
| $\boldsymbol{a_3}$ | 0.0000 | 0.0000 | 25.1855 |

**Atomic positions:** Fractional coordinates, Hubbard $U$ (in eV) and magnetic moments (in $\mu_B$, computed from orbital occupations $m_o$ or integration spheres $m_i$) of each atom of monolayer GaSe.

| atom | $x$ | $y$ | $z$ | $U$ | $m_o$ | $m_i$ |
|---|---|---|---|---|---|---|
| Ga | 0.33 | 0.67 | 0.55 | 0.00 | −0.13 | −0.08 |
| Se | 0.67 | 0.33 | 0.60 | 0.00 | −0.17 | −0.14 |
| Ga | 0.67 | 0.33 | 0.45 | 0.00 | −0.13 | −0.08 |
| Se | 0.33 | 0.67 | 0.40 | 0.00 | −0.17 | −0.14 |

## $Gd_2CCl_2$ (FM)

Band gap: 0.43 eV
Total magnetization: $-13.86\ \mu_B/cell$
Absolute magnetization: 14.52 $\mu_B/cell$
MC2D entry: https://mc2d.materialscloud.org/#/details/mc2d-2518

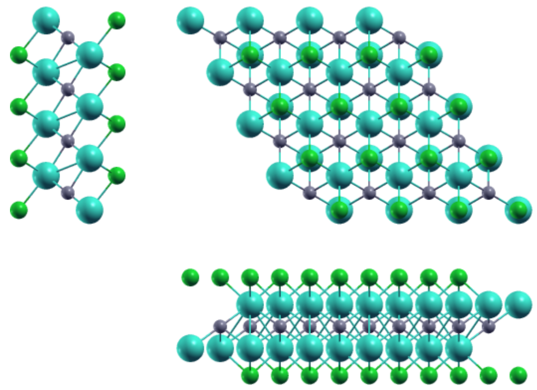

**Geometry:** Views of $Gd_2CCl_2$ as seen from the $x$ axis (left), the $y$ axis (bottom), and the $z$ axis (center).

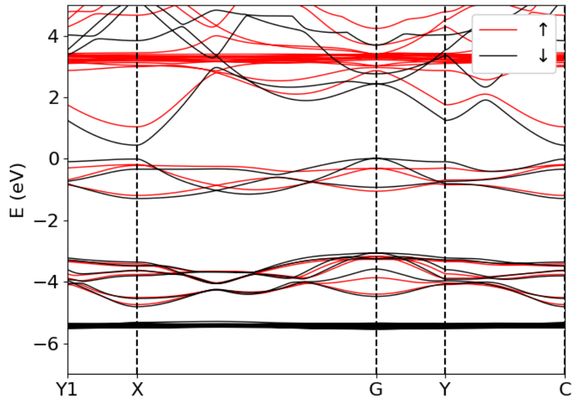


**Electronic bandstructure:** Spin-resolved energy bands of monolayer $Gd_2CCl_2$ along a high-symmetry path.

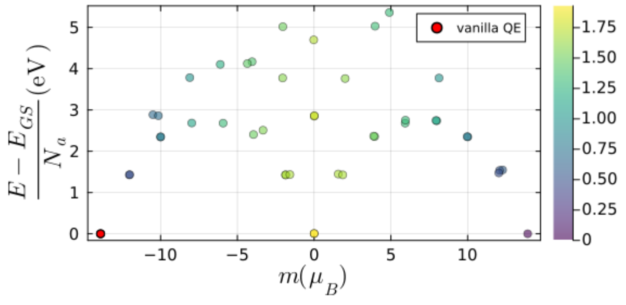


**Unique states:** Representation of 44 self-consistent unique states for monolayer $Gd_2CCl_2$ identified using RomeoDFT (see Section S6).

**Lattice vectors:** Cartesian components (in [Å]) of the lattice vectors for $Gd_2CCl_2$.

| | $x$ | $y$ | $z$ |
|---|---|---|---|
| $\boldsymbol{a_1}$ | 1.8802 | −3.2567 | 0.0000 |
| $\boldsymbol{a_2}$ | 1.8802 | 3.2567 | 0.0000 |
| $\boldsymbol{a_3}$ | 0.0000 | 0.0000 | 24.2650 |

**Atomic positions:** Fractional coordinates, Hubbard $U$ (in eV) and magnetic moments (in $\mu_B$, computed from orbital occupations $m_o$ or integration spheres $m_i$) of each atom of monolayer $Gd_2CCl_2$.

| atom | $x$ | $y$ | $z$ | $U$ | $m_o$ | $m_i$ |
|---|---|---|---|---|---|---|
| Gd | 0.17 | −0.17 | 0.06 | 3.38 | −6.96 | −6.93 |
| Gd | −0.17 | 0.17 | −0.06 | 3.38 | −6.96 | −6.93 |
| C | −0.50 | −0.50 | 0.0 | 0.0 | – | 0.26 |
| Cl | 0.17 | −0.17 | −0.13 | 0.0 | – | 0.02 |
| Cl | −0.17 | 0.17 | 0.13 | 0.0 | – | 0.02 |

## $Gd_2GeBr_2$ (AFM)

Band gap: 0.21 eV
Total magnetization: $-0.0\ \mu_B/cell$
Absolute magnetization: 14.49 $\mu_B/cell$
MC2D entry: https://mc2d.materialscloud.org/#/details/mc2d-2411

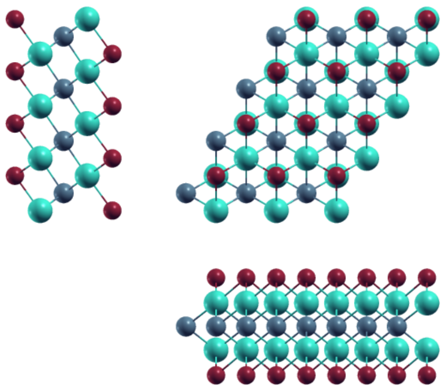

**Geometry:** Views of $Gd_2GeBr_2$ as seen from the $x$ axis (left), the $y$ axis (bottom), and the $z$ axis (center).

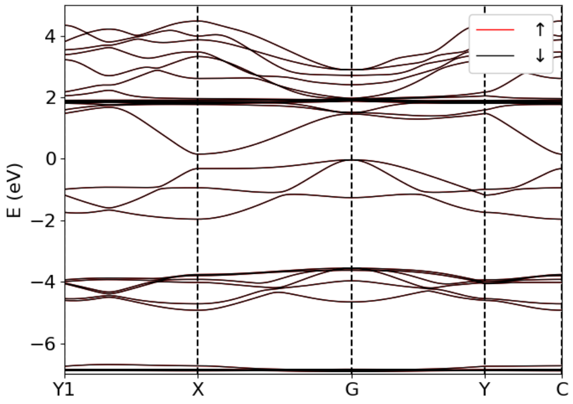


**Electronic bandstructure:** Spin-resolved energy bands of monolayer $Gd_2GeBr_2$ along a high-symmetry path.

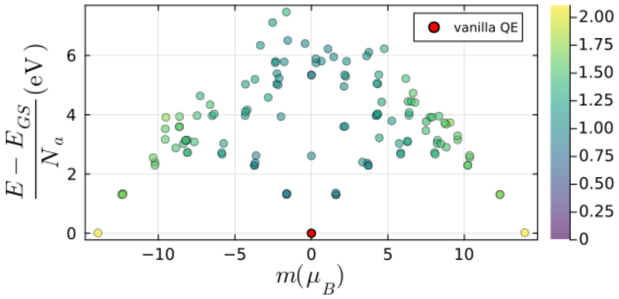


**Unique states:** Representation of 127 self-consistent unique states for monolayer $Gd_2GeBr_2$ identified using RomeoDFT (see Section S6).

**Lattice vectors:** Cartesian components (in [Å]) of the lattice vectors for $Gd_2GeBr_2$.

| | $x$ | $y$ | $z$ |
|---|---|---|---|
| $\boldsymbol{a_1}$ | 2.0979 | −3.6337 | 0.0000 |
| $\boldsymbol{a_2}$ | 4.1958 | 0.0000 | 0.0000 |
| $\boldsymbol{a_3}$ | 0.0000 | 0.0000 | 25.7076 |

**Atomic positions:** Fractional coordinates, Hubbard $U$ (in eV) and magnetic moments (in $\mu_B$, computed from orbital occupations $m_o$ or integration spheres $m_i$) of each atom of monolayer $Gd_2GeBr_2$.

| atom | $x$ | $y$ | $z$ | $U$ | $m_o$ | $m_i$ |
|---|---|---|---|---|---|---|
| Gd | 0.67 | 0.17 | 0.06 | 3.42 | −6.98 | −7.05 |
| Gd | 0.33 | 0.83 | 0.94 | 3.42 | 6.98 | 7.05 |
| Ge | 1.00 | 0.50 | 0.0 | 0.0 | – | 0.0 |
| Br | 0.67 | 0.17 | 0.87 | 0.0 | – | −0.02 |
| Br | 0.33 | 0.83 | 0.13 | 0.0 | – | 0.02 |

## $Gd_2GeI_2$ (AFM)

Band gap: 0.05 eV
Total magnetization: $-0.0$ $\mu_B/cell$
Absolute magnetization: 14.58 $\mu_B/cell$
MC2D entry: https://mc2d.materialscloud.org/#/details/mc2d-2288

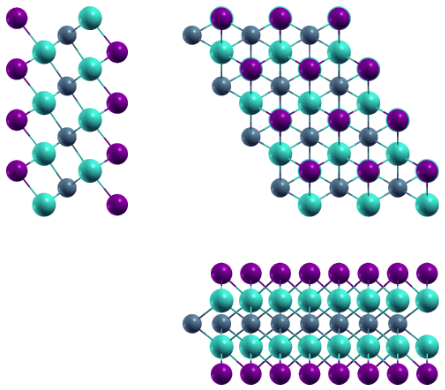

**Geometry:** Views of $Gd_2GeI_2$ as seen from the $x$ axis (left), the $y$ axis (bottom), and the $z$ axis (center).

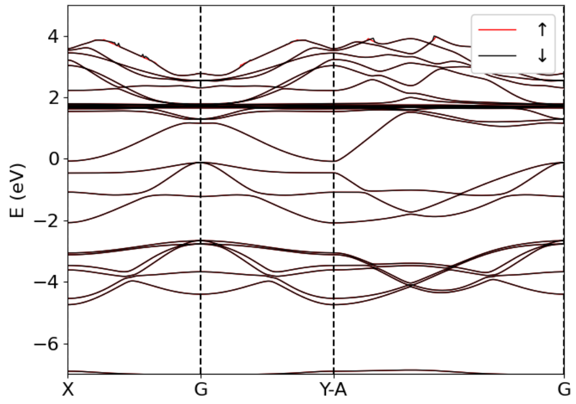


**Electronic bandstructure:** Spin-resolved energy bands of monolayer $Gd_2GeI_2$ along a high-symmetry path.

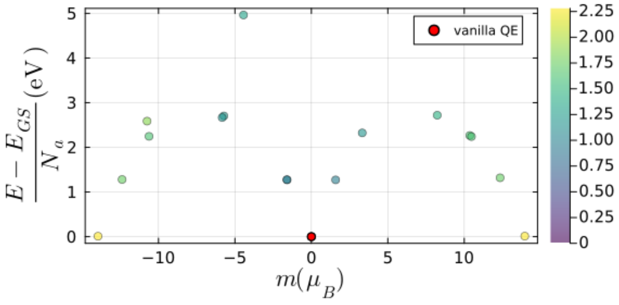


**Unique states:** Representation of 18 self-consistent unique states for monolayer $Gd_2GeI_2$ identified using RomeoDFT (see Section S6).

**Lattice vectors:** Cartesian components (in [Å]) of the lattice vectors for $Gd_2GeI_2$.

| | $x$ | $y$ | $z$ |
|---|---|---|---|
| $\boldsymbol{a_1}$ | 2.1385 | −3.7045 | 0.0000 |
| $\boldsymbol{a_2}$ | 2.1390 | 3.7048 | 0.0000 |
| $\boldsymbol{a_3}$ | 0.0000 | 0.0000 | 26.5104 |

**Atomic positions:** Fractional coordinates, Hubbard $U$ (in eV) and magnetic moments (in $\mu_B$, computed from orbital occupations $m_o$ or integration spheres $m_i$) of each atom of monolayer $Gd_2GeI_2$.

| atom | $x$ | $y$ | $z$ | $U$ | $m_o$ | $m_i$ |
|---|---|---|---|---|---|---|
| Gd | −0.33 | −0.17 | −0.06 | 3.45 | 6.98 | 7.07 |
| Gd | 0.33 | 0.17 | 0.06 | 3.45 | −6.98 | −7.07 |
| I | 0.33 | 0.17 | −0.14 | 0.0 | – | −0.02 |
| I | −0.33 | −0.17 | 0.14 | 0.0 | – | 0.02 |
| Ge | 0.0 | 0.50 | 0.0 | 0.0 | – | 0.00 |

## GdBr (FM)

Band gap: 0.43 eV
Total magnetization: 13.85 $\mu_B/cell$
Absolute magnetization: 14.52 $\mu_B/cell$
MC2D entry: https://mc2d.materialscloud.org/#/details/mc2d-2144

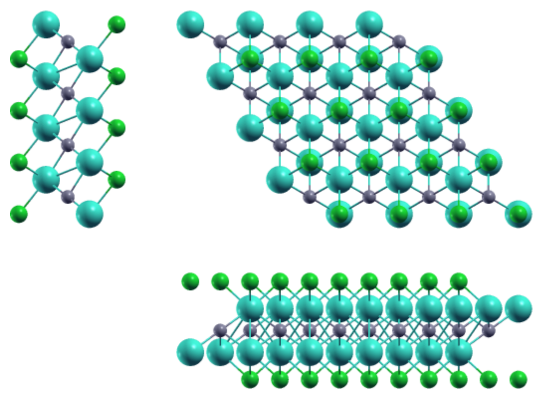

**Geometry:** Views of GdBr as seen from the $x$ axis (left), the $y$ axis (bottom), and the $z$ axis (center).

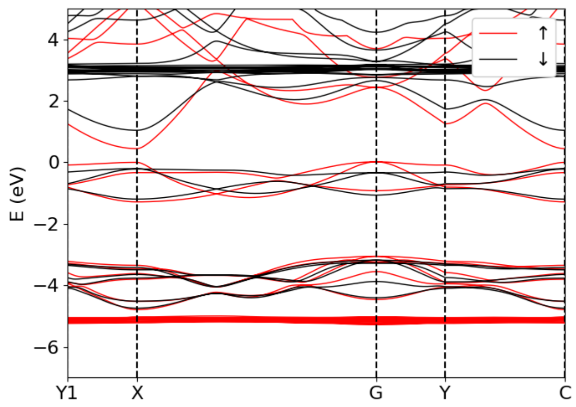


**Electronic bandstructure:** Spin-resolved energy bands of monolayer GdBr along a high-symmetry path.

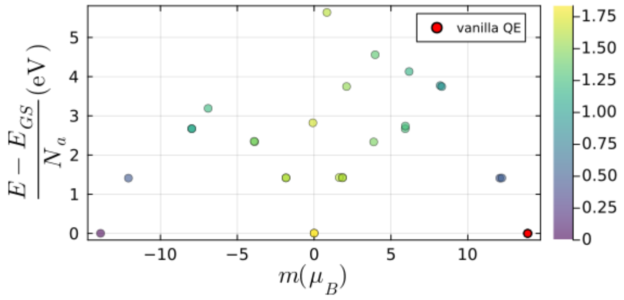


**Unique states:** Representation of 28 self-consistent unique states for monolayer GdBr identified using RomeoDFT (see Section S6).

**Lattice vectors:** Cartesian components (in [Å]) of the lattice vectors for GdBr.

| | $x$ | $y$ | $z$ |
|---|---|---|---|
| $\boldsymbol{a_1}$ | 1.8802 | −3.2567 | 0.0000 |
| $\boldsymbol{a_2}$ | 1.8802 | 3.2567 | 0.0000 |
| $\boldsymbol{a_3}$ | 0.0000 | 0.0000 | 24.2650 |

**Atomic positions:** Fractional coordinates, Hubbard $U$ (in eV) and magnetic moments (in $\mu_B$, computed from orbital occupations $m_o$ or integration spheres $m_i$) of each atom of monolayer GdBr.

| atom | $x$ | $y$ | $z$ | $U$ | $m_o$ | $m_i$ |
|---|---|---|---|---|---|---|
| Gd | 0.17 | −0.17 | 0.06 | 2.83 | 6.95 | 6.92 |
| Gd | −0.17 | 0.17 | −0.06 | 2.83 | 6.95 | 6.92 |
| C | −0.50 | −0.50 | 0.0 | 0.0 | – | −0.26 |
| Cl | 0.17 | −0.17 | −0.13 | 0.0 | – | −0.02 |
| Cl | −0.17 | 0.17 | 0.13 | 0.0 | – | −0.02 |

# $GdBr_3$ (C2/m) (AFM)

Band gap: 3.67 eV
Total magnetization: 0.0 $\mu_B/cell$
Absolute magnetization: 14.24 $\mu_B/cell$
MC2D entry: https://mc2d.materialscloud.org/#/details/mc2d-2509

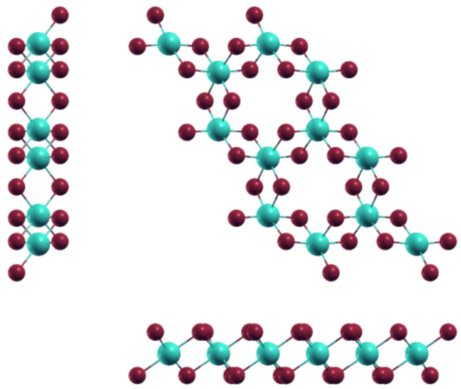

**Geometry:** Views of $GdBr_3$ (C2/m) as seen from the $x$ axis (left), the $y$ axis (bottom), and the $z$ axis (center).

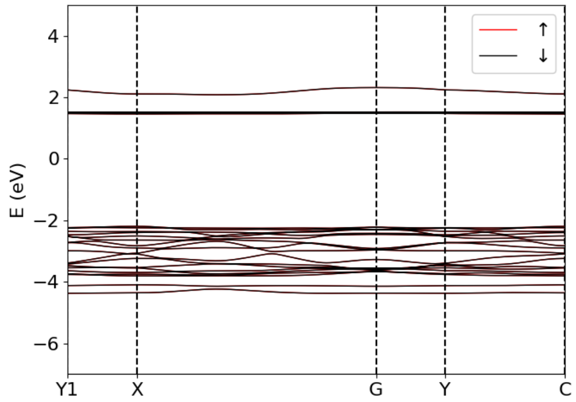


**Electronic bandstructure:** Spin-resolved energy bands of monolayer $GdBr_3$ (C2/m) along a high-symmetry path.

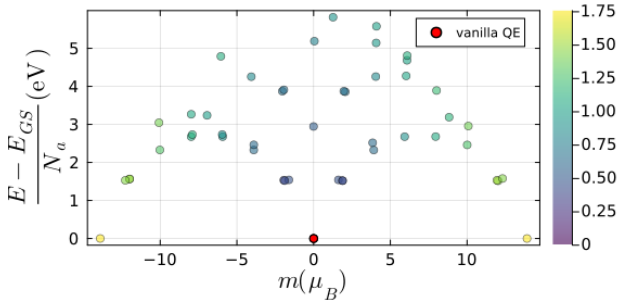


**Unique states:** Representation of 50 self-consistent unique states for monolayer $GdBr_3$ (C2/m) identified using RomeoDFT (see Section S6).

**Lattice vectors:** Cartesian components (in [Å]) of the lattice vectors for $GdBr_3$ (C2/m).

| | $x$ | $y$ | $z$ |
|---|---|---|---|
| $\boldsymbol{a_1}$ | 3.6723 | −6.3619 | 0.0000 |
| $\boldsymbol{a_2}$ | 3.6723 | 6.3619 | 0.0000 |
| $\boldsymbol{a_3}$ | 0.0000 | 0.0000 | 18.4401 |

**Atomic positions:** Fractional coordinates, Hubbard $U$ (in eV) and magnetic moments (in $\mu_B$, computed from orbital occupations $m_o$ or integration spheres $m_i$) of each atom of monolayer $GdBr_3$ (C2/m).

| atom | $x$ | $y$ | $z$ | $U$ | $m_o$ | $m_i$ |
|---|---|---|---|---|---|---|
| Gd | −0.67 | 0.67 | 0.0 | 3.29 | −6.95 | −6.97 |
| Gd | −0.33 | 0.33 | 0.0 | 3.29 | 6.95 | 6.97 |
| Br | −0.36 | 0.00 | 0.09 | 0.0 | – | 0.0 |
| Br | −1.00 | 0.64 | 0.09 | 0.0 | – | 0.0 |
| Br | −1.00 | 0.36 | −0.09 | 0.0 | – | 0.0 |
| Br | −0.64 | 0.00 | −0.09 | 0.0 | – | 0.0 |
| Br | −0.64 | 0.36 | 0.09 | 0.0 | – | 0.0 |
| Br | −0.36 | 0.64 | −0.09 | 0.0 | – | 0.0 |

## $GdBr_3$ (Pmmn) (AFM)

Band gap: 2.63 eV
Total magnetization: 0.0 $\mu_B/cell$
Absolute magnetization: 14.31 $\mu_B/cell$
MC2D entry: https://mc2d.materialscloud.org/#/details/mc2d-2600

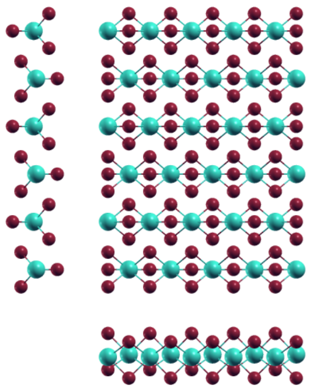

**Geometry:** Views of $GdBr_3$ (Pmmn) as seen from the $x$ axis (left), the $y$ axis (bottom), and the $z$ axis (center).

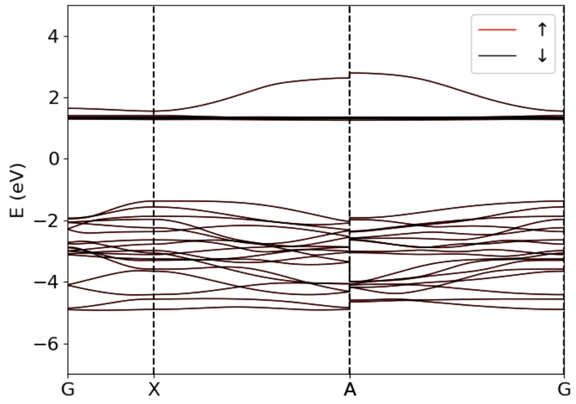


**Electronic bandstructure:** Spin-resolved energy bands of monolayer $GdBr_3$ (Pmmn) along a high-symmetry path.

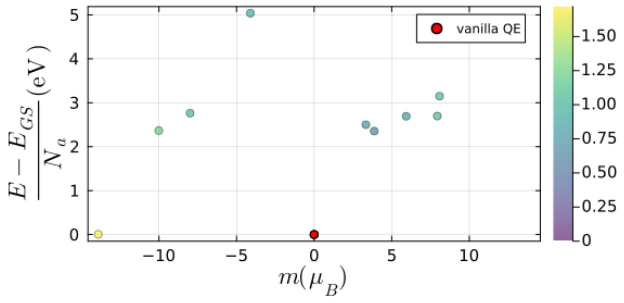


**Unique states:** Representation of 12 self-consistent unique states for monolayer $GdBr_3$ (Pmmn) identified using RomeoDFT (see Section S6).

**Lattice vectors:** Cartesian components (in [Å]) of the lattice vectors for $GdBr_3$ (Pmmn).

| | $x$ | $y$ | $z$ |
|---|---|---|---|
| $\boldsymbol{a_1}$ | 4.0132 | 0.0000 | 0.0000 |
| $\boldsymbol{a_2}$ | 0.0000 | 9.1761 | 0.0000 |
| $\boldsymbol{a_3}$ | 0.0000 | 0.0000 | 20.4651 |

**Atomic positions:** Fractional coordinates, Hubbard $U$ (in eV) and magnetic moments (in $\mu_B$, computed from orbital occupations $m_o$ or integration spheres $m_i$) of each atom of monolayer $GdBr_3$ (Pmmn).

| atom | $x$ | $y$ | $z$ | $U$ | $m_o$ | $m_i$ |
|---|---|---|---|---|---|---|
| Gd | −0.25 | −0.75 | −0.01 | 3.07 | 6.96 | 6.98 |
| Gd | 0.25 | −1.25 | 0.01 | 3.07 | −6.96 | −6.98 |
| Br | 0.25 | −0.57 | 0.07 | 0.0 | – | −0.01 |
| Br | 0.25 | −0.93 | 0.07 | 0.0 | – | −0.01 |
| Br | −0.25 | −1.07 | −0.07 | 0.0 | – | 0.01 |
| Br | −0.25 | −1.43 | −0.07 | 0.0 | – | 0.01 |
| Br | 0.25 | −0.75 | −0.10 | 0.0 | – | −0.02 |
| Br | −0.25 | −1.25 | 0.10 | 0.0 | – | 0.02 |

## GdCBr (AFM)

Band gap: 0.0 eV
Total magnetization: −0.0 $\mu_B/cell$
Absolute magnetization: 14.39 $\mu_B/cell$
MC2D entry: https://mc2d.materialscloud.org/#/details/mc2d-1882

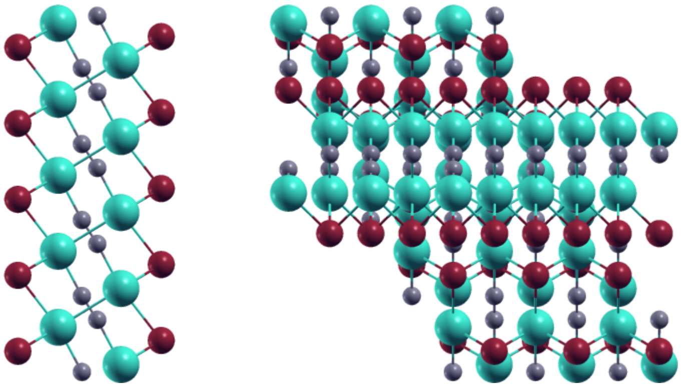

**Geometry:** Views of GdCBr as seen from the $x$ axis (left), the $y$ axis (bottom), and the $z$ axis (center).

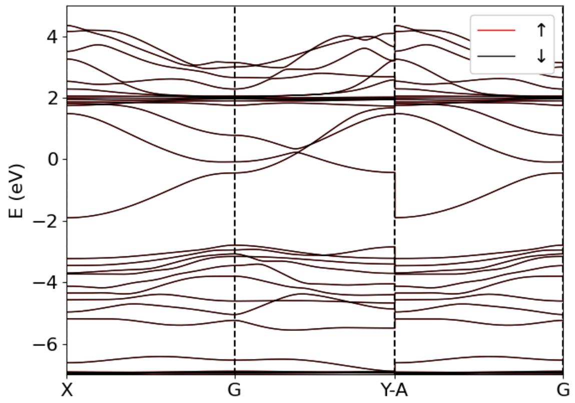


**Electronic bandstructure:** Spin-resolved energy bands of monolayer GdCBr along a high-symmetry path.

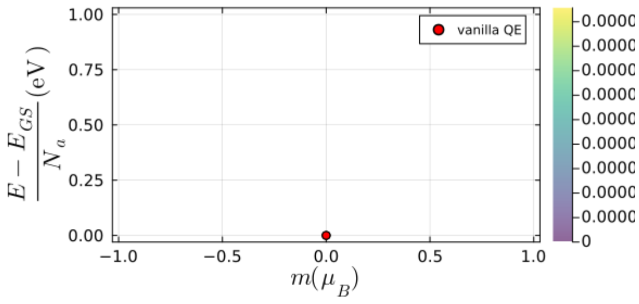


**Unique states:** Representation of 2 self-consistent unique states for monolayer GdCBr identified using RomeoDFT (see Section S6).

**Lattice vectors:** Cartesian components (in [Å]) of the lattice vectors for GdCBr.

| | $x$ | $y$ | $z$ |
|---|---|---|---|
| $a_1$ | −0.0034 | 3.8699 | 0.0000 |
| $a_2$ | 3.5588 | 1.9317 | 0.0000 |
| $a_3$ | 0.0000 | 0.0000 | 25.1618 |

**Atomic positions:** Fractional coordinates, Hubbard $U$ (in eV) and magnetic moments (in $\mu_B$, computed from orbital occupations $m_o$ or integration spheres $m_i$) of each atom of monolayer GdCBr.

| atom | $x$ | $y$ | $z$ | $U$ | $m_o$ | $m_i$ |
|---|---|---|---|---|---|---|
| Gd | 0.37 | 0.25 | 0.06 | 3.60 | 6.95 | 6.89 |
| Gd | 0.63 | −0.25 | −0.06 | 3.60 | −6.95 | −6.89 |
| C | 0.08 | −0.16 | 0.01 | 0.0 | – | −0.01 |
| Br | 0.71 | −0.43 | 0.13 | 0.0 | – | −0.02 |
| C | −0.08 | 0.16 | −0.01 | 0.0 | – | 0.01 |
| Br | 0.29 | 0.43 | −0.13 | 0.0 | – | 0.02 |

# GdCl (FM)

Band gap: 0.0 eV
Total magnetization: −15.82 $\mu_B/cell$
Absolute magnetization: 15.88 $\mu_B/cell$
MC2D entry: https://mc2d.materialscloud.org/#/details/mc2d-2517

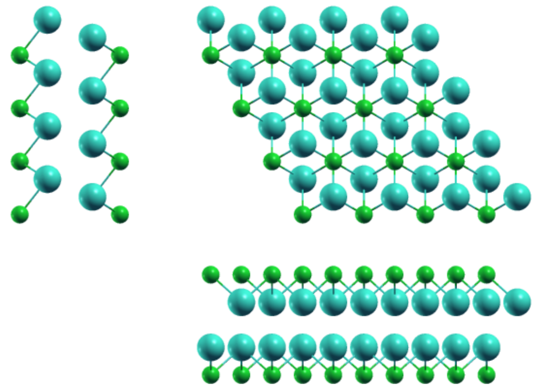

**Geometry:** Views of GdCl as seen from the $x$ axis (left), the $y$ axis (bottom), and the $z$ axis (center).

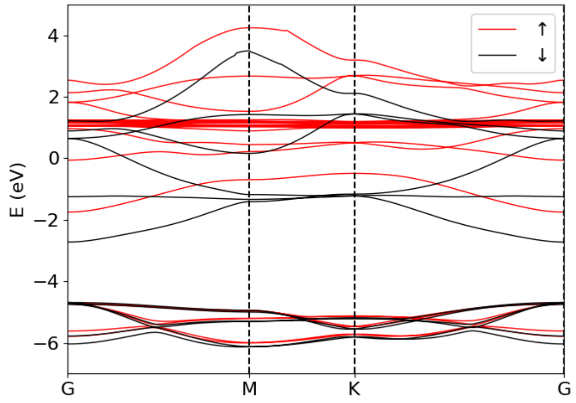


**Electronic bandstructure:** Spin-resolved energy bands of monolayer GdCl along a high-symmetry path.

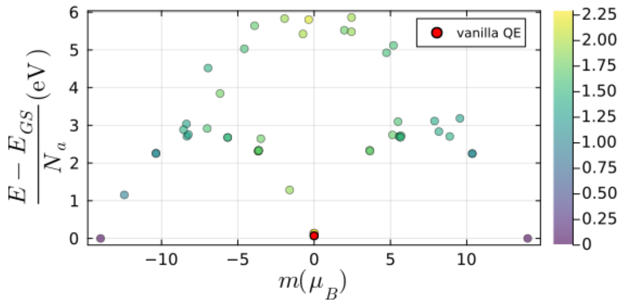


**Unique states:** Representation of 49 self-consistent unique states for monolayer GdCl identified using RomeoDFT (see Section S6).

**Lattice vectors:** Cartesian components (in [Å]) of the lattice vectors for GdCl.

| | $x$ | $y$ | $z$ |
|---|---|---|---|
| $\boldsymbol{a_1}$ | 1.9191 | −3.3240 | 0.0000 |
| $\boldsymbol{a_2}$ | 1.9191 | 3.3240 | 0.0000 |
| $\boldsymbol{a_3}$ | 0.0000 | 0.0000 | 24.6462 |

**Atomic positions:** Fractional coordinates, Hubbard $U$ (in eV) and magnetic moments (in $\mu_B$, computed from orbital occupations $m_o$ or integration spheres $m_i$) of each atom of monolayer GdCl.

| atom | $x$ | $y$ | $z$ | $U$ | $m_o$ | $m_i$ |
|---|---|---|---|---|---|---|
| Gd | 0.33 | 0.67 | 0.06 | 3.22 | −7.00 | −7.25 |
| Gd | 0.67 | 0.33 | −0.06 | 3.22 | −7.00 | −7.25 |
| Cl | 0.0 | 0.0 | 0.13 | 0.0 | – | 0.00 |
| Cl | 0.0 | 0.0 | −0.13 | 0.0 | – | 0.00 |

## $GdCl_3$ (FM)

Band gap: 3.35 eV
Total magnetization: −14.0 $\mu_B/cell$
Absolute magnetization: 14.25 $\mu_B/cell$
MC2D entry: https://mc2d.materialscloud.org/#/details/mc2d-2606

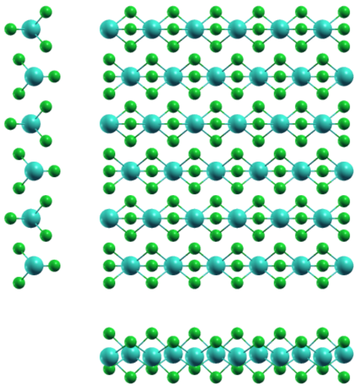

**Geometry:** Views of $GdCl_3$ as seen from the $x$ axis (left), the $y$ axis (bottom), and the $z$ axis (center).

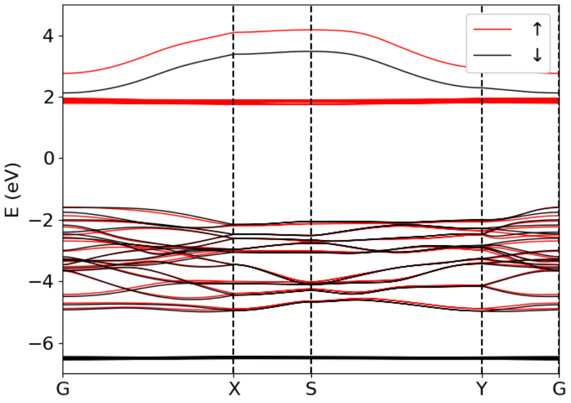


**Electronic bandstructure:** Spin-resolved energy bands of monolayer $GdCl_3$ along a high-symmetry path.

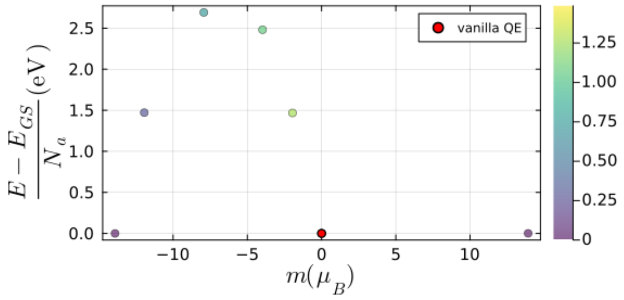


**Unique states:** Representation of 8 self-consistent unique states for monolayer $GdCl_3$ identified using RomeoDFT (see Section S6).

**Lattice vectors:** Cartesian components (in [Å]) of the lattice vectors for $GdCl_3$.

| | $x$ | $y$ | $z$ |
|---|---|---|---|
| $a_1$ | 3.8742 | 0.0000 | 0.0000 |
| $a_2$ | 0.0000 | 8.5898 | 0.0000 |
| $a_3$ | 0.0000 | 0.0000 | 19.8114 |

**Atomic positions:** Fractional coordinates, Hubbard $U$ (in eV) and magnetic moments (in $\mu_B$, computed from orbital occupations $m_o$ or integration spheres $m_i$) of each atom of monolayer $GdCl_3$.

| atom | $x$ | $y$ | $z$ | $U$ | $m_o$ | $m_i$ |
|---|---|---|---|---|---|---|
| Gd | −0.25 | −0.75 | −0.01 | 3.00 | −6.95 | −6.95 |
| Gd | 0.25 | −1.25 | 0.01 | 3.00 | −6.95 | −6.95 |
| Cl | 0.25 | −0.57 | 0.06 | 0.0 | – | 0.02 |
| Cl | 0.25 | −0.93 | 0.06 | 0.0 | – | 0.02 |
| Cl | −0.25 | −1.07 | −0.06 | 0.0 | – | 0.02 |
| Cl | −0.25 | −1.43 | −0.06 | 0.0 | – | 0.02 |
| Cl | 0.25 | −0.75 | −0.10 | 0.0 | – | 0.02 |
| Cl | −0.25 | −1.25 | 0.10 | 0.0 | – | 0.02 |

## GdGaI (AFM)

Band gap: 0.0 eV
Total magnetization: −0.0 $\mu_B/cell$
Absolute magnetization: 14.69 $\mu_B/cell$
MC2D entry: https://mc2d.materialscloud.org/#/details/mc2d-2235

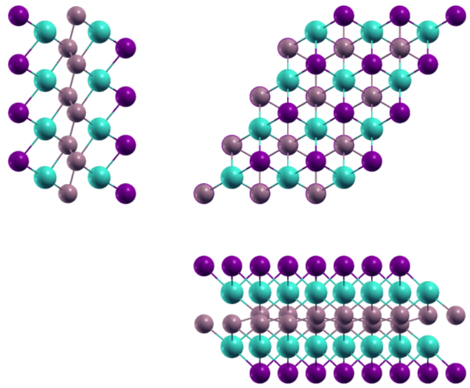

**Geometry:** Views of GdGaI as seen from the $x$ axis (left), the $y$ axis (bottom), and the $z$ axis (center).

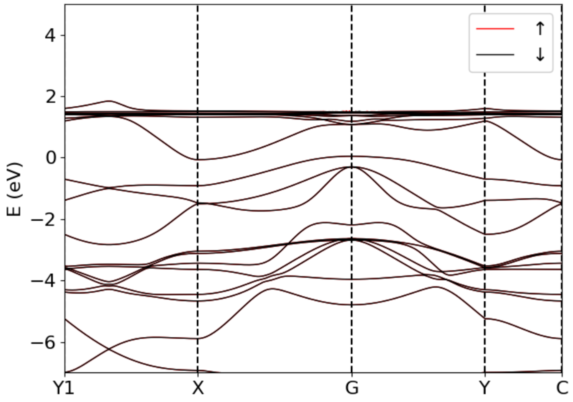


**Electronic bandstructure:** Spin-resolved energy bands of monolayer GdGaI along a high-symmetry path.

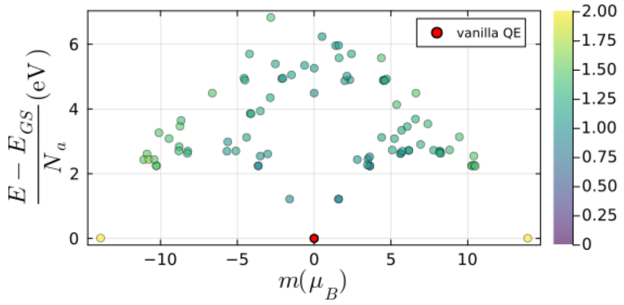


**Unique states:** Representation of 92 self-consistent unique states for monolayer GdGaI identified using RomeoDFT (see Section S6).

**Lattice vectors:** Cartesian components (in [Å]) of the lattice vectors for GdGaI.

| | $x$ | $y$ | $z$ |
|---|---|---|---|
| $\boldsymbol{a_1}$ | 2.1223 | −3.6759 | 0.0000 |
| $\boldsymbol{a_2}$ | 4.2446 | 0.0000 | 0.0000 |
| $\boldsymbol{a_3}$ | 0.0000 | 0.0000 | 28.0521 |

**Atomic positions:** Fractional coordinates, Hubbard $U$ (in eV) and magnetic moments (in $\mu_B$, computed from orbital occupations $m_o$ or integration spheres $m_i$) of each atom of monolayer GdGaI.

| atom | $x$ | $y$ | $z$ | $U$ | $m_o$ | $m_i$ |
|---|---|---|---|---|---|---|
| Gd | 0.50 | 0.50 | 0.43 | 3.20 | −6.95 | −7.09 |
| Gd | 0.50 | 0.50 | 0.57 | 3.20 | 6.95 | 7.09 |
| Ga | 0.17 | 0.17 | 0.51 | 0.0 | – | −0.01 |
| Ga | 0.83 | 0.83 | 0.49 | 0.0 | – | 0.01 |
| I | 0.83 | 0.83 | 0.64 | 0.0 | – | −0.03 |
| I | 0.17 | 0.17 | 0.36 | 0.0 | – | 0.03 |

## GdI (FM)

Band gap: 0.0 eV
Total magnetization: 15.79 $\mu_B/cell$
Absolute magnetization: 15.93 $\mu_B/cell$
MC2D entry: https://mc2d.materialscloud.org/#/details/mc2d-2602

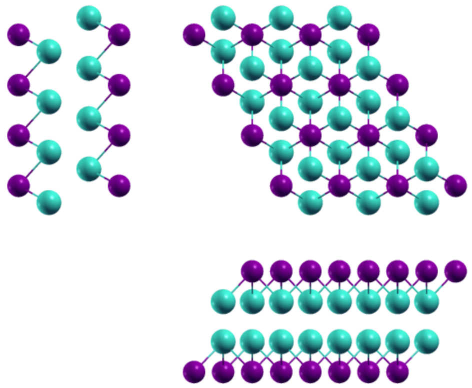

**Geometry:** Views of GdI as seen from the $x$ axis (left), the $y$ axis (bottom), and the $z$ axis (center).

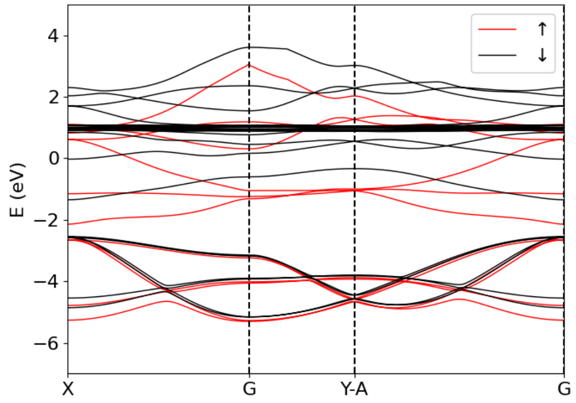


**Electronic bandstructure:** Spin-resolved energy bands of monolayer GdI along a high-symmetry path.

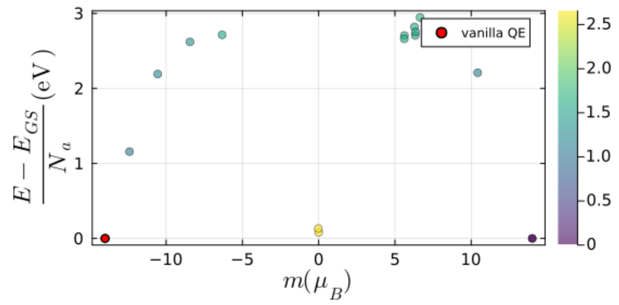


**Unique states:** Representation of 16 self-consistent unique states for monolayer GdI identified using RomeoDFT (see Section S6).

**Lattice vectors:** Cartesian components (in [Å]) of the lattice vectors for GdI.

| | $x$ | $y$ | $z$ |
|---|---|---|---|
| $\boldsymbol{a_1}$ | 2.0242 | −3.5060 | 0.0000 |
| $\boldsymbol{a_2}$ | 2.0242 | 3.5061 | 0.0000 |
| $\boldsymbol{a_3}$ | 0.0000 | 0.0000 | 26.2526 |

**Atomic positions:** Fractional coordinates, Hubbard $U$ (in eV) and magnetic moments (in $\mu_B$, computed from orbital occupations $m_o$ or integration spheres $m_i$) of each atom of monolayer GdI.

| atom | $x$ | $y$ | $z$ | $U$ | $m_o$ | $m_i$ |
|---|---|---|---|---|---|---|
| Gd | 0.33 | 0.17 | 0.05 | 2.94 | 7.00 | 7.36 |
| Gd | 0.67 | 0.83 | −0.05 | 2.94 | 7.00 | 7.36 |
| I | 0.00 | 0.50 | 0.13 | 0.0 | – | −0.02 |
| I | 0.00 | 0.50 | −0.13 | 0.0 | – | −0.02 |

## $GdI_2$ (FM)

Band gap: 0.56 eV
Total magnetization: 16.0 $\mu_B/cell$
Absolute magnetization: 16.28 $\mu_B/cell$
MC2D entry: https://mc2d.materialscloud.org/#/details/mc2d-2039

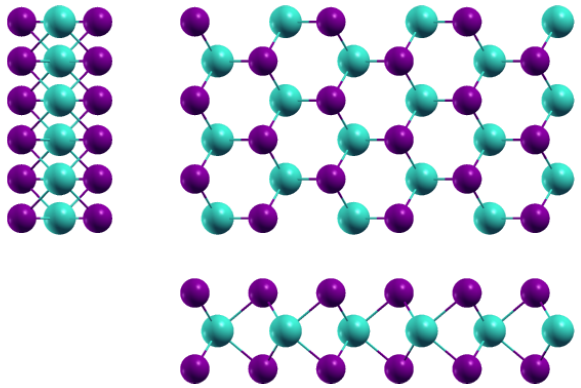

**Geometry:** Views of $GdI_2$ as seen from the $x$ axis (left), the $y$ axis (bottom), and the $z$ axis (center).

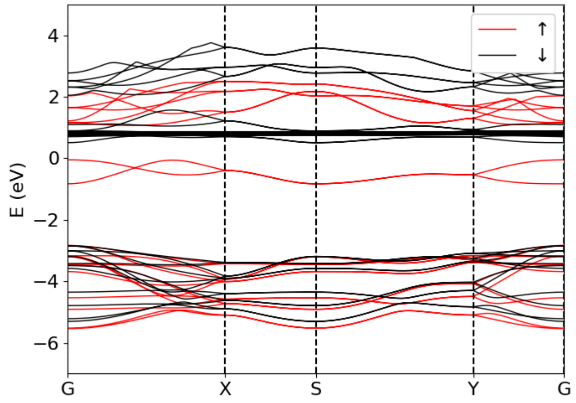


**Electronic bandstructure:** Spin-resolved energy bands of monolayer $GdI_2$ along a high-symmetry path.

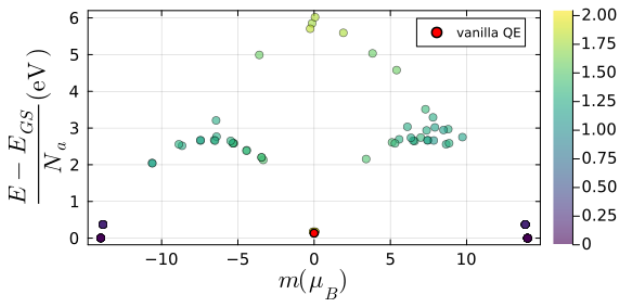


**Unique states:** Representation of 52 self-consistent unique states for monolayer $GdI_2$ identified using RomeoDFT (see Section S6).

**Lattice vectors:** Cartesian components (in [Å]) of the lattice vectors for $GdI_2$.

| | $x$ | $y$ | $z$ |
|---|---|---|---|
| $\boldsymbol{a_1}$ | −6.2797 | −3.6256 | 0.0000 |
| $\boldsymbol{a_2}$ | 2.0932 | −3.6256 | 0.0000 |
| $\boldsymbol{a_3}$ | 0.0000 | 0.0000 | 19.9386 |

**Atomic positions:** Fractional coordinates, Hubbard $U$ (in eV) and magnetic moments (in $\mu_B$, computed from orbital occupations $m_o$ or integration spheres $m_i$) of each atom of monolayer $GdI_2$.

| atom | $x$ | $y$ | $z$ | $U$ | $m_o$ | $m_i$ |
|---|---|---|---|---|---|---|
| Gd | 0.83 | 0.50 | 0.0 | 3.06 | 7.00 | 7.42 |
| Gd | 0.33 | 0.0 | 0.0 | 3.06 | 7.00 | 7.42 |
| I | 0.67 | 0.0 | 0.90 | 0.0 | – | −0.02 |
| I | 1.17 | 0.50 | 0.90 | 0.0 | – | −0.02 |
| I | 0.17 | 0.50 | 0.10 | 0.0 | – | −0.02 |
| I | 0.67 | 0.0 | 0.10 | 0.0 | – | −0.02 |

## $GdI_3$ (AFM)

Band gap: 2.75 eV
Total magnetization: $-0.0\ \mu_B/cell$
Absolute magnetization: $14.32\ \mu_B/cell$
MC2D entry: https://mc2d.materialscloud.org/#/details/mc2d-1852

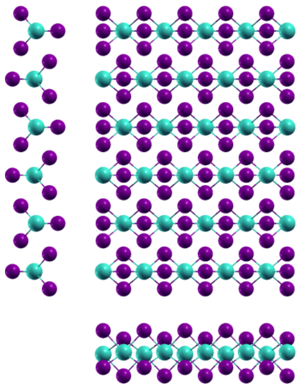

**Geometry:** Views of $GdI_3$ as seen from the $x$ axis (left), the $y$ axis (bottom), and the $z$ axis (center).

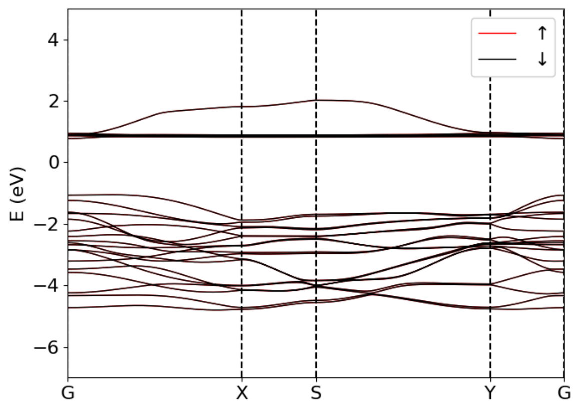


**Electronic bandstructure:** Spin-resolved energy bands of monolayer $GdI_3$ along a high-symmetry path.

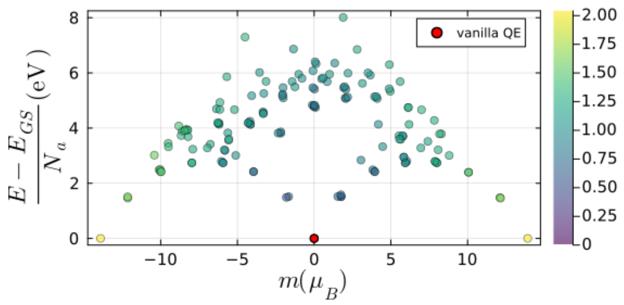


**Unique states:** Representation of 147 self-consistent unique states for monolayer $GdI_3$ identified using RomeoDFT (see Section S6).

**Lattice vectors:** Cartesian components (in [Å]) of the lattice vectors for $GdI_3$.

| | $x$ | $y$ | $z$ |
|---|---|---|---|
| $\boldsymbol{a_1}$ | 3.8844 | −6.7279 | 0.0000 |
| $\boldsymbol{a_2}$ | 7.7687 | 0.0000 | 0.0000 |
| $\boldsymbol{a_3}$ | 0.0000 | 0.0000 | 19.0453 |

**Atomic positions:** Fractional coordinates, Hubbard $U$ (in eV) and magnetic moments (in $\mu_B$, computed from orbital occupations $m_o$ or integration spheres $m_i$) of each atom of monolayer $GdI_3$.

| atom | $x$ | $y$ | $z$ | $U$ | $m_o$ | $m_i$ |
|---|---|---|---|---|---|---|
| Gd | −0.33 | −0.33 | 0.0 | 3.28 | 6.96 | 7.01 |
| Gd | −0.67 | 0.33 | 0.0 | 3.28 | −6.96 | −7.01 |
| I | −1.00 | 0.64 | 0.09 | 0.0 | – | 0.0 |
| I | −0.36 | 0.0 | −0.09 | 0.0 | – | 0.0 |
| I | −0.64 | 0.64 | −0.09 | 0.0 | – | 0.0 |
| I | −1.00 | 0.36 | −0.09 | 0.0 | – | 0.0 |
| I | −0.64 | 0.0 | 0.09 | 0.0 | – | 0.0 |
| I | −0.36 | 0.36 | 0.09 | 0.0 | – | 0.0 |

# GdOBr (AFM)

Band gap: 4.21 eV
Total magnetization: 0.0 $\mu_B/cell$
Absolute magnetization: 14.15 $\mu_B/cell$
MC2D entry: https://mc2d.materialscloud.org/#/details/mc2d-2431

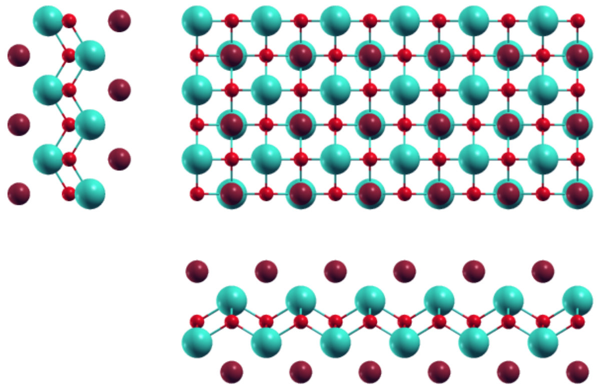

**Geometry:** Views of GdOBr as seen from the $x$ axis (left), the $y$ axis (bottom), and the $z$ axis (center).

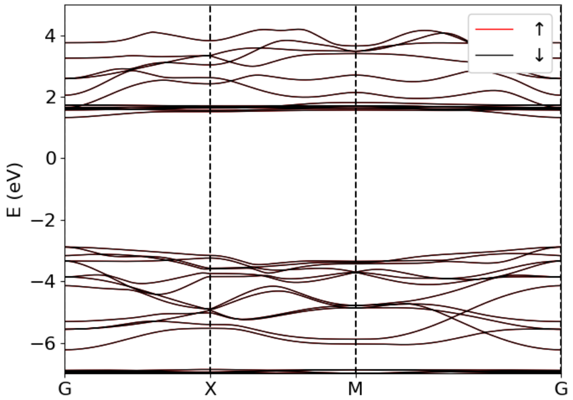


**Electronic bandstructure:** Spin-resolved energy bands of monolayer GdOBr along a high-symmetry path.

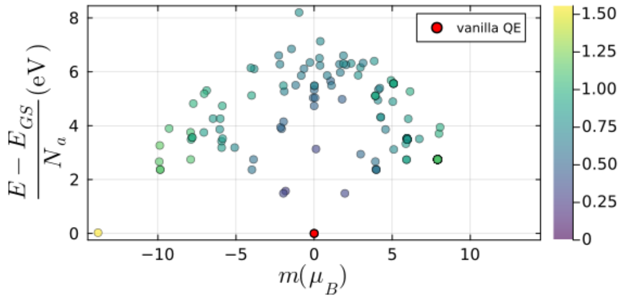


**Unique states:** Representation of 92 self-consistent unique states for monolayer GdOBr identified using RomeoDFT (see Section S6).

**Lattice vectors:** Cartesian components (in [Å]) of the lattice vectors for GdOBr.

| | $x$ | $y$ | $z$ |
|---|---|---|---|
| $\boldsymbol{a_1}$ | 3.9047 | 0.0000 | 0.0000 |
| $\boldsymbol{a_2}$ | 0.0000 | 3.9047 | 0.0000 |
| $\boldsymbol{a_3}$ | 0.0000 | 0.0000 | 22.9017 |

**Atomic positions:** Fractional coordinates, Hubbard $U$ (in eV) and magnetic moments (in $\mu_B$, computed from orbital occupations $m_o$ or integration spheres $m_i$) of each atom of monolayer GdOBr.

| atom | $x$ | $y$ | $z$ | $U$ | $m_o$ | $m_i$ |
|---|---|---|---|---|---|---|
| Gd | 0.25 | −0.25 | 0.05 | 3.27 | 6.92 | 6.83 |
| Gd | −0.25 | −0.75 | −0.05 | 3.27 | −6.92 | −6.83 |
| Br | −0.25 | −0.75 | 0.12 | 0.0 | – | −0.01 |
| Br | 0.25 | −0.25 | −0.12 | 0.0 | – | 0.01 |
| O | 0.25 | −0.75 | 0.0 | 0.0 | – | 0.0 |
| O | −0.25 | −0.25 | 0.0 | 0.0 | – | 0.0 |

# GdSI (AFM)

Band gap: 2.72 eV
Total magnetization: −0.0 $\mu_B/cell$
Absolute magnetization: 14.27 $\mu_B/cell$
MC2D entry: https://mc2d.materialscloud.org/#/details/mc2d-2697

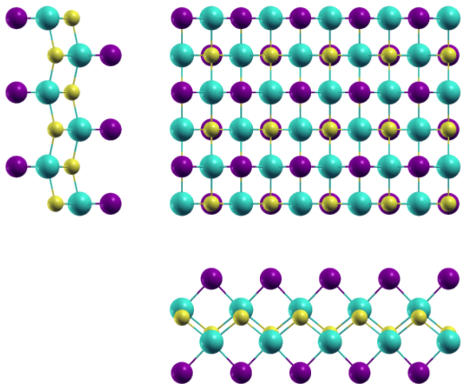

**Geometry:** Views of GdSI as seen from the $x$ axis (left), the $y$ axis (bottom), and the $z$ axis (center).

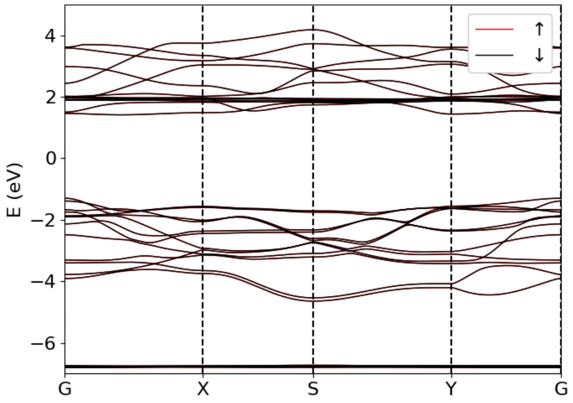


**Electronic bandstructure:** Spin-resolved energy bands of monolayer GdSI along a high-symmetry path.

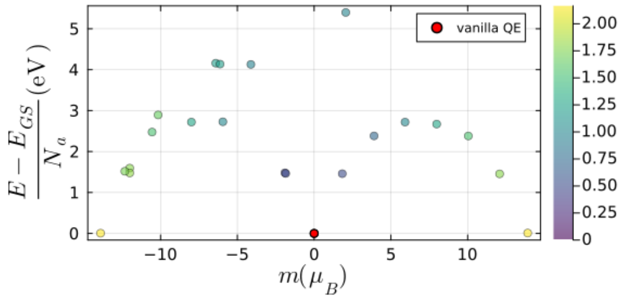


**Unique states:** Representation of 24 self-consistent unique states for monolayer GdSI identified using RomeoDFT (see Section S6).

**Lattice vectors:** Cartesian components (in [Å]) of the lattice vectors for GdSI.

| | $x$ | $y$ | $z$ |
|---|---|---|---|
| $a_1$ | 4.2559 | 0.0000 | 0.0000 |
| $a_2$ | 0.0000 | 5.3350 | 0.0000 |
| $a_3$ | 0.0000 | 0.0000 | 25.3026 |

**Atomic positions:** Fractional coordinates, Hubbard $U$ (in eV) and magnetic moments (in $\mu_B$, computed from orbital occupations $m_o$ or integration spheres $m_i$) of each atom of monolayer GdSI.

| atom | $x$ | $y$ | $z$ | $U$ | $m_o$ | $m_i$ |
|---|---|---|---|---|---|---|
| Gd | 0.25 | −0.75 | −0.05 | 3.38 | 6.95 | 6.96 |
| Gd | −0.25 | −0.25 | 0.05 | 3.38 | −6.95 | −6.96 |
| S | 0.25 | −0.25 | −0.02 | 0.0 | – | 0.00 |
| I | −0.25 | −0.75 | −0.13 | 0.0 | – | −0.02 |
| S | −0.25 | −0.75 | 0.02 | 0.0 | – | 0.00 |
| I | 0.25 | −0.25 | 0.13 | 0.0 | – | 0.02 |

## GdSeI (AFM)

Band gap: 2.35 eV
Total magnetization: −0.0 $\mu_B/cell$
Absolute magnetization: 14.3 $\mu_B/cell$
MC2D entry: https://mc2d.materialscloud.org/#/details/mc2d-1900

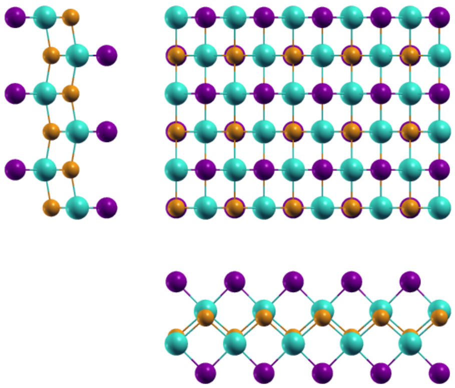

**Geometry:** Views of GdSeI as seen from the $x$ axis (left), the $y$ axis (bottom), and the $z$ axis (center).

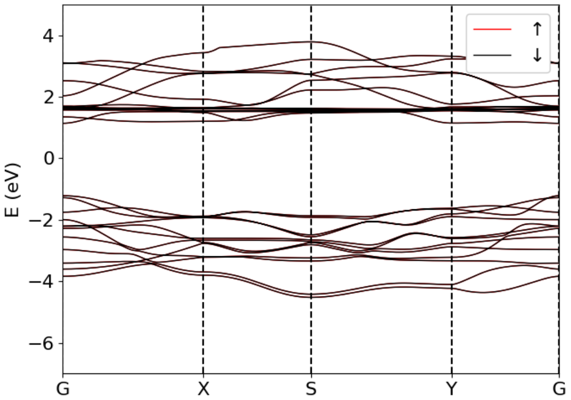


**Electronic bandstructure:** Spin-resolved energy bands of monolayer GdSeI along a high-symmetry path.

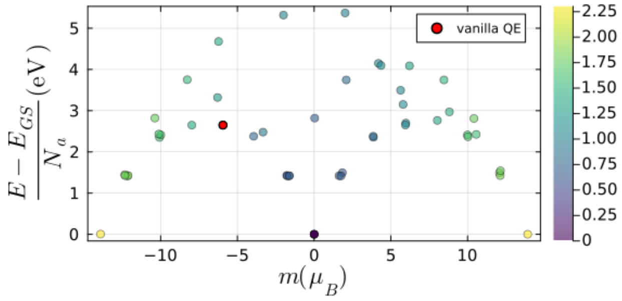


**Unique states:** Representation of 50 self-consistent unique states for monolayer GdSeI identified using RomeoDFT (see Section S6).

**Lattice vectors:** Cartesian components (in [Å]) of the lattice vectors for GdSeI.

| | $x$ | $y$ | $z$ |
|---|---|---|---|
| $a_1$ | 4.3263 | 0.0000 | 0.0000 |
| $a_2$ | 0.0000 | 5.6371 | 0.0000 |
| $a_3$ | 0.0000 | 0.0000 | 25.2438 |

**Atomic positions:** Fractional coordinates, Hubbard $U$ (in eV) and magnetic moments (in $\mu_B$, computed from orbital occupations $m_o$ or integration spheres $m_i$) of each atom of monolayer GdSeI.

| atom | $x$ | $y$ | $z$ | $U$ | $m_o$ | $m_i$ |
|---|---|---|---|---|---|---|
| Gd | −0.25 | −0.75 | −0.05 | 3.43 | 6.96 | 6.98 |
| Gd | 0.25 | −0.25 | 0.05 | 3.43 | −6.96 | −6.98 |
| I | 0.25 | −0.75 | −0.13 | 0.0 | – | −0.02 |
| Se | −0.25 | −0.25 | −0.03 | 0.0 | – | 0.00 |
| Se | 0.25 | −0.75 | 0.03 | 0.0 | – | 0.00 |
| I | −0.25 | −0.25 | 0.13 | 0.0 | – | 0.02 |

## $HfFeCl_6$ (FM)

Band gap: 3.82 eV
Total magnetization: 8.0 $\mu_B/cell$
Absolute magnetization: 8.05 $\mu_B/cell$
MC2D entry: https://mc2d.materialscloud.org/#/details/mc2d-692

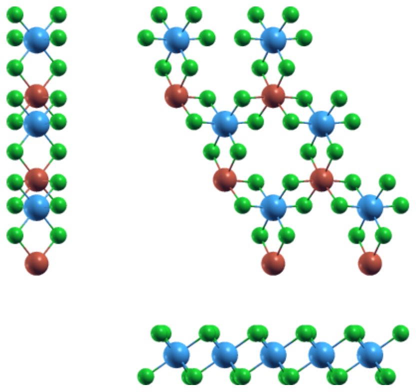

**Geometry:** Views of $HfFeCl_6$ as seen from the $x$ axis (left), the $y$ axis (bottom), and the $z$ axis (center).

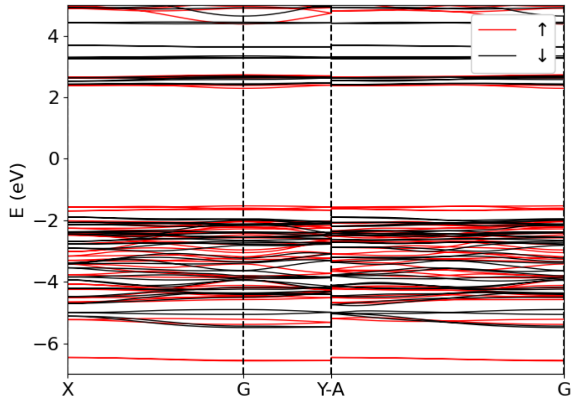


**Electronic bandstructure:** Spin-resolved energy bands of monolayer $HfFeCl_6$ along a high-symmetry path.

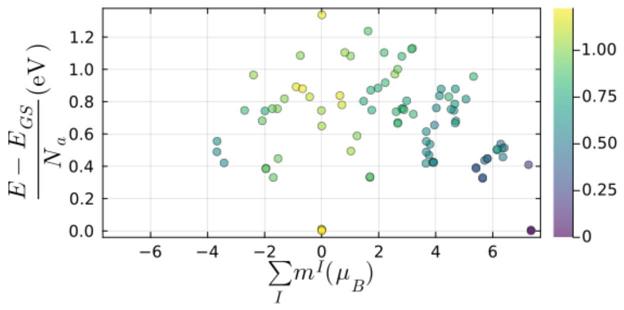


**Unique states:** Representation of 91 self-consistent unique states for monolayer $HfFeCl_6$ identified using RomeoDFT (see Section S6).

**Lattice vectors:** Cartesian components (in [Å]) of the lattice vectors for $HfFeCl_6$.

| | $x$ | $y$ | $z$ |
|---|---|---|---|
| $\boldsymbol{a_1}$ | 12.7071 | 0.0000 | 0.0000 |
| $\boldsymbol{a_2}$ | −3.1768 | 5.5024 | 0.0000 |
| $\boldsymbol{a_3}$ | 0.0000 | 0.0000 | 22.7705 |

**Atomic positions:** Fractional coordinates, Hubbard $U$ (in eV) and magnetic moments (in $\mu_B$, computed from orbital occupations $m_o$ or integration spheres $m_i$) of each atom of monolayer $HfFeCl_6$.

| atom | $x$ | $y$ | $z$ | $U$ | $m_o$ | $m_i$ |
|---|---|---|---|---|---|---|
| Hf | 0.17 | 0.67 | 0.50 | 1.74 | 0.00 | 0.00 |
| Fe | 0.0 | 0.0 | 0.50 | 7.51 | 3.67 | 3.68 |
| Hf | 0.67 | 0.67 | 0.50 | 1.74 | 0.00 | 0.00 |
| Fe | 0.50 | 0.0 | 0.50 | 7.51 | 3.67 | 3.68 |
| Cl | 0.15 | −0.03 | 0.44 | 0.0 | – | 0.01 |
| Cl | 0.52 | 0.34 | 0.44 | 0.0 | – | 0.01 |
| Cl | 0.33 | 0.69 | 0.44 | 0.0 | – | 0.02 |
| Cl | 0.33 | −0.03 | 0.56 | 0.0 | – | 0.02 |
| Cl | 0.52 | 0.69 | 0.56 | 0.0 | – | 0.01 |
| Cl | 0.15 | 0.34 | 0.56 | 0.0 | – | 0.01 |
| Cl | 0.65 | −0.03 | 0.44 | 0.0 | – | 0.01 |
| Cl | 1.02 | 0.34 | 0.44 | 0.0 | – | 0.01 |
| Cl | 0.83 | 0.69 | 0.44 | 0.0 | – | 0.02 |
| Cl | 0.83 | −0.03 | 0.56 | 0.0 | – | 0.02 |
| Cl | 1.02 | 0.69 | 0.56 | 0.0 | – | 0.01 |
| Cl | 0.65 | 0.34 | 0.56 | 0.0 | – | 0.01 |

## $HoBr_3$ (AFM)

Band gap: 1.17 eV
Total magnetization: $-0.0\ \mu_B/cell$
Absolute magnetization: 8.13 $\mu_B/cell$
MC2D entry: https://mc2d.materialscloud.org/#/details/mc2d-2490

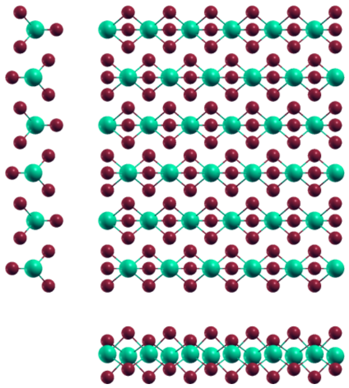

**Geometry:** Views of $HoBr_3$ as seen from the $x$ axis (left), the $y$ axis (bottom), and the $z$ axis (center).

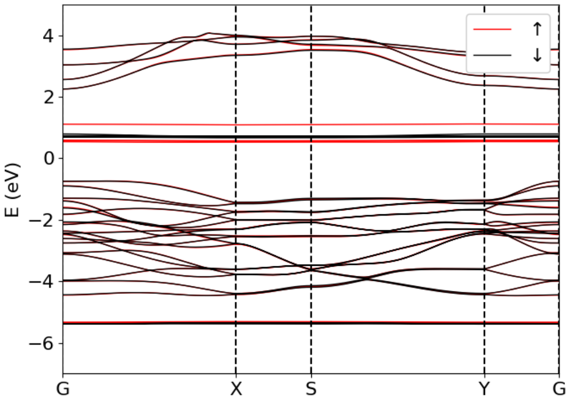


**Electronic bandstructure:** Spin-resolved energy bands of monolayer $HoBr_3$ along a high-symmetry path.

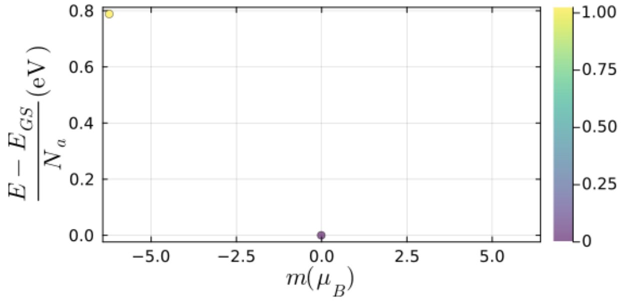


**Unique states:** Representation of 3 self-consistent unique states for monolayer $HoBr_3$ identified using RomeoDFT (see Section S6).

**Lattice vectors:** Cartesian components (in [Å]) of the lattice vectors for $HoBr_3$.

| | $x$ | $y$ | $z$ |
|---|---|---|---|
| $\boldsymbol{a_1}$ | 3.9613 | 0.0000 | 0.0000 |
| $\boldsymbol{a_2}$ | 0.0000 | 9.1392 | 0.0000 |
| $\boldsymbol{a_3}$ | 0.0000 | 0.0000 | 20.4015 |

**Atomic positions:** Fractional coordinates, Hubbard $U$ (in eV) and magnetic moments (in $\mu_B$, computed from orbital occupations $m_o$ or integration spheres $m_i$) of each atom of monolayer $HoBr_3$.

| atom | $x$ | $y$ | $z$ | $U$ | $m_o$ | $m_i$ |
|---|---|---|---|---|---|---|
| Ho | 0.25 | −1.25 | 0.00 | 4.74 | 3.96 | 3.99 |
| Ho | −0.25 | −0.75 | 0.00 | 4.74 | −3.97 | −4.00 |
| Br | −0.25 | −1.43 | 0.07 | 0.0 | – | 0.00 |
| Br | −0.25 | −1.07 | 0.07 | 0.0 | – | 0.00 |
| Br | 0.25 | −0.93 | −0.07 | 0.0 | – | 0.01 |
| Br | 0.25 | −0.57 | −0.07 | 0.0 | – | 0.01 |
| Br | −0.25 | −1.25 | −0.10 | 0.0 | – | 0.00 |
| Br | 0.25 | −0.75 | 0.10 | 0.0 | – | 0.01 |

## $HoCl_3$ (AFM)

Band gap: 2.26 eV
Total magnetization: 0.0 $\mu_B/cell$
Absolute magnetization: 8.11 $\mu_B/cell$
MC2D entry: https://mc2d.materialscloud.org/#/details/mc2d-2647

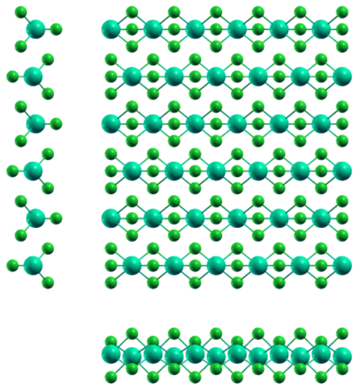

**Geometry:** Views of $HoCl_3$ as seen from the $x$ axis (left), the $y$ axis (bottom), and the $z$ axis (center).

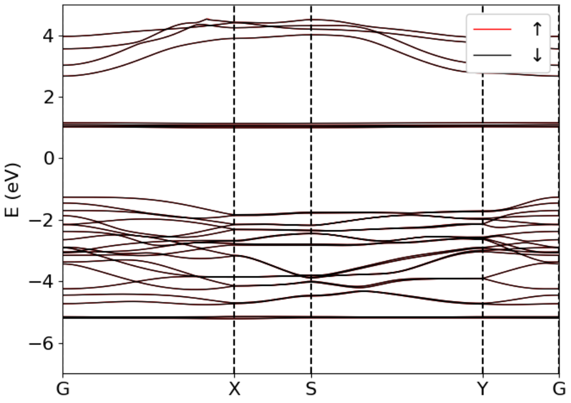


**Electronic bandstructure:** Spin-resolved energy bands of monolayer $HoCl_3$ along a high-symmetry path.

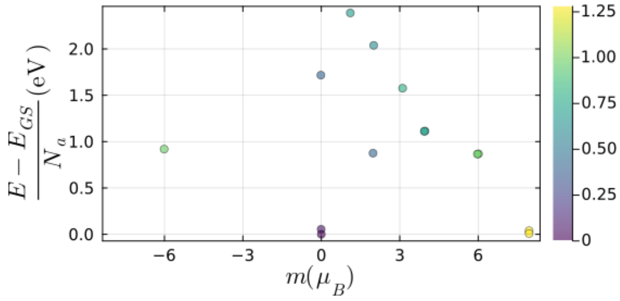


**Unique states:** Representation of 13 self-consistent unique states for monolayer $HoCl_3$ identified using RomeoDFT (see Section S6).

**Lattice vectors:** Cartesian components (in [Å]) of the lattice vectors for $HoCl_3$.

| | $x$ | $y$ | $z$ |
|---|---|---|---|
| $\boldsymbol{a_1}$ | 3.8153 | 0.0000 | 0.0000 |
| $\boldsymbol{a_2}$ | 0.0000 | 8.5696 | 0.0000 |
| $\boldsymbol{a_3}$ | 0.0000 | 0.0000 | 19.7828 |

**Atomic positions:** Fractional coordinates, Hubbard $U$ (in eV) and magnetic moments (in $\mu_B$, computed from orbital occupations $m_o$ or integration spheres $m_i$) of each atom of monolayer $HoCl_3$.

| atom | $x$ | $y$ | $z$ | $U$ | $m_o$ | $m_i$ |
|---|---|---|---|---|---|---|
| Ho | 0.25 | −0.25 | −0.51 | 4.87 | 3.97 | 3.98 |
| Ho | −0.25 | −0.75 | −0.49 | 4.87 | −3.97 | −3.98 |
| Cl | −0.25 | −0.07 | −0.44 | 0.0 | – | 0.00 |
| Cl | −0.25 | −0.43 | −0.44 | 0.0 | – | 0.00 |
| Cl | 0.25 | −0.57 | −0.56 | 0.0 | – | 0.00 |
| Cl | 0.25 | −0.93 | −0.56 | 0.0 | – | 0.00 |
| Cl | −0.25 | −0.25 | −0.60 | 0.0 | – | −0.01 |
| Cl | 0.25 | −0.75 | −0.40 | 0.0 | – | 0.01 |

## $HoI_3$ (FM)

Band gap: 2.72 eV
Total magnetization: 8.0 $\mu_B/cell$
Absolute magnetization: 8.28 $\mu_B/cell$
MC2D entry: https://mc2d.materialscloud.org/#/details/mc2d-2532

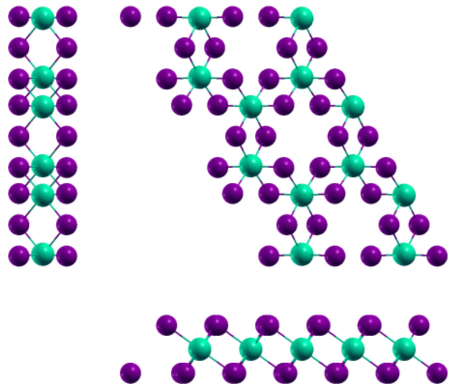

**Geometry:** Views of $HoI_3$ as seen from the $x$ axis (left), the $y$ axis (bottom), and the $z$ axis (center).

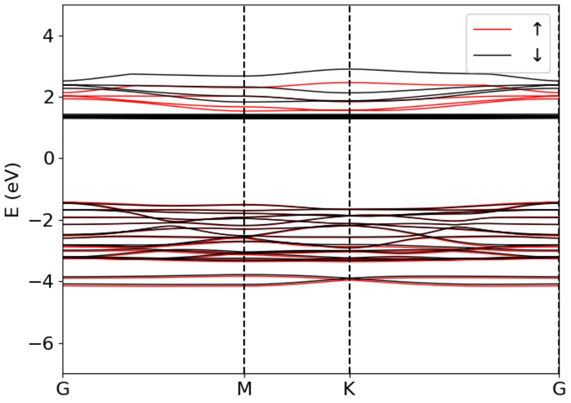


**Electronic bandstructure:** Spin-resolved energy bands of monolayer $HoI_3$ along a high-symmetry path.

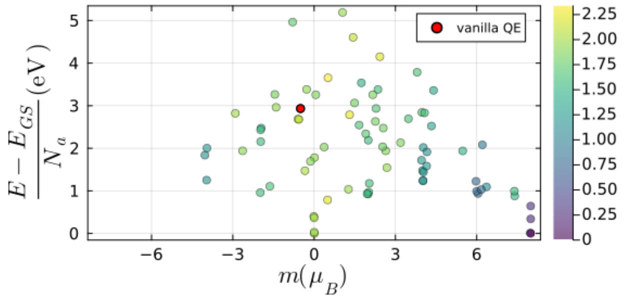


**Unique states:** Representation of 78 self-consistent unique states for monolayer $HoI_3$ identified using RomeoDFT (see Section S6).

**Lattice vectors:** Cartesian components (in [Å]) of the lattice vectors for $HoI_3$.

| | $x$ | $y$ | $z$ |
|---|---|---|---|
| $a_1$ | −3.8196 | 6.6157 | 0.0000 |
| $a_2$ | −3.8196 | −6.6157 | 0.0000 |
| $a_3$ | 0.0000 | 0.0000 | 19.0253 |

**Atomic positions:** Fractional coordinates, Hubbard $U$ (in eV) and magnetic moments (in $\mu_B$, computed from orbital occupations $m_o$ or integration spheres $m_i$) of each atom of monolayer $HoI_3$.

| atom | $x$ | $y$ | $z$ | $U$ | $m_o$ | $m_i$ |
|---|---|---|---|---|---|---|
| Ho | −0.33 | −0.17 | 0.0 | 7.69 | 3.99 | 4.03 |
| Ho | 0.33 | 0.17 | 0.0 | 7.69 | 4.00 | 4.03 |
| I | 0.65 | 0.50 | −0.09 | 0.0 | – | −0.02 |
| I | 0.0 | −0.15 | 0.09 | 0.0 | – | −0.02 |
| I | 0.65 | 0.15 | 0.09 | 0.0 | – | −0.02 |
| I | 0.35 | 0.50 | 0.09 | 0.0 | – | −0.02 |
| I | 0.0 | 0.15 | −0.09 | 0.0 | – | −0.02 |
| I | 0.35 | −0.15 | −0.09 | 0.0 | – | −0.02 |

# HoOBr (FM)

Band gap: 2.98 eV
Total magnetization: 8.0 $\mu_B/cell$
Absolute magnetization: 8.06 $\mu_B/cell$
MC2D entry: https://mc2d.materialscloud.org/#/details/mc2d-2236

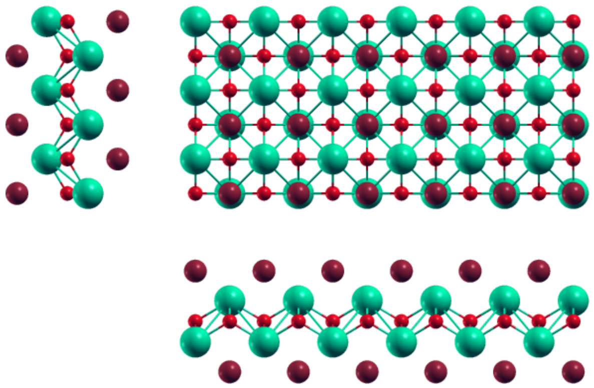

**Geometry:** Views of HoOBr as seen from the $x$ axis (left), the $y$ axis (bottom), and the $z$ axis (center).

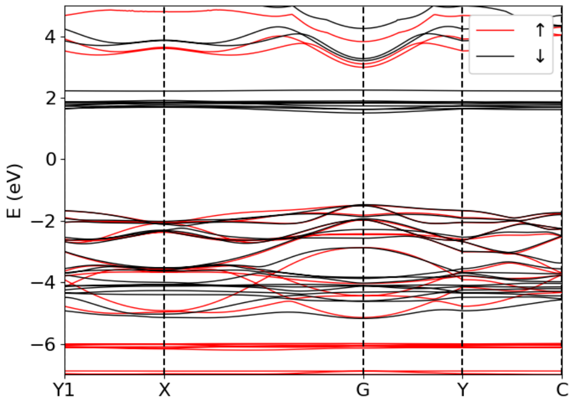


**Electronic bandstructure:** Spin-resolved energy bands of monolayer HoOBr along a high-symmetry path.

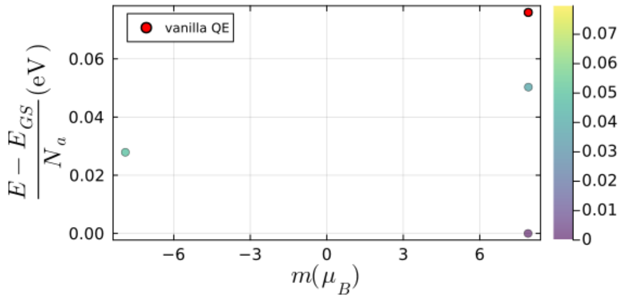


**Unique states:** Representation of 3 self-consistent unique states for monolayer HoOBr identified using RomeoDFT (see Section S6).

**Lattice vectors:** Cartesian components (in [Å]) of the lattice vectors for HoOBr.

| | $x$ | $y$ | $z$ |
|---|---|---|---|
| $\boldsymbol{a_1}$ | 3.8325 | 0.0003 | 0.0000 |
| $\boldsymbol{a_2}$ | 0.0003 | 3.8325 | 0.0000 |
| $\boldsymbol{a_3}$ | 0.0000 | 0.0000 | 22.9223 |

**Atomic positions:** Fractional coordinates, Hubbard $U$ (in eV) and magnetic moments (in $\mu_B$, computed from orbital occupations $m_o$ or integration spheres $m_i$) of each atom of monolayer HoOBr.

| atom | $x$ | $y$ | $z$ | $U$ | $m_o$ | $m_i$ |
|---|---|---|---|---|---|---|
| Ho | 0.25 | −0.25 | 0.05 | 4.62 | 3.94 | 3.93 |
| Ho | −0.25 | −0.75 | −0.05 | 4.62 | 3.95 | 3.93 |
| Br | −0.25 | −0.75 | 0.12 | 0.0 | – | 0.00 |
| Br | 0.25 | −0.25 | −0.12 | 0.0 | – | −0.01 |
| O | −0.25 | −0.25 | 0.0 | 0.0 | – | 0.00 |
| O | 0.25 | −0.75 | 0.0 | 0.0 | – | 0.00 |

# HoSI (AFM)

Band gap: 1.97 eV
Total magnetization: $-0.0\ \mu_B/cell$
Absolute magnetization: $8.1\ \mu_B/cell$
MC2D entry: https://mc2d.materialscloud.org/#/details/mc2d-175

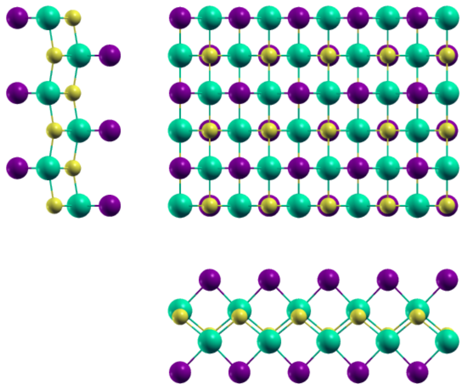

**Geometry:** Views of HoSI as seen from the $x$ axis (left), the $y$ axis (bottom), and the $z$ axis (center).

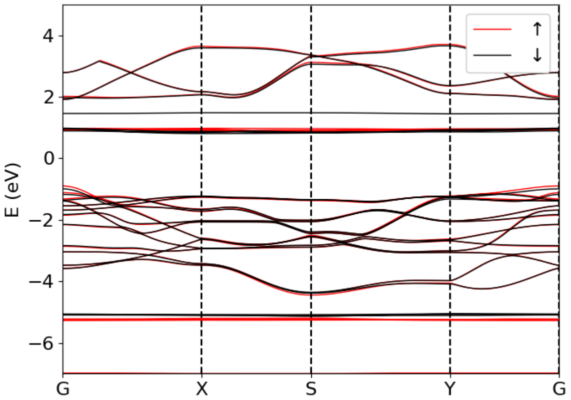


**Electronic bandstructure:** Spin-resolved energy bands of monolayer HoSI along a high-symmetry path.

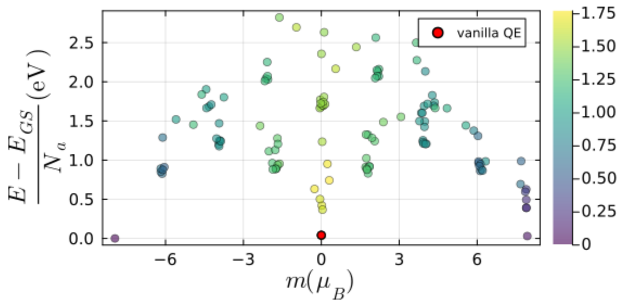


**Unique states:** Representation of 124 self-consistent unique states for monolayer HoSI identified using RomeoDFT (see Section S6).

**Lattice vectors:** Cartesian components (in [Å]) of the lattice vectors for HoSI.

| | $x$ | $y$ | $z$ |
|---|---|---|---|
| $\boldsymbol{a_1}$ | 4.2322 | 0.0000 | 0.0000 |
| $\boldsymbol{a_2}$ | 0.0000 | 5.3604 | 0.0000 |
| $\boldsymbol{a_3}$ | 0.0000 | 0.0000 | 25.1969 |

**Atomic positions:** Fractional coordinates, Hubbard $U$ (in eV) and magnetic moments (in $\mu_B$, computed from orbital occupations $m_o$ or integration spheres $m_i$) of each atom of monolayer HoSI.

| atom | $x$ | $y$ | $z$ | $U$ | $m_o$ | $m_i$ |
|---|---|---|---|---|---|---|
| Ho | 0.25 | −0.75 | −0.54 | 4.84 | 3.95 | 3.97 |
| Ho | −0.25 | −0.25 | −0.46 | 4.84 | −3.97 | −3.99 |
| S | 0.25 | −0.25 | −0.53 | 0.0 | – | 0.00 |
| I | −0.25 | −0.75 | −0.63 | 0.0 | – | −0.01 |
| S | −0.25 | −0.75 | −0.47 | 0.0 | – | 0.00 |
| I | 0.25 | −0.25 | −0.37 | 0.0 | – | 0.01 |

## HoSeI (AFM)

Band gap: 1.84 eV
Total magnetization: −0.0 $\mu_B/cell$
Absolute magnetization: 8.16 $\mu_B/cell$
MC2D entry: https://mc2d.materialscloud.org/#/details/mc2d-2493

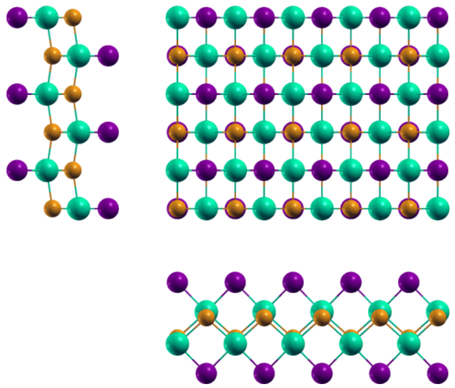

**Geometry:** Views of HoSeI as seen from the $x$ axis (left), the $y$ axis (bottom), and the $z$ axis (center).

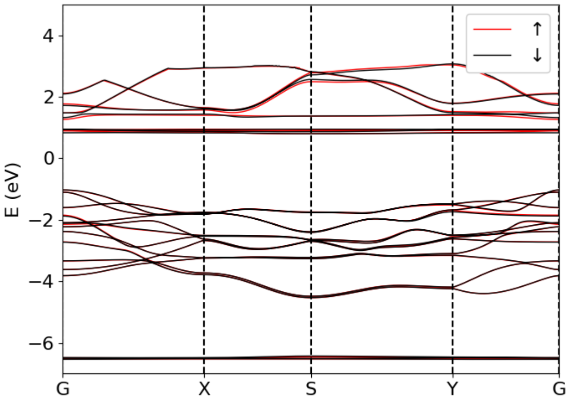


**Electronic bandstructure:** Spin-resolved energy bands of monolayer HoSeI along a high-symmetry path.

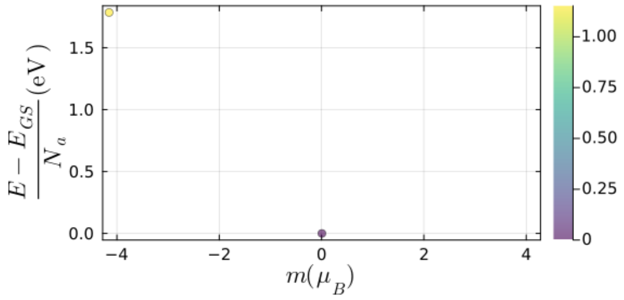


**Unique states:** Representation of 3 self-consistent unique states for monolayer HoSeI identified using RomeoDFT (see Section S6).

**Lattice vectors:** Cartesian components (in [Å]) of the lattice vectors for HoSeI.

| | $x$ | $y$ | $z$ |
|---|---|---|---|
| $\boldsymbol{a_1}$ | −4.2610 | 0.0000 | 0.0000 |
| $\boldsymbol{a_2}$ | 0.0000 | −5.6311 | 0.0000 |
| $\boldsymbol{a_3}$ | 0.0000 | 0.0000 | 25.0582 |

**Atomic positions:** Fractional coordinates, Hubbard $U$ (in eV) and magnetic moments (in $\mu_B$, computed from orbital occupations $m_o$ or integration spheres $m_i$) of each atom of monolayer HoSeI.

| atom | $x$ | $y$ | $z$ | $U$ | $m_o$ | $m_i$ |
|---|---|---|---|---|---|---|
| Ho | −0.25 | 0.25 | −0.05 | 6.23 | −3.98 | −4.00 |
| Ho | 0.25 | −0.25 | 0.05 | 6.23 | 3.98 | 4.01 |
| I | 0.25 | 0.25 | −0.13 | 0.0 | – | 0.01 |
| Se | −0.25 | −0.25 | −0.03 | 0.0 | – | 0.00 |
| Se | 0.25 | 0.25 | 0.03 | 0.0 | – | 0.00 |
| I | −0.25 | −0.25 | 0.13 | 0.0 | – | −0.01 |

## $HoTe_3$ (AFM)

Band gap: 0.0 eV
Total magnetization: 0.0 $\mu_B/cell$
Absolute magnetization: 8.23 $\mu_B/cell$
MC2D entry: https://mc2d.materialscloud.org/#/details/mc2d-1387

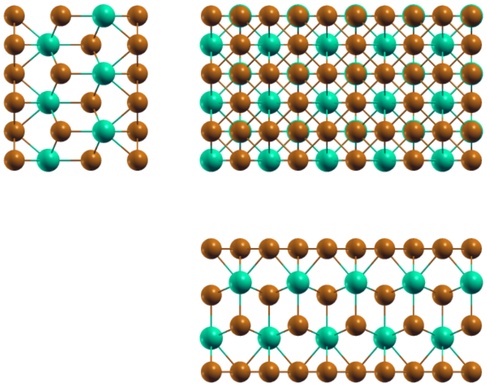

**Geometry:** Views of $HoTe_3$ as seen from the $x$ axis (left), the $y$ axis (bottom), and the $z$ axis (center).

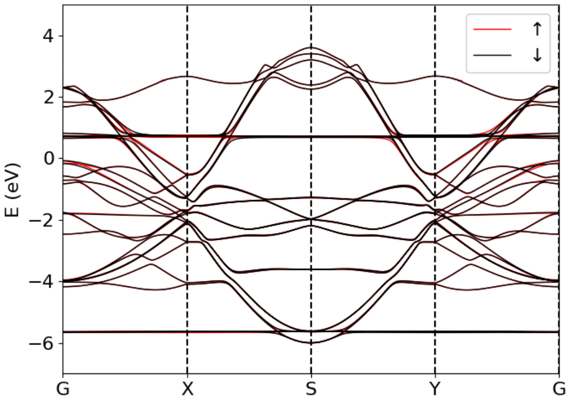


**Electronic bandstructure:** Spin-resolved energy bands of monolayer $HoTe_3$ along a high-symmetry path.

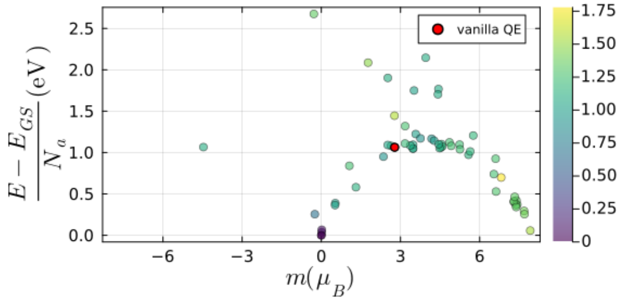


**Unique states:** Representation of 58 self-consistent unique states for monolayer $HoTe_3$ identified using RomeoDFT (see Section S6).

**Lattice vectors:** Cartesian components (in [Å]) of the lattice vectors for $HoTe_3$.

| | $x$ | $y$ | $z$ |
|---|---|---|---|
| $a_1$ | 4.3403 | 0.0000 | 0.0000 |
| $a_2$ | 0.0000 | 4.3310 | 0.0000 |
| $a_3$ | 0.0000 | 0.0000 | 28.9658 |

**Atomic positions:** Fractional coordinates, Hubbard $U$ (in eV) and magnetic moments (in $\mu_B$, computed from orbital occupations $m_o$ or integration spheres $m_i$) of each atom of monolayer $HoTe_3$.

| atom | $x$ | $y$ | $z$ | $U$ | $m_o$ | $m_i$ |
|---|---|---|---|---|---|---|
| Ho | 0.50 | 0.75 | 0.43 | 5.05 | 3.97 | 4.02 |
| Ho | 0.0 | 0.25 | 0.57 | 5.05 | −3.97 | −4.02 |
| Te | 0.50 | 0.75 | 0.54 | 0.0 | – | 0.01 |
| Te | 0.50 | 0.25 | 0.66 | 0.0 | – | 0.01 |
| Te | 0.50 | 0.25 | 0.34 | 0.0 | – | −0.01 |
| Te | 0.0 | 0.25 | 0.46 | 0.0 | – | −0.01 |
| Te | 0.0 | 0.75 | 0.34 | 0.0 | – | −0.01 |
| Te | 0.0 | 0.75 | 0.66 | 0.0 | – | 0.01 |

## $IrN_4Cl_6$ (AFM)

Band gap: 0.27 eV
Total magnetization: 0.0 $\mu_B/cell$
Absolute magnetization: 2.13 $\mu_B/cell$
MC2D entry: https://mc2d.materialscloud.org/#/details/mc2d-2173

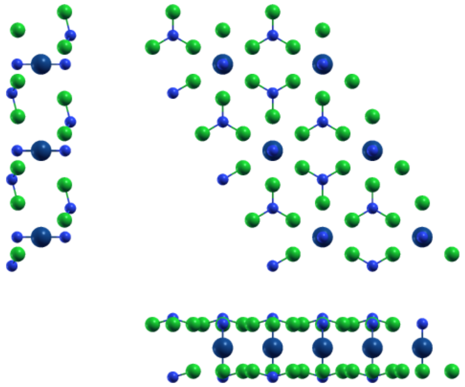

**Geometry:** Views of $IrN_4Cl_6$ as seen from the $x$ axis (left), the $y$ axis (bottom), and the $z$ axis (center).

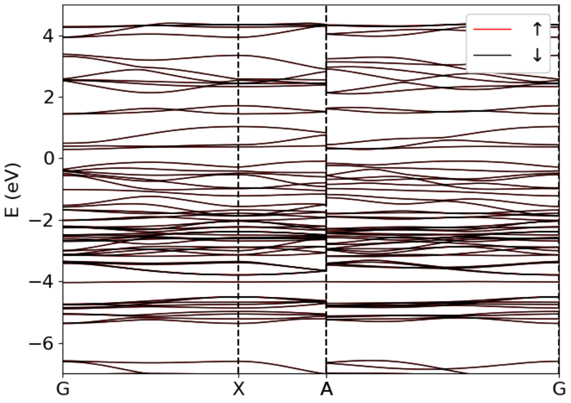


**Electronic bandstructure:** Spin-resolved energy bands of monolayer $IrN_4Cl_6$ along a high-symmetry path.

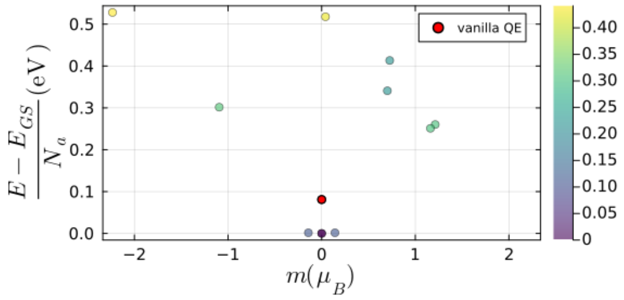


**Unique states:** Representation of 13 self-consistent unique states for monolayer $IrN_4Cl_6$ identified using RomeoDFT (see Section S6).

**Lattice vectors:** Cartesian components (in [Å]) of the lattice vectors for $IrN_4Cl_6$.

| | $x$ | $y$ | $z$ |
|---|---|---|---|
| $a_1$ | −7.3093 | −12.6601 | 0.0000 |
| $a_2$ | 7.3093 | 0.0000 | 0.0000 |
| $a_3$ | 0.0000 | 0.0000 | 19.3002 |

**Atomic positions:** Fractional coordinates, Hubbard $U$ (in eV) and magnetic moments (in $\mu_B$, computed from orbital occupations $m_o$ or integration spheres $m_i$) of each atom of monolayer $IrN_4Cl_6$.

| atom | $x$ | $y$ | $z$ | $U$ | $m_o$ | $m_i$ |
|---|---|---|---|---|---|---|
| Ir | 0.33 | 0.33 | 0.00 | 5.56 | −0.07 | −0.03 |
| Ir | 0.83 | 0.33 | 0.00 | 5.56 | 0.07 | 0.03 |
| Cl | 0.43 | 0.73 | 0.09 | 0.0 | – | 0.01 |
| Cl | 0.14 | 0.14 | 0.09 | 0.0 | – | 0.01 |
| Cl | 0.43 | 0.14 | 0.09 | 0.0 | – | 0.01 |
| N | 0.33 | 0.33 | 0.09 | 0.0 | – | 0.31 |
| N | 0.17 | 0.67 | −0.11 | 0.0 | – | −0.01 |
| N | 0.33 | 0.33 | −0.09 | 0.0 | – | 0.31 |
| Cl | 0.23 | 0.94 | −0.09 | 0.0 | – | 0.01 |
| Cl | 0.03 | 0.53 | −0.09 | 0.0 | – | −0.01 |
| Cl | 0.23 | 0.53 | −0.09 | 0.0 | – | 0.01 |
| N | 0.0 | 0.0 | 0.11 | 0.0 | – | 0.01 |
| Cl | 0.93 | 0.73 | 0.09 | 0.0 | – | 0.00 |
| Cl | 0.64 | 0.14 | 0.09 | 0.0 | – | −0.01 |
| Cl | 0.93 | 0.14 | 0.09 | 0.0 | – | 0.00 |
| N | 0.83 | 0.33 | 0.09 | 0.0 | – | −0.31 |
| N | 0.67 | 0.67 | −0.11 | 0.0 | – | 0.01 |
| N | 0.83 | 0.33 | −0.09 | 0.0 | – | −0.31 |
| Cl | 0.73 | 0.94 | −0.09 | 0.0 | – | 0.00 |
| Cl | 0.53 | 0.53 | −0.09 | 0.0 | – | 0.01 |
| Cl | 0.73 | 0.53 | −0.09 | 0.0 | – | 0.00 |
| N | 0.50 | 0.0 | 0.11 | 0.0 | – | −0.01 |

## $K_4Fe$ (FM)

Band gap: 0.0 eV
Total magnetization: 6.64 $\mu_B/cell$
Absolute magnetization: 6.72 $\mu_B/cell$
MC2D entry: https://mc2d.materialscloud.org/#/details/mc2d-2045

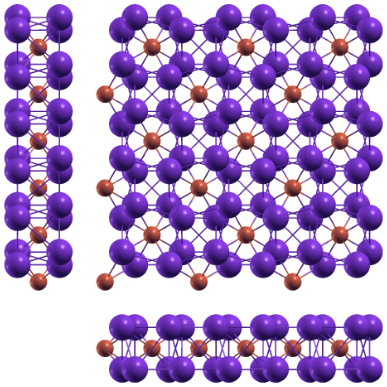

**Geometry:** Views of $K_4Fe$ as seen from the $x$ axis (left), the $y$ axis (bottom), and the $z$ axis (center).

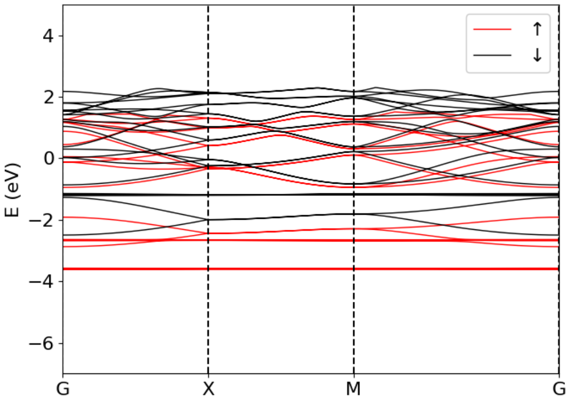


**Electronic bandstructure:** Spin-resolved energy bands of monolayer $K_4Fe$ along a high-symmetry path.

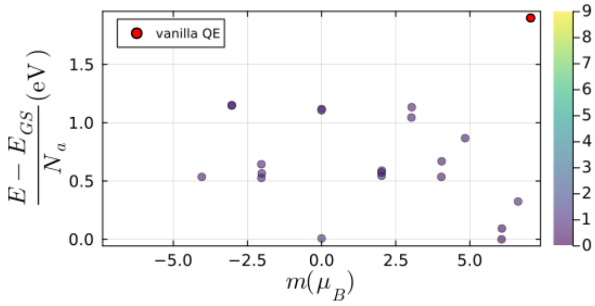


**Unique states:** Representation of 22 self-consistent unique states for monolayer $K_4Fe$ identified using RomeoDFT (see Section S6).

**Lattice vectors:** Cartesian components (in [Å]) of the lattice vectors for $K_4Fe$.

| | $x$ | $y$ | $z$ |
|---|---|---|---|
| $a_1$ | 8.4648 | 0.0000 | 0.0000 |
| $a_2$ | 0.0000 | 8.4648 | 0.0000 |
| $a_3$ | 0.0000 | 0.0000 | 19.4283 |

**Atomic positions:** Fractional coordinates, Hubbard $U$ (in eV) and magnetic moments (in $\mu_B$, computed from orbital occupations $m_o$ or integration spheres $m_i$) of each atom of monolayer $K_4Fe$.

| atom | $x$ | $y$ | $z$ | $U$ | $m_o$ | $m_i$ |
|---|---|---|---|---|---|---|
| Fe | 0.0 | 0.0 | 0.00 | 3.50 | 3.04 | 3.10 |
| Fe | 0.50 | −0.50 | 0.00 | 3.50 | 3.03 | 3.09 |
| K | 0.68 | −0.82 | 0.10 | 0.0 | – | 0.01 |
| K | 0.32 | −0.18 | 0.10 | 0.0 | – | 0.01 |
| K | 0.32 | −0.82 | −0.10 | 0.0 | – | 0.01 |
| K | 0.68 | −0.18 | −0.10 | 0.0 | – | 0.01 |
| K | 0.18 | −0.68 | 0.10 | 0.0 | – | 0.01 |
| K | 0.82 | −0.32 | 0.10 | 0.0 | – | 0.01 |
| K | 0.18 | −0.32 | −0.10 | 0.0 | – | 0.01 |
| K | 0.82 | −0.68 | −0.10 | 0.0 | – | 0.01 |

## $KFeSe_2$ (AFM)

Band gap: 0.99 eV
Total magnetization: −0.0 $\mu_B/cell$
Absolute magnetization: 7.37 $\mu_B/cell$
MC2D entry: https://mc2d.materialscloud.org/#/details/mc2d-764

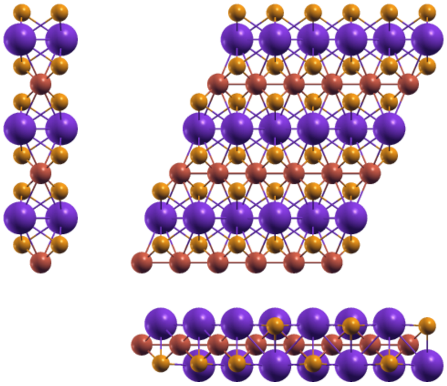

**Geometry:** Views of $KFeSe_2$ as seen from the $x$ axis (left), the $y$ axis (bottom), and the $z$ axis (center).

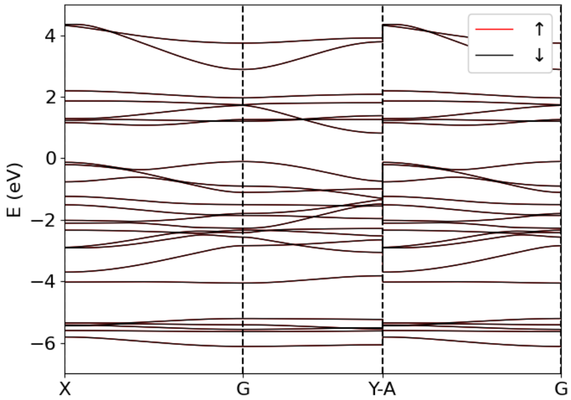


**Electronic bandstructure:** Spin-resolved energy bands of monolayer $KFeSe_2$ along a high-symmetry path.

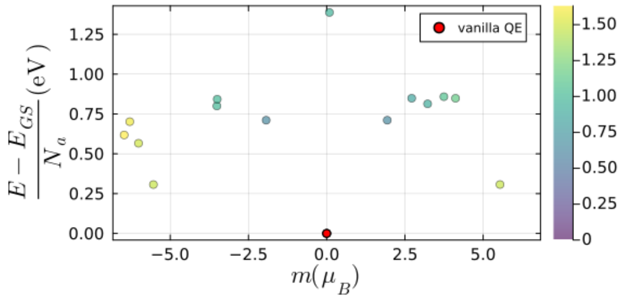


**Unique states:** Representation of 17 self-consistent unique states for monolayer $KFeSe_2$ identified using RomeoDFT (see Section S6).

**Lattice vectors:** Cartesian components (in [Å]) of the lattice vectors for $KFeSe_2$.

| | $x$ | $y$ | $z$ |
|---|---|---|---|
| $\boldsymbol{a_1}$ | 5.5602 | 0.0296 | 0.0000 |
| $\boldsymbol{a_2}$ | 2.7483 | 6.5365 | 0.0000 |
| $\boldsymbol{a_3}$ | 0.0000 | 0.0000 | 23.2732 |

**Atomic positions:** Fractional coordinates, Hubbard $U$ (in eV) and magnetic moments (in $\mu_B$, computed from orbital occupations $m_o$ or integration spheres $m_i$) of each atom of monolayer $KFeSe_2$.

| atom | $x$ | $y$ | $z$ | $U$ | $m_o$ | $m_i$ |
|---|---|---|---|---|---|---|
| Fe | 0.75 | 1.00 | 0.50 | 4.01 | 3.06 | 3.24 |
| Fe | 0.25 | 0.0 | 0.50 | 4.01 | −3.06 | −3.24 |
| K | 0.75 | 0.50 | 0.57 | 0.0 | – | 0.0 |
| K | 0.25 | 0.50 | 0.43 | 0.0 | – | 0.0 |
| Se | 0.60 | 0.80 | 0.44 | 0.0 | – | 0.0 |
| Se | 0.10 | 0.80 | 0.56 | 0.0 | – | 0.0 |
| Se | 0.90 | 0.20 | 0.44 | 0.0 | – | 0.0 |
| Se | 0.40 | 0.20 | 0.56 | 0.0 | – | 0.0 |

# $KPtNCl_3$ (FM)

Band gap: 0.32 eV
Total magnetization: 1.99 $\mu_B/cell$
Absolute magnetization: 2.46 $\mu_B/cell$
MC2D entry: https://mc2d.materialscloud.org/#/details/mc2d-2259

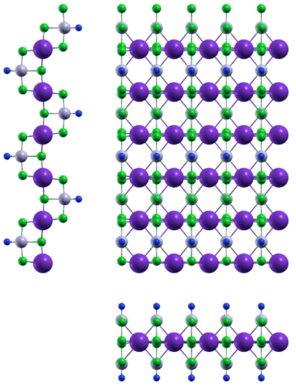

**Geometry:** Views of $KPtNCl_3$ as seen from the $x$ axis (left), the $y$ axis (bottom), and the $z$ axis (center).

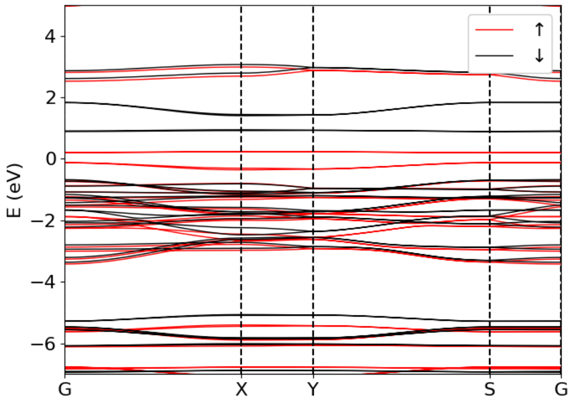


**Electronic bandstructure:** Spin-resolved energy bands of monolayer $KPtNCl_3$ along a high-symmetry path.

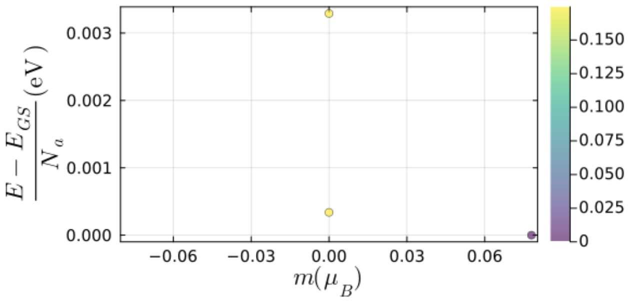


**Unique states:** Representation of 4 self-consistent unique states for monolayer $KPtNCl_3$ identified using RomeoDFT (see Section S6).

**Lattice vectors:** Cartesian components (in [Å]) of the lattice vectors for $KPtNCl_3$.

| | $x$ | $y$ | $z$ |
|---|---|---|---|
| $a_1$ | 0.0000 | −4.2229 | 0.0000 |
| $a_2$ | 10.3728 | 0.0000 | 0.0000 |
| $a_3$ | 0.0000 | 0.0000 | 29.9230 |

**Atomic positions:** Fractional coordinates, Hubbard $U$ (in eV) and magnetic moments (in $\mu_B$, computed from orbital occupations $m_o$ or integration spheres $m_i$) of each atom of monolayer $KPtNCl_3$.

| atom | $x$ | $y$ | $z$ | $U$ | $m_o$ | $m_i$ |
|---|---|---|---|---|---|---|
| Pt | 0.0 | −0.25 | −0.59 | 7.26 | 0.04 | 0.08 |
| Pt | 0.0 | 0.25 | −0.41 | 7.26 | 0.04 | 0.08 |
| N | 0.0 | −0.25 | −0.64 | 0.0 | – | 0.60 |
| Cl | 0.0 | −0.25 | −0.51 | 0.0 | – | 0.01 |
| Cl | 0.0 | −0.03 | −0.58 | 0.0 | – | −0.01 |
| Cl | 0.0 | −0.47 | −0.58 | 0.0 | – | −0.01 |
| K | −0.50 | 0.0 | −0.50 | 0.0 | – | 0.00 |
| K | −0.50 | 0.50 | −0.50 | 0.0 | – | 0.00 |
| N | 0.0 | 0.25 | −0.36 | 0.0 | – | 0.60 |
| Cl | 0.0 | 0.25 | −0.49 | 0.0 | – | 0.01 |
| Cl | 0.0 | 0.03 | −0.42 | 0.0 | – | −0.01 |
| Cl | 0.0 | 0.47 | −0.42 | 0.0 | – | −0.01 |

## LaBr (FM)

Band gap: 0.0 eV
Total magnetization: $-1.93\ \mu_B/cell$
Absolute magnetization: $1.96\ \mu_B/cell$
MC2D entry: https://mc2d.materialscloud.org/#/details/mc2d-55

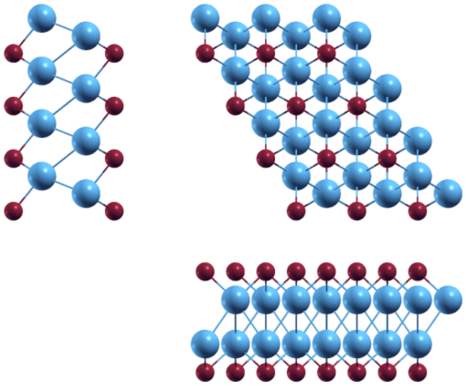

**Geometry:** Views of LaBr as seen from the $x$ axis (left), the $y$ axis (bottom), and the $z$ axis (center).

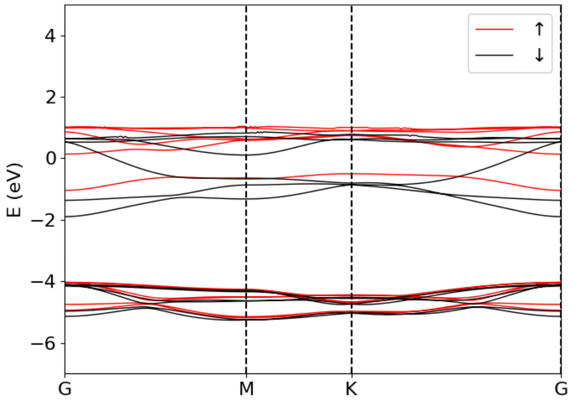


**Electronic bandstructure:** Spin-resolved energy bands of monolayer LaBr along a high-symmetry path.

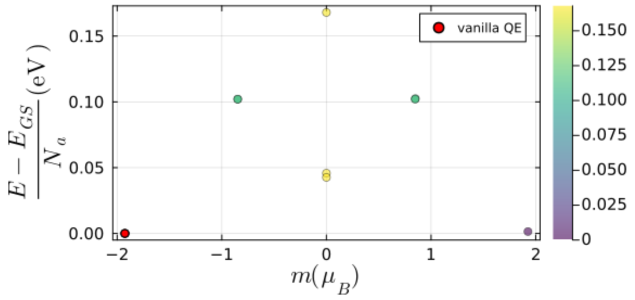


**Unique states:** Representation of 10 self-consistent unique states for monolayer LaBr identified using RomeoDFT (see Section S6).

**Lattice vectors:** Cartesian components (in [Å]) of the lattice vectors for LaBr.

| | $x$ | $y$ | $z$ |
|---|---|---|---|
| $\boldsymbol{a_1}$ | 4.1455 | 0.0000 | 0.0000 |
| $\boldsymbol{a_2}$ | −2.0727 | 3.5901 | 0.0000 |
| $\boldsymbol{a_3}$ | 0.0000 | 0.0000 | 27.0184 |

**Atomic positions:** Fractional coordinates, Hubbard $U$ (in eV) and magnetic moments (in $\mu_B$, computed from orbital occupations $m_o$ or integration spheres $m_i$) of each atom of monolayer LaBr.

| atom | $x$ | $y$ | $z$ | $U$ | $m_o$ | $m_i$ |
|---|---|---|---|---|---|---|
| La | 0.33 | 0.67 | 0.56 | 1.12 | −0.96 | −0.32 |
| La | 0.67 | 0.33 | 0.44 | 1.12 | −0.96 | −0.32 |
| Br | 0.0 | 0.0 | 0.63 | 0.0 | – | 0.00 |
| Br | 0.0 | 0.0 | 0.37 | 0.0 | – | 0.00 |

## $LaBr_2$ (FM)

Band gap: 0.98 eV
Total magnetization: $-2.01\ \mu_B/cell$
Absolute magnetization: $2.37\ \mu_B/cell$
MC2D entry: https://mc2d.materialscloud.org/#/details/mc2d-34

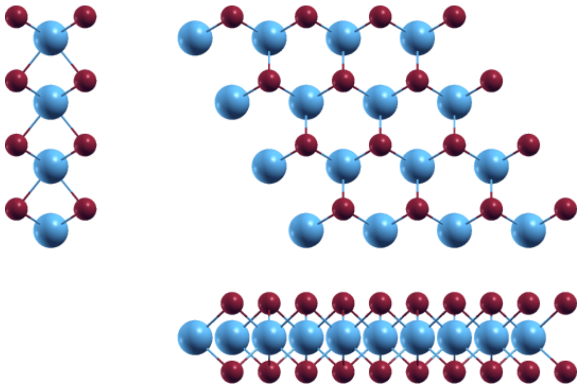

**Geometry:** Views of $LaBr_2$ as seen from the $x$ axis (left), the $y$ axis (bottom), and the $z$ axis (center).

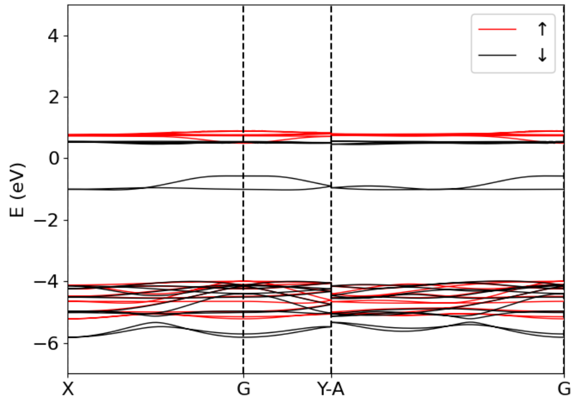


**Electronic bandstructure:** Spin-resolved energy bands of monolayer $LaBr_2$ along a high-symmetry path.

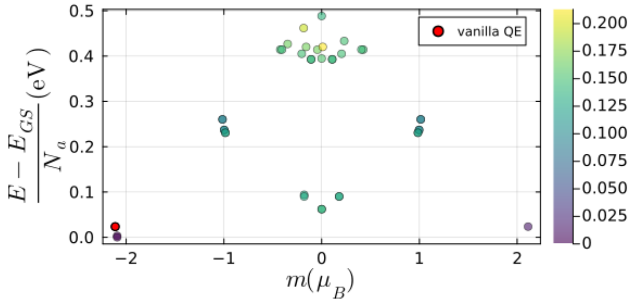


**Unique states:** Representation of 39 self-consistent unique states for monolayer $LaBr_2$ identified using RomeoDFT (see Section S6).

**Lattice vectors:** Cartesian components (in [Å]) of the lattice vectors for $LaBr_2$.

| | $x$ | $y$ | $z$ |
|---|---|---|---|
| $\boldsymbol{a_1}$ | 8.2903 | 0.0000 | 0.0000 |
| $\boldsymbol{a_2}$ | −2.0726 | 3.5898 | 0.0000 |
| $\boldsymbol{a_3}$ | 0.0000 | 0.0000 | 23.8357 |

**Atomic positions:** Fractional coordinates, Hubbard $U$ (in eV) and magnetic moments (in $\mu_B$, computed from orbital occupations $m_o$ or integration spheres $m_i$) of each atom of monolayer $LaBr_2$.

| atom | $x$ | $y$ | $z$ | $U$ | $m_o$ | $m_i$ |
|---|---|---|---|---|---|---|
| La | 0.0 | 0.0 | 0.50 | 4.38 | −1.05 | −0.40 |
| La | 0.50 | 0.0 | 0.50 | 4.38 | −1.05 | −0.40 |
| Br | 0.33 | 0.33 | 0.58 | 0.0 | – | 0.03 |
| Br | 0.33 | 0.33 | 0.42 | 0.0 | – | 0.03 |
| Br | 0.83 | 0.33 | 0.58 | 0.0 | – | 0.03 |
| Br | 0.83 | 0.33 | 0.42 | 0.0 | – | 0.03 |

## LaCl (FM)

Band gap: 0.0 eV
Total magnetization: $-1.87\ \mu_B/cell$
Absolute magnetization: $1.88\ \mu_B/cell$
MC2D entry: https://mc2d.materialscloud.org/#/details/mc2d-113

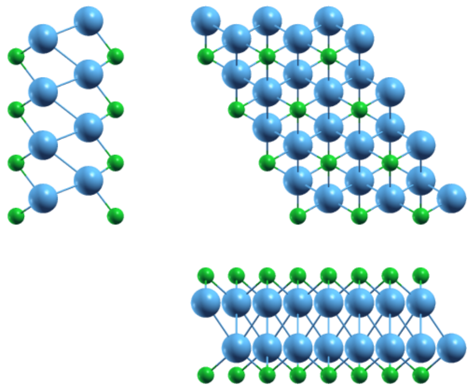

**Geometry:** Views of LaCl as seen from the $x$ axis (left), the $y$ axis (bottom), and the $z$ axis (center).

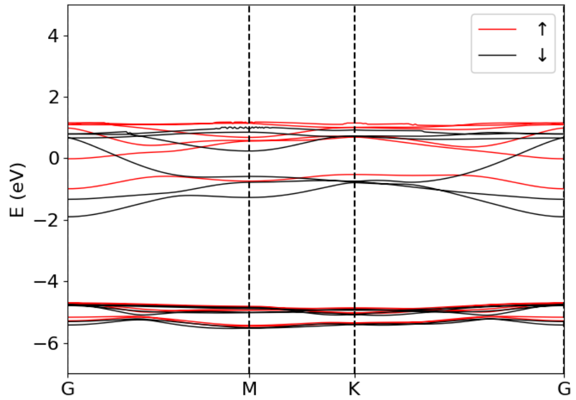


**Electronic bandstructure:** Spin-resolved energy bands of monolayer LaCl along a high-symmetry path.

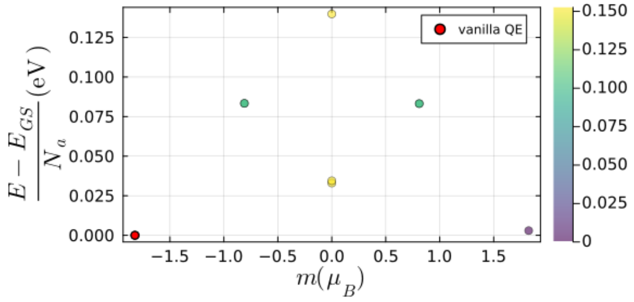


**Unique states:** Representation of 10 self-consistent unique states for monolayer LaCl identified using RomeoDFT (see Section S6).

**Lattice vectors:** Cartesian components (in [Å]) of the lattice vectors for LaCl.

| | $x$ | $y$ | $z$ |
|---|---|---|---|
| $\boldsymbol{a_1}$ | 2.0325 | −3.5205 | 0.0000 |
| $\boldsymbol{a_2}$ | 2.0325 | 3.5205 | 0.0000 |
| $\boldsymbol{a_3}$ | 0.0000 | 0.0000 | 25.1484 |

**Atomic positions:** Fractional coordinates, Hubbard $U$ (in eV) and magnetic moments (in $\mu_B$, computed from orbital occupations $m_o$ or integration spheres $m_i$) of each atom of monolayer LaCl.

| atom | $x$ | $y$ | $z$ | $U$ | $m_o$ | $m_i$ |
|---|---|---|---|---|---|---|
| La | 0.67 | 0.33 | 0.06 | 0.58 | −0.91 | −0.29 |
| La | 0.33 | 0.67 | −0.06 | 0.58 | −0.91 | −0.29 |
| Cl | 0.0 | 0.0 | −0.13 | 0.0 | – | −0.01 |
| Cl | 0.0 | 0.0 | 0.13 | 0.0 | – | −0.01 |

# LaI (FM)

Band gap: 0.0 eV
Total magnetization: $-1.88\ \mu_B/cell$
Absolute magnetization: 1.94 $\mu_B/cell$
MC2D entry: https://mc2d.materialscloud.org/#/details/mc2d-2213

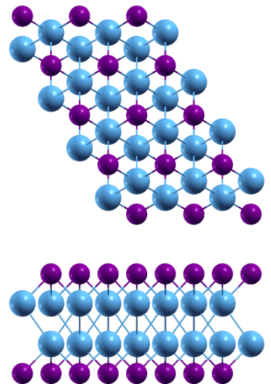

**Geometry:** Views of LaI as seen from the $x$ axis (left), the $y$ axis (bottom), and the $z$ axis (center).

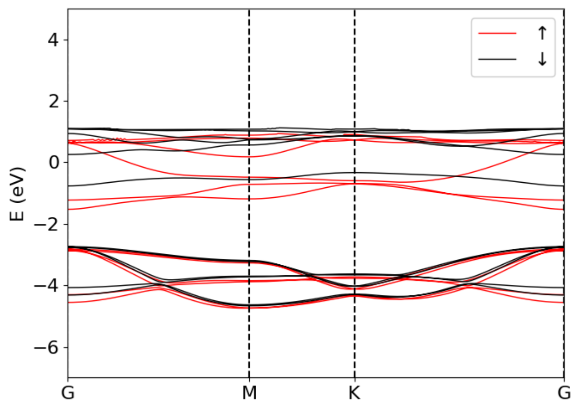


**Electronic bandstructure:** Spin-resolved energy bands of monolayer LaI along a high-symmetry path.

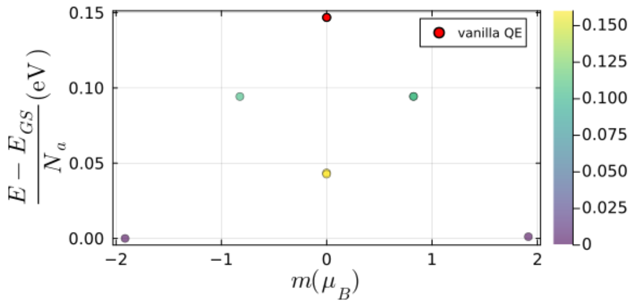


**Unique states:** Representation of 9 self-consistent unique states for monolayer LaI identified using RomeoDFT (see Section S6).

**Lattice vectors:** Cartesian components (in [Å]) of the lattice vectors for LaI.

| | $x$ | $y$ | $z$ |
|---|---|---|---|
| $\boldsymbol{a_1}$ | 2.1151 | −3.6635 | 0.0000 |
| $\boldsymbol{a_2}$ | 2.1151 | 3.6635 | 0.0000 |
| $\boldsymbol{a_3}$ | 0.0000 | 0.0000 | 26.6702 |

**Atomic positions:** Fractional coordinates, Hubbard $U$ (in eV) and magnetic moments (in $\mu_B$, computed from orbital occupations $m_o$ or integration spheres $m_i$) of each atom of monolayer LaI.

| atom | $x$ | $y$ | $z$ | $U$ | $m_o$ | $m_i$ |
|---|---|---|---|---|---|---|
| La | 0.17 | 0.33 | −0.06 | 1.16 | −0.96 | −0.36 |
| La | 0.83 | 0.67 | 0.06 | 1.16 | −0.96 | −0.36 |
| I | 0.50 | 0.00 | −0.14 | 0.0 | – | 0.01 |
| I | 0.50 | 0.00 | 0.14 | 0.0 | – | 0.01 |

## $LaNb_2O_7$ (FM)

Band gap: 0.0 eV
Total magnetization: 2.39 $\mu_B/cell$
Absolute magnetization: 2.74 $\mu_B/cell$
MC2D entry: https://mc2d.materialscloud.org/#/details/mc2d-2353

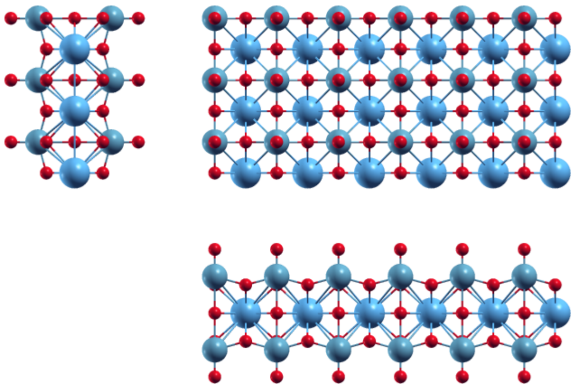

**Geometry:** Views of $LaNb_2O_7$ as seen from the $x$ axis (left), the $y$ axis (bottom), and the $z$ axis (center).

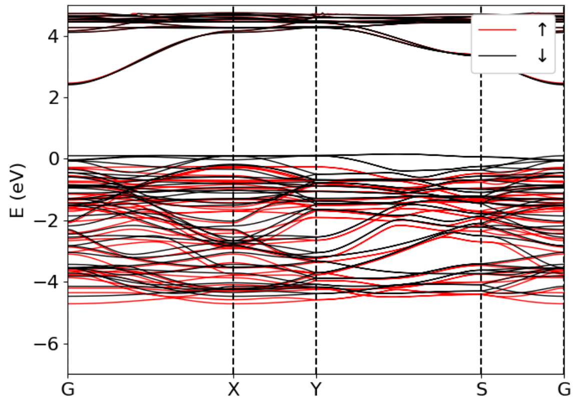


**Electronic bandstructure:** Spin-resolved energy bands of monolayer $LaNb_2O_7$ along a high-symmetry path.

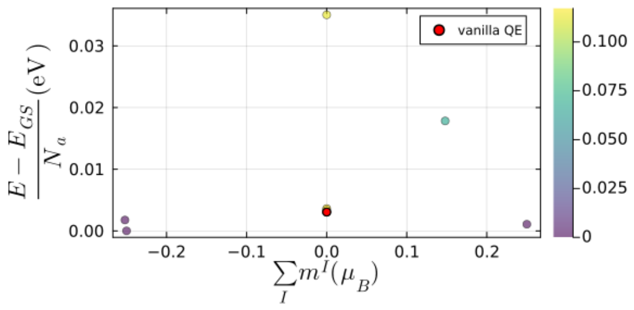


**Unique states:** Representation of 7 self-consistent unique states for monolayer $LaNb_2O_7$ identified using RomeoDFT (see Section S6).

**Lattice vectors:** Cartesian components (in [Å]) of the lattice vectors for $LaNb_2O_7$.

| | $x$ | $y$ | $z$ |
|---|---|---|---|
| $\boldsymbol{a_1}$ | 7.8641 | 0.0000 | 0.0000 |
| $\boldsymbol{a_2}$ | 0.0000 | 3.9321 | 0.0000 |
| $\boldsymbol{a_3}$ | 0.0000 | 0.0000 | 28.3480 |

**Atomic positions:** Fractional coordinates, Hubbard $U$ (in eV) and magnetic moments (in $\mu_B$, computed from orbital occupations $m_o$ or integration spheres $m_i$) of each atom of monolayer $LaNb_2O_7$.

| atom | $x$ | $y$ | $z$ | $U$ | $m_o$ | $m_i$ |
|---|---|---|---|---|---|---|
| Nb | 0.0 | −0.50 | 0.08 | 1.26 | −0.05 | 0.00 |
| La | 0.25 | −1.00 | 0.0 | 2.63 | −0.04 | −0.01 |
| Nb | 0.0 | −0.50 | −0.08 | 1.26 | −0.05 | 0.00 |
| Nb | 0.50 | −0.50 | 0.08 | 1.26 | −0.05 | 0.00 |
| La | 0.75 | −1.00 | 0.0 | 2.63 | −0.04 | −0.01 |
| Nb | 0.50 | −0.50 | −0.08 | 1.26 | −0.05 | 0.00 |
| O | 0.25 | −0.50 | 0.06 | 0.0 | – | 0.07 |
| O | 0.0 | −1.00 | 0.06 | 0.0 | – | 0.07 |
| O | 0.0 | −0.50 | 0.14 | 0.0 | – | 0.15 |
| O | 0.25 | −0.50 | −0.06 | 0.0 | – | 0.07 |
| O | 0.0 | −1.00 | −0.06 | 0.0 | – | 0.07 |
| O | 0.0 | −0.50 | −0.14 | 0.0 | – | 0.15 |
| O | 0.0 | −0.50 | 0.0 | 0.0 | – | 0.45 |
| O | 0.75 | −0.50 | 0.06 | 0.0 | – | 0.07 |
| O | 0.50 | −1.00 | 0.06 | 0.0 | – | 0.07 |
| O | 0.50 | −0.50 | 0.14 | 0.0 | – | 0.15 |
| O | 0.75 | −0.50 | −0.06 | 0.0 | – | 0.07 |
| O | 0.50 | −1.00 | −0.06 | 0.0 | – | 0.07 |
| O | 0.50 | −0.50 | −0.14 | 0.0 | – | 0.15 |
| O | 0.50 | −0.50 | 0.0 | 0.0 | – | 0.45 |

## $Li_5O_2Br_3$ (FM)

Band gap: 0.57 eV
Total magnetization: −4.58 $\mu_B/cell$
Absolute magnetization: 4.77 $\mu_B/cell$
MC2D entry: https://mc2d.materialscloud.org/#/details/mc2d-1802

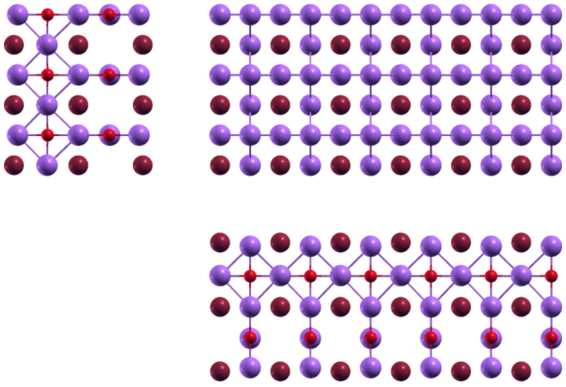

**Geometry:** Views of $Li_5O_2Br_3$ as seen from the $x$ axis (left), the $y$ axis (bottom), and the $z$ axis (center).

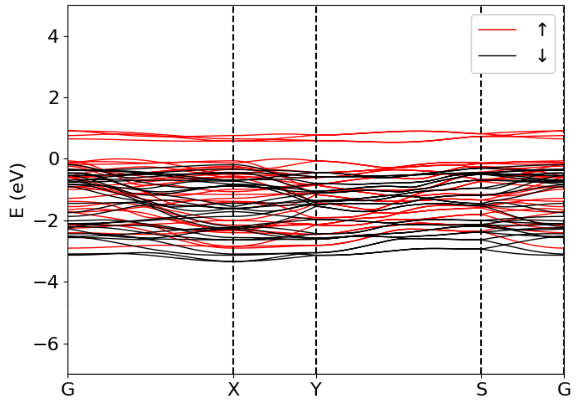


**Electronic bandstructure:** Spin-resolved energy bands of monolayer $Li_5O_2Br_3$ along a high-symmetry path.

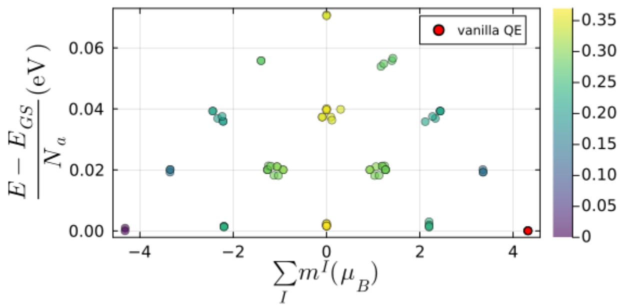


**Unique states:** Representation of 74 self-consistent unique states for monolayer $Li_5O_2Br_3$ identified using RomeoDFT (see Section S6).

**Lattice vectors:** Cartesian components (in [Å]) of the lattice vectors for $Li_5O_2Br_3$.

| | $x$ | $y$ | $z$ |
|---|---|---|---|
| $\boldsymbol{a_1}$ | 7.7442 | 0.0000 | 0.0000 |
| $\boldsymbol{a_2}$ | 0.0000 | 3.8721 | 0.0000 |
| $\boldsymbol{a_3}$ | 0.0000 | 0.0000 | 27.8989 |

**Atomic positions:** Fractional coordinates, Hubbard $U$ (in eV) and magnetic moments (in $\mu_B$, computed from orbital occupations $m_o$ or integration spheres $m_i$) of each atom of monolayer $Li_5O_2Br_3$.

| atom | $x$ | $y$ | $z$ | $U$ | $m_o$ | $m_i$ |
|---|---|---|---|---|---|---|
| Li | 0.25 | −1.00 | 0.07 | 0.00 | 0.00 | 0.00 |
| Li | 0.25 | −0.50 | 0.0 | 0.00 | 0.00 | 0.00 |
| Li | 0.25 | −0.50 | 0.14 | 0.00 | 0.00 | 0.00 |
| Br | 0.0 | −1.00 | 0.15 | 0.00 | −0.13 | −0.12 |
| Br | 0.0 | −1.00 | 0.0 | 0.00 | −0.13 | −0.14 |
| O | 0.25 | −0.50 | 0.07 | 0.00 | −0.88 | −0.74 |
| Li | 0.0 | −0.50 | −0.07 | 0.00 | 0.00 | 0.00 |
| Li | 0.25 | −0.50 | −0.14 | 0.00 | 0.00 | 0.00 |
| Br | 0.0 | −1.00 | −0.15 | 0.00 | −0.13 | −0.12 |
| O | 0.25 | −0.50 | −0.07 | 0.00 | −0.88 | −0.74 |
| Li | 0.75 | −1.00 | 0.07 | 0.00 | 0.00 | 0.00 |
| Li | 0.75 | −0.50 | 0.0 | 0.00 | 0.00 | 0.00 |
| Li | 0.75 | −0.50 | 0.14 | 0.00 | 0.00 | 0.00 |
| Br | 0.50 | −1.00 | 0.15 | 0.00 | −0.13 | −0.12 |
| Br | 0.50 | −1.00 | 0.0 | 0.00 | −0.13 | −0.14 |
| O | 0.75 | −0.50 | 0.07 | 0.00 | −0.88 | −0.74 |
| Li | 0.50 | −0.50 | −0.07 | 0.00 | 0.00 | 0.00 |
| Li | 0.75 | −0.50 | −0.14 | 0.00 | 0.00 | 0.00 |
| Br | 0.50 | −1.00 | −0.15 | 0.00 | −0.13 | −0.12 |
| O | 0.75 | −0.50 | −0.07 | 0.00 | −0.88 | −0.74 |

## LiFeAs (FM)

Band gap: 0.0 eV
Total magnetization: 0.32 $\mu_B/cell$
Absolute magnetization: 5.55 $\mu_B/cell$
MC2D entry: https://mc2d.materialscloud.org/#/details/mc2d-965

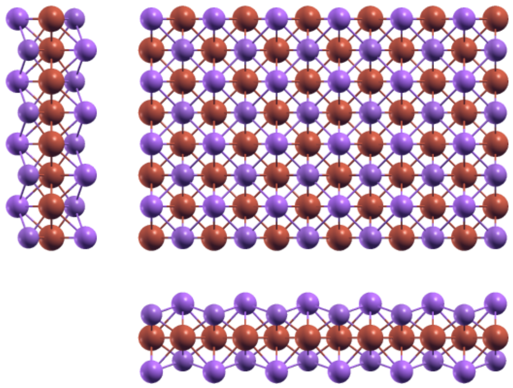

**Geometry:** Views of LiFeAs as seen from the $x$ axis (left), the $y$ axis (bottom), and the $z$ axis (center).

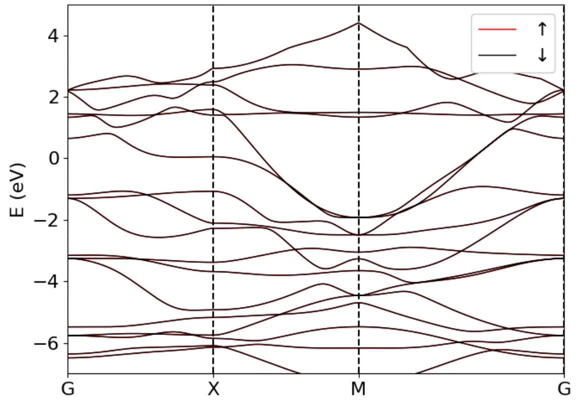


**Electronic bandstructure:** Spin-resolved energy bands of monolayer LiFeAs along a high-symmetry path.

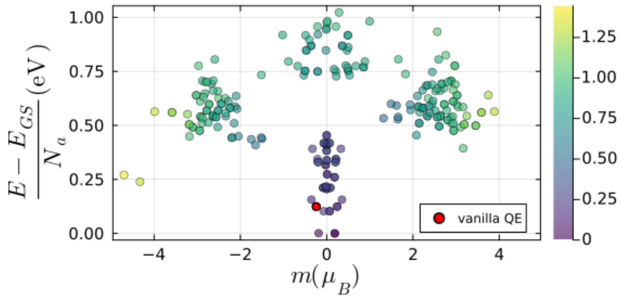


**Unique states:** Representation of 240 self-consistent unique states for monolayer LiFeAs identified using RomeoDFT (see Section S6).

**Lattice vectors:** Cartesian components (in [Å]) of the lattice vectors for LiFeAs.

| | $x$ | $y$ | $z$ |
|---|---|---|---|
| $\boldsymbol{a_1}$ | 3.7481 | 0.0000 | 0.0000 |
| $\boldsymbol{a_2}$ | 0.0000 | 3.7481 | 0.0000 |
| $\boldsymbol{a_3}$ | 0.0000 | 0.0000 | 24.0757 |

**Atomic positions:** Fractional coordinates, Hubbard $U$ (in eV) and magnetic moments (in $\mu_B$, computed from orbital occupations $m_o$ or integration spheres $m_i$) of each atom of monolayer LiFeAs.

| atom | $x$ | $y$ | $z$ | $U$ | $m_o$ | $m_i$ |
|---|---|---|---|---|---|---|
| Fe | 0.0 | 0.0 | 0.50 | 4.94 | −2.33 | −2.33 |
| Fe | 0.50 | 0.50 | 0.50 | 4.94 | 2.51 | 2.55 |
| Li | 0.0 | 0.50 | 0.58 | 0.0 | – | 0.00 |
| As | 0.50 | 0.0 | 0.56 | 0.0 | – | 0.03 |
| Li | 0.50 | 0.0 | 0.42 | 0.0 | – | 0.00 |
| As | 0.0 | 0.50 | 0.44 | 0.0 | – | 0.03 |

## $MnBi_2Te_4$ (FM)

Band gap: 0.87 eV
Total magnetization: 10.01 $\mu_B/cell$
Absolute magnetization: 10.16 $\mu_B/cell$
MC2D entry: https://mc2d.materialscloud.org/#/details/mc2d-776

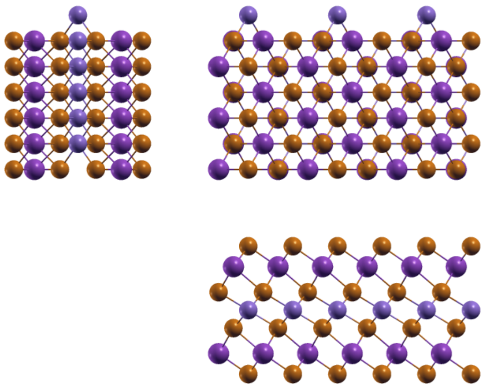

**Geometry:** Views of $MnBi_2Te_4$ as seen from the $x$ axis (left), the $y$ axis (bottom), and the $z$ axis (center).

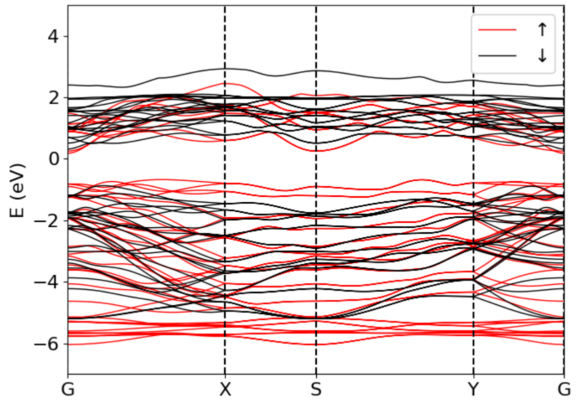


**Electronic bandstructure:** Spin-resolved energy bands of monolayer $MnBi_2Te_4$ along a high-symmetry path.

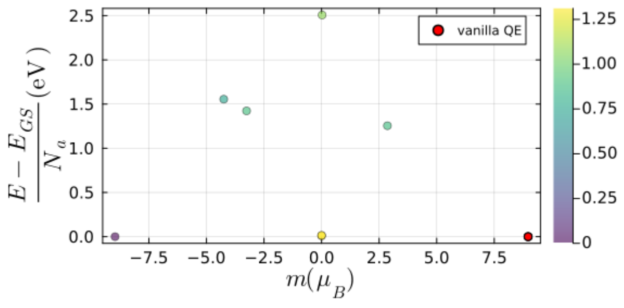


**Unique states:** Representation of 9 self-consistent unique states for monolayer $MnBi_2Te_4$ identified using RomeoDFT (see Section S6).

**Lattice vectors:** Cartesian components (in [Å]) of the lattice vectors for $MnBi_2Te_4$.

| | $x$ | $y$ | $z$ |
|---|---|---|---|
| $\boldsymbol{a_1}$ | 0.0000 | −7.5342 | 0.0000 |
| $\boldsymbol{a_2}$ | 4.3499 | 0.0000 | 0.0000 |
| $\boldsymbol{a_3}$ | 0.0000 | 0.0000 | 30.9061 |

**Atomic positions:** Fractional coordinates, Hubbard $U$ (in eV) and magnetic moments (in $\mu_B$, computed from orbital occupations $m_o$ or integration spheres $m_i$) of each atom of monolayer $MnBi_2Te_4$.

| atom | $x$ | $y$ | $z$ | $U$ | $m_o$ | $m_i$ |
|---|---|---|---|---|---|---|
| Mn | 0.33 | 1.00 | 0.50 | 2.95 | 4.48 | 4.52 |
| Mn | 0.83 | 0.50 | 0.50 | 2.95 | 4.48 | 4.52 |
| Bi | 0.67 | 1.00 | 0.38 | 0.0 | – | 0.03 |
| Bi | 1.17 | 0.50 | 0.38 | 0.0 | – | 0.03 |
| Bi | 1.00 | 1.00 | 0.62 | 0.0 | – | 0.03 |
| Bi | 0.50 | 0.50 | 0.62 | 0.0 | – | 0.03 |
| Te | 0.33 | 1.00 | 0.33 | 0.0 | – | 0.03 |
| Te | 0.83 | 0.50 | 0.33 | 0.0 | – | 0.03 |
| Te | 1.33 | 1.00 | 0.67 | 0.0 | – | 0.03 |
| Te | 0.83 | 0.50 | 0.67 | 0.0 | – | 0.03 |
| Te | 1.17 | 0.50 | 0.55 | 0.0 | – | 0.00 |
| Te | 0.67 | 1.00 | 0.55 | 0.0 | – | 0.00 |
| Te | 0.50 | 0.50 | 0.45 | 0.0 | – | 0.00 |
| Te | 1.00 | 1.00 | 0.45 | 0.0 | – | 0.00 |

## $MnBr_2$ (AFM)

Band gap: 3.32 eV
Total magnetization: 0.0 $\mu_B/cell$
Absolute magnetization: 9.76 $\mu_B/cell$
MC2D entry: https://mc2d.materialscloud.org/#/details/mc2d-37

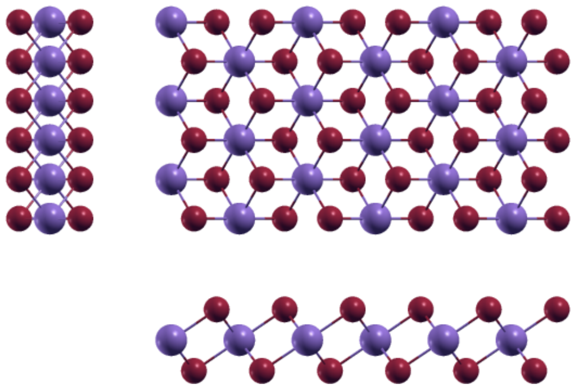

**Geometry:** Views of $MnBr_2$ as seen from the $x$ axis (left), the $y$ axis (bottom), and the $z$ axis (center).

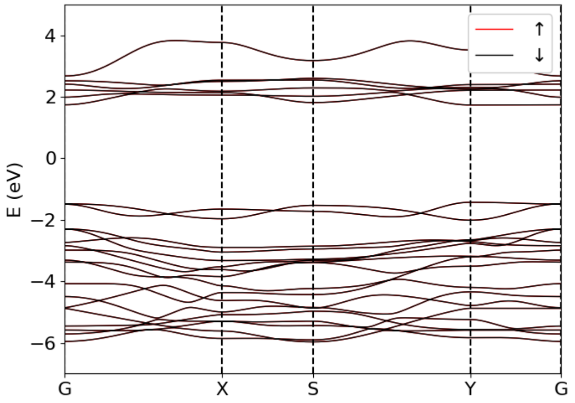


**Electronic bandstructure:** Spin-resolved energy bands of monolayer $MnBr_2$ along a high-symmetry path.

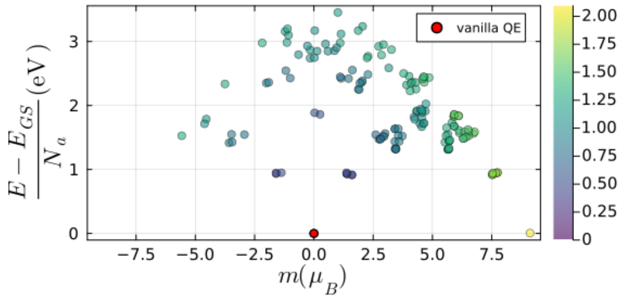


**Unique states:** Representation of 147 self-consistent unique states for monolayer $MnBr_2$ identified using RomeoDFT (see Section S6).

**Lattice vectors:** Cartesian components (in [Å]) of the lattice vectors for $MnBr_2$.

| | $x$ | $y$ | $z$ |
|---|---|---|---|
| $\boldsymbol{a_1}$ | −5.8191 | −3.3597 | 0.0000 |
| $\boldsymbol{a_2}$ | 1.9397 | −3.3597 | 0.0000 |
| $\boldsymbol{a_3}$ | 0.0000 | 0.0000 | 17.4869 |

**Atomic positions:** Fractional coordinates, Hubbard $U$ (in eV) and magnetic moments (in $\mu_B$, computed from orbital occupations $m_o$ or integration spheres $m_i$) of each atom of monolayer $MnBr_2$.

| atom | $x$ | $y$ | $z$ | $U$ | $m_o$ | $m_i$ |
|---|---|---|---|---|---|---|
| Mn | 0.50 | 1.00 | 0.0 | 2.66 | −4.55 | −4.53 |
| Mn | 1.00 | 0.50 | 0.0 | 2.66 | 4.55 | 4.53 |
| Br | 1.33 | 0.50 | 0.91 | 0.0 | – | −0.02 |
| Br | 0.83 | 1.00 | 0.91 | 0.0 | – | 0.02 |
| Br | 0.67 | 0.50 | 0.09 | 0.0 | – | −0.02 |
| Br | 0.17 | 1.00 | 0.09 | 0.0 | – | 0.02 |

## $MnCl_2$ (AFM)

Band gap: 3.49 eV
Total magnetization: $-0.0\ \mu_B/cell$
Absolute magnetization: $9.74\ \mu_B/cell$
MC2D entry: https://mc2d.materialscloud.org/#/details/mc2d-91

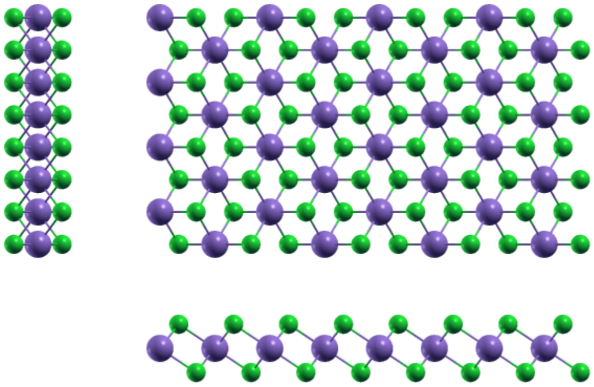

**Geometry:** Views of $MnCl_2$ as seen from the $x$ axis (left), the $y$ axis (bottom), and the $z$ axis (center).

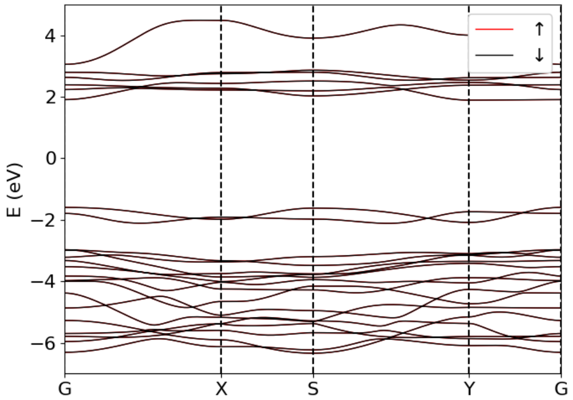


**Electronic bandstructure:** Spin-resolved energy bands of monolayer $MnCl_2$ along a high-symmetry path.

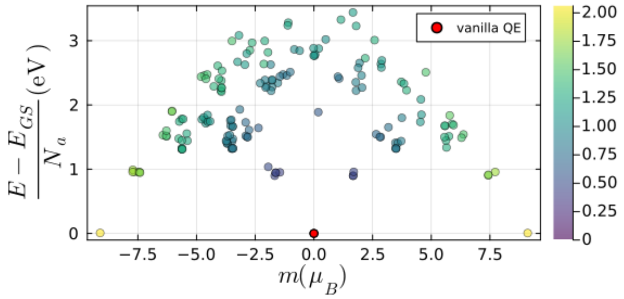


**Unique states:** Representation of 163 self-consistent unique states for monolayer $MnCl_2$ identified using RomeoDFT (see Section S6).

**Lattice vectors:** Cartesian components (in [Å]) of the lattice vectors for $MnCl_2$.

| | $x$ | $y$ | $z$ |
|---|---|---|---|
| $\boldsymbol{a_1}$ | 0.0000 | −6.3456 | 0.0000 |
| $\boldsymbol{a_2}$ | 3.7355 | 0.0000 | 0.0000 |
| $\boldsymbol{a_3}$ | 0.0000 | 0.0000 | 22.6312 |

**Atomic positions:** Fractional coordinates, Hubbard $U$ (in eV) and magnetic moments (in $\mu_B$, computed from orbital occupations $m_o$ or integration spheres $m_i$) of each atom of monolayer $MnCl_2$.

| atom | $x$ | $y$ | $z$ | $U$ | $m_o$ | $m_i$ |
|---|---|---|---|---|---|---|
| Mn | 1.17 | 0.50 | 0.50 | 2.62 | −4.55 | −4.44 |
| Mn | 0.67 | 0.0 | 0.50 | 2.62 | 4.55 | 4.44 |
| Cl | 0.84 | 0.50 | 0.44 | 0.0 | – | 0.02 |
| Cl | 0.34 | 0.0 | 0.44 | 0.0 | – | −0.02 |
| Cl | 1.00 | 0.0 | 0.56 | 0.0 | – | −0.02 |
| Cl | 0.50 | 0.50 | 0.56 | 0.0 | – | 0.02 |

## $MnGa_2S_4$ (AFM)

Band gap: 0.0 eV
Total magnetization: $-0.0\ \mu_B/cell$
Absolute magnetization: $9.32\ \mu_B/cell$
MC2D entry: https://mc2d.materialscloud.org/#/details/mc2d-1455

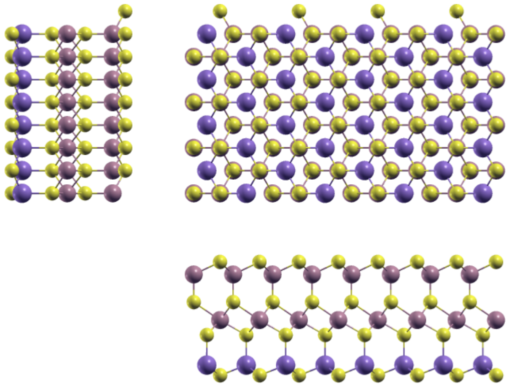

**Geometry:** Views of $MnGa_2S_4$ as seen from the $x$ axis (left), the $y$ axis (bottom), and the $z$ axis (center).

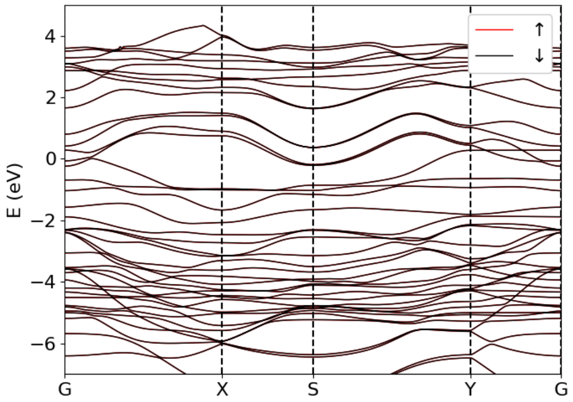


**Electronic bandstructure:** Spin-resolved energy bands of monolayer $MnGa_2S_4$ along a high-symmetry path.

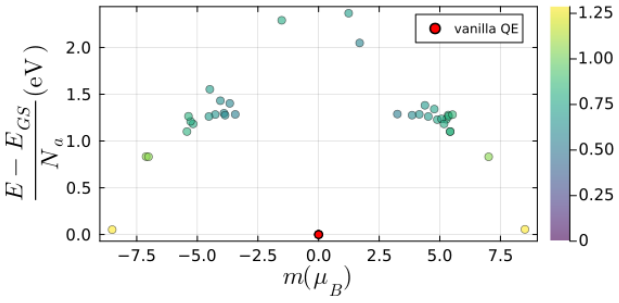


**Unique states:** Representation of 38 self-consistent unique states for monolayer $MnGa_2S_4$ identified using RomeoDFT (see Section S6).

**Lattice vectors:** Cartesian components (in [Å]) of the lattice vectors for $MnGa_2S_4$.

| | $x$ | $y$ | $z$ |
|---|---|---|---|
| $\boldsymbol{a_1}$ | 0.0000 | −6.3877 | 0.0000 |
| $\boldsymbol{a_2}$ | 3.6847 | 0.0000 | 0.0000 |
| $\boldsymbol{a_3}$ | 0.0000 | 0.0000 | 28.8953 |

**Atomic positions:** Fractional coordinates, Hubbard $U$ (in eV) and magnetic moments (in $\mu_B$, computed from orbital occupations $m_o$ or integration spheres $m_i$) of each atom of monolayer $MnGa_2S_4$.

| atom | $x$ | $y$ | $z$ | $U$ | $m_o$ | $m_i$ |
|---|---|---|---|---|---|---|
| Mn | 1.17 | 0.50 | 0.63 | 3.37 | −4.15 | −3.90 |
| Mn | 0.67 | 1.00 | 0.63 | 3.37 | 4.15 | 3.90 |
| Ga | 1.00 | 1.00 | 0.38 | 0.0 | – | 0.00 |
| Ga | 0.50 | 0.50 | 0.38 | 0.0 | – | 0.00 |
| Ga | 0.33 | 1.00 | 0.51 | 0.0 | – | 0.00 |
| Ga | 0.83 | 0.50 | 0.51 | 0.0 | – | 0.00 |
| S | 1.17 | 0.50 | 0.55 | 0.0 | – | 0.01 |
| S | 0.67 | 1.00 | 0.55 | 0.0 | – | −0.01 |
| S | 1.00 | 1.00 | 0.46 | 0.0 | – | −0.01 |
| S | 0.50 | 0.50 | 0.46 | 0.0 | – | 0.01 |
| S | 0.33 | 1.00 | 0.66 | 0.0 | – | 0.05 |
| S | 0.83 | 0.50 | 0.66 | 0.0 | – | −0.05 |
| S | 0.83 | 0.50 | 0.35 | 0.0 | – | 0.00 |
| S | 0.33 | 1.00 | 0.35 | 0.0 | – | 0.00 |

# $MnI_2$ (AFM)

Band gap: 2.59 eV
Total magnetization: 0.0 $\mu_B/cell$
Absolute magnetization: 9.76 $\mu_B/cell$
MC2D entry: https://mc2d.materialscloud.org/#/details/mc2d-2719

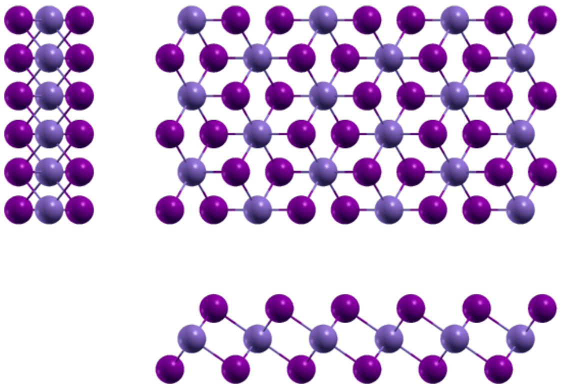

**Geometry:** Views of $MnI_2$ as seen from the $x$ axis (left), the $y$ axis (bottom), and the $z$ axis (center).

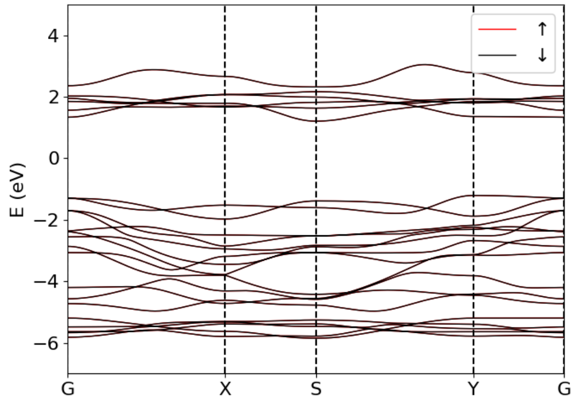


**Electronic bandstructure:** Spin-resolved energy bands of monolayer $MnI_2$ along a high-symmetry path.

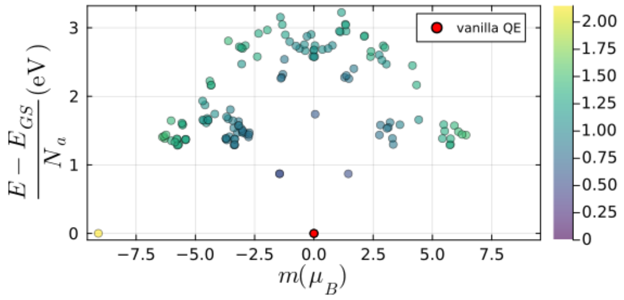


**Unique states:** Representation of 136 self-consistent unique states for monolayer $MnI_2$ identified using RomeoDFT (see Section S6).

**Lattice vectors:** Cartesian components (in [Å]) of the lattice vectors for $MnI_2$.

| | $x$ | $y$ | $z$ |
|---|---|---|---|
| $\boldsymbol{a_1}$ | −6.2173 | −3.5895 | 0.0000 |
| $\boldsymbol{a_2}$ | 2.0724 | −3.5895 | 0.0000 |
| $\boldsymbol{a_3}$ | 0.0000 | 0.0000 | 17.8728 |

**Atomic positions:** Fractional coordinates, Hubbard $U$ (in eV) and magnetic moments (in $\mu_B$, computed from orbital occupations $m_o$ or integration spheres $m_i$) of each atom of monolayer $MnI_2$.

| atom | $x$ | $y$ | $z$ | $U$ | $m_o$ | $m_i$ |
|---|---|---|---|---|---|---|
| Mn | 0.75 | 0.25 | 0.0 | 2.74 | −4.54 | −4.58 |
| Mn | 0.25 | 0.75 | 0.0 | 2.74 | 4.54 | 4.58 |
| I | 1.42 | 1.25 | 0.91 | 0.0 | – | 0.02 |
| I | 0.92 | 0.75 | 0.91 | 0.0 | – | −0.02 |
| I | 0.08 | 0.25 | 0.09 | 0.0 | – | 0.02 |
| I | 0.58 | 0.75 | 0.09 | 0.0 | – | −0.02 |

## $MoCl_3$ (AFM)

Band gap: 2.59 eV
Total magnetization: $-0.0\ \mu_B/cell$
Absolute magnetization: $5.06\ \mu_B/cell$
MC2D entry: https://mc2d.materialscloud.org/#/details/mc2d-339

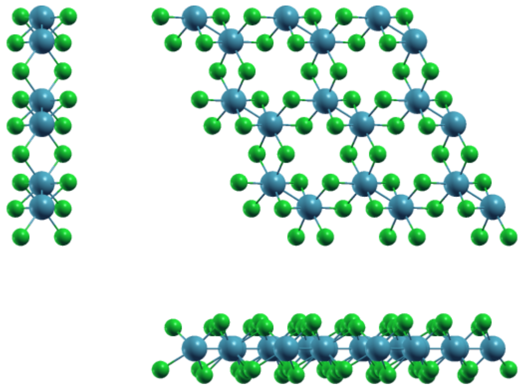

**Geometry:** Views of $MoCl_3$ as seen from the $x$ axis (left), the $y$ axis (bottom), and the $z$ axis (center).

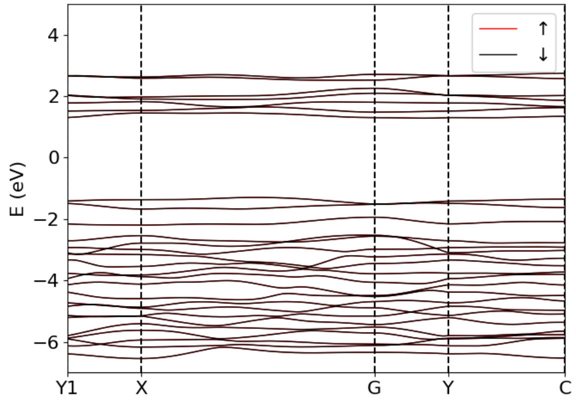


**Electronic bandstructure:** Spin-resolved energy bands of monolayer $MoCl_3$ along a high-symmetry path.

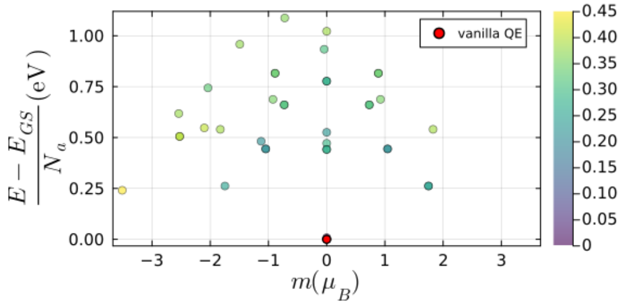


**Unique states:** Representation of 39 self-consistent unique states for monolayer $MoCl_3$ identified using RomeoDFT (see Section S6).

**Lattice vectors:** Cartesian components (in [Å]) of the lattice vectors for $MoCl_3$.

| | $x$ | $y$ | $z$ |
|---|---|---|---|
| $\boldsymbol{a_1}$ | 5.8047 | 0.0422 | 0.0000 |
| $\boldsymbol{a_2}$ | −2.5201 | 5.2293 | 0.0000 |
| $\boldsymbol{a_3}$ | 0.0000 | 0.0000 | 23.2910 |

**Atomic positions:** Fractional coordinates, Hubbard $U$ (in eV) and magnetic moments (in $\mu_B$, computed from orbital occupations $m_o$ or integration spheres $m_i$) of each atom of monolayer $MoCl_3$.

| atom | $x$ | $y$ | $z$ | $U$ | $m_o$ | $m_i$ |
|---|---|---|---|---|---|---|
| Mo | 0.84 | 0.56 | 0.50 | 2.74 | −2.12 | −1.67 |
| Mo | 0.56 | 0.84 | 0.50 | 2.74 | 2.12 | 1.67 |
| Cl | 0.54 | 0.54 | 0.57 | 0.0 | – | 0.0 |
| Cl | 0.86 | 0.86 | 0.43 | 0.0 | – | 0.0 |
| Cl | 0.86 | 0.20 | 0.56 | 0.0 | – | 0.02 |
| Cl | 0.20 | 0.86 | 0.56 | 0.0 | – | −0.02 |
| Cl | 0.20 | 0.54 | 0.44 | 0.0 | – | 0.02 |
| Cl | 0.54 | 0.20 | 0.44 | 0.0 | – | −0.02 |

## $MoS_2I_2$ (FM)

Band gap: 0.05 eV
Total magnetization: −2.0 $\mu_B/cell$
Absolute magnetization: 3.04 $\mu_B/cell$
MC2D entry: https://mc2d.materialscloud.org/#/details/mc2d-1839

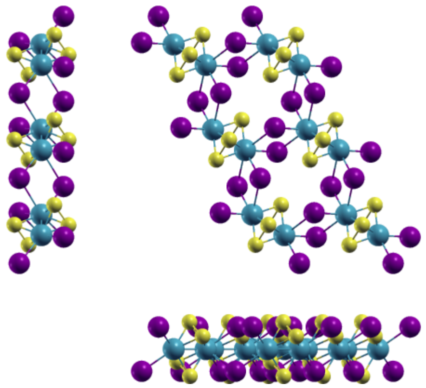

**Geometry:** Views of $MoS_2I_2$ as seen from the $x$ axis (left), the $y$ axis (bottom), and the $z$ axis (center).

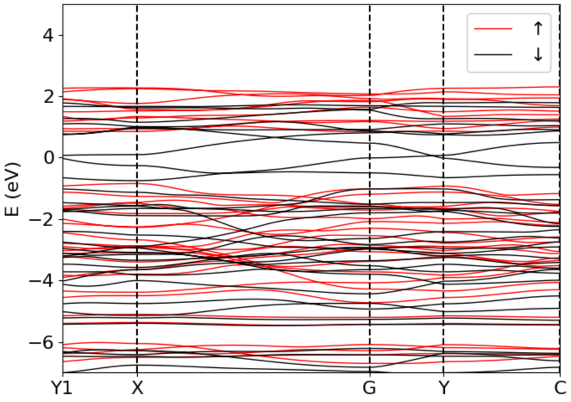


**Electronic bandstructure:** Spin-resolved energy bands of monolayer $MoS_2I_2$ along a high-symmetry path.

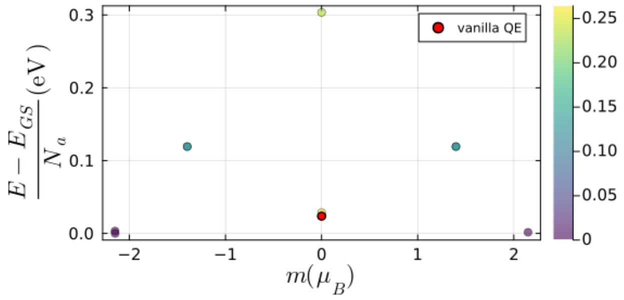


**Unique states:** Representation of 10 self-consistent unique states for monolayer $MoS_2I_2$ identified using RomeoDFT (see Section S6).

**Lattice vectors:** Cartesian components (in [Å]) of the lattice vectors for $MoS_2I_2$.

| | $x$ | $y$ | $z$ |
|---|---|---|---|
| $\boldsymbol{a_1}$ | 3.7113 | −5.8015 | 0.0000 |
| $\boldsymbol{a_2}$ | 3.7113 | 5.8015 | 0.0000 |
| $\boldsymbol{a_3}$ | 0.0000 | 0.0000 | 19.9256 |

**Atomic positions:** Fractional coordinates, Hubbard $U$ (in eV) and magnetic moments (in $\mu_B$, computed from orbital occupations $m_o$ or integration spheres $m_i$) of each atom of monolayer $MoS_2I_2$.

| atom | $x$ | $y$ | $z$ | $U$ | $m_o$ | $m_i$ |
|---|---|---|---|---|---|---|
| Mo | −0.38 | 0.38 | 0.0 | 3.39 | −1.07 | −0.87 |
| Mo | −0.62 | 0.62 | 0.0 | 3.39 | −1.07 | −0.87 |
| I | −0.36 | 0.05 | 0.08 | 0.0 | – | 0.03 |
| I | −0.95 | 0.64 | 0.08 | 0.0 | – | 0.03 |
| I | −1.05 | 0.36 | −0.08 | 0.0 | – | 0.03 |
| I | −0.64 | −0.05 | −0.08 | 0.0 | – | 0.03 |
| S | −0.73 | 0.27 | 0.05 | 0.0 | – | 0.04 |
| S | −0.50 | 0.50 | 0.10 | 0.0 | – | 0.04 |
| S | −0.27 | 0.73 | −0.05 | 0.0 | – | 0.04 |
| S | −0.50 | 0.50 | −0.10 | 0.0 | – | 0.04 |

## $NaFeS_2O_2$ (FM)

Band gap: 0.0 eV
Total magnetization: 2.53 $\mu_B/cell$
Absolute magnetization: 4.05 $\mu_B/cell$
MC2D entry: https://mc2d.materialscloud.org/#/details/mc2d-1774

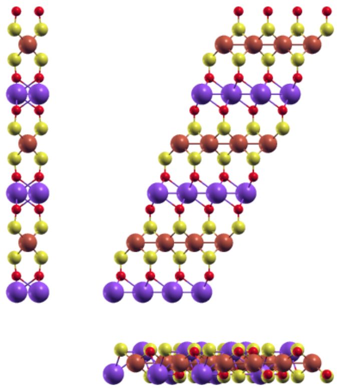

**Geometry:** Views of $NaFeS_2O_2$ as seen from the $x$ axis (left), the $y$ axis (bottom), and the $z$ axis (center).

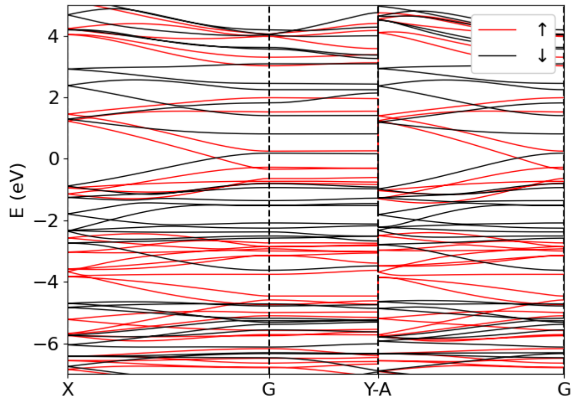


**Electronic bandstructure:** Spin-resolved energy bands of monolayer $NaFeS_2O_2$ along a high-symmetry path.

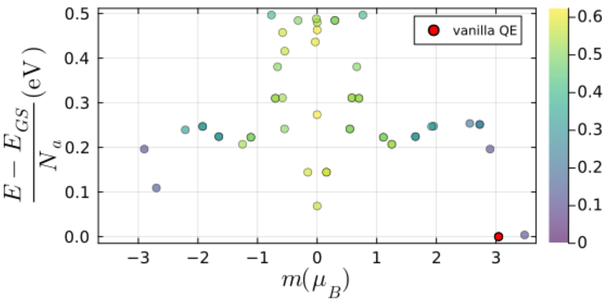


**Unique states:** Representation of 53 self-consistent unique states for monolayer $NaFeS_2O_2$ identified using RomeoDFT (see Section S6).

**Lattice vectors:** Cartesian components (in [Å]) of the lattice vectors for $NaFeS_2O_2$.

| | $x$ | $y$ | $z$ |
|---|---|---|---|
| $\boldsymbol{a_1}$ | 0.7256 | 4.9158 | 0.0000 |
| $\boldsymbol{a_2}$ | −7.7476 | 4.9191 | 0.0000 |
| $\boldsymbol{a_3}$ | 0.0000 | 0.0000 | 16.3735 |

**Atomic positions:** Fractional coordinates, Hubbard $U$ (in eV) and magnetic moments (in $\mu_B$, computed from orbital occupations $m_o$ or integration spheres $m_i$) of each atom of monolayer $NaFeS_2O_2$.

| atom | $x$ | $y$ | $z$ | $U$ | $m_o$ | $m_i$ |
|---|---|---|---|---|---|---|
| Fe | 0.25 | 0.50 | 0.00 | 5.31 | 1.52 | 1.46 |
| Fe | −0.25 | 0.50 | 0.00 | 5.31 | 1.52 | 1.46 |
| Na | 0.25 | 0.0 | 0.06 | 0.0 | – | 0.00 |
| O | 0.25 | 0.84 | −0.06 | 0.0 | – | −0.05 |
| O | 0.25 | 0.16 | −0.06 | 0.0 | – | −0.05 |
| S | −0.39 | 0.35 | 0.07 | 0.0 | – | −0.03 |
| S | 0.11 | 0.35 | −0.07 | 0.0 | – | −0.03 |
| S | −0.11 | 0.65 | 0.07 | 0.0 | – | −0.03 |
| S | 0.39 | 0.65 | −0.07 | 0.0 | – | −0.03 |
| Na | −0.25 | 1.00 | −0.06 | 0.0 | – | 0.00 |
| O | −0.25 | 0.84 | 0.06 | 0.0 | – | −0.05 |
| O | −0.25 | 0.16 | 0.06 | 0.0 | – | −0.05 |

# $NaN_3$ (FM)

Band gap: 1.09 eV
Total magnetization: $-4.0\ \mu_B/cell$
Absolute magnetization: $4.08\ \mu_B/cell$

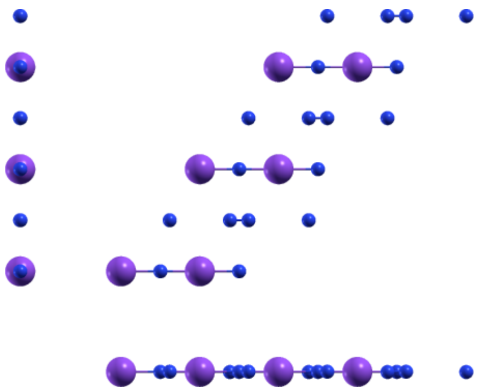

**Geometry:** Views of $NaN_3$ as seen from the $x$ axis (left), the $y$ axis (bottom), and the $z$ axis (center).

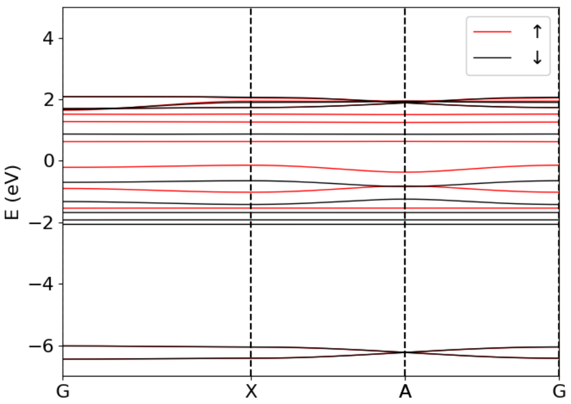


**Electronic bandstructure:** Spin-resolved energy bands of monolayer $NaN_3$ along a high-symmetry path.

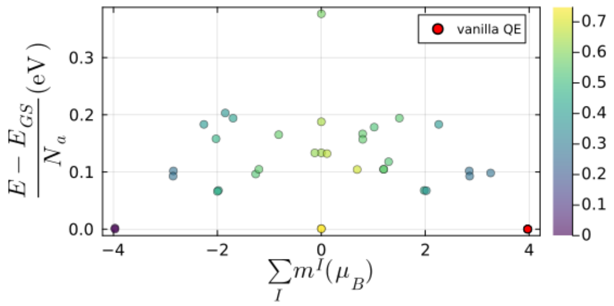


**Unique states:** Representation of 35 self-consistent unique states for monolayer $NaN_3$ identified using RomeoDFT (see Section S6).

**Lattice vectors:** Cartesian components (in [Å]) of the lattice vectors for $NaN_3$.

| | $x$ | $y$ | $z$ |
|---|---|---|---|
| $\boldsymbol{a_1}$ | 9.3867 | 0.0000 | 0.0000 |
| $\boldsymbol{a_2}$ | 4.6934 | 6.0944 | 0.0000 |
| $\boldsymbol{a_3}$ | 0.0000 | 0.0000 | 20.0006 |

**Atomic positions:** Fractional coordinates, Hubbard $U$ (in eV) and magnetic moments (in $\mu_B$, computed from orbital occupations $m_o$ or integration spheres $m_i$) of each atom of monolayer $NaN_3$.

| atom | $x$ | $y$ | $z$ | $U$ | $m_o$ | $m_i$ |
|---|---|---|---|---|---|---|
| Na | 0.50 | 0.0 | 0.50 | 0.00 | 0.00 | 0.00 |
| N | 0.25 | 0.0 | 0.50 | 0.00 | −1.99 | −0.58 |
| N | 0.06 | 0.50 | 0.50 | 0.00 | 0.00 | 0.00 |
| N | 0.44 | 0.50 | 0.50 | 0.00 | 0.00 | 0.00 |
| Na | 1.00 | 0.0 | 0.50 | 0.00 | 0.00 | 0.00 |
| N | 0.75 | 0.0 | 0.50 | 0.00 | −1.99 | −0.58 |
| N | 0.56 | 0.50 | 0.50 | 0.00 | 0.00 | 0.00 |
| N | 0.94 | 0.50 | 0.50 | 0.00 | 0.00 | 0.00 |

# $NbSe_2$ (FM)

Band gap: 0.48 eV
Total magnetization: 1.99 $\mu_B/cell$
Absolute magnetization: 2.54 $\mu_B/cell$
MC2D entry: https://mc2d.materialscloud.org/#/details/mc2d-212

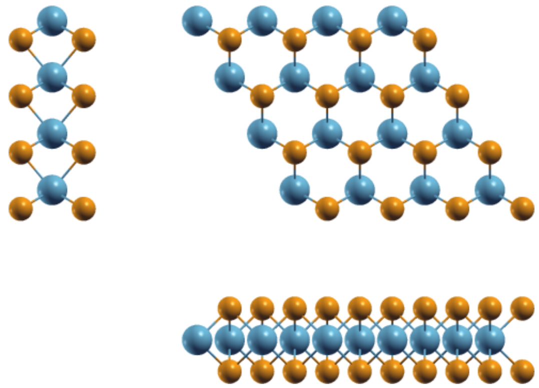

**Geometry:** Views of $NbSe_2$ as seen from the $x$ axis (left), the $y$ axis (bottom), and the $z$ axis (center).

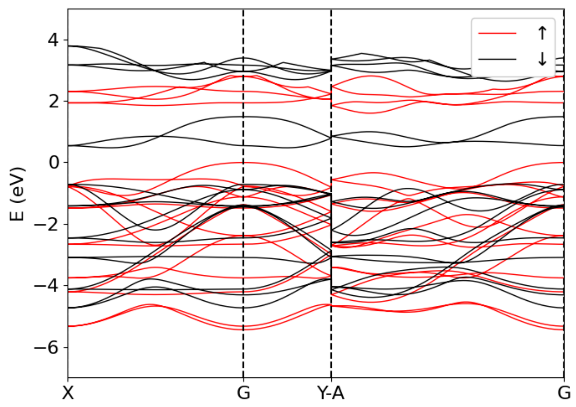


**Electronic bandstructure:** Spin-resolved energy bands of monolayer $NbSe_2$ along a high-symmetry path.

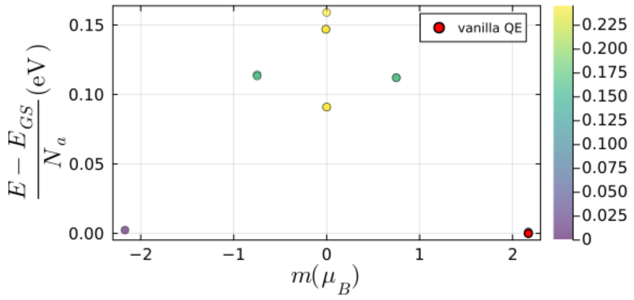


**Unique states:** Representation of 12 self-consistent unique states for monolayer $NbSe_2$ identified using RomeoDFT (see Section S6).

**Lattice vectors:** Cartesian components (in [Å]) of the lattice vectors for $NbSe_2$.

| | $x$ | $y$ | $z$ |
|---|---|---|---|
| $a_1$ | 6.9449 | 0.0000 | 0.0000 |
| $a_2$ | −1.7362 | 3.0073 | 0.0000 |
| $a_3$ | 0.0000 | 0.0000 | 23.3599 |

**Atomic positions:** Fractional coordinates, Hubbard $U$ (in eV) and magnetic moments (in $\mu_B$, computed from orbital occupations $m_o$ or integration spheres $m_i$) of each atom of monolayer $NbSe_2$.

| atom | $x$ | $y$ | $z$ | $U$ | $m_o$ | $m_i$ |
|---|---|---|---|---|---|---|
| Nb | 0.17 | 0.67 | 0.50 | 2.64 | 1.08 | 0.69 |
| Nb | 0.67 | 0.67 | 0.50 | 2.64 | 1.08 | 0.69 |
| Se | 0.33 | 0.33 | 0.57 | 0.0 | – | −0.05 |
| Se | 0.33 | 0.33 | 0.43 | 0.0 | – | −0.05 |
| Se | 0.83 | 0.33 | 0.57 | 0.0 | – | −0.05 |
| Se | 0.83 | 0.33 | 0.43 | 0.0 | – | −0.05 |

# $NdI_3$ (AFM)

Band gap: 0.82 eV
Total magnetization: 0.0 $\mu_B/cell$
Absolute magnetization: 6.4 $\mu_B/cell$
MC2D entry: https://mc2d.materialscloud.org/#/details/mc2d-2281

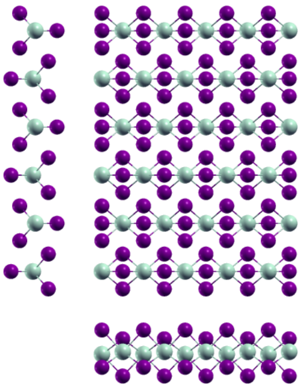

**Geometry:** Views of $NdI_3$ as seen from the $x$ axis (left), the $y$ axis (bottom), and the $z$ axis (center).

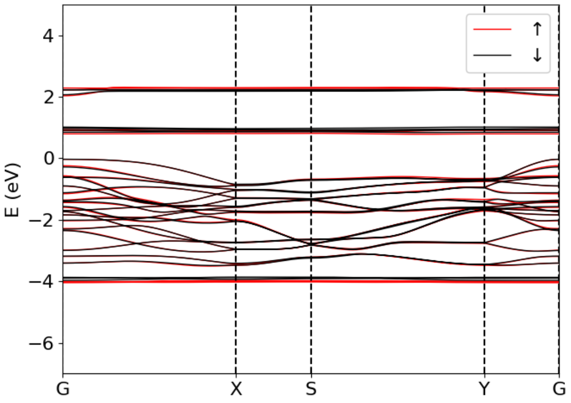


**Electronic bandstructure:** Spin-resolved energy bands of monolayer $NdI_3$ along a high-symmetry path.

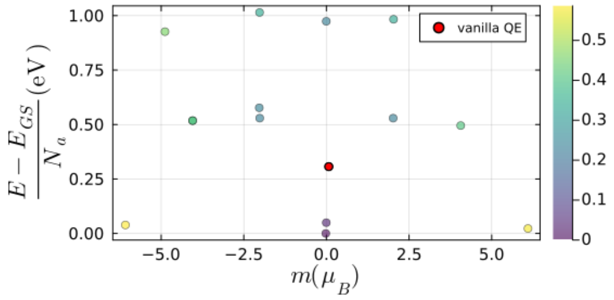


**Unique states:** Representation of 16 self-consistent unique states for monolayer $NdI_3$ identified using RomeoDFT (see Section S6).

**Lattice vectors:** Cartesian components (in [Å]) of the lattice vectors for $NdI_3$.

| | $x$ | $y$ | $z$ |
|---|---|---|---|
| $a_1$ | 4.3406 | 0.0000 | 0.0000 |
| $a_2$ | 0.0000 | 10.0627 | 0.0000 |
| $a_3$ | 0.0000 | 0.0000 | 21.4554 |

**Atomic positions:** Fractional coordinates, Hubbard $U$ (in eV) and magnetic moments (in $\mu_B$, computed from orbital occupations $m_o$ or integration spheres $m_i$) of each atom of monolayer $NdI_3$.

| atom | $x$ | $y$ | $z$ | $U$ | $m_o$ | $m_i$ |
|---|---|---|---|---|---|---|
| Nd | 0.25 | 0.25 | 0.01 | 3.79 | −3.05 | −3.05 |
| Nd | 0.75 | 0.75 | 0.99 | 3.84 | 3.03 | 3.04 |
| I | 0.25 | 0.93 | 0.07 | 0.0 | – | −0.01 |
| I | 0.25 | 0.57 | 0.07 | 0.0 | – | −0.01 |
| I | 0.75 | 0.43 | 0.93 | 0.0 | – | 0.02 |
| I | 0.75 | 0.07 | 0.93 | 0.0 | – | 0.02 |
| I | 0.25 | 0.75 | 0.89 | 0.0 | – | −0.02 |
| I | 0.75 | 0.25 | 0.11 | 0.0 | – | 0.02 |

## NdOBr (FM)

Band gap: 2.92 eV
Total magnetization: $-6.0\ \mu_B/cell$
Absolute magnetization: 6.16 $\mu_B/cell$
MC2D entry: https://mc2d.materialscloud.org/#/details/mc2d-62

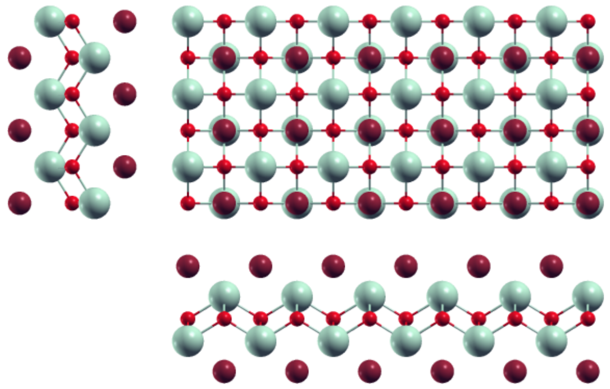

**Geometry:** Views of NdOBr as seen from the $x$ axis (left), the $y$ axis (bottom), and the $z$ axis (center).

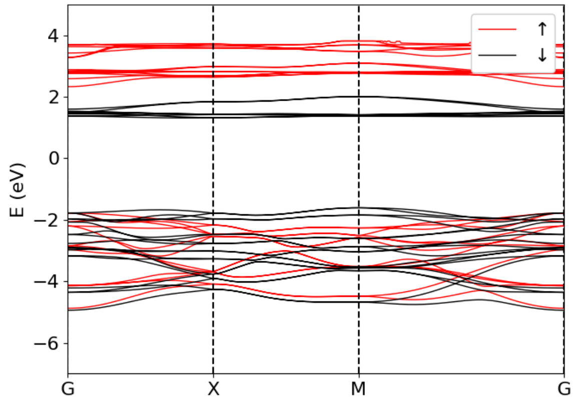


**Electronic bandstructure:** Spin-resolved energy bands of monolayer NdOBr along a high-symmetry path.

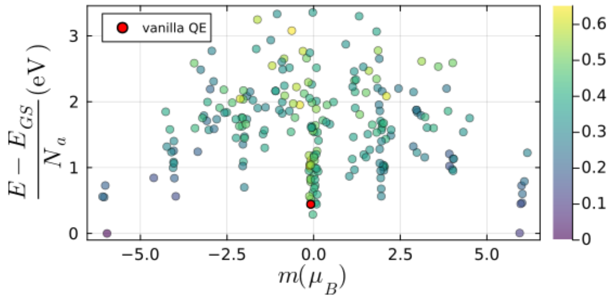


**Unique states:** Representation of 222 self-consistent unique states for monolayer NdOBr identified using RomeoDFT (see Section S6).

**Lattice vectors:** Cartesian components (in [Å]) of the lattice vectors for NdOBr.

| | $x$ | $y$ | $z$ |
|---|---|---|---|
| $\boldsymbol{a_1}$ | 3.9697 | 0.0000 | 0.0000 |
| $\boldsymbol{a_2}$ | 0.0000 | 3.9697 | 0.0000 |
| $\boldsymbol{a_3}$ | 0.0000 | 0.0000 | 25.4481 |

**Atomic positions:** Fractional coordinates, Hubbard $U$ (in eV) and magnetic moments (in $\mu_B$, computed from orbital occupations $m_o$ or integration spheres $m_i$) of each atom of monolayer NdOBr.

| atom | $x$ | $y$ | $z$ | $U$ | $m_o$ | $m_i$ |
|---|---|---|---|---|---|---|
| Nd | 0.0 | 0.50 | 0.55 | 3.38 | −2.98 | −2.88 |
| Nd | 0.50 | 0.0 | 0.45 | 3.38 | −2.98 | −2.88 |
| Br | 0.50 | 0.0 | 0.61 | 0.0 | – | 0.01 |
| Br | 0.0 | 0.50 | 0.39 | 0.0 | – | 0.01 |
| O | 0.0 | 0.0 | 0.50 | 0.0 | – | 0.02 |
| O | 0.50 | 0.50 | 0.50 | 0.0 | – | 0.02 |

# NdOI (AFM)

Band gap: 3.68 eV
Total magnetization: $-0.0\ \mu_B/cell$
Absolute magnetization: 6.16 $\mu_B/cell$
MC2D entry: https://mc2d.materialscloud.org/#/details/mc2d-2610

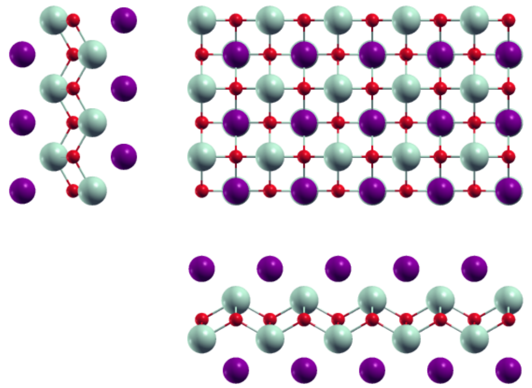

**Geometry:** Views of NdOI as seen from the $x$ axis (left), the $y$ axis (bottom), and the $z$ axis (center).

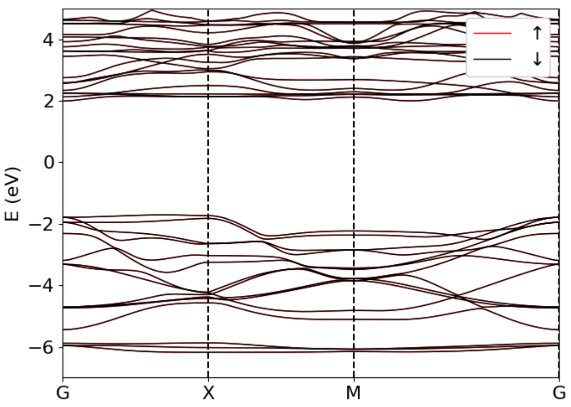


**Electronic bandstructure:** Spin-resolved energy bands of monolayer NdOI along a high-symmetry path.

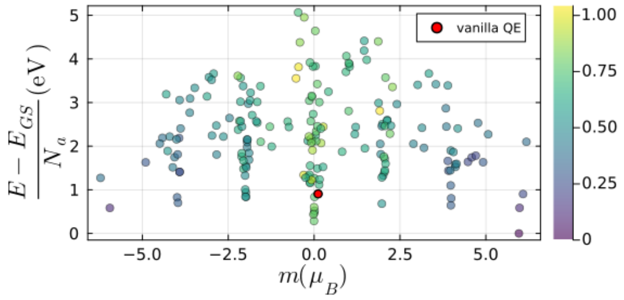


**Unique states:** Representation of 188 self-consistent unique states for monolayer NdOI identified using RomeoDFT (see Section S6).

**Lattice vectors:** Cartesian components (in [Å]) of the lattice vectors for NdOI.

| | $x$ | $y$ | $z$ |
|---|---|---|---|
| $\boldsymbol{a_1}$ | 4.0802 | 0.0000 | 0.0000 |
| $\boldsymbol{a_2}$ | 0.0000 | 4.0802 | 0.0000 |
| $\boldsymbol{a_3}$ | 0.0000 | 0.0000 | 23.9691 |

**Atomic positions:** Fractional coordinates, Hubbard $U$ (in eV) and magnetic moments (in $\mu_B$, computed from orbital occupations $m_o$ or integration spheres $m_i$) of each atom of monolayer NdOI.

| atom | $x$ | $y$ | $z$ | $U$ | $m_o$ | $m_i$ |
|---|---|---|---|---|---|---|
| Nd | 0.25 | −0.25 | 0.05 | 7.25 | 2.98 | 2.89 |
| Nd | −0.25 | −0.75 | −0.05 | 7.25 | −2.98 | −2.88 |
| I | −0.25 | −0.75 | 0.13 | 0.0 | – | −0.01 |
| I | 0.25 | −0.25 | −0.13 | 0.0 | – | 0.01 |
| O | −0.25 | −0.25 | 0.0 | 0.0 | – | 0.00 |
| O | 0.25 | −0.75 | 0.0 | 0.0 | – | 0.00 |

## NdSI (AFM)

Band gap: 2.53 eV
Total magnetization: 0.0 $\mu_B/cell$
Absolute magnetization: 6.24 $\mu_B/cell$
MC2D entry: https://mc2d.materialscloud.org/#/details/mc2d-1824

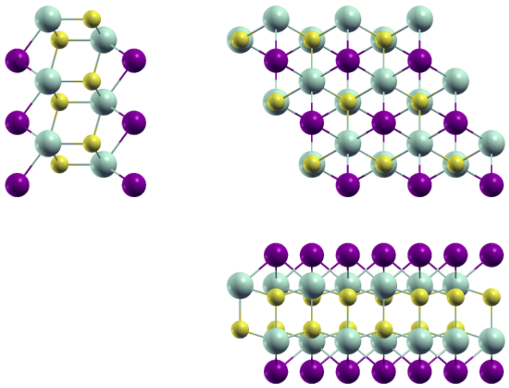

**Geometry:** Views of NdSI as seen from the $x$ axis (left), the $y$ axis (bottom), and the $z$ axis (center).

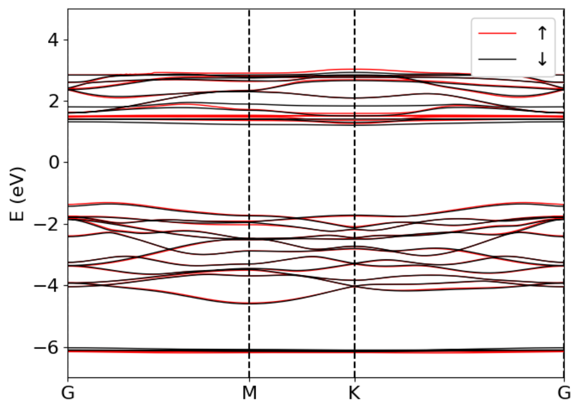


**Electronic bandstructure:** Spin-resolved energy bands of monolayer NdSI along a high-symmetry path.

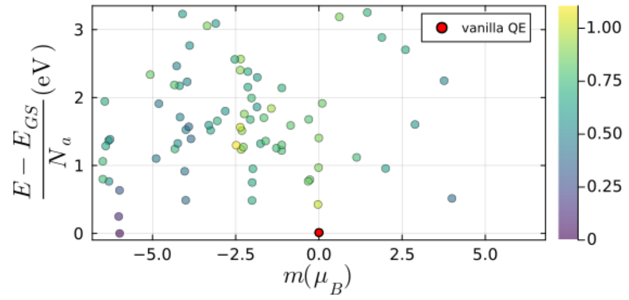


**Unique states:** Representation of 78 self-consistent unique states for monolayer NdSI identified using RomeoDFT (see Section S6).

**Lattice vectors:** Cartesian components (in [Å]) of the lattice vectors for NdSI.

| | $x$ | $y$ | $z$ |
|---|---|---|---|
| $\boldsymbol{a_1}$ | 2.3231 | −4.0237 | 0.0000 |
| $\boldsymbol{a_2}$ | 2.3231 | 4.0237 | 0.0000 |
| $\boldsymbol{a_3}$ | 0.0000 | 0.0000 | 26.9328 |

**Atomic positions:** Fractional coordinates, Hubbard $U$ (in eV) and magnetic moments (in $\mu_B$, computed from orbital occupations $m_o$ or integration spheres $m_i$) of each atom of monolayer NdSI.

| atom | $x$ | $y$ | $z$ | $U$ | $m_o$ | $m_i$ |
|---|---|---|---|---|---|---|
| Nd | −0.17 | −0.33 | 0.07 | 6.57 | 3.00 | 2.97 |
| Nd | 0.17 | 0.33 | −0.07 | 6.57 | −3.01 | −2.98 |
| S | −0.17 | −0.33 | −0.04 | 0.0 | – | 0.02 |
| S | 0.17 | 0.33 | 0.04 | 0.0 | – | −0.02 |
| I | 0.50 | 0.0 | 0.14 | 0.0 | – | −0.02 |
| I | 0.50 | 0.0 | −0.14 | 0.0 | – | 0.02 |

## $NiBr_2$ (FM)

Band gap: 1.89 eV
Total magnetization: $-4.0\ \mu_B/cell$
Absolute magnetization: $4.11\ \mu_B/cell$
MC2D entry: https://mc2d.materialscloud.org/#/details/mc2d-38

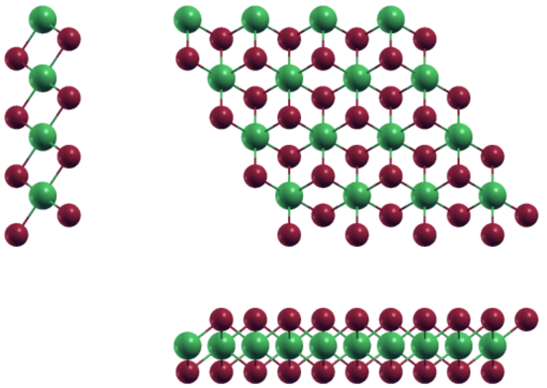

**Geometry:** Views of $NiBr_2$ as seen from the $x$ axis (left), the $y$ axis (bottom), and the $z$ axis (center).

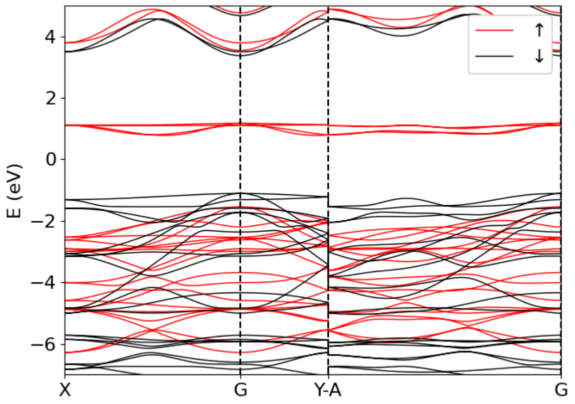


**Electronic bandstructure:** Spin-resolved energy bands of monolayer $NiBr_2$ along a high-symmetry path.

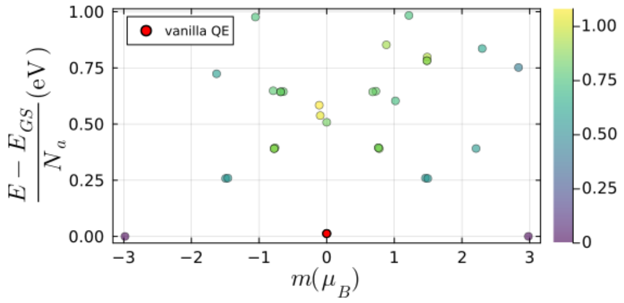


**Unique states:** Representation of 34 self-consistent unique states for monolayer $NiBr_2$ identified using RomeoDFT (see Section S6).

**Lattice vectors:** Cartesian components (in [Å]) of the lattice vectors for $NiBr_2$.

| | $x$ | $y$ | $z$ |
|---|---|---|---|
| $\boldsymbol{a_1}$ | 7.3343 | 0.0000 | 0.0000 |
| $\boldsymbol{a_2}$ | −1.8336 | 3.1758 | 0.0000 |
| $\boldsymbol{a_3}$ | 0.0000 | 0.0000 | 22.8371 |

**Atomic positions:** Fractional coordinates, Hubbard $U$ (in eV) and magnetic moments (in $\mu_B$, computed from orbital occupations $m_o$ or integration spheres $m_i$) of each atom of monolayer $NiBr_2$.

| atom | $x$ | $y$ | $z$ | $U$ | $m_o$ | $m_i$ |
|---|---|---|---|---|---|---|
| Ni | 0.17 | 0.67 | 0.50 | 5.40 | −1.49 | −1.59 |
| Ni | 0.67 | 0.67 | 0.50 | 5.40 | −1.49 | −1.59 |
| Br | 0.33 | 0.33 | 0.44 | 0.0 | – | −0.15 |
| Br | 0.50 | 1.00 | 0.56 | 0.0 | – | −0.15 |
| Br | 0.83 | 0.33 | 0.44 | 0.0 | – | −0.15 |
| Br | 1.00 | 1.00 | 0.56 | 0.0 | – | −0.15 |

## $NiC_4N_2Cl_2$ (FM)

Band gap: 0.0 eV
Total magnetization: 0.09 $\mu_B/cell$
Absolute magnetization: 6.21 $\mu_B/cell$
MC2D entry: https://mc2d.materialscloud.org/#/details/mc2d-2494

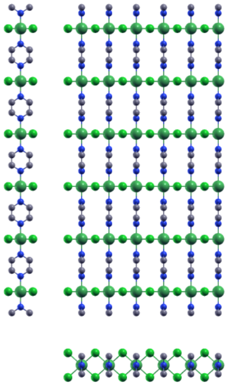

**Geometry:** Views of $NiC_4N_2Cl_2$ as seen from the $x$ axis (left), the $y$ axis (bottom), and the $z$ axis (center).

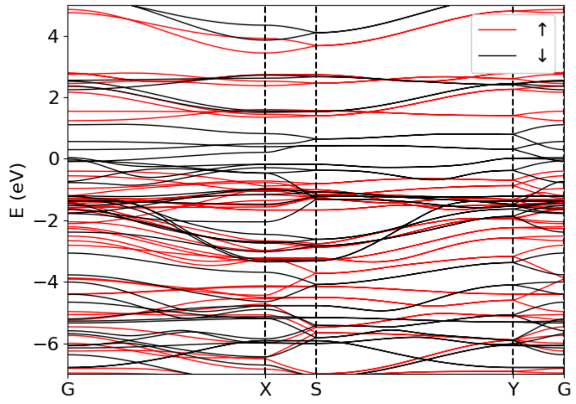


**Electronic bandstructure:** Spin-resolved energy bands of monolayer $NiC_4N_2Cl_2$ along a high-symmetry path.

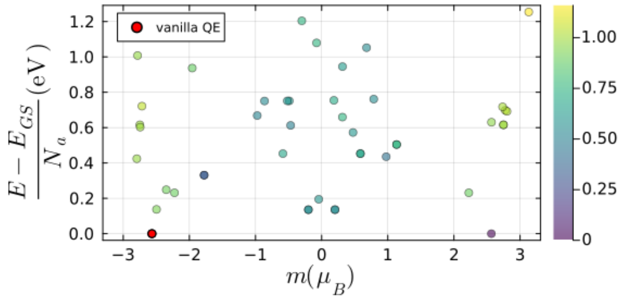


**Unique states:** Representation of 46 self-consistent unique states for monolayer $NiC_4N_2Cl_2$ identified using RomeoDFT (see Section S6).

**Lattice vectors:** Cartesian components (in [Å]) of the lattice vectors for $NiC_4N_2Cl_2$.

| | $x$ | $y$ | $z$ |
|---|---|---|---|
| $a_1$ | 3.4617 | 0.0000 | 0.0000 |
| $a_2$ | 0.0000 | 13.3793 | 0.0000 |
| $a_3$ | 0.0000 | 0.0000 | 17.9382 |

**Atomic positions:** Fractional coordinates, Hubbard $U$ (in eV) and magnetic moments (in $\mu_B$, computed from orbital occupations $m_o$ or integration spheres $m_i$) of each atom of monolayer $NiC_4N_2Cl_2$.

| atom | $x$ | $y$ | $z$ | $U$ | $m_o$ | $m_i$ |
|---|---|---|---|---|---|---|
| Ni | −0.50 | −0.25 | 0.0 | 6.81 | −1.28 | −1.35 |
| Ni | −0.50 | 0.25 | 0.0 | 6.81 | −1.28 | −1.35 |
| C | −0.50 | −0.05 | 0.06 | 0.0 | – | 0.12 |
| C | −0.50 | −0.45 | 0.06 | 0.0 | – | 0.12 |
| C | −0.50 | −0.05 | −0.06 | 0.0 | – | 0.12 |
| C | −0.50 | −0.45 | −0.06 | 0.0 | – | 0.12 |
| N | −0.50 | −0.10 | 0.0 | 0.0 | – | 0.05 |
| N | −0.50 | −0.40 | 0.0 | 0.0 | – | 0.05 |
| Cl | 0.0 | −0.25 | 0.09 | 0.0 | – | −0.02 |
| Cl | 0.0 | −0.25 | −0.09 | 0.0 | – | −0.02 |
| C | −0.50 | 0.45 | 0.06 | 0.0 | – | 0.12 |
| C | −0.50 | 0.05 | 0.06 | 0.0 | – | 0.12 |
| C | −0.50 | 0.45 | −0.06 | 0.0 | – | 0.12 |
| C | −0.50 | 0.05 | −0.06 | 0.0 | – | 0.12 |
| N | −0.50 | 0.40 | 0.0 | 0.0 | – | 0.05 |
| N | −0.50 | 0.10 | 0.0 | 0.0 | – | 0.05 |
| Cl | 0.0 | 0.25 | 0.09 | 0.0 | – | −0.02 |
| Cl | 0.0 | 0.25 | −0.09 | 0.0 | – | −0.02 |

## $NiCl_2$ (FM)

Band gap: 2.51 eV
Total magnetization: 4.0 $\mu_B/cell$
Absolute magnetization: 4.11 $\mu_B/cell$
MC2D entry: https://mc2d.materialscloud.org/#/details/mc2d-93

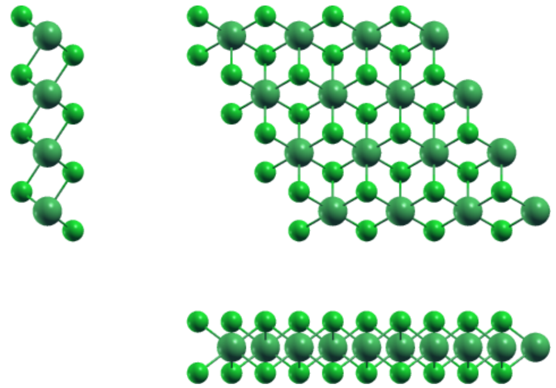

**Geometry:** Views of $NiCl_2$ as seen from the $x$ axis (left), the $y$ axis (bottom), and the $z$ axis (center).

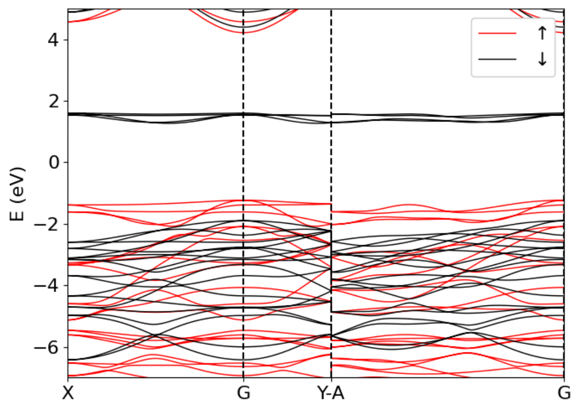


**Electronic bandstructure:** Spin-resolved energy bands of monolayer $NiCl_2$ along a high-symmetry path.

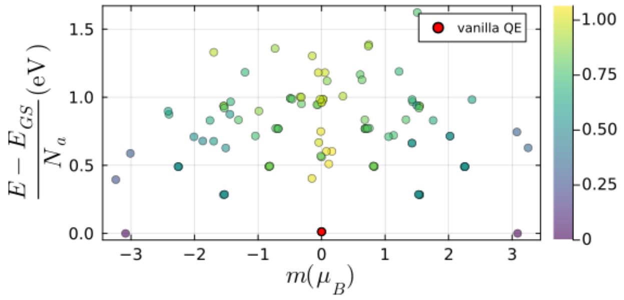


**Unique states:** Representation of 108 self-consistent unique states for monolayer $NiCl_2$ identified using RomeoDFT (see Section S6).

**Lattice vectors:** Cartesian components (in [Å]) of the lattice vectors for $NiCl_2$.

| | $x$ | $y$ | $z$ |
|---|---|---|---|
| $\boldsymbol{a_1}$ | 6.9465 | 0.0000 | 0.0000 |
| $\boldsymbol{a_2}$ | −1.7366 | 3.0079 | 0.0000 |
| $\boldsymbol{a_3}$ | 0.0000 | 0.0000 | 22.6403 |

**Atomic positions:** Fractional coordinates, Hubbard $U$ (in eV) and magnetic moments (in $\mu_B$, computed from orbital occupations $m_o$ or integration spheres $m_i$) of each atom of monolayer $NiCl_2$.

| atom | $x$ | $y$ | $z$ | $U$ | $m_o$ | $m_i$ |
|---|---|---|---|---|---|---|
| Ni | 0.33 | 0.33 | 0.50 | 5.34 | 1.54 | 1.64 |
| Ni | 0.83 | 0.33 | 0.50 | 5.34 | 1.54 | 1.64 |
| Cl | 0.0 | 0.0 | 0.44 | 0.0 | – | 0.14 |
| Cl | 0.17 | 0.67 | 0.56 | 0.0 | – | 0.14 |
| Cl | 0.50 | 0.0 | 0.44 | 0.0 | – | 0.14 |
| Cl | 0.67 | 0.67 | 0.56 | 0.0 | – | 0.14 |

## $NiGa_2S_4$ (AFM)

Band gap: 1.02 eV
Total magnetization: $-0.0\ \mu_B/cell$
Absolute magnetization: $3.7\ \mu_B/cell$
MC2D entry: https://mc2d.materialscloud.org/#/details/mc2d-960

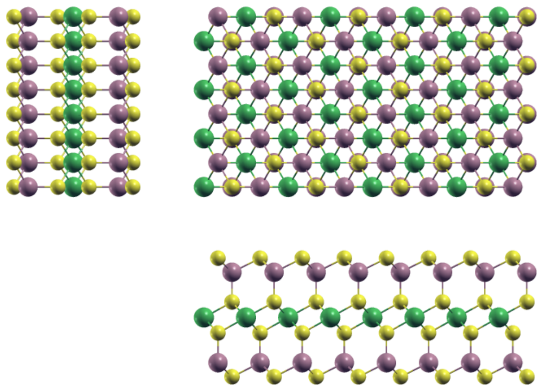

**Geometry:** Views of $NiGa_2S_4$ as seen from the $x$ axis (left), the $y$ axis (bottom), and the $z$ axis (center).

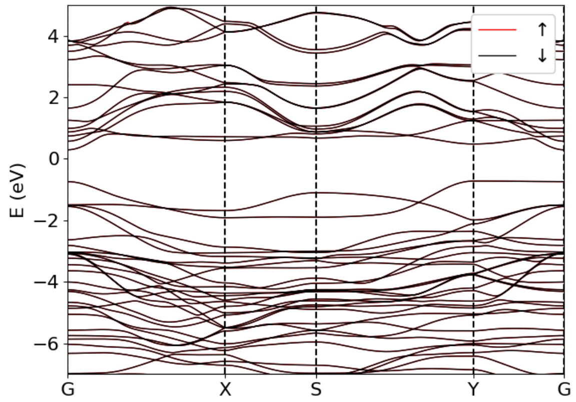


**Electronic bandstructure:** Spin-resolved energy bands of monolayer $NiGa_2S_4$ along a high-symmetry path.

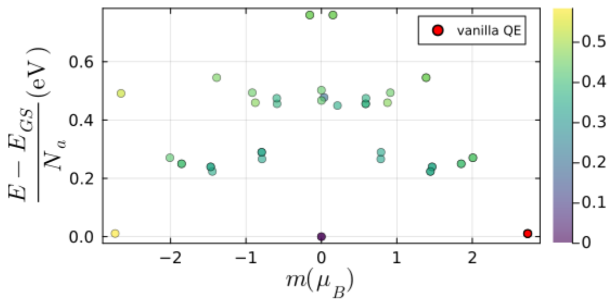


**Unique states:** Representation of 45 self-consistent unique states for monolayer $NiGa_2S_4$ identified using RomeoDFT (see Section S6).

**Lattice vectors:** Cartesian components (in [Å]) of the lattice vectors for $NiGa_2S_4$.

| | $x$ | $y$ | $z$ |
|---|---|---|---|
| $\boldsymbol{a_1}$ | 0.0000 | −6.3166 | 0.0000 |
| $\boldsymbol{a_2}$ | 3.6469 | 0.0000 | 0.0000 |
| $\boldsymbol{a_3}$ | 0.0000 | 0.0000 | 29.0044 |

**Atomic positions:** Fractional coordinates, Hubbard $U$ (in eV) and magnetic moments (in $\mu_B$, computed from orbital occupations $m_o$ or integration spheres $m_i$) of each atom of monolayer $NiGa_2S_4$.

| atom | $x$ | $y$ | $z$ | $U$ | $m_o$ | $m_i$ |
|---|---|---|---|---|---|---|
| Ni | 0.50 | 0.50 | 0.50 | 5.82 | −1.35 | −1.47 |
| Ni | 1.00 | 1.00 | 0.50 | 5.82 | 1.35 | 1.47 |
| Ga | 0.33 | 1.00 | 0.38 | 0.0 | – | 0.00 |
| Ga | 0.83 | 0.50 | 0.38 | 0.0 | – | 0.00 |
| Ga | 1.17 | 0.50 | 0.62 | 0.0 | – | 0.00 |
| Ga | 0.67 | 1.00 | 0.62 | 0.0 | – | 0.00 |
| S | 0.83 | 0.50 | 0.46 | 0.0 | – | 0.04 |
| S | 0.33 | 1.00 | 0.46 | 0.0 | – | −0.04 |
| S | 0.67 | 1.00 | 0.54 | 0.0 | – | −0.04 |
| S | 1.17 | 0.50 | 0.54 | 0.0 | – | 0.04 |
| S | 0.83 | 0.50 | 0.65 | 0.0 | – | −0.02 |
| S | 0.33 | 1.00 | 0.65 | 0.0 | – | 0.02 |
| S | 0.67 | 1.00 | 0.35 | 0.0 | – | 0.02 |
| S | 1.17 | 0.50 | 0.35 | 0.0 | – | −0.02 |

# $NiI_2$ (FM)

Band gap: 1.19 eV
Total magnetization: 4.0 $\mu_B/cell$
Absolute magnetization: 4.15 $\mu_B/cell$
MC2D entry: https://mc2d.materialscloud.org/#/details/mc2d-182

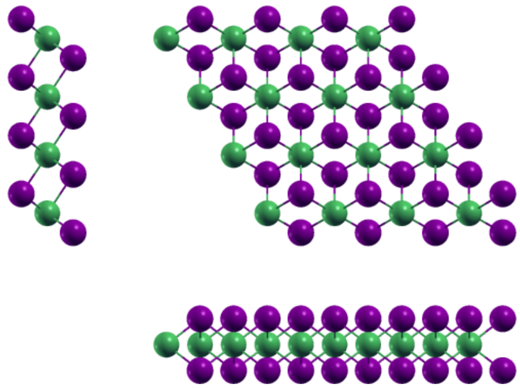

**Geometry:** Views of $NiI_2$ as seen from the $x$ axis (left), the $y$ axis (bottom), and the $z$ axis (center).

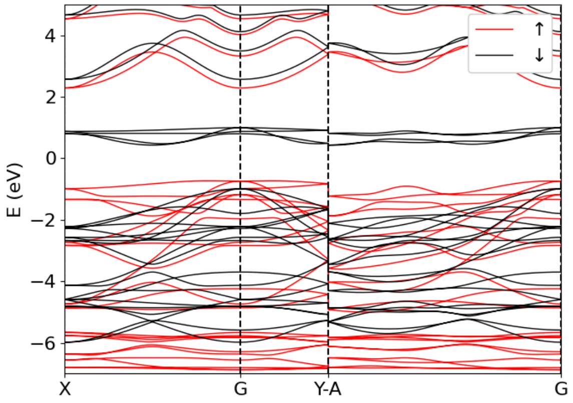


**Electronic bandstructure:** Spin-resolved energy bands of monolayer $NiI_2$ along a high-symmetry path.

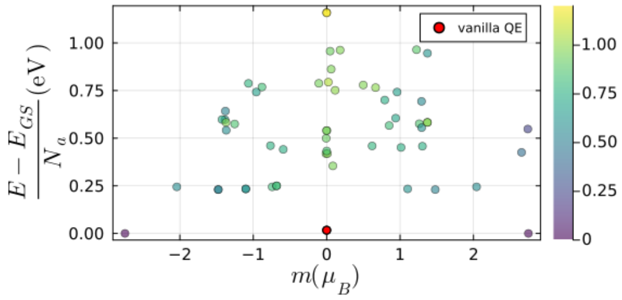


**Unique states:** Representation of 60 self-consistent unique states for monolayer $NiI_2$ identified using RomeoDFT (see Section S6).

**Lattice vectors:** Cartesian components (in [Å]) of the lattice vectors for $NiI_2$.

| | $x$ | $y$ | $z$ |
|---|---|---|---|
| $\boldsymbol{a_1}$ | 3.9451 | −6.8332 | 0.0000 |
| $\boldsymbol{a_2}$ | 1.9726 | 3.4166 | 0.0000 |
| $\boldsymbol{a_3}$ | 0.0000 | 0.0000 | 18.1096 |

**Atomic positions:** Fractional coordinates, Hubbard $U$ (in eV) and magnetic moments (in $\mu_B$, computed from orbital occupations $m_o$ or integration spheres $m_i$) of each atom of monolayer $NiI_2$.

| atom | $x$ | $y$ | $z$ | $U$ | $m_o$ | $m_i$ |
|---|---|---|---|---|---|---|
| Ni | 0.0 | 0.50 | 0.0 | 5.57 | 1.37 | 1.47 |
| Ni | 0.50 | 0.50 | 0.0 | 5.57 | 1.37 | 1.47 |
| I | 0.17 | 0.17 | −0.08 | 0.0 | – | 0.16 |
| I | 0.33 | 0.83 | 0.08 | 0.0 | – | 0.16 |
| I | 0.67 | 0.17 | −0.08 | 0.0 | – | 0.16 |
| I | 0.83 | 0.83 | 0.08 | 0.0 | – | 0.16 |

## $NiPS_3$ (AFM)

Band gap: 1.84 eV
Total magnetization: $-0.0\ \mu_B/cell$
Absolute magnetization: $3.61\ \mu_B/cell$
MC2D entry: https://mc2d.materialscloud.org/#/details/mc2d-1161

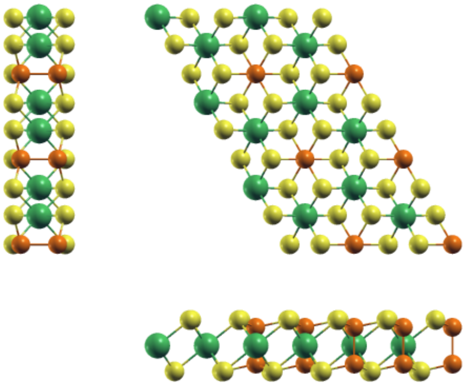

**Geometry:** Views of $NiPS_3$ as seen from the $x$ axis (left), the $y$ axis (bottom), and the $z$ axis (center).

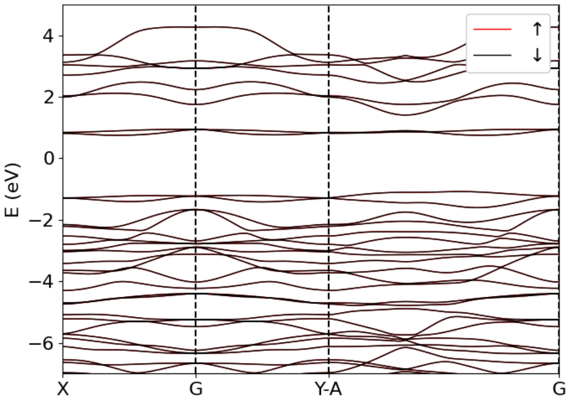


**Electronic bandstructure:** Spin-resolved energy bands of monolayer $NiPS_3$ along a high-symmetry path.

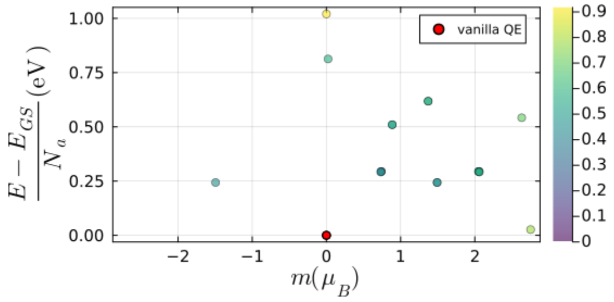


**Unique states:** Representation of 21 self-consistent unique states for monolayer $NiPS_3$ identified using RomeoDFT (see Section S6).

**Lattice vectors:** Cartesian components (in [Å]) of the lattice vectors for $NiPS_3$.

| | $x$ | $y$ | $z$ |
|---|---|---|---|
| $\boldsymbol{a_1}$ | 5.8283 | 0.0000 | 0.0000 |
| $\boldsymbol{a_2}$ | −2.9142 | 5.0527 | 0.0000 |
| $\boldsymbol{a_3}$ | 0.0000 | 0.0000 | 22.9410 |

**Atomic positions:** Fractional coordinates, Hubbard $U$ (in eV) and magnetic moments (in $\mu_B$, computed from orbital occupations $m_o$ or integration spheres $m_i$) of each atom of monolayer $NiPS_3$.

| atom | $x$ | $y$ | $z$ | $U$ | $m_o$ | $m_i$ |
|---|---|---|---|---|---|---|
| Ni | 0.34 | 0.33 | 0.50 | 5.85 | 1.35 | 1.46 |
| Ni | 0.01 | 0.67 | 0.50 | 5.85 | −1.35 | −1.46 |
| P | 0.67 | 0.0 | 0.45 | 0.0 | – | 0.0 |
| P | 0.67 | 0.0 | 0.55 | 0.0 | – | 0.0 |
| S | 0.33 | 0.0 | 0.57 | 0.0 | – | 0.0 |
| S | 0.02 | 0.35 | 0.57 | 0.0 | – | 0.00 |
| S | 0.67 | 0.65 | 0.57 | 0.0 | – | 0.00 |
| S | 0.02 | 0.0 | 0.43 | 0.0 | – | 0.0 |
| S | 0.33 | 0.65 | 0.43 | 0.0 | – | 0.00 |
| S | 0.67 | 0.35 | 0.43 | 0.0 | – | 0.00 |

## $NiPSe_3$ (AFM)

Band gap: 1.32 eV
Total magnetization: $-0.0$ $\mu_B/cell$
Absolute magnetization: 3.49 $\mu_B/cell$
MC2D entry: https://mc2d.materialscloud.org/#/details/mc2d-1418

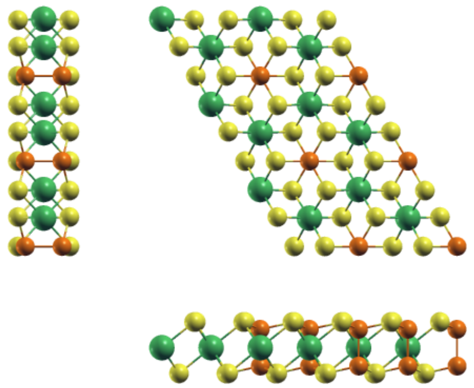

**Geometry:** Views of $NiPSe_3$ as seen from the $x$ axis (left), the $y$ axis (bottom), and the $z$ axis (center).

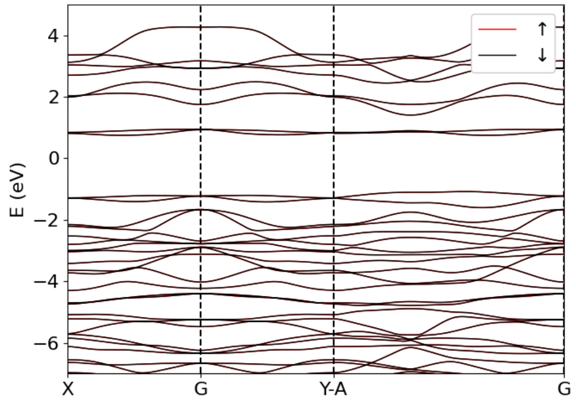


**Electronic bandstructure:** Spin-resolved energy bands of monolayer $NiPSe_3$ along a high-symmetry path.

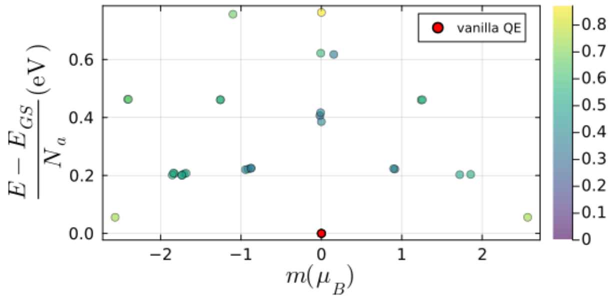


**Unique states:** Representation of 33 self-consistent unique states for monolayer $NiPSe_3$ identified using RomeoDFT (see Section S6).

**Lattice vectors:** Cartesian components (in [Å]) of the lattice vectors for $NiPSe_3$.

| | $x$ | $y$ | $z$ |
|---|---|---|---|
| $a_1$ | 6.1734 | 0.0000 | 0.0000 |
| $a_2$ | 3.0867 | 5.3532 | 0.0000 |
| $a_3$ | 0.0000 | 0.0000 | 23.0387 |

**Atomic positions:** Fractional coordinates, Hubbard $U$ (in eV) and magnetic moments (in $\mu_B$, computed from orbital occupations $m_o$ or integration spheres $m_i$) of each atom of monolayer $NiPSe_3$.

| atom | $x$ | $y$ | $z$ | $U$ | $m_o$ | $m_i$ |
|---|---|---|---|---|---|---|
| Ni | 0.01 | 0.33 | 0.50 | 6.01 | 1.25 | 1.35 |
| Ni | 0.34 | 0.67 | 0.50 | 6.01 | −1.25 | −1.35 |
| P | 0.67 | 0.0 | 0.45 | 0.0 | – | 0.0 |
| P | 0.67 | 0.0 | 0.55 | 0.0 | – | 0.0 |
| Se | 0.32 | 0.0 | 0.57 | 0.0 | – | 0.0 |
| Se | 0.67 | 0.36 | 0.57 | 0.0 | – | 0.00 |
| Se | 0.03 | 0.64 | 0.57 | 0.0 | – | 0.00 |
| Se | 0.03 | 0.0 | 0.43 | 0.0 | – | 0.0 |
| Se | 0.67 | 0.64 | 0.43 | 0.0 | – | 0.00 |
| Se | 0.32 | 0.36 | 0.43 | 0.0 | – | 0.00 |

# $NiTl_2F_4$ (AFM)

Band gap: 3.63 eV
Total magnetization: $-0.0\ \mu_B/cell$
Absolute magnetization: 3.91 $\mu_B/cell$
MC2D entry: https://mc2d.materialscloud.org/#/details/mc2d-2020

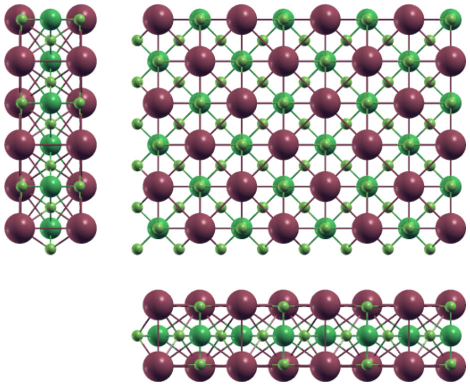

**Geometry:** Views of $NiTl_2F_4$ as seen from the $x$ axis (left), the $y$ axis (bottom), and the $z$ axis (center).

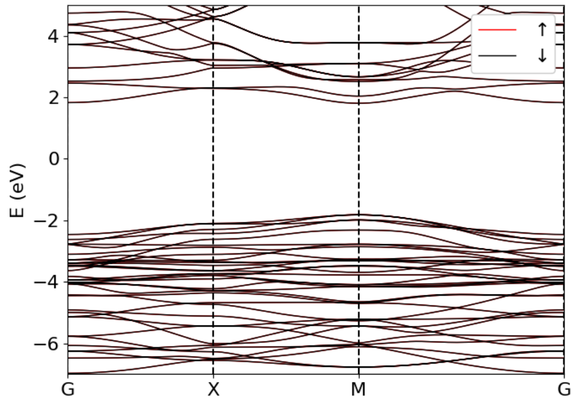


**Electronic bandstructure:** Spin-resolved energy bands of monolayer $NiTl_2F_4$ along a high-symmetry path.

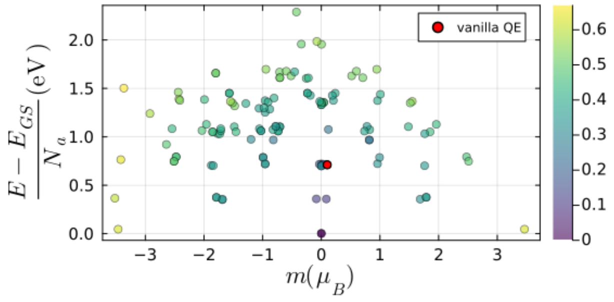


$$\frac{E - E_{GS}}{N_a}\ (eV)$$

$$m(\mu_B)$$

**Unique states:** Representation of 126 self-consistent unique states for monolayer $NiTl_2F_4$ identified using RomeoDFT (see Section S6).

**Lattice vectors:** Cartesian components (in [Å]) of the lattice vectors for $NiTl_2F_4$.

| | $x$ | $y$ | $z$ |
|---|---|---|---|
| $\boldsymbol{a_1}$ | −4.0918 | 4.0918 | 0.0000 |
| $\boldsymbol{a_2}$ | −4.0918 | −4.0918 | 0.0000 |
| $\boldsymbol{a_3}$ | 0.0000 | 0.0000 | 19.9974 |

**Atomic positions:** Fractional coordinates, Hubbard $U$ (in eV) and magnetic moments (in $\mu_B$, computed from orbital occupations $m_o$ or integration spheres $m_i$) of each atom of monolayer $NiTl_2F_4$.

| atom | $x$ | $y$ | $z$ | $U$ | $m_o$ | $m_i$ |
|---|---|---|---|---|---|---|
| Ni | 0.75 | 0.75 | 0.0 | 5.11 | −1.71 | −1.73 |
| Ni | 0.25 | 0.25 | 0.0 | 5.11 | 1.71 | 1.73 |
| Tl | 0.75 | 1.25 | 0.89 | 0.0 | – | 0.0 |
| Tl | 1.25 | 0.75 | 0.89 | 0.0 | – | 0.0 |
| Tl | 0.25 | 0.75 | 0.11 | 0.0 | – | 0.0 |
| Tl | 0.75 | 0.25 | 0.11 | 0.0 | – | 0.0 |
| F | 0.75 | 0.75 | 0.90 | 0.0 | – | −0.03 |
| F | 1.25 | 1.25 | 0.90 | 0.0 | – | 0.03 |
| F | 0.25 | 0.25 | 0.10 | 0.0 | – | 0.03 |
| F | 0.75 | 0.75 | 0.10 | 0.0 | – | −0.03 |
| F | 0.50 | 0.0 | 0.0 | 0.0 | – | 0.0 |
| F | 0.0 | 0.50 | 0.0 | 0.0 | – | 0.0 |
| F | 0.50 | 0.50 | 0.0 | 0.0 | – | 0.0 |
| F | 0.0 | 0.0 | 0.0 | 0.0 | – | 0.0 |

## $PrBr_3$ (FM)

Band gap: 1.73 eV
Total magnetization: 4.0 $\mu_B/cell$
Absolute magnetization: 4.25 $\mu_B/cell$
MC2D entry: https://mc2d.materialscloud.org/#/details/mc2d-2670

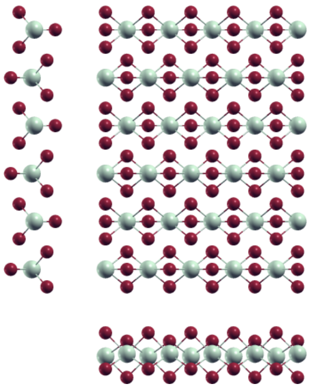

**Geometry:** Views of $PrBr_3$ as seen from the $x$ axis (left), the $y$ axis (bottom), and the $z$ axis (center).

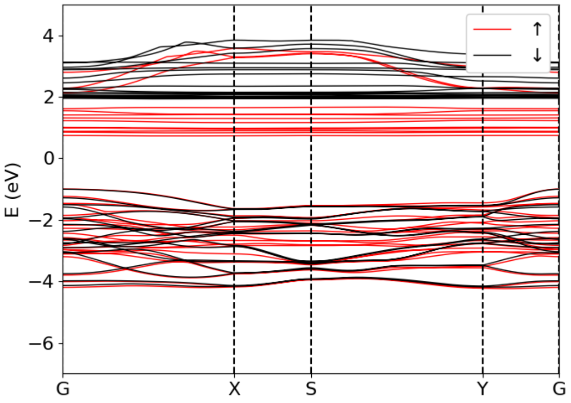


**Electronic bandstructure:** Spin-resolved energy bands of monolayer $PrBr_3$ along a high-symmetry path.

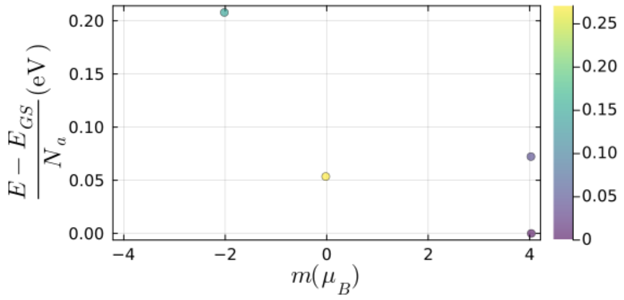


**Unique states:** Representation of 4 self-consistent unique states for monolayer $PrBr_3$ identified using RomeoDFT (see Section S6).

**Lattice vectors:** Cartesian components (in [Å]) of the lattice vectors for $PrBr_3$.

| | $x$ | $y$ | $z$ |
|---|---|---|---|
| $\boldsymbol{a_1}$ | 4.1423 | 0.0000 | 0.0000 |
| $\boldsymbol{a_2}$ | 0.0000 | 9.3088 | 0.0000 |
| $\boldsymbol{a_3}$ | 0.0000 | 0.0000 | 20.5299 |

**Atomic positions:** Fractional coordinates, Hubbard $U$ (in eV) and magnetic moments (in $\mu_B$, computed from orbital occupations $m_o$ or integration spheres $m_i$) of each atom of monolayer $PrBr_3$.

| atom | $x$ | $y$ | $z$ | $U$ | $m_o$ | $m_i$ |
|---|---|---|---|---|---|---|
| Pr | −0.25 | −0.25 | −0.01 | 3.09 | 2.02 | 1.99 |
| Pr | 0.25 | −0.75 | 0.01 | 3.09 | 2.01 | 1.99 |
| Br | 0.25 | −0.07 | 0.07 | 0.0 | – | −0.02 |
| Br | 0.25 | −0.43 | 0.07 | 0.0 | – | −0.02 |
| Br | −0.25 | −0.57 | −0.07 | 0.0 | – | −0.02 |
| Br | −0.25 | −0.93 | −0.07 | 0.0 | – | −0.02 |
| Br | 0.25 | −0.25 | −0.11 | 0.0 | – | −0.02 |
| Br | −0.25 | −0.75 | 0.11 | 0.0 | – | −0.02 |

# $PrI_2$ (FM)

Band gap: 0.29 eV
Total magnetization: 6.0 $\mu_B/cell$
Absolute magnetization: 6.17 $\mu_B/cell$
MC2D entry: https://mc2d.materialscloud.org/#/details/mc2d-2230

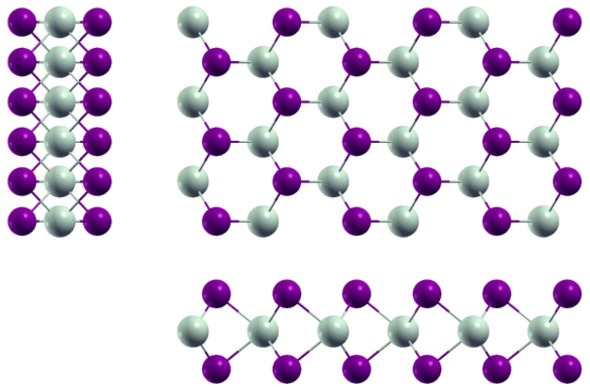

**Geometry:** Views of $PrI_2$ as seen from the $x$ axis (left), the $y$ axis (bottom), and the $z$ axis (center).

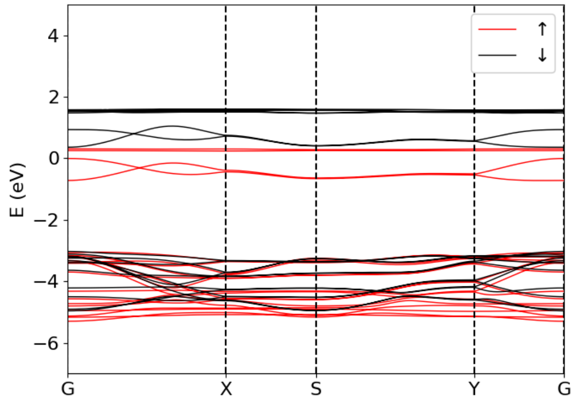


**Electronic bandstructure:** Spin-resolved energy bands of monolayer $PrI_2$ along a high-symmetry path.

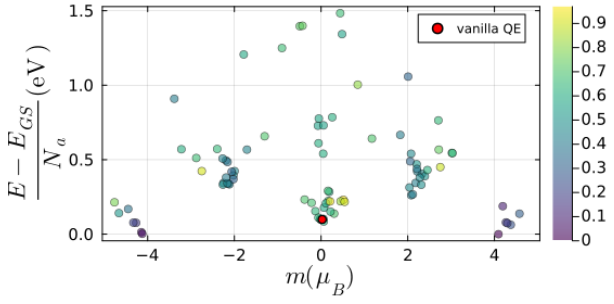


**Unique states:** Representation of 89 self-consistent unique states for monolayer $PrI_2$ identified using RomeoDFT (see Section S6).

**Lattice vectors:** Cartesian components (in [Å]) of the lattice vectors for $PrI_2$.

| | $x$ | $y$ | $z$ |
|---|---|---|---|
| $\boldsymbol{a_1}$ | −6.6186 | −3.8213 | 0.0000 |
| $\boldsymbol{a_2}$ | 2.1702 | −3.7589 | 0.0000 |
| $\boldsymbol{a_3}$ | 0.0000 | 0.0000 | 20.2533 |

**Atomic positions:** Fractional coordinates, Hubbard $U$ (in eV) and magnetic moments (in $\mu_B$, computed from orbital occupations $m_o$ or integration spheres $m_i$) of each atom of monolayer $PrI_2$.

| atom | $x$ | $y$ | $z$ | $U$ | $m_o$ | $m_i$ |
|---|---|---|---|---|---|---|
| Pr | 0.66 | 1.00 | 0.0 | 4.91 | 2.04 | 2.40 |
| Pr | 0.17 | 0.50 | 0.0 | 4.72 | 2.05 | 2.39 |
| I | 0.84 | 0.50 | 0.90 | 0.0 | – | −0.01 |
| I | 1.33 | 0.0 | 0.90 | 0.0 | – | −0.01 |
| I | 0.33 | 0.0 | 0.10 | 0.0 | – | −0.01 |
| I | 0.84 | 0.50 | 0.10 | 0.0 | – | −0.01 |

## $PrI_3$ (FM)

Band gap: 1.16 eV
Total magnetization: −4.0 $\mu_B/cell$
Absolute magnetization: 4.35 $\mu_B/cell$
MC2D entry: https://mc2d.materialscloud.org/#/details/mc2d-2556

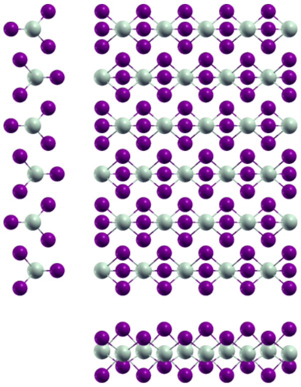

**Geometry:** Views of $PrI_3$ as seen from the $x$ axis (left), the $y$ axis (bottom), and the $z$ axis (center).

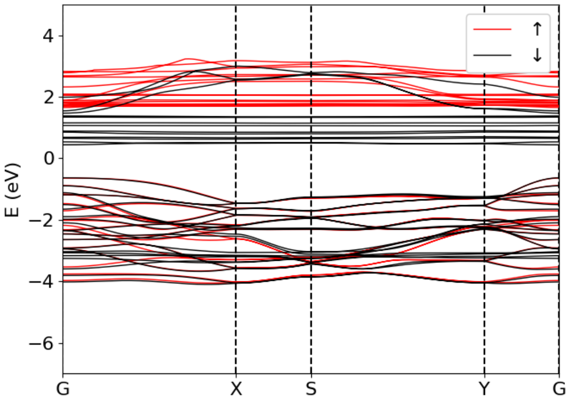


**Electronic bandstructure:** Spin-resolved energy bands of monolayer $PrI_3$ along a high-symmetry path.

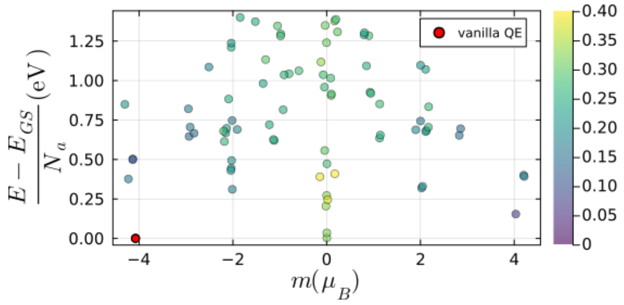


**Unique states:** Representation of 82 self-consistent unique states for monolayer $PrI_3$ identified using RomeoDFT (see Section S6).

**Lattice vectors:** Cartesian components (in [Å]) of the lattice vectors for $PrI_3$.

| | $x$ | $y$ | $z$ |
|---|---|---|---|
| $a_1$ | 4.3405 | 0.0000 | 0.0000 |
| $a_2$ | 0.0000 | 10.0668 | 0.0000 |
| $a_3$ | 0.0000 | 0.0000 | 21.4869 |

**Atomic positions:** Fractional coordinates, Hubbard $U$ (in eV) and magnetic moments (in $\mu_B$, computed from orbital occupations $m_o$ or integration spheres $m_i$) of each atom of monolayer $PrI_3$.

| atom | $x$ | $y$ | $z$ | $U$ | $m_o$ | $m_i$ |
|---|---|---|---|---|---|---|
| Pr | 0.25 | −0.75 | −0.01 | 3.27 | −2.04 | −2.02 |
| Pr | −0.25 | −0.25 | 0.01 | 3.27 | −2.04 | −2.02 |
| I | −0.25 | −0.93 | 0.07 | 0.0 | – | 0.02 |
| I | −0.25 | −0.57 | 0.07 | 0.0 | – | 0.02 |
| I | 0.25 | −0.43 | −0.07 | 0.0 | – | 0.02 |
| I | 0.25 | −0.07 | −0.07 | 0.0 | – | 0.02 |
| I | −0.25 | −0.75 | −0.11 | 0.0 | – | 0.02 |
| I | 0.25 | −0.25 | 0.11 | 0.0 | – | 0.02 |

# PrOI (FM)

Band gap: 2.29 eV
Total magnetization: 4.0 $\mu_B/cell$
Absolute magnetization: 4.2 $\mu_B/cell$
MC2D entry: https://mc2d.materialscloud.org/#/details/mc2d-195

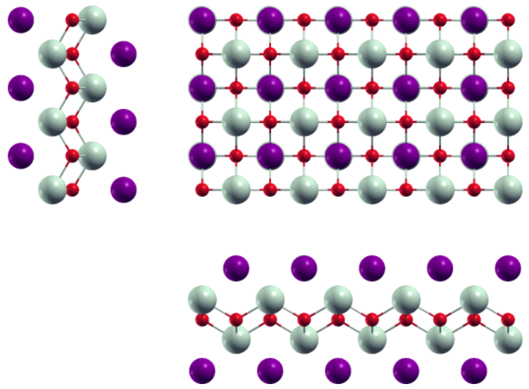

**Geometry:** Views of PrOI as seen from the $x$ axis (left), the $y$ axis (bottom), and the $z$ axis (center).

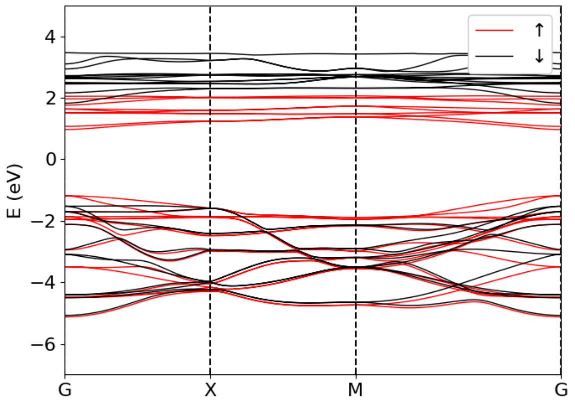


**Electronic bandstructure:** Spin-resolved energy bands of monolayer PrOI along a high-symmetry path.

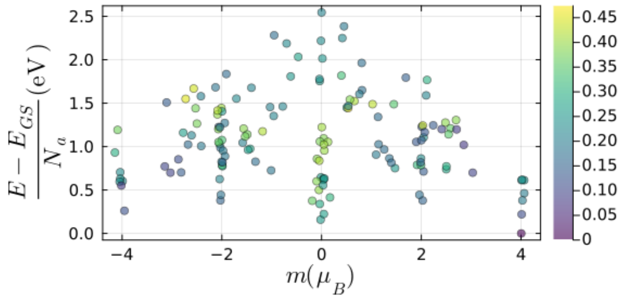


**Unique states:** Representation of 143 self-consistent unique states for monolayer PrOI identified using RomeoDFT (see Section S6).

**Lattice vectors:** Cartesian components (in [Å]) of the lattice vectors for PrOI.

| | $x$ | $y$ | $z$ |
|---|---|---|---|
| $\boldsymbol{a_1}$ | 4.0755 | 0.0000 | 0.0000 |
| $\boldsymbol{a_2}$ | 0.0000 | 4.0755 | 0.0000 |
| $\boldsymbol{a_3}$ | 0.0000 | 0.0000 | 24.3141 |

**Atomic positions:** Fractional coordinates, Hubbard $U$ (in eV) and magnetic moments (in $\mu_B$, computed from orbital occupations $m_o$ or integration spheres $m_i$) of each atom of monolayer PrOI.

| atom | $x$ | $y$ | $z$ | $U$ | $m_o$ | $m_i$ |
|---|---|---|---|---|---|---|
| Pr | −0.25 | −0.75 | 0.05 | 2.99 | 2.00 | 1.90 |
| Pr | 0.25 | −0.25 | −0.05 | 2.99 | 2.00 | 1.90 |
| I | 0.25 | −0.25 | 0.13 | 0.0 | – | −0.01 |
| I | −0.25 | −0.75 | −0.13 | 0.0 | – | −0.01 |
| O | −0.25 | −0.25 | 0.0 | 0.0 | – | −0.03 |
| O | 0.25 | −0.75 | 0.0 | 0.0 | – | −0.03 |

# $Re_2CoO_8$ (FM)

Band gap: 3.35 eV
Total magnetization: 6.0 $\mu_B/cell$
Absolute magnetization: 6.11 $\mu_B/cell$
MC2D entry: https://mc2d.materialscloud.org/#/details/mc2d-490

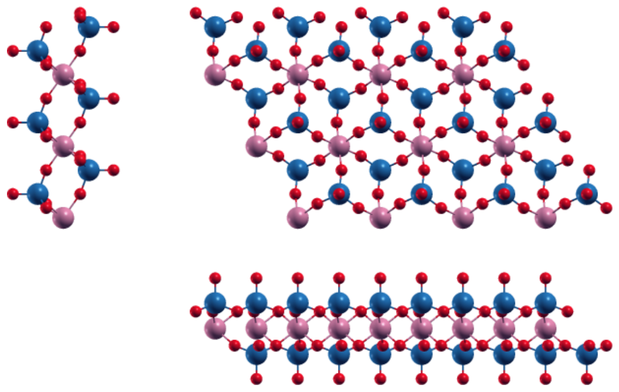

**Geometry:** Views of $Re_2CoO_8$ as seen from the $x$ axis (left), the $y$ axis (bottom), and the $z$ axis (center).

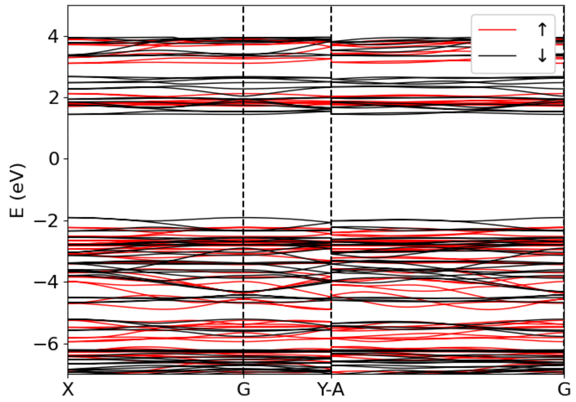


**Electronic bandstructure:** Spin-resolved energy bands of monolayer $Re_2CoO_8$ along a high-symmetry path.

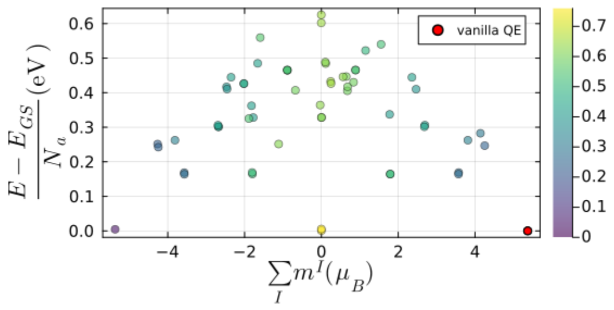


**Unique states:** Representation of 60 self-consistent unique states for monolayer $Re_2CoO_8$ identified using RomeoDFT (see Section S6).

**Lattice vectors:** Cartesian components (in [Å]) of the lattice vectors for $Re_2CoO_8$.

| | $x$ | $y$ | $z$ |
|---|---|---|---|
| $a_1$ | 11.4106 | 0.0000 | 0.0000 |
| $a_2$ | −2.8526 | 4.9409 | 0.0000 |
| $a_3$ | 0.0000 | 0.0000 | 26.8962 |

**Atomic positions:** Fractional coordinates, Hubbard $U$ (in eV) and magnetic moments (in $\mu_B$, computed from orbital occupations $m_o$ or integration spheres $m_i$) of each atom of monolayer $Re_2CoO_8$.

| atom | $x$ | $y$ | $z$ | $U$ | $m_o$ | $m_i$ |
|---|---|---|---|---|---|---|
| Re | 0.33 | 0.33 | 0.57 | 4.04 | 0.03 | 0.02 |
| Co | 0.0 | 0.0 | 0.50 | 4.80 | 2.62 | 2.61 |
| Re | 0.17 | 0.67 | 0.43 | 4.04 | 0.03 | 0.02 |
| Re | 0.83 | 0.33 | 0.57 | 4.04 | 0.03 | 0.02 |
| Co | 0.50 | 0.0 | 0.50 | 4.80 | 2.62 | 2.61 |
| Re | 0.67 | 0.67 | 0.43 | 4.04 | 0.03 | 0.02 |
| O | 0.17 | 0.20 | 0.54 | 0.0 | – | 0.02 |
| O | 0.43 | 0.67 | 0.54 | 0.0 | – | 0.02 |
| O | 0.40 | 0.14 | 0.54 | 0.0 | – | 0.02 |
| O | 0.33 | 0.33 | 0.63 | 0.0 | – | 0.00 |
| O | 0.33 | 0.80 | 0.46 | 0.0 | – | 0.02 |
| O | 0.07 | 0.33 | 0.46 | 0.0 | – | 0.02 |
| O | 0.10 | 0.86 | 0.46 | 0.0 | – | 0.02 |
| O | 0.17 | 0.67 | 0.37 | 0.0 | – | 0.00 |
| O | 0.67 | 0.20 | 0.54 | 0.0 | – | 0.02 |
| O | 0.93 | 0.67 | 0.54 | 0.0 | – | 0.02 |
| O | 0.90 | 0.14 | 0.54 | 0.0 | – | 0.02 |
| O | 0.83 | 0.33 | 0.63 | 0.0 | – | 0.00 |
| O | 0.83 | 0.80 | 0.46 | 0.0 | – | 0.02 |
| O | 0.57 | 0.33 | 0.46 | 0.0 | – | 0.02 |
| O | 0.60 | 0.86 | 0.46 | 0.0 | – | 0.02 |
| O | 0.67 | 0.67 | 0.37 | 0.0 | – | 0.00 |

## $Re_2NiO_8$ (AFM)

Band gap: 3.65 eV
Total magnetization: 0.0 $\mu_B/cell$
Absolute magnetization: 4.04 $\mu_B/cell$
MC2D entry: https://mc2d.materialscloud.org/#/details/mc2d-541

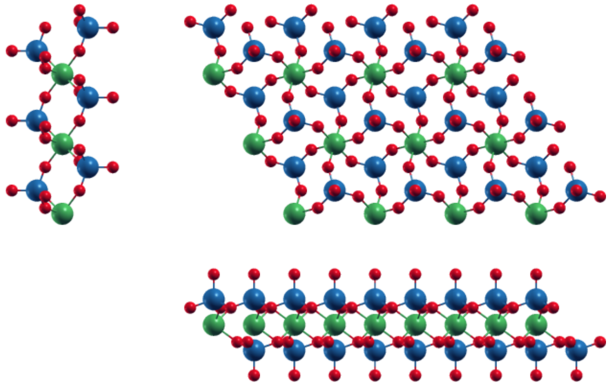

**Geometry:** Views of $Re_2NiO_8$ as seen from the $x$ axis (left), the $y$ axis (bottom), and the $z$ axis (center).

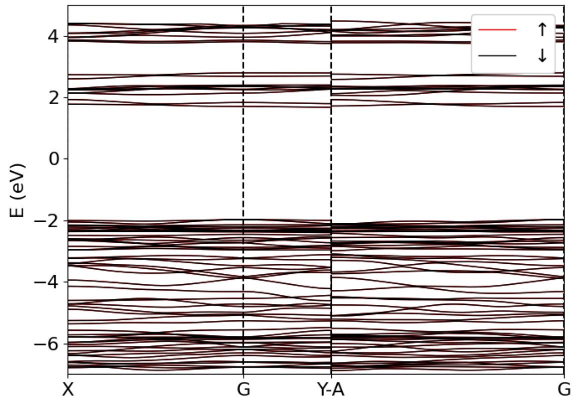


**Electronic bandstructure:** Spin-resolved energy bands of monolayer $Re_2NiO_8$ along a high-symmetry path.

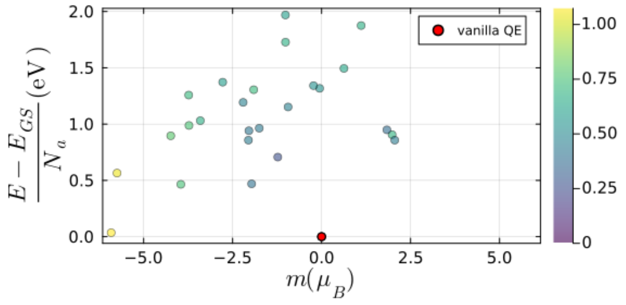


**Unique states:** Representation of 28 self-consistent unique states for monolayer $Re_2NiO_8$ identified using RomeoDFT (see Section S6).

**Lattice vectors:** Cartesian components (in [Å]) of the lattice vectors for $Re_2NiO_8$.

| | $x$ | $y$ | $z$ |
|---|---|---|---|
| $a_1$ | 11.0323 | 0.0000 | 0.0000 |
| $a_2$ | −2.7581 | 4.7771 | 0.0000 |
| $a_3$ | 0.0000 | 0.0000 | 26.9114 |

**Atomic positions:** Fractional coordinates, Hubbard $U$ (in eV) and magnetic moments (in $\mu_B$, computed from orbital occupations $m_o$ or integration spheres $m_i$) of each atom of monolayer $Re_2NiO_8$.

| atom | $x$ | $y$ | $z$ | $U$ | $m_o$ | $m_i$ |
|---|---|---|---|---|---|---|
| Re | 0.33 | 0.33 | 0.56 | 4.02 | 2.92 | −0.01 |
| Re | 0.17 | 0.67 | 0.44 | 4.02 | −2.92 | 0.01 |
| Ni | 0.0 | 0.0 | 0.50 | 5.77 | – | 1.72 |
| Re | 0.83 | 0.33 | 0.56 | 4.02 | – | 0.01 |
| Re | 0.67 | 0.67 | 0.44 | 4.02 | – | −0.01 |
| Ni | 0.50 | 0.0 | 0.50 | 5.77 | – | −1.72 |
| O | 0.38 | 0.67 | 0.54 | 0.0 | – | −0.01 |
| O | 0.17 | 0.08 | 0.54 | 0.0 | – | 0.01 |
| O | 0.46 | 0.25 | 0.54 | 0.0 | – | −0.02 |
| O | 0.33 | 0.33 | 0.63 | 0.0 | – | 0.00 |
| O | 0.12 | 0.33 | 0.46 | 0.0 | – | 0.01 |
| O | 0.33 | 0.92 | 0.46 | 0.0 | – | −0.01 |
| O | 0.04 | 0.75 | 0.46 | 0.0 | – | 0.02 |
| O | 0.17 | 0.67 | 0.37 | 0.0 | – | 0.00 |
| O | 0.88 | 0.67 | 0.54 | 0.0 | – | 0.01 |
| O | 0.67 | 0.08 | 0.54 | 0.0 | – | −0.01 |
| O | 0.96 | 0.25 | 0.54 | 0.0 | – | 0.02 |
| O | 0.83 | 0.33 | 0.63 | 0.0 | – | 0.00 |
| O | 0.62 | 0.33 | 0.46 | 0.0 | – | −0.01 |
| O | 0.83 | 0.92 | 0.46 | 0.0 | – | 0.01 |
| O | 0.54 | 0.75 | 0.46 | 0.0 | – | −0.02 |
| O | 0.67 | 0.67 | 0.37 | 0.0 | – | 0.00 |

## $RhN_4Cl_6$ (FM)

Band gap: 0.59 eV
Total magnetization: 1.6 $\mu_B/cell$
Absolute magnetization: 2.57 $\mu_B/cell$
MC2D entry: https://mc2d.materialscloud.org/#/details/mc2d-1972

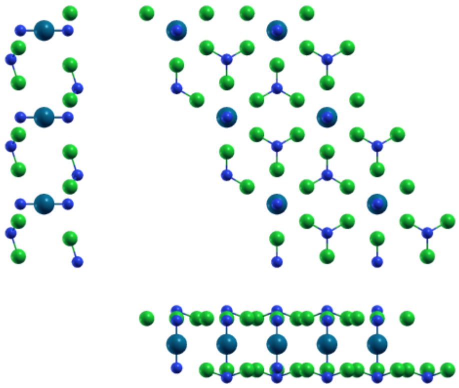

**Geometry:** Views of $RhN_4Cl_6$ as seen from the $x$ axis (left), the $y$ axis (bottom), and the $z$ axis (center).

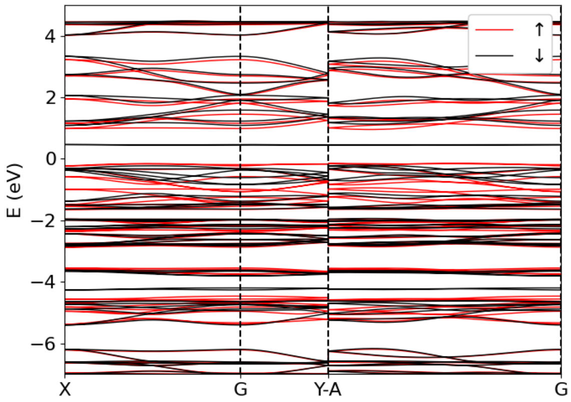


**Electronic bandstructure:** Spin-resolved energy bands of monolayer $RhN_4Cl_6$ along a high-symmetry path.

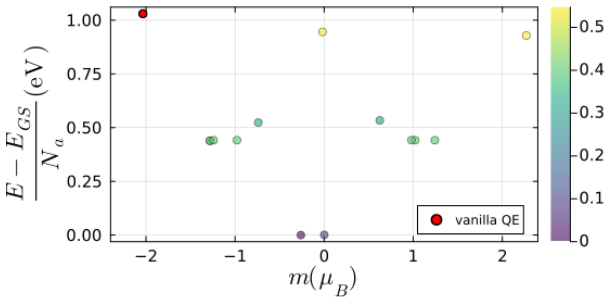


**Unique states:** Representation of 15 self-consistent unique states for monolayer $RhN_4Cl_6$ identified using RomeoDFT (see Section S6).

**Lattice vectors:** Cartesian components (in [Å]) of the lattice vectors for $RhN_4Cl_6$.

| | $x$ | $y$ | $z$ |
|---|---|---|---|
| $a_1$ | −7.3081 | −12.6579 | 0.0000 |
| $a_2$ | 7.3081 | 0.0000 | 0.0000 |
| $a_3$ | 0.0000 | 0.0000 | 19.3592 |

**Atomic positions:** Fractional coordinates, Hubbard $U$ (in eV) and magnetic moments (in $\mu_B$, computed from orbital occupations $m_o$ or integration spheres $m_i$) of each atom of monolayer $RhN_4Cl_6$.

| atom | $x$ | $y$ | $z$ | $U$ | $m_o$ | $m_i$ |
|---|---|---|---|---|---|---|
| Rh | 0.17 | 0.67 | 0.00 | 6.47 | −0.13 | −0.07 |
| Rh | 0.67 | 0.67 | 0.00 | 6.47 | −0.13 | −0.07 |
| Cl | 0.37 | 0.87 | −0.10 | 0.0 | – | 0.00 |
| Cl | 0.07 | 0.27 | −0.10 | 0.0 | – | 0.00 |
| Cl | 0.07 | 0.87 | −0.10 | 0.0 | – | 0.00 |
| N | 0.17 | 0.67 | −0.09 | 0.0 | – | 0.35 |
| N | 0.33 | 0.33 | 0.13 | 0.0 | – | −0.03 |
| N | 0.17 | 0.67 | 0.09 | 0.0 | – | 0.35 |
| Cl | 0.47 | 0.47 | 0.10 | 0.0 | – | 0.00 |
| Cl | 0.27 | 0.06 | 0.10 | 0.0 | – | 0.00 |
| Cl | 0.27 | 0.47 | 0.10 | 0.0 | – | 0.00 |
| N | 0.0 | 0.0 | −0.13 | 0.0 | – | −0.03 |
| Cl | 0.87 | 0.87 | −0.10 | 0.0 | – | 0.00 |
| Cl | 0.57 | 0.27 | −0.10 | 0.0 | – | 0.00 |
| Cl | 0.57 | 0.87 | −0.10 | 0.0 | – | 0.00 |
| N | 0.67 | 0.67 | −0.09 | 0.0 | – | 0.35 |
| N | 0.83 | 0.33 | 0.13 | 0.0 | – | −0.03 |
| N | 0.67 | 0.67 | 0.09 | 0.0 | – | 0.35 |
| Cl | 0.97 | 0.47 | 0.10 | 0.0 | – | 0.00 |
| Cl | 0.77 | 0.06 | 0.10 | 0.0 | – | 0.00 |
| Cl | 0.77 | 0.47 | 0.10 | 0.0 | – | 0.00 |
| N | 0.50 | 0.0 | −0.13 | 0.0 | – | −0.03 |

## $RuCl_3$ (FM)

Band gap: 1.98 eV
Total magnetization: $-2.0\ \mu_B/cell$
Absolute magnetization: $2.37\ \mu_B/cell$
MC2D entry: https://mc2d.materialscloud.org/#/details/mc2d-343

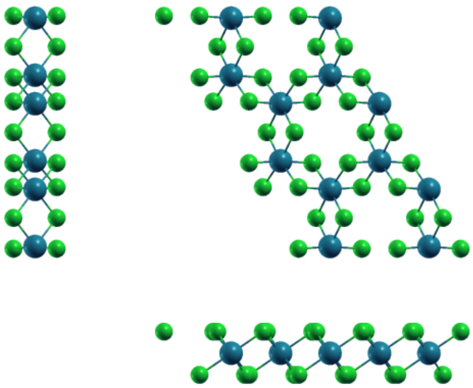

**Geometry:** Views of $RuCl_3$ as seen from the $x$ axis (left), the $y$ axis (bottom), and the $z$ axis (center).

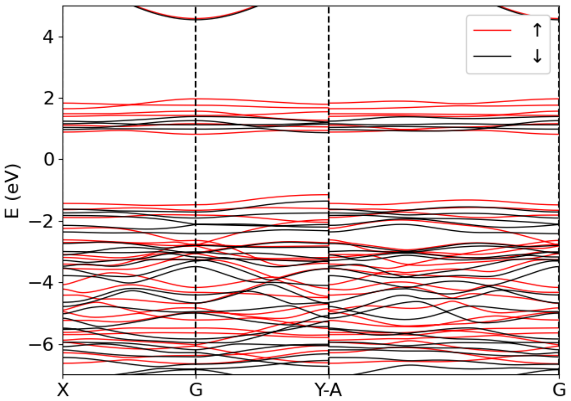


**Electronic bandstructure:** Spin-resolved energy bands of monolayer $RuCl_3$ along a high-symmetry path.

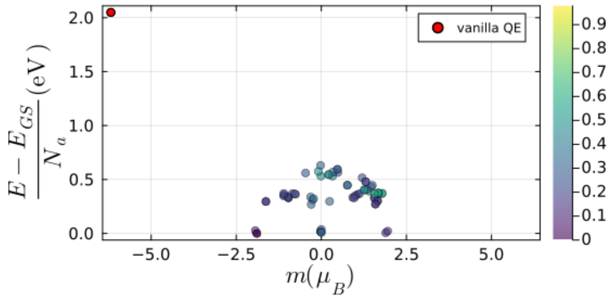


**Unique states:** Representation of 66 self-consistent unique states for monolayer $RuCl_3$ identified using RomeoDFT (see Section S6).

**Lattice vectors:** Cartesian components (in [Å]) of the lattice vectors for $RuCl_3$.

| | $x$ | $y$ | $z$ |
|---|---|---|---|
| $\boldsymbol{a_1}$ | 6.1029 | 0.0000 | 0.0000 |
| $\boldsymbol{a_2}$ | −3.0515 | 5.2876 | 0.0000 |
| $\boldsymbol{a_3}$ | 0.0000 | 0.0000 | 22.7474 |

**Atomic positions:** Fractional coordinates, Hubbard $U$ (in eV) and magnetic moments (in $\mu_B$, computed from orbital occupations $m_o$ or integration spheres $m_i$) of each atom of monolayer $RuCl_3$.

| atom | $x$ | $y$ | $z$ | $U$ | $m_o$ | $m_i$ |
|---|---|---|---|---|---|---|
| Ru | 0.56 | 0.11 | 0.50 | 4.70 | −0.95 | −0.83 |
| Ru | 0.89 | 0.78 | 0.50 | 4.70 | −0.95 | −0.83 |
| Cl | 0.22 | 0.09 | 0.56 | 0.0 | – | 0.01 |
| Cl | 0.86 | 0.09 | 0.44 | 0.0 | – | 0.01 |
| Cl | 0.58 | 0.80 | 0.56 | 0.0 | – | 0.01 |
| Cl | 0.22 | 0.80 | 0.44 | 0.0 | – | 0.01 |
| Cl | 0.86 | 0.45 | 0.56 | 0.0 | – | 0.01 |
| Cl | 0.58 | 0.45 | 0.44 | 0.0 | – | 0.01 |

## $RuF_4$ (AM)

Band gap: 1.87 eV
Total magnetization: −0.0 $\mu_B/cell$
Absolute magnetization: 4.21 $\mu_B/cell$
MC2D entry: https://mc2d.materialscloud.org/#/details/mc2d-1509

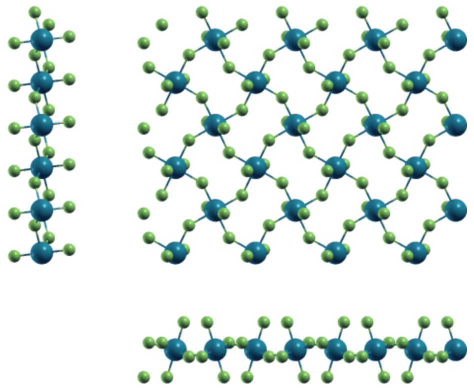

**Geometry:** Views of $RuF_4$ as seen from the $x$ axis (left), the $y$ axis (bottom), and the $z$ axis (center).

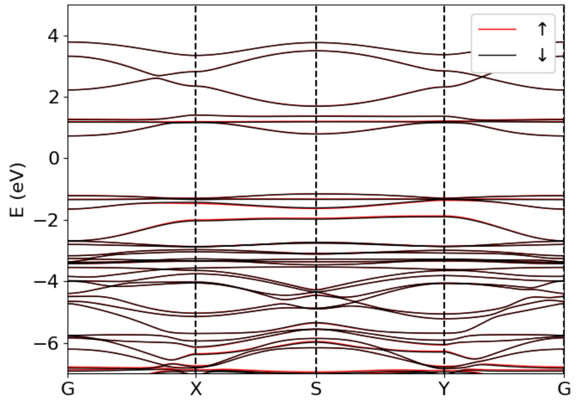


**Electronic bandstructure:** Spin-resolved energy bands of monolayer $RuF_4$ along a high-symmetry path.

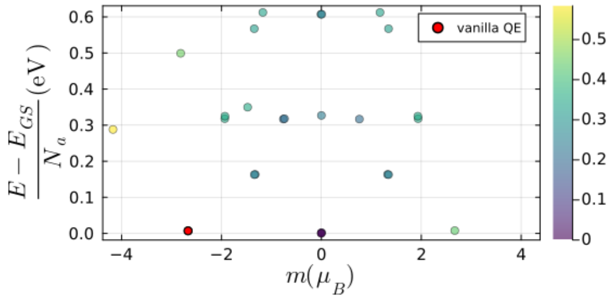


**Unique states:** Representation of 26 self-consistent unique states for monolayer $RuF_4$ identified using RomeoDFT (see Section S6).

**Lattice vectors:** Cartesian components (in [Å]) of the lattice vectors for $RuF_4$.

| | $x$ | $y$ | $z$ |
|---|---|---|---|
| $\boldsymbol{a_1}$ | 5.1349 | 0.0000 | 0.0000 |
| $\boldsymbol{a_2}$ | 0.0000 | 5.4653 | 0.0000 |
| $\boldsymbol{a_3}$ | 0.0000 | 0.0000 | 23.4375 |

**Atomic positions:** Fractional coordinates, Hubbard $U$ (in eV) and magnetic moments (in $\mu_B$, computed from orbital occupations $m_o$ or integration spheres $m_i$) of each atom of monolayer $RuF_4$.

| atom | $x$ | $y$ | $z$ | $U$ | $m_o$ | $m_i$ |
|---|---|---|---|---|---|---|
| Ru | 0.0 | 0.54 | 0.50 | 3.52 | 1.34 | 1.05 |
| Ru | 0.50 | 0.04 | 0.50 | 3.66 | −1.35 | −1.05 |
| F | 0.60 | 0.08 | 0.42 | 0.0 | – | −0.20 |
| F | 0.90 | 0.58 | 0.42 | 0.0 | – | 0.20 |
| F | 0.40 | 1.00 | 0.58 | 0.0 | – | −0.20 |
| F | 0.10 | 0.50 | 0.58 | 0.0 | – | 0.20 |
| F | 0.33 | 0.72 | 0.48 | 0.0 | – | 0.01 |
| F | 0.17 | 0.22 | 0.48 | 0.0 | – | 0.00 |
| F | 0.67 | 0.36 | 0.52 | 0.0 | – | 0.01 |
| F | 0.83 | 0.86 | 0.52 | 0.0 | – | 0.00 |

## ScCl (FM)

Band gap: 0.0 eV
Total magnetization: $-1.51\ \mu_B/cell$
Absolute magnetization: $1.52\ \mu_B/cell$
MC2D entry: https://mc2d.materialscloud.org/#/details/mc2d-123

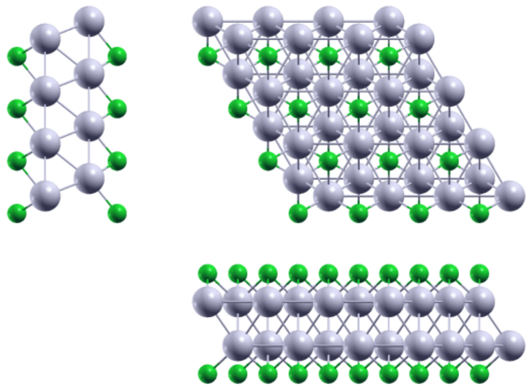

**Geometry:** Views of ScCl as seen from the $x$ axis (left), the $y$ axis (bottom), and the $z$ axis (center).

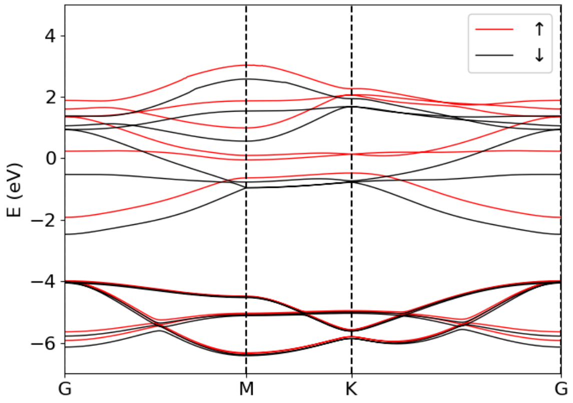


**Electronic bandstructure:** Spin-resolved energy bands of monolayer ScCl along a high-symmetry path.

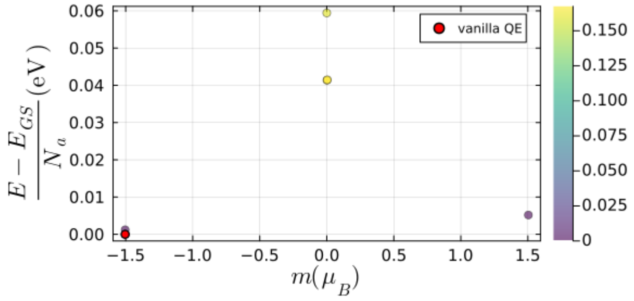


**Unique states:** Representation of 6 self-consistent unique states for monolayer ScCl identified using RomeoDFT (see Section S6).

**Lattice vectors:** Cartesian components (in [Å]) of the lattice vectors for ScCl.

| | $x$ | $y$ | $z$ |
|---|---|---|---|
| $\boldsymbol{a_1}$ | 3.4955 | 0.0000 | 0.0000 |
| $\boldsymbol{a_2}$ | −1.7478 | 3.0272 | 0.0000 |
| $\boldsymbol{a_3}$ | 0.0000 | 0.0000 | 25.8028 |

**Atomic positions:** Fractional coordinates, Hubbard $U$ (in eV) and magnetic moments (in $\mu_B$, computed from orbital occupations $m_o$ or integration spheres $m_i$) of each atom of monolayer ScCl.

| atom | $x$ | $y$ | $z$ | $U$ | $m_o$ | $m_i$ |
|---|---|---|---|---|---|---|
| Sc | 0.67 | 0.33 | 0.55 | 0.00 | −0.75 | −0.35 |
| Sc | 0.33 | 0.67 | 0.45 | 0.00 | −0.75 | −0.35 |
| Cl | 1.00 | 1.00 | 0.61 | 0.0 | – | −0.01 |
| Cl | 1.00 | 1.00 | 0.39 | 0.0 | – | −0.01 |

## $SiN_2F_6$ (AFM)

Band gap: 0.00 eV
Total magnetization: 0.0 $\mu_B/cell$
Absolute magnetization: 4.36 $\mu_B/cell$
MC2D entry: https://mc2d.materialscloud.org/#/details/mc2d-569

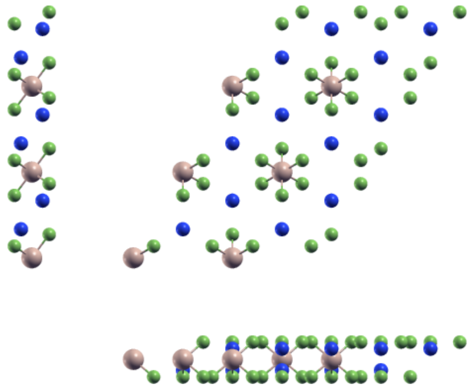

**Geometry:** Views of $SiN_2F_6$ as seen from the $x$ axis (left), the $y$ axis (bottom), and the $z$ axis (center).

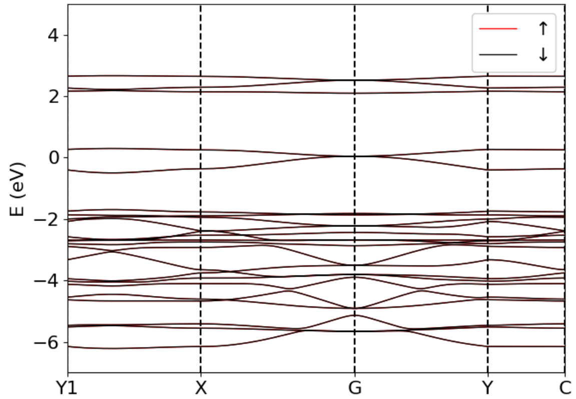


**Electronic bandstructure:** Spin-resolved energy bands of monolayer $SiN_2F_6$ along a high-symmetry path.

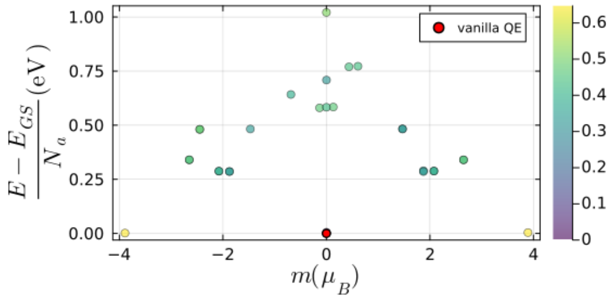


**Unique states:** Representation of 30 self-consistent unique states for monolayer $SiN_2F_6$ identified using RomeoDFT (see Section S6).

**Lattice vectors:** Cartesian components (in [Å]) of the lattice vectors for $SiN_2F_6$.

| | $x$ | $y$ | $z$ |
|---|---|---|---|
| $\boldsymbol{a_1}$ | 5.8482 | 0.0000 | 0.0000 |
| $\boldsymbol{a_2}$ | 2.9241 | 5.0647 | 0.0000 |
| $\boldsymbol{a_3}$ | 0.0000 | 0.0000 | 22.0894 |

**Atomic positions:** Fractional coordinates, Hubbard $U$ (in eV) and magnetic moments (in $\mu_B$, computed from orbital occupations $m_o$ or integration spheres $m_i$) of each atom of monolayer $SiN_2F_6$.

| atom | $x$ | $y$ | $z$ | $U$ | $m_o$ | $m_i$ |
|---|---|---|---|---|---|---|
| N | 0.67 | 0.67 | 0.47 | 0.00 | −1.95 | −1.64 |
| N | 0.33 | 0.33 | 0.53 | 0.00 | 1.95 | 1.64 |
| Si | 0.0 | 0.0 | 0.50 | 0.0 | – | 0.0 |
| F | 0.73 | 0.14 | 0.55 | 0.0 | – | −0.02 |
| F | 0.14 | 0.14 | 0.55 | 0.0 | – | −0.02 |
| F | 0.14 | 0.73 | 0.55 | 0.0 | – | −0.02 |
| F | 0.86 | 0.27 | 0.45 | 0.0 | – | 0.02 |
| F | 0.27 | 0.86 | 0.45 | 0.0 | – | 0.02 |
| F | 0.86 | 0.86 | 0.45 | 0.0 | – | 0.02 |

# $SmI_3$ (FM)

Band gap: 0.09 eV
Total magnetization: $-10.16\ \mu_B/cell$
Absolute magnetization: $10.86\ \mu_B/cell$
MC2D entry: https://mc2d.materialscloud.org/#/details/mc2d-2524

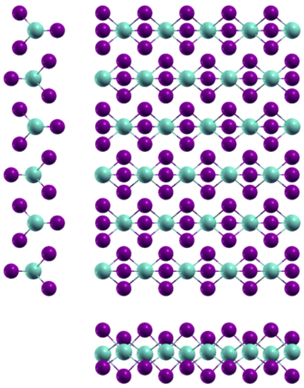

**Geometry:** Views of $SmI_3$ as seen from the $x$ axis (left), the $y$ axis (bottom), and the $z$ axis (center).

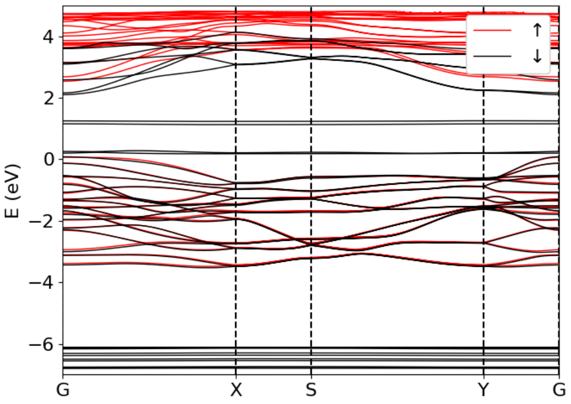


**Electronic bandstructure:** Spin-resolved energy bands of monolayer $SmI_3$ along a high-symmetry path.

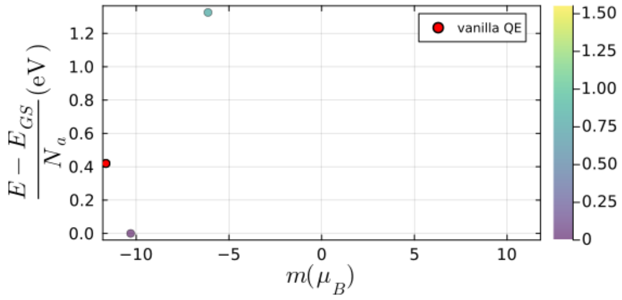


**Unique states:** Representation of 4 self-consistent unique states for monolayer $SmI_3$ identified using RomeoDFT (see Section S6).

**Lattice vectors:** Cartesian components (in [Å]) of the lattice vectors for $SmI_3$.

| | $x$ | $y$ | $z$ |
|---|---|---|---|
| $\boldsymbol{a_1}$ | 4.3490 | 0.0000 | 0.0000 |
| $\boldsymbol{a_2}$ | 0.0000 | 9.9743 | 0.0000 |
| $\boldsymbol{a_3}$ | 0.0000 | 0.0000 | 21.3906 |

**Atomic positions:** Fractional coordinates, Hubbard $U$ (in eV) and magnetic moments (in $\mu_B$, computed from orbital occupations $m_o$ or integration spheres $m_i$) of each atom of monolayer $SmI_3$.

| atom | $x$ | $y$ | $z$ | $U$ | $m_o$ | $m_i$ |
|---|---|---|---|---|---|---|
| Sm | −0.25 | −0.25 | −0.01 | 6.96 | −5.15 | −5.16 |
| Sm | 0.25 | −0.75 | 0.01 | 6.96 | −5.15 | −5.16 |
| I | 0.25 | −0.07 | 0.07 | 0.0 | – | 0.04 |
| I | 0.25 | −0.43 | 0.07 | 0.0 | – | 0.04 |
| I | −0.25 | −0.57 | −0.07 | 0.0 | – | 0.04 |
| I | −0.25 | −0.93 | −0.07 | 0.0 | – | 0.04 |
| I | 0.25 | −0.25 | −0.11 | 0.0 | – | 0.05 |
| I | −0.25 | −0.75 | 0.11 | 0.0 | – | 0.05 |

## SmOBr (AFM)

Band gap: 1.80 eV
Total magnetization: 0.0 $\mu_B/cell$
Absolute magnetization: 10.2 $\mu_B/cell$
MC2D entry: https://mc2d.materialscloud.org/#/details/mc2d-65

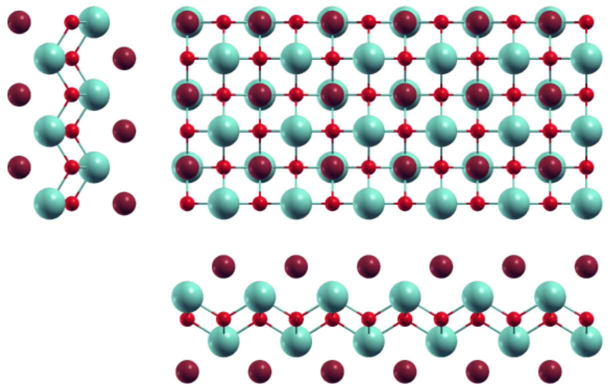

**Geometry:** Views of SmOBr as seen from the $x$ axis (left), the $y$ axis (bottom), and the $z$ axis (center).

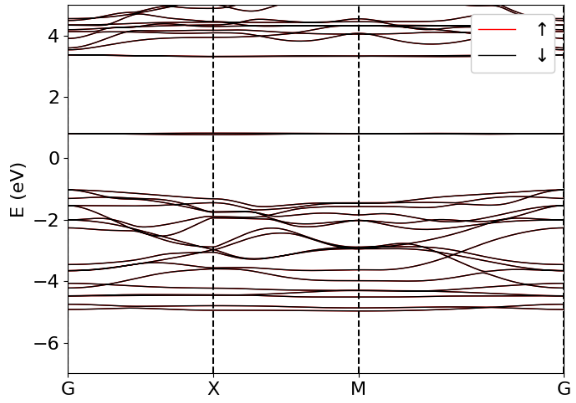


**Electronic bandstructure:** Spin-resolved energy bands of monolayer SmOBr along a high-symmetry path.

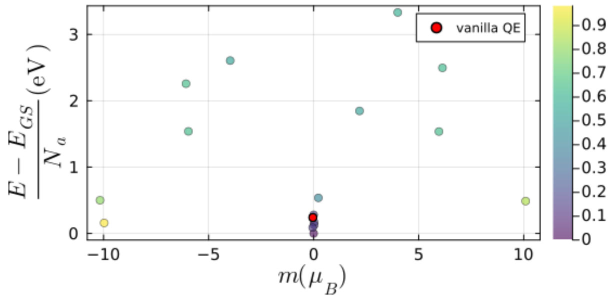


**Unique states:** Representation of 18 self-consistent unique states for monolayer SmOBr identified using RomeoDFT (see Section S6).

**Lattice vectors:** Cartesian components (in [Å]) of the lattice vectors for SmOBr.

| | $x$ | $y$ | $z$ |
|---|---|---|---|
| $a_1$ | 3.9395 | 0.0000 | 0.0000 |
| $a_2$ | 0.0000 | 3.9395 | 0.0000 |
| $a_3$ | 0.0000 | 0.0000 | 25.3988 |

**Atomic positions:** Fractional coordinates, Hubbard $U$ (in eV) and magnetic moments (in $\mu_B$, computed from orbital occupations $m_o$ or integration spheres $m_i$) of each atom of monolayer SmOBr.

| atom | $x$ | $y$ | $z$ | $U$ | $m_o$ | $m_i$ |
|---|---|---|---|---|---|---|
| Sm | 0.50 | 0.0 | 0.55 | 4.66 | 4.97 | 4.87 |
| Sm | 0.0 | 0.50 | 0.45 | 4.66 | −4.97 | −4.87 |
| Br | 0.0 | 0.50 | 0.61 | 0.0 | – | −0.01 |
| Br | 0.50 | 0.0 | 0.39 | 0.0 | – | 0.01 |
| O | 0.0 | 0.0 | 0.50 | 0.0 | – | 0.0 |
| O | 0.50 | 0.50 | 0.50 | 0.0 | – | 0.0 |

## SmSI (AFM)

Band gap: 1.63 eV
Total magnetization: 0.0 $\mu_B/cell$
Absolute magnetization: 10.38 $\mu_B/cell$
MC2D entry: https://mc2d.materialscloud.org/#/details/mc2d-197

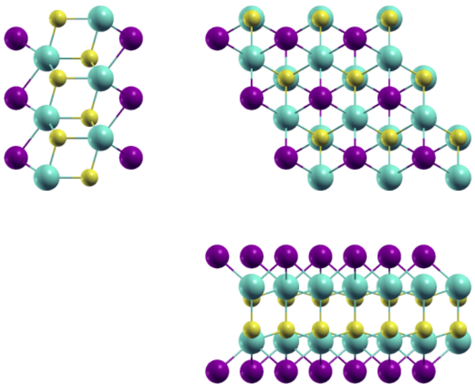

**Geometry:** Views of SmSI as seen from the $x$ axis (left), the $y$ axis (bottom), and the $z$ axis (center).

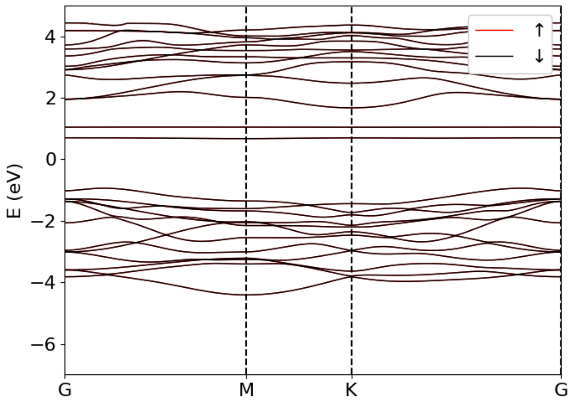


**Electronic bandstructure:** Spin-resolved energy bands of monolayer SmSI along a high-symmetry path.

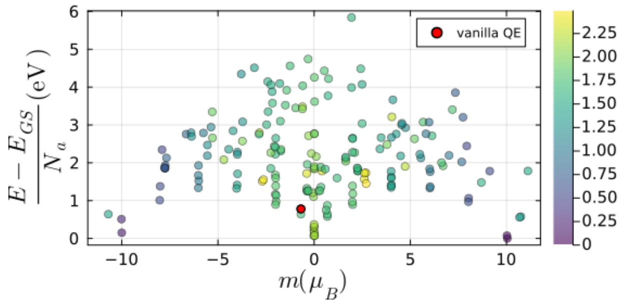


**Unique states:** Representation of 184 self-consistent unique states for monolayer SmSI identified using RomeoDFT (see Section S6).

**Lattice vectors:** Cartesian components (in [Å]) of the lattice vectors for SmSI.

| | $x$ | $y$ | $z$ |
|---|---|---|---|
| $\boldsymbol{a_1}$ | 2.2780 | −3.9455 | 0.0000 |
| $\boldsymbol{a_2}$ | 2.2780 | 3.9455 | 0.0000 |
| $\boldsymbol{a_3}$ | 0.0000 | 0.0000 | 27.2237 |

**Atomic positions:** Fractional coordinates, Hubbard $U$ (in eV) and magnetic moments (in $\mu_B$, computed from orbital occupations $m_o$ or integration spheres $m_i$) of each atom of monolayer SmSI.

| atom | $x$ | $y$ | $z$ | $U$ | $m_o$ | $m_i$ |
|---|---|---|---|---|---|---|
| Sm | 0.33 | 0.17 | 0.07 | 8.16 | 5.02 | 4.99 |
| Sm | 0.67 | 0.83 | −0.07 | 8.16 | −5.02 | −4.99 |
| I | 0.0 | 0.50 | 0.14 | 0.0 | – | −0.03 |
| S | 0.33 | 0.17 | −0.04 | 0.0 | – | 0.03 |
| S | 0.67 | 0.83 | 0.04 | 0.0 | – | −0.03 |
| I | 0.0 | 0.50 | −0.14 | 0.0 | – | 0.03 |

## $Sr_2CoO_2Br_2$ (AFM)

Band gap: 1.76 eV
Total magnetization: $-0.0\ \mu_B/cell$
Absolute magnetization: $5.79\ \mu_B/cell$
MC2D entry: https://mc2d.materialscloud.org/#/details/mc2d-495

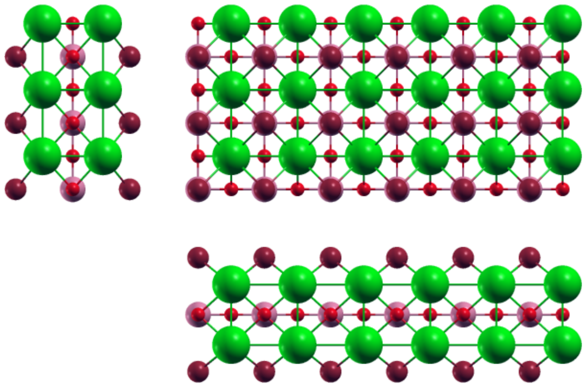

**Geometry:** Views of $Sr_2CoO_2Br_2$ as seen from the $x$ axis (left), the $y$ axis (bottom), and the $z$ axis (center).

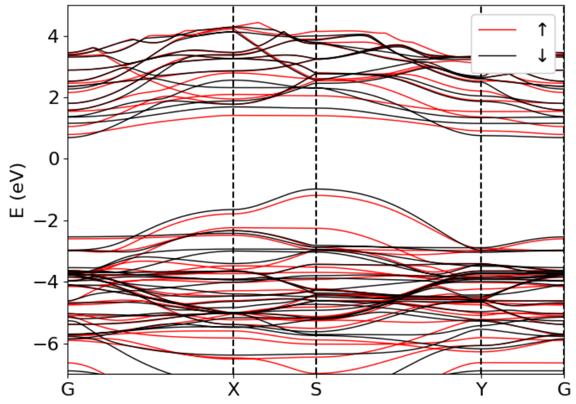


**Electronic bandstructure:** Spin-resolved energy bands of monolayer $Sr_2CoO_2Br_2$ along a high-symmetry path.

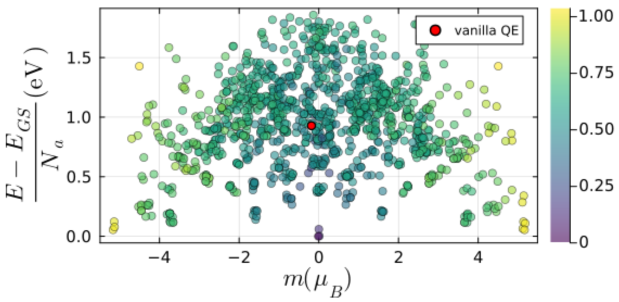


**Unique states:** Representation of 1169 self-consistent unique states for monolayer $Sr_2CoO_2Br_2$ identified using RomeoDFT (see Section S6).

**Lattice vectors:** Cartesian components (in [Å]) of the lattice vectors for $Sr_2CoO_2Br_2$.

| | $x$ | $y$ | $z$ |
|---|---|---|---|
| $\boldsymbol{a_1}$ | 7.8022 | 0.0000 | 0.0000 |
| $\boldsymbol{a_2}$ | 0.0000 | 3.9011 | 0.0000 |
| $\boldsymbol{a_3}$ | 0.0000 | 0.0000 | 26.6290 |

**Atomic positions:** Fractional coordinates, Hubbard $U$ (in eV) and magnetic moments (in $\mu_B$, computed from orbital occupations $m_o$ or integration spheres $m_i$) of each atom of monolayer $Sr_2CoO_2Br_2$.

| atom | $x$ | $y$ | $z$ | $U$ | $m_o$ | $m_i$ |
|---|---|---|---|---|---|---|
| Co | 0.0 | 0.0 | 0.50 | 6.28 | −2.52 | −2.45 |
| Co | 0.50 | 0.0 | 0.50 | 6.28 | 2.53 | 2.48 |
| Br | 0.0 | 0.0 | 0.37 | 0.0 | – | −0.01 |
| Sr | 0.25 | 0.50 | 0.57 | 0.0 | – | 0.00 |
| Sr | 0.25 | 0.50 | 0.43 | 0.0 | – | 0.00 |
| Br | 0.0 | 0.0 | 0.63 | 0.0 | – | −0.01 |
| O | 0.25 | 0.0 | 0.50 | 0.0 | – | 0.01 |
| O | 0.0 | 0.50 | 0.50 | 0.0 | – | −0.07 |
| Br | 0.50 | 0.0 | 0.37 | 0.0 | – | 0.00 |
| Sr | 0.75 | 0.50 | 0.57 | 0.0 | – | 0.00 |
| Sr | 0.75 | 0.50 | 0.43 | 0.0 | – | 0.00 |
| Br | 0.50 | 0.0 | 0.63 | 0.0 | – | 0.00 |
| O | 0.75 | 0.0 | 0.50 | 0.0 | – | 0.01 |
| O | 0.50 | 0.50 | 0.50 | 0.0 | – | 0.12 |

## $SrO_{10}$ (FM)

Band gap: 0.0 eV
Total magnetization: $-4.34\ \mu_B/cell$
Absolute magnetization: $5.12\ \mu_B/cell$
MC2D entry: https://mc2d.materialscloud.org/#/details/mc2d-1475

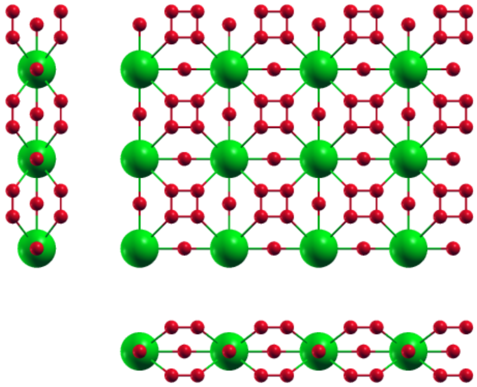

**Geometry:** Views of $SrO_{10}$ as seen from the $x$ axis (left), the $y$ axis (bottom), and the $z$ axis (center).

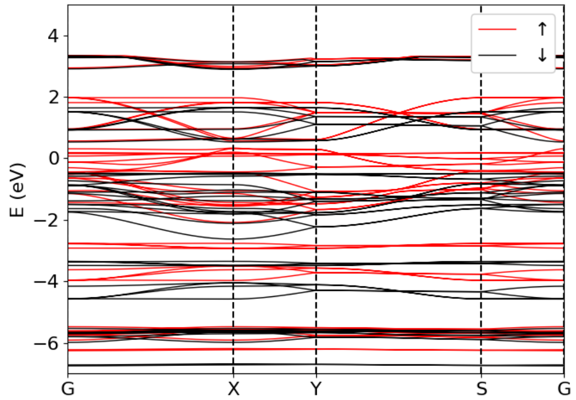


**Electronic bandstructure:** Spin-resolved energy bands of monolayer $SrO_{10}$ along a high-symmetry path.

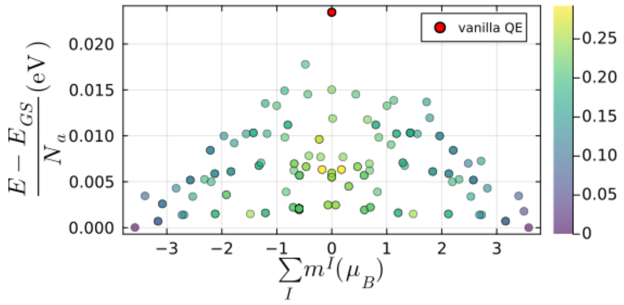


**Unique states:** Representation of 139 self-consistent unique states for monolayer $SrO_{10}$ identified using RomeoDFT (see Section S6).

**Lattice vectors:** Cartesian components (in [Å]) of the lattice vectors for $SrO_{10}$.

| | $x$ | $y$ | $z$ |
|---|---|---|---|
| $a_1$ | 10.4476 | 0.0000 | 0.0000 |
| $a_2$ | 0.0000 | 5.2238 | 0.0000 |
| $a_3$ | 0.0000 | 0.0000 | 22.7222 |

**Atomic positions:** Fractional coordinates, Hubbard $U$ (in eV) and magnetic moments (in $\mu_B$, computed from orbital occupations $m_o$ or integration spheres $m_i$) of each atom of monolayer $SrO_{10}$.

| atom | $x$ | $y$ | $z$ | $U$ | $m_o$ | $m_i$ |
|---|---|---|---|---|---|---|
| O | 0.18 | 0.36 | 0.56 | 0.00 | −0.09 | −0.11 |
| O | 0.32 | 0.36 | 0.56 | 0.00 | −0.09 | −0.11 |
| O | 0.18 | 0.64 | 0.56 | 0.00 | −0.09 | −0.11 |
| O | 0.32 | 0.64 | 0.56 | 0.00 | −0.09 | −0.11 |
| Sr | 0.0 | 0.0 | 0.50 | 0.00 | 0.00 | 0.00 |
| O | 0.18 | 0.36 | 0.44 | 0.00 | −0.09 | −0.11 |
| O | 0.32 | 0.64 | 0.44 | 0.00 | −0.09 | −0.11 |
| O | 0.18 | 0.64 | 0.44 | 0.00 | −0.09 | −0.11 |
| O | 0.32 | 0.36 | 0.44 | 0.00 | −0.09 | −0.11 |
| O | 0.25 | 0.0 | 0.50 | 0.00 | −0.54 | −0.40 |
| O | 0.0 | 0.50 | 0.50 | 0.00 | −0.53 | −0.40 |
| O | 0.68 | 0.36 | 0.56 | 0.00 | −0.09 | −0.11 |
| O | 0.82 | 0.36 | 0.56 | 0.00 | −0.09 | −0.11 |
| O | 0.68 | 0.64 | 0.56 | 0.00 | −0.09 | −0.11 |
| O | 0.82 | 0.64 | 0.56 | 0.00 | −0.09 | −0.11 |
| Sr | 0.50 | 0.0 | 0.50 | 0.00 | 0.00 | 0.00 |
| O | 0.68 | 0.36 | 0.44 | 0.00 | −0.09 | −0.11 |
| O | 0.82 | 0.64 | 0.44 | 0.00 | −0.09 | −0.11 |
| O | 0.68 | 0.64 | 0.44 | 0.00 | −0.09 | −0.11 |
| O | 0.82 | 0.36 | 0.44 | 0.00 | −0.09 | −0.11 |
| O | 0.75 | 0.0 | 0.50 | 0.00 | −0.54 | −0.40 |
| O | 0.50 | 0.50 | 0.50 | 0.00 | −0.54 | −0.40 |

## $TbBr_3$ (C2/m) (AFM)

Band gap: 2.23 eV
Total magnetization: $-0.0\ \mu_B/cell$
Absolute magnetization: 12.19 $\mu_B/cell$
MC2D entry: https://mc2d.materialscloud.org/#/details/mc2d-2007

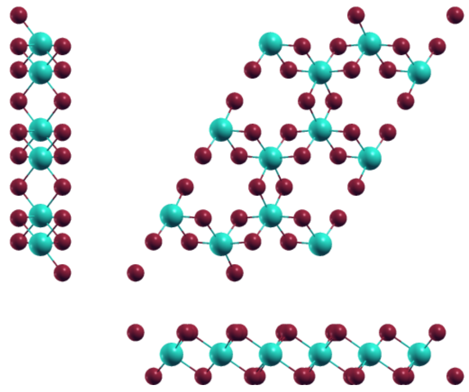

**Geometry:** Views of $TbBr_3$ (C2/m) as seen from the $x$ axis (left), the $y$ axis (bottom), and the $z$ axis (center).

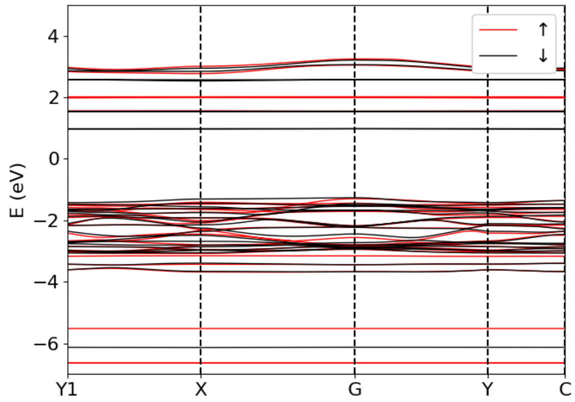


**Electronic bandstructure:** Spin-resolved energy bands of monolayer $TbBr_3$ (C2/m) along a high-symmetry path.

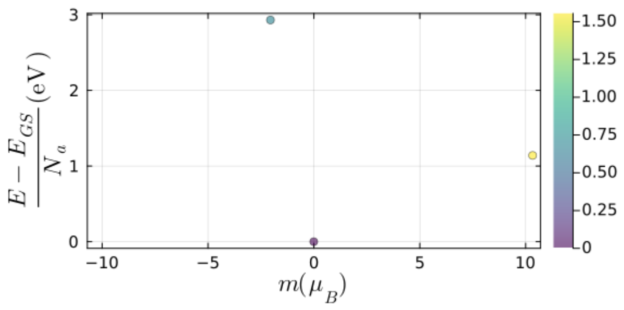


**Unique states:** Representation of 4 self-consistent unique states for monolayer $TbBr_3$ (C2/m) identified using RomeoDFT (see Section S6).

**Lattice vectors:** Cartesian components (in [Å]) of the lattice vectors for $TbBr_3$ (C2/m).

| | $x$ | $y$ | $z$ |
|---|---|---|---|
| $a_1$ | 3.6497 | 6.3173 | 0.0000 |
| $a_2$ | −3.6497 | 6.3173 | 0.0000 |
| $a_3$ | 0.0000 | 0.0000 | 18.4068 |

**Atomic positions:** Fractional coordinates, Hubbard $U$ (in eV) and magnetic moments (in $\mu_B$, computed from orbital occupations $m_o$ or integration spheres $m_i$) of each atom of monolayer $TbBr_3$ (C2/m).

| atom | $x$ | $y$ | $z$ | $U$ | $m_o$ | $m_i$ |
|---|---|---|---|---|---|---|
| Tb | −0.17 | 0.33 | 0.0 | 4.10 | 5.96 | 5.98 |
| Tb | 0.17 | 0.67 | 0.0 | 4.10 | −5.97 | −5.99 |
| Br | −0.14 | 0.00 | 0.09 | 0.0 | – | 0.00 |
| Br | 0.50 | 0.64 | 0.09 | 0.0 | – | 0.00 |
| Br | 0.50 | 0.36 | −0.09 | 0.0 | – | 0.00 |
| Br | 0.14 | 0.00 | −0.09 | 0.0 | – | 0.00 |
| Br | 0.14 | 0.36 | 0.09 | 0.0 | – | 0.00 |
| Br | −0.14 | 0.64 | −0.09 | 0.0 | – | 0.00 |

## $TbBr_3$ (Pmmn) (FM)

Band gap: 1.95 eV
Total magnetization: 12.0 $\mu_B/cell$
Absolute magnetization: 12.25 $\mu_B/cell$
MC2D entry: https://mc2d.materialscloud.org/#/details/mc2d-1892

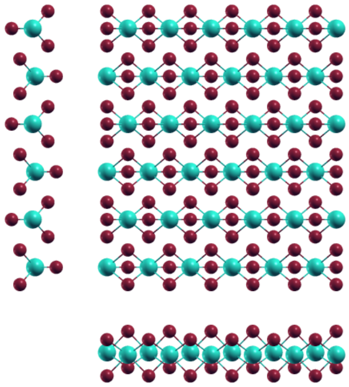

**Geometry:** Views of $TbBr_3$ (Pmmn) as seen from the $x$ axis (left), the $y$ axis (bottom), and the $z$ axis (center).

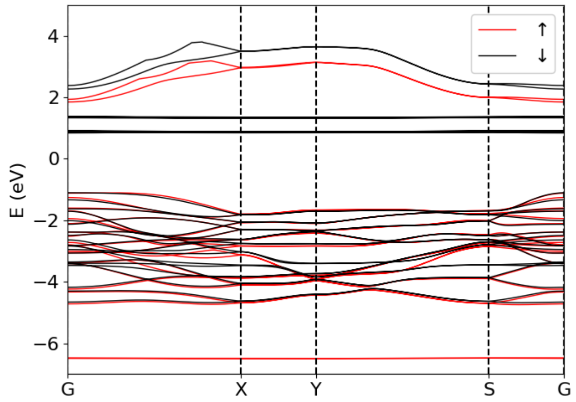


**Electronic bandstructure:** Spin-resolved energy bands of monolayer $TbBr_3$ (Pmmn) along a high-symmetry path.

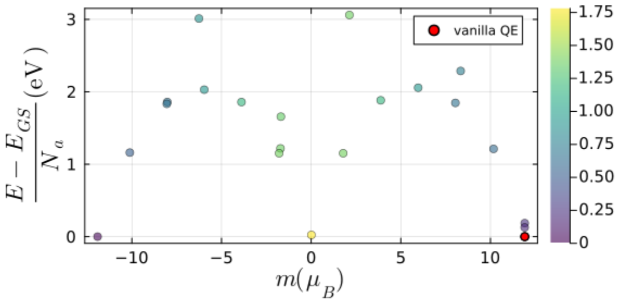


**Unique states:** Representation of 22 self-consistent unique states for monolayer $TbBr_3$ (Pmmn) identified using RomeoDFT (see Section S6).

**Lattice vectors:** Cartesian components (in [Å]) of the lattice vectors for $TbBr_3$ (Pmmn).

| | $x$ | $y$ | $z$ |
|---|---|---|---|
| $\boldsymbol{a_1}$ | 3.9919 | 0.0000 | 0.0000 |
| $\boldsymbol{a_2}$ | 0.0000 | 9.1676 | 0.0000 |
| $\boldsymbol{a_3}$ | 0.0000 | 0.0000 | 20.4351 |

**Atomic positions:** Fractional coordinates, Hubbard $U$ (in eV) and magnetic moments (in $\mu_B$, computed from orbital occupations $m_o$ or integration spheres $m_i$) of each atom of monolayer $TbBr_3$ (Pmmn).

| atom | $x$ | $y$ | $z$ | $U$ | $m_o$ | $m_i$ |
|---|---|---|---|---|---|---|
| Tb | 0.25 | −0.75 | −0.01 | 3.80 | 5.95 | 5.99 |
| Tb | −0.25 | −0.25 | 0.01 | 3.80 | 5.96 | 5.99 |
| Br | −0.25 | −0.93 | 0.07 | 0.0 | – | −0.02 |
| Br | −0.25 | −0.57 | 0.07 | 0.0 | – | −0.02 |
| Br | 0.25 | −0.43 | −0.07 | 0.0 | – | −0.02 |
| Br | 0.25 | −0.07 | −0.07 | 0.0 | – | −0.02 |
| Br | −0.25 | −0.75 | −0.10 | 0.0 | – | −0.01 |
| Br | 0.25 | −0.25 | 0.10 | 0.0 | – | −0.01 |

# $TbCl_3$ (FM)

Band gap: 2.71 eV
Total magnetization: 12.0 $\mu_B/cell$
Absolute magnetization: 12.19 $\mu_B/cell$

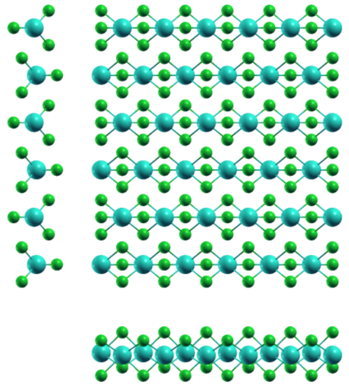

**Geometry:** Views of $TbCl_3$ as seen from the $x$ axis (left), the $y$ axis (bottom), and the $z$ axis (center).

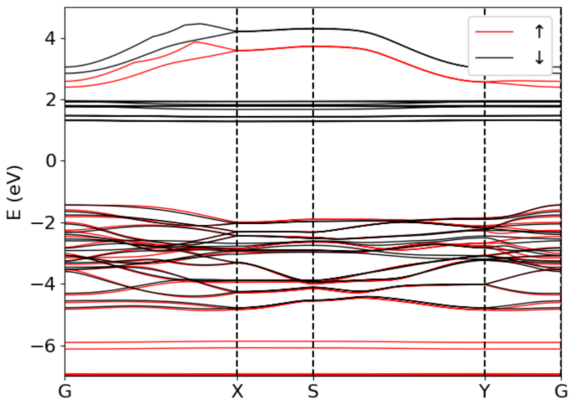


**Electronic bandstructure:** Spin-resolved energy bands of monolayer $TbCl_3$ along a high-symmetry path.

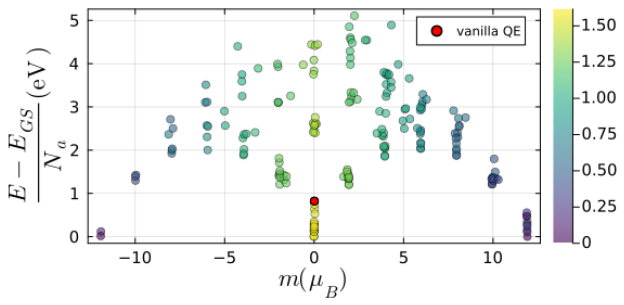


**Unique states:** Representation of 194 self-consistent unique states for monolayer $TbCl_3$ identified using RomeoDFT (see Section S6).

**Lattice vectors:** Cartesian components (in [Å]) of the lattice vectors for $TbCl_3$.

| | $x$ | $y$ | $z$ |
|---|---|---|---|
| $\boldsymbol{a_1}$ | 3.8254 | 0.0000 | 0.0000 |
| $\boldsymbol{a_2}$ | 0.0000 | 8.6310 | 0.0000 |
| $\boldsymbol{a_3}$ | 0.0000 | 0.0000 | 23.8706 |

**Atomic positions:** Fractional coordinates, Hubbard $U$ (in eV) and magnetic moments (in $\mu_B$, computed from orbital occupations $m_o$ or integration spheres $m_i$) of each atom of monolayer $TbCl_3$.

| atom | $x$ | $y$ | $z$ | $U$ | $m_o$ | $m_i$ |
|---|---|---|---|---|---|---|
| Tb | 0.50 | 0.75 | 0.50 | 3.62 | 5.95 | 5.96 |
| Tb | 0.0 | 0.25 | 0.50 | 3.98 | 5.96 | 5.97 |
| Cl | 0.50 | 0.07 | 0.55 | 0.0 | – | −0.01 |
| Cl | 0.50 | 0.43 | 0.55 | 0.0 | – | −0.01 |
| Cl | 0.50 | 0.25 | 0.42 | 0.0 | – | −0.01 |
| Cl | 0.0 | 0.93 | 0.45 | 0.0 | – | −0.01 |
| Cl | 0.0 | 0.57 | 0.45 | 0.0 | – | −0.01 |
| Cl | 0.0 | 0.75 | 0.58 | 0.0 | – | −0.01 |

## $TbH_2Br$ (AFM)

Band gap: 1.40 eV
Total magnetization: 0.0 $\mu_B/cell$
Absolute magnetization: 12.23 $\mu_B/cell$
MC2D entry: https://mc2d.materialscloud.org/#/details/mc2d-2480

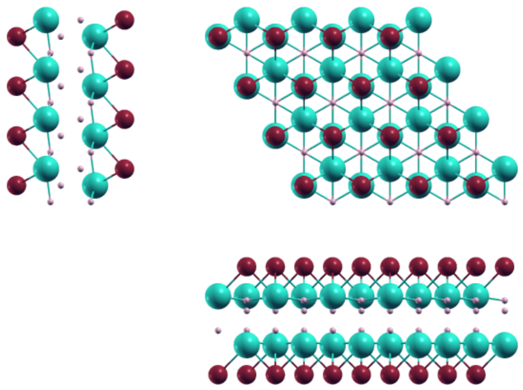

**Geometry:** Views of $TbH_2Br$ as seen from the $x$ axis (left), the $y$ axis (bottom), and the $z$ axis (center).

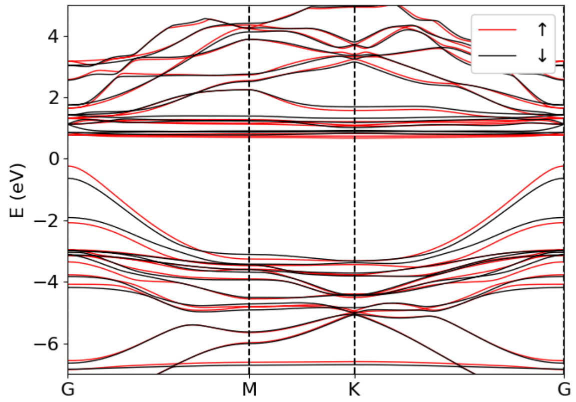


**Electronic bandstructure:** Spin-resolved energy bands of monolayer $TbH_2Br$ along a high-symmetry path.

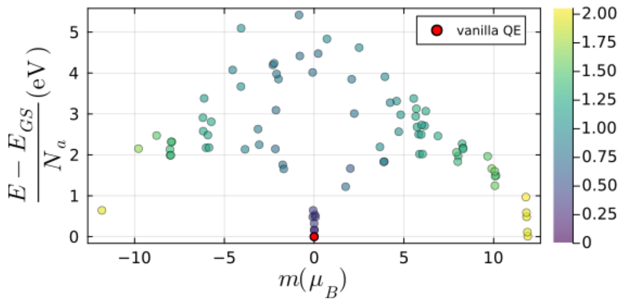


**Unique states:** Representation of 85 self-consistent unique states for monolayer $TbH_2Br$ identified using RomeoDFT (see Section S6).

**Lattice vectors:** Cartesian components (in [Å]) of the lattice vectors for $TbH_2Br$.

| | $x$ | $y$ | $z$ |
|---|---|---|---|
| $a_1$ | 1.8892 | −3.2723 | 0.0000 |
| $a_2$ | 1.8892 | 3.2723 | 0.0000 |
| $a_3$ | 0.0000 | 0.0000 | 26.1027 |

**Atomic positions:** Fractional coordinates, Hubbard $U$ (in eV) and magnetic moments (in $\mu_B$, computed from orbital occupations $m_o$ or integration spheres $m_i$) of each atom of monolayer $TbH_2Br$.

| atom | $x$ | $y$ | $z$ | $U$ | $m_o$ | $m_i$ |
|---|---|---|---|---|---|---|
| Tb | −0.17 | −0.33 | 0.06 | 3.96 | 5.95 | 5.87 |
| Tb | 0.17 | 0.33 | −0.06 | 3.96 | −5.95 | −5.87 |
| H | 0.50 | 0.0 | 0.05 | 0.0 | – | −0.01 |
| H | 0.17 | 0.33 | 0.02 | 0.0 | – | −0.01 |
| Br | 0.17 | 0.33 | 0.14 | 0.0 | – | −0.02 |
| H | 0.50 | 0.0 | −0.05 | 0.0 | – | 0.01 |
| H | −0.17 | −0.33 | −0.02 | 0.0 | – | 0.01 |
| Br | −0.17 | −0.33 | −0.14 | 0.0 | – | 0.02 |

# $TbI_3$ (AFM)

Band gap: 1.31 eV
Total magnetization: 0.0 $\mu_B/cell$
Absolute magnetization: 12.33 $\mu_B/cell$
MC2D entry: https://mc2d.materialscloud.org/#/details/mc2d-1843

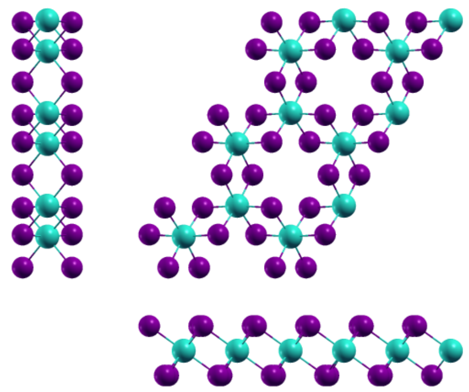

**Geometry:** Views of $TbI_3$ as seen from the $x$ axis (left), the $y$ axis (bottom), and the $z$ axis (center).

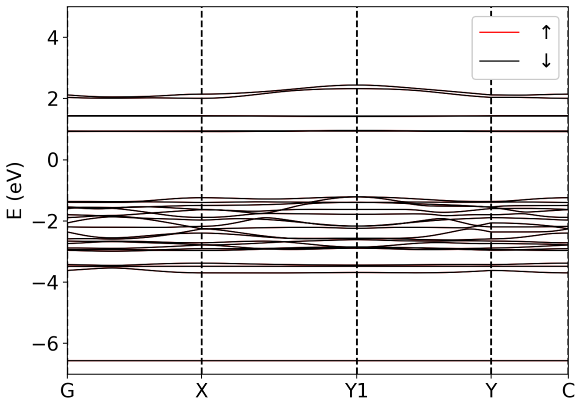


**Electronic bandstructure:** Spin-resolved energy bands of monolayer $TbI_3$ along a high-symmetry path.

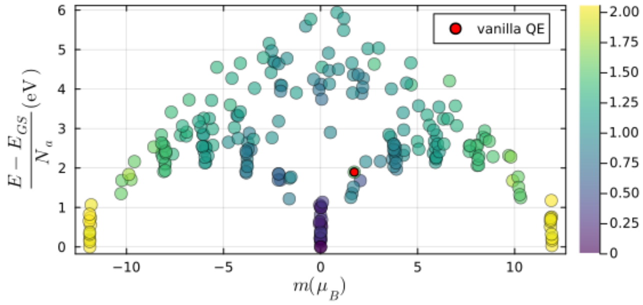


**Unique states:** Representation of 193 self-consistent unique states for monolayer $TbI_3$ identified using RomeoDFT (see Section S6)

**Lattice vectors:** Cartesian components (in [Å]) of the lattice vectors for $TbI_3$.

| | $x$ | $y$ | $z$ |
|---|---|---|---|
| $\boldsymbol{a_1}$ | 4.2068 | 0.0000 | 0.0000 |
| $\boldsymbol{a_2}$ | 0.0000 | 10.0452 | 0.0000 |
| $\boldsymbol{a_3}$ | 0.0000 | 0.0000 | 21.3151 |

**Atomic positions:** Fractional coordinates, Hubbard $U$ (in eV) and magnetic moments (in $\mu_B$, computed from orbital occupations $m_o$ or integration spheres $m_i$) of each atom of monolayer $TbI_3$.

| atom | $x$ | $y$ | $z$ | $U$ | $m_o$ | $m_i$ |
|---|---|---|---|---|---|---|
| Tb | 0.25 | 0.25 | 0.25 | 3.99 | −5.96 | −6.02 |
| Tb | 0.75 | 0.75 | 0.75 | 3.99 | 5.96 | 6.02 |
| I | 0.25 | 0.25 | 0.25 | 0.0 | – | −0.01 |
| I | 0.25 | 0.25 | 0.25 | 0.0 | – | −0.01 |
| I | 0.75 | 0.75 | 0.75 | 0.0 | – | 0.01 |
| I | 0.75 | 0.75 | 0.75 | 0.0 | – | 0.01 |
| I | 0.25 | 0.25 | 0.25 | 0.0 | – | −0.02 |
| I | 0.75 | 0.75 | 0.75 | 0.0 | – | 0.02 |

## TbOBr (FM)

Band gap: 4.45 eV
Total magnetization: $-12.0\ \mu_B/cell$
Absolute magnetization: $12.22\ \mu_B/cell$
MC2D entry: https://mc2d.materialscloud.org/#/details/mc2d-2467

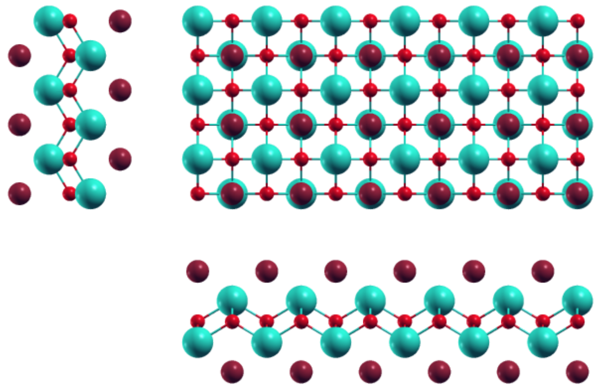

**Geometry:** Views of TbOBr as seen from the $x$ axis (left), the $y$ axis (bottom), and the $z$ axis (center).

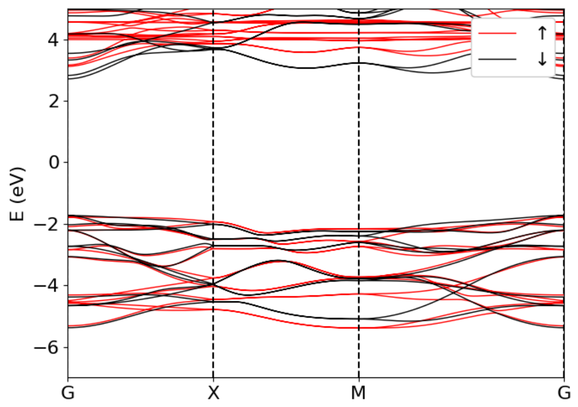


**Electronic bandstructure:** Spin-resolved energy bands of monolayer TbOBr along a high-symmetry path.

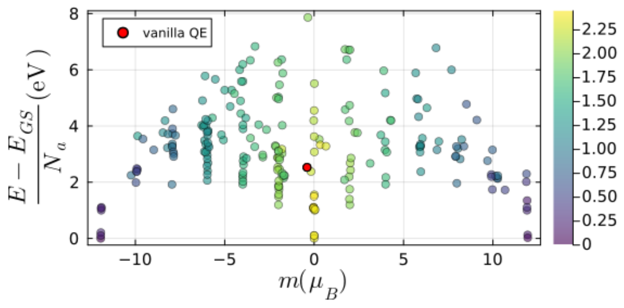


**Unique states:** Representation of 220 self-consistent unique states for monolayer TbOBr identified using RomeoDFT (see Section S6).

**Lattice vectors:** Cartesian components (in [Å]) of the lattice vectors for TbOBr.

| | $x$ | $y$ | $z$ |
|---|---|---|---|
| $\boldsymbol{a_1}$ | 3.8591 | 0.0000 | 0.0000 |
| $\boldsymbol{a_2}$ | 0.0000 | 3.8591 | 0.0000 |
| $\boldsymbol{a_3}$ | 0.0000 | 0.0000 | 22.8911 |

**Atomic positions:** Fractional coordinates, Hubbard $U$ (in eV) and magnetic moments (in $\mu_B$, computed from orbital occupations $m_o$ or integration spheres $m_i$) of each atom of monolayer TbOBr.

| atom | $x$ | $y$ | $z$ | $U$ | $m_o$ | $m_i$ |
|---|---|---|---|---|---|---|
| Tb | 0.25 | −0.25 | 0.05 | 8.21 | −5.97 | −5.90 |
| Tb | −0.25 | −0.75 | −0.05 | 8.21 | −5.97 | −5.90 |
| Br | −0.25 | −0.75 | 0.12 | 0.0 | – | 0.02 |
| Br | 0.25 | −0.25 | −0.12 | 0.0 | – | 0.02 |
| O | −0.25 | −0.25 | 0.0 | 0.0 | – | 0.03 |
| O | 0.25 | −0.75 | 0.0 | 0.0 | – | 0.03 |

# $TbTe_3$ (AFM)

Band gap: 0.0 eV
Total magnetization: 0.0 $\mu_B/cell$
Absolute magnetization: 12.35 $\mu_B/cell$
MC2D entry: https://mc2d.materialscloud.org/#/details/mc2d-2408

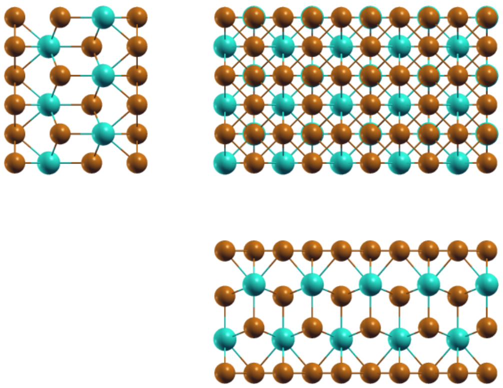

**Geometry:** Views of $TbTe_3$ as seen from the $x$ axis (left), the $y$ axis (bottom), and the $z$ axis (center).

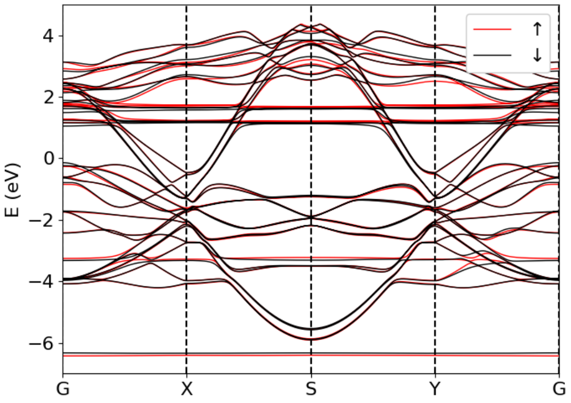


**Electronic bandstructure:** Spin-resolved energy bands of monolayer $TbTe_3$ along a high-symmetry path.

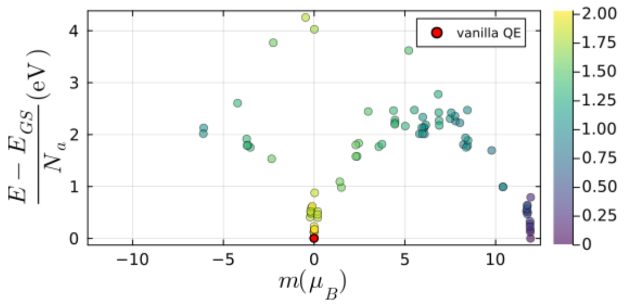


**Unique states:** Representation of 83 self-consistent unique states for monolayer $TbTe_3$ identified using RomeoDFT (see Section S6).

**Lattice vectors:** Cartesian components (in [Å]) of the lattice vectors for $TbTe_3$.

| | $x$ | $y$ | $z$ |
|---|---|---|---|
| $a_1$ | 4.3514 | 0.0000 | 0.0000 |
| $a_2$ | 0.0000 | 4.3513 | 0.0000 |
| $a_3$ | 0.0000 | 0.0000 | 29.9309 |

**Atomic positions:** Fractional coordinates, Hubbard $U$ (in eV) and magnetic moments (in $\mu_B$, computed from orbital occupations $m_o$ or integration spheres $m_i$) of each atom of monolayer $TbTe_3$.

| atom | $x$ | $y$ | $z$ | $U$ | $m_o$ | $m_i$ |
|---|---|---|---|---|---|---|
| Tb | −0.25 | −0.25 | −0.07 | 4.03 | 5.96 | 6.04 |
| Tb | 0.25 | −0.75 | 0.07 | 4.03 | −5.96 | −6.04 |
| Te | 0.25 | −0.25 | 0.15 | 0.0 | – | 0.01 |
| Te | −0.25 | −0.75 | −0.15 | 0.0 | – | −0.01 |
| Te | 0.25 | −0.25 | −0.15 | 0.0 | – | −0.01 |
| Te | −0.25 | −0.75 | 0.15 | 0.0 | – | 0.01 |
| Te | −0.25 | −0.25 | 0.04 | 0.0 | – | 0.02 |
| Te | 0.25 | −0.75 | −0.04 | 0.0 | – | −0.02 |

## $TiBr_2$ (AFM)

Band gap: 3.70 eV
Total magnetization: 0.0 $\mu_B/cell$
Absolute magnetization: 4.09 $\mu_B/cell$
MC2D entry: https://mc2d.materialscloud.org/#/details/mc2d-40

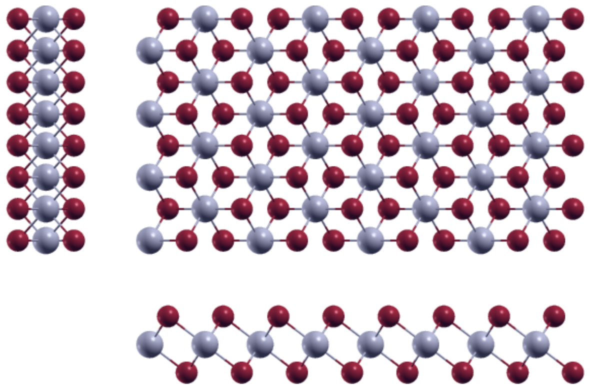

**Geometry:** Views of $TiBr_2$ as seen from the $x$ axis (left), the $y$ axis (bottom), and the $z$ axis (center).

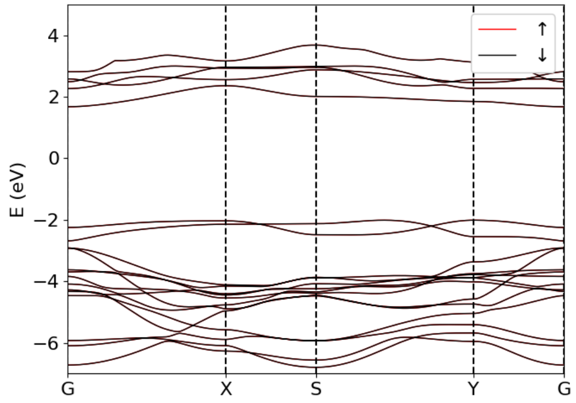


**Electronic bandstructure:** Spin-resolved energy bands of monolayer $TiBr_2$ along a high-symmetry path.

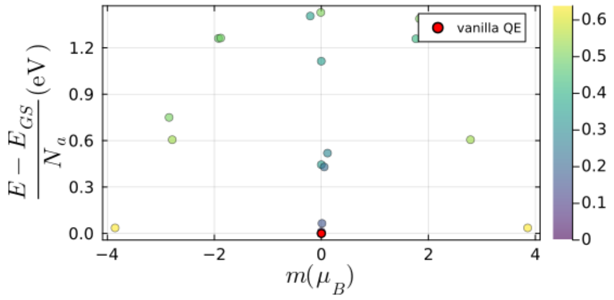


**Unique states:** Representation of 20 self-consistent unique states for monolayer $TiBr_2$ identified using RomeoDFT (see Section S6).

**Lattice vectors:** Cartesian components (in [Å]) of the lattice vectors for $TiBr_2$.

| | $x$ | $y$ | $z$ |
|---|---|---|---|
| $\boldsymbol{a_1}$ | 0.0000 | −6.3753 | 0.0000 |
| $\boldsymbol{a_2}$ | 3.6553 | 0.0000 | 0.0000 |
| $\boldsymbol{a_3}$ | 0.0000 | 0.0000 | 23.3858 |

**Atomic positions:** Fractional coordinates, Hubbard $U$ (in eV) and magnetic moments (in $\mu_B$, computed from orbital occupations $m_o$ or integration spheres $m_i$) of each atom of monolayer $TiBr_2$.

| atom | $x$ | $y$ | $z$ | $U$ | $m_o$ | $m_i$ |
|---|---|---|---|---|---|---|
| Ti | 0.50 | 0.50 | 0.50 | 5.08 | 1.91 | 1.63 |
| Ti | 1.00 | 0.0 | 0.50 | 5.08 | −1.91 | −1.63 |
| Br | 0.67 | 0.0 | 0.43 | 0.0 | – | 0.00 |
| Br | 1.17 | 0.50 | 0.43 | 0.0 | – | 0.00 |
| Br | 0.83 | 0.50 | 0.57 | 0.0 | – | 0.00 |
| Br | 0.33 | 0.0 | 0.57 | 0.0 | – | 0.00 |

## $TiBr_3$ (FM)

Band gap: 2.89 eV
Total magnetization: 2.0 $\mu_B/cell$
Absolute magnetization: 2.46 $\mu_B/cell$
MC2D entry: https://mc2d.materialscloud.org/#/details/mc2d-1490

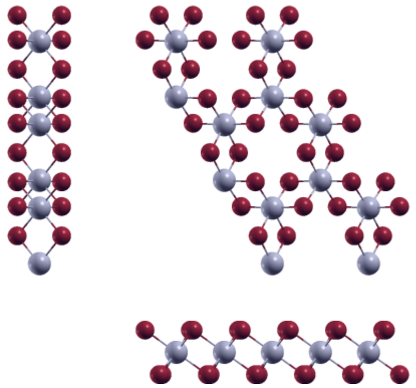

**Geometry:** Views of $TiBr_3$ as seen from the $x$ axis (left), the $y$ axis (bottom), and the $z$ axis (center).

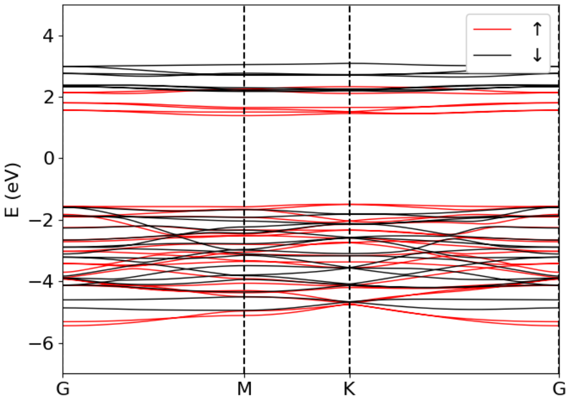


**Electronic bandstructure:** Spin-resolved energy bands of monolayer $TiBr_3$ along a high-symmetry path.

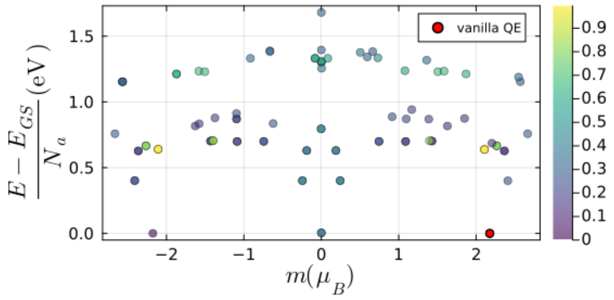


**Unique states:** Representation of 91 self-consistent unique states for monolayer $TiBr_3$ identified using RomeoDFT (see Section S6).

**Lattice vectors:** Cartesian components (in [Å]) of the lattice vectors for $TiBr_3$.

| | $x$ | $y$ | $z$ |
|---|---|---|---|
| $\boldsymbol{a_1}$ | 6.5062 | 0.0000 | 0.0000 |
| $\boldsymbol{a_2}$ | −3.2531 | 5.6346 | 0.0000 |
| $\boldsymbol{a_3}$ | 0.0000 | 0.0000 | 23.0394 |

**Atomic positions:** Fractional coordinates, Hubbard $U$ (in eV) and magnetic moments (in $\mu_B$, computed from orbital occupations $m_o$ or integration spheres $m_i$) of each atom of monolayer $TiBr_3$.

| atom | $x$ | $y$ | $z$ | $U$ | $m_o$ | $m_i$ |
|---|---|---|---|---|---|---|
| Ti | 0.33 | 0.67 | 0.50 | 5.22 | 1.09 | 0.94 |
| Ti | 1.00 | 1.00 | 0.50 | 5.22 | 1.09 | 0.94 |
| Br | 0.31 | 0.33 | 0.43 | 0.0 | – | −0.03 |
| Br | 0.67 | 0.98 | 0.43 | 0.0 | – | −0.03 |
| Br | 0.02 | 0.69 | 0.43 | 0.0 | – | −0.03 |
| Br | 0.02 | 0.33 | 0.57 | 0.0 | – | −0.03 |
| Br | 0.67 | 0.69 | 0.57 | 0.0 | – | −0.03 |
| Br | 0.31 | 0.98 | 0.57 | 0.0 | – | −0.03 |

# $TiCl_3$ (FM)

Band gap: 2.50 eV
Total magnetization: $-2.0\ \mu_B/cell$
Absolute magnetization: 2.25 $\mu_B/cell$
MC2D entry: https://mc2d.materialscloud.org/#/details/mc2d-1369

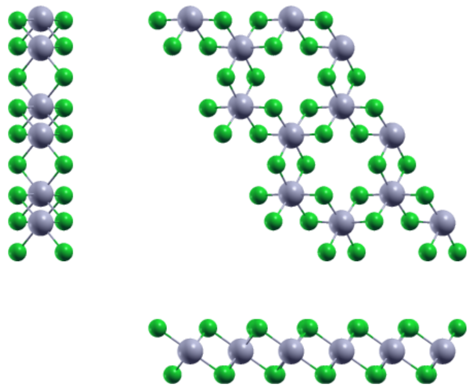

**Geometry:** Views of $TiCl_3$ as seen from the $x$ axis (left), the $y$ axis (bottom), and the $z$ axis (center).

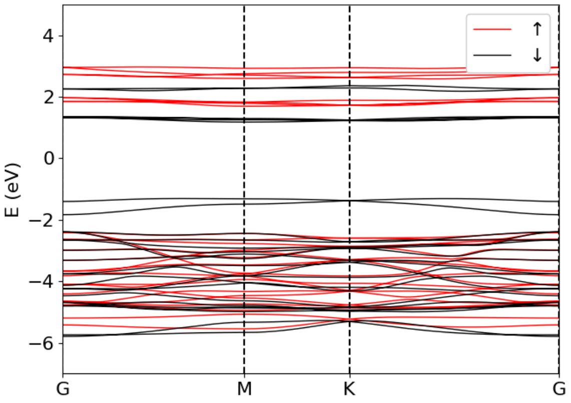


**Electronic bandstructure:** Spin-resolved energy bands of monolayer $TiCl_3$ along a high-symmetry path.

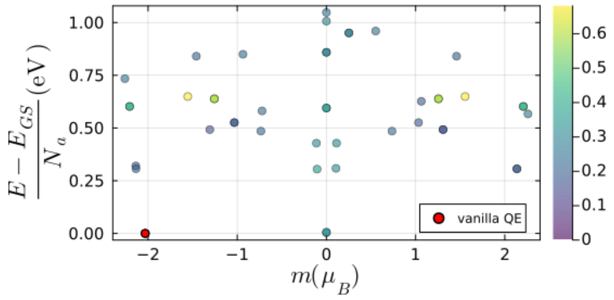


**Unique states:** Representation of 46 self-consistent unique states for monolayer $TiCl_3$ identified using RomeoDFT (see Section S6).

**Lattice vectors:** Cartesian components (in [Å]) of the lattice vectors for $TiCl_3$.

| | $x$ | $y$ | $z$ |
|---|---|---|---|
| $\boldsymbol{a_1}$ | 6.1091 | 0.0000 | 0.0000 |
| $\boldsymbol{a_2}$ | −3.0545 | 5.2906 | 0.0000 |
| $\boldsymbol{a_3}$ | 0.0000 | 0.0000 | 22.8356 |

**Atomic positions:** Fractional coordinates, Hubbard $U$ (in eV) and magnetic moments (in $\mu_B$, computed from orbital occupations $m_o$ or integration spheres $m_i$) of each atom of monolayer $TiCl_3$.

| atom | $x$ | $y$ | $z$ | $U$ | $m_o$ | $m_i$ |
|---|---|---|---|---|---|---|
| Ti | 0.67 | 0.33 | 0.50 | 3.72 | −1.02 | −0.85 |
| Ti | 0.33 | 0.67 | 0.50 | 3.72 | −1.02 | −0.85 |
| Cl | 0.0 | 0.35 | 0.56 | 0.0 | – | 0.01 |
| Cl | 0.65 | 0.65 | 0.56 | 0.0 | – | 0.01 |
| Cl | 0.35 | 1.00 | 0.56 | 0.0 | – | 0.01 |
| Cl | 1.00 | 0.65 | 0.44 | 0.0 | – | 0.01 |
| Cl | 0.35 | 0.35 | 0.44 | 0.0 | – | 0.01 |
| Cl | 0.65 | 1.00 | 0.44 | 0.0 | – | 0.01 |

# $TiI_2$ (AFM)

Band gap: 2.57 eV
Total magnetization: $-0.0\ \mu_B/cell$
Absolute magnetization: 4.16 $\mu_B/cell$
MC2D entry: https://mc2d.materialscloud.org/#/details/mc2d-2266

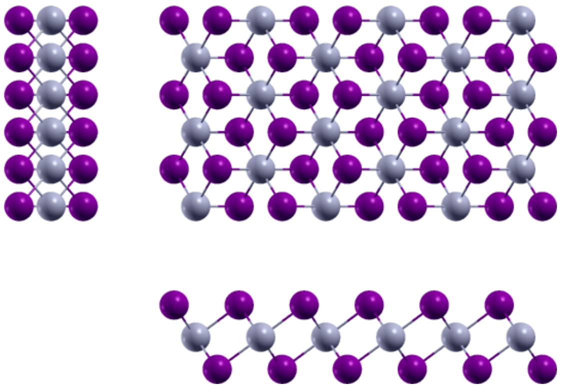

**Geometry:** Views of $TiI_2$ as seen from the $x$ axis (left), the $y$ axis (bottom), and the $z$ axis (center).

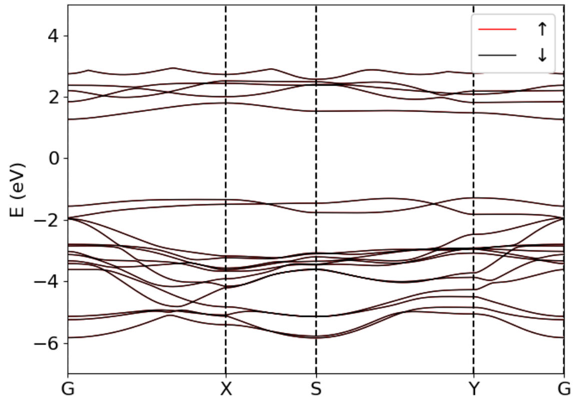


**Electronic bandstructure:** Spin-resolved energy bands of monolayer $TiI_2$ along a high-symmetry path.

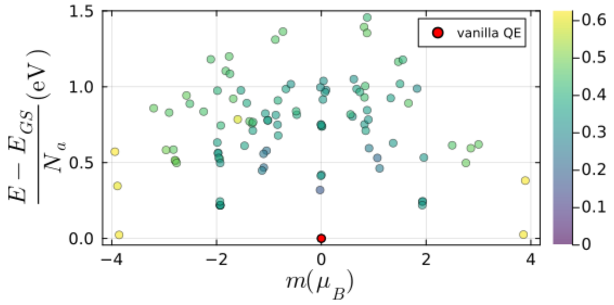


**Unique states:** Representation of 104 self-consistent unique states for monolayer $TiI_2$ identified using RomeoDFT (see Section S6).

**Lattice vectors:** Cartesian components (in [Å]) of the lattice vectors for $TiI_2$.

| | $x$ | $y$ | $z$ |
|---|---|---|---|
| $\boldsymbol{a_1}$ | −6.0294 | −3.4811 | 0.0000 |
| $\boldsymbol{a_2}$ | 1.9896 | −3.4460 | 0.0000 |
| $\boldsymbol{a_3}$ | 0.0000 | 0.0000 | 19.0450 |

**Atomic positions:** Fractional coordinates, Hubbard $U$ (in eV) and magnetic moments (in $\mu_B$, computed from orbital occupations $m_o$ or integration spheres $m_i$) of each atom of monolayer $TiI_2$.

| atom | $x$ | $y$ | $z$ | $U$ | $m_o$ | $m_i$ |
|---|---|---|---|---|---|---|
| Ti | 0.75 | 0.75 | 0.0 | 3.65 | 1.90 | 1.67 |
| Ti | 0.25 | 0.25 | 0.0 | 3.65 | −1.90 | −1.67 |
| I | 0.92 | 0.25 | 0.09 | 0.0 | – | −0.01 |
| I | 0.42 | 0.75 | 0.09 | 0.0 | – | 0.01 |
| I | 0.58 | 1.25 | 0.91 | 0.0 | – | −0.01 |
| I | 1.08 | 0.75 | 0.91 | 0.0 | – | 0.01 |

## $TmBr_3$ (AFM)

Band gap: 1.56 eV
Total magnetization: $-0.0\ \mu_B/cell$
Absolute magnetization: 4.01 $\mu_B/cell$
MC2D entry: https://mc2d.materialscloud.org/#/details/mc2d-2654

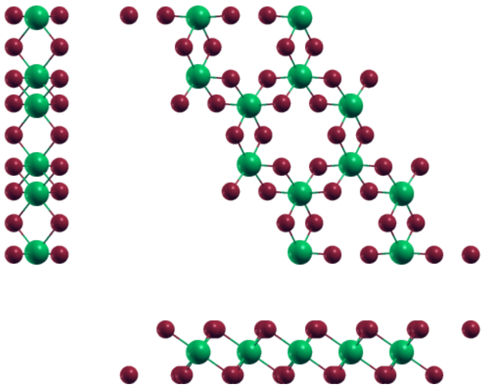

**Geometry:** Views of $TmBr_3$ as seen from the $x$ axis (left), the $y$ axis (bottom), and the $z$ axis (center).

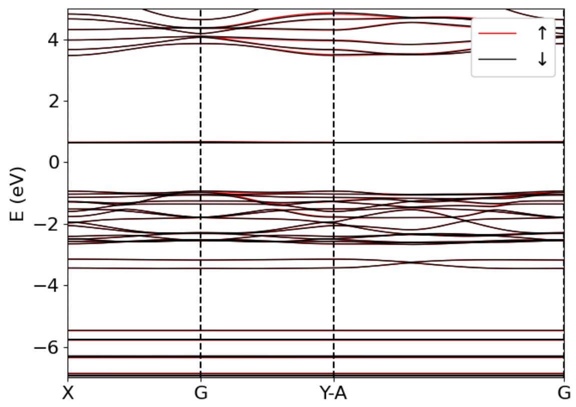


**Electronic bandstructure:** Spin-resolved energy bands of monolayer $TmBr_3$ along a high-symmetry path.

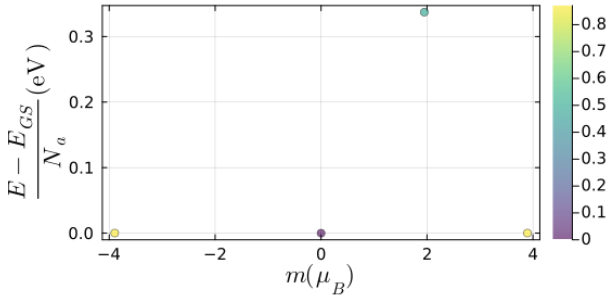


**Unique states:** Representation of 5 self-consistent unique states for monolayer $TmBr_3$ identified using RomeoDFT (see Section S6).

**Lattice vectors:** Cartesian components (in [Å]) of the lattice vectors for $TmBr_3$.

| | $x$ | $y$ | $z$ |
|---|---|---|---|
| $\mathbf{a_1}$ | −3.5810 | −6.2023 | 0.0000 |
| $\mathbf{a_2}$ | 7.1619 | 0.0000 | 0.0000 |
| $\mathbf{a_3}$ | 0.0000 | 0.0000 | 18.3304 |

**Atomic positions:** Fractional coordinates, Hubbard $U$ (in eV) and magnetic moments (in $\mu_B$, computed from orbital occupations $m_o$ or integration spheres $m_i$) of each atom of monolayer $TmBr_3$.

| atom | $x$ | $y$ | $z$ | $U$ | $m_o$ | $m_i$ |
|---|---|---|---|---|---|---|
| Tm | −0.33 | −0.17 | 0.00 | 5.86 | −1.95 | −1.97 |
| Tm | 0.33 | 0.17 | 0.00 | 5.84 | 1.95 | 1.97 |
| Br | 0.64 | 0.50 | −0.09 | 0.0 | – | 0.00 |
| Br | 0.00 | −0.14 | 0.09 | 0.0 | – | 0.00 |
| Br | 0.64 | 0.14 | 0.09 | 0.0 | – | 0.00 |
| Br | 0.36 | 0.50 | 0.09 | 0.0 | – | 0.00 |
| Br | 0.00 | 0.14 | −0.09 | 0.0 | – | 0.0 |
| Br | 0.36 | −0.14 | −0.09 | 0.0 | – | 0.0 |

## TmBrF (FM)

Band gap: 0.0 eV
Total magnetization: 2.04 $\mu_B/cell$
Absolute magnetization: 2.18 $\mu_B/cell$
MC2D entry: https://mc2d.materialscloud.org/#/details/mc2d-2555

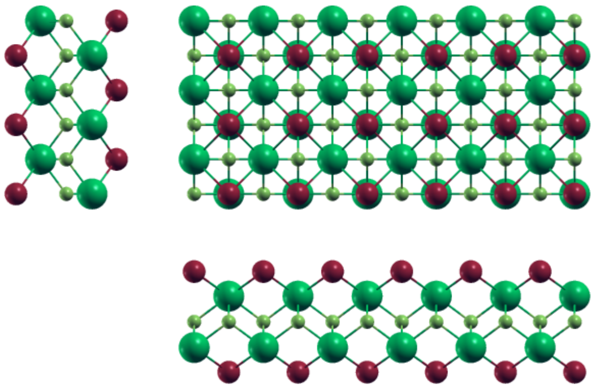

**Geometry:** Views of TmBrF as seen from the $x$ axis (left), the $y$ axis (bottom), and the $z$ axis (center).

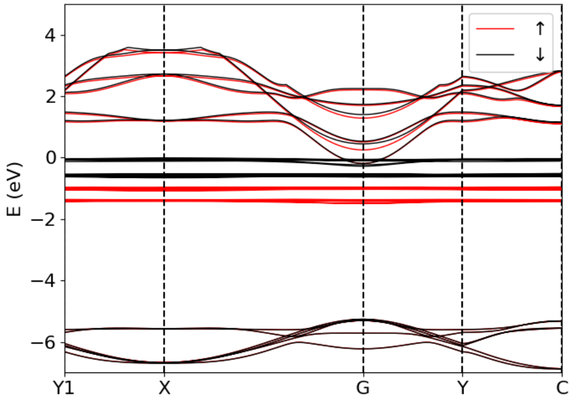


**Electronic bandstructure:** Spin-resolved energy bands of monolayer TmBrF along a high-symmetry path.

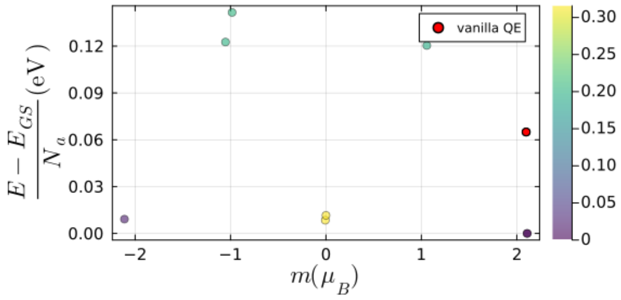


**Unique states:** Representation of 8 self-consistent unique states for monolayer TmBrF identified using RomeoDFT (see Section S6).

**Lattice vectors:** Cartesian components (in [Å]) of the lattice vectors for TmBrF.

| | $x$ | $y$ | $z$ |
|---|---|---|---|
| $\boldsymbol{a_1}$ | 3.8815 | 0.0001 | 0.0000 |
| $\boldsymbol{a_2}$ | 0.0001 | 3.8815 | 0.0000 |
| $\boldsymbol{a_3}$ | 0.0000 | 0.0000 | 22.3313 |

**Atomic positions:** Fractional coordinates, Hubbard $U$ (in eV) and magnetic moments (in $\mu_B$, computed from orbital occupations $m_o$ or integration spheres $m_i$) of each atom of monolayer TmBrF.

| atom | $x$ | $y$ | $z$ | $U$ | $m_o$ | $m_i$ |
|---|---|---|---|---|---|---|
| Tm | 0.25 | −0.25 | 0.06 | 0.00 | 1.05 | 1.04 |
| Tm | −0.25 | −0.75 | −0.06 | 0.00 | 1.05 | 1.04 |
| Br | −0.25 | −0.75 | 0.13 | 0.0 | – | 0.00 |
| Br | 0.25 | −0.25 | −0.13 | 0.0 | – | 0.00 |
| F | −0.25 | −0.25 | 0.0 | 0.0 | – | 0.00 |
| F | 0.25 | −0.75 | 0.0 | 0.0 | – | 0.00 |

# $TmCl_3$ (AFM)

Band gap: 1.24 eV
Total magnetization: 0.0 $\mu_B/cell$
Absolute magnetization: 4.06 $\mu_B/cell$
MC2D entry: https://mc2d.materialscloud.org/#/details/mc2d-2471

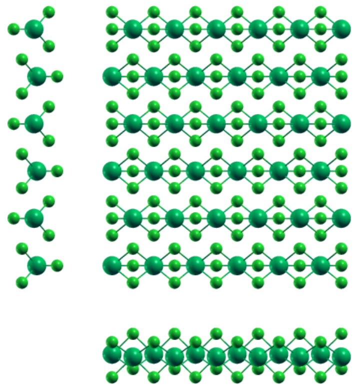

**Geometry:** Views of $TmCl_3$ as seen from the $x$ axis (left), the $y$ axis (bottom), and the $z$ axis (center).

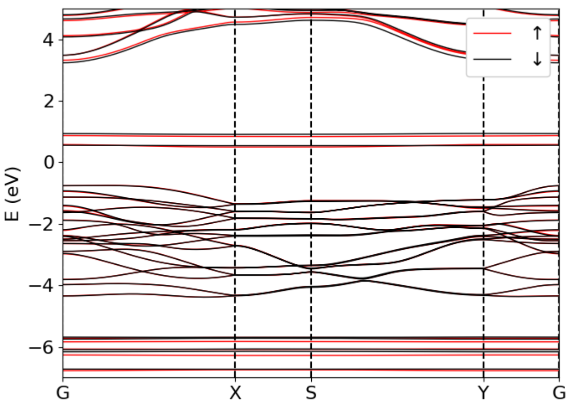


**Electronic bandstructure:** Spin-resolved energy bands of monolayer $TmCl_3$ along a high-symmetry path.

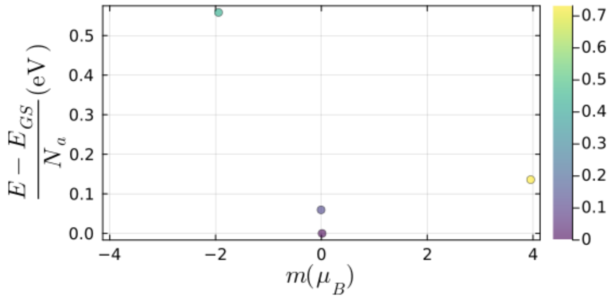


**Unique states:** Representation of 5 self-consistent unique states for monolayer $TmCl_3$ identified using RomeoDFT (see Section S6).

**Lattice vectors:** Cartesian components (in [Å]) of the lattice vectors for $TmCl_3$.

| | $x$ | $y$ | $z$ |
|---|---|---|---|
| $\boldsymbol{a_1}$ | 3.7761 | 0.0000 | 0.0000 |
| $\boldsymbol{a_2}$ | 0.0000 | 8.5706 | 0.0000 |
| $\boldsymbol{a_3}$ | 0.0000 | 0.0000 | 19.7731 |

**Atomic positions:** Fractional coordinates, Hubbard $U$ (in eV) and magnetic moments (in $\mu_B$, computed from orbital occupations $m_o$ or integration spheres $m_i$) of each atom of monolayer $TmCl_3$.

| atom | $x$ | $y$ | $z$ | $U$ | $m_o$ | $m_i$ |
|---|---|---|---|---|---|---|
| Tm | 0.25 | −0.75 | −0.01 | 6.01 | 1.99 | 2.00 |
| Tm | −0.25 | −1.25 | 0.01 | 6.01 | −1.98 | −1.99 |
| Cl | −0.25 | −0.57 | 0.06 | 0.0 | – | 0.00 |
| Cl | −0.25 | −0.93 | 0.06 | 0.0 | – | 0.00 |
| Cl | 0.25 | −1.07 | −0.06 | 0.0 | – | 0.00 |
| Cl | 0.25 | −1.43 | −0.06 | 0.0 | – | 0.00 |
| Cl | −0.25 | −0.75 | −0.10 | 0.0 | – | 0.00 |
| Cl | 0.25 | −1.25 | 0.10 | 0.0 | – | 0.00 |

# $TmI_2$ (FM)

Band gap: 0.0 eV
Total magnetization: 2.05 $\mu_B/cell$
Absolute magnetization: 2.22 $\mu_B/cell$
MC2D entry: https://mc2d.materialscloud.org/#/details/mc2d-185

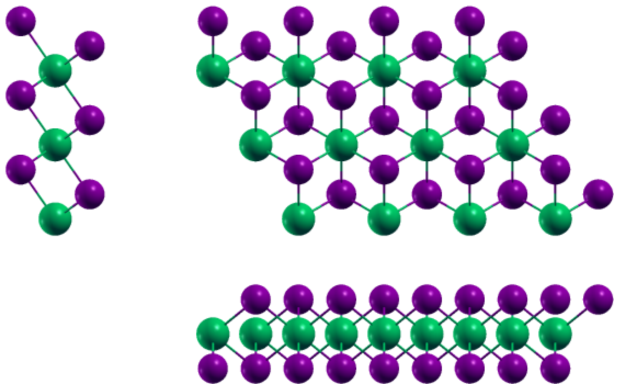

**Geometry:** Views of $TmI_2$ as seen from the $x$ axis (left), the $y$ axis (bottom), and the $z$ axis (center).

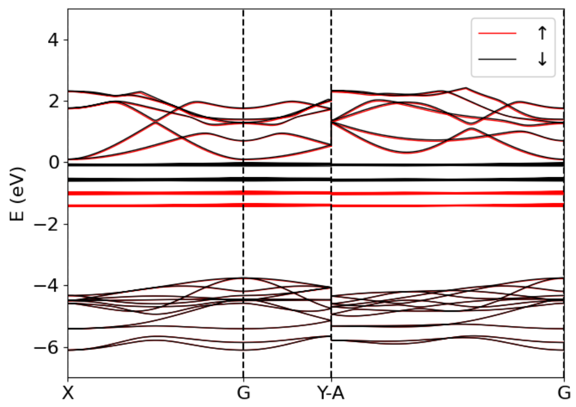


**Electronic bandstructure:** Spin-resolved energy bands of monolayer $TmI_2$ along a high-symmetry path.

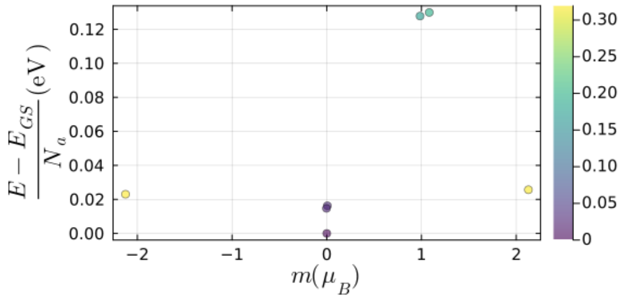


**Unique states:** Representation of 7 self-consistent unique states for monolayer $TmI_2$ identified using RomeoDFT (see Section S6).

**Lattice vectors:** Cartesian components (in [Å]) of the lattice vectors for $TmI_2$.

| | $x$ | $y$ | $z$ |
|---|---|---|---|
| $\boldsymbol{a_1}$ | 8.9383 | 0.0000 | 0.0000 |
| $\boldsymbol{a_2}$ | −2.2346 | 3.8704 | 0.0000 |
| $\boldsymbol{a_3}$ | 0.0000 | 0.0000 | 23.6051 |

**Atomic positions:** Fractional coordinates, Hubbard $U$ (in eV) and magnetic moments (in $\mu_B$, computed from orbital occupations $m_o$ or integration spheres $m_i$) of each atom of monolayer $TmI_2$.

| atom | $x$ | $y$ | $z$ | $U$ | $m_o$ | $m_i$ |
|---|---|---|---|---|---|---|
| Tm | 0.0 | 0.0 | 0.50 | 0.00 | 1.06 | 1.06 |
| Tm | 0.50 | 0.0 | 0.50 | 0.00 | 1.07 | 1.06 |
| I | 0.17 | 0.67 | 0.58 | 0.0 | – | 0.00 |
| I | 0.33 | 0.33 | 0.42 | 0.0 | – | 0.00 |
| I | 0.67 | 0.67 | 0.58 | 0.0 | – | 0.00 |
| I | 0.83 | 0.33 | 0.42 | 0.0 | – | 0.00 |

# $TmI_3$ (FM)

Band gap: 0.83 eV
Total magnetization: $-3.86\ \mu_B/cell$
Absolute magnetization: $4.08\ \mu_B/cell$
MC2D entry: https://mc2d.materialscloud.org/#/details/mc2d-2187

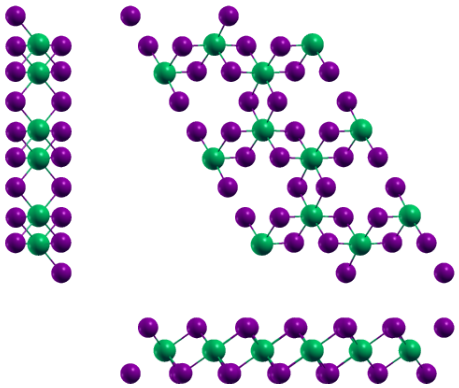

**Geometry:** Views of $TmI_3$ as seen from the $x$ axis (left), the $y$ axis (bottom), and the $z$ axis (center).

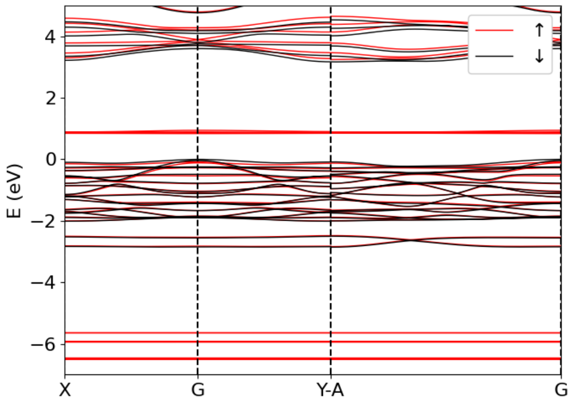


**Electronic bandstructure:** Spin-resolved energy bands of monolayer $TmI_3$ along a high-symmetry path.

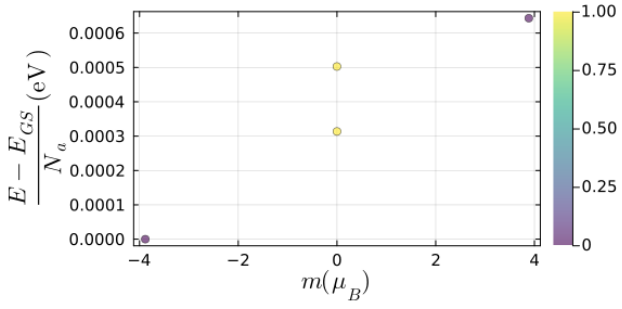


**Unique states:** Representation of 5 self-consistent unique states for monolayer $TmI_3$ identified using RomeoDFT (see Section S6).

**Lattice vectors:** Cartesian components (in [Å]) of the lattice vectors for $TmI_3$.

| | $x$ | $y$ | $z$ |
|---|---|---|---|
| $\boldsymbol{a_1}$ | −3.7724 | −6.5745 | 0.0000 |
| $\boldsymbol{a_2}$ | 7.6026 | −0.0335 | 0.0000 |
| $\boldsymbol{a_3}$ | 0.0000 | 0.0000 | 19.0007 |

**Atomic positions:** Fractional coordinates, Hubbard $U$ (in eV) and magnetic moments (in $\mu_B$, computed from orbital occupations $m_o$ or integration spheres $m_i$) of each atom of monolayer $TmI_3$.

| atom | $x$ | $y$ | $z$ | $U$ | $m_o$ | $m_i$ |
|---|---|---|---|---|---|---|
| Tm | −0.16 | −0.33 | 0.00 | 6.29 | −1.94 | −1.97 |
| Tm | 0.16 | 0.33 | 0.00 | 6.29 | −1.94 | −1.97 |
| I | 0.50 | 0.65 | 0.09 | 0.0 | – | 0.01 |
| I | −0.15 | 0.00 | −0.09 | 0.0 | – | 0.01 |
| I | 0.15 | 0.65 | −0.09 | 0.0 | – | 0.01 |
| I | 0.50 | 0.35 | −0.09 | 0.0 | – | 0.01 |
| I | 0.15 | 0.00 | 0.09 | 0.0 | – | 0.01 |
| I | −0.15 | 0.35 | 0.09 | 0.0 | – | 0.02 |

# TmOBr (AFM)

Band gap: 2.22 eV
Total magnetization: $-0.0\ \mu_B/cell$
Absolute magnetization: $4.02\ \mu_B/cell$
MC2D entry: https://mc2d.materialscloud.org/#/details/mc2d-2280

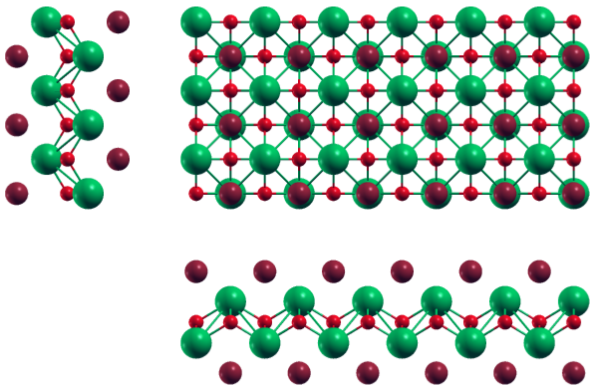

**Geometry:** Views of TmOBr as seen from the $x$ axis (left), the $y$ axis (bottom), and the $z$ axis (center).

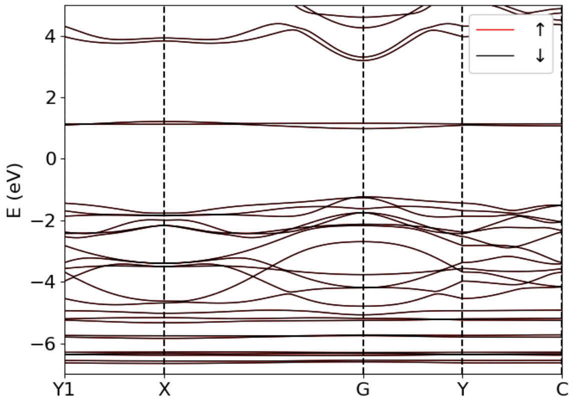


**Electronic bandstructure:** Spin-resolved energy bands of monolayer TmOBr along a high-symmetry path.

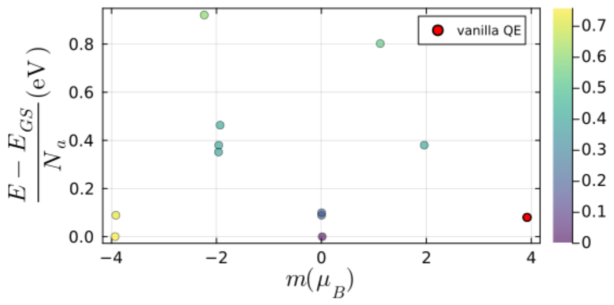


**Unique states:** Representation of 13 self-consistent unique states for monolayer TmOBr identified using RomeoDFT (see Section S6).

**Lattice vectors:** Cartesian components (in [Å]) of the lattice vectors for TmOBr.

| | $x$ | $y$ | $z$ |
|---|---|---|---|
| $\boldsymbol{a}_1$ | 3.8090 | 0.0005 | 0.0000 |
| $\boldsymbol{a}_2$ | 0.0005 | 3.8090 | 0.0000 |
| $\boldsymbol{a}_3$ | 0.0000 | 0.0000 | 22.9516 |

**Atomic positions:** Fractional coordinates, Hubbard $U$ (in eV) and magnetic moments (in $\mu_B$, computed from orbital occupations $m_o$ or integration spheres $m_i$) of each atom of monolayer TmOBr.

| atom | $x$ | $y$ | $z$ | $U$ | $m_o$ | $m_i$ |
|---|---|---|---|---|---|---|
| Tm | 0.25 | −0.25 | 0.05 | 5.67 | −1.96 | −1.97 |
| Tm | −0.25 | −0.75 | −0.05 | 5.67 | 1.96 | 1.97 |
| Br | −0.25 | −0.75 | 0.12 | 0.0 | – | 0.00 |
| Br | 0.25 | −0.25 | −0.12 | 0.0 | – | 0.00 |
| O | −0.25 | −0.25 | 0.0 | 0.0 | – | 0.0 |
| O | 0.25 | −0.75 | 0.0 | 0.0 | – | 0.0 |

# TmOI (AFM)

Band gap: 1.74 eV
Total magnetization: $-0.0$ $\mu_B/cell$
Absolute magnetization: 4.03 $\mu_B/cell$
MC2D entry: https://mc2d.materialscloud.org/#/details/mc2d-196

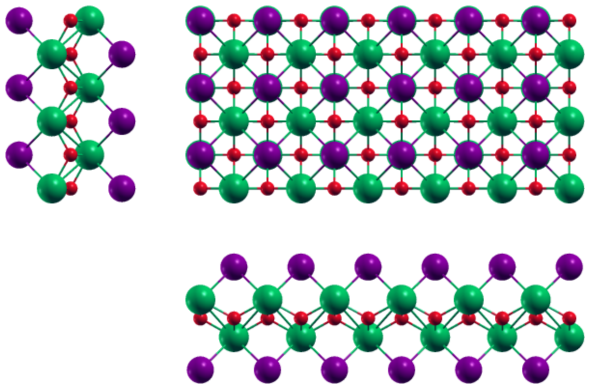

**Geometry:** Views of TmOI as seen from the $x$ axis (left), the $y$ axis (bottom), and the $z$ axis (center).

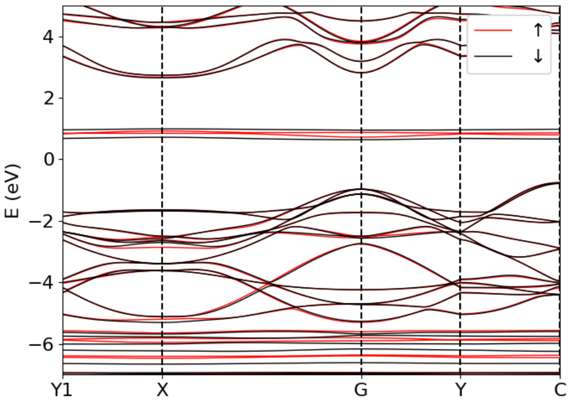


**Electronic bandstructure:** Spin-resolved energy bands of monolayer TmOI along a high-symmetry path.

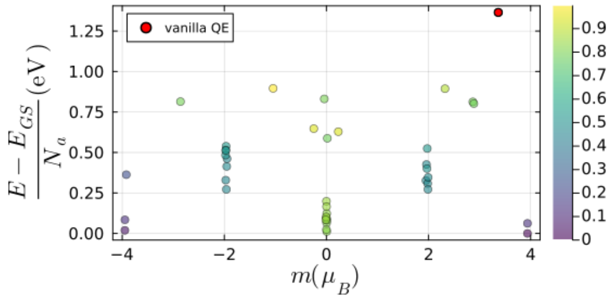


**Unique states:** Representation of 41 self-consistent unique states for monolayer TmOI identified using RomeoDFT (see Section S6).

**Lattice vectors:** Cartesian components (in [Å]) of the lattice vectors for TmOI.

| | $x$ | $y$ | $z$ |
|---|---|---|---|
| $\boldsymbol{a_1}$ | 3.8868 | 0.0003 | 0.0000 |
| $\boldsymbol{a_2}$ | 0.0003 | 3.8868 | 0.0000 |
| $\boldsymbol{a_3}$ | 0.0000 | 0.0000 | 23.9040 |

**Atomic positions:** Fractional coordinates, Hubbard $U$ (in eV) and magnetic moments (in $\mu_B$, computed from orbital occupations $m_o$ or integration spheres $m_i$) of each atom of monolayer TmOI.

| atom | $x$ | $y$ | $z$ | $U$ | $m_o$ | $m_i$ |
|---|---|---|---|---|---|---|
| Tm | −0.25 | −0.75 | 0.05 | 6.01 | 1.97 | 1.97 |
| Tm | 0.25 | −0.25 | −0.05 | 6.01 | −1.97 | −1.97 |
| I | 0.25 | −0.25 | 0.12 | 0.0 | – | 0.00 |
| I | −0.25 | −0.75 | −0.12 | 0.0 | – | 0.00 |
| O | −0.25 | −0.25 | 0.0 | 0.0 | – | 0.0 |
| O | 0.25 | −0.75 | 0.0 | 0.0 | – | 0.0 |

## TmSeI (FM)

Band gap: 0.87 eV
Total magnetization: 3.87 $\mu_B/cell$
Absolute magnetization: 4.09 $\mu_B/cell$
MC2D entry: https://mc2d.materialscloud.org/#/details/mc2d-2636

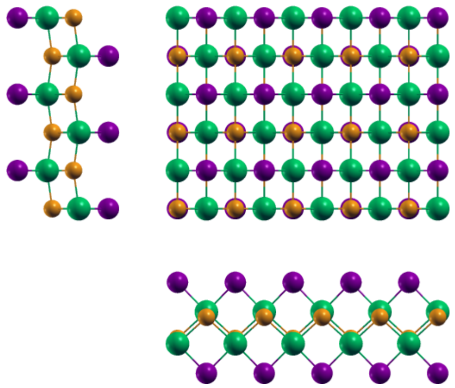

**Geometry:** Views of TmSeI as seen from the $x$ axis (left), the $y$ axis (bottom), and the $z$ axis (center).

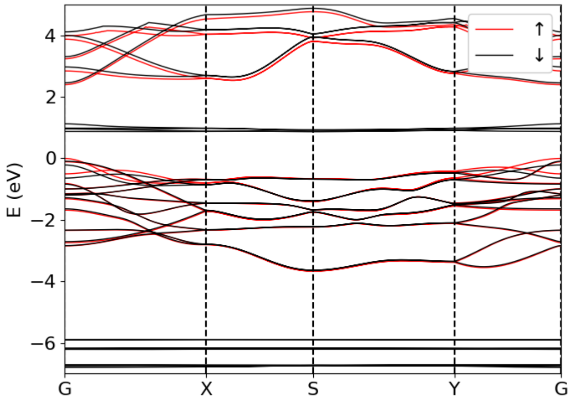


**Electronic bandstructure:** Spin-resolved energy bands of monolayer TmSeI along a high-symmetry path.

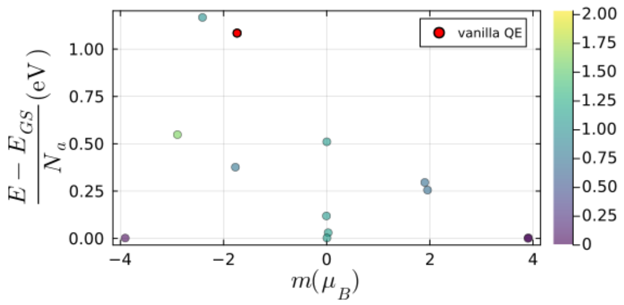


**Unique states:** Representation of 13 self-consistent unique states for monolayer TmSeI identified using RomeoDFT (see Section S6).

**Lattice vectors:** Cartesian components (in [Å]) of the lattice vectors for TmSeI.

| | $x$ | $y$ | $z$ |
|---|---|---|---|
| $\boldsymbol{a_1}$ | −4.2302 | 0.0000 | 0.0000 |
| $\boldsymbol{a_2}$ | 0.0000 | −5.5878 | 0.0000 |
| $\boldsymbol{a_3}$ | 0.0000 | 0.0000 | 24.9874 |

**Atomic positions:** Fractional coordinates, Hubbard $U$ (in eV) and magnetic moments (in $\mu_B$, computed from orbital occupations $m_o$ or integration spheres $m_i$) of each atom of monolayer TmSeI.

| atom | $x$ | $y$ | $z$ | $U$ | $m_o$ | $m_i$ |
|---|---|---|---|---|---|---|
| Tm | −0.25 | 0.25 | −0.55 | 6.58 | 1.95 | 1.97 |
| Tm | 0.25 | −0.25 | −0.45 | 6.58 | 1.95 | 1.97 |
| I | 0.25 | 0.25 | −0.63 | 0.0 | – | −0.02 |
| Se | −0.25 | −0.25 | −0.53 | 0.0 | – | −0.02 |
| Se | 0.25 | 0.25 | −0.47 | 0.0 | – | −0.02 |
| I | −0.25 | −0.25 | −0.37 | 0.0 | – | −0.02 |

# $V_2NiSe_4$ (FM)

Band gap: 0.89 eV
Total magnetization: −4.0 $\mu_B/cell$
Absolute magnetization: 14.59 $\mu_B/cell$
MC2D entry: https://mc2d.materialscloud.org/#/details/mc2d-1439

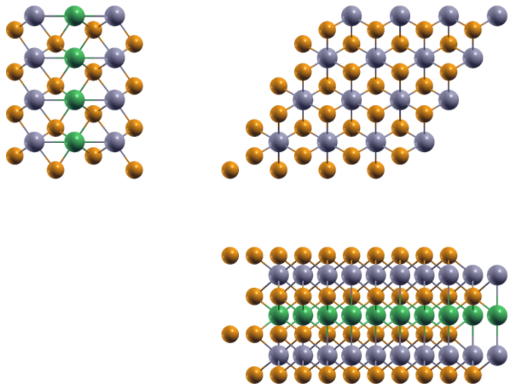

**Geometry:** Views of $V_2NiSe_4$ as seen from the $x$ axis (left), the $y$ axis (bottom), and the $z$ axis (center).

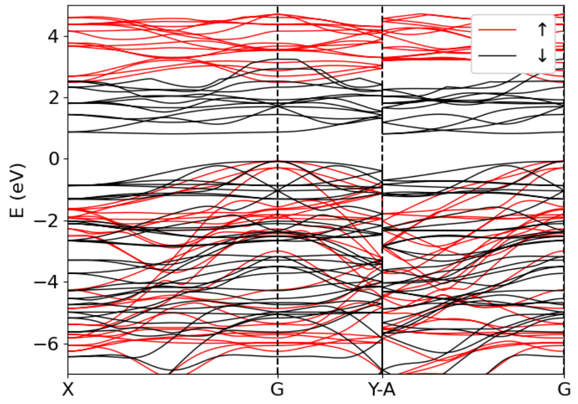


**Electronic bandstructure:** Spin-resolved energy bands of monolayer $V_2NiSe_4$ along a high-symmetry path.

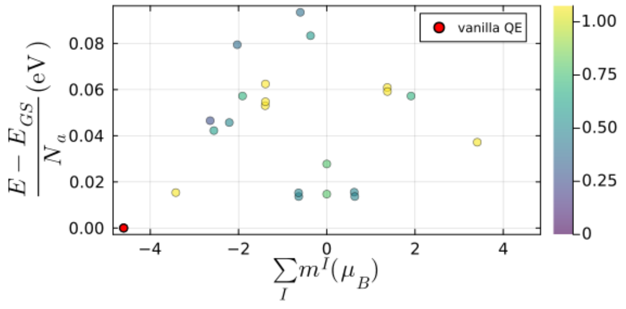


**Unique states:** Representation of 23 self-consistent unique states for monolayer $V_2NiSe_4$ identified using RomeoDFT (see Section S6).

**Lattice vectors:** Cartesian components (in [Å]) of the lattice vectors for $V_2NiSe_4$.

| | $x$ | $y$ | $z$ |
|---|---|---|---|
| $\boldsymbol{a_1}$ | 7.1358 | −0.0001 | 0.0000 |
| $\boldsymbol{a_2}$ | 1.7840 | 3.0899 | 0.0000 |
| $\boldsymbol{a_3}$ | 0.0000 | 0.0000 | 29.0540 |

**Atomic positions:** Fractional coordinates, Hubbard $U$ (in eV) and magnetic moments (in $\mu_B$, computed from orbital occupations $m_o$ or integration spheres $m_i$) of each atom of monolayer $V_2NiSe_4$.

| atom | $x$ | $y$ | $z$ | $U$ | $m_o$ | $m_i$ |
|---|---|---|---|---|---|---|
| V | 0.50 | 1.00 | 0.60 | 4.59 | −2.32 | −2.03 |
| V | 0.50 | 1.00 | 0.40 | 4.59 | −2.32 | −2.03 |
| Ni | 0.50 | 1.00 | 0.50 | 6.39 | 1.39 | 1.49 |
| V | 1.00 | 1.00 | 0.60 | 4.59 | −2.32 | −2.03 |
| V | 1.00 | 1.00 | 0.40 | 4.59 | −2.32 | −2.03 |
| Ni | 1.00 | 1.00 | 0.50 | 6.39 | 1.39 | 1.49 |
| Se | 0.33 | 0.67 | 0.65 | 6.39 | 0.34 | 0.27 |
| Se | 0.17 | 0.33 | 0.55 | 6.39 | 0.13 | 0.14 |
| Se | 0.33 | 0.67 | 0.45 | 6.39 | 0.13 | 0.14 |
| Se | 0.17 | 0.33 | 0.35 | 6.39 | 0.34 | 0.27 |
| Se | 0.83 | 0.67 | 0.65 | 6.39 | 0.34 | 0.27 |
| Se | 0.67 | 0.33 | 0.55 | 6.39 | 0.13 | 0.14 |
| Se | 0.83 | 0.67 | 0.45 | 6.39 | 0.13 | 0.14 |
| Se | 0.67 | 0.33 | 0.35 | 6.39 | 0.34 | 0.27 |

## $VAgP_2Se_6$ (FM)

Band gap: 0.96 eV
Total magnetization: 3.85 $\mu_B/cell$
Absolute magnetization: 5.98 $\mu_B/cell$
MC2D entry: https://mc2d.materialscloud.org/#/details/mc2d-914

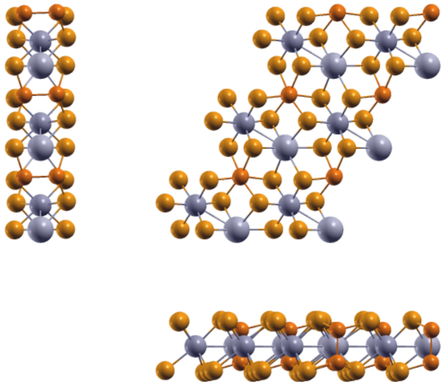

**Geometry:** Views of $VAgP_2Se_6$ as seen from the $x$ axis (left), the $y$ axis (bottom), and the $z$ axis (center).

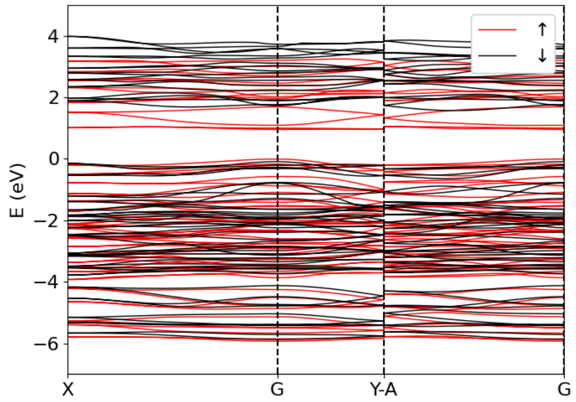


**Electronic bandstructure:** Spin-resolved energy bands of monolayer $VAgP_2Se_6$ along a high-symmetry path.

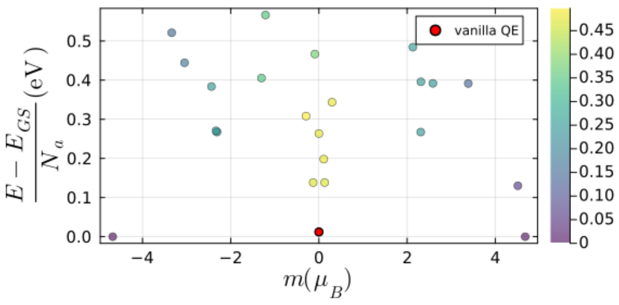


**Unique states:** Representation of 25 self-consistent unique states for monolayer $VAgP_2Se_6$ identified using RomeoDFT (see Section S6).

**Lattice vectors:** Cartesian components (in [Å]) of the lattice vectors for $VAgP_2Se_6$.

| | $x$ | $y$ | $z$ |
|---|---|---|---|
| $a_1$ | 12.7536 | 0.1203 | 0.0000 |
| $a_2$ | 3.2420 | 5.6018 | 0.0000 |
| $a_3$ | 0.0000 | 0.0000 | 23.3990 |

**Atomic positions:** Fractional coordinates, Hubbard $U$ (in eV) and magnetic moments (in $\mu_B$, computed from orbital occupations $m_o$ or integration spheres $m_i$) of each atom of monolayer $VAgP_2Se_6$.

| atom | $x$ | $y$ | $z$ | $U$ | $m_o$ | $m_i$ |
|---|---|---|---|---|---|---|
| V | 0.19 | 0.64 | 0.50 | 3.48 | 2.34 | 2.00 |
| V | 0.69 | 0.64 | 0.50 | 3.48 | 2.34 | 2.00 |
| P | 0.34 | 1.00 | 0.45 | 0.0 | – | 0.01 |
| Se | 0.02 | 0.95 | 0.43 | 0.0 | – | −0.04 |
| Se | 0.18 | 0.35 | 0.43 | 0.0 | – | −0.04 |
| Se | 0.32 | 0.69 | 0.42 | 0.0 | – | −0.05 |
| Ag | −0.01 | 0.35 | 0.50 | 0.0 | – | −0.02 |
| P | 0.34 | 0.99 | 0.55 | 0.0 | – | 0.01 |
| Se | 0.18 | 0.95 | 0.57 | 0.0 | – | −0.04 |
| Se | 0.32 | 0.34 | 0.58 | 0.0 | – | −0.05 |
| Se | 0.02 | 0.67 | 0.57 | 0.0 | – | −0.04 |
| P | 0.84 | 1.00 | 0.45 | 0.0 | – | 0.01 |
| Se | 0.52 | 0.95 | 0.43 | 0.0 | – | −0.04 |
| Se | 0.68 | 0.35 | 0.43 | 0.0 | – | −0.04 |
| Se | 0.82 | 0.69 | 0.42 | 0.0 | – | −0.05 |
| Ag | 0.49 | 0.35 | 0.50 | 0.0 | – | −0.02 |
| P | 0.84 | 0.99 | 0.55 | 0.0 | – | 0.01 |
| Se | 0.68 | 0.95 | 0.57 | 0.0 | – | −0.04 |
| Se | 0.82 | 0.34 | 0.58 | 0.0 | – | −0.05 |
| Se | 0.52 | 0.67 | 0.57 | 0.0 | – | −0.04 |

# $VBr_2$ (AFM)

Band gap: 2.72 eV
Total magnetization: 0.0 $\mu_B/cell$
Absolute magnetization: 6.32 $\mu_B/cell$
MC2D entry: https://mc2d.materialscloud.org/#/details/mc2d-41

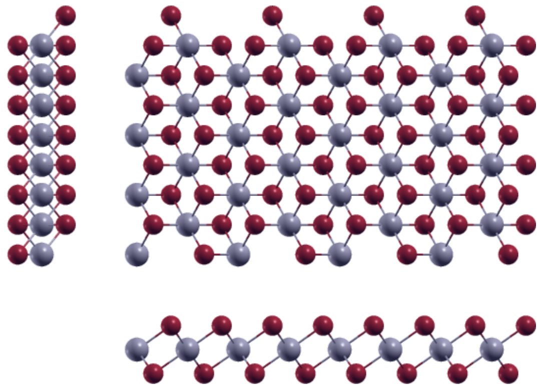

**Geometry:** Views of $VBr_2$ as seen from the $x$ axis (left), the $y$ axis (bottom), and the $z$ axis (center).

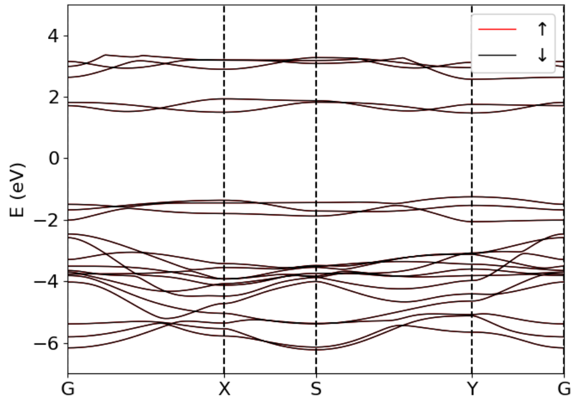


**Electronic bandstructure:** Spin-resolved energy bands of monolayer $VBr_2$ along a high-symmetry path.

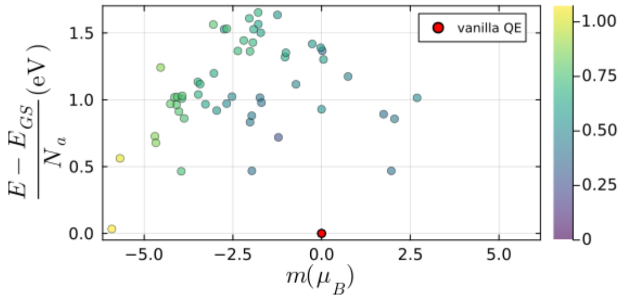


**Unique states:** Representation of 56 self-consistent unique states for monolayer $VBr_2$ identified using RomeoDFT (see Section S6).

**Lattice vectors:** Cartesian components (in [Å]) of the lattice vectors for $VBr_2$.

| | $x$ | $y$ | $z$ |
|---|---|---|---|
| $\boldsymbol{a_1}$ | 0.0000 | −6.3944 | 0.0000 |
| $\boldsymbol{a_2}$ | 3.7686 | 0.0000 | 0.0000 |
| $\boldsymbol{a_3}$ | 0.0000 | 0.0000 | 23.2524 |

**Atomic positions:** Fractional coordinates, Hubbard $U$ (in eV) and magnetic moments (in $\mu_B$, computed from orbital occupations $m_o$ or integration spheres $m_i$) of each atom of monolayer $VBr_2$.

| atom | $x$ | $y$ | $z$ | $U$ | $m_o$ | $m_i$ |
|---|---|---|---|---|---|---|
| V | 0.50 | 0.50 | 0.50 | 2.87 | 2.92 | 2.58 |
| V | 1.00 | 1.00 | 0.50 | 2.87 | −2.92 | −2.58 |
| Br | 0.84 | 0.50 | 0.43 | 0.0 | – | 0.01 |
| Br | 0.34 | 1.00 | 0.43 | 0.0 | – | −0.01 |
| Br | 0.66 | 1.00 | 0.57 | 0.0 | – | −0.01 |
| Br | 1.16 | 0.50 | 0.57 | 0.0 | – | 0.01 |

# $VBr_3$ (FM)

Band gap: 1.26 eV
Total magnetization: $-4.0\ \mu_B/cell$
Absolute magnetization: 4.97 $\mu_B/cell$
MC2D entry: https://mc2d.materialscloud.org/#/details/mc2d-2312

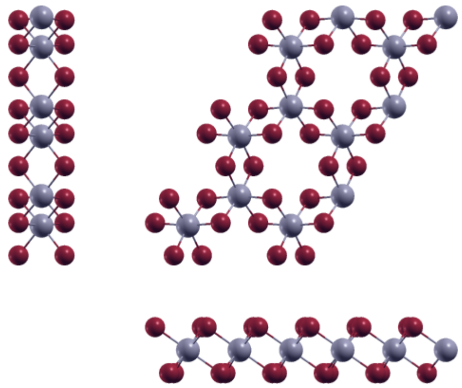

**Geometry:** Views of $VBr_3$ as seen from the $x$ axis (left), the $y$ axis (bottom), and the $z$ axis (center).

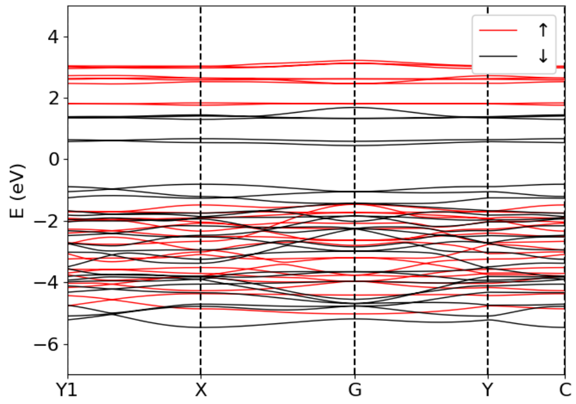


**Electronic bandstructure:** Spin-resolved energy bands of monolayer $VBr_3$ along a high-symmetry path.

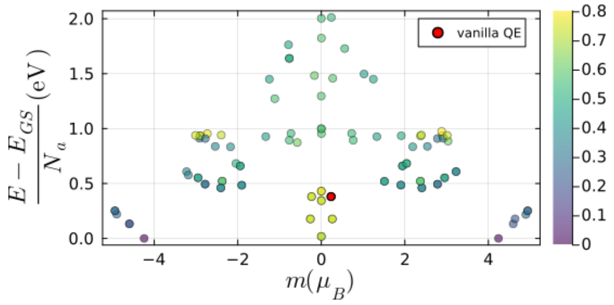


**Unique states:** Representation of 104 self-consistent unique states for monolayer $VBr_3$ identified using RomeoDFT (see Section S6).

**Lattice vectors:** Cartesian components (in [Å]) of the lattice vectors for $VBr_3$.

| | $x$ | $y$ | $z$ |
|---|---|---|---|
| $\boldsymbol{a_1}$ | 3.2642 | 5.6538 | 0.0000 |
| $\boldsymbol{a_2}$ | −3.2642 | 5.6538 | 0.0000 |
| $\boldsymbol{a_3}$ | 0.0000 | 0.0000 | 17.9200 |

**Atomic positions:** Fractional coordinates, Hubbard $U$ (in eV) and magnetic moments (in $\mu_B$, computed from orbital occupations $m_o$ or integration spheres $m_i$) of each atom of monolayer $VBr_3$.

| atom | $x$ | $y$ | $z$ | $U$ | $m_o$ | $m_i$ |
|---|---|---|---|---|---|---|
| V | −0.33 | −0.33 | 0.0 | 3.30 | −2.12 | −1.93 |
| V | −0.67 | 0.33 | 0.0 | 3.30 | −2.12 | −1.93 |
| Br | −1.00 | 0.36 | −0.08 | 0.0 | – | 0.06 |
| Br | −0.36 | 0.36 | 0.08 | 0.0 | – | 0.06 |
| Br | −0.64 | 0.0 | 0.08 | 0.0 | – | 0.06 |
| Br | −1.00 | 0.64 | 0.08 | 0.0 | – | 0.06 |
| Br | −0.64 | 0.64 | −0.08 | 0.0 | – | 0.06 |
| Br | −0.36 | 0.0 | −0.08 | 0.0 | – | 0.06 |

# $VCl_2$ (AFM)

Band gap: 2.82 eV
Total magnetization: $-0.0\ \mu_B/cell$
Absolute magnetization: 6.2 $\mu_B/cell$
MC2D entry: https://mc2d.materialscloud.org/#/details/mc2d-99

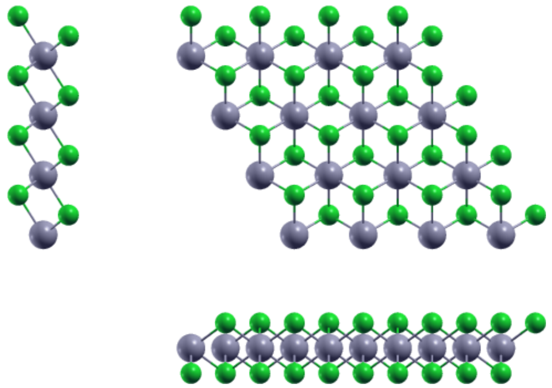

**Geometry:** Views of $VCl_2$ as seen from the $x$ axis (left), the $y$ axis (bottom), and the $z$ axis (center).

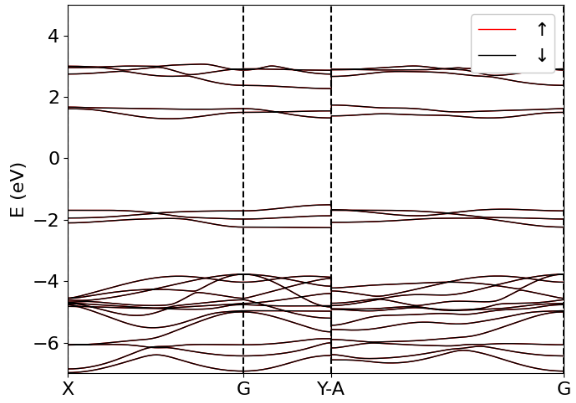


**Electronic bandstructure:** Spin-resolved energy bands of monolayer $VCl_2$ along a high-symmetry path.

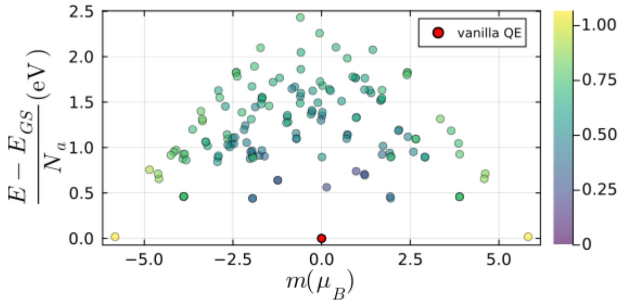


**Unique states:** Representation of 153 self-consistent unique states for monolayer $VCl_2$ identified using RomeoDFT (see Section S6).

**Lattice vectors:** Cartesian components (in [Å]) of the lattice vectors for $VCl_2$.

| | $x$ | $y$ | $z$ |
|---|---|---|---|
| $\boldsymbol{a_1}$ | 7.2959 | 0.0000 | 0.0000 |
| $\boldsymbol{a_2}$ | −1.8240 | 3.1592 | 0.0000 |
| $\boldsymbol{a_3}$ | 0.0000 | 0.0000 | 23.0872 |

**Atomic positions:** Fractional coordinates, Hubbard $U$ (in eV) and magnetic moments (in $\mu_B$, computed from orbital occupations $m_o$ or integration spheres $m_i$) of each atom of monolayer $VCl_2$.

| atom | $x$ | $y$ | $z$ | $U$ | $m_o$ | $m_i$ |
|---|---|---|---|---|---|---|
| V | 0.0 | 0.0 | 0.50 | 2.75 | 2.88 | 2.49 |
| V | 0.50 | 0.0 | 0.50 | 2.75 | −2.88 | −2.49 |
| Cl | 0.17 | 0.67 | 0.56 | 0.0 | – | −0.01 |
| Cl | 0.33 | 0.33 | 0.44 | 0.0 | – | 0.01 |
| Cl | 0.67 | 0.67 | 0.56 | 0.0 | – | 0.01 |
| Cl | 0.83 | 0.33 | 0.44 | 0.0 | – | −0.01 |

## $VCl_2O$ (FM)

Band gap: 0.84 eV
Total magnetization: $-2.01\ \mu_B/cell$
Absolute magnetization: $3.27\ \mu_B/cell$
MC2D entry: https://mc2d.materialscloud.org/#/details/mc2d-96

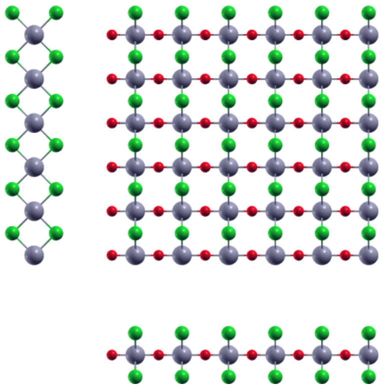

**Geometry:** Views of $VCl_2O$ as seen from the $x$ axis (left), the $y$ axis (bottom), and the $z$ axis (center).

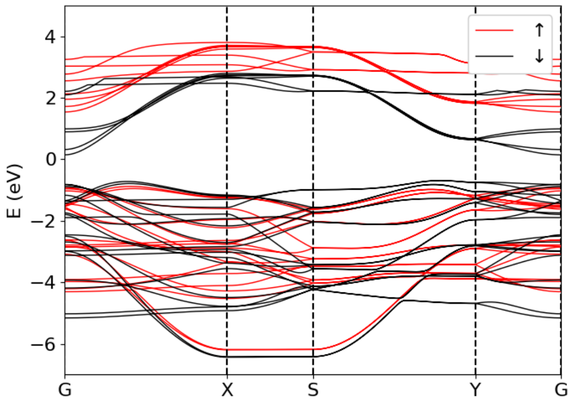


**Electronic bandstructure:** Spin-resolved energy bands of monolayer $VCl_2O$ along a high-symmetry path.

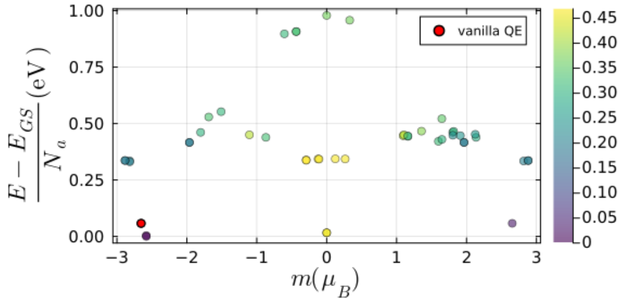


**Unique states:** Representation of 46 self-consistent unique states for monolayer $VCl_2O$ identified using RomeoDFT (see Section S6).

**Lattice vectors:** Cartesian components (in [Å]) of the lattice vectors for $VCl_2O$.

| | $x$ | $y$ | $z$ |
|---|---|---|---|
| $\boldsymbol{a_1}$ | 0.0000 | 3.5998 | 0.0000 |
| $\boldsymbol{a_2}$ | −6.8127 | 0.0000 | 0.0000 |
| $\boldsymbol{a_3}$ | 0.0000 | 0.0000 | 23.4793 |

**Atomic positions:** Fractional coordinates, Hubbard $U$ (in eV) and magnetic moments (in $\mu_B$, computed from orbital occupations $m_o$ or integration spheres $m_i$) of each atom of monolayer $VCl_2O$.

| atom | $x$ | $y$ | $z$ | $U$ | $m_o$ | $m_i$ |
|---|---|---|---|---|---|---|
| V | 0.50 | 0.25 | 0.50 | 3.90 | −1.29 | −0.91 |
| V | 0.50 | 0.75 | 0.50 | 3.90 | −1.29 | −0.91 |
| Cl | 0.50 | 0.50 | 0.43 | 0.0 | – | 0.05 |
| Cl | 0.50 | 1.00 | 0.43 | 0.0 | – | 0.05 |
| Cl | 0.50 | 1.00 | 0.57 | 0.0 | – | 0.05 |
| Cl | 0.50 | 0.50 | 0.57 | 0.0 | – | 0.05 |
| O | 0.0 | 0.25 | 0.50 | 0.0 | – | 0.17 |
| O | 0.0 | 0.75 | 0.50 | 0.0 | – | 0.17 |

# $VCl_3$ (FM)

Band gap: 2.06 eV
Total magnetization: 4.0 $\mu_B/cell$
Absolute magnetization: 4.81 $\mu_B/cell$
MC2D entry: https://mc2d.materialscloud.org/#/details/mc2d-563

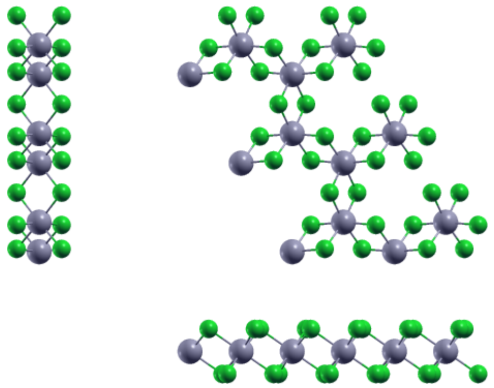

**Geometry:** Views of $VCl_3$ as seen from the $x$ axis (left), the $y$ axis (bottom), and the $z$ axis (center).

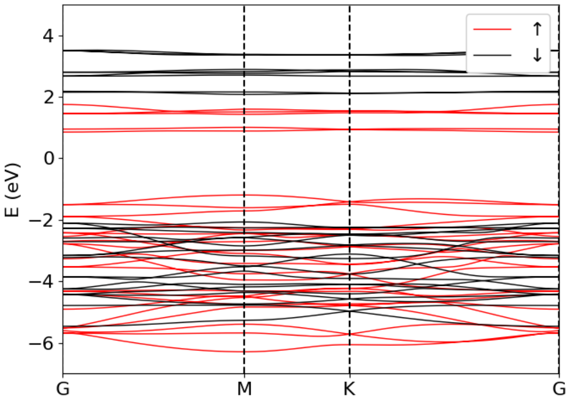


**Electronic bandstructure:** Spin-resolved energy bands of monolayer $VCl_3$ along a high-symmetry path.

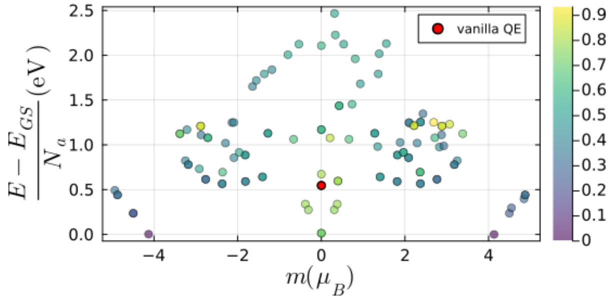


**Unique states:** Representation of 117 self-consistent unique states for monolayer $VCl_3$ identified using RomeoDFT (see Section S6).

**Lattice vectors:** Cartesian components (in [Å]) of the lattice vectors for $VCl_3$.

| | $x$ | $y$ | $z$ |
|---|---|---|---|
| $\boldsymbol{a_1}$ | 6.1503 | 0.0000 | 0.0000 |
| $\boldsymbol{a_2}$ | −3.0752 | 5.3264 | 0.0000 |
| $\boldsymbol{a_3}$ | 0.0000 | 0.0000 | 22.8071 |

**Atomic positions:** Fractional coordinates, Hubbard $U$ (in eV) and magnetic moments (in $\mu_B$, computed from orbital occupations $m_o$ or integration spheres $m_i$) of each atom of monolayer $VCl_3$.

| atom | $x$ | $y$ | $z$ | $U$ | $m_o$ | $m_i$ |
|---|---|---|---|---|---|---|
| V | 0.67 | 0.33 | 0.50 | 4.65 | 2.06 | 1.84 |
| V | 0.0 | 0.0 | 0.50 | 4.65 | 2.06 | 1.84 |
| Cl | 0.70 | 0.67 | 0.56 | 0.0 | – | −0.05 |
| Cl | 0.97 | 0.30 | 0.56 | 0.0 | – | −0.05 |
| Cl | 0.33 | 0.03 | 0.56 | 0.0 | – | −0.05 |
| Cl | 0.97 | 0.67 | 0.44 | 0.0 | – | −0.05 |
| Cl | 0.70 | 0.03 | 0.44 | 0.0 | – | −0.05 |
| Cl | 0.33 | 0.30 | 0.44 | 0.0 | – | −0.05 |

# $VF_4$ (AM)

Band gap: 2.28 eV
Total magnetization: 0.0 $\mu_B/cell$
Absolute magnetization: 2.49 $\mu_B/cell$
MC2D entry: https://mc2d.materialscloud.org/#/details/mc2d-1010

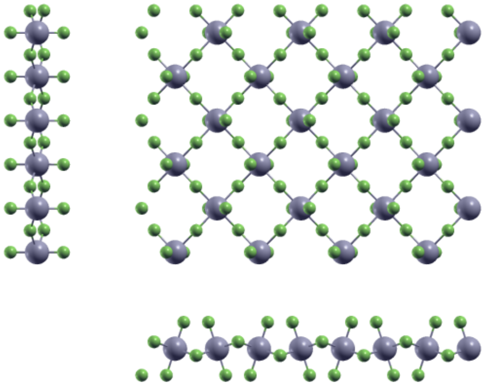

**Geometry:** Views of $VF_4$ as seen from the $x$ axis (left), the $y$ axis (bottom), and the $z$ axis (center).

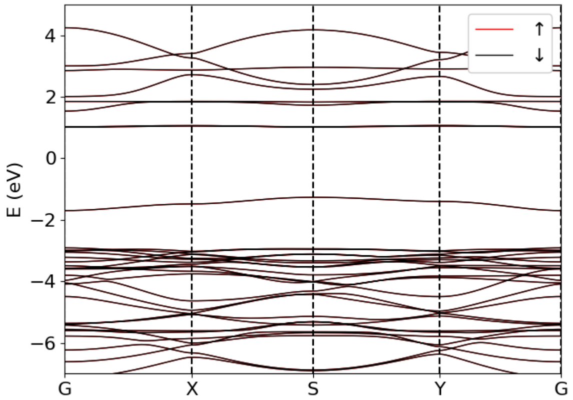


**Electronic bandstructure:** Spin-resolved energy bands of monolayer $VF_4$ along a high-symmetry path.

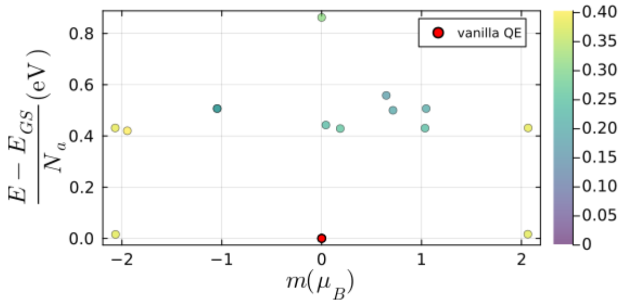


**Unique states:** Representation of 17 self-consistent unique states for monolayer $VF_4$ identified using RomeoDFT (see Section S6).

**Lattice vectors:** Cartesian components (in [Å]) of the lattice vectors for $VF_4$.

| | $x$ | $y$ | $z$ |
|---|---|---|---|
| $\boldsymbol{a_1}$ | 5.2402 | 0.0000 | 0.0000 |
| $\boldsymbol{a_2}$ | 0.0000 | 5.4868 | 0.0000 |
| $\boldsymbol{a_3}$ | 0.0000 | 0.0000 | 23.3319 |

**Atomic positions:** Fractional coordinates, Hubbard $U$ (in eV) and magnetic moments (in $\mu_B$, computed from orbital occupations $m_o$ or integration spheres $m_i$) of each atom of monolayer $VF_4$.

| atom | $x$ | $y$ | $z$ | $U$ | $m_o$ | $m_i$ |
|---|---|---|---|---|---|---|
| V | 1.00 | 0.50 | 0.50 | 3.87 | 1.02 | 0.78 |
| V | 0.50 | 0.00 | 0.50 | 3.87 | −1.02 | −0.78 |
| F | 0.90 | 0.50 | 0.43 | 0.0 | – | −0.05 |
| F | 0.60 | 0.00 | 0.43 | 0.0 | – | 0.05 |
| F | 0.10 | 0.50 | 0.57 | 0.0 | – | −0.05 |
| F | 0.40 | 0.00 | 0.57 | 0.0 | – | 0.05 |
| F | 0.25 | 0.26 | 0.48 | 0.0 | – | 0.00 |
| F | 0.25 | 0.76 | 0.48 | 0.0 | – | 0.00 |
| F | 0.75 | 0.75 | 0.52 | 0.0 | – | 0.0 |
| F | 0.75 | 0.25 | 0.52 | 0.0 | – | 0.0 |

# $VI_2$ (AFM)

Band gap: 2.49 eV
Total magnetization: 0.0 $\mu_B/cell$
Absolute magnetization: 6.57 $\mu_B/cell$
MC2D entry: https://mc2d.materialscloud.org/#/details/mc2d-186

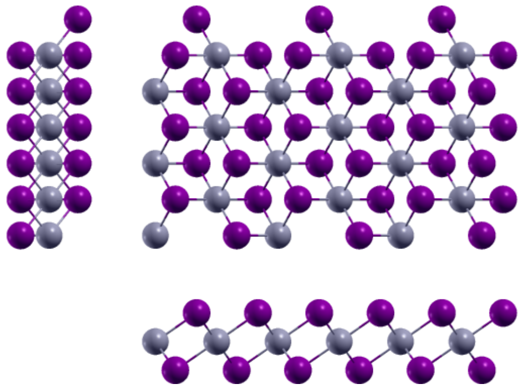

**Geometry:** Views of $VI_2$ as seen from the $x$ axis (left), the $y$ axis (bottom), and the $z$ axis (center).

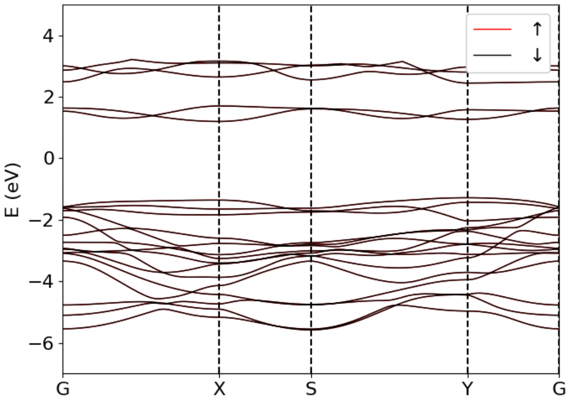


**Electronic bandstructure:** Spin-resolved energy bands of monolayer $VI_2$ along a high-symmetry path.

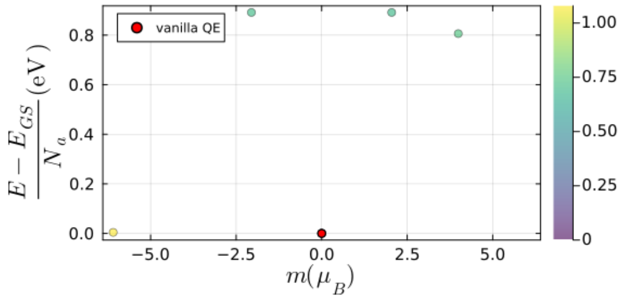


**Unique states:** Representation of 7 self-consistent unique states for monolayer $VI_2$ identified using RomeoDFT (see Section S6).

**Lattice vectors:** Cartesian components (in [Å]) of the lattice vectors for $VI_2$.

| | $x$ | $y$ | $z$ |
|---|---|---|---|
| $\boldsymbol{a_1}$ | 0.0000 | −6.9275 | 0.0000 |
| $\boldsymbol{a_2}$ | 4.0714 | 0.0000 | 0.0000 |
| $\boldsymbol{a_3}$ | 0.0000 | 0.0000 | 23.3511 |

**Atomic positions:** Fractional coordinates, Hubbard $U$ (in eV) and magnetic moments (in $\mu_B$, computed from orbital occupations $m_o$ or integration spheres $m_i$) of each atom of monolayer $VI_2$.

| atom | $x$ | $y$ | $z$ | $U$ | $m_o$ | $m_i$ |
|---|---|---|---|---|---|---|
| V | 0.50 | 0.50 | 0.50 | 2.96 | 3.01 | 2.70 |
| V | 1.00 | 1.00 | 0.50 | 2.96 | −3.01 | −2.70 |
| I | 0.66 | 1.00 | 0.57 | 0.0 | – | −0.02 |
| I | 1.16 | 0.50 | 0.57 | 0.0 | – | 0.02 |
| I | 0.84 | 0.50 | 0.43 | 0.0 | – | 0.02 |
| I | 0.34 | 1.00 | 0.43 | 0.0 | – | −0.02 |

# $VO_3$ (FM)

Band gap: 0.0 eV
Total magnetization: $-2.07\ \mu_B/cell$
Absolute magnetization: $3.21\ \mu_B/cell$
MC2D entry: https://mc2d.materialscloud.org/#/details/mc2d-2475

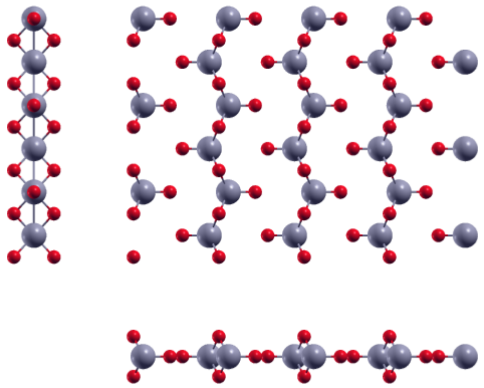

**Geometry:** Views of $VO_3$ as seen from the $x$ axis (left), the $y$ axis (bottom), and the $z$ axis (center).

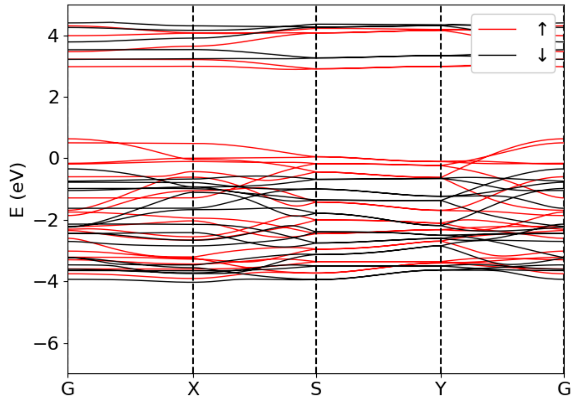


**Electronic bandstructure:** Spin-resolved energy bands of monolayer $VO_3$ along a high-symmetry path.

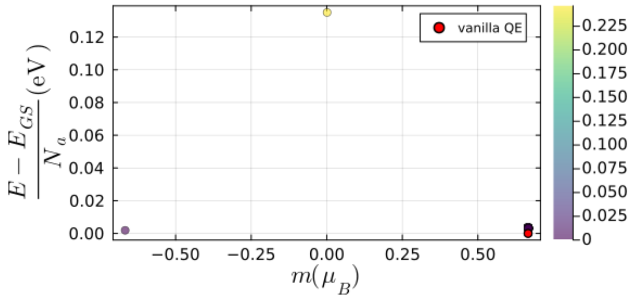


**Unique states:** Representation of 4 self-consistent unique states for monolayer $VO_3$ identified using RomeoDFT (see Section S6).

**Lattice vectors:** Cartesian components (in [Å]) of the lattice vectors for $VO_3$.

| | $x$ | $y$ | $z$ |
|---|---|---|---|
| $a_1$ | 0.0000 | 5.1420 | 0.0000 |
| $a_2$ | −5.2233 | 0.0000 | 0.0000 |
| $a_3$ | 0.0000 | 0.0000 | 16.8948 |

**Atomic positions:** Fractional coordinates, Hubbard $U$ (in eV) and magnetic moments (in $\mu_B$, computed from orbital occupations $m_o$ or integration spheres $m_i$) of each atom of monolayer $VO_3$.

| atom | $x$ | $y$ | $z$ | $U$ | $m_o$ | $m_i$ |
|---|---|---|---|---|---|---|
| V | −0.11 | 0.25 | 0.0 | 4.31 | 0.33 | 0.18 |
| V | 0.11 | 0.75 | 0.0 | 4.31 | 0.33 | 0.18 |
| O | 0.57 | 0.25 | 0.0 | 0.0 | – | −0.29 |
| O | −0.57 | 0.75 | 0.0 | 0.0 | – | −0.29 |
| O | 0.0 | 0.0 | −0.07 | 0.0 | – | −0.36 |
| O | 0.0 | 0.0 | 0.07 | 0.0 | – | −0.36 |
| O | 0.0 | 0.50 | 0.07 | 0.0 | – | −0.36 |
| O | 0.0 | 0.50 | −0.07 | 0.0 | – | −0.36 |

## VOBr (AFM)

Band gap: 1.69 eV
Total magnetization: 0.0 $\mu_B/cell$
Absolute magnetization: 4.4 $\mu_B/cell$
MC2D entry: https://mc2d.materialscloud.org/#/details/mc2d-67

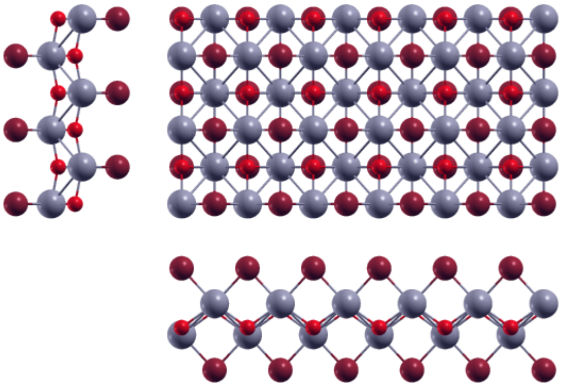

**Geometry:** Views of VOBr as seen from the $x$ axis (left), the $y$ axis (bottom), and the $z$ axis (center).

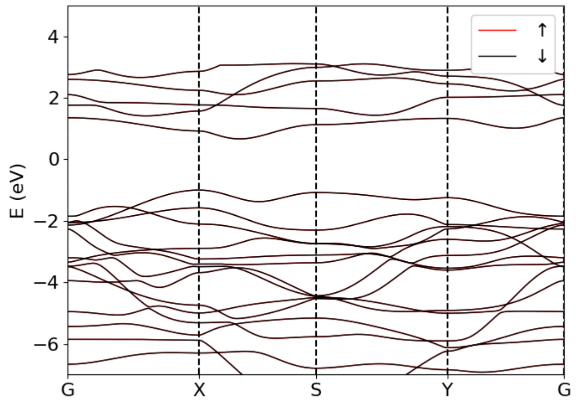


**Electronic bandstructure:** Spin-resolved energy bands of monolayer VOBr along a high-symmetry path.

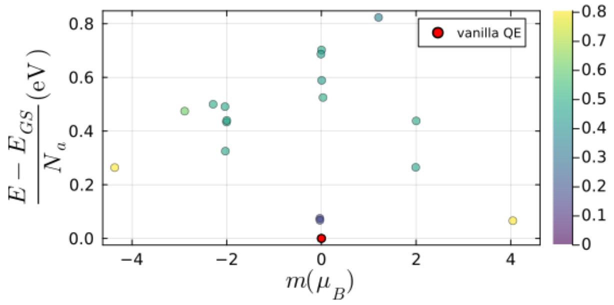


**Unique states:** Representation of 19 self-consistent unique states for monolayer VOBr identified using RomeoDFT (see Section S6).

**Lattice vectors:** Cartesian components (in [Å]) of the lattice vectors for VOBr.

| | $x$ | $y$ | $z$ |
|---|---|---|---|
| $a_1$ | 3.4332 | 0.0000 | 0.0000 |
| $a_2$ | 0.0000 | 3.8558 | 0.0000 |
| $a_3$ | 0.0000 | 0.0000 | 25.2142 |

**Atomic positions:** Fractional coordinates, Hubbard $U$ (in eV) and magnetic moments (in $\mu_B$, computed from orbital occupations $m_o$ or integration spheres $m_i$) of each atom of monolayer VOBr.

| atom | $x$ | $y$ | $z$ | $U$ | $m_o$ | $m_i$ |
|---|---|---|---|---|---|---|
| V | 0.0 | 0.0 | 0.53 | 4.90 | 1.97 | 1.56 |
| V | 0.50 | 0.50 | 0.47 | 4.90 | −1.97 | −1.56 |
| Br | 0.0 | 0.50 | 0.39 | 0.0 | – | 0.05 |
| Br | 0.50 | 0.0 | 0.61 | 0.0 | – | −0.05 |
| O | 0.50 | 0.0 | 0.48 | 0.0 | – | 0.00 |
| O | 0.0 | 0.50 | 0.52 | 0.0 | – | 0.00 |

## $VOBr_2$ (AFM)

Band gap: 0.67 eV
Total magnetization: 0.0 $\mu_B/cell$
Absolute magnetization: 3.58 $\mu_B/cell$
MC2D entry: https://mc2d.materialscloud.org/#/details/mc2d-39

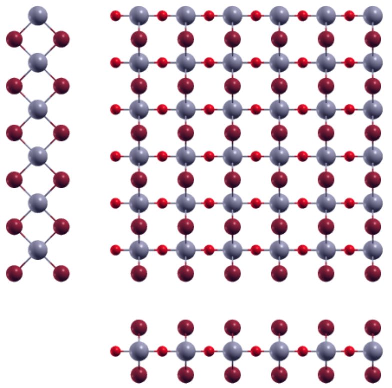

**Geometry:** Views of $VOBr_2$ as seen from the $x$ axis (left), the $y$ axis (bottom), and the $z$ axis (center).

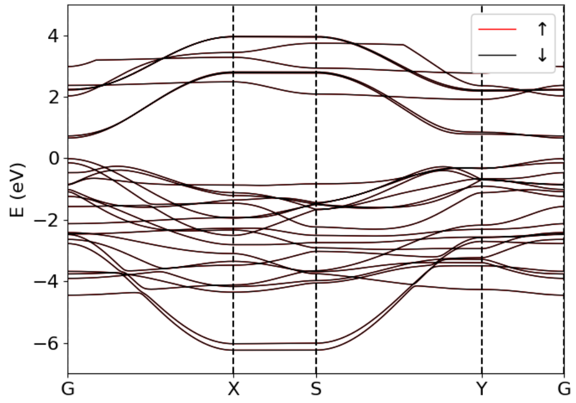


**Electronic bandstructure:** Spin-resolved energy bands of monolayer $VOBr_2$ along a high-symmetry path.

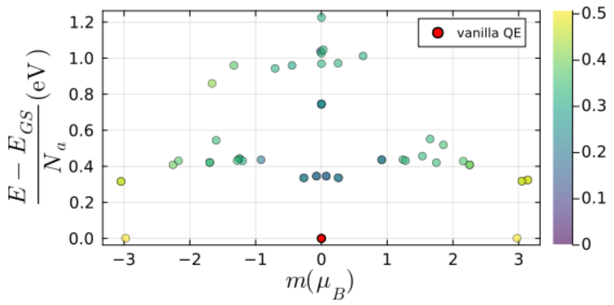


**Unique states:** Representation of 54 self-consistent unique states for monolayer $VOBr_2$ identified using RomeoDFT (see Section S6).

**Lattice vectors:** Cartesian components (in [Å]) of the lattice vectors for $VOBr_2$.

| | $x$ | $y$ | $z$ |
|---|---|---|---|
| $\boldsymbol{a_1}$ | 0.0000 | 3.6099 | 0.0000 |
| $\boldsymbol{a_2}$ | −7.2295 | 0.0000 | 0.0000 |
| $\boldsymbol{a_3}$ | 0.0000 | 0.0000 | 23.7367 |

**Atomic positions:** Fractional coordinates, Hubbard $U$ (in eV) and magnetic moments (in $\mu_B$, computed from orbital occupations $m_o$ or integration spheres $m_i$) of each atom of monolayer $VOBr_2$.

| atom | $x$ | $y$ | $z$ | $U$ | $m_o$ | $m_i$ |
|---|---|---|---|---|---|---|
| V | 0.50 | 0.25 | 0.50 | 3.98 | 1.44 | 0.98 |
| V | 0.50 | 0.75 | 0.50 | 3.98 | −1.44 | −0.98 |
| Br | 0.50 | 0.50 | 0.42 | 0.0 | – | 0.0 |
| Br | 0.50 | 1.00 | 0.42 | 0.0 | – | 0.0 |
| Br | 0.50 | 0.50 | 0.58 | 0.0 | – | 0.0 |
| Br | 0.50 | 1.00 | 0.58 | 0.0 | – | 0.0 |
| O | 0.0 | 0.25 | 0.50 | 0.0 | – | −0.19 |
| O | 0.0 | 0.75 | 0.50 | 0.0 | – | 0.19 |

# VOCl (AFM)

Band gap: 1.84 eV
Total magnetization: 0.0 $\mu_B/cell$
Absolute magnetization: 4.31 $\mu_B/cell$
MC2D entry: https://mc2d.materialscloud.org/#/details/mc2d-118

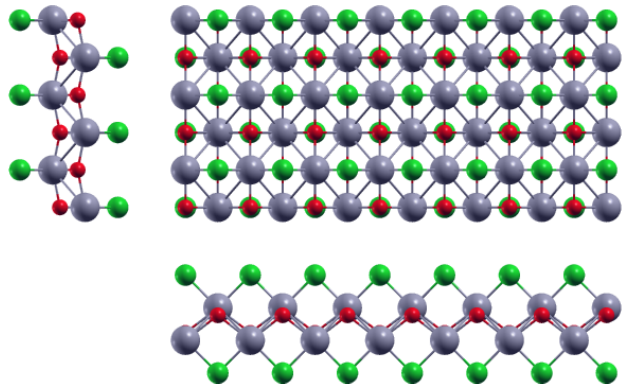

**Geometry:** Views of VOCl as seen from the $x$ axis (left), the $y$ axis (bottom), and the $z$ axis (center).

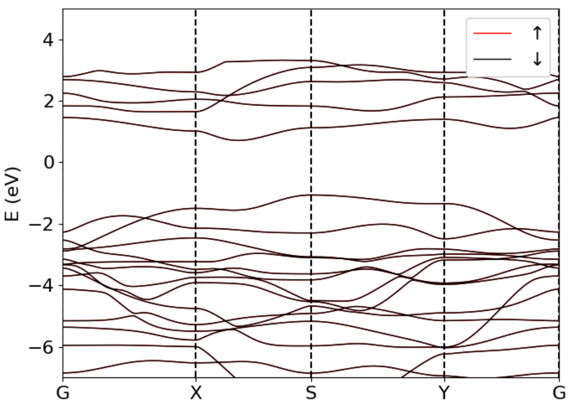


**Electronic bandstructure:** Spin-resolved energy bands of monolayer VOCl along a high-symmetry path.

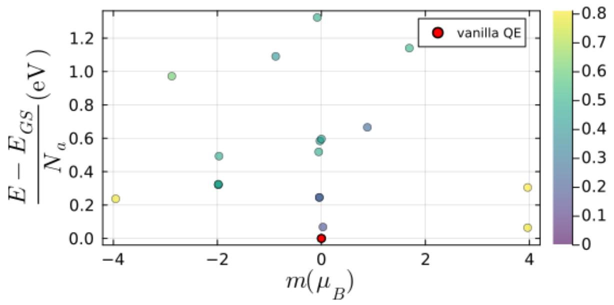


**Unique states:** Representation of 20 self-consistent unique states for monolayer VOCl identified using RomeoDFT (see Section S6).

**Lattice vectors:** Cartesian components (in [Å]) of the lattice vectors for VOCl.

| | $x$ | $y$ | $z$ |
|---|---|---|---|
| $a_1$ | 3.3325 | 0.0000 | 0.0000 |
| $a_2$ | 0.0000 | 3.8489 | 0.0000 |
| $a_3$ | 0.0000 | 0.0000 | 24.8839 |

**Atomic positions:** Fractional coordinates, Hubbard $U$ (in eV) and magnetic moments (in $\mu_B$, computed from orbital occupations $m_o$ or integration spheres $m_i$) of each atom of monolayer VOCl.

| atom | $x$ | $y$ | $z$ | $U$ | $m_o$ | $m_i$ |
|---|---|---|---|---|---|---|
| V | 0.50 | 0.0 | 0.47 | 4.96 | 1.94 | 1.55 |
| V | 0.0 | 0.50 | 0.53 | 4.96 | −1.94 | −1.55 |
| Cl | 0.0 | 0.0 | 0.40 | 0.0 | – | −0.03 |
| O | 0.50 | 0.50 | 0.48 | 0.0 | – | 0.0 |
| Cl | 0.50 | 0.50 | 0.60 | 0.0 | – | 0.03 |
| O | 0.0 | 0.0 | 0.52 | 0.0 | – | 0.0 |

# $VPS_3$ (AFM)

Band gap: 2.18 eV
Total magnetization: 0.0 $\mu_B/cell$
Absolute magnetization: 6.16 $\mu_B/cell$
MC2D entry: https://mc2d.materialscloud.org/#/details/mc2d-1727

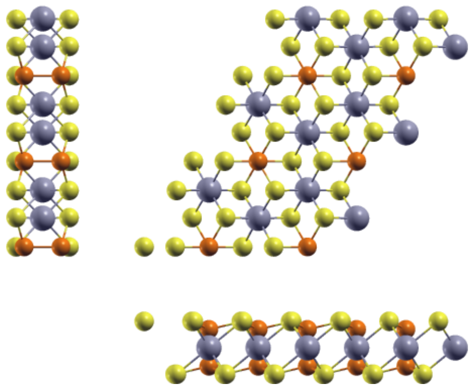

**Geometry:** Views of $VPS_3$ as seen from the $x$ axis (left), the $y$ axis (bottom), and the $z$ axis (center).

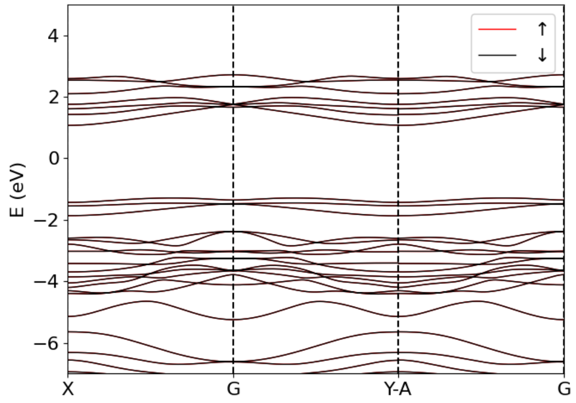


**Electronic bandstructure:** Spin-resolved energy bands of monolayer $VPS_3$ along a high-symmetry path.

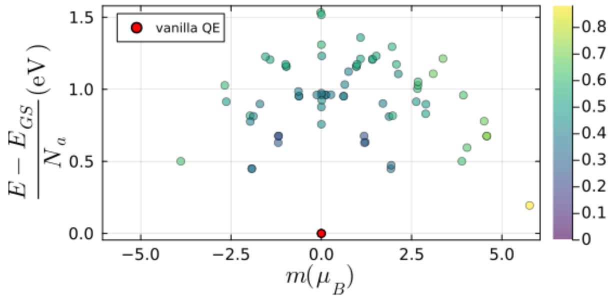


**Unique states:** Representation of 74 self-consistent unique states for monolayer $VPS_3$ identified using RomeoDFT (see Section S6).

**Lattice vectors:** Cartesian components (in [Å]) of the lattice vectors for $VPS_3$.

| | $x$ | $y$ | $z$ |
|---|---|---|---|
| $\boldsymbol{a_1}$ | 5.8628 | 0.0000 | 0.0000 |
| $\boldsymbol{a_2}$ | 2.9314 | 5.0915 | 0.0000 |
| $\boldsymbol{a_3}$ | 0.0000 | 0.0000 | 23.1337 |

**Atomic positions:** Fractional coordinates, Hubbard $U$ (in eV) and magnetic moments (in $\mu_B$, computed from orbital occupations $m_o$ or integration spheres $m_i$) of each atom of monolayer $VPS_3$.

| atom | $x$ | $y$ | $z$ | $U$ | $m_o$ | $m_i$ |
|---|---|---|---|---|---|---|
| V | 1.00 | 0.33 | 0.50 | 3.34 | 2.81 | 2.35 |
| V | 0.33 | 0.67 | 0.50 | 3.34 | −2.81 | −2.35 |
| P | 0.66 | 1.00 | 0.45 | 0.0 | – | 0.0 |
| P | 0.66 | 0.0 | 0.55 | 0.0 | – | 0.0 |
| S | 0.01 | 0.0 | 0.43 | 0.0 | – | 0.0 |
| S | 0.66 | 0.66 | 0.43 | 0.0 | – | 0.0 |
| S | 0.32 | 0.34 | 0.43 | 0.0 | – | 0.0 |
| S | 0.32 | 0.0 | 0.57 | 0.0 | – | 0.0 |
| S | 0.66 | 0.34 | 0.57 | 0.0 | – | 0.0 |
| S | 0.01 | 0.66 | 0.57 | 0.0 | – | 0.0 |

# $VS_2$ (AFM)

Band gap: 0.0 eV
Total magnetization: $-0.0\ \mu_B/cell$
Absolute magnetization: 3.69 $\mu_B/cell$
MC2D entry: https://mc2d.materialscloud.org/#/details/mc2d-233

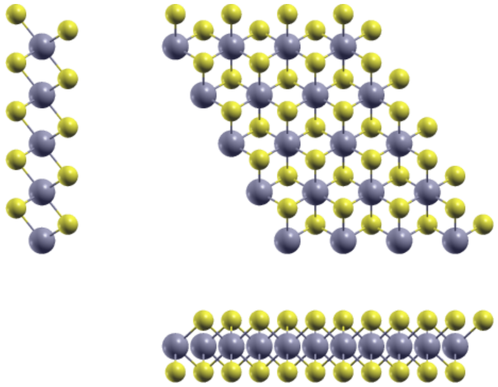

**Geometry:** Views of $VS_2$ as seen from the $x$ axis (left), the $y$ axis (bottom), and the $z$ axis (center).

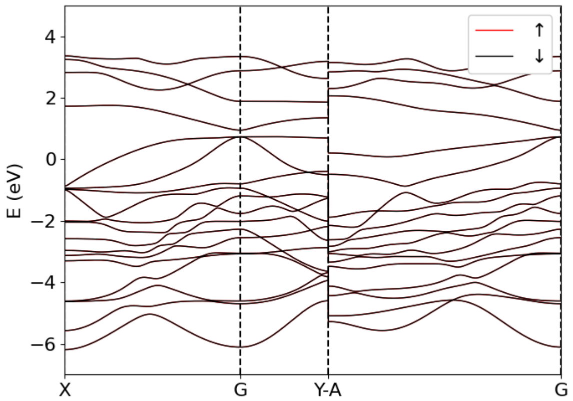


**Electronic bandstructure:** Spin-resolved energy bands of monolayer $VS_2$ along a high-symmetry path.

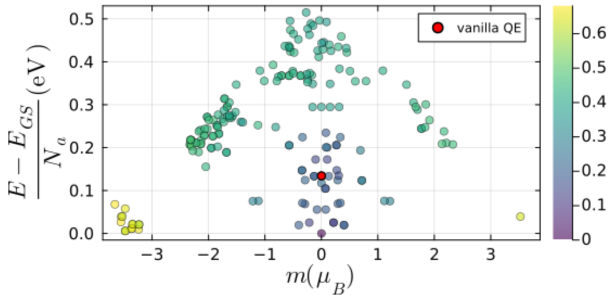


**Unique states:** Representation of 188 self-consistent unique states for monolayer $VS_2$ identified using RomeoDFT (see Section S6).

**Lattice vectors:** Cartesian components (in [Å]) of the lattice vectors for $VS_2$.

| | $x$ | $y$ | $z$ |
|---|---|---|---|
| $\boldsymbol{a_1}$ | 6.3649 | 0.0000 | 0.0000 |
| $\boldsymbol{a_2}$ | −1.5912 | 2.7561 | 0.0000 |
| $\boldsymbol{a_3}$ | 0.0000 | 0.0000 | 22.9395 |

**Atomic positions:** Fractional coordinates, Hubbard $U$ (in eV) and magnetic moments (in $\mu_B$, computed from orbital occupations $m_o$ or integration spheres $m_i$) of each atom of monolayer $VS_2$.

| atom | $x$ | $y$ | $z$ | $U$ | $m_o$ | $m_i$ |
|---|---|---|---|---|---|---|
| V | 0.0 | 0.0 | 0.50 | 4.44 | 1.65 | 1.39 |
| V | 0.50 | 0.0 | 0.50 | 4.44 | −1.65 | −1.39 |
| S | 0.17 | 0.67 | 0.56 | 0.0 | – | −0.05 |
| S | 0.33 | 0.33 | 0.44 | 0.0 | – | 0.05 |
| S | 0.67 | 0.67 | 0.56 | 0.0 | – | 0.05 |
| S | 0.83 | 0.33 | 0.44 | 0.0 | – | −0.05 |

## $VS_2O_8$ (FM)

Band gap: 0.00 eV
Total magnetization: 1.99 $\mu_B/cell$
Absolute magnetization: 4.14 $\mu_B/cell$
MC2D entry: https://mc2d.materialscloud.org/#/details/mc2d-2066

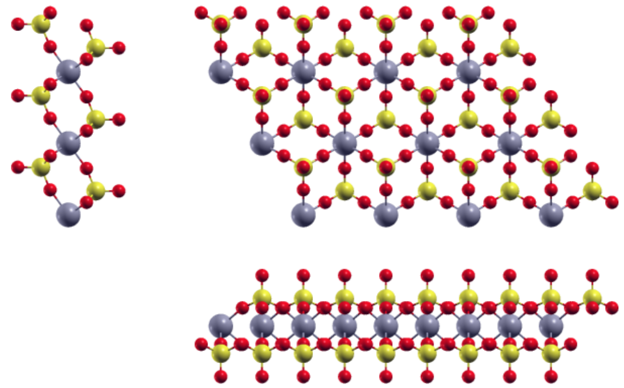

**Geometry:** Views of $VS_2O_8$ as seen from the $x$ axis (left), the $y$ axis (bottom), and the $z$ axis (center).

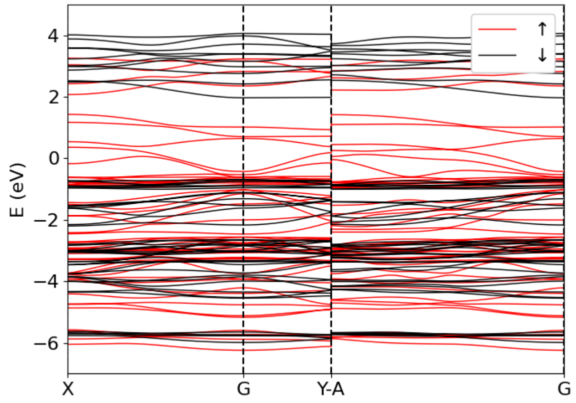


**Electronic bandstructure:** Spin-resolved energy bands of monolayer $VS_2O_8$ along a high-symmetry path.

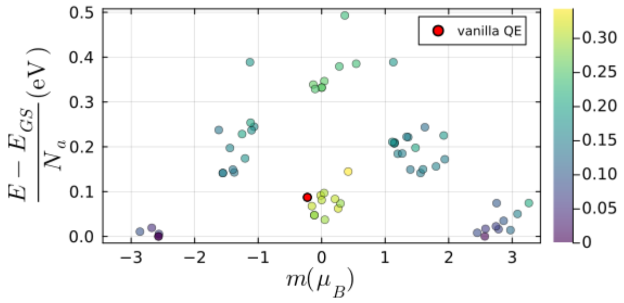


**Unique states:** Representation of 65 self-consistent unique states for monolayer $VS_2O_8$ identified using RomeoDFT (see Section S6).

**Lattice vectors:** Cartesian components (in [Å]) of the lattice vectors for $VS_2O_8$.

| | $x$ | $y$ | $z$ |
|---|---|---|---|
| $\boldsymbol{a_1}$ | 5.0986 | −8.8310 | 0.0000 |
| $\boldsymbol{a_2}$ | 2.5493 | 4.4155 | 0.0000 |
| $\boldsymbol{a_3}$ | 0.0000 | 0.0000 | 24.3646 |

**Atomic positions:** Fractional coordinates, Hubbard $U$ (in eV) and magnetic moments (in $\mu_B$, computed from orbital occupations $m_o$ or integration spheres $m_i$) of each atom of monolayer $VS_2O_8$.

| atom | $x$ | $y$ | $z$ | $U$ | $m_o$ | $m_i$ |
|---|---|---|---|---|---|---|
| V | 0.0 | 0.0 | 0.0 | 4.41 | 1.45 | 1.13 |
| V | 0.50 | 0.0 | 0.0 | 4.41 | 1.45 | 1.13 |
| S | 0.17 | 0.67 | 0.07 | 0.0 | – | 0.00 |
| O | 0.09 | 0.82 | 0.05 | 0.0 | – | −0.05 |
| O | 0.32 | 0.82 | 0.05 | 0.0 | – | −0.06 |
| O | 0.09 | 0.35 | 0.05 | 0.0 | – | −0.05 |
| O | 0.17 | 0.67 | 0.13 | 0.0 | – | −0.03 |
| S | 0.33 | 0.33 | −0.07 | 0.0 | – | 0.00 |
| O | 0.41 | 0.18 | −0.05 | 0.0 | – | −0.05 |
| O | 0.41 | 0.65 | −0.05 | 0.0 | – | −0.05 |
| O | 0.18 | 0.18 | −0.05 | 0.0 | – | −0.06 |
| O | 0.33 | 0.33 | −0.13 | 0.0 | – | −0.03 |
| S | 0.67 | 0.67 | 0.07 | 0.0 | – | 0.00 |
| O | 0.59 | 0.82 | 0.05 | 0.0 | – | −0.05 |
| O | 0.82 | 0.82 | 0.05 | 0.0 | – | −0.06 |
| O | 0.59 | 0.35 | 0.05 | 0.0 | – | −0.05 |
| O | 0.67 | 0.67 | 0.13 | 0.0 | – | −0.03 |
| S | 0.83 | 0.33 | −0.07 | 0.0 | – | 0.00 |
| O | 0.91 | 0.18 | −0.05 | 0.0 | – | −0.05 |
| O | 0.91 | 0.65 | −0.05 | 0.0 | – | −0.05 |
| O | 0.68 | 0.18 | −0.05 | 0.0 | – | −0.06 |
| O | 0.83 | 0.33 | −0.13 | 0.0 | – | −0.03 |

# $VSe_2$ (AFM)

Band gap: 0.0 eV
Total magnetization: $-0.0\ \mu_B/cell$
Absolute magnetization: $4.27\ \mu_B/cell$
MC2D entry: https://mc2d.materialscloud.org/#/details/mc2d-246

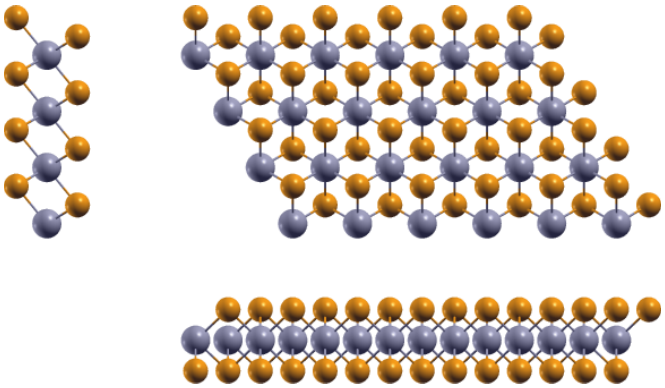

**Geometry:** Views of $VSe_2$ as seen from the $x$ axis (left), the $y$ axis (bottom), and the $z$ axis (center).

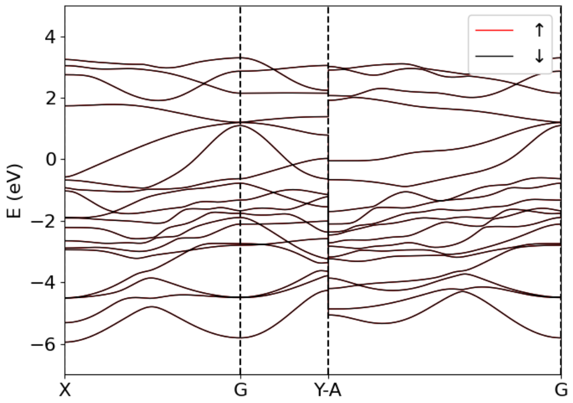


**Electronic bandstructure:** Spin-resolved energy bands of monolayer $VSe_2$ along a high-symmetry path.

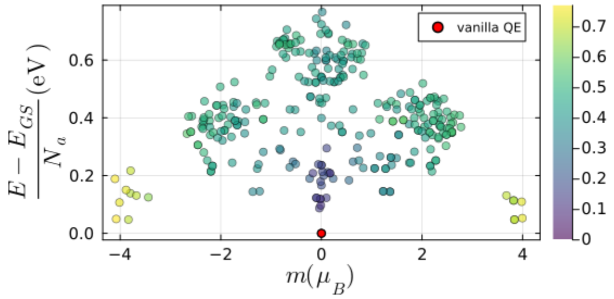


**Unique states:** Representation of 310 self-consistent unique states for monolayer $VSe_2$ identified using RomeoDFT (see Section S6).

**Lattice vectors:** Cartesian components (in [Å]) of the lattice vectors for $VSe_2$.

| | $x$ | $y$ | $z$ |
|---|---|---|---|
| $\boldsymbol{a_1}$ | 6.6729 | 0.0000 | 0.0000 |
| $\boldsymbol{a_2}$ | −1.6682 | 2.8894 | 0.0000 |
| $\boldsymbol{a_3}$ | 0.0000 | 0.0000 | 23.1591 |

**Atomic positions:** Fractional coordinates, Hubbard $U$ (in eV) and magnetic moments (in $\mu_B$, computed from orbital occupations $m_o$ or integration spheres $m_i$) of each atom of monolayer $VSe_2$.

| atom | $x$ | $y$ | $z$ | $U$ | $m_o$ | $m_i$ |
|---|---|---|---|---|---|---|
| V | 0.0 | 0.0 | 0.50 | 4.46 | −1.90 | −1.64 |
| V | 0.50 | 0.0 | 0.50 | 4.46 | 1.90 | 1.64 |
| Se | 0.33 | 0.33 | 0.43 | 0.0 | – | −0.06 |
| Se | 0.17 | 0.67 | 0.57 | 0.0 | – | 0.06 |
| Se | 0.83 | 0.33 | 0.43 | 0.0 | – | 0.06 |
| Se | 0.67 | 0.67 | 0.57 | 0.0 | – | −0.06 |

## $VTe_2$ (AFM)

Band gap: 0.0 eV
Total magnetization: $-0.0\ \mu_B/cell$
Absolute magnetization: $5.08\ \mu_B/cell$
MC2D entry: https://mc2d.materialscloud.org/#/details/mc2d-254

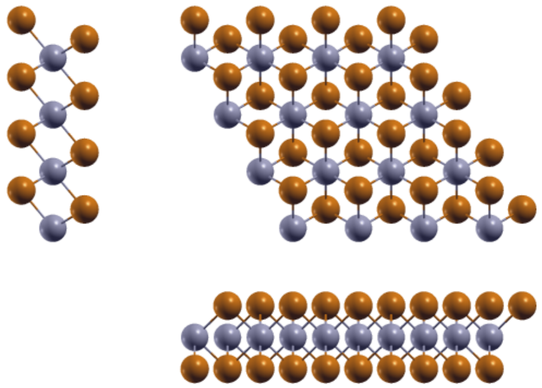

**Geometry:** Views of $VTe_2$ as seen from the $x$ axis (left), the $y$ axis (bottom), and the $z$ axis (center).

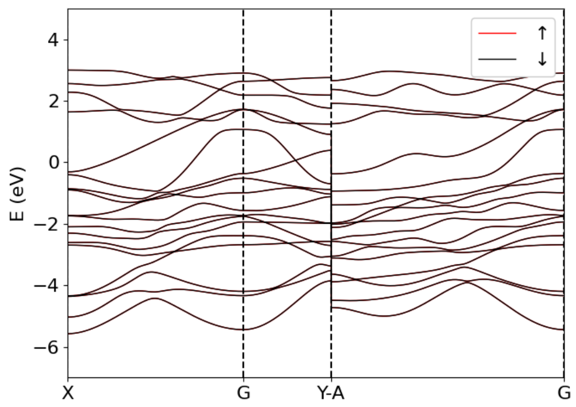


**Electronic bandstructure:** Spin-resolved energy bands of monolayer $VTe_2$ along a high-symmetry path.

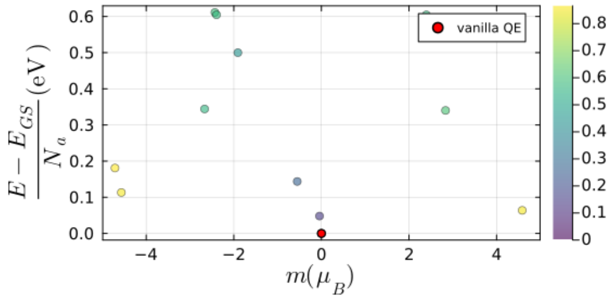


**Unique states:** Representation of 13 self-consistent unique states for monolayer $VTe_2$ identified using RomeoDFT (see Section S6).

**Lattice vectors:** Cartesian components (in [Å]) of the lattice vectors for $VTe_2$.

| | $x$ | $y$ | $z$ |
|---|---|---|---|
| $\boldsymbol{a_1}$ | 7.2110 | 0.0000 | 0.0000 |
| $\boldsymbol{a_2}$ | −1.8028 | 3.1225 | 0.0000 |
| $\boldsymbol{a_3}$ | 0.0000 | 0.0000 | 23.3997 |

**Atomic positions:** Fractional coordinates, Hubbard $U$ (in eV) and magnetic moments (in $\mu_B$, computed from orbital occupations $m_o$ or integration spheres $m_i$) of each atom of monolayer $VTe_2$.

| atom | $x$ | $y$ | $z$ | $U$ | $m_o$ | $m_i$ |
|---|---|---|---|---|---|---|
| V | 0.0 | 0.0 | 0.50 | 4.38 | 2.21 | 1.97 |
| V | 0.50 | 0.0 | 0.50 | 4.38 | −2.21 | −1.97 |
| Te | 0.33 | 0.33 | 0.43 | 0.0 | – | 0.06 |
| Te | 0.17 | 0.67 | 0.57 | 0.0 | – | −0.06 |
| Te | 0.83 | 0.33 | 0.43 | 0.0 | – | −0.06 |
| Te | 0.67 | 0.67 | 0.57 | 0.0 | – | 0.06 |

## $WCuO_4$ (AFM)

Band gap: 1.44 eV
Total magnetization: 0.0 $\mu_B/cell$
Absolute magnetization: 1.85 $\mu_B/cell$
MC2D entry: https://mc2d.materialscloud.org/#/details/mc2d-2552

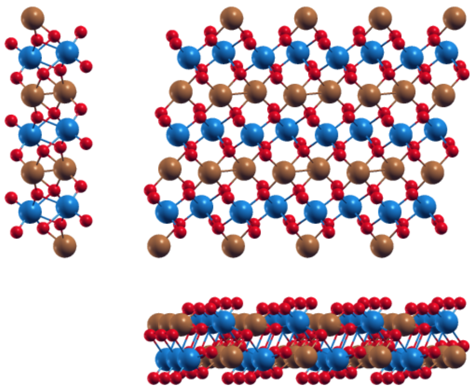

**Geometry:** Views of $WCuO_4$ as seen from the $x$ axis (left), the $y$ axis (bottom), and the $z$ axis (center).

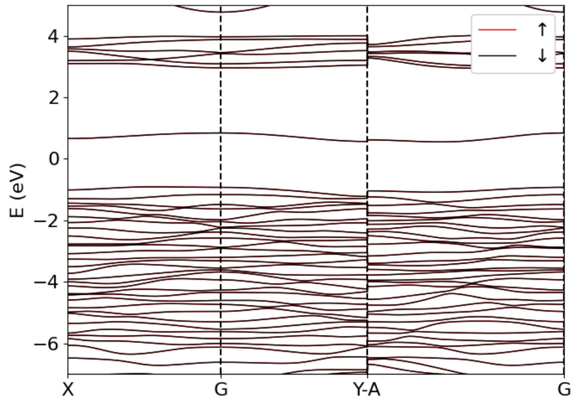


**Electronic bandstructure:** Spin-resolved energy bands of monolayer $WCuO_4$ along a high-symmetry path.

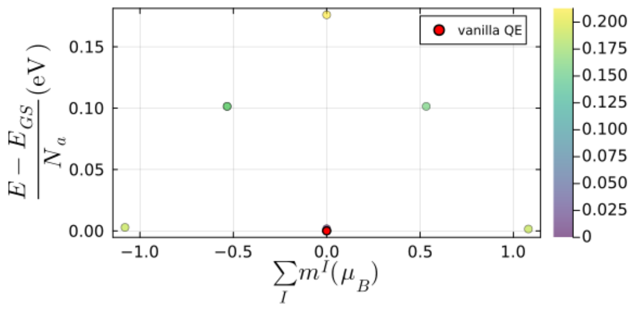


**Unique states:** Representation of 9 self-consistent unique states for monolayer $WCuO_4$ identified using RomeoDFT (see Section S6).

**Lattice vectors:** Cartesian components (in [Å]) of the lattice vectors for $WCuO_4$.

| | $x$ | $y$ | $z$ |
|---|---|---|---|
| $\boldsymbol{a_1}$ | 4.8015 | −0.0163 | 0.0000 |
| $\boldsymbol{a_2}$ | 0.7277 | 4.9774 | 0.0000 |
| $\boldsymbol{a_3}$ | 0.0000 | 0.0000 | 21.2993 |

**Atomic positions:** Fractional coordinates, Hubbard $U$ (in eV) and magnetic moments (in $\mu_B$, computed from orbital occupations $m_o$ or integration spheres $m_i$) of each atom of monolayer $WCuO_4$.

| atom | $x$ | $y$ | $z$ | $U$ | $m_o$ | $m_i$ |
|---|---|---|---|---|---|---|
| Cu | 0.23 | 0.02 | 0.45 | 6.14 | −0.52 | −0.62 |
| Cu | 0.77 | 0.98 | 0.55 | 6.14 | 0.52 | 0.62 |
| W | 0.73 | 0.53 | 0.44 | 3.15 | −0.01 | −0.01 |
| W | 0.27 | 0.47 | 0.56 | 3.15 | 0.01 | 0.01 |
| O | 0.07 | 0.65 | 0.61 | 0.0 | – | 0.02 |
| O | 0.93 | 0.35 | 0.39 | 0.0 | – | −0.02 |
| O | 0.99 | 0.25 | 0.52 | 0.0 | – | −0.02 |
| O | 0.01 | 0.75 | 0.48 | 0.0 | – | 0.02 |
| O | 0.46 | 0.31 | 0.46 | 0.0 | – | −0.05 |
| O | 0.54 | 0.69 | 0.54 | 0.0 | – | 0.05 |
| O | 0.51 | 0.80 | 0.40 | 0.0 | – | −0.06 |
| O | 0.49 | 0.20 | 0.60 | 0.0 | – | 0.06 |

# YBr (FM)

Band gap: 0.0 eV
Total magnetization: 1.77 $\mu_B/cell$
Absolute magnetization: 1.83 $\mu_B/cell$
MC2D entry: https://mc2d.materialscloud.org/#/details/mc2d-2210

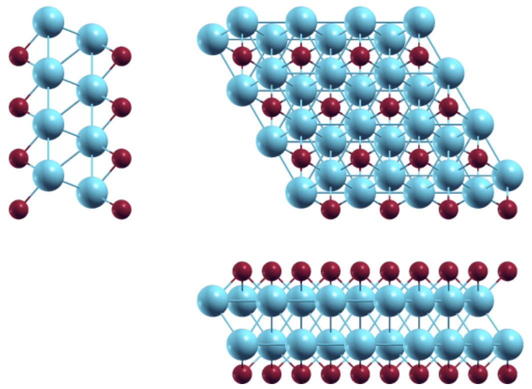

**Geometry:** Views of YBr as seen from the $x$ axis (left), the $y$ axis (bottom), and the $z$ axis (center).

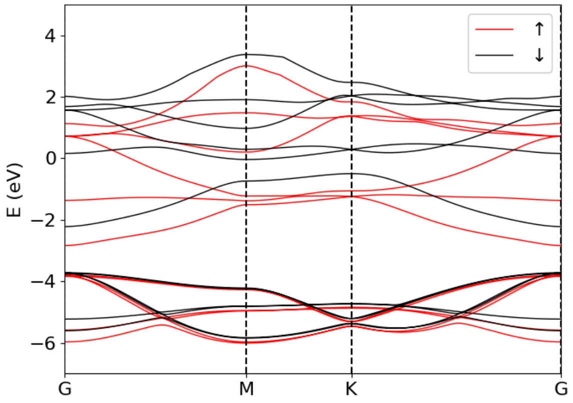


**Electronic bandstructure:** Spin-resolved energy bands of monolayer YBr along a high-symmetry path.

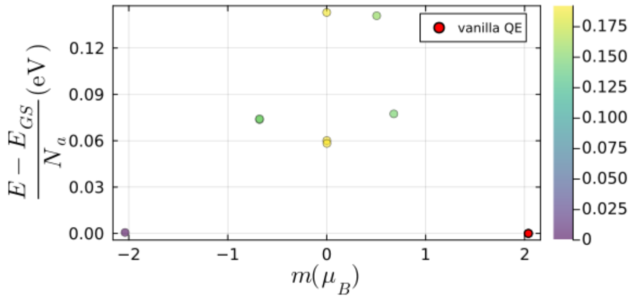


**Unique states:** Representation of 10 self-consistent unique states for monolayer YBr identified using RomeoDFT (see Section S6).

**Lattice vectors:** Cartesian components (in [Å]) of the lattice vectors for YBr.

| | $x$ | $y$ | $z$ |
|---|---|---|---|
| $\boldsymbol{a_1}$ | 1.9104 | −3.3089 | 0.0000 |
| $\boldsymbol{a_2}$ | 1.9104 | 3.3089 | 0.0000 |
| $\boldsymbol{a_3}$ | 0.0000 | 0.0000 | 25.2813 |

**Atomic positions:** Fractional coordinates, Hubbard $U$ (in eV) and magnetic moments (in $\mu_B$, computed from orbital occupations $m_o$ or integration spheres $m_i$) of each atom of monolayer YBr.

| atom | $x$ | $y$ | $z$ | $U$ | $m_o$ | $m_i$ |
|---|---|---|---|---|---|---|
| Y | 0.17 | 0.33 | −0.06 | 1.58 | 1.02 | 0.33 |
| Y | −0.17 | −0.33 | 0.06 | 1.58 | 1.02 | 0.33 |
| Br | 0.50 | 0.0 | −0.13 | 0.0 | – | −0.01 |
| Br | 0.50 | 0.0 | 0.13 | 0.0 | – | −0.01 |

# YCl (FM)

Band gap: 0.0 eV
Total magnetization: 1.79 $\mu_B/cell$
Absolute magnetization: 1.83 $\mu_B/cell$
MC2D entry: https://mc2d.materialscloud.org/#/details/mc2d-125

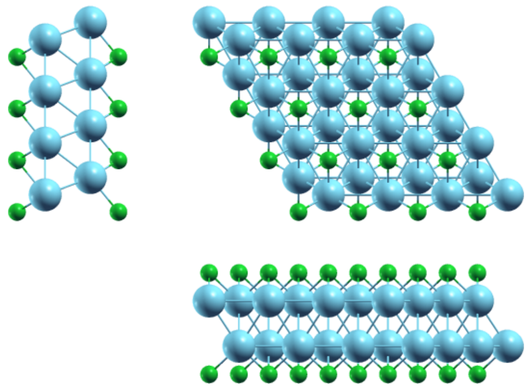

**Geometry:** Views of YCl as seen from the $x$ axis (left), the $y$ axis (bottom), and the $z$ axis (center).

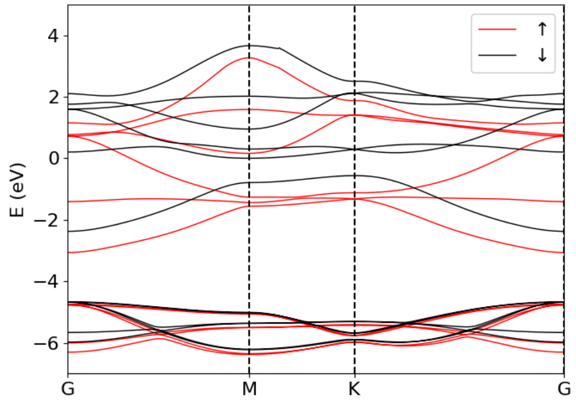


**Electronic bandstructure:** Spin-resolved energy bands of monolayer YCl along a high-symmetry path.

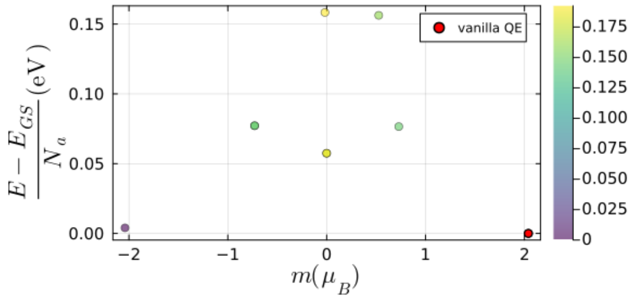


**Unique states:** Representation of 10 self-consistent unique states for monolayer YCl identified using RomeoDFT (see Section S6).

**Lattice vectors:** Cartesian components (in [Å]) of the lattice vectors for YCl.

| | $x$ | $y$ | $z$ |
|---|---|---|---|
| $\boldsymbol{a_1}$ | 3.7382 | 0.0000 | 0.0000 |
| $\boldsymbol{a_2}$ | −1.8691 | 3.2373 | 0.0000 |
| $\boldsymbol{a_3}$ | 0.0000 | 0.0000 | 26.3582 |

**Atomic positions:** Fractional coordinates, Hubbard $U$ (in eV) and magnetic moments (in $\mu_B$, computed from orbital occupations $m_o$ or integration spheres $m_i$) of each atom of monolayer YCl.

| atom | $x$ | $y$ | $z$ | $U$ | $m_o$ | $m_i$ |
|---|---|---|---|---|---|---|
| Y | 0.33 | 0.67 | 0.45 | 1.60 | 1.02 | 0.31 |
| Y | 0.67 | 0.33 | 0.55 | 1.60 | 1.02 | 0.31 |
| Cl | 1.00 | 1.00 | 0.38 | 0.0 | – | 0.00 |
| Cl | 1.00 | 1.00 | 0.62 | 0.0 | – | 0.00 |

# YI (FM)

Band gap: 0.0 eV
Total magnetization: $-1.99\ \mu_B/cell$
Absolute magnetization: $2.07\ \mu_B/cell$
MC2D entry: https://mc2d.materialscloud.org/#/details/mc2d-1794

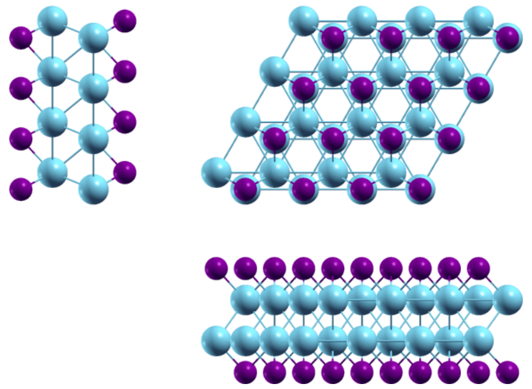

**Geometry:** Views of YI as seen from the $x$ axis (left), the $y$ axis (bottom), and the $z$ axis (center).

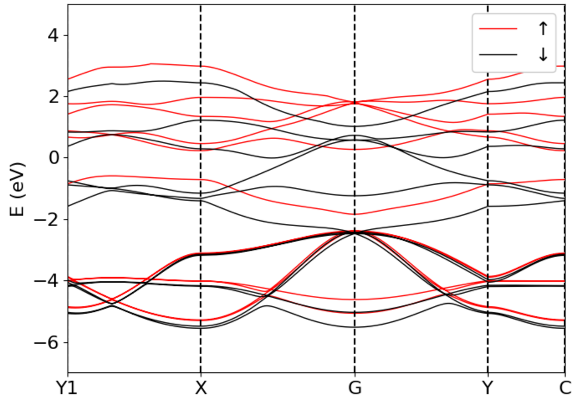


**Electronic bandstructure:** Spin-resolved energy bands of monolayer YI along a high-symmetry path.

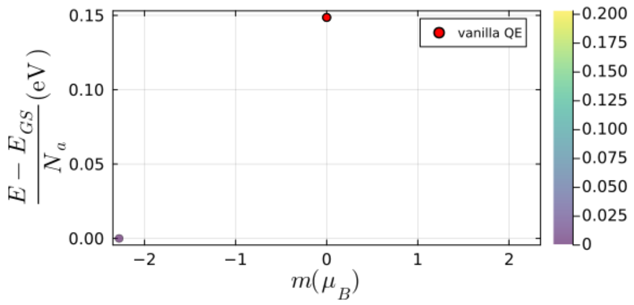


**Unique states:** Representation of 3 self-consistent unique states for monolayer YI identified using RomeoDFT (see Section S6).

**Lattice vectors:** Cartesian components (in [Å]) of the lattice vectors for YI.

| | $x$ | $y$ | $z$ |
|---|---|---|---|
| $\boldsymbol{a_1}$ | 1.9760 | −3.4225 | 0.0000 |
| $\boldsymbol{a_2}$ | 3.9519 | 0.0000 | 0.0000 |
| $\boldsymbol{a_3}$ | 0.0000 | 0.0000 | 26.1729 |

**Atomic positions:** Fractional coordinates, Hubbard $U$ (in eV) and magnetic moments (in $\mu_B$, computed from orbital occupations $m_o$ or integration spheres $m_i$) of each atom of monolayer YI.

| atom | $x$ | $y$ | $z$ | $U$ | $m_o$ | $m_i$ |
|---|---|---|---|---|---|---|
| Y | 0.83 | 0.33 | 0.95 | 1.62 | −1.14 | −0.44 |
| Y | 0.17 | 0.67 | 0.05 | 1.62 | −1.14 | −0.44 |
| I | 0.17 | 0.67 | 0.87 | 0.0 | – | 0.01 |
| I | 0.83 | 0.33 | 0.13 | 0.0 | – | 0.01 |

# $YbBr_3$ (FM)

Band gap: 0.0 eV
Total magnetization: $-2.56\ \mu_B/cell$
Absolute magnetization: $2.62\ \mu_B/cell$
MC2D entry: https://mc2d.materialscloud.org/#/details/mc2d-1902

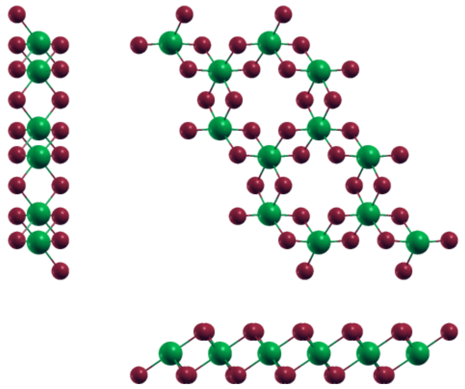

**Geometry:** Views of $YbBr_3$ as seen from the $x$ axis (left), the $y$ axis (bottom), and the $z$ axis (center).

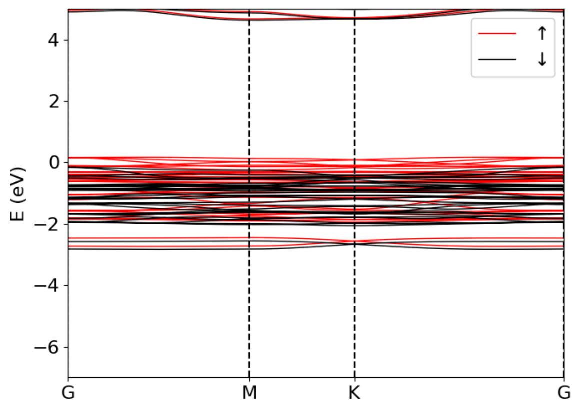


**Electronic bandstructure:** Spin-resolved energy bands of monolayer $YbBr_3$ along a high-symmetry path.

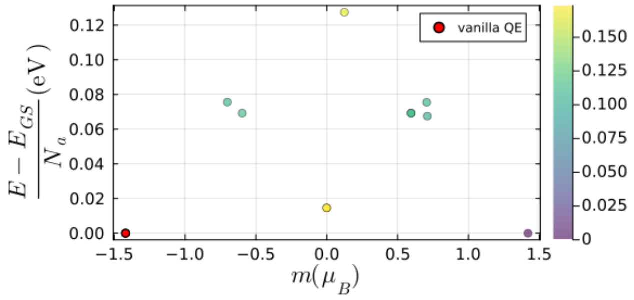


**Unique states:** Representation of 12 self-consistent unique states for monolayer $YbBr_3$ identified using RomeoDFT (see Section S6).

**Lattice vectors:** Cartesian components (in [Å]) of the lattice vectors for $YbBr_3$.

| | $x$ | $y$ | $z$ |
|---|---|---|---|
| $\boldsymbol{a_1}$ | −3.5917 | −6.2209 | 0.0000 |
| $\boldsymbol{a_2}$ | 7.1833 | 0.0000 | 0.0000 |
| $\boldsymbol{a_3}$ | 0.0000 | 0.0000 | 18.2902 |

**Atomic positions:** Fractional coordinates, Hubbard $U$ (in eV) and magnetic moments (in $\mu_B$, computed from orbital occupations $m_o$ or integration spheres $m_i$) of each atom of monolayer $YbBr_3$.

| atom | $x$ | $y$ | $z$ | $U$ | $m_o$ | $m_i$ |
|---|---|---|---|---|---|---|
| Yb | 0.33 | 0.67 | 0.00 | 0.00 | −0.71 | −0.74 |
| Yb | 0.67 | 0.33 | 0.00 | 0.00 | −0.71 | −0.74 |
| Br | 1.00 | 0.64 | 0.09 | 0.0 | – | −0.14 |
| Br | 0.36 | 1.00 | −0.09 | 0.0 | – | −0.14 |
| Br | 0.64 | 0.64 | −0.09 | 0.0 | – | −0.14 |
| Br | 0.00 | 0.36 | −0.09 | 0.0 | – | −0.14 |
| Br | 0.64 | 0.00 | 0.09 | 0.0 | – | −0.14 |
| Br | 0.36 | 0.36 | 0.09 | 0.0 | – | −0.14 |

## $YbCl_3$ (C2/m) (FM)

Band gap: 0.0 eV
Total magnetization: $-2.42\ \mu_B/cell$
Absolute magnetization: $2.47\ \mu_B/cell$
MC2D entry: https://mc2d.materialscloud.org/#/details/mc2d-2184

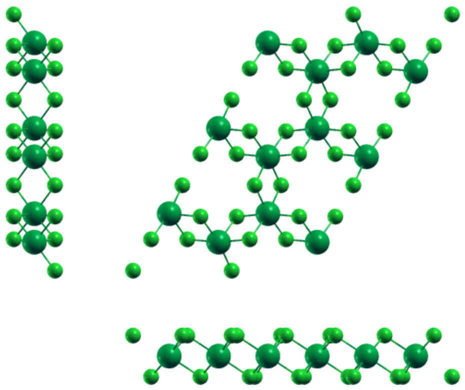

**Geometry:** Views of $YbCl_3$ (C2/m) as seen from the $x$ axis (left), the $y$ axis (bottom), and the $z$ axis (center).

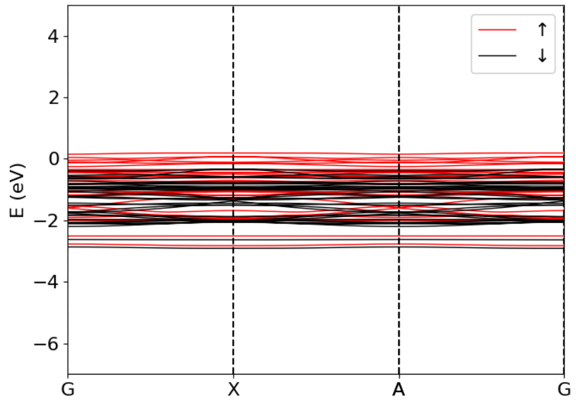


**Electronic bandstructure:** Spin-resolved energy bands of monolayer $YbCl_3$ (C2/m) along a high-symmetry path.

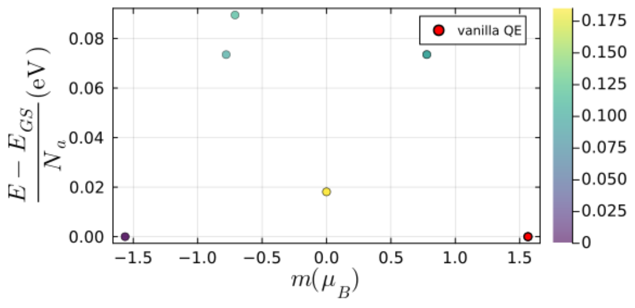


**Unique states:** Representation of 9 self-consistent unique states for monolayer $YbCl_3$ (C2/m) identified using RomeoDFT (see Section S6).

**Lattice vectors:** Cartesian components (in [Å]) of the lattice vectors for $YbCl_3$ (C2/m).

| | $x$ | $y$ | $z$ |
|---|---|---|---|
| $\boldsymbol{a_1}$ | 3.4615 | 5.9916 | 0.0000 |
| $\boldsymbol{a_2}$ | −3.4614 | 5.9915 | 0.0000 |
| $\boldsymbol{a_3}$ | 0.0000 | 0.0000 | 17.8847 |

**Atomic positions:** Fractional coordinates, Hubbard $U$ (in eV) and magnetic moments (in $\mu_B$, computed from orbital occupations $m_o$ or integration spheres $m_i$) of each atom of monolayer $YbCl_3$ (C2/m).

| atom | $x$ | $y$ | $z$ | $U$ | $m_o$ | $m_i$ |
|---|---|---|---|---|---|---|
| Yb | −0.17 | 0.33 | 0.0 | 0.00 | −0.78 | −0.82 |
| Yb | 0.17 | 0.67 | 0.0 | 0.00 | −0.78 | −0.82 |
| Cl | −0.13 | 0.00 | 0.08 | 0.0 | – | −0.11 |
| Cl | 0.50 | 0.63 | 0.08 | 0.0 | – | −0.11 |
| Cl | 0.50 | 0.37 | −0.08 | 0.0 | – | −0.11 |
| Cl | 0.13 | 0.00 | −0.08 | 0.0 | – | −0.11 |
| Cl | 0.13 | 0.37 | 0.08 | 0.0 | – | −0.11 |
| Cl | −0.13 | 0.63 | −0.08 | 0.0 | – | −0.11 |

# $YbCl_3$ (Pmmn) (FM)

Band gap: 0.0 eV
Total magnetization: $-2.25\ \mu_B/cell$
Absolute magnetization: $2.29\ \mu_B/cell$
MC2D entry: https://mc2d.materialscloud.org/#/details/mc2d-1898

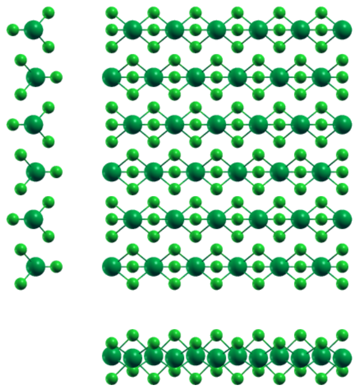

**Geometry:** Views of $YbCl_3$ (Pmmn) as seen from the $x$ axis (left), the $y$ axis (bottom), and the $z$ axis (center).

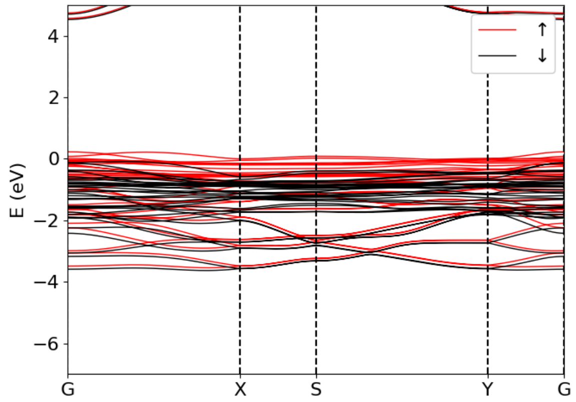


**Electronic bandstructure:** Spin-resolved energy bands of monolayer $YbCl_3$ (Pmmn) along a high-symmetry path.

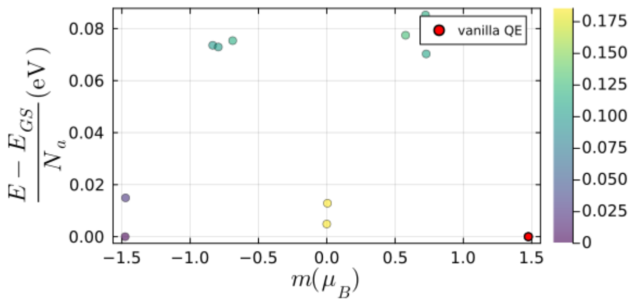


**Unique states:** Representation of 11 self-consistent unique states for monolayer $YbCl_3$ (Pmmn) identified using RomeoDFT (see Section S6).

**Lattice vectors:** Cartesian components (in [Å]) of the lattice vectors for $YbCl_3$ (Pmmn).

| | $x$ | $y$ | $z$ |
|---|---|---|---|
| $\boldsymbol{a_1}$ | 3.7732 | 0.0000 | 0.0000 |
| $\boldsymbol{a_2}$ | 0.0000 | 8.5257 | 0.0000 |
| $\boldsymbol{a_3}$ | 0.0000 | 0.0000 | 19.7647 |

**Atomic positions:** Fractional coordinates, Hubbard $U$ (in eV) and magnetic moments (in $\mu_B$, computed from orbital occupations $m_o$ or integration spheres $m_i$) of each atom of monolayer $YbCl_3$ (Pmmn).

| atom | $x$ | $y$ | $z$ | $U$ | $m_o$ | $m_i$ |
|---|---|---|---|---|---|---|
| Yb | 0.25 | −0.75 | 0.00 | 0.00 | −0.74 | −0.76 |
| Yb | −0.25 | −1.25 | 0.00 | 0.00 | −0.74 | −0.76 |
| Cl | −0.25 | −0.57 | 0.06 | 0.0 | – | −0.09 |
| Cl | −0.25 | −0.93 | 0.06 | 0.0 | – | −0.09 |
| Cl | 0.25 | −1.07 | −0.06 | 0.0 | – | −0.09 |
| Cl | 0.25 | −1.43 | −0.06 | 0.0 | – | −0.09 |
| Cl | −0.25 | −0.75 | −0.10 | 0.0 | – | −0.13 |
| Cl | 0.25 | −1.25 | 0.10 | 0.0 | – | −0.13 |

## $YbI_3$ (FM)

Band gap: 0.0 eV
Total magnetization: $-2.02\ \mu_B/cell$
Absolute magnetization: $2.06\ \mu_B/cell$
MC2D entry: https://mc2d.materialscloud.org/#/details/mc2d-1996

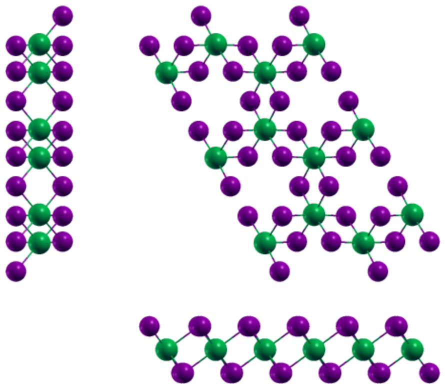

**Geometry:** Views of $YbI_3$ as seen from the $x$ axis (left), the $y$ axis (bottom), and the $z$ axis (center).

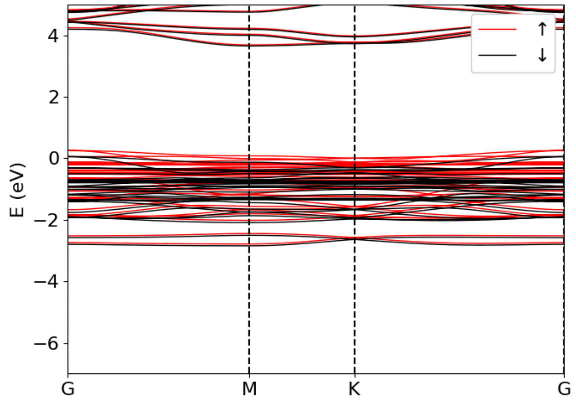


**Electronic bandstructure:** Spin-resolved energy bands of monolayer $YbI_3$ along a high-symmetry path.

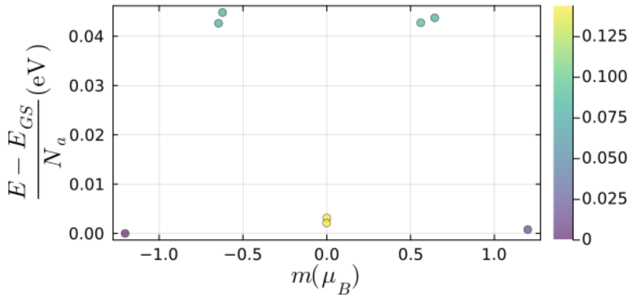


**Unique states:** Representation of 9 self-consistent unique states for monolayer $YbI_3$ identified using RomeoDFT (see Section S6).

**Lattice vectors:** Cartesian components (in [Å]) of the lattice vectors for $YbI_3$.

| | $x$ | $y$ | $z$ |
|---|---|---|---|
| $a_1$ | −3.7701 | −6.5299 | 0.0000 |
| $a_2$ | 7.5401 | 0.0000 | 0.0000 |
| $a_3$ | 0.0000 | 0.0000 | 18.9839 |

**Atomic positions:** Fractional coordinates, Hubbard $U$ (in eV) and magnetic moments (in $\mu_B$, computed from orbital occupations $m_o$ or integration spheres $m_i$) of each atom of monolayer $YbI_3$.

| atom | $x$ | $y$ | $z$ | $U$ | $m_o$ | $m_i$ |
|---|---|---|---|---|---|---|
| Yb | 0.83 | 0.67 | 0.00 | 0.00 | −0.60 | −0.62 |
| Yb | 0.17 | 0.33 | 0.00 | 0.00 | −0.60 | −0.62 |
| I | 0.50 | 0.65 | 0.09 | 0.0 | – | −0.09 |
| I | −0.15 | 0.00 | −0.09 | 0.0 | – | −0.09 |
| I | 0.15 | 0.65 | −0.09 | 0.0 | – | −0.09 |
| I | 0.50 | 0.35 | −0.09 | 0.0 | – | −0.09 |
| I | 0.15 | 0.00 | 0.09 | 0.0 | – | −0.09 |
| I | −0.15 | 0.35 | 0.09 | 0.0 | – | −0.09 |

## YbOBr (AFM)

Band gap: 1.84 eV
Total magnetization: 0.0 $\mu_B/cell$
Absolute magnetization: 2.02 $\mu_B/cell$
MC2D entry: https://mc2d.materialscloud.org/#/details/mc2d-68

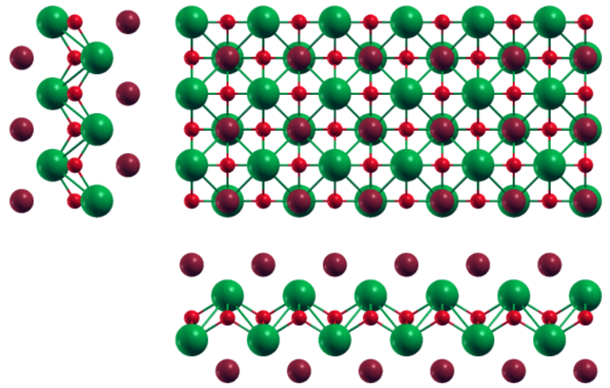

**Geometry:** Views of YbOBr as seen from the $x$ axis (left), the $y$ axis (bottom), and the $z$ axis (center).

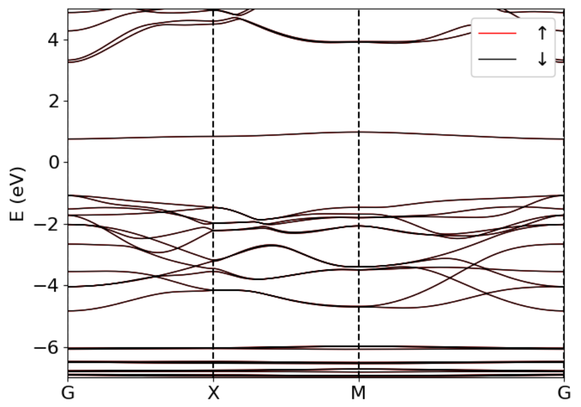


**Electronic bandstructure:** Spin-resolved energy bands of monolayer YbOBr along a high-symmetry path.

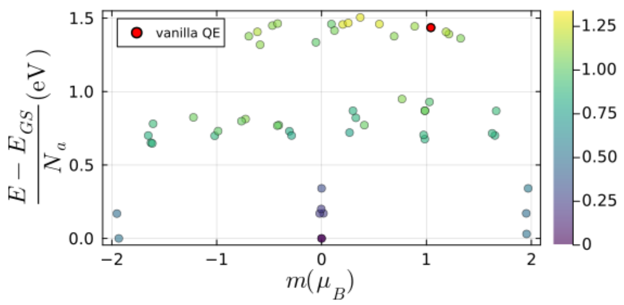


**Unique states:** Representation of 57 self-consistent unique states for monolayer YbOBr identified using RomeoDFT (see Section S6).

**Lattice vectors:** Cartesian components (in [Å]) of the lattice vectors for YbOBr.

| | $x$ | $y$ | $z$ |
|---|---|---|---|
| $a_1$ | 3.7923 | 0.0000 | 0.0000 |
| $a_2$ | 0.0000 | 3.7923 | 0.0000 |
| $a_3$ | 0.0000 | 0.0000 | 25.5929 |

**Atomic positions:** Fractional coordinates, Hubbard $U$ (in eV) and magnetic moments (in $\mu_B$, computed from orbital occupations $m_o$ or integration spheres $m_i$) of each atom of monolayer YbOBr.

| atom | $x$ | $y$ | $z$ | $U$ | $m_o$ | $m_i$ |
|---|---|---|---|---|---|---|
| Yb | 0.0 | 0.50 | 0.55 | 6.78 | −0.97 | −0.98 |
| Yb | 0.50 | 0.0 | 0.45 | 6.78 | 0.97 | 0.98 |
| Br | 0.50 | 0.0 | 0.61 | 0.0 | – | 0.00 |
| Br | 0.0 | 0.50 | 0.39 | 0.0 | – | 0.00 |
| O | 0.50 | 0.50 | 0.50 | 0.0 | – | 0.0 |
| O | 0.0 | 0.0 | 0.50 | 0.0 | – | 0.0 |

# YbOCl (FM)

Band gap: 1.99 eV
Total magnetization: −2.0 $\mu_B/cell$
Absolute magnetization: 2.03 $\mu_B/cell$
MC2D entry: https://mc2d.materialscloud.org/#/details/mc2d-120

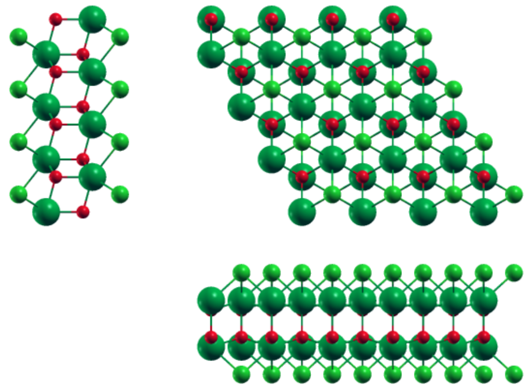

**Geometry:** Views of YbOCl as seen from the $x$ axis (left), the $y$ axis (bottom), and the $z$ axis (center).

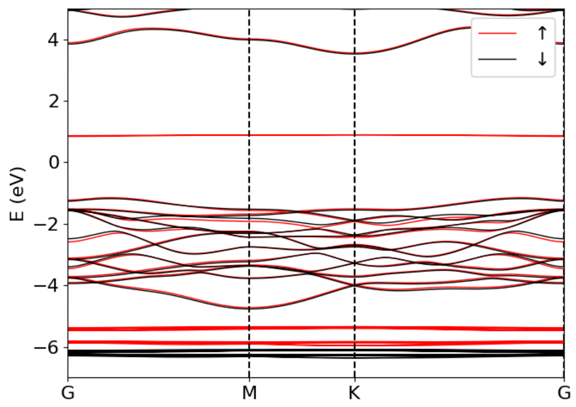


**Electronic bandstructure:** Spin-resolved energy bands of monolayer YbOCl along a high-symmetry path.

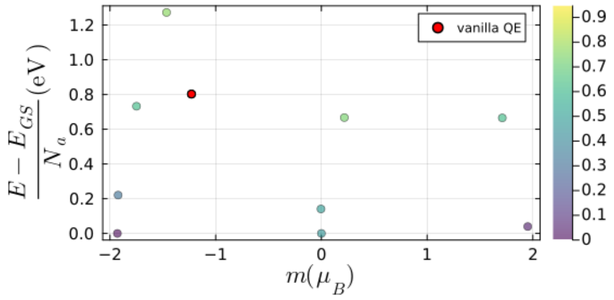


**Unique states:** Representation of 11 self-consistent unique states for monolayer YbOCl identified using RomeoDFT (see Section S6).

**Lattice vectors:** Cartesian components (in [Å]) of the lattice vectors for YbOCl.

| | $x$ | $y$ | $z$ |
|---|---|---|---|
| $a_1$ | 3.7766 | 0.0000 | 0.0000 |
| $a_2$ | −1.8883 | 3.2706 | 0.0000 |
| $a_3$ | 0.0000 | 0.0000 | 26.3254 |

**Atomic positions:** Fractional coordinates, Hubbard $U$ (in eV) and magnetic moments (in $\mu_B$, computed from orbital occupations $m_o$ or integration spheres $m_i$) of each atom of monolayer YbOCl.

| atom | $x$ | $y$ | $z$ | $U$ | $m_o$ | $m_i$ |
|---|---|---|---|---|---|---|
| Yb | 0.33 | 0.67 | 0.45 | 6.15 | −0.97 | −0.98 |
| Yb | 0.0 | 0.0 | 0.55 | 6.15 | −0.97 | −0.98 |
| Cl | 0.67 | 0.33 | 0.38 | 0.0 | – | 0.00 |
| O | 0.33 | 0.67 | 0.53 | 0.0 | – | −0.01 |
| O | 0.0 | 0.0 | 0.47 | 0.0 | – | −0.01 |
| Cl | 0.67 | 0.33 | 0.62 | 0.0 | – | 0.00 |

## YbSBr (FM)

Band gap: 0.62 eV
Total magnetization: 2.0 $\mu_B/cell$
Absolute magnetization: 2.02 $\mu_B/cell$
MC2D entry: https://mc2d.materialscloud.org/#/details/mc2d-2563

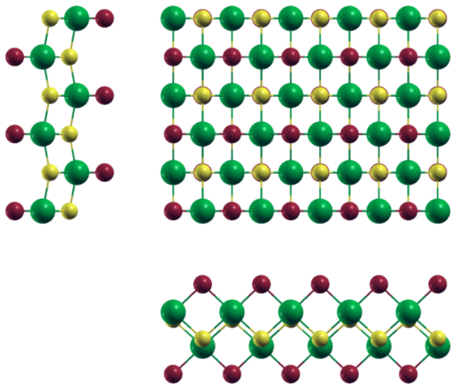

**Geometry:** Views of YbSBr as seen from the $x$ axis (left), the $y$ axis (bottom), and the $z$ axis (center).

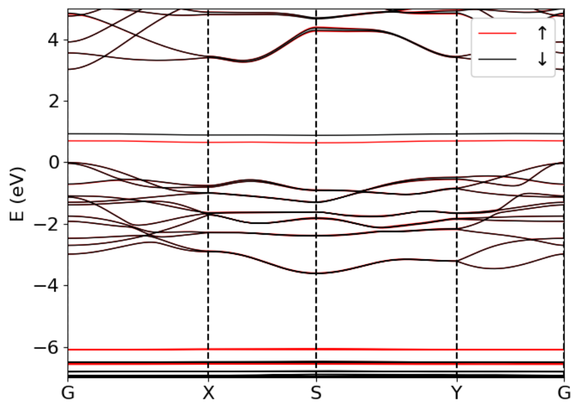


**Electronic bandstructure:** Spin-resolved energy bands of monolayer YbSBr along a high-symmetry path.

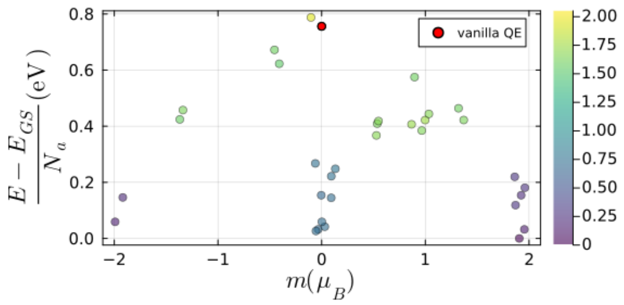


**Unique states:** Representation of 34 self-consistent unique states for monolayer YbSBr identified using RomeoDFT (see Section S6).

**Lattice vectors:** Cartesian components (in [Å]) of the lattice vectors for YbSBr.

| | $x$ | $y$ | $z$ |
|---|---|---|---|
| $\boldsymbol{a_1}$ | 4.0455 | 0.0000 | 0.0000 |
| $\boldsymbol{a_2}$ | 0.0000 | 5.3102 | 0.0000 |
| $\boldsymbol{a_3}$ | 0.0000 | 0.0000 | 24.0902 |

**Atomic positions:** Fractional coordinates, Hubbard $U$ (in eV) and magnetic moments (in $\mu_B$, computed from orbital occupations $m_o$ or integration spheres $m_i$) of each atom of monolayer YbSBr.

| atom | $x$ | $y$ | $z$ | $U$ | $m_o$ | $m_i$ |
|---|---|---|---|---|---|---|
| Yb | 0.75 | 0.25 | 0.05 | 6.86 | 0.94 | 0.96 |
| Yb | 0.25 | 0.75 | 0.95 | 7.41 | 0.96 | 0.98 |
| S | 0.75 | 0.75 | 0.03 | 0.0 | – | 0.01 |
| S | 0.25 | 0.25 | 0.97 | 0.0 | – | 0.02 |
| Br | 0.25 | 0.25 | 0.13 | 0.0 | – | 0.01 |
| Br | 0.75 | 0.75 | 0.87 | 0.0 | – | 0.00 |

# YbSeF (FM)

Band gap: 0.39 eV
Total magnetization: 2.01 $\mu_B/cell$
Absolute magnetization: 2.03 $\mu_B/cell$
MC2D entry: https://mc2d.materialscloud.org/#/details/mc2d-2275

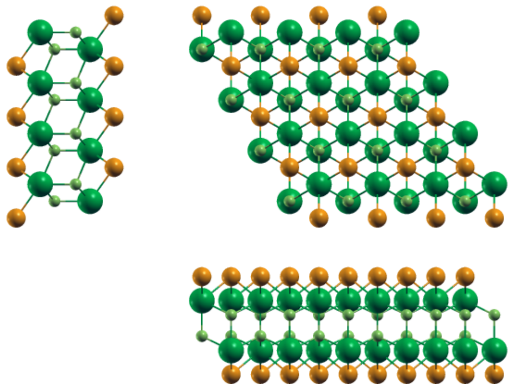

**Geometry:** Views of YbSeF as seen from the $x$ axis (left), the $y$ axis (bottom), and the $z$ axis (center).

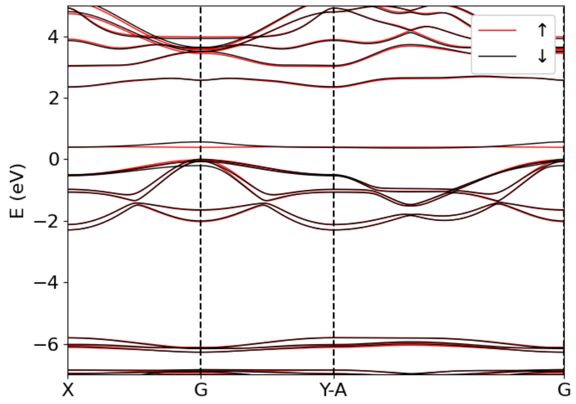


**Electronic bandstructure:** Spin-resolved energy bands of monolayer YbSeF along a high-symmetry path.

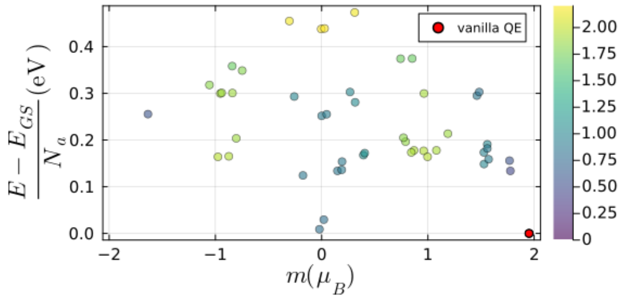


**Unique states:** Representation of 49 self-consistent unique states for monolayer YbSeF identified using RomeoDFT (see Section S6).

**Lattice vectors:** Cartesian components (in [Å]) of the lattice vectors for YbSeF.

| | $x$ | $y$ | $z$ |
|---|---|---|---|
| $\boldsymbol{a_1}$ | 1.9758 | −3.4222 | 0.0000 |
| $\boldsymbol{a_2}$ | 1.9756 | 3.4221 | 0.0000 |
| $\boldsymbol{a_3}$ | 0.0000 | 0.0000 | 25.2711 |

**Atomic positions:** Fractional coordinates, Hubbard $U$ (in eV) and magnetic moments (in $\mu_B$, computed from orbital occupations $m_o$ or integration spheres $m_i$) of each atom of monolayer YbSeF.

| atom | $x$ | $y$ | $z$ | $U$ | $m_o$ | $m_i$ |
|---|---|---|---|---|---|---|
| Yb | 0.17 | 0.33 | −0.07 | 8.13 | 0.98 | 0.98 |
| Yb | 0.83 | 0.67 | 0.07 | 8.13 | 0.98 | 0.98 |
| Se | 0.50 | 0.00 | −0.13 | 0.0 | – | 0.00 |
| F | 0.83 | 0.67 | −0.03 | 0.0 | – | 0.00 |
| Se | 0.50 | 0.00 | 0.13 | 0.0 | – | 0.00 |
| F | 0.17 | 0.33 | 0.03 | 0.0 | – | 0.00 |

## $ZrCl_3$ (FM)

Band gap: 0.30 eV
Total magnetization: −2.0 $\mu_B/cell$
Absolute magnetization: 2.23 $\mu_B/cell$
MC2D entry: https://mc2d.materialscloud.org/#/details/mc2d-424

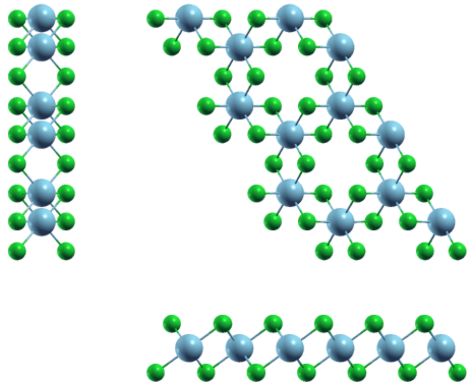

**Geometry:** Views of $ZrCl_3$ as seen from the $x$ axis (left), the $y$ axis (bottom), and the $z$ axis (center).

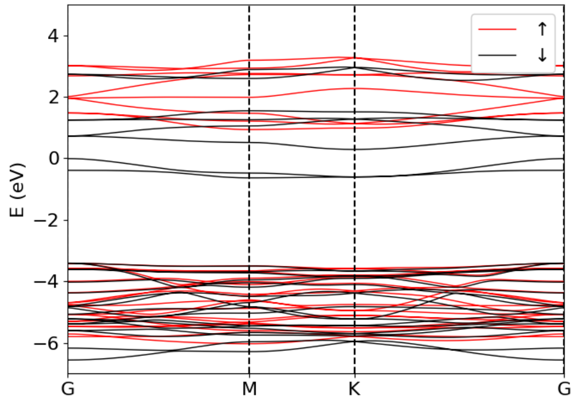


**Electronic bandstructure:** Spin-resolved energy bands of monolayer $ZrCl_3$ along a high-symmetry path.

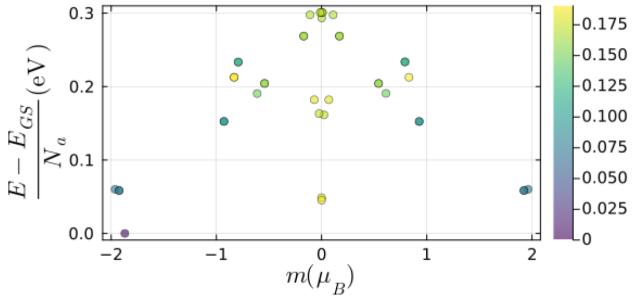


**Unique states:** Representation of 41 self-consistent unique states for monolayer $ZrCl_3$ identified using RomeoDFT (see Section S6).

**Lattice vectors:** Cartesian components (in [Å]) of the lattice vectors for $ZrCl_3$.

| | $x$ | $y$ | $z$ |
|---|---|---|---|
| $\boldsymbol{a_1}$ | 6.2401 | 0.0000 | 0.0000 |
| $\boldsymbol{a_2}$ | −3.1200 | 5.4041 | 0.0000 |
| $\boldsymbol{a_3}$ | 0.0000 | 0.0000 | 23.0292 |

**Atomic positions:** Fractional coordinates, Hubbard $U$ (in eV) and magnetic moments (in $\mu_B$, computed from orbital occupations $m_o$ or integration spheres $m_i$) of each atom of monolayer $ZrCl_3$.

| atom | $x$ | $y$ | $z$ | $U$ | $m_o$ | $m_i$ |
|---|---|---|---|---|---|---|
| Zr | 0.67 | 0.33 | 0.50 | 2.82 | −0.94 | −0.56 |
| Zr | 0.33 | 0.67 | 0.50 | 2.82 | −0.94 | −0.56 |
| Cl | 0.0 | 0.66 | 0.57 | 0.0 | – | 0.00 |
| Cl | 0.34 | 0.34 | 0.57 | 0.0 | – | 0.00 |
| Cl | 0.66 | 0.0 | 0.57 | 0.0 | – | 0.00 |
| Cl | 0.0 | 0.34 | 0.43 | 0.0 | – | 0.00 |
| Cl | 0.66 | 0.66 | 0.43 | 0.0 | – | 0.00 |
| Cl | 0.34 | 0.0 | 0.43 | 0.0 | – | 0.00 |

## $ZrFeCl_6$ (AFM)

Band gap: 2.85 eV
Total magnetization: $-0.0\ \mu_B/cell$
Absolute magnetization: $8.05\ \mu_B/cell$
MC2D entry: https://mc2d.materialscloud.org/#/details/mc2d-489

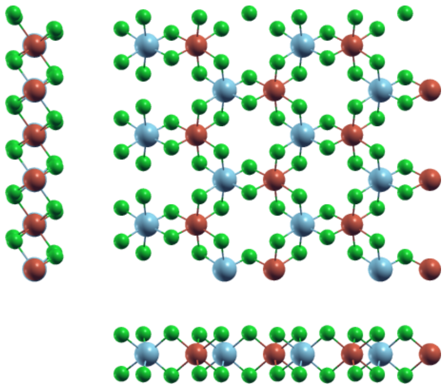

**Geometry:** Views of $ZrFeCl_6$ as seen from the $x$ axis (left), the $y$ axis (bottom), and the $z$ axis (center).

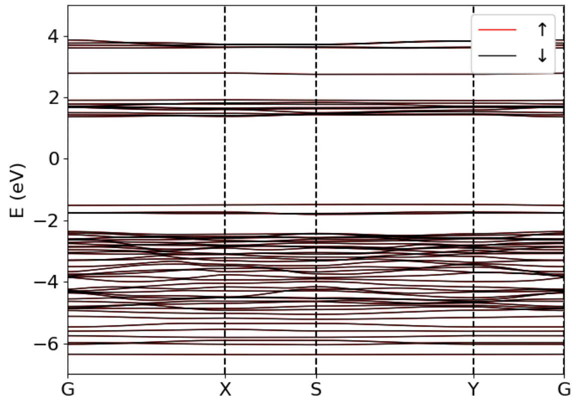


**Electronic bandstructure:** Spin-resolved energy bands of monolayer $ZrFeCl_6$ along a high-symmetry path.

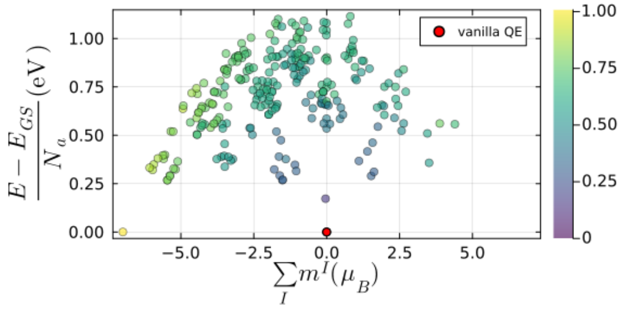


**Unique states:** Representation of 265 self-consistent unique states for monolayer $ZrFeCl_6$ identified using RomeoDFT (see Section S6).

**Lattice vectors:** Cartesian components (in [Å]) of the lattice vectors for $ZrFeCl_6$.

| | $x$ | $y$ | $z$ |
|---|---|---|---|
| $\boldsymbol{a_1}$ | 0.0000 | −11.0064 | 0.0000 |
| $\boldsymbol{a_2}$ | 6.3325 | 0.0000 | 0.0000 |
| $\boldsymbol{a_3}$ | 0.0000 | 0.0000 | 22.7950 |

**Atomic positions:** Fractional coordinates, Hubbard $U$ (in eV) and magnetic moments (in $\mu_B$, computed from orbital occupations $m_o$ or integration spheres $m_i$) of each atom of monolayer $ZrFeCl_6$.

| atom | $x$ | $y$ | $z$ | $U$ | $m_o$ | $m_i$ |
|---|---|---|---|---|---|---|
| Zr | 1.17 | 0.50 | 0.50 | 2.08 | 0.01 | 0.00 |
| Zr | 0.67 | 0.0 | 0.50 | 2.08 | −0.01 | 0.00 |
| Fe | 0.49 | 0.50 | 0.50 | 3.28 | −3.49 | −3.53 |
| Fe | 0.99 | 0.0 | 0.50 | 3.28 | 3.49 | 3.53 |
| Cl | 0.83 | −0.14 | 0.57 | 0.0 | – | 0.03 |
| Cl | 0.33 | 0.36 | 0.57 | 0.0 | – | −0.03 |
| Cl | 1.01 | 0.31 | 0.56 | 0.0 | – | 0.04 |
| Cl | 0.51 | −0.19 | 0.56 | 0.0 | – | −0.04 |
| Cl | 0.65 | 0.32 | 0.56 | 0.0 | – | −0.03 |
| Cl | 1.15 | −0.18 | 0.56 | 0.0 | – | 0.03 |
| Cl | 0.65 | 0.68 | 0.44 | 0.0 | – | −0.03 |
| Cl | 1.15 | 0.18 | 0.44 | 0.0 | – | 0.03 |
| Cl | 1.01 | 0.69 | 0.44 | 0.0 | – | 0.04 |
| Cl | 0.51 | 0.19 | 0.44 | 0.0 | – | −0.04 |
| Cl | 0.83 | 0.14 | 0.43 | 0.0 | – | 0.03 |
| Cl | 0.33 | 0.64 | 0.43 | 0.0 | – | −0.03 |

[1] D. Campi, N. Mounet, M. Gibertini, G. Pizzi, and N. Marzari, ACS Nano **17**, 11268 (2023).
[2] N. Mounet, M. Gibertini, P. Schwaller, D. Campi, A. Merkys, A. Marrazzo, T. Sohier, I. E. Castelli, A. Cepellotti, G. Pizzi, and N. Marzari, Nature Nanotechnology **13**, 246 (2018).
[3] M. A. McGuire, J. Yan, P. Lampen-Kelley, A. F. May, V. R. Cooper, L. Lindsay, A. Puretzky, L. Liang, S. Kc, E. Cakmak, S. Calder, and B. C. Sales, Physical Review Materials **1**, 064001 (2017).
[4] L. Šmejkal, J. Sinova, and T. Jungwirth, Physical Review X **12**, 040501 (2022).
[5] A. Smolyanyuk, L. Šmejkal, and I. I. Mazin, SciPost Physics Codebases , 30 (2024).
[6] J. Sødequist and T. Olsen, Applied Physics Letters **124**, 182409 (2024).
[7] S. Zeng and Y.-J. Zhao, arXiv preprint arXiv:2405.03557 **2405.03557**, âĂŤ (2024).
[8] L. Ponet, E. Di Lucente, and N. Marzari, npj Computational Materials **10**, 151 (2024).
[9] G. Prandini, A. Marrazzo, I. E. Castelli, N. Mounet, and N. Marzari, npj Computational Materials **4**, 72 (2018)
